# Dual inhibitors of CDK2 and TRKA kinases: design, synthesis, antiproliferative activity and molecular modeling of novel pyrazolopyrimidine derivatives

**DOI:** 10.1039/d6ra02521h

**Published:** 2026-09-16

**Authors:** Mohamed H. Attia, Deena S. Lasheen, Nermin Samir, Azza T. Taher, Hatem A. Abdel-Aziz, Dalal A. Abou El Ella

**Affiliations:** a Department of Pharmaceutical Chemistry, Faculty of Pharmacy, October 6 University 6 of October City Giza Egypt mohammed_attia@o6u.edu.eg; b Pharmaceutical Chemistry Department, Faculty of Pharmacy, Ain Shams University Abbassia 11566 Cairo Egypt dalal@pharma.asu.edu.eg; c Department of Pharmaceutical Organic Chemistry Department, Faculty of Pharmacy, Cairo University Cairo Egypt; d Department of Pharmaceutical Organic Chemistry, Faculty of Pharmacy, October 6 University (O6U) Giza Egypt; e Department of Applied Organic Chemistry, National Research Center Dokki Cairo 12622 Egypt; f Department of Pharmaceutical Chemistry, Faculty of Pharmacy, Pharos University in Alexandria Canal El Mahmoudia St. Alexandria 21648 Egypt

## Abstract

Simultaneous inhibition of CDK2 and TRKA presents a promising therapeutic avenue for cancer treatment, capitalizing on the critical roles of CDK2 in cell cycle regulation and TRKA in tumor cell survival and progression. In continuation of our previous work on dual CDK2/TRKA inhibitors, two series of pyrazolo[1,5-*a*]pyrimidines (6a–j, 9a–h) and pyrazolo[3,4-*d*]pyrimidines (11–13) comprising a total of 21 compounds were developed and assessed for their antiproliferative potential across 60 cancer cell lines of NCI. Among them, compound 9e exhibited broad-spectrum anticancer activity, inhibiting growth across all 60 cell lines with an overall mean %GI of 36.91%, showing maximal inhibition against SNB-75. The most active compounds were further evaluated for kinase inhibition. Compounds 6j and 6i displayed potent dual activity, with inhibitory potency (IC_50_) of 0.33 and 0.34 µM for CDK2, and 0.078 and 0.16 µM for TRKA, respectively. Both also showed strong antiproliferative effects against SNB-75, with IC_50_ values of 23.40 µM (6j) and 7.39 µM (6i) and against K562, with IC_50_ values of 42.28 µM (6j) and 14.08 µM (6i) and exhibiting a superior safety profile over Quercetin in normal fibroblasts. Cell cycle analysis of compounds 6i and 6j revealed significant arrest in the G2/M stage. Compound 6i induced the highest apoptosis (35.02%), dominated by late apoptosis (22.07%), and the highest necrosis level (7.10%), indicating more aggressive or less selective cytotoxicity. Overall, cell death was predominantly apoptotic, with limited necrotic contribution. Complementary docking studies confirmed that pyrazolopyrimidines adopted binding conformations similar to reference inhibitors within CDK2 and TRKA active sites. These findings highlight compounds 6i and 6j as promising dual CDK2/TRKA inhibitors for further development as anticancer therapeutics.

## Abstract 2

Simultaneous inhibition of CDK2 and TRKA presents a promising therapeutic avenue for cancer treatment, capitalizing on the critical roles of CDK2 in cell cycle regulation and TRKA in tumor cell survival and progression.

## Graphical abstract

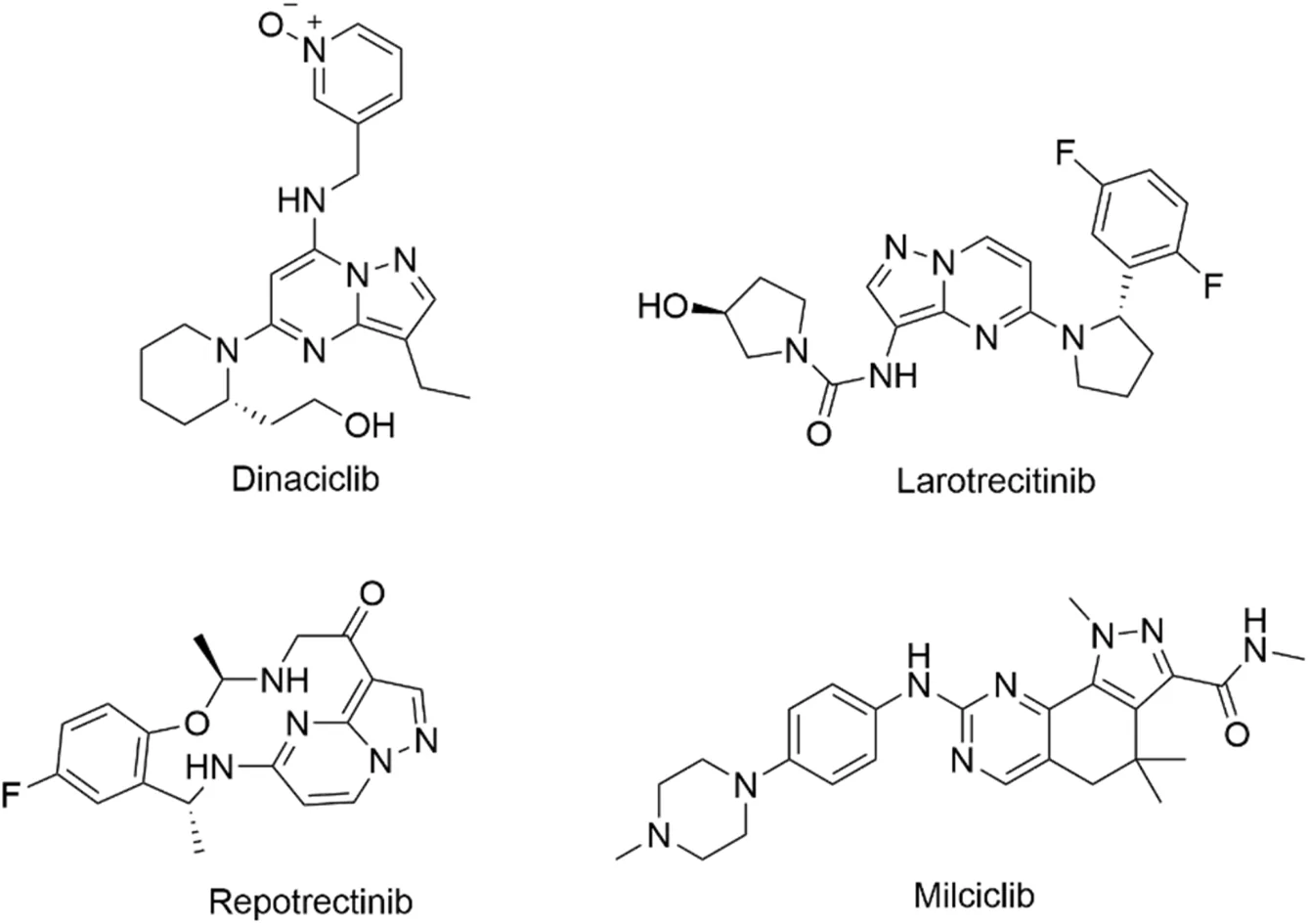

## 1 Introduction

Protein kinases catalyze phosphate transfer from ATP to target substrates, yielding ADP and site-specific phosphorylation. The human kinome contains nearly 634 kinases, nearly 500 of which belong to the eukaryotic protein kinase family.^1,2^

By simultaneously targeting two kinases, dual inhibitors block redundant oncogenic pathways and limit resistance. They may offer enhanced efficacy and duration with lower toxicity compared to combination regimens.^3,4^

Cyclin-dependent kinase 2 (CDK2) drives cell-cycle progression, especially the G1/S transition and DNA replication, *via* binding to cyclins E and A.^5^ Dysregulated CDK2 has been reported in several malignancies, such as breast, ovarian, lung, endometrial, and thyroid cancers, as well as melanoma and osteosarcoma, highlighting its potential as an anticancer target.^6–10^

Pyrazolo[1,5-*a*]pyrimidine-based inhibitors, such as Dinaciclib, exhibit potent CDK2 inhibition *via* ATP-competitive binding within the kinase active site (Fig. 1).^11^

**Fig. 1:**
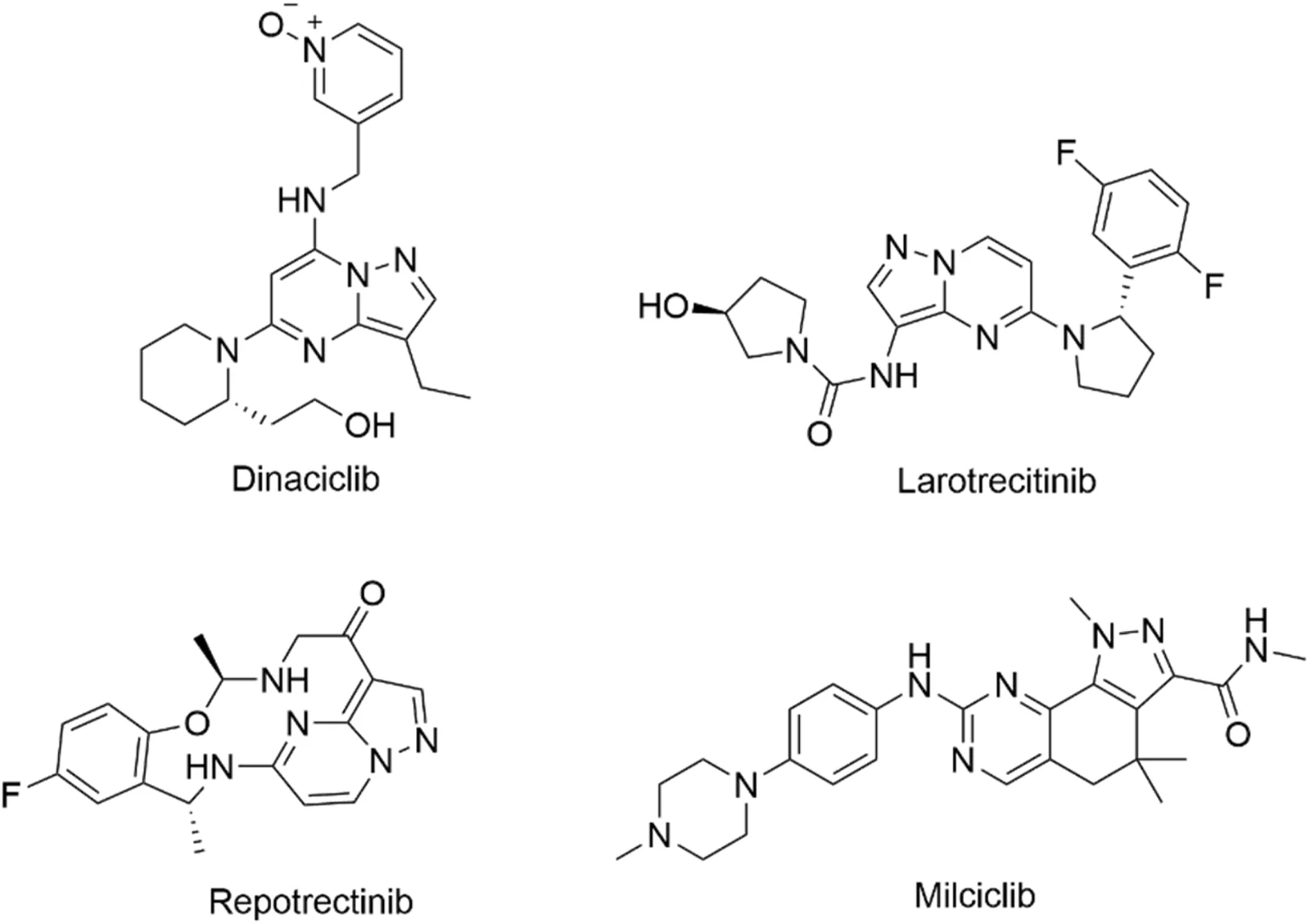
Structures of highly effective inhibitors targeting CDK2 and TRKA proteins.^11,17,18,22,23^

Tropomyosin receptor kinase A (TRKA), encoded by NTRK1, is a validated cancer target. Beyond oncogenic NTRK1 fusions, wild-type TRKA overexpression drives tumor progression, proliferation, survival, migration, and metastasis by activating PI3K/AKT and RAS/MAPK pathways.^12–16^

Clinically approved TRK inhibitors, such as Larotrectinib and Repotrectinib, validate TRK as a therapeutic target (Fig. 1).^17,18^ Milciclib has also gained attention for its potent dual inhibition of CDK2 and TRKA, supporting the feasibility of developing dual-target kinase inhibitors (Fig. 1).^19–21^

Based on our previous work showing that pyrazolopyrimidine derivatives I and II have promising dual CDK2/TRKA inhibitory activity, the pyrazolopyrimidine scaffold was selected as the core pharmacophore for further optimization to enhance dual kinase inhibition and antiproliferative activity.^24^

The design strategy focused on preserving the pyrazolopyrimidine nucleus while systematically modifying the substituents attached to the C-7 aryl moiety of the pyrazolo[1,5-*a*]pyrimidine scaffold (6a–j, 9a–h). A diverse set of substituents was introduced, including electron-donating groups (*e.g.*, methyl and methoxy), electron-withdrawing groups (*e.g.*, chloro and nitro), and heteroaryl rings such as furan, to probe the effects of electronic, steric, and hydrogen-bonding properties on binding to the ATP pockets of CDK2 and TRKA.

A second series of pyrazolo[3,4-*d*]pyrimidines 11–13 with comparable substituents was also designed to assess the impact of scaffold topology and ring fusion on kinase inhibition and antiproliferative activity.

Furthermore, selected derivatives bearing mono- and dihalogenated phenyl substituents were incorporated to evaluate the contribution of electron-withdrawing halogens to hydrophobic interactions within the kinase active sites. Likewise, heteroaryl-containing analogues were designed to explore the possibility of establishing additional hydrogen-bonding interactions with key amino acid residues lining the catalytic pocket.

Accordingly, the designed compounds were synthesized and biologically evaluated through antiproliferative screening, dual CDK2/TRKA kinase inhibition assays, mechanistic investigations, molecular docking, and *in silico* ADME studies. This systematic approach aimed to establish structure–activity relationships and identify promising dual CDK2/TRKA anticancer leads (Fig. 2).

**Fig. 2:**
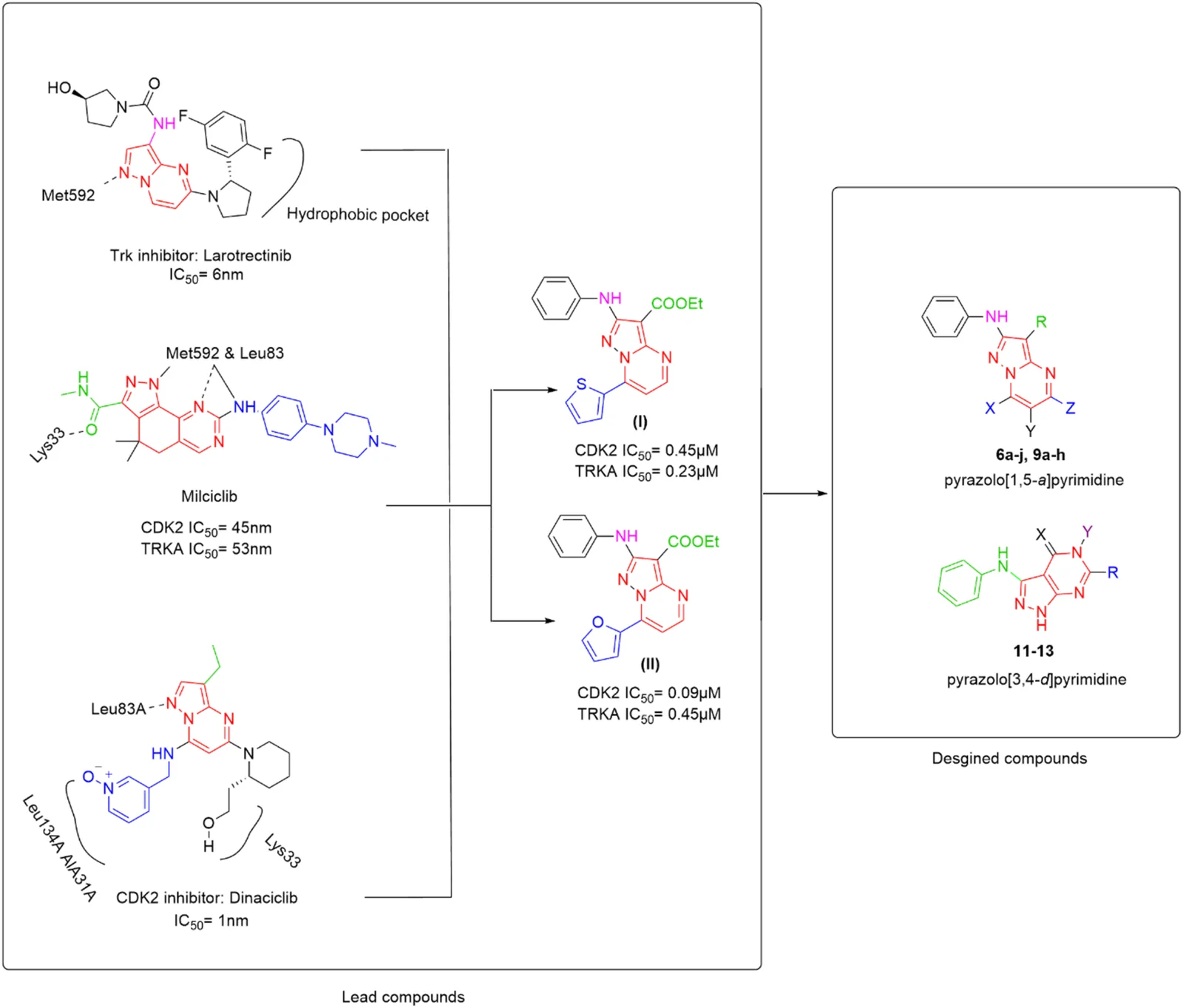
Rational design strategy for the development of novel derivatives based on pyrazolopyrimidines of the [1,5-*a*] and [3,4-*d*] scaffolds through structural optimization.

## 2 Results and discussion

### 2.1 Chemistry

The molecules with the given titles N3,4-diphenyl-1*H*-pyrazole-3,5-diamine (3a), ethyl 5-amino-3-(phenylamino)-1*H*-pyrazole-4-carboxylate (3b) and 5-amino-3-(phenylamino)-1*H*-pyrazole-4-carbonitrile (3c) have been synthesized through reaction of benzoyl acetonitrile (1a) or ethyl cyanoacetate (1b) or malononitrile (1c) with phenylisothiocyanate under the influence of potassium hydroxide to yield the respective potassium thiolate salts, which were then treated with methyl iodide to give compounds 2a–c followed by hydrazine hydrate to afford the pyrazolo derivatives 3a–c (Scheme 1).^25^

**Scheme 1:**
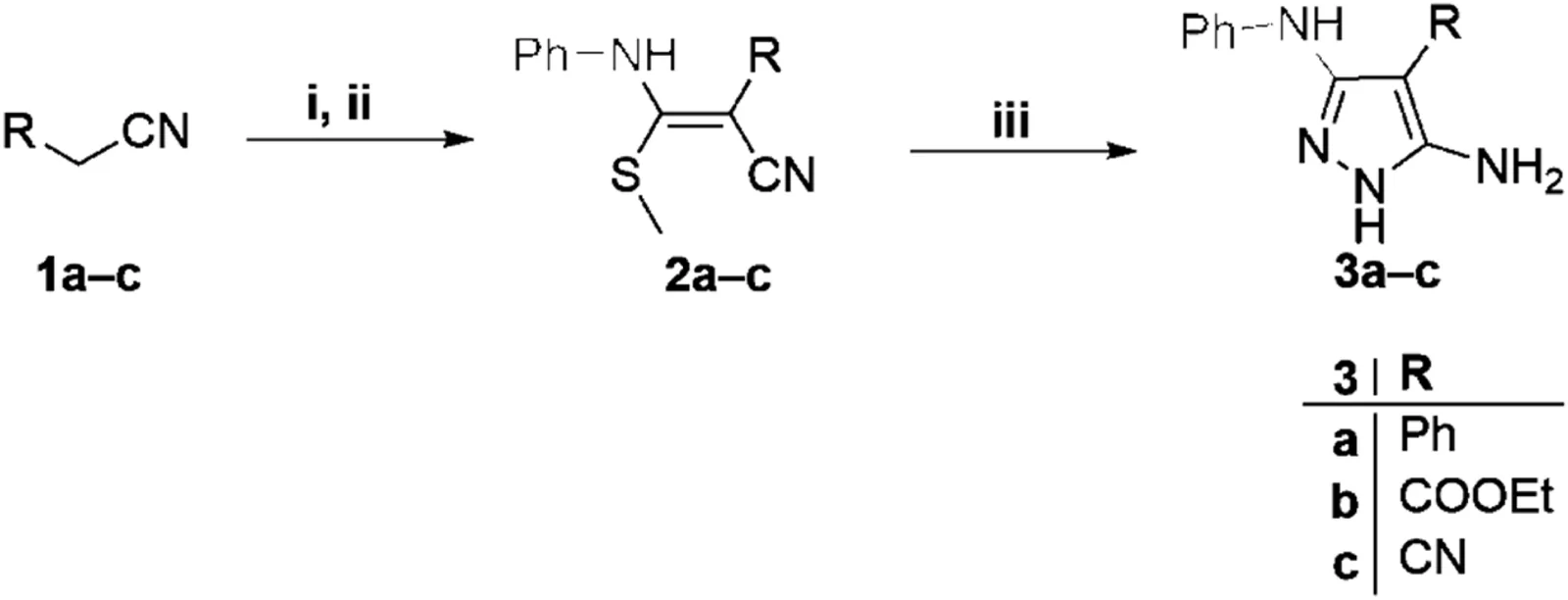
Reagents and conditions: (i) phenyl isothiocyanate/anhydrous KOH/DMF/rt 12 h; (ii) methyliodide/rt 8 h; (iii) hydrazine hydrate 80%/abs. MeOH/reflux 3–4 h.

In this research, enaminones (5a–j) were chosen as adaptable reagents to synthesize pyrazolo[1,5-*a*]pyrimidines (6a–j). A common method for obtaining enaminones (5a–j) involves the condensation of methyl aryl ketones (4a–j) with dimethylformamide dimethylacetal.^26–28^ Following this, the reaction of N3,4-diphenyl-1*H*-pyrazole-3,5-diamine (3a) with enaminones (5a–j) afforded the target pyrazolo[1,5-*a*]pyrimidines (6a–j). It is suggested that this reaction proceeds *via* a nucleophilic attack by the amino group of pyrazole (3a) on the β-carbon of enaminones (5a–j), followed by cyclization *via* subsequent attack of pyrazole NH on carbonyl carbon with the loss of one mole of H_2_O, yielding the ultimate separable products (6a–j) (Scheme 2).

**Scheme 2:**
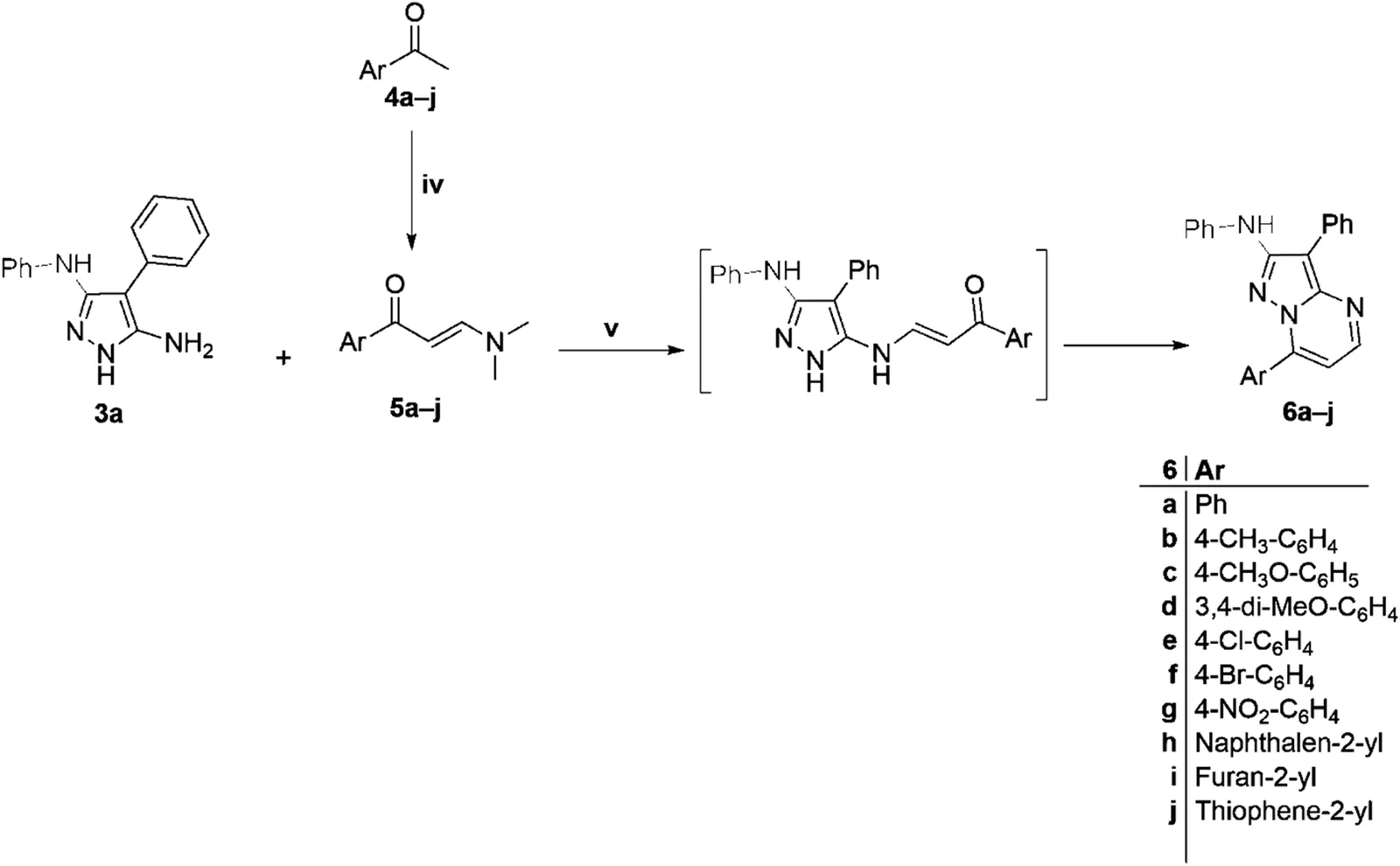
Reagents and conditions: (iv) DMF-DMA/dry xylene/reflux 7 h; (v) glacial AcOH/reflux 3 h.

Furthermore, the reaction of pyrazole (3a–c) derivatives with 2-(arylidene)malononitrile (8a–e) derivatives, synthesized *via* Knoevenagel condensation of malononitrile with aromatic aldehydes (7a–e)^29–31^ in refluxing EtOH under the influence of catalytic quantity of piperidine yielding the respective pyrazolo[1,5-*a*]pyrimidine derivative (9a–d) and dihydropyrazolo[1,5-*a*]pyrimidine derivative (9e–h). The mild ethanolic/piperidine medium promotes cyclization but is not strongly oxidizing, allowing some products to remain in the dihydro state.^32^ The obtained dihydropyrazolo[1,5-*a*]pyrimidine derivative (9e–h) was verified by ^1^H and ^13^C NMR spectral data. The ^1^H NMR spectra showed a singlet signal at *δ* 5.25–5.73 ppm corresponding to the H5 of pyrazolopyrimidine and two exchangeable signals at *δ* 7.61–8.30 ppm for the two NH protons. In the ^13^C NMR spectra of 9e–h, the dihydropyrazolo[1,5-*a*]pyrimidine aliphatic carbon (C-5) signal was detected at *δ* 51.30–74.30 ppm. Moreover, pyrazolo[1,5-*a*]pyrimidine derivative (9a–d) the ^1^H NMR spectra showed only one exchangeable signals at *δ* 8.86–9.51 ppm for the NH proton and absent of H5 of pyrazolopyrimidine signal. In the ^13^C NMR spectra of 9a–d the pyrazolo[1,5-*a*]pyrimidine carbon (C-5) signal was detected at *δ* 156.17–161.76 ppm (Scheme 3).^33–35^

**Scheme 3:**
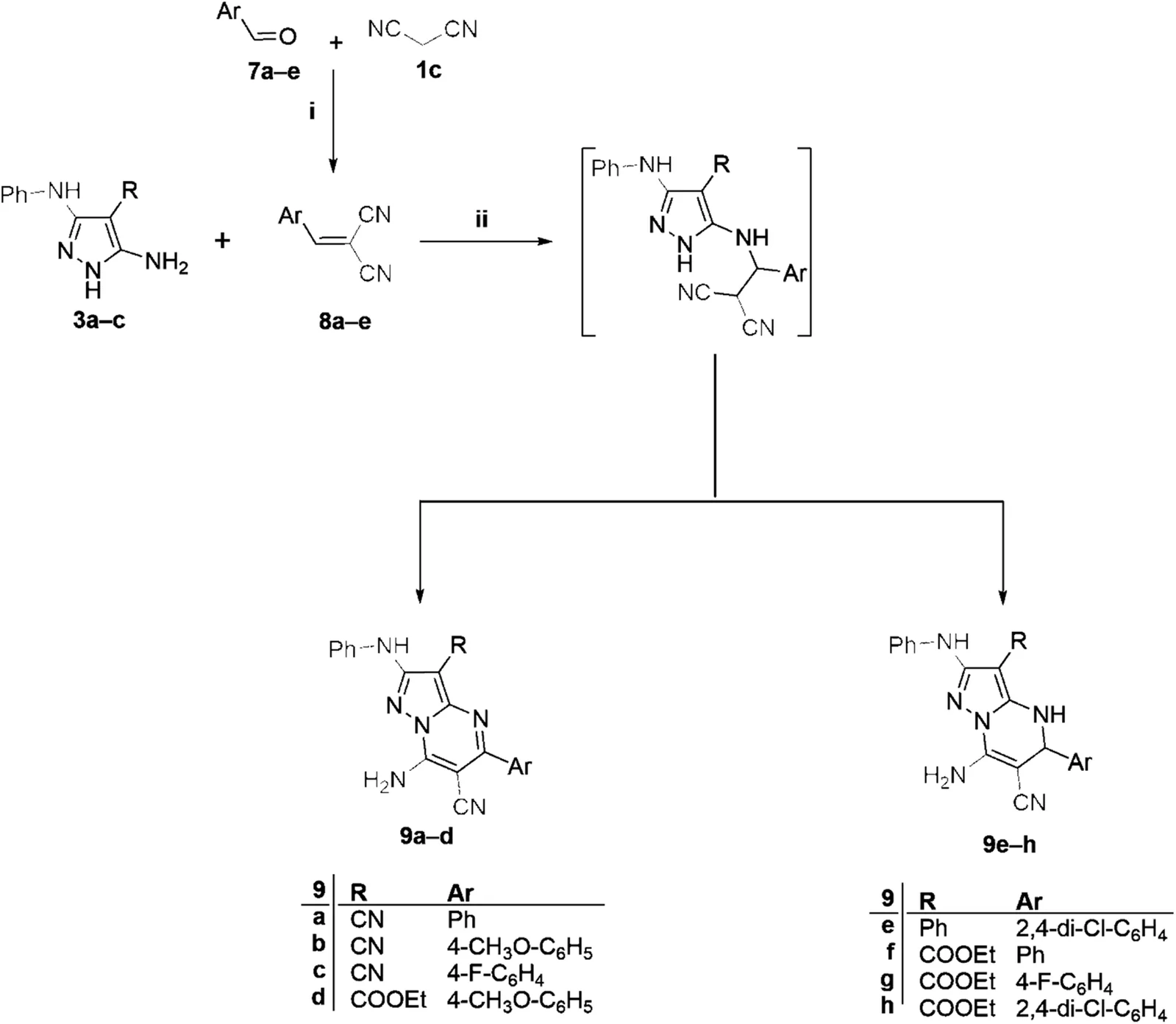
Reagents and conditions: (i) abs. EtOH/piperidine/rt 5 h; (ii) abs. EtOH/piperidine/reflux 8–12 h.

Moreover, the combination of 3c with DMF-DMA yielded the formimidamide derivative (10) which was further cyclized using hydrazine hydrate and give pyrazolo[3,4-*d*]pyrimidine derivative (11) (Scheme 4). Furthermore, reaction of 3c with phenyl isothiocyanate gave pyrazolo[3,4-*d*]pyrimidine-6-thiol derivative (12). On the other hand, reaction of the carboxylate isostere (3b) with phenyl isothiocyanate resulted the cyclized pyrazolo[3,4-*d*]pyrimidine derivative (13) (Scheme 4).

**Scheme 4:**
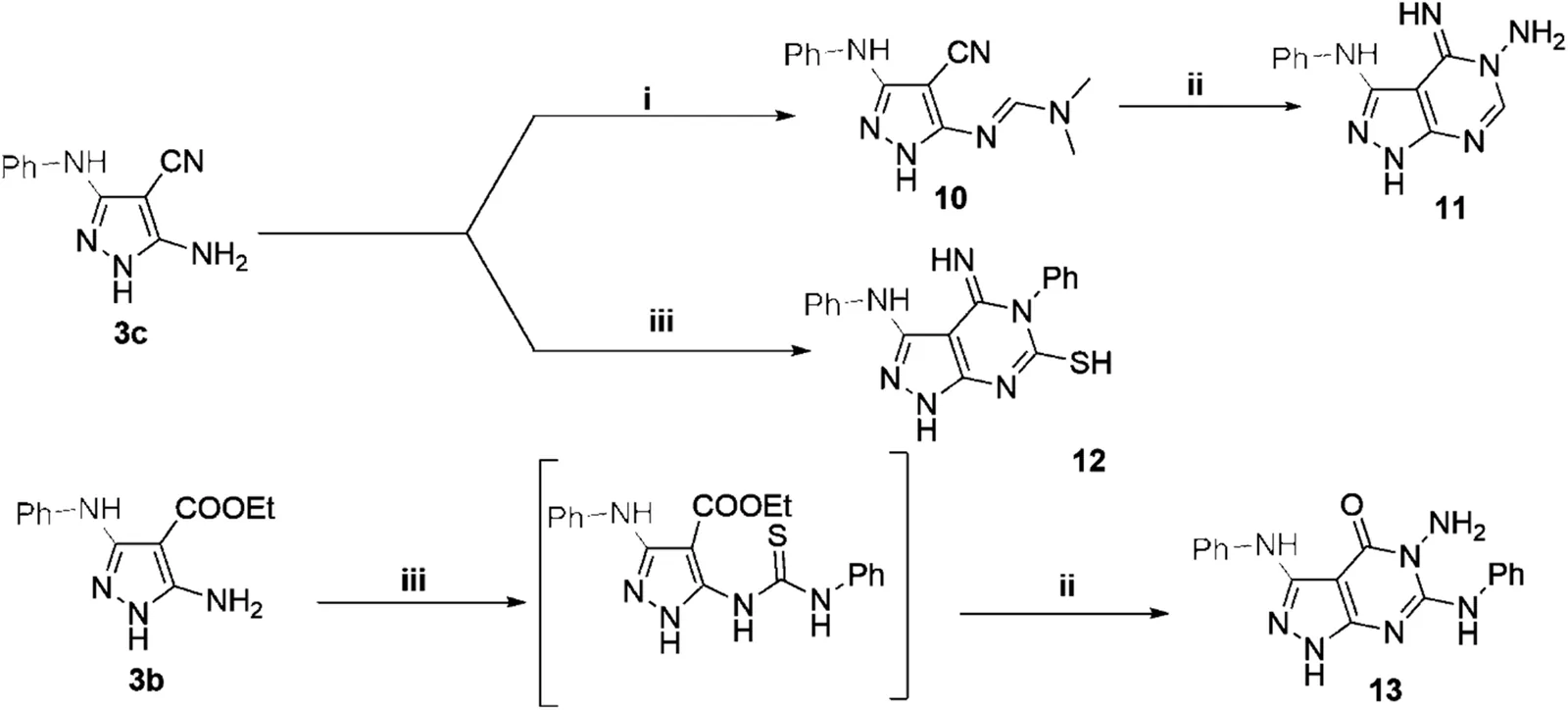
Reagents and conditions: (i) DMF.DMA/reflux 1 h; (ii) hydrazine hydrate 80%/reflux 1 h; (iii) phenyl isothiocyanate/pyridine/reflux 1 h.

### 2.2 Assessment of *in vitro* anticancer potential

#### 2.2.1 Inhibitory effects on cell proliferation across 60 cancer cell lines of NCI

The prepared pyrazolopyrimidines 6a–j, 9a–h and 11–13 were screened for their anti-proliferative activity toward 60 diverse tumor cell lines by the National Cancer Institute (NCI) Developmental Therapeutic Program (https://www.dtp.nci.nih.gov). These cell lines, which represent nine malignant neoplasms: hematologic (leukemia) and solid tumors (melanoma, lung, colon, CNS, ovarian, renal, prostate, and breast carcinomas)—have been evaluated to assess the compounds' effects. A concentration of 10 µM was used for all compounds in the screening. The results, which are shown as growth inhibition (GI %) percentages, represent the effectiveness of the compounds toward the entire set of tumor cell lines.

As a result, compounds 6a–c, 6i, 6j and 9 d–h showed the best inhibition toward certain cell lines (Table 1).

**Table 1:** Compounds 6a–c, 6i, 6j and 9d–h that showed GI% above 50% against 23 NCI cell lines

		6a	6b	6c	6i	6j	9d	9e	9f	9g	9h
Non-small cell lung carcinoma	HOP-92	—	—	—	—	—	53.04	—	—	—	—
	NCI-H460	—	—	—	—	—	—	50.22	—	—	—
	NCI-H226	—	—	—	—	—	57.66	—	—	—	—
Leukemia cell lines	RPMI-8226	—	—	57.19	—	—	—	69.59	—	—	—
	K-562	—	50.61	62.25	—	—	—	69.53	51.51	—	—
	SR	—	—	—	—	—	—	65.34	—	—	—
	MOLT-4	—	—	—	—	—	—	73.68	53.16	54	51.16
	HL-60(TB)	—	—	—	—	—	—	58.51	—	—	—
Colon cancer cell line	HCT-15	—	—	—	—	—	—	60	—	—	—
	HCT-116	—	—	—	—	—	—	52.79	—	—	—
	HT29	46.07	—	—	—	—	—	—	—	—	—
Melanoma cancer cell lines	MDA-MB-435	—	—	—	—	—	—	57.67	—	—	—
Renal cancer cell line	786-0	—	—	—	—	—	67.19	—	—	—	—
	ACHN	—	—	—	—	—	61.47	—	—	—	—
	RFX 393	—	—	—	—	—	54	—	—	—	—
	CAKI-1	—	—	—	—	—	—	51.62	—	—	—
	UO-31	—	—	—	—	—	—	63.82	—	—	—
Prostate cancer cell line	PC-3	—	—	—	—	—	—	56.30	—	—	—
Ovarian cancer cell line	OVCAR-4	—	—	—	—	—	64.13	—	—	—	—
Breast cancer cell lines	MDA-MB-231/ATCC	—	—	—	68.74	—	—	—	—	—	—
	HS 578T	—	—	—	—	—	80.61	—	—	—	—
	MCF7	—	—	—	—	—	—	67.68	—	—	65.18
	T-47D	—	—	—	—	59.80	—	67.68	—	—	—
CNS cancer cell line	SNB-75	—	—	—	—	—	115.32	—	—	—	—
	SF-295	—	—	—	—	—	57.66	—	—	—	—

Compound 9e showed broad-spectrum anticancer activity against a variety of cell panel representing leukemia cell line with GI % ranging from 58.51 to 73.68, lung carcinoma with GI% = 50.22, colon carcinoma cell lines exhibiting GI % falling within the range of 52.79 to 60, prostate carcinoma cell lines with GI% = 56.30, renal carcinoma cell lines exhibiting GI % falling within the range of 51.62 to 63.82, breast carcinoma cell lines with GI % = 67.68 and melanoma cell lines with GI% = 57.67.

Additionally, compounds 9d and 9e displayed significant inhibition toward CNS carcinoma cell line SNB-75 with GI % = 115.32 and 42.17, respectively.

These studies led us to conclude that pyrazolo[1,5-*a*]pyrimidine exhibited more promising results compared to pyrazolo[3,4-*d*]pyrimidine.

#### 2.2.2 Kinase inhibition

The structure–activity relationship of the synthesized pyrazolopyrimidines was established by correlating the structural modifications with their inhibitory activities against CDK2 and TRKA (Table 2). Overall, the pyrazolo[1,5-*a*]pyrimidine scaffold displayed superior dual kinase inhibition compared with the pyrazolo[3,4-*d*]pyrimidine analogues, indicating that the [1,5-*a*]-fused framework provides a more favorable geometry for occupying the ATP-binding pockets of both kinases.

**Table 2:** Kinase inhibition potential of the newly synthesized compounds 6a–j and 9a–i (IC_50_ µM). General structure of the newly synthesized pyrazolopyrimidine compounds 6a–j and 9a–h

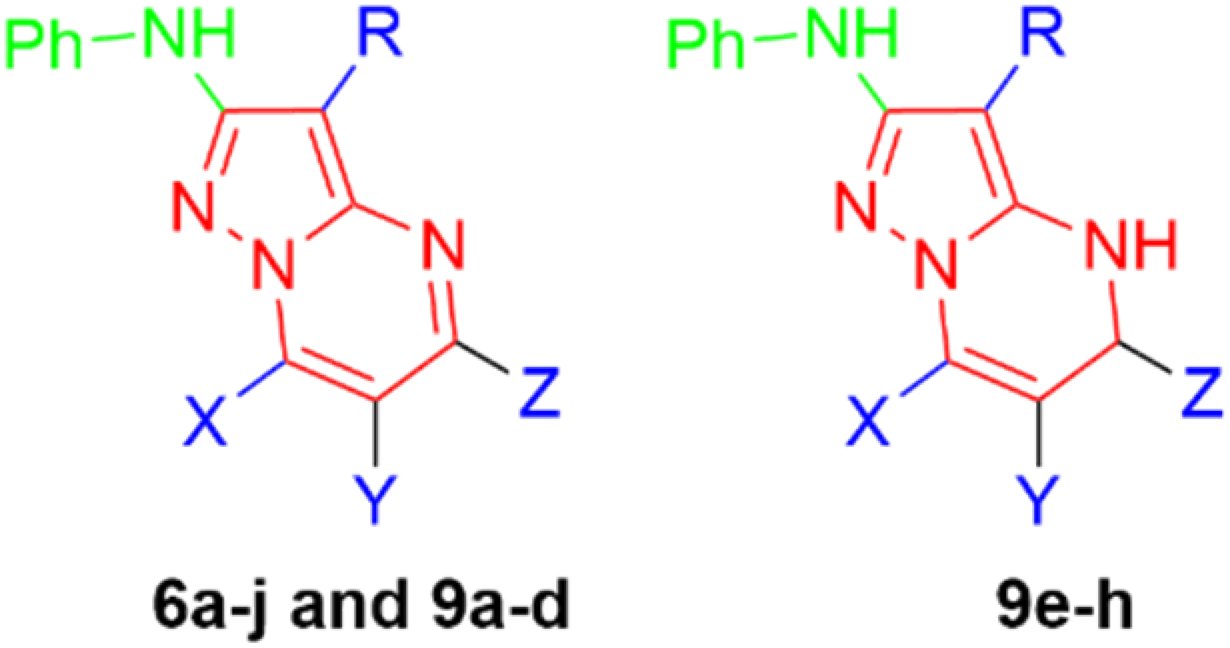						
Code	R	X	Y	Z	Enzyme inhibition IC_50_ (µM)	
6a	Ph	Ph	H	H	CDK-2	TRK-A
					0.77 ± 0.05	2.35 ± 0.07
6b	Ph	4-CH_3_-C_6_H_5_	H	H	0.78 ± 0.04	3.42 ± 0.09
6c	Ph	4-CH_3_O-C_6_H_5_	H	H	0.79 ± 0.05	0.89 ± 0.03
6i	Ph	Furan-2-yl	H	H	0.34 ± 0.01	0.16 ± 0.01
6j	Ph	Thiophene-2-yl	H	H	0.33 ± 0.01	0.08 ± 0.004
9d	COOEt	NH_2_	CN	4-CH_3_O-C_6_H_5_	0.35 ± 0.01	0.23 ± 0.01
9e	Ph	NH_2_	CN	2,4-Di-Cl-C_6_H_5_	1.07 ± 0.03	0.32 ± 0.01
9f	COOEt	NH_2_	CN	Ph	0.89 ± 0.03	3.29 ± 0.08
9g	COOEt	NH_2_	CN	4-F-C_6_H_5_	0.21 ± 0.01	0.14 ± 0.004
9h	COOEt	NH_2_	CN	2,4-Di-Cl-C_6_H_5_	0.56 ± 0.02	0.34 ± 0.03
Ribociclib					0.07 ± 0.01	—
Larotrectinib					—	0.07 ± 0.01

Within the first series (6a–j), modification of the C-7 aromatic substituent (X) had the greatest impact on biological activity. The parent phenyl derivative 6a exhibited only moderate inhibition against CDK2 (IC_50_ = 0.77 µM) and TRKA (IC_50_ = 2.35 µM). Introducing para-electron-donating substituents such as methyl (6b) or methoxy (6c) produced only limited improvement. Although the methoxy derivative showed enhanced TRKA inhibition compared with the methyl analogue, neither approached the potency of the heteroaryl-containing compounds.

Replacement of the phenyl ring with five-membered heteroaromatic rings dramatically enhanced dual kinase inhibition. The furan analogue 6i and the thiophene analogue 6j were the most potent members of this series, exhibiting CDK2 IC_50_ values of 0.34 and 0.33 µM and TRKA IC_50_ values of 0.16 and 0.08 µM, respectively. This improvement can be attributed to the smaller size and increased electronic richness of the heteroaryl rings, which facilitate a better fit within the ATP-binding pocket while providing favorable hydrophobic and hydrogen-bonding interactions. These findings are supported by the molecular docking study, where compound 6i established key interactions with Lys33 in CDK2 and Met592 in TRKA, together with additional hydrophobic contacts that stabilized the ligand within both active sites.

The second pyrazolo[1,5-*a*]pyrimidine series (9a–h) further demonstrated that both scaffold aromatization and terminal aryl substitution significantly influenced activity. The aromatic analogue 9d showed excellent dual inhibition (CDK2 IC_50_ = 0.35 µM; TRKA IC_50_ = 0.23 µM), suggesting that increased planarity of the aromatic pyrazolopyrimidine core favors binding to both enzymes. In contrast, the corresponding dihydro analogue 9f displayed substantially weaker activity, highlighting the importance of aromatic stabilization for productive kinase binding.

The effect of electronic substitution on the terminal phenyl ring was also evident within the dihydro series. Incorporation of electron-withdrawing halogens markedly enhanced inhibitory potency. The *para*-fluoro derivative 9g exhibited the highest activity within this series (CDK2 IC_50_ = 0.21 µM; TRKA IC_50_ = 0.14 µM), while the 2,4-dichloro derivative 9h also demonstrated potent dual inhibition. These results suggest that halogen substitution enhances hydrophobic interactions within the kinase binding pocket and may optimize ligand orientation through favorable steric and electronic effects (Fig. 3).

**Fig. 3:**
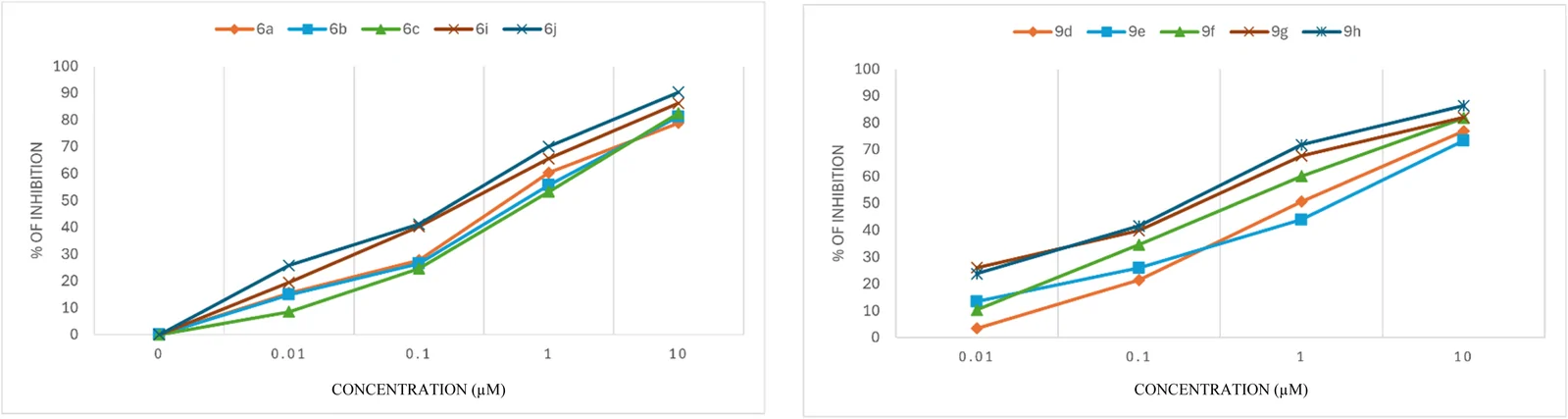
A plot of the inhibitory potency (IC_50_) of the compounds 6a–c, 6i, 6j and 9d–h for inhibition of CDK2 activity. Compounds were evaluated at different concentrations spanning 0.01 to 10 µM. The results as % inhibition are displayed as mean values ± standard deviation across three independently performed assays.

Collectively, the SAR indicates that dual CDK2/TRKA inhibition is favored by (i) maintaining the pyrazolo[1,5-*a*]pyrimidine scaffold, (ii) introducing small heteroaryl substituents at the C-7 position, (iii) preserving the aromatic fused core, and (iv) incorporating appropriately positioned electron-withdrawing substituents on the terminal aryl ring. These structural features produced the most balanced kinase inhibition and translated into superior antiproliferative activity, identifying compounds 6i and 6j as the most promising lead structures for further optimization (Fig. 4).

**Fig. 4:**
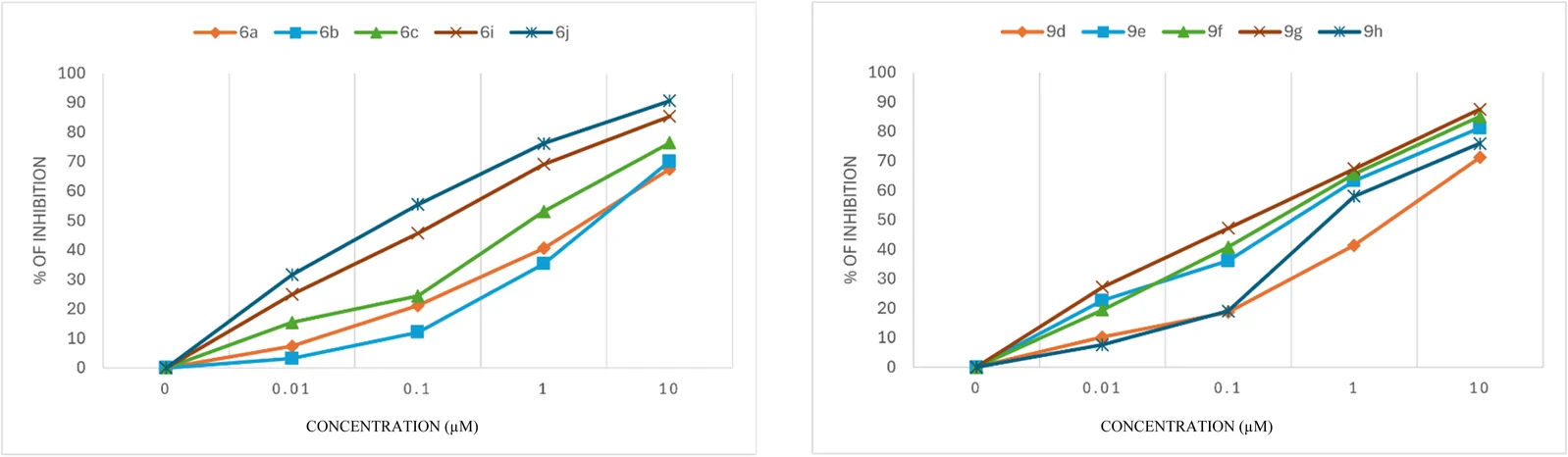
A plot of the inhibitory potency (IC_50_) of the compounds 6a–c, 6i, 6j and 9d–h for inhibition of TRKA activity. Compounds were evaluated at different concentrations spanning 0.01 to 10 µM. The results as % inhibition are displayed as mean values ± standard deviation across three independently performed assays.

#### 2.2.3 *In vitro* cytotoxicity against the CNS cancer cell line SNB-75

In CNS cancers, both CDK2 and TRKA are often overexpressed, contributing to tumor progression and poor prognosis.^36,37^

Compounds 6i, 6j and 9d were chosen for testing as they presented enhanced enzyme inhibition, exhibiting the strongest activity among the synthesized pyrazolopyrimidine analogs. The anticancer potential of pyrazolopyrimidine compounds was tested *in vitro* using the SNB-75 CNS carcinoma cell line due to its overexpression of CDK2 and TRKA. The standard inhibitor used for comparison was staurosporine. According to the results in Table 3, compound 6i caused stronger inhibition compared to the benchmark with inhibitory potency (IC_50_) of 7.39 µM.

**Table 3:** Assessment of *in vitro* cytotoxicity for 6i, 6j and 9d against the CNS cancer cell line SNB-75 (IC_50_ µM)

Code	SNB-75 (IC_50_, µM)
6i	7.39 ± 0.25
6j	23.40 ± 0.79
9d	22.10 ± 0.75
Staurosporine	9.99 ± 0.34

#### 2.2.4 *In vitro* cytotoxicity against the CML cancer cell line K562 and PCS-800-017 normal human fibroblast cell line

Targeting CDK2 is a validated strategy in CML, as its activity is essential for BCR-ABL-driven cell cycle progression.^38^ Although NTRK1 gene fusions are rare in hematological malignancies,^39^ the simultaneous inhibition of CDK2 and TRKA represents a multi-targeted approach designed to disrupt complementary survival pathways, a strategy that may be effective in overcoming drug resistance.^40^

Compounds 6i, 6j and 9d were advanced for antiproliferative evaluation based on their superior enzyme inhibition profiles. Their efficacy and selectivity were assessed against the K562 human chronic myeloid leukemia cell line and the PCS-800-017 normal human fibroblast cell line, with the natural flavonoid quercetin serving as the benchmark. According to the results in Table 4, compound 6i possesses remarkable cytotoxicity against K562 cells (IC_50_ = 14.08 µM), which is comparable to the potency of quercetin (IC_50_ = 15.82 µM). Furthermore, compound 6i demonstrated significantly stronger anticancer activity than its analogs 6j and 9d, which showed markedly reduced potency with an IC_50_ of 42.28 µM and 17.47 µM, respectively. Crucially, 6i also exhibited the most favorable selectivity profile, being less toxic to normal fibroblasts (IC_50_ = 65.25 µM) than quercetin (IC_50_ = 37.53 µM), underscoring its potential as a promising and selective anticancer agent.

**Table 4:** Assessment of *in vitro* cytotoxicity for 6i, 6j and 9d against the CML cancer cell line K562 and PCS-800-017 normal human fibroblast cell line (IC_50_ µM)

Code	K562 (IC_50_, µM)	Pcs-800-17
6i	14.08 ± 0.41	65.25 ± 2.19
6j	42.28 ± 1.24	51.51 ± 1.73
9d	17.47 ± 0.59	50.99 ± 1.73
Quercetin	15.82 ± 0.39	37.53 ± 1.26

#### 2.2.5 Assessment of cell cycle progression and apoptosis

##### 2.2.5.1 Assessment of cell cycle progression

CDK2 enzyme has been well studied to regulate all stages of the cell cycle.^41^ It is a master regulator of the cell cycle that is responsible for late G1 phase as well as throughout the S phase.^42^ Likewise, it has been reported that TRKA signaling (*via* NGF) can promote proliferation and cell cycle progression, facilitating the G2/M transition.^43^ In this investigation, compounds 6i and 6j, which were found to have significant inhibition activity against CDK2 and TRKA and good cytotoxicity against SNB-75 cancer cells, were investigated for their influence on the cell cycle process and triggering of programmed cell death. SNB-75 cells received treatment with GI_50_ concentrations of pyrazolopyrimidine derivatives 6i and 6j for 48 hours. The outcomes in Table 5 also showed that the two compounds induced marked blockade of G2/M stage of the cell cycle, where the treated cell population raised to 44.29% and 33.32%, respectively, in comparison with 8.45% of the control (Fig. 5). Whereas the G0–G1 phase population declined to 41.39% and 50.88%, compared to 59.03% in the control. Additionally, S phase cell fraction reduced to 14.33% and 15.81%, respectively, against 32.53% in the control. These results demonstrate that cytotoxicity is inhibited by the stimulation of both cell cycle arrest and apoptosis by compounds 6i and 6j.

**Table 5:** Assessment of cell cycle progression for compounds 6i and 6j on SNB-75 cell line. The results as % inhibition are displayed as mean values ± standard deviation across two independently performed assays

Compound	%G0–G1	%S	%G2/M
6i/SNB-75	41.39 ± 5.51	14.33 ± 1.35	44.29 ± 4.16
6j/SNB-75	50.88 ± 3.30	15.81 ± 1.83	33.32 ± 5.13
SNB-75 control	59.03 ± 2.85	32.53 ± 3.98	8.45 ± 1.13

**Fig. 5:**
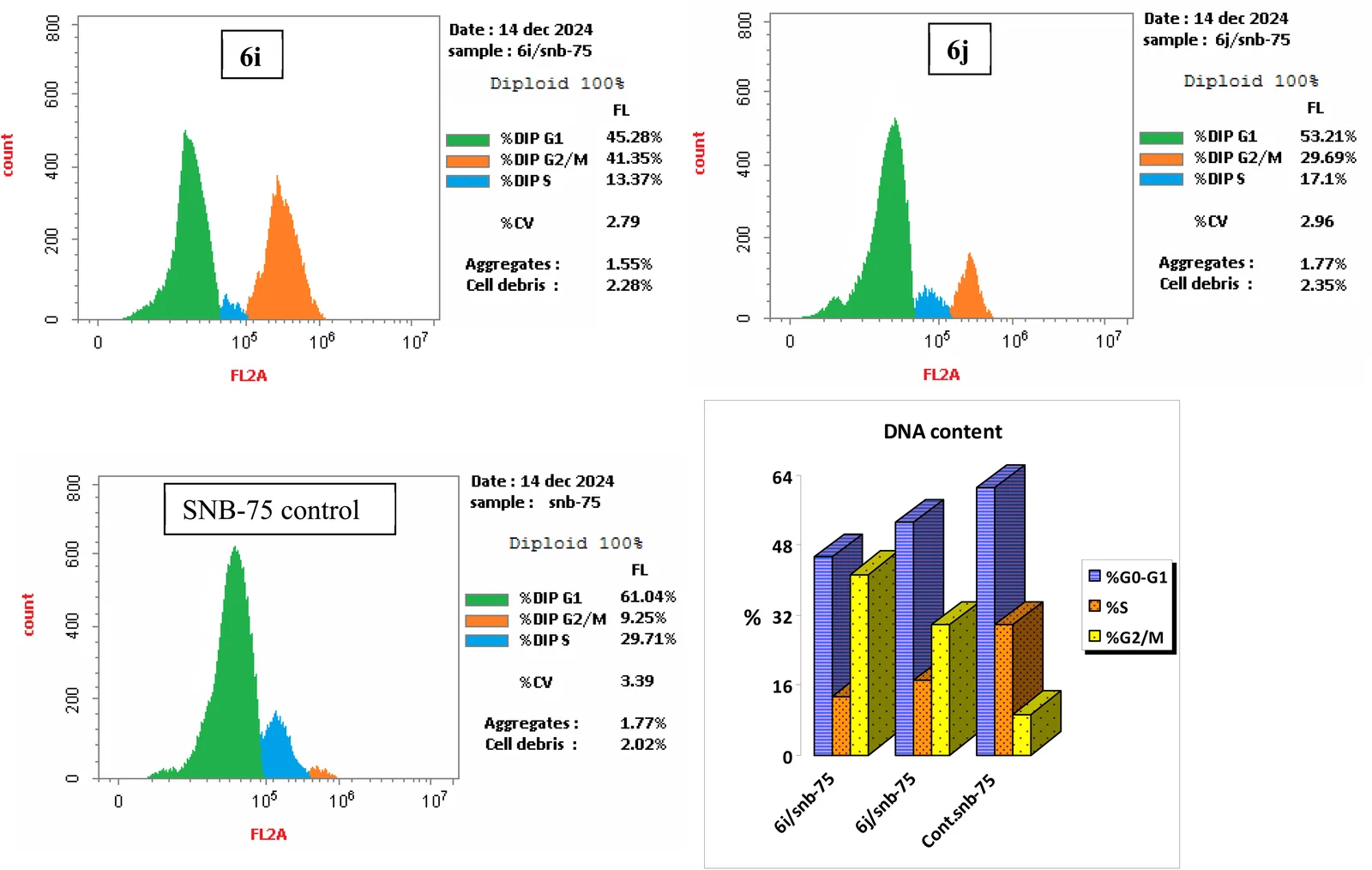
Assessment of cell cycle progression for compounds 6i and 6j on SNB-75 cell line.

##### 2.2.5.2 Assessment of apoptosis and necrosis

Apoptosis plays a crucial protective mechanism against cancer development.^44^ The ability of malignant cells to evade apoptosis is one of the most important characteristics of cancer.^45^ Novel anticancer drugs have been hopeful in triggering the apoptotic cascade, later on by triggering pro-apoptotic or inhibiting anti-apoptotic proteins.^46^ Necrosis is defined by cell damage that is irreversible and culminates in cell death due to pathological processes. In contrast to the regulated process of apoptotic cell death, necrosis is an uncontrolled form of death that causes cells to spill their contents, often resulting in harmful inflammation.^47^

In an effort to determine the effect of 6i and 6j on apoptotic processes, SNB-75 cells received a 48-hours treatment with the compounds after which they were subjected to Annexin V/PI staining. The findings in comparison with the control, it was evident that these compounds triggered a marked blockade at the G2/M checkpoint. Compound 6i caused the highest total apoptosis (33.72%), dominated by late apoptosis (21.98%) also showed the highest necrosis level (6.85%), which might indicate more aggressive or less selective cytotoxicity, compound 6j also increased apoptosis to 12.35%, mainly late-stage, with moderate necrosis (3.49%). The cell death pathway is predominantly apoptotic, with limited necrotic contribution. Detailed findings are presented in Table 6 and represented in (Fig. 6).

**Table 6:** Assessment of apoptosis and necrosis for compounds 6i and 6j on SNB-75 cell line. The results as % inhibition are displayed as mean values ± standard deviation across two independently performed assays

Compound	Apoptosis			Necrosis
	%Total	%Early	%Late	
6i/SNB-75	33.72 ± 1.85	4.90 ± 1.35	21.98 ± 0.13	6.85 ± 0.36
6j/SNB-75	20.36 ± 0.98	4.53 ± 0.94	12.35 ± 0.76	3.49 ± 0.80
SNB-75 control	3.10 ± 0.31	0.55 ± 0.10	0.13 ± 0.08	2.42 ± 0.30

**Fig. 6:**
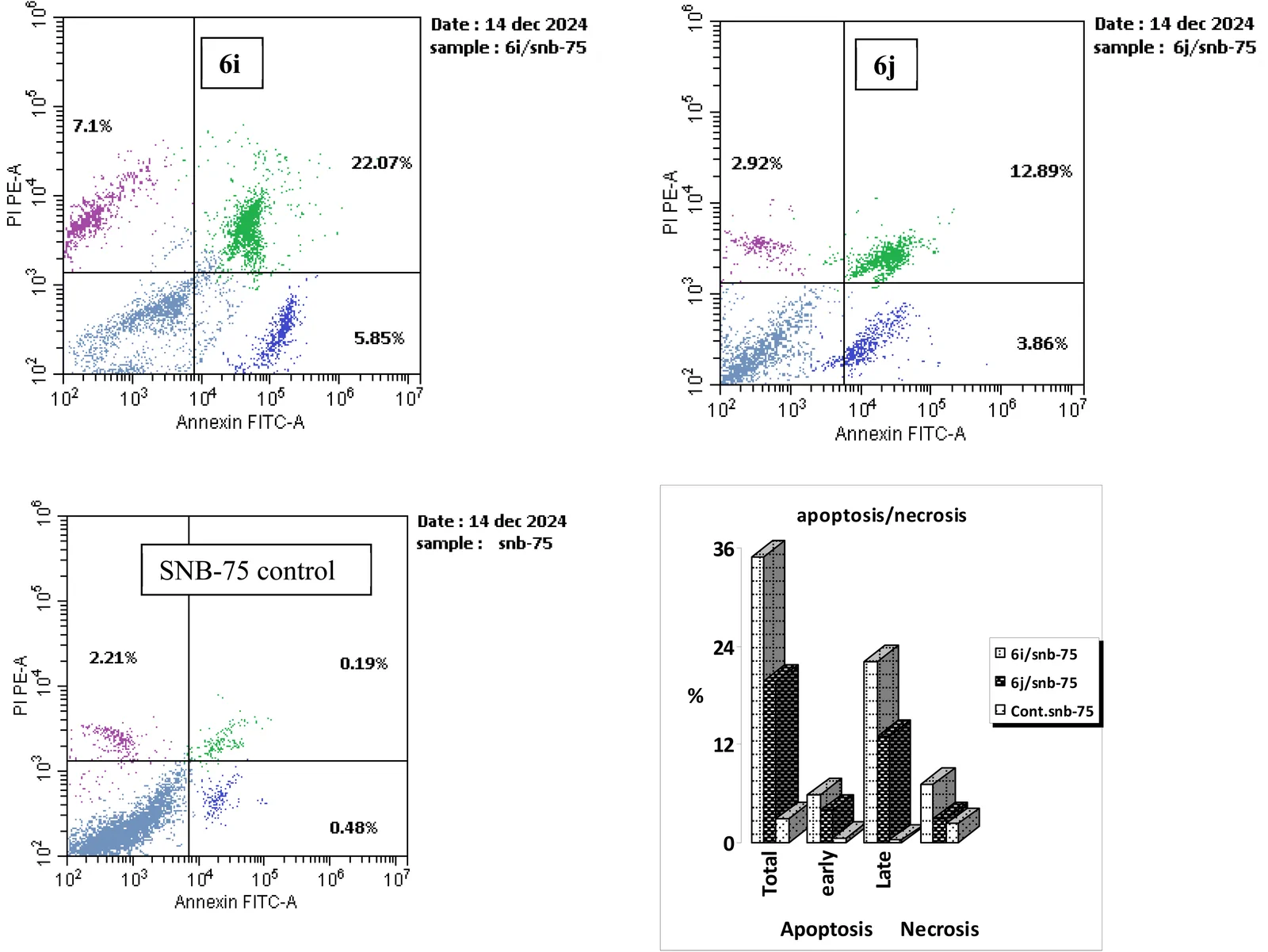
Assessment of apoptotic and necrotic effects of compounds 6i and 6j on SNB-75 cell line.

#### 2.2.6 Molecular docking study

Extensive docking analysis were accomplished to offer supporting evidence for the antitumor activity for various natural and synthetic drugs. Molecular docking simulation through the Molecular Operating Environment (MOE, 2019) suite had been carried out to analyze the binding patterns and spatial orientation of the synthesized CDK2 and TRKA kinase inhibitors and to offer multiple crystalline frameworks of CDK2 and TRKA, they have been recognized and documented as co-crystallized with various inhibitors.^48–51^ The key binding interactions of the targeted pyrazolopyrimidines was investigated by utilizing the co-crystallized CDK2 and TRKA with milciclib and repotrectinib (PDB codes 2WIH and 7VKO, respectively). To validate the docking protocol, the co-crystallized ligands (milciclib and repotrectinib) have been redocked at CDK2 and TRKA active sites, respectively. Where they showed value of root mean square deviation (RMSD) < 2 Å. The corresponding criteria of validation yielded RMSD values of 0.920 Å for CDK2 and 0.292 Å for TRKA.

Co-crystallized ligand is involved in H-bond interaction with CDK2 with Leu-83 in the hinge region with pyrimidine ring nitrogen and anilino group NH, along with another H-bonding interaction of Lys-33 with the amide group C=O (Fig. 7A). For TRKA, the co-crystallized ligand interacted with Met-592 *via* H-bonding, and further interactions of H-bonding have been observed for Glu-590 and Arg-654 with the pyrimidine ring and phenyl ring, respectively (Fig. 7B).

**Fig. 7:**
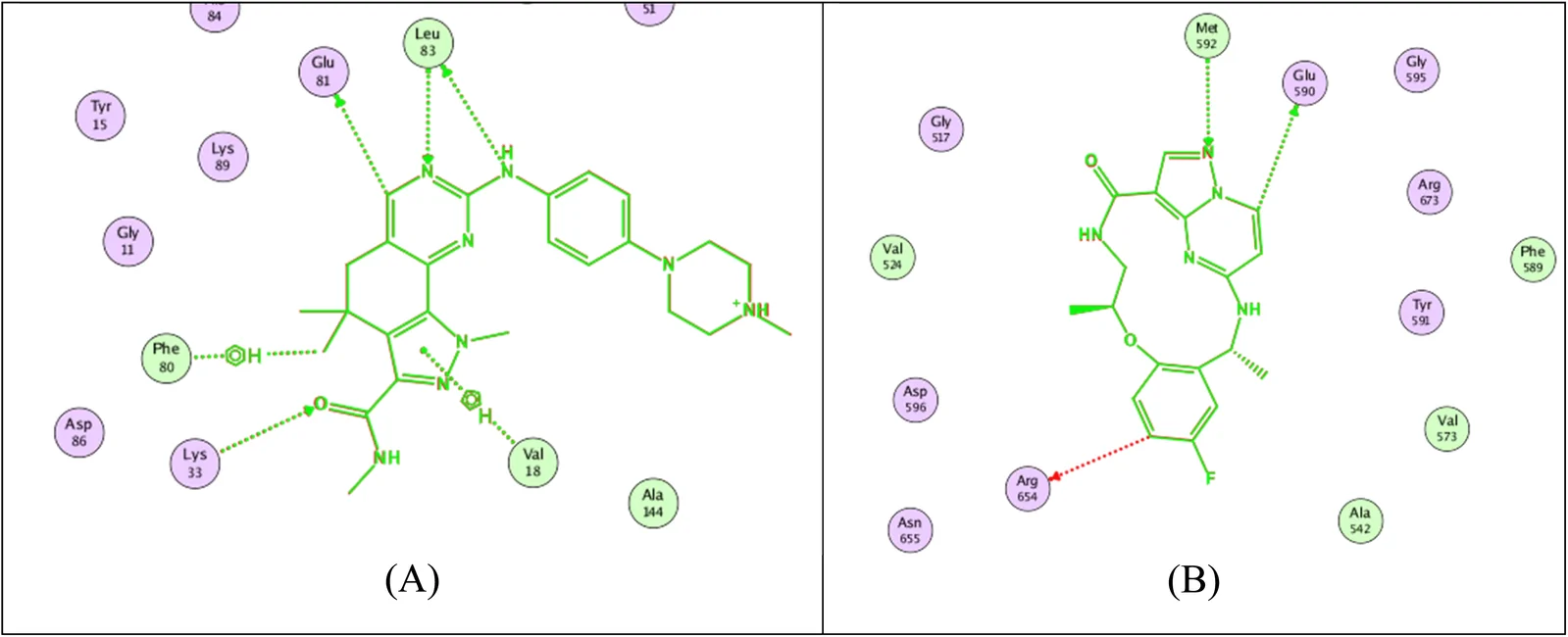
(A) The binding of CDK2 with its co-crystallized ligand (milciclib), and (B) the binding of TRKA with the co-crystallized ligand (repotrectinib).

The most potent newly synthesized pyrazolo[1,5-*a*]pyrimidines 6a–c, 6i, 6j, 9d, 9f and 9h were docked into binding pockets of CDK2 (PDB ID: 2WIH) and TRKA (PDB ID: 7VKO). These molecules demonstrated binding pattern and orientations similar to those of the known CDK2 inhibitor milciclib and TRKA inhibitor repotrectinib. Among them, compound 6i, the most potent CDK2 inhibitor with good TRKA activity, demonstrated strong interactions with critical amino acids formed H-bonding interactions with Lys-33 of CDK2 (similar to milciclib) and Met-592 of TRKA (similar to repotrectinib). Hydrophobic interactions in CDK2 involved the pyrimidine group with Val-18 and the furan group with Phe-80, contributing to the complex's stability. In TRKA, the pyrazolo group interacted with Val-524, while the phenyl group interacted with Leu-516, further stabilizing the complex (Fig. 8).

**Fig. 8:**
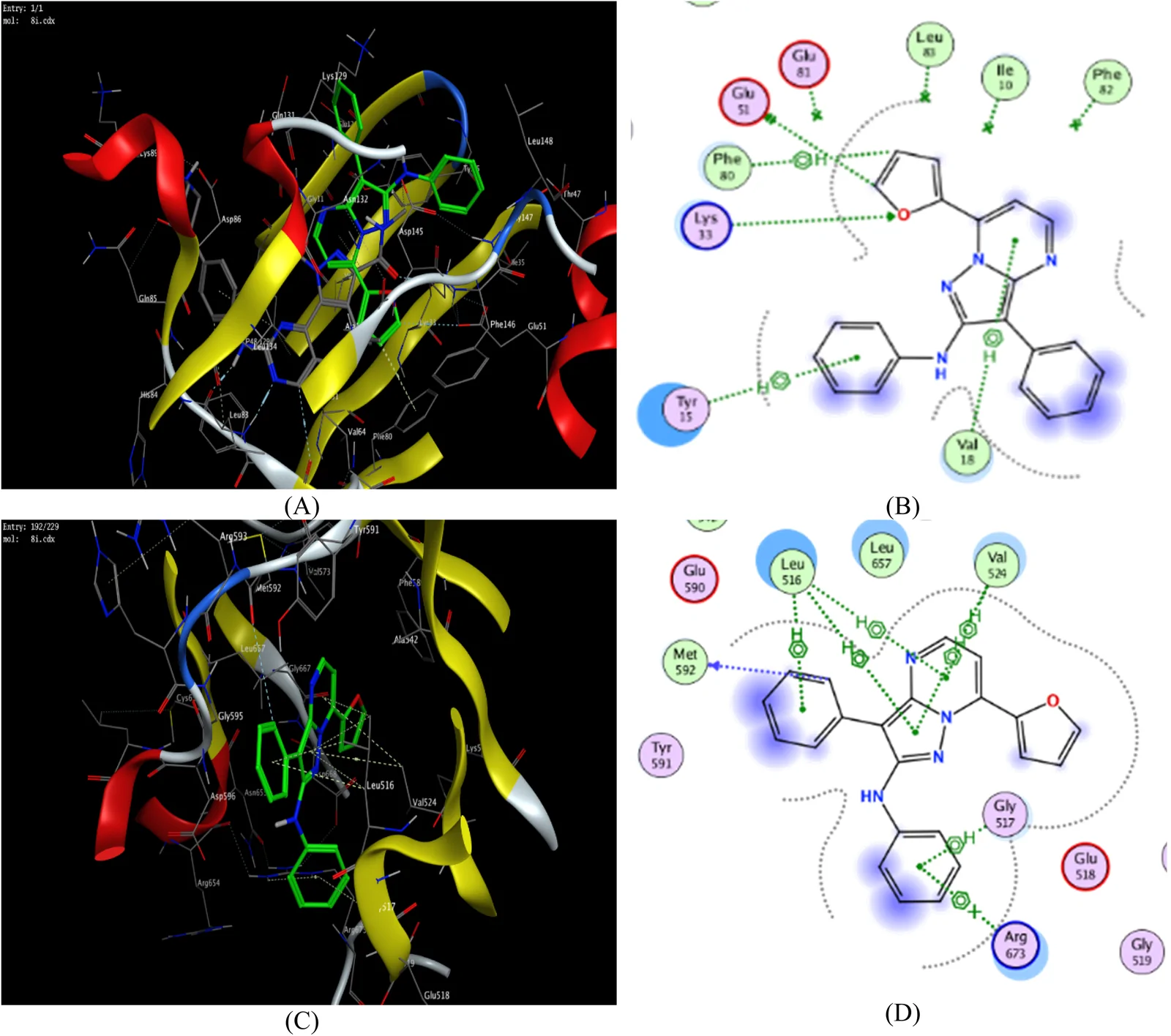
(A and B) Three-dimensional (green) and two-dimensional interactions of compound 6i within the CDK2 active site; (C and D) three-dimensional (green) and two-dimensional interactions within the TRKA active site.

#### 2.2.7 *In silico* ADME study

An ADME analysis utilizing computer-aided tools was carried out with the Swiss ADME computational tools. This analysis concentrates on the molecular composition of the molecule and calculates several parameters, such as intestinal absorption and brain penetration, employing the BOILED-Egg approach to evaluate the compound's interaction with permeability glycoprotein (P-gp) as either a substrate or non-substrate. It also examines the interaction between the molecule and cytochromes P450 (CYP), analyzes the bioavailability index, and examines the molecular characteristics grounded in the five rule of Lipinski. Additionally, the analysis includes calculations for molar refractivity, the partition coefficient between *n*-octanol and water (log P_o/w) and total polar surface area (TPSA).

##### 2.2.7.1 ADME study findings

The ADME analysis provided valuable insights into the pharmacokinetic properties of the most active newly synthesized pyrazolopyrimidines 6a–c, 6i, 6j and 9d–h. Most compounds exhibited moderate aqueous solubility, a key factor for bioavailability. CYP2D6 interaction analysis identified the majority of compounds as potential inhibitors, highlighting possible drug–drug interactions. The BOILED-EGG plot indicated that three molecules could cross the Blood–Brain Barrier (BBB), suggesting potential CNS effects, while seven were unlikely to do so. The Human Intestinal Absorption (HIA) plot confirmed excellent absorption potential for all compounds. These findings, along with structural analysis, will aid in selecting the most promising candidates for further development (Fig. 9).

**Fig. 9:**
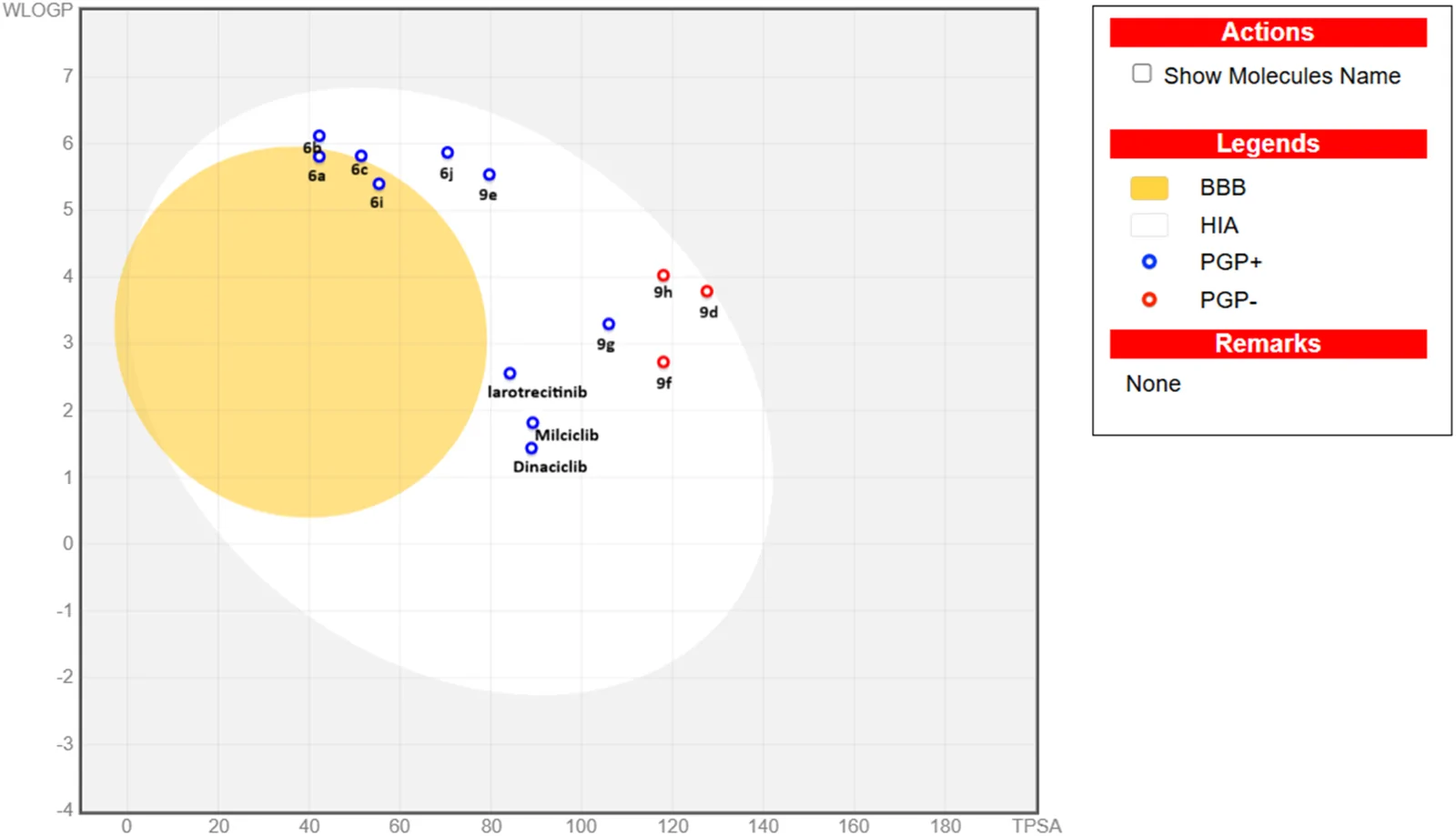
The most active newly synthesized molecules' BOILED-Egg plot generated by swissADME.

##### 2.2.7.2 Prediction of toxicity

To predict the toxicological endpoints and organ toxicities of the ligands, including their LD_50_ values, the Protox 3.0 server^52^ was used for the most active newly synthesized pyrazolopyrimidines 6a–c, 6i, 6j, 9d, 9f and 9h. Chemical structures were retrieved through the merged PubChem by entering the compound names. After selecting the appropriate models, the online server calculated the acute toxicity and identified the toxicity targets for each compound.

The toxicity assessment was based on six parameters associated with adverse drug effects: hepatotoxicity, carcinogenicity, immunotoxicity, mutagenicity, and cytotoxicity. Hepatotoxicity was observed in all tested compounds except for 9d. Compound 9h was identified as non-carcinogenic, while mutagenic potential was detected in compounds 6a–c, 6j and 9d.

For acute toxicity evaluation, compounds from the 6a–d series, with LD_50_ values of 800 mg kg^−1^, were categorized under toxicity class 4. Compounds 6i and 6j, with LD_50_ values of 300 mg kg^−1^ and 500 mg kg^−1^, respectively, were categorized under class 3. Similarly, compound 9d with an LD_50_ value of 300 mg kg^−1^, was also classified as class 3, whereas compounds 9e–h, with LD_50_ values of 1000 mg kg^−1^, were placed in class 4.

## 3 Conclusion

In this study, a series of novel pyrazolo[1,5-*a*]pyrimidine and pyrazolo[3,4-*d*]pyrimidine derivatives were successfully designed, synthesized, and biologically evaluated as potential dual CDK2/TRKA inhibitors. The adopted molecular design, based on systematic structural modifications of the pyrazolopyrimidine scaffold, led to the identification of compounds with promising antiproliferative and dual kinase inhibitory activities.

Among the synthesized compounds, derivatives 6i and 6j exhibited the most favorable biological profiles, demonstrating potent inhibition of both CDK2 and TRKA in the submicromolar range together with remarkable antiproliferative activity against the SNB-75 CNS cancer cell line. In addition, compound 6i displayed selective cytotoxicity toward K562 leukemia cells while maintaining low toxicity toward normal WI-38 fibroblasts. Flow cytometric analyses further demonstrated that the lead compounds induced significant G2/M cell-cycle arrest and apoptosis, supporting their proposed mechanism of anticancer activity.

The molecular docking studies confirmed that compounds 6i and 6j adopted favorable binding orientations within the ATP-binding pockets of both CDK2 and TRKA, in agreement with their experimentally determined kinase inhibitory activities. Furthermore, the *in silico* ADME assessment indicated acceptable drug-like properties for the most active compounds, supporting their suitability as lead candidates for further development.

Overall, the present study establishes the pyrazolopyrimidine scaffold as a promising platform for the development of dual CDK2/TRKA inhibitors and provides valuable structure–activity relationship insights for future optimization. The identified lead compounds, particularly 6i and 6j, warrant further pharmacological investigations, including *in vivo* efficacy, pharmacokinetic evaluation, and safety studies, to explore their potential as targeted anticancer agents.

## 4 Experimental

### 4.1 Chemistry

Melting points (°C, uncorrected) were recorded on a Stuart melting point apparatus. IR spectra were recorded on a SHIMADZU FT/IR spectrometer as KBr pellets. NMR spectra were recorded on a BRUKER 400 MHz NMR spectrometer in DMSO-*d*_6_ as a solvent. All N**H** and N**H**_2_ are D_2_O exchangeable. Chemical shifts are expressed in parts per million (*δ*) and coupling constants (*J*) are reported in Hertz. TMS was utilized as an internal standard, and chemical shifts were quoted in ppm. ^1^H and ^13^C NMR spectra were recorded at 400 MHz and 100 MHz, respectively. Electron impact mass spectrometry was performed using a Direct Probe Controller Inlet, which is interfaced with a single quadrupole mass analyzer (THERMO SCIENTIFIC GCMS, ISQ LT model), data analysis was performed *via* Thermo X-calibur software. HRMS data were acquired using LC/Q-TOF 6530 (Agilent) at the Natural Product Research Lab, Fayoum University.

#### 4.1.1 Preparation of 3-(methylthio)-2-phenyl-3-(phenylamino)acrylonitrile (2a), ethyl 2-cyano-3-(methylthio)-3-(phenylamino)acrylate (2b) and 2-((methylthio)(phenylamino)methylene)malononitrile (2c)

A stirred mixture of phenyl isothiocyanate (0.14 g, 2 mmol) within DMF (5 mL), has been introduced to malononitrile (1.32 g, 2 mmol) for 2a, ethyl cyanoacetate (0.23 g, 2 mmol) for 2b and benzyl cyanide (0.23 g, 2 mmol) for 2c under the influence of potassium hydroxide (0.073 g, 1.3 mmol). The reaction proceeded with constant agitation under ambient conditions for 12 h. The mixture had been stirred continuously for an additional 8 h after methyl iodide (0.28 gm, 2 mmol) being gradually introduced dropwise, resulting in a crystalline precipitate. The precipitate had been purified using ethanol after being separated *via* filtration and extensively washed using ether to give compounds 2a–c. The physical characteristics of 2a–c matched perfectly following the prior documented for 3-(methylthio)-2-phenyl-3-(phenylamino)acrylonitrile (2a), as white crystals in 62% yield, mp 118–120 °C,^53^ ethyl 2-cyano-3-(methylthio)-3-(phenylamino)acrylate (2b), as white crystals in 51.52% yield, mp 67–73 °C,^54^ and 2-((methylthio)(phenylamino)methylene)malononitrile (2c), as yellow crystals in 82% yield, mp 178–180 °C.^55^

#### 4.1.2 Preparation of N3,4-diphenyl-1*H*-pyrazole-3,5-diamine (3a), ethyl 5-amino-3-(phenylamino)-1*H*-pyrazole-4-carboxylate (3b) and 5-amino-3-(phenylamino)-1*H*-pyrazole-4-carbonitrile (3c)

To 250 mL of methanol one of compounds 2a–c (34 mmol) was added, reflux was applied for approximately 30 minutes until complete solubilization occurred. Hydrazine hydrate (1.6 mL, 34 mmol) had been gradually introduced into the warmed solution while maintaining continuous stirring for a further 30 min. The interaction had been subsequently maintained within a water bath 60–70 °C for 3 to 4 h, leading to the development of a precipitate. It was obtained through filtration, extensively rinsed *via* ether, and purified in ethanol to provide the compound (3a) as white crystalline powder in 60% yield, mp 118–122 °C, ^56,57^ the compound (3b) as white crystalline powder in 80% yield, mp 170 °C,^58^ and the compound (3c) as crystalline whitish grey powder in 80% yield, mp 205–208 °C.^59^

#### 4.1.3 Preparation of 3-(dimethylamino)-1-arylprop-2-en-1-ones 5a–j

A mixture of DMF-DMA (2.38 g, 20 mmol) in dry xylene (50 mL) received the addition of aryl ethanone 4a–j (20 mmol). Reflux was applied for 6–7 h, with progress assessed by TLC. Following reflux, the xylene had been distilled and trituration of crude product was carried out with diethyl ether (5 mL). The product had been purified and washed *via* cold petroleum ether then crystallized from ethanol to provide the products 5a–j. The compounds' physicochemical properties aligned with already reported findings.^28,60,61^

#### 4.1.4 Synthesis of N,3-diphenyl-7-(aryl)pyrazolo[1,5-*a*]pyrimidin-2-amine 6a–j

Acetic acid (25 mL) served as the reaction medium for dissolving (3a) (2.5 g, 10 mmol) and the associated enaminone 5a–j (10 mmol). The solution had refluxed for around 3 h and afterward underwent cooling. The precipitated solid has been obtained through filtration, extensively washed *via* ethanol, and subsequently dried. The final compounds, 6a–j, were obtained *via* crystallization from a dimethylformamide/water combination (v/v = 1 : 4).

##### 4.1.4.1 N,3,7-triphenylpyrazolo[1,5-*a*]pyrimidin-2-amine (6a)

Yellow powder, 60% yield; mp 116–117 °C; IR (KBr) *ν*_max_/cm^−1^ 3310 (NH), 1600 (C=N); ^1^H NMR *δ* 6.82 (t, *J* = 7.0 Hz, 1H, H4 of PhNH), 7.13 (d, *J* = 5.0 Hz, 1H, H3 of pyrimidine), 7.19 (t, *J* = 8.0 Hz, 2H, H3 and H3′ of PhNH), 7.30 (t, *J* = 7.5 Hz, 1H, H4 of Ph pyrazole), 7.47 (t, *J* = 7.5 Hz, 2H, H3 and H3′ of Ph pyrazole), 7.52 (d, *J* = 8.0 Hz, 2H, H2 and H2′ of PhNH), 7.63 (t, *J* = 3.0 Hz, 3H, H3, H3′and H4 of Ph), 7.83 (d, *J* = 7.0 Hz, 2H, H2 and H2′ of Ph pyrazole), 8.18–8.20 (m, 2H, H2 and H2′ of Ph), 8.50 (s, 1H, N**H**), 8.52 (d, *J* = 4.5 Hz, 1H, H2 of pyrimidine); ^13^C NMR *δ* 97.17 (C3 of pyrazolopyrimidine), 107.12, 117.41 (2C), 120.31, 126.40, 128.79 (2C), 128.90 (2C), 128.93 (2C), 128.98 (2C), 129.72 (2C), 131.24, 131.50, 131.83, 142.93, 145.11, 147.10, 150.15, 153.18. HRMS (ESI): *m*/*z* [M + H]^+^ calcd. 363.1531 and found 363.1624; Anal. Calcd. For: C_24_H_18_N_4_ (362.43): C, 79.54; H, 5.01; N, 15.64; Found: C, 79.31; H, 5.28; N, 15.90.

##### 4.1.4.2 N,3-diphenyl-7-(*p*-tolyl)pyrazolo[1,5-*a*]pyrimidin-2-amine (6b)

Yellow powder, 70% yield; mp 194–195 °C; IR (KBr) *ν*_max_/cm^−1^ 3300 (NH), 1600 (C=N); ^1^H NMR *δ* 3.38 (s, 3H, C**H**_3_), 6.81 (t, *J* = 8.0 Hz, 1H, H4 of PhNH), 7.12 (d, *J* = 5.5 Hz, 1H, H3 of pyrimidine), 7.20 (t, *J* = 7.5 Hz, 2H, H3 and H3′ of PhNH), 7.29 (t, *J* = 7.0 Hz, 1H, H4 of Ph), 7.43–7.48 (m, 4H, H2 and H2′ of PhNH and H3 and H3′ of Ph), 7.52 (d, *J* = 9.5 Hz, 2H, H2 and H2′ of Ph), 7.82 (d, *J* = 9.0 Hz, 2H, H3 and H3′ of Ar), 8.12 (d, *J* = 9.5 Hz, 2H, H2 and H2′ of Ar), 8.47 (s, 1H, N**H**), 8.49 (d, *J* = 8.5 Hz, 1H, H2 of pyrimidine); ^13^C NMR *δ* 21.49 (**C**H_3_), 93.03, 97.15 (C3 of pyrazolopyrimidine), 117.34 (2C), 128.74 (2C), 129.10 (4C), 129.17 (4C), 129.53 (4C), 129.58 (2C), 135.28 (2C). HRMS (ESI): *m*/*z* [M + H]^+^ calcd. 377.1688 and found 377.1782; Anal. Calcd. For: C_25_H_20_N_4_ (376.45): C, 79.76; H, 5.35; N, 14.88; Found: C, 79.94; H, 5.61; N, 15.09.

##### 4.1.4.3 7-(4-Methoxyphenyl)-N,3-diphenylpyrazolo[1,5-*a*]pyrimidin-2-amine (6c)

Light brown powder, 80% yield; mp 167–168 °C; IR (KBr) *ν*_max_/cm^−1^ 3300 (NH), 1610 (C=N); ^1^H NMR *δ* 3.89 (s, 3H, OC**H**_3_), 6.83 (t, *J* = 7.5 Hz, 1H, H4 of PhNH), 7.12 (d, *J* = 4.5 Hz, 1H, H3 of pyrimidine), 7.17–7.24 (m, 4H, H2, H2′, H3 and H3′ of PhNH), 7.29 (t, *J* = 8.0 Hz, 1H, H4 of Ph), 7.47 (t, *J* = 7.5 Hz, 2H, H3 and H3′ of Ph), 7.54 (d, *J* = 9.0 Hz, 2H, H3 and H3′ of Ar), 7.83 (d, *J* = 6.5 Hz, 2H, H2 and H2′ of Ph), 8.24 (d, *J* = 9.5 Hz, 2H, H2 and H2′ of Ar), 8.47–8.49 (m, 1H, H2 of pyrimidine and 1H, N**H**); ^13^C NMR *δ* 55.90 (O**C**H_3_), 97.01 (C3 of pyrazolopyrimidine), 106.30, 114.34 (2C), 117.34 (2C), 120.45, 123.10, 126.48, 128.75 (2C), 128.96 (2C), 129.10 (2C), 131.49 (2C), 131.71, 142.83, 144.87, 147.14, 150.04, 153.03, 161.81. HRMS (ESI): *m*/*z* [M + H]^+^ calcd. 393.1637 and found 393.1726; Anal. Calcd. For: C_25_H_20_N_4_O (392.45): C, 76.51; H, 5.14; N, 14.28; Found: C, 76.78; H, 5.25; N, 14.47.

##### 4.1.4.4 7-(3,4-Dimethoxyphenyl)-N,3-diphenylpyrazolo[1,5-*a*]pyrimidin-2-amine (6d)

Orange powder, 90% yield; mp 239–241 °C; IR (KBr) *ν*_max_/cm^−1^ 3330 (NH), 1590 (C=N); ^1^H NMR *δ* 3.85 (s, 3H, OC**H**_3_), 3.89 (s, 3H, OC**H**_3_), 6.85 (t, *J* = 9.0 Hz, 1H, H4 of PhNH), 7.16 (d, *J* = 5.0 Hz, 1H, H3 of pyrimidine), 7.21 (t, *J* = 8.5 Hz, 3H, H3 and H3′ of PhNH and H5 of Ar), 7.30 (t, *J* = 7.5 Hz, 1H, H4 of Ph), 7.48 (t, *J* = 8.0 Hz, 2H, H3 and H3′ of Ph), 7.61 (d, *J* = 7.0 Hz, 2H, H2 and H2′ of PhNH), 7.76 (d, *J* = 11.0 Hz, 1H, H6 of Ar), 7.83 (d, *J* = 7.0 Hz, 2H, H2 and H2′ of Ph), 8.03 (s, 1H, H2 of Ar), 8.46 (s, 1H, N**H**), 8.48 (d, *J* = 5.0 Hz, 1H, H2 of pyrimidine); ^13^C NMR *δ* 56.08 (2C) (O**C**H_3_), 70.95 (2C), 84.79 (2C), 86.85 (C3 of pyrazolopyrimidine), 117.76 (2C), 128.84 (4C), 129.12 (4C), 177.08 (3C), 178.85 (2C), 194.01 (4C). HRMS (ESI): *m*/*z* [M + H]^+^ calcd. 423.1743 and found 423.1840; Anal. Calcd. For: C_26_H_22_N_4_O_2_ (422.48): C, 73.92; H, 5.25; N, 13.26; Found: C, 73.80; H, 5.41; N, 13.50.

##### 4.1.4.5 7-(4-Chlorophenyl)-N,3-diphenylpyrazolo[1,5-*a*]pyrimidin-2-amine (6e)

Yellow powder, 87% yield; mp 162–163 °C; IR (KBr) *ν*_max_/cm^−1^ 3420 (NH), 1600 (C=N); ^1^H NMR *δ* 6.83 (t, *J* = 7.5 Hz, 1H, H4 of PhNH), 7.15 (d, *J* = 4.0 Hz, 1H, H3 of pyrimidine), 7.21 (t, *J* = 7.5 Hz, 2H, H3 and H3′ of PhNH), 7.30 (t, *J* = 7.0 Hz, 1H, H4 of Ph), 7.45–7.51 (m, 4H, H2 and H2′ of PhNH and H3 and H3′ of Ph), 7.69 (d, *J* = 8.0 Hz, 2H, H2 and H2′ of Ph), 7.81 (d, *J* = 7.0 Hz, 2H, H3 and H3′ of Ar), 8.24 (d, *J* = 7.0 Hz, 2H, H2 and H2′ of Ar), 8.48 (s, 1H, N**H**), 8.50 (d, *J* = 5.0 Hz, 1H, H2 of pyrimidine); ^13^C NMR *δ* 97.26 (C3 of pyrazolopyrimidine), 107.31, 117.47 (2C), 120.11, 126.74, 128.78 (2C), 128.96 (2C), 129.03 (2C), 129.07 (2C), 129.94, 131.58 (2C), 136.31, 142.75 (2C), 143.96, 147.02, 150.19, 153.53. HRMS (ESI): *m*/*z* [M + H]^+^ calcd. 397.1142 and found 397.1227; Anal. Calcd. For: C_24_H_17_ClN_4_ (396.87): C, 72.63; H, 4.32; N, 14.12; Found: C, 72.86; H, 4.50; N, 14.36.

##### 4.1.4.6 7-(4-Bromophenyl)-N,3-diphenylpyrazolo[1,5-*a*]pyrimidin-2-amine (6f)

Yellow powder, 78% yield; mp 158–159 °C; IR (KBr) *ν*_max_/cm^−1^ 3400 (NH), 1600 (C=N); ^1^H NMR *δ* 6.82 (t, *J* = 7.5 Hz, 1H, H4 of PhNH), 7.15 (d, *J* = 5.0 Hz, 1H, H3 of pyrimidine), 7.22 (t, *J* = 6.5 Hz, 2H, H3 and H3′ of PhNH), 7.30 (t, *J* = 7.0 Hz, 1H, H4 of Ph), 7.47 (t, *J* = 8.0 Hz, 2H, H3 and H3′ of Ph), 7.51 (d, *J* = 7.5 Hz, 2H, H2 and H2′ of PhNH), 7.82–7.86 (m, 4H, H3 and H3′ of Ar and H2 and H2′ of Ph), 8.15 (d, *J* = 6.5 Hz, 2H, H2 and H2′ of Ar), 8.50–8.53 (m, 1H, H2 of pyrimidine and 1H, N**H**); ^13^C NMR *δ* 97.26 (C3 of pyrazolopyrimidine), 107.05, 117.47 (2C), 120.47, 125.04, 126.52, 128.78 (2C), 128.94 (2C), 129.06, 130.31, 131.64 (2C), 131.73 (2C), 131.94 (2C), 142.76, 143.96, 147.02, 150.15, 153.20. HRMS (ESI): *m*/*z* [M + H]^+^ calcd. 441.0637 and found 441.0725; Anal. Calcd. For: C_24_H_17_BrN_4_ (441.32): C, 65.32; H, 3.88; N, 12.70; Found: C, 65.48; H, 4.02; N, 12.97.

##### 4.1.4.7 7-(4-Nitrophenyl)-N,3-diphenylpyrazolo[1,5-*a*]pyrimidin-2-amine (6g)

Red powder, 80% yield; mp 198–200 °C; IR (KBr) *ν*_max_/cm^−1^ 3390 (NH), 1600 (C=N), 1350 (NO_2_); ^1^H NMR *δ* 6.83 (t, *J* = 7.5 Hz, 1H, H4 of PhNH), 7.21 (t, *J* = 8.0 Hz, 2H, H3 and H3′ of PhNH), 7.24 (t, *J* = 4.5 Hz, 1H, H3 of pyrimidine), 7.31 (t, *J* = 7.5 Hz, 1H, H4 of Ph), 7.46–7.51 (m, 4H, H2 and H2′ of PhNH and H3 and H3′ of Ph), 7.82 (d, *J* = 7.5 Hz, 2H, H2 and H2′ of Ph), 8.46 (s, 4H, H2, H2′, H3 and H3′ of Ar), 8.55 (s, 1H, N**H**), 8.58 (d, *J* = 8.5 Hz, 1H, H2 of pyrimidine); ^13^C NMR *δ* 117.54 (C3 of pyrazolopyrimidine), 123.89 (2C), 128.78 (4C), 129.02 (4C), 129.12 (4C), 131.19 (4C), 137.49 (2C), 142.64 (2C), 148.82. HRMS (ESI): *m*/*z* [M + H]^+^ calcd. 408.1382 and found 408.1475; Anal. Calcd. For: C_24_H_17_N_5_O (407.42): C, 70.75; H, 4.21; N, 17.19; Found: C, 70.99; H, 4.37; N, 17.40.

##### 4.1.4.8 7-(Naphthalen-2-yl)-N,3-diphenylpyrazolo[1,5-*a*]pyrimidin-2-amine (6h)

Yellow powder, 82% yield; mp 184–186 °C; IR (KBr) *ν*_max_/cm^−1^ 3420 (NH), 1600 (C=N); ^1^H NMR *δ* 6.84 (t, *J* = 7.5 Hz, 1H, H4 of PhNH), 7.21 (t, *J* = 8.0 Hz, 2H, H3 and H3′ of PhNH), 7.30–7.33 (m, 2H, H3 of pyrimidine and H4 of Ph), 7.49 (t, *J* = 7.5 Hz, 2H, H3 and H3′ of Ph), 7.61 (d, *J* = 8.0 Hz, 2H, H2 and H2′ of PhNH), 7.66–7.71 (m, 2H of Ar), 7.86 (d, *J* = 7.0 Hz, 2H, H2 and H2′ of Ph), 8.05–8.10 (m, 3H of Ar), 8.14 (d, *J* = 8.5 Hz, 1H of Ar), 8.23–8.26 (m, 1H of Ar), 8.57 (s, 1H, N**H**), 8.58 (d, *J* = 3.5 Hz, 1H, H2 of pyrimidine); ^13^C NMR *δ* 96.99 (C3 of pyrazolopyrimidine), 107.28, 117.64 (2C), 120.63, 126.12 (2C), 127.52, 128.14, 128.32, 128.40 (2C), 128.51, 128.80 (2C), 129.01 (4C), 129.21, 130.29, 132.69, 134.24, 142.70, 144.87, 147.11, 150,21, 153.22. HRMS (ESI): *m*/*z* [M + H]^+^ calcd. 413.1688 and found 413.1784; Anal. Calcd. For: C_28_H_20_N_4_ (412.49): C, 81.53; H, 4.89; N, 13.58; Found: C, 81.29; H, 4.95; N, 13.79.

##### 4.1.4.9 7-(Furan-2-yl)-N,3-diphenylpyrazolo[1,5-*a*]pyrimidin-2-amine (6i)

Brown, 91% yield; mp 166–167 °C; IR (KBr) *ν*_max_/cm^−1^ 3400 (NH), 1600 (C=N); ^1^H NMR *δ* 6.95 (t, *J* = 4.0 Hz, 1H, H4 of PhNH), 6.95–6.96 (m, 1H, H4 of furan), 7.30 (t, *J* = 8.0 Hz, 3H, H3 and H3′ of PhNH and H4 of Ph), 7.36 (d, *J* = 5.0 Hz, 1H, H3 of pyrimidine), 7.48 (t, *J* = 7.5 Hz, 2H, H3 and H3′ of Ph), 7.63 (d, *J* = 8.0 Hz, 2H, H2 and H2′ of PhNH), 7.85 (d, *J* = 7.0 Hz, 2H, H2 and H2′ of Ph), 8.09 (d, *J* = 7.0 Hz, 1H, H3 of furan), 8.18 (s, 1H, H5 of furan), 8.52 (d, *J* = 5.0 Hz, 1H, H2 of pyrimidine), 8.60 (s, 1H, N**H**); ^13^C NMR *δ* 96.73 (C3 of pyrazolopyrimidine), 101.98, 113.74, 117.76 (2C), 119.23, 120.96, 126.63, 128.80 (2C), 128.99 (2C), 129.20 (2C), 131.41, 134.25, 142.52, 143.63, 146.76, 147.40, 149.25, 153.49. HRMS (ESI): *m*/*z* [M + H]^+^ calcd. 353.1324 and found 353.1415; Anal. Calcd. For: C_22_H_16_N_4_O (352.39): C, 74.98; H, 4.58; N, 15.90; Found: C, 74.76; H, 4.67; N, 16.08.

##### 4.1.4.10 N,3-diphenyl-7-(thiophen-2-yl)pyrazolo[1,5-*a*]pyrimidin-2-amine (6j)

Orange powder, 84% yield; mp 168–170 °C; IR (KBr) *ν*_max_/cm^−1^ 3400 (NH), 1600 (C=N); ^1^H NMR *δ* 6.91 (t, *J* = 8.0 Hz, 1H, H4 of PhNH), 7.31 (t, *J* = 7.5 Hz, 3H, H3 and H3′ of PhNH and H4 of Ph), 7.41 (t, *J* = 5.0 Hz, 1H, H4 of thiophene), 7.49 (t, *J* = 8.0 Hz, 2H, H3 and H3′ of Ph), 7.64 (d, *J* = 6.0 Hz, 1H, H3 of pyrimidine), 7.78 (d, *J* = 8.0 Hz, 2H, H2 and H2′ of PhNH), 7.84 (d, *J* = 6.0 Hz, 2H, H2 and H2′ of Ph), 8.15 (d, *J* = 5.5 Hz, 1H, H5 of thiophene), 8.49 (m, 1H, H2 of pyrimidine and 1H, N**H**), 8.59 (s, 1H, H3 of thiophene); ^13^C NMR *δ* 96.50 (C3 of pyrazolopyrimidine), 103.23, 117.77 (2C), 120.64, 125.12, 126.45, 128.18, 128.95, 128.97 (2C), 129.10 (2C), 130.50, 131.74, 132.06, 134.92, 138.69, 142.50, 147.01, 149.31, 153.01. HRMS (ESI): *m*/*z* [M + H]^+^ calcd. 369.1069 and found 369.1188; Anal. Calcd. For: C_22_H_16_N_4_S (368.45): C, 71.71; H, 4.38; N, 15.21; Found: C, 71.88; H, 4.50; N, 15.43.

#### 4.1.5. 2-(Arylidene)malononitrile 8a–e preparation

Ethanol (10 mL) was served as the solvent for aryl aldehyde 7a–e (10 mmol), to which malononitrile (0.66 g, 10 mmol) and piperidine (0.01 mL, 1 mmol) have been incorporated. This mixture was maintained under stirring at 25 °C for 5 h. The product had been filtered and crystallized from *n*-hexane and ethanol mixture (v/v = 10 : 1) to obtain the pure compounds 8a–e. The physical properties of 8a–e matched those already reported.^29–31^

#### 4.1.6 Preparation of 7-amino-5-(aryl)-2-(phenylamino)pyrazolo[1,5-*a*]pyrimidine-6-carbonitrile 9a–d

Ethanol (20 mL), along with a catalytic quantity of piperidine (0.01 mL, 1 mmol), had been employed to dissolve a mixture of the suitable 2-(arylidene)malononitrile 8a–e (10 mmol) and 5-amino-3-(phenylamino)-1*H*-pyrazole-4-carbonitrile (3c) (1.99 g, 10 mmol) or ethyl 5-amino-3-(phenylamino)-1*H*-pyrazole-4-carboxylate (3b) (2.46 g, 10 mmol). Reflux was applied for approximately 8 to 12 h. Subsequent to cooling down, the resultant had been crystallized and purified *via* ethanol to get the corresponding 7-amino-3-(substituted)-5-(aryl)-2-(phenylamino)pyrazolo[1,5-*a*]pyrimidine-6-carbonitrile 9a–d.

##### 4.1.6.1. 7-Amino-5-phenyl-2-(phenylamino)pyrazolo[1,5-*a*]pyrimidine-3,6-dicarbonitrile (9a)

Brown powder, 80% yield; mp over 300 °C; IR (KBr) *ν*_max_/cm^−1^ 3300–3410 (NH,NH_2_), 2215 (2C≡N), 1620 (C=N); ^1^H NMR *δ* 6.99 (t, *J* = 7.5 Hz, 1H, H4 of PhNH), 7.32 (t, *J* = 8.0 Hz, 2H, H3 and H3′ of PhNH), 7.57–7.59 (m, 3H, H2 and H2′ of PhNH and H4 of Ph), 7.84–7.86 (m, 2H, H3 and H3′ of Ph), 7.95 (d, *J* = 8.0 Hz, 2H, H2 and H2′ of Ph), 9.01 (s, 2H, N**H**_2_), 9.51 (s, 1H, N**H**); ^13^C NMR *δ* 70.69 (C3 of pyrazolopyrimidine), 76.22 (C6 of pyrazolopyrimidine), 113.80, 116.33, 118.67, 121.89, 125.12, 126.15, 128.89 (2C), 129.11 (2C), 129.23, 131.13, 137.10, 140.76, 149.95, 151.34, 156.17 (C5 of pyrazolopyrimidine), 162.67 (C7 of pyrazolopyrimidine). HRMS (ESI): *m*/*z* [M + H]^+^ calcd. 352.1232 and found 352.1319; Anal. Calcd. For: C_20_H_13_N_7_ (351.36): C, 68.37; H, 3.73; N, 27.90; Found: C, 68.60; H, 3.84; N, 27.72.

##### 4.1.6.2. 7-Amino-5-(4-methoxyphenyl)-2-(phenylamino)pyrazolo[1,5-*a*]pyrimidine-3,6-dicarbonitrile (9b)

Yellow powder, 75% yield; mp over 300 °C; IR (KBr) *ν*_max_/cm^−1^ 3310–3410 (NH,NH_2_), 2210 (2C≡N), 1620 (C=N); ^1^H NMR *δ* 3.87 (s, 3H, OC**H**_3_), 6.98 (t, *J* = 7.5 Hz, 1H, H4 of PhNH), 7.12 (d, *J* = 9.0 Hz, 2H, H3 and H3′ of Ar), 7.31 (t, *J* = 8.0 Hz, 2H, H3 and H3′ of PhNH), 7.86 (d, *J* = 9.0 Hz, 2H, H2 and H2′ of PhNH), 7.94 (d, *J* = 8.0 Hz, 2H, H2 and H2′ of Ar), 8.94 (s, 2H, N**H**_2_), 9.48 (s, 1H, N**H**); ^13^C NMR *δ* 55.89 (OCH_3_), 70.38 (C3 of pyrazolopyrimidine), 75.54 (C6 of pyrazolopyrimidine), 93.44, 102.00, 113.88 (2C), 114.28 (2C), 118.65, 121.93, 129.26, 130.91 (2C), 140.73, 150.03 (2C), 151.33, 156.17, 161.76 (C5 of pyrazolopyrimidine), 161.99 (C7 of pyrazolopyrimidine). HRMS (ESI): *m*/*z* [M + H]^+^ calcd. 382.1338 and found 382.1422; Anal. Calcd. For: C_21_H_15_N_7_O (381.39): C, 66.13; H, 3.96; N, 25.71; Found: C, 66.41; H, 4.09; N, 25.87.

##### 4.1.6.3. 7-Amino-5-(4-fluorophenyl)-2-(phenylamino)pyrazolo[1,5-*a*]pyrimidine-3,6-dicarbonitrile (9c)

Yellow powder, 78% yield; mp over 300 °C; IR (KBr) *ν*_max_/cm^−1^ 3300–3420 (NH,NH_2_), 2210 (2C≡N), 1610 (C=N); ^1^H NMR *δ* 6.99 (t, *J* = 7.5 Hz, 1H, H4 of PhNH), 7.32 (t, *J* = 8.0 Hz, 2H, H3 and H3′ of PhNH), 7.42 (t, *J* = 9.0 Hz, 2H, H2 and H2′ of PhNH), 7.91–7.96 (m, 4H, H2, H2′, H3 and H3′ of Ar), 9.03 (s, 2H, N**H**_2_), 9.51 (s, 1H, N**H**); ^13^C NMR *δ* 70.72 (C3 of pyrazolopyrimidine), 76.17 (C6 of pyrazolopyrimidine), 113.75, 115.83, 116.05 (2C), 116.29, 118.67 (2C), 121.93, 129.24 (2C), 131.62 (2C), 131.71, 140.72, 149.90, 151.28, 156.17 (C5 of pyrazolopyrimidine), 161.56 (C7 of pyrazolopyrimidine). MS *m*/*z* (%) 371.35 (M^+^ +2, 30.75), 370.41 (M^+^ + H, 30.37), 369.26 (M^+^, 100); Anal. Calcd. For: C_20_H_12_FN_7_ (369.35): C, 65.04; H, 3.27; N, 26.55; Found: C, 65.32; H, 3.58; N, 26.73.

##### 4.1.6.4 Ethyl 7-amino-6-cyano-5-(4-methoxyphenyl)-2-(phenylamino)pyrazolo[1,5-*a*]pyrimidine-3-carboxylate (9d)

Grayish brown powder, 78% yield; mp 250–251 °C; IR (KBr) *ν*_max_/cm^−1^ 3300–3410 (NH,NH_2_), 2200 (C≡N), 1660 (C=O), 1620 (C=N); ^1^H NMR *δ* 1.35 (t, *J* = 8.5 Hz, 3H, C**H**_3_), 3.87 (s, 3H, OC**H**_3_), 4.34 (q, *J* = 6.5 Hz, 2H, C**H**_2_), 7.0 (t, *J* = 8.5 Hz, 1H, H4 of PhNH), 7.12 (d, *J* = 11.0 Hz, 2H, H3 and H3′ of Ar), 7.35 (t, *J* = 8.5 Hz, 2H, H3 and H3′ of PhNH), 7.89 (d, *J* = 6.0 Hz, 2H, H2 and H2′ of PhNH), 7.94 (d, *J* = 8.0 Hz, 2H, H2 and H2′ of Ar), 8.86 (s, 2H, N**H**_2_), 9.05 (s, 1H, N**H**); ^13^C NMR *δ* 14.75 (–**C**H_3_), 55.89 (O**C**H_3_), 60.28 (–**C**H_2_), 87.80 (C3 of pyrazolopyrimidine), 97.50 (C6 of pyrazolopyrimidine), 114.22 (2C), 116.88 (2C), 118.28 (2C), 121.80, 126.86, 129.52 (2C), 129.68, 130.89 (2C), 140.13, 147.54, 156.86 (**C**=O), 161.27 (C5 of pyrazolopyrimidine), 161.70 (C7 of pyrazolopyrimidine). MS *m*/*z* (%) 429.91 (M^+^ +H, 36.63), 428.30 (M^+^, 57.60); Anal. Calcd. For: C_23_H_20_N_6_O_3_ (428.44): C, 64.48; H, 4.71; N, 19.62; Found: C, 64.67; H, 4.83; N, 19.89.

#### 4.1.7 Synthesis of 7-amino-5-(aryl)-2-(phenylamino)-4,5-dihydropyrazolo[1,5-*a*]pyrimidine-6-carbonitrile 9e–h

Following the previous method, a mixture of N3,4-diphenyl-1*H*-pyrazole-3,5-diamine (3a) (2.50 g, 10 mmol) or ethyl 5-amino-3-(phenylamino)-1*H*-pyrazole-4-carboxylate (3b) (2.46 g, 10 mmol) and the appropriate 2-(arylidene)malononitrile 8a–e (10 mmol) was dissolved in ethanol (20 mL) along with a catalytic quantity of piperidine (0.01 mL, 1 mmol). The reaction had been subjected to reflux for a duration of 8 to 12 h. Subsequent to cooling down, the product had been purified *via* ethanol to get the equivalent ethyl 7-amino-5-(aryl)-6-cyano-2-(phenylamino)-4,5-dihydropyrazolo[1,5-*a*]pyrimidine-3-carboxylate 9e–h.

##### 4.1.7.1. 7-Amino-5-(2,4-dichlorophenyl)-3-phenyl-2-(phenylamino)-4,5-dihydropyrazolo[1,5-*a*]pyrimidine-6-carbonitrile (9e)

Pale yellow powder, 78% yield; mp 241–242 °C; IR (KBr) *ν*_max_/cm^−1^ 3320–3450 (NH,NH_2_), 2200 (C≡N), 1600 (C=N); ^1^H NMR *δ* 5.55 (s, 1H, H2 of pyrimidine), 6.84 (t, *J* = 7.0 Hz, 1H, H4 of PhNH), 6.96 (s, 2H, N**H**_2_), 7.20 (t, *J* = 8.0 Hz, 2H, H3 and H3′ of PhNH), 7.26 (t, *J* = 7.0 Hz, 1H, H4 of Ph), 7.34–7.47 (m, 6H, H2, H2′, H3 and H3′ of Ph and H5 of Ar), 7.61–7.66 (m, 4H, H2 and H2′ of PhNH, H6 of Ar and N**H** of pyrimidine), 7.84 (s, 1H, N**H**); ^13^C NMR *δ* 74.30 (C5 of pyrazolopyrimidine), 92.68 (C6 of pyrazolopyrimidine), 117.73 (C3 of pyrazolopyrimidine), 118.17, 120.73, 126.92, 128.48, 129.18, 129.35, 130.33, 130.85, 132.39, 133.05, 135.30, 136.54, 140.62, 142.11, 142.52, 142.77, 144.64, 148.36, 149.09, 151.90, 154.51, 157.91 (C7 of pyrazolopyrimidine). MS *m*/*z* (%) 475.32 (M^+^ +2, 24.35), 474.38 (M^+^ +H, 35.26), 473.42 (M^+^, 38.14); Anal. Calcd. For: C_25_H_18_Cl_2_N_6_ (473.36): C, 63.43; H, 3.83; N, 17.75; Found: C, 63.61; H, 3.98; N, 17.97.

##### 4.1.7.2 Ethyl 7-amino-6-cyano-5-phenyl-2-(phenylamino)-4,5-dihydropyrazolo[1,5-*a*]pyrimidine-3-carboxylate (9f)

Off white powder, 92% yield; mp 230–233 °C; IR (KBr) *ν*_max_/cm^−1^ 3300–3420 (NH,NH_2_), 2200 (C≡N), 1700 (C=O), 1650 (C=N); ^1^H NMR *δ* 1.34 (t, *J* = 8.5 Hz, 3H, C**H**_3_), 4.28 (q, *J* = 8.5 Hz, 2H, C**H**_2_), 5.25 (d, *J* = 8.5 Hz, 1H, H2 of pyrimidine), 6.92 (t, *J* = 7.0 Hz, 1H, H4 of PhNH), 7.13 (s, 2H, N**H**_2_), 7.26–7.36 (m, 5H, H3 and H3′ of PhNH and H3, H3′ and H4 of Ph), 7.38 (d, *J* = 7.0 Hz, 2H, H2 and H2′ of Ph), 7.68 (d, *J* = 7.0 Hz, 2H, H2 and H2′ of PhNH), 8.12 (d, *J* = 7.0 Hz, 1H, N**H** of pyrimidine), 8.29 (s, 1H, N**H**); ^13^C NMR *δ* 14.86 (–**C**H_3_), 60.37 (C5 of pyrazolopyrimidine), 82.87 (–**C**H_2_), 85.00 (C6 of pyrazolopyrimidine), 117.30 (C3 of pyrazolopyrimidine), 117.76 (2C), 122.40 (2C), 125.94 (2C), 129.34 (2C), 129.51 (2C), 140.90 (2C), 143.67, 147.94 (2C), 156.40 (C7 of pyrazolopyrimidine), 165.90 (**C**=O). MS *m*/*z* (%) 429.91 (M^+^+H, 36.63), 428.30 (M^+^, 57.60); Anal. Calcd. For: C_23_H_20_N_6_O_3_ (428.44): C, 64.48; H, 4.71; N, 19.62; Found: C, 64.67; H, 4.83; N, 19.89. MS *m*/*z* (%) 401.39 (M^+^ +H, 34.91), 400.42 (M^+^, 100); Anal. Calcd. For: C_22_H_20_N_6_O_2_ (400.43): C, 65.99; H, 5.03; N, 20.99; Found: C, 66.12; H, 5.24; N, 20.78.

##### 4.1.7.3 Ethyl 7-amino-6-cyano-5-(4-fluorophenyl)-2-(phenylamino)-4,5-dihydropyrazolo[1,5-*a*]pyrimidine-3-carboxylate (9g)

Yellow powder, 89% yield; mp 294–295 °C; IR (KBr) *ν*_max_/cm^−1^ 3290–3420 (NH,NH_2_), 2200 (C≡N), 1700 (C=O), 1660 (C=N); ^1^H NMR *δ* 1.31 (t, *J* = 9.5 Hz, 3H, C**H**_3_), 4.32 (q, *J* = 9.5 Hz, 2H, C**H**_2_), 5.28 (d, *J* = 6.5 Hz, 1H, H2 of pyrimidine), 6.93 (t, *J* = 7.5 Hz, 1H, H4 of PhNH), 7.17 (s, 2H, N**H**_2_), 7.22 (t, *J* = 8.5 Hz, 2H, H3 and H3′ of PhNH), 7.28 (t, *J* = 9.0 Hz, 2H, H3 and H3′ of Ar), 7.35–7.38 (m, 2H, H2 and H2′ of Ar), 7.67 (d, *J* = 7.0 Hz, 2H, H2 and H2′ of PhNH), 8.12 (d, *J* = 6.5 Hz, 1H, N**H** of pyrimidine), 8.23 (s, 1H, N**H**); ^13^C NMR *δ* 14.66 (–**C**H_3_), 59.96 (C5 of pyrazolopyrimidine), 60.80 (–**C**H_2_), 88.42 (C6 of pyrazolopyrimidine), 115.97 (C3 of pyrazolopyrimidine), 117.02 (2C), 118.25 (3C), 129.61 (4C), 131.57 (2C), 131.66 (2C), 139.96 (2C), 150.12 (C7 of pyrazolopyrimidine), 192.00 (**C**=O). HRMS (ESI): *m*/*z* [M + H]^+^ calcd. 419.1554 and found 419.1640; Anal. Calcd. For: C_22_H_19_FN_6_O_2_ (418.42): C, 63.15; H, 4.58; N, 20.08; Found: C, 63.41; H, 4.69; N, 19.87.

##### 4.1.7.4 Ethyl 7-amino-6-cyano-5-(2,4-dichlorophenyl)-2-(phenylamino)-4,5-dihydropyrazolo[1,5-*a*]pyrimidine-3-carboxylate (9h)

Yellow powder, 79% yield; mp 215–216 °C; IR (KBr) *ν*_max_/cm^−1^ 3300–3450 (NH,NH_2_), 2200 (C≡N), 1700 (C=O), 1610 (C=N); ^1^H NMR *δ* 1.29 (t, *J* = 7.0 Hz, 3H, C**H**_3_), 4.27 (q, *J* = 7.0 Hz, 2H, C**H**_2_), 5.73 (d, *J* = 6.5 Hz, 1H, H2 of pyrimidine), 6.93 (t, *J* = 7.0 Hz, 1H, H4 of PhNH), 7.22 (s, 2H, N**H**_2_), 7.29 (t, *J* = 8.0 Hz, 2H, H3 and H3′ of PhNH), 7.38 (d, *J* = 8.5 Hz, 1H, H5 of Ar), 7.47 (d, *J* = 7.0 Hz, 1H, H3 of Ar), 7.66 (s, 1H, H6 of Ar), 7.69 (d, *J* = 7.5 Hz, 2H, H2 and H2′ of PhNH), 7.94 (s, 1H, N**H** of pyrimidine), 8.30 (s, 1H, N**H**); ^13^C NMR *δ* 14.88 (–**C**H_3_), 51.30 (C5 of pyrazolopyrimidine), 52.94 (–**C**H_2_), 60.24 (C6 of pyrazolopyrimidine), 82.88 (C3 of pyrazolopyrimidine), 117.83 (2C), 119.53, 121.38, 128.53, 129.41 (2C), 129.75, 130.36, 132.52, 133.77, 139.92, 140.38, 147.21, 147.94, 152.63 (C7 of pyrazolopyrimidine), 163.78 (**C**=O). HRMS (ESI): *m*/*z* [M + H]^+^ calcd. 469.0868 and found 469.0952; Anal. Calcd. For: C_22_H_18_Cl_2_N_6_O_2_ (469.32): C, 56.30; H, 3.87; N, 17.91; Found: C, 56.53; H, 3.98; N, 18.13.

##### 4.1.8 Synthesis of (*E*)-*N*′-(4-cyano-3-(phenylamino)-1*H*-pyrazol-5-yl)-*N*,*N*-dimethylformimidamide (10)

A DMF-DMA solution (2.0 mL, 15 mmol) had been combined with compound 3c (2.0 g, 10 mmol), reflux was applied for 1 h. Afterwards undergoing cooling. The product had been gathered *via* filtration then crystallized from hexane to produce the expected product (10) as white powder, 70% yield; mp 174–175 °C.

#### 4.1.9 Synthesis of 4-imino-N3-phenyl-1*H*-pyrazolo[3,4-*d*]pyrimidine-3,5(4*H*)-diamine (11)

A hydrazine hydrate solution (80%, 0.75 mL, 15 mmol) was combined with compound 10 (2.0 g, 10 mmol), reflux was applied for approximately 1 h. Afterwards undergoing cooling. The resultant precipitate had been crystallized from ethanol to afford compound 11 as faint pink powder, 75% yield; mp 240–243 °C; IR (KBr) *ν*_max_/cm^−1^ 3300–3600 (NH,NH_2_), 1600 (C=N); ^1^H NMR *δ* 3.79 (s, 2H, N**H**_2_), 5.62 (br. s, 1H, N**H**), 6.82 (t, *J* = 8 Hz, 1H, H4 of PhNH), 7.26 (t, *J* = 8.4 Hz, 2H, H3 and H3′ of PhNH), 7.59 (d, *J* = 8.4 Hz, 2H, H2 and H2′ of PhNH), 8.05 (s, 1H, C**H** pyrimidine), 9.02 (br. s, 1H, N**H**), 11.66 (br. s, 1H, N**H**); ^13^C NMR *δ* 107.31, 116.51 (2C), 120.26, 129.31 (2C), 129.42, 132.48, 142.05, 153.53, 156.03 (C4 of pyrazolopyrimidine). MS *m*/*z* (%) 242.55 (M^+^+H, 10.03), 241.25 (M^+^, 47.38); Anal. Calcd. For: C_11_H_11_N_7_ (241.25): C, 54.76; H, 4.60; N, 40.64; Found: C, 54.98; H, 4.81; N, 40.73.

#### 4.1.10 Synthesis of 4-imino-5-phenyl-3-(phenylamino)-4,5-dihydro-1*H*-pyrazolo[3,4-*d*]pyrimidine-6-thiol (12)

A pyridine solution (20 mL) including compound 3c (2.0 g, 10 mmol) and phenyl isothiocyanate (1.19 mL, 10 mmol), reflux was applied for approximately 1 h. After that, it was cooled and carefully poured over ice-water. The precipitated solid has been isolated *via* filtration, thoroughly dried, and subsequently purified by crystallization using ethanol to obtain the titled compound 4-imino-5-phenyl-3-(phenylamino)-4,5-dihydro-1*H*-pyrazolo[3,4-*d*]pyrimidine-6-thiol (12) as off white powder in 65% yield; mp 276–277 °C; IR (KBr) *ν*_max_/cm^−1^ 3150–3600 (NH); ^1^H NMR *δ* 6.84 (t, *J* = 7.70 Hz, 1H of Ar), 7.01 (t, *J* = 7.60 Hz, 1H of Ar), 7.18 (t, *J* = 7.40 Hz, 2H of Ar), 7.24 (t, *J* = 7.60 Hz, 2H of Ar), 7.49 (d, *J* = 9.60 Hz, 2H of Ar), 7.64 (d, *J* = 10.0 Hz, 2H of Ar), 8.70 (br. s, 1H, N**H**), 9.03 (br. s, 1H, N**H**), 12.86 (br. s, 1H, N**H**) 15.50 (br. s, 1H, S**H**); ^13^C NMR *δ* 91.09, 117.13 (2C), 120.49, 122.36 (2C), 124.28, 128.83 (2C), 129.26 (2C), 138.73, 143.17, 143.65, 154.00, 156.12 (C4 of pyrazolopyrimidine), 166.17 (C6 of pyrazolopyrimidine). MS *m*/*z* (%) 335.34 (M^+^ +H, 21.44), 334.36 (M^+^, 100); Anal. Calcd. For: C_17_H_14_N_6_S (334.40): C, 61.06; H, 4.22; N, 25.13; Found: C, 60.97; H, 4.35; N, 25.41.

#### 4.1.11 Preparation of 5-amino-3,6-bis(phenylamino)-1*H*-pyrazolo[3,4-*d*]pyrimidin-4(5*H*)-one (13)

A pyridine solution (20 mL) including compound 3b (2.46 g, 10 mmol) and phenyl isothiocyanate (1.19 mL, 10 mmol), reflux was applied for 2 h then a hydrazine hydrate solution (80%, 0.75 mL, 15 mmol) was added. Reflux was applied for extra 1 h, After that, it was cooled, the solid isolated *via* filtration, dried and purified by crystallization using ethanol to obtain the matching compound 5-amino-3,6-bis(phenylamino)-1*H*-pyrazolo[3,4-*d*]pyrimidin-4(5*H*)-one (13) as grayish white powder in 85% yield; mp over 300 °C; IR (KBr) *ν*_max_/cm^−1^ 3100–3600 (NH, NH_2_), 1690 (C=O), 1610 (C=N); ^1^H NMR *δ* 6.83 (m, 2H, N**H**_2_), 7.10 (t, *J* = 6.6 Hz, 1H, of PhNH), 7.22–7.26 (m, 2H, of PhNH), 7.35 (t, *J* = 8.8 Hz, 2H, of PhNH), 7.65 (d, *J* = 6.8 Hz, 2H, of PhNH), 7.75–7.78 (m, 4H, of PhNH and N**H**), 9.52 (br. s, 1H, N**H**), 12.20 (br. s, 1H, N**H**); ^13^C NMR *δ* 89.07, 116.80 (2C), 120.26 (2C), 121.96, 124.25, 129.15 (4C), 129.21 (2C), 138.37, 142.09, 151.75, 158.30 (–**C**=O). HRMS (ESI): *m*/*z* [M + H]^+^ calcd. 334.1338 and found 334.1421; Anal. Calcd. For: C_17_H_15_N_7_O (333.35): C, 61.25; H, 4.54; N, 29.41; Found: C, 61.37; H, 4.68; N, 29.67.

### 4.2 Biological assessment

#### 4.2.1 *In vitro* anti-proliferative activity across NCI's comprehensive set of 60 characterized human cancer cell lines

The cancer-preventing potential of the synthesized pyrazolopyrimidines was assessed *in vitro* using a panel of sixty cancer cell lines from the NCI, following standard protocols.^62–65^ Stock solutions of the test compounds were initially prepared in DMSO at concentrations 400-fold higher than the desired final dose and stored at subzero temperatures. Prior to use, the stock solutions were thawed, diluted in gentamicin solution (50 µg mL^−1^) to twice the required concentration, and 100 µL of the diluted solution was added to wells containing 100 µL of medium to achieve the intended final concentration.

Cells were inoculated into 96-well plates at densities ranging from 5000 to 40 000 cells per well, depending on the doubling time of each cell line. Each well was filled with 100 µL of growth medium, and the plates were incubated at 37 °C under 5% CO_2_ and 95% humidity for 24 hours to allow cell attachment. After treatment with the test compounds, the plates were incubated for an additional 48 hours under the same conditions.

For adherent cells, 50 µL of cold 50% (w/v) trichloroacetic acid (TCA) was added to each well to obtain a final concentration of 10% TCA. The plates were kept at 4 °C for 1 hour to ensure cell fixation. For suspension cells, 80% TCA was used instead of 50%, resulting in a final concentration of 16% TCA. After fixation, the plates were washed five times with tap water, air-dried, and the supernatant was removed.

Each well was subsequently treated with 100 µL of 0.4% (w/v) sulforhodamine B (SRB) in 1% acetic acid. The plates were left at room temperature for 10 minutes, excess dye was removed by washing with 1% acetic acid five times, and the plates were air-dried. The bound dye was dissolved with 10 mM Tris base, and absorbance was measured at 515 nm using an automated microplate reader.

Growth inhibition percentages were calculated using absorbance values recorded at time zero (*T*_z_), after control growth (C), and following treatment (*T*_i_). When *T*_i_ was equal to or greater than *T*_z_, the formula ((*T*_i_ − *T*_z_)/(C − *T*_z_)) × 100 was applied. When *T*i was lower than *T*_z_, the formula ((*T*_i_ − *T*_z_)/*T*_z_) × 100 was used.

#### 4.2.2 Kinases assay

##### 4.2.2.1 CDK2 inhibition assay

The suppressive impact of the targeted pyrazolopyrimidines on the CDK2/CyclinA2 enzyme was evaluated using an assay kit from BPS Bioscience, San Diego, CA, for CDK2 (Cat. #79599), following the manufacturer's protocol. Before starting the assay, ATP, peptide substrate (10×), and assay buffer (5×) were equilibrated to room temperature. A master mix was prepared by combining distilled water, ATP (10 µM), 5× Kinase Assay Buffer 1, and 10× CDK substrate peptide in volumes of 13, 6, 1, and 5 µL, respectively, per assay (25 µL total), scaled according to the number of wells. A 25 µL volume of this master mix was dispensed into all wells.

For assay setup, 5 µL of the inhibitor mixture was added to the designated test wells, whereas 5 µL of inhibitor-free buffer was added to the “Positive Control’’ and “Blank’’ wells. A working solution of 1× kinase assay buffer was prepared by diluting 600 µL of 5× kinase assay buffer 1 with 2400 µL of distilled water, yielding 3 mL, which was sufficient for one batch of 100 tests. In wells designated as “Blank’’, 20 µL of the 1× kinase assay buffer was added.

The CDK2/CyclinA2 enzyme was kept on ice and, after thawing, was briefly centrifuged to collect the contents. It was diluted in 1× kinase assay buffer 1 to approximately 5 ng µL^−1^, as recommended for the assay. To minimize freeze–thaw cycles, enzyme aliquots were stored in concentrated form at −80 °C, and previously thawed or diluted samples were not reused.

Reactions in both positive control wells and test compound wells were initiated by adding 20 µL of the enzyme solution, followed by 45 minutes of incubation at 30 °C. After incubation, 50 µL of Kinase-Glo Max reagent was added to all wells after thawing. The plate was wrapped with foil and incubated for 15 minutes at room temperature. Luminescence was recorded using a Tecan Spark Microplate Reader, and blank values were subtracted from all readings.

The experiment evaluated samples at concentrations of 0.01, 0.1, 1, and 10 µM, with standards, blanks, and test samples assayed in triplicate. Ribociclib was used as the standard reference drug and positive control.^62^

##### 4.2.2.2 TRKA inhibition assay

The inhibitory activity of the tested pyrazolopyrimidines against the TRKA enzyme was evaluated using a TRKA kinase assay kit from BPS Bioscience, San Diego, CA (Cat. #79548), following the manufacturer's protocol. ATP (20 µM), 5× Kinase Assay Buffer 1, and the PTK Substrate Poly(Glu : Tyr 4 : 1) (10 mg mL^−1^) were thawed prior to use. Dithiothreitol (DTT) was added to 5× Kinase Assay Buffer 1 to prepare the working assay buffer, yielding a final concentration of 10 µM.

To prepare the blank wells, 20 µL of 1× Kinase Assay Buffer 1 (prepared by diluting 600 µL of 5× buffer with 2400 µL of distilled water to produce 3 mL, sufficient for 100 reactions) was added.

A master mix was prepared to dispense 25 µL per well by combining distilled water, 500 µM ATP, the protein tyrosine kinase substrate Poly(Glu : Tyr 4 : 1) (10 mg mL^−1^), and 5× Kinase Assay Buffer 1 in volumes of 17, 1, 1, and 6 µL, respectively, per reaction. A 25 µL aliquot of this master solution was dispensed into all assay wells.

In the positive control and blank wells, 5 µL of inhibitor-free buffer was added, whereas 5 µL of inhibitor solution was pipetted into the designated test wells. To maintain a final reaction DMSO concentration below 1%, the inhibitor solutions were prepared so that DMSO did not exceed 10%.

The TRKA enzyme was kept on ice and, after thawing, briefly centrifuged to collect the contents and diluted in 1× Kinase Assay Buffer 1 to a final concentration of 5 ng µL^−1^. Because the enzyme was sensitive to freeze–thaw cycles, aliquots were stored at −80 °C, and previously thawed or diluted samples were not reused.

Reactions in both test and positive control wells were initiated by the addition of 20 µL of diluted enzyme solution, followed by incubation at 30 °C for 45 minutes. After incubation, 50 µL of thawed Kinase-Glo Max reagent was added to each well. The plate was wrapped in foil and incubated at room temperature for 15 minutes. Luminescence was recorded using a Tecan Spark Microplate Reader, and blank readings were subtracted from all measurements.

Samples were tested at concentrations of 0.01, 0.1, 1, and 10 µM, and all samples, standards, and blanks were analyzed in triplicate. Larotrectinib sulfate was used as the standard reference drug and positive control (Catalog No. S7960, Houston, TX, USA).^63,64^

#### 4.2.3 *In vitro* cytotoxicity screening against the SNB-75 CNS cancer cell line, K562 CML cancer cell line and the PCS-800-017 normal human fibroblast cell line

The MTT cell viability assay was performed as follows. SNB-75, K562, and PCS-800-017 cells were seeded in flat-bottom 96-well plates at a density of 5 × 10^3^ cells per well in 100 µL of complete medium and were allowed to adhere for 18–24 h. Test compounds, prepared as 10 mM stocks in DMSO, were serially diluted in medium to generate a 10-point half-log concentration series. The final DMSO concentration in all wells did not exceed 0.5% (v/v).

After 48 h of treatment with the compounds, 10 µL of MTT solution (5 mg mL^−1^ in PBS) was added to each well and incubated for 3–4 h at 37 °C. The resulting formazan crystals were solubilized using 100 µL of DMSO, and absorbance was measured at 570 nm with a reference wavelength of 630 nm. Wells containing medium only served as blanks for background subtraction. Percent cell viability was calculated using the following equation:% Viability = (Abs_treated − Abs_blank)/(Abs_vehicle − Abs_blank) × 100

The half-maximal inhibitory concentration (IC_50_) values were determined by nonlinear regression analysis of the dose–response curves using a four-parameter logistic model in GraphPad Prism software (or equivalent). Data were presented as mean IC_50_ ± SEM from three independent experiments, each performed in technical triplicate.^65^

#### 4.2.4 Comprehensive analysis of the mechanisms controlling the cell cycle progression

SNB-75 cells were collected following 48 hours of treatment with compounds 6i and 6j at their GI_50_ values and were resuspended in 0.5 mL of PBS. The cell suspension was centrifuged, and the resulting pellets were fixed at 4 °C with 70% (v/v) ethanol and washed with PBS. The cells were treated with RNase and subsequently stained with propidium iodide (PI). Flow cytometry was performed using a FACScalibur system (Becton Dickinson, Franklin Lakes, NJ, USA), and cell cycle profiles were analyzed using software from Verity Software House and Phoenix Flow System.^66^

#### 4.2.5 Examination of the cellular pathways involved in apoptosis and necrosis

SNB-75 cells were harvested following a 48-hours exposure to compounds 6i and 6j at their GI_50_ values, and centrifuged. The pellets were washed with PBS and resuspended in binding buffer without fixation. The cells were then stained with PE Annexin V and propidium iodide (PI) according to the manufacturer's protocol, ensuring membrane integrity. Flow cytometric analysis was performed using a FACScalibur system (Becton Dickinson, Franklin Lakes, NJ, USA), and the apoptotic/necrotic populations were quantified using analytical software from Verity Software House and Phoenix Flow Systems.^67^

## Conflicts of interest

The authors declare that they have no known competing financial interests or personal relationships that could have appeared to influence the work reported in this paper.

## Supplementary Material

RA-016-D6RA02521H-s001

RA-016-D6RA02521H-s002

## Data Availability

All relevant data are provided within the article and its supplementary information (SI). Supplementary information is available. See DOI: https://doi.org/10.1039/d6ra02521h.
