# Supplementary material for: Dual inhibitors of CDK2 and TRKA kinases: design, synthesis, antiproliferative activity and molecular modeling of novel pyrazolopyrimidine derivatives
Source: RSC Adv. 2026 Sep 16;16(44):55449–69. doi: 10.1039/d6ra02521h (PMC13579623; doi:10.1039/d6ra02521h)
Supplement: Supplementary file 1 [file RA-016-D6RA02521H-s001.pdf]

Supplementary Information (SI) for RSC Advances.
This journal is © The Royal Society of Chemistry 2026

Chemistry

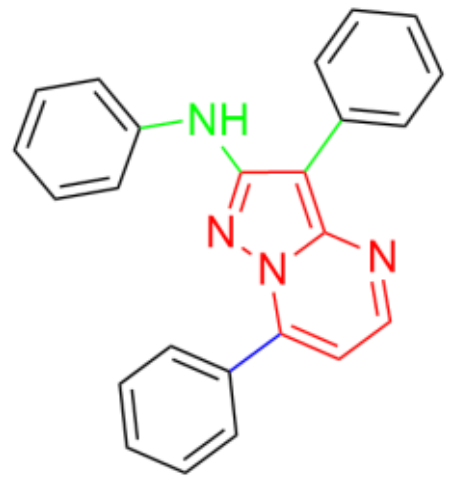

6a

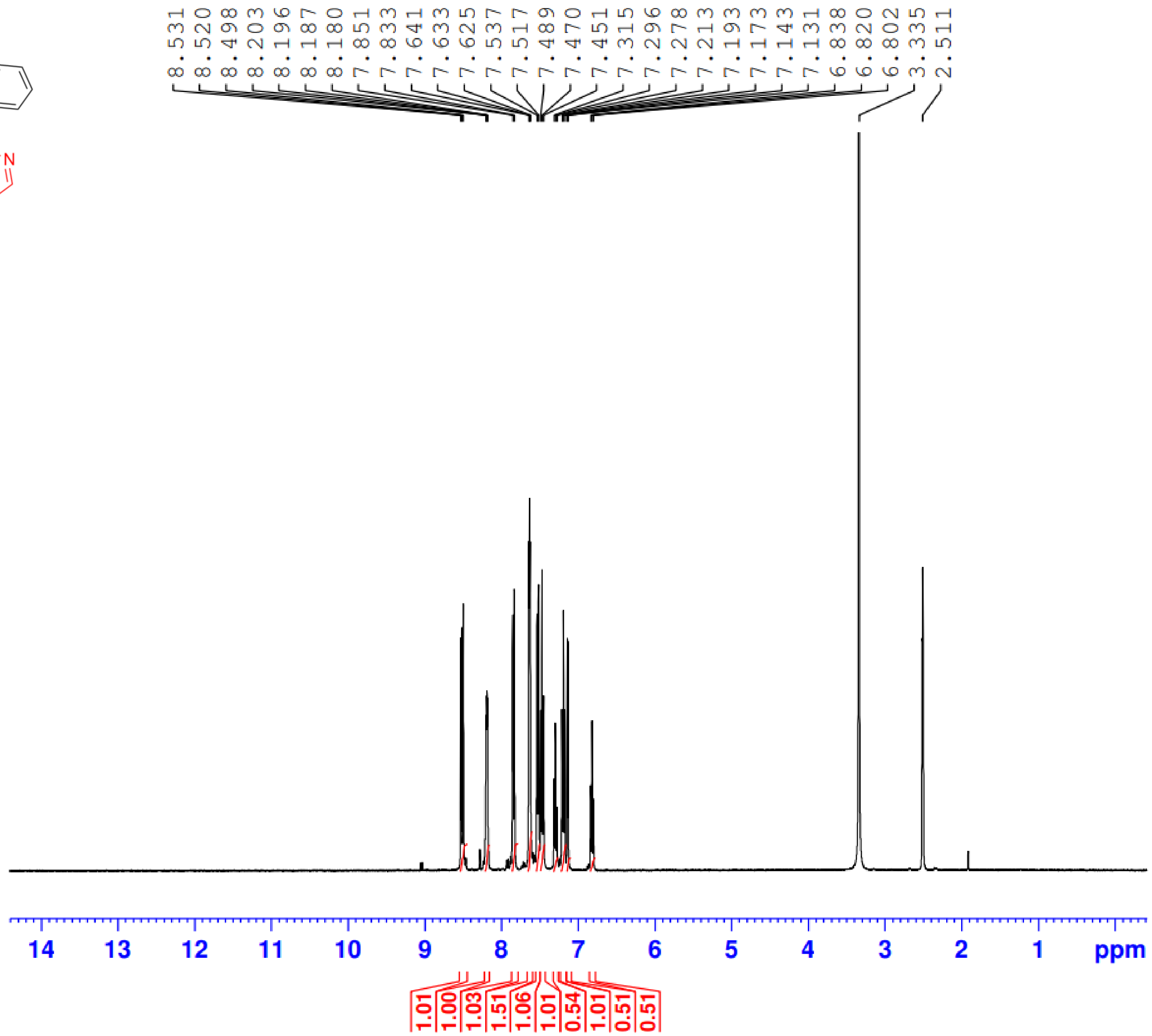

BRUKER

```
Current Data Parameters
NAME        mohammed-attia-13a
EXPNO                        1
PROCNO                       1

F2 - Acquisition Parameters
Date_                 20210520
Time                     14.20
INSTRUM                  spect
PROBHD        5 mm PABBO BB/
PULPROG                   zg30
TD                       65536
SOLVENT                   DMSO
NS                          22
DS                           2
SWH                   8012.820 Hz
FIDRES                0.122266 Hz
AQ                   4.0894465 sec
RG                      205.37
DW                      62.400 usec
DE                        6.50 usec
TE                       300.0 K
D1                  1.00000000 sec
TD0                          1

======== CHANNEL f1 ========
SFO1               400.1524711 MHz
NUC1                        1H
P1                       12.00 usec
PLW1               18.00000000 W

F2 - Processing parameters
SI                       65536
SF                 400.1500000 MHz
WDW                         EM
SSB         0
LB                        0.30 Hz
GB          0
PC                        1.00
```

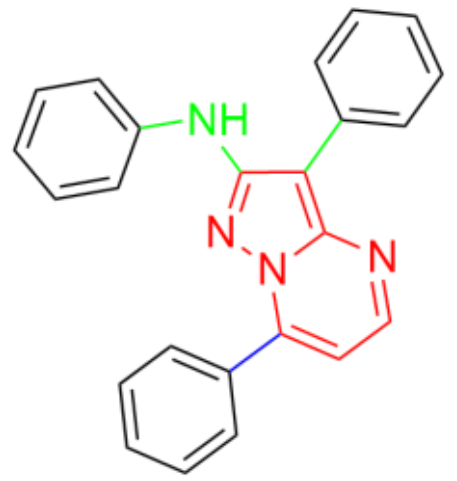

6a

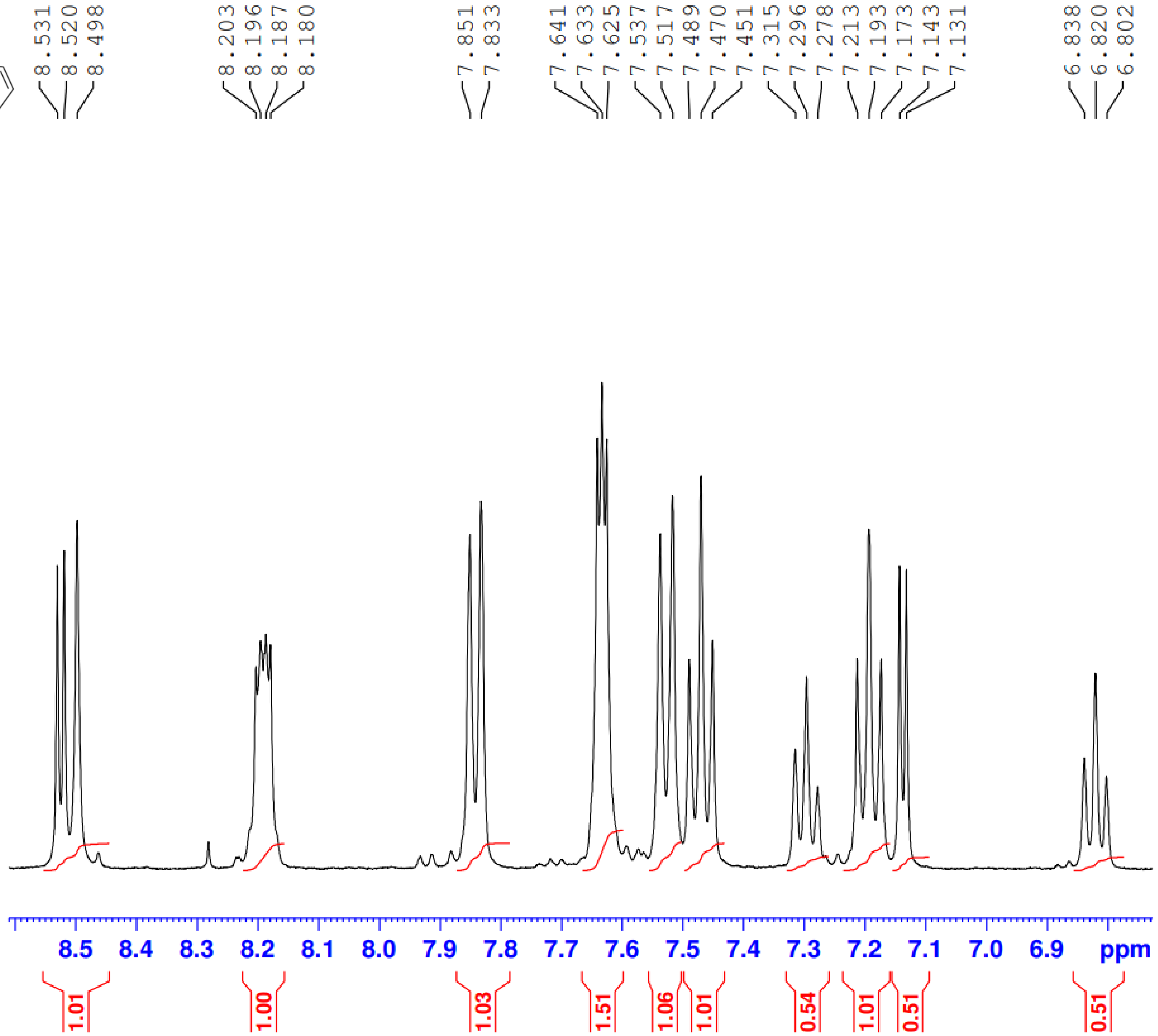

BRUKER

Current Data Parameters

NAME	mohammed-attia-13a
EXPNO	1
PROCNO	1

F2 - Acquisition Parameters

Date_	20210520
Time	14.20
INSTRUM	spect
PROBHD	5 mm PABBO BB/
PULPROG	zg30
TD	65536
SOLVENT	DMSO
NS	22
DS	2
SWH	8012.820 Hz
FIDRES	0.122266 Hz
AQ	4.0894465 sec
RG	205.37
DW	62.400 usec
DE	6.50 usec
TE	300.0 K
D1	1.00000000 sec
TD0	1

======== CHANNEL f1 ========

SFO1	400.1524711 MHz
NUC1	1H
P1	12.00 usec
PLW1	18.00000000 W

F2 - Processing parameters

SI	65536
SF	400.1500000 MHz
WDW	EM
SSB	0
LB	0.30 Hz
GB	0
PC	1.00

D_2O

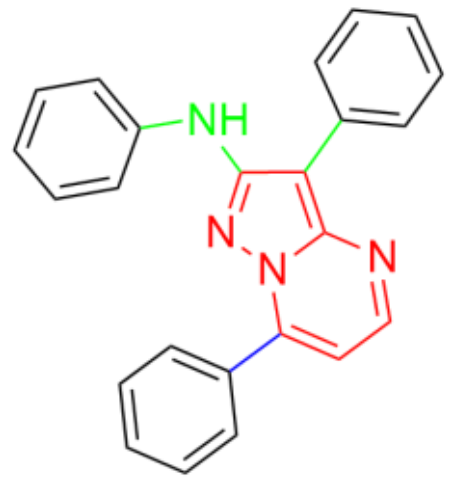

6a

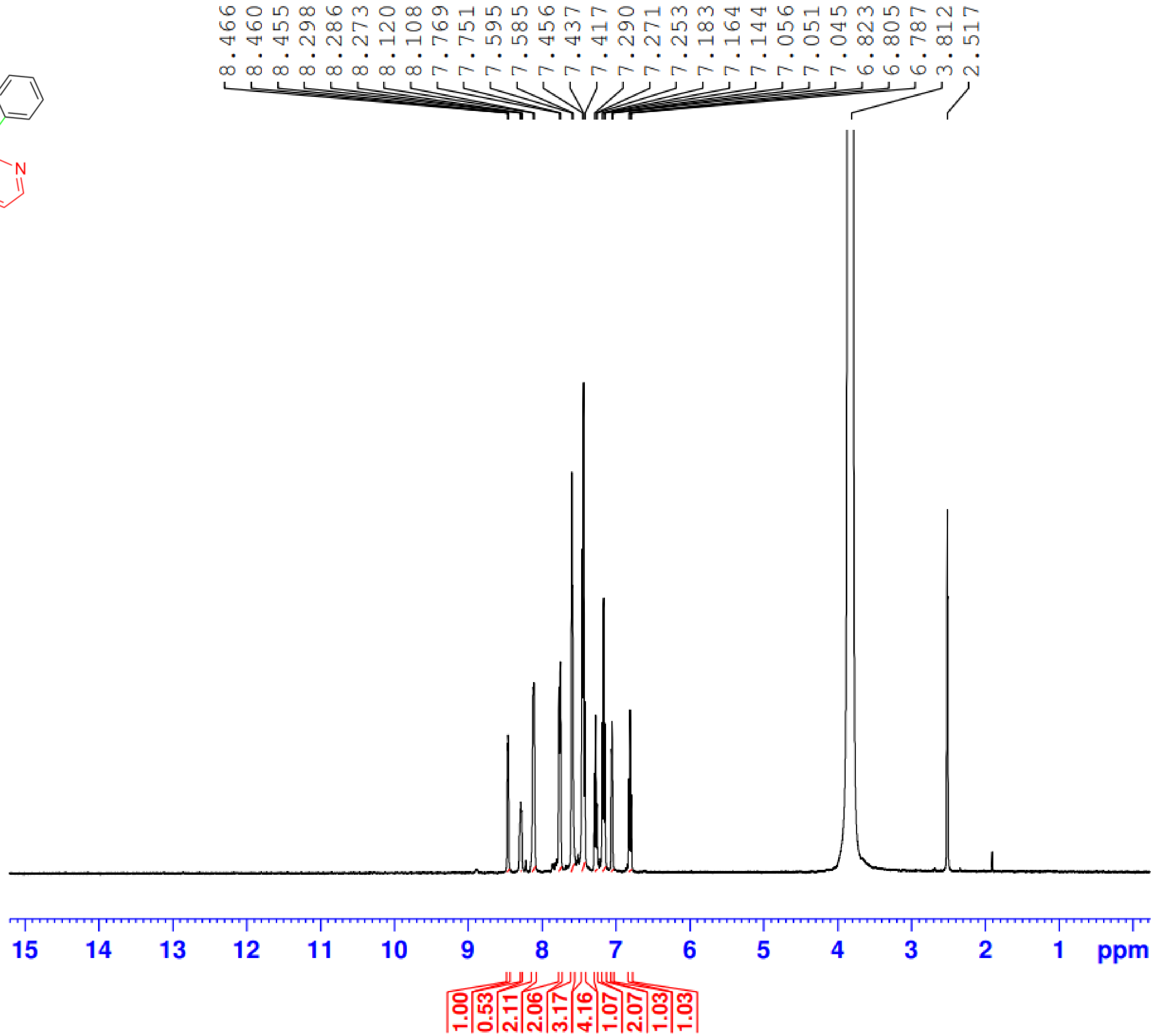


```
Current Data Parameters
NAME      mohammed-attia-13a-d2o
EXPNO                     1
PROCNO                    1

F2 - Acquisition Parameters
Date_              20210830
Time                  15.25
INSTRUM               spect
PROBHD    5 mm PABBO BB/
PULPROG                zg30
TD                    65536
SOLVENT                DMSO
NS                       16
DS                        2
SWH              8012.820 Hz
FIDRES           0.122266 Hz
AQ              4.0894465 sec
RG                   126.31
DW                   62.400 usec
DE                     6.50 usec
TE                    300.0 K
D1               1.00000000 sec
TD0                       1

======== CHANNEL f1 ========
SFO1          400.1524711 MHz
NUC1                     1H
P1                    12.00 usec
PLW1            18.00000000 W

F2 - Processing parameters
SI                    65536
SF            400.1500000 MHz
WDW                      EM
SSB          0
LB                     0.30 Hz
GB           0
PC                     1.00
```

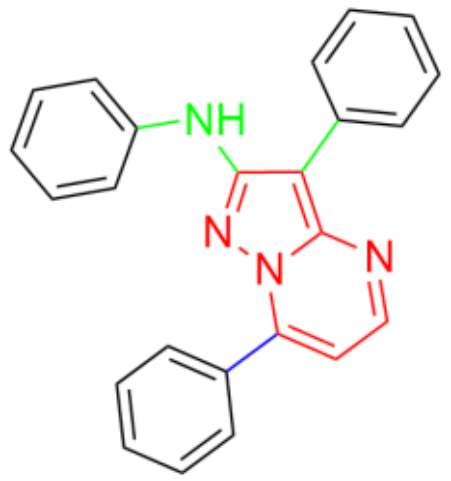

6a

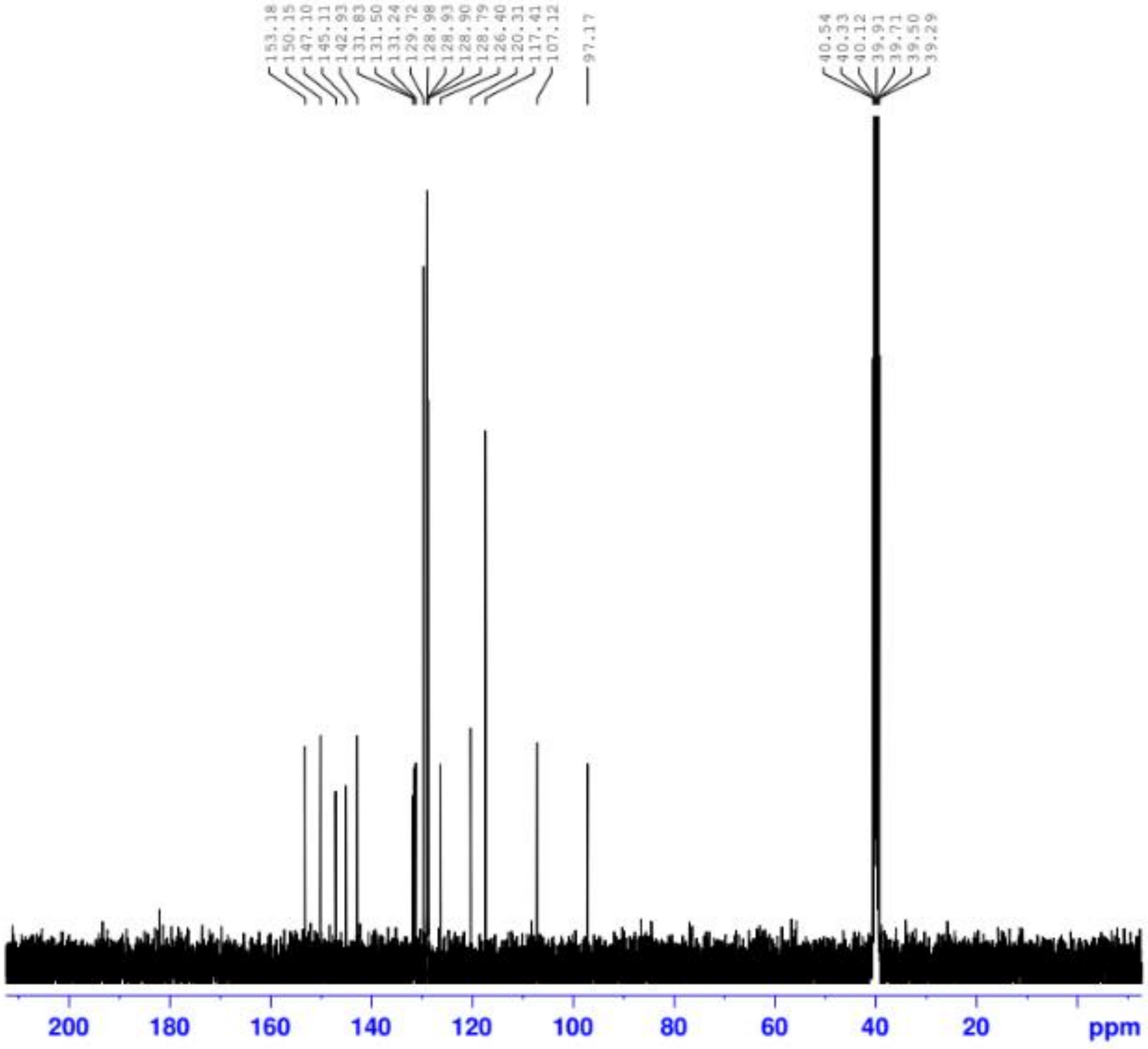

BRUKER

Current Data Parameters
NAME mohammed-attia-13a
EXPNO 2
PROCNO 1

F2 - Acquisition Parameters
Date_ 20210624
Time 16.18
INSTRUM spect
PROBHD 5 mm PABBO BB/
PULPROG zgpg30
TD 65536
SOLVENT DMSO
NS 1010
DS 4
SWH 24038.461 Hz
FIDRES 0.366798 Hz
AQ 1.3631488 sec
RG 205.37
DW 20.800 usec
DE 6.50 usec
TE 300.0 K
D1 2.00000000 sec
D11 0.03000000 sec
TD0 1

======== CHANNEL f1 ========
SFO1 100.6278588 MHz
NUC1 13C
P1 10.00 usec
PLW1 47.00000000 W

======== CHANNEL f2 ========
SFO2 400.1516006 MHz
NUC2 1H
CPDPRG[2 waltz16
PCPD2 90.00 usec
PLW2 18.00000000 W
PLW12 0.34722000 W
PLW13 0.28125000 W

F2 - Processing parameters
SI 32768
SF 100.6177975 MHz
WDW EM
SSB 0
LB 1.00 Hz
GB 0
PC 1.40

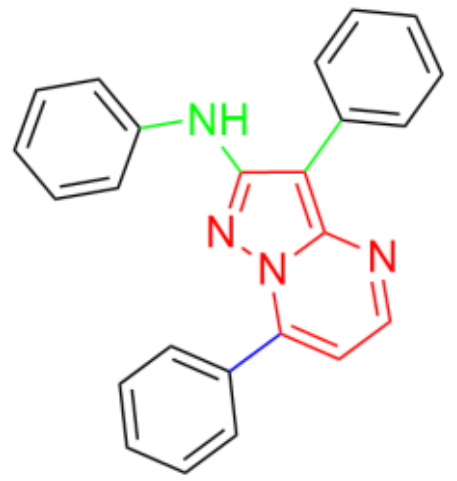

6a

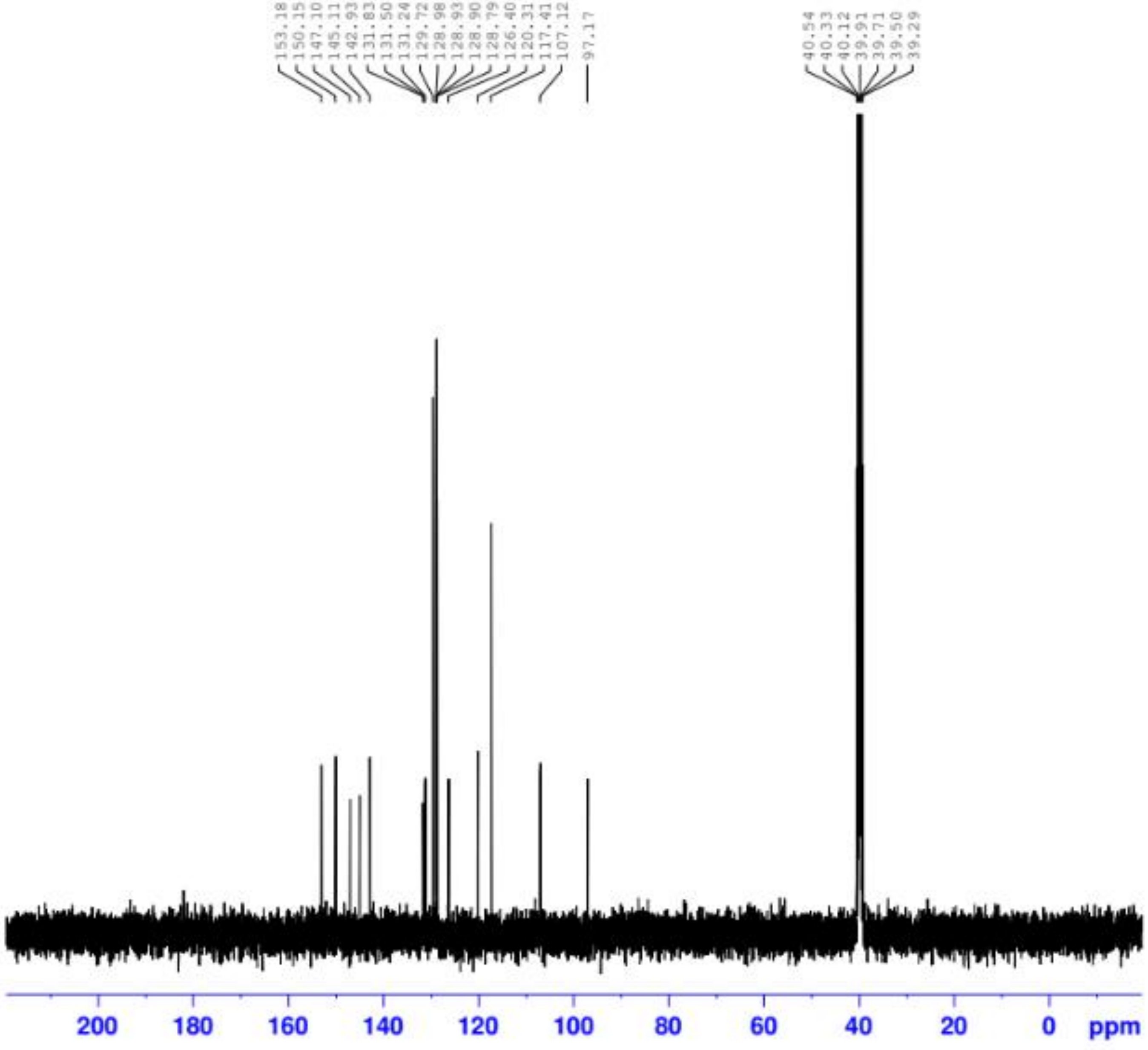

BRUKER

```
Current Data Parameters
NAME       mohammed-attia-13a
EXPNO                       2
PROCNO                      1

F2 - Acquisition Parameters
Date_                20210624
Time                    16.18
INSTRUM                 spect
PROBHD     5 mm PABBO BB/
PULPROG               zgpg30
TD                      65536
SOLVENT                  DMSO
NS                       1010
DS                          4
SWH                 24038.461 Hz
FIDRES               0.366798 Hz
AQ                  1.3631488 sec
RG                     205.37
DW                     20.800 usec
DE                       6.50 usec
TE                      300.0 K
D1                 2.00000000 sec
D11                0.03000000 sec
TD0                         1

======== CHANNEL f1 ========
SFO1              100.6278588 MHz
NUC1                      13C
P1                      10.00 usec
PLW1              47.00000000 W

======== CHANNEL f2 ========
SFO2              400.1516006 MHz
NUC2                       1H
CPDPRG[2              waltz16
PCPD2                   90.00 usec
PLW2              18.00000000 W
PLW12              0.34722000 W
PLW13              0.28125000 W

F2 - Processing parameters
SI                      32768
SF                100.6177975 MHz
WDW                        EM
SSB        0
LB                       1.00 Hz
GB         0
PC                       1.40
```

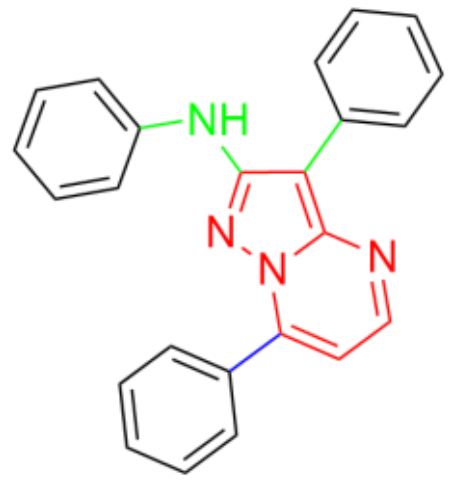

6a

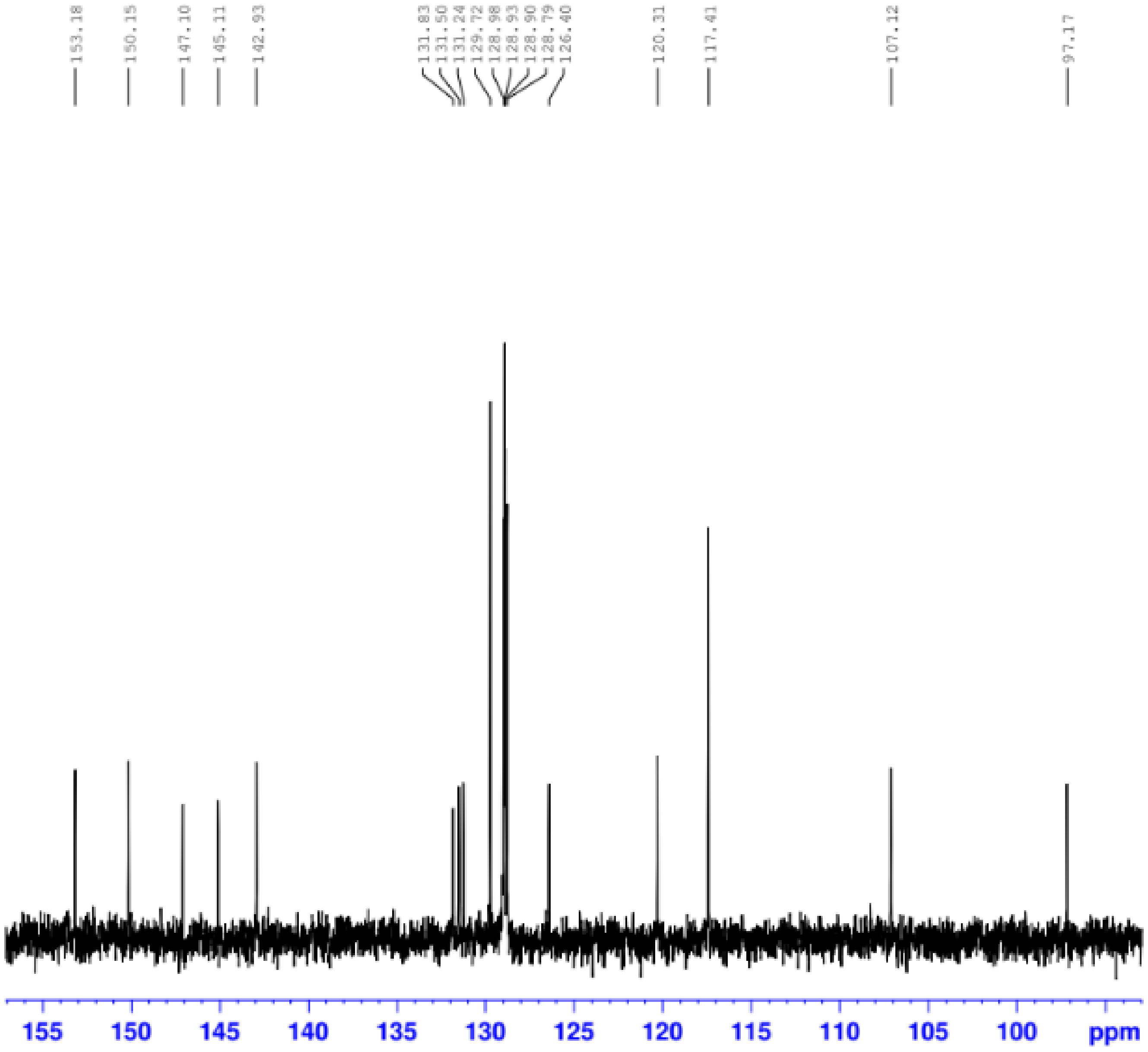

BRUKER

```
Current Data Parameters
NAME      mohammed-attia-13a
EXPNO                    2
PROCNO                   1

F2 - Acquisition Parameters
Date_             20210624
Time                 16.18
INSTRUM              spect
PROBHD    5 mm PABBO BB/
PULPROG            zgpg30
TD                  65536
SOLVENT              DMSO
NS                   1010
DS                      4
SWH             24038.461 Hz
FIDRES           0.366798 Hz
AQ              1.3631488 sec
RG                 205.37
DW                 20.800 usec
DE                   6.50 usec
TE                  300.0 K
D1             2.00000000 sec
D11            0.03000000 sec
TD0                     1

======== CHANNEL f1 ========
SFO1          100.6278588 MHz
NUC1                  13C
P1                  10.00 usec
PLW1          47.00000000 W

======== CHANNEL f2 ========
SFO2          400.1516006 MHz
NUC2                   1H
CPDPRG[2          waltz16
PCPD2               90.00 usec
PLW2          18.00000000 W
PLW12          0.34722000 W
PLW13          0.28125000 W

F2 - Processing parameters
SI                  32768
SF            100.6177975 MHz
WDW                    EM
SSB       0
LB                   1.00 Hz
GB        0
PC                   1.40
```

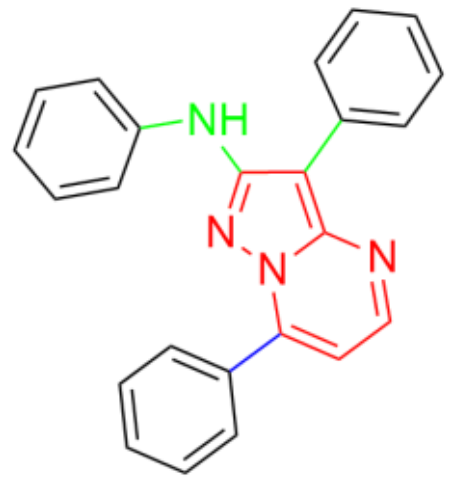
NH
N
N
N

6a

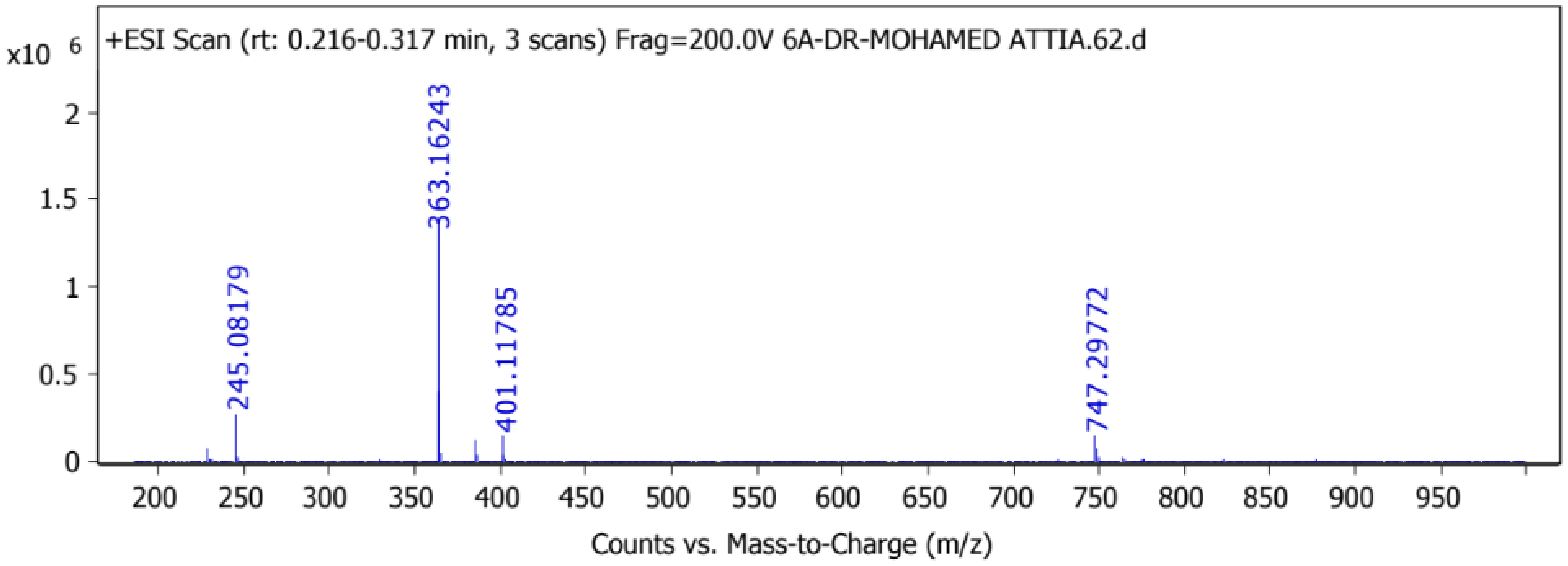
+ESI Scan (rt: 0.216-0.317 min, 3 scans) Frag=200.0V 6A-DR-MOHAMED ATTIA.62.d
x10 6
2
1.5
1
0.5
0
363.16243
245.08179
401.11785
747.29772
200
250
300
350
400
450
500
550
600
650
700
750
800
850
900
950
Counts vs. Mass-to-Charge (m/z)

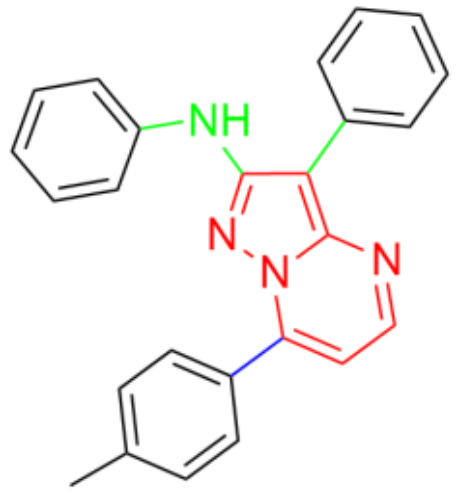

6b

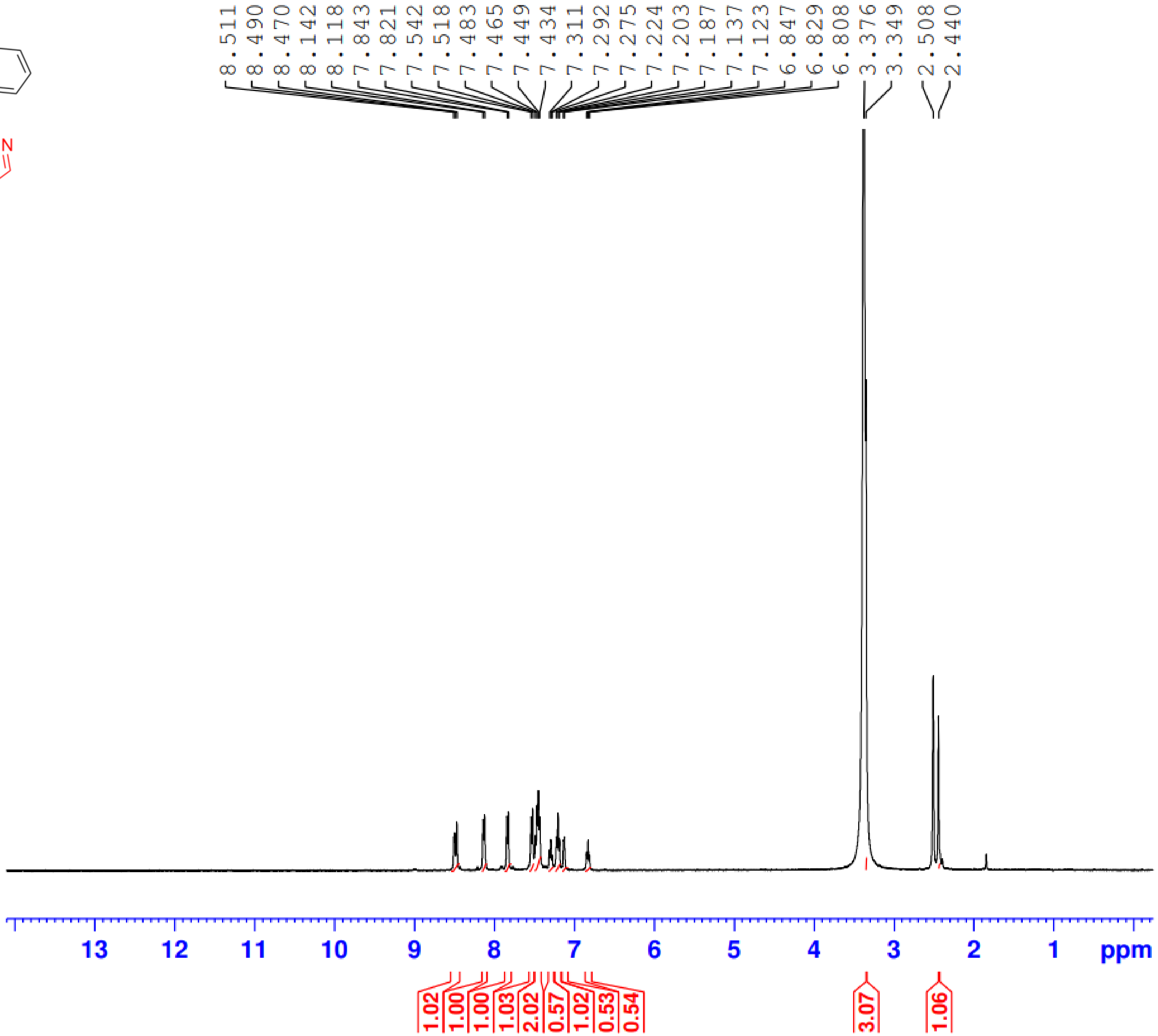

BRUKER

Current Data Parameters

NAME	mohammed-attia-13b
EXPNO	1
PROCNO	1

F2 - Acquisition Parameters

Date_	20210520
Time	14.51
INSTRUM	spect
PROBHD	5 mm PABBO BB/
PULPROG	zg30
TD	65536
SOLVENT	DMSO
NS	16
DS	2
SWH	8012.820 Hz
FIDRES	0.122266 Hz
AQ	4.0894465 sec
RG	205.37
DW	62.400 usec
DE	6.50 usec
TE	300.0 K
D1	1.00000000 sec
TD0	1

======== CHANNEL f1 ========

SFO1	400.1524711 MHz
NUC1	1H
P1	12.00 usec
PLW1	18.00000000 W

F2 - Processing parameters

SI	65536
SF	400.1500000 MHz
WDW	EM
SSB	0
LB	0.30 Hz
GB	0
PC	1.00

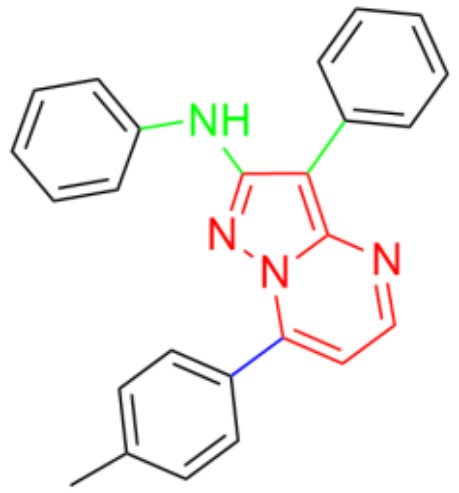

6b

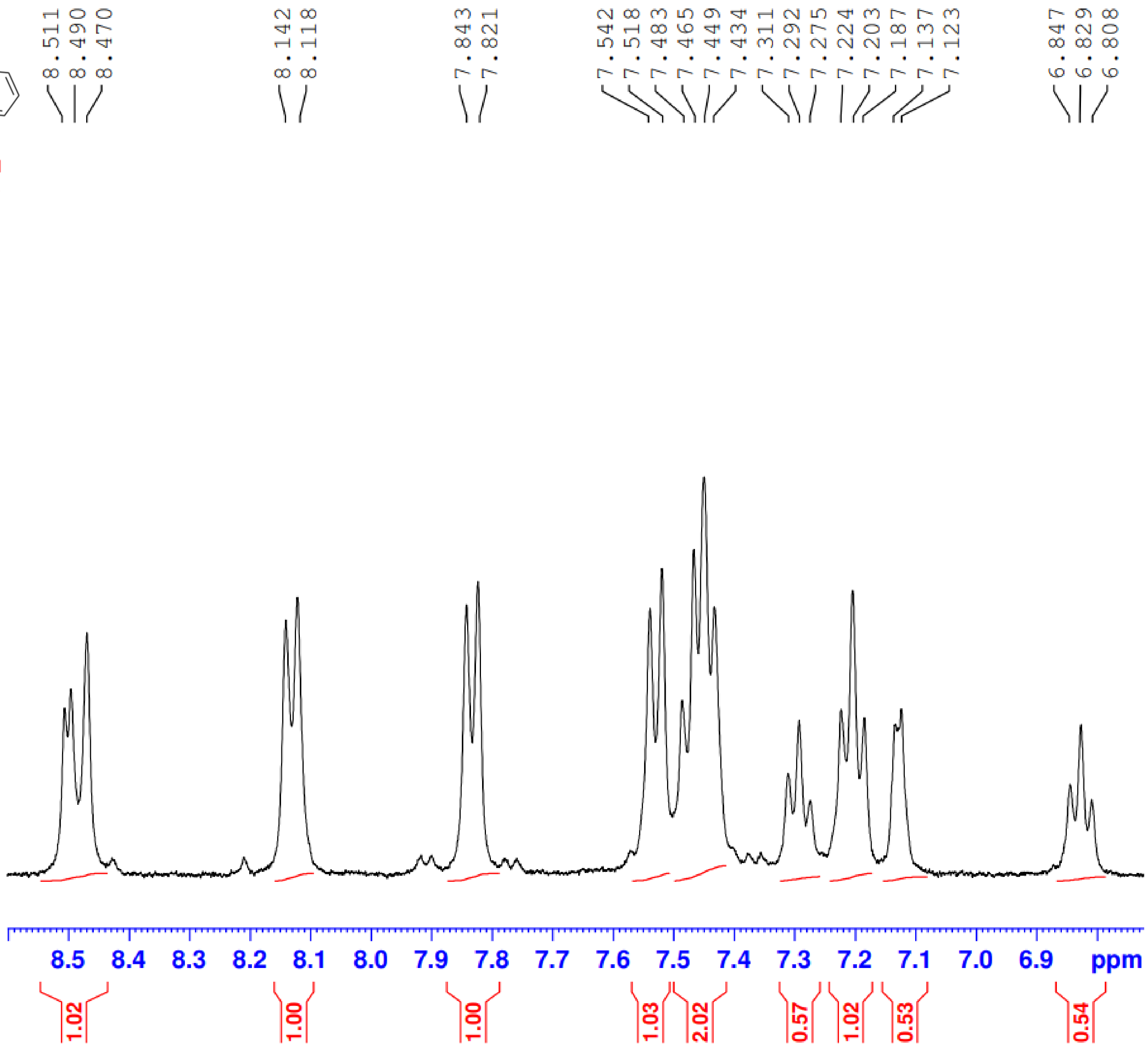


```
Current Data Parameters
NAME      mohammed-attia-13b
EXPNO                      1
PROCNO                     1

F2 - Acquisition Parameters
Date_               20210520
Time                   14.51
INSTRUM                spect
PROBHD    5 mm PABBO BB/
PULPROG                 zg30
TD                     65536
SOLVENT                 DMSO
NS                        16
DS                         2
SWH                 8012.820 Hz
FIDRES              0.122266 Hz
AQ                 4.0894465 sec
RG                    205.37
DW                    62.400 usec
DE                      6.50 usec
TE                     300.0 K
D1                1.00000000 sec
TD0                        1

======== CHANNEL f1 ========
SFO1             400.1524711 MHz
NUC1                      1H
P1                     12.00 usec
PLW1             18.00000000 W

F2 - Processing parameters
SI                     65536
SF               400.1500000 MHz
WDW                       EM
SSB      0
LB                      0.30 Hz
GB       0
PC                      1.00
```

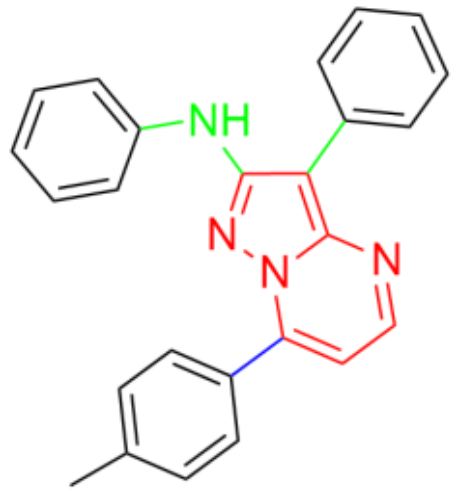

6b

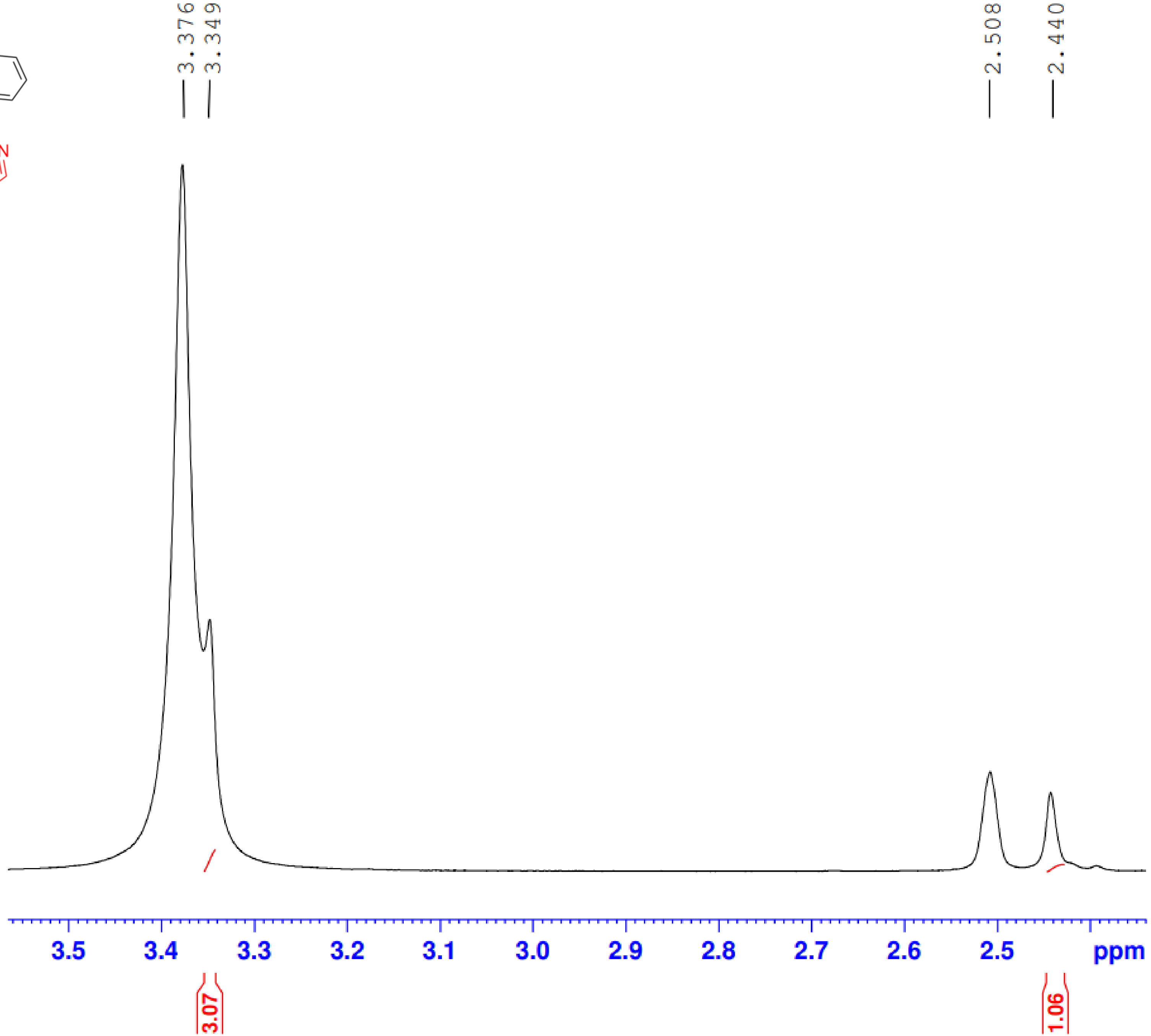

BRUKER

```
Current Data Parameters
NAME       mohammed-attia-13b
EXPNO                       1
PROCNO                      1

F2 - Acquisition Parameters
Date_              20210520
Time                  14.51
INSTRUM               spect
PROBHD     5 mm PABBO BB/
PULPROG                zg30
TD                    65536
SOLVENT                DMSO
NS                       16
DS                        2
SWH                8012.820 Hz
FIDRES             0.122266 Hz
AQ                4.0894465 sec
RG                   205.37
DW                   62.400 usec
DE                     6.50 usec
TE                    300.0 K
D1               1.00000000 sec
TD0                       1

======== CHANNEL f1 ========
SFO1            400.1524711 MHz
NUC1                     1H
P1                    12.00 usec
PLW1            18.00000000 W

F2 - Processing parameters
SI                    65536
SF              400.1500000 MHz
WDW                      EM
SSB        0
LB                     0.30 Hz
GB         0
PC                     1.00
```

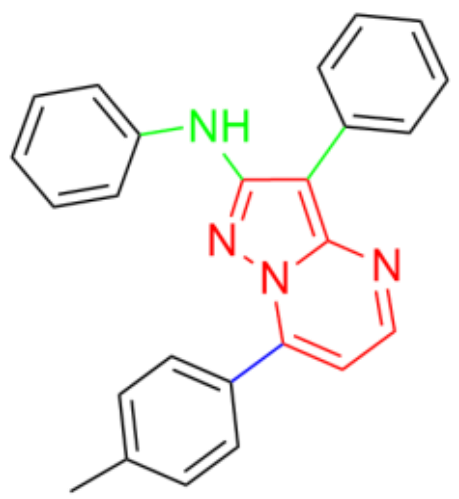
NH
N
N
N

6b

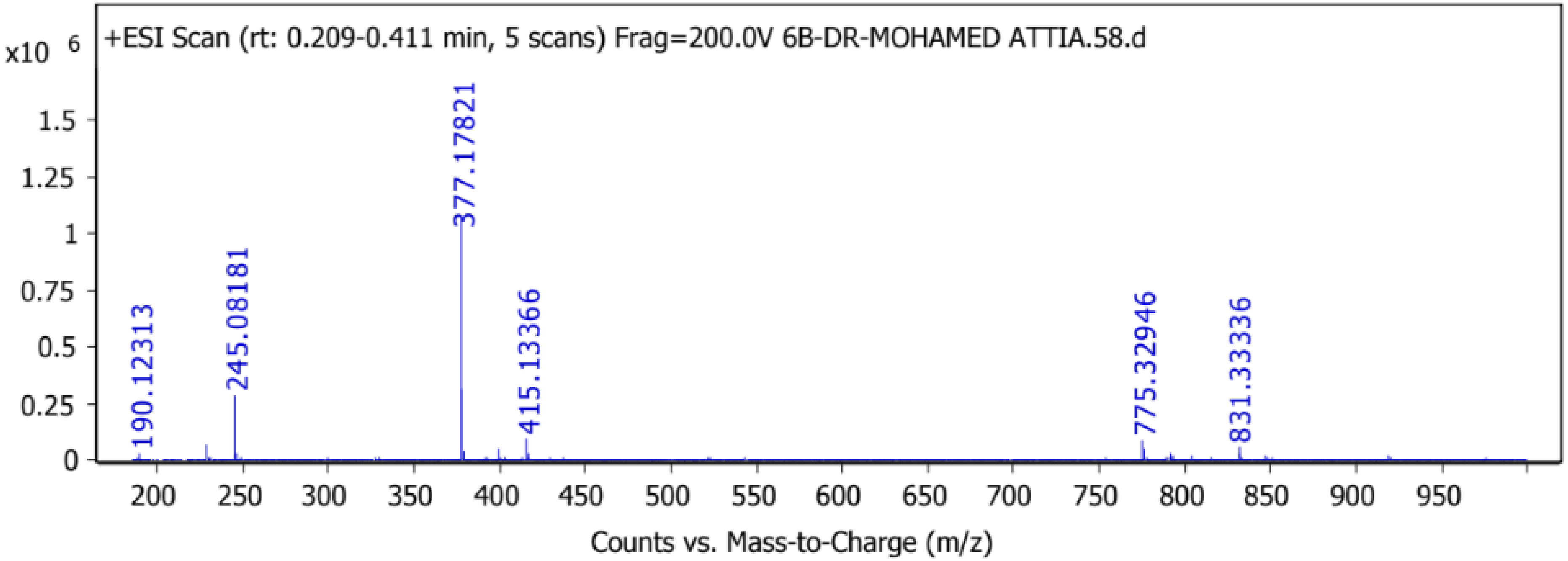
+ESI Scan (rt: 0.209-0.411 min, 5 scans) Frag=200.0V 6B-DR-MOHAMED ATTIA.58.d
x10 6
1.5
1.25
1
0.75
0.5
0.25
0
190.12313
245.08181
377.17821
415.13366
775.32946
831.33336
200
250
300
350
400
450
500
550
600
650
700
750
800
850
900
950
Counts vs. Mass-to-Charge (m/z)

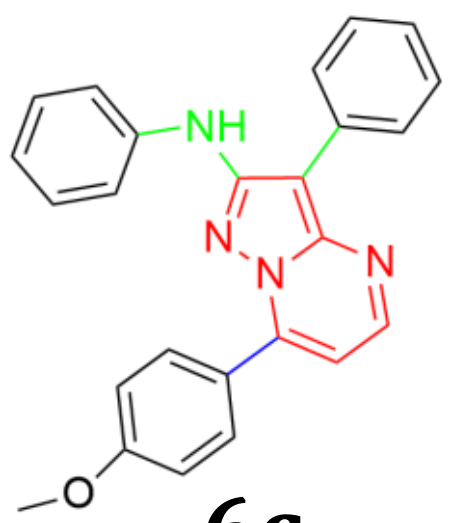

6c

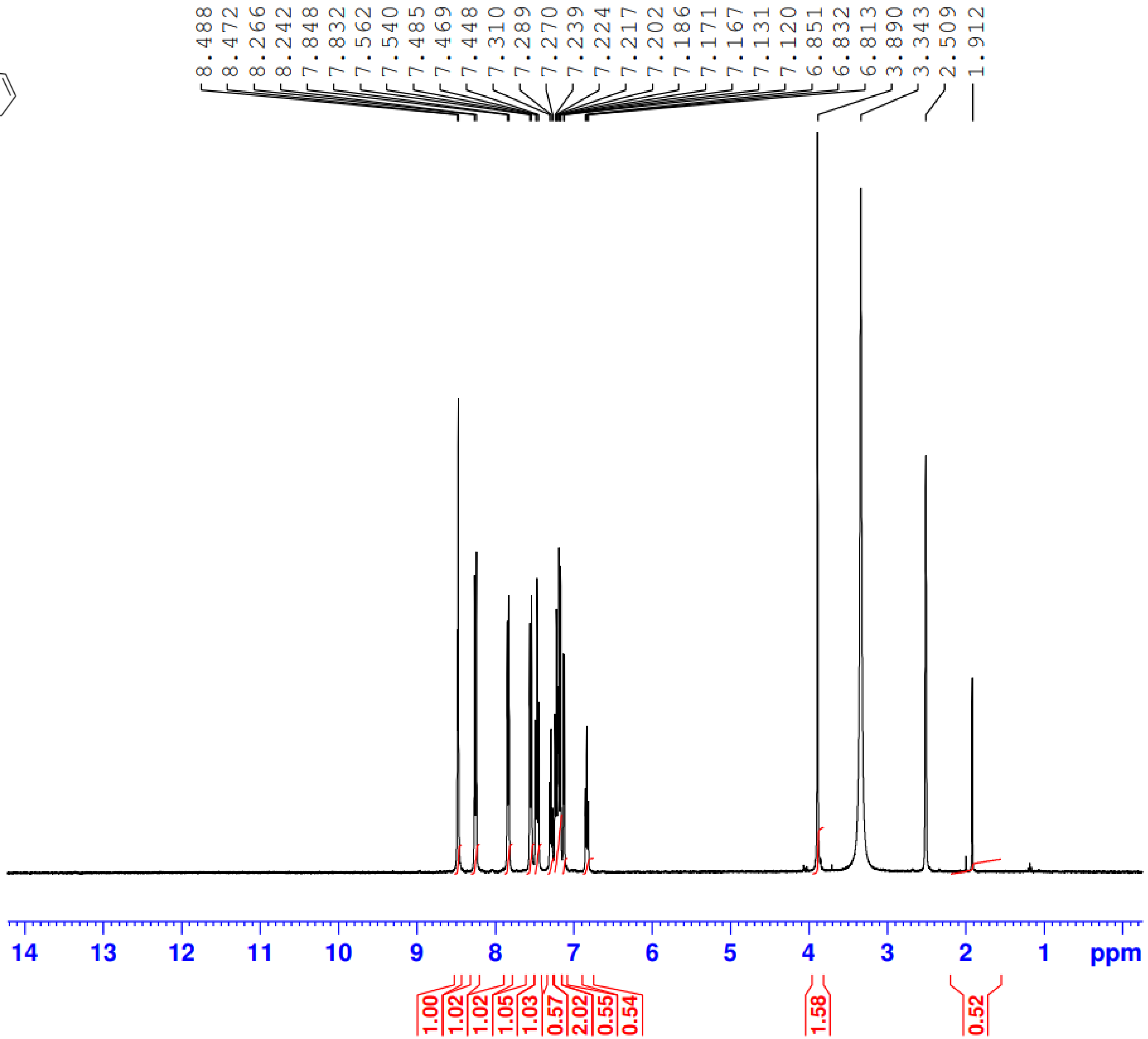

BRUKER

```
Current Data Parameters
NAME        mohammed-attia-13c
EXPNO                        1
PROCNO                       1

F2 - Acquisition Parameters
Date_                 20210520
Time                     13.56
INSTRUM                  spect
PROBHD    5 mm PABBO BB/
PULPROG                   zg30
TD                       65536
SOLVENT                   DMSO
NS                          16
DS                           2
SWH                   8012.820 Hz
FIDRES                0.122266 Hz
AQ                   4.0894465 sec
RG                      205.37
DW                      62.400 usec
DE                        6.50 usec
TE                       300.0 K
D1                  1.00000000 sec
TD0                          1

======== CHANNEL f1 ========
SFO1               400.1524711 MHz
NUC1                        1H
P1                       12.00 usec
PLW1               18.00000000 W

F2 - Processing parameters
SI                       65536
SF                 400.1500000 MHz
WDW                         EM
SSB       0
LB                        0.30 Hz
GB        0
PC                        1.00
```

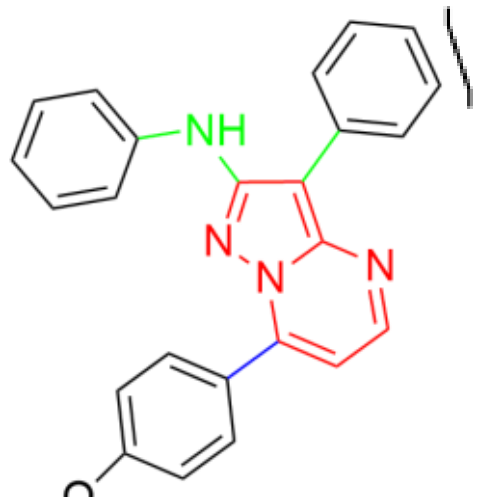

6c

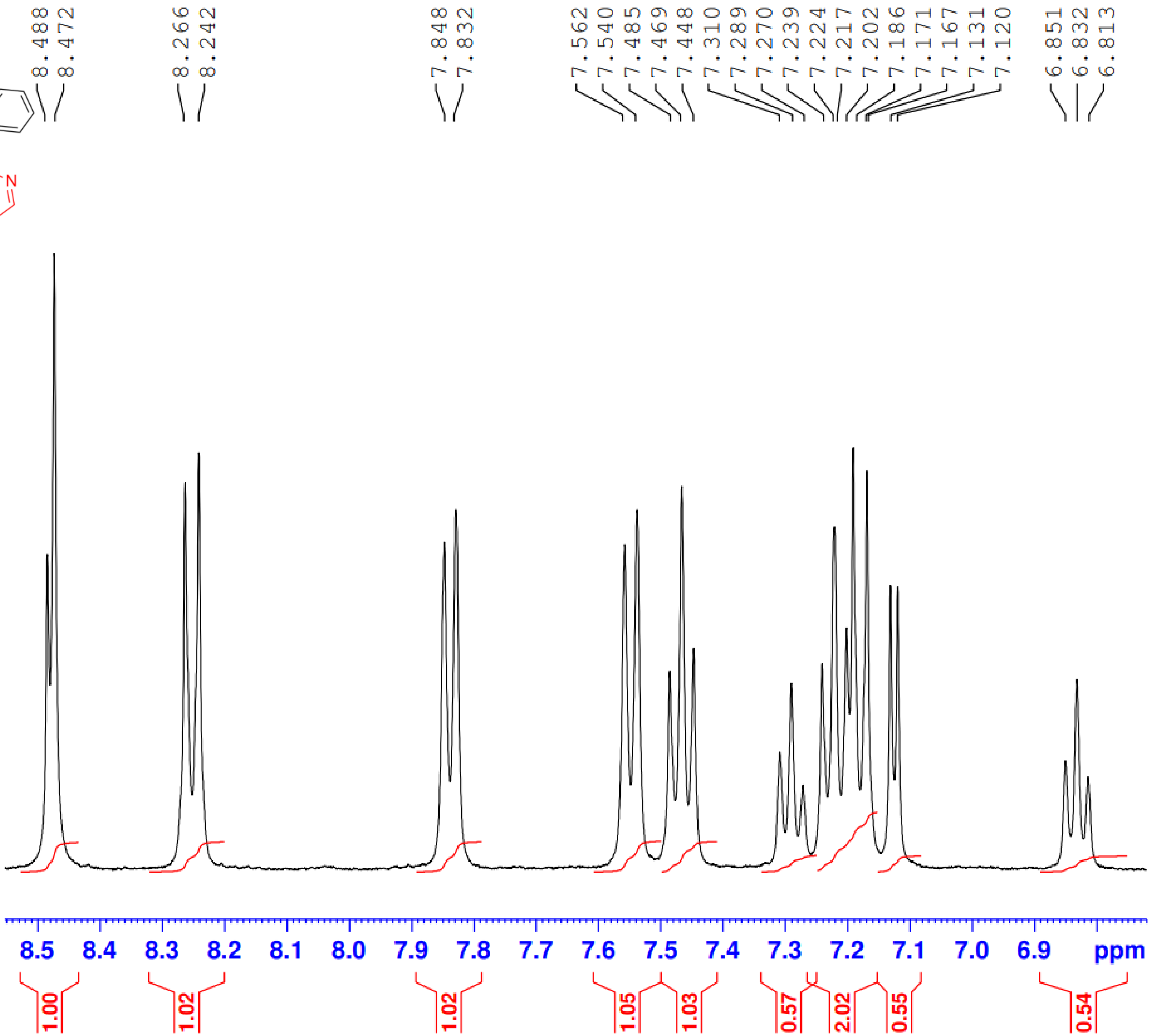


```
Current Data Parameters
NAME       mohammed-attia-13c
EXPNO                       1
PROCNO                      1

F2 - Acquisition Parameters
Date_              20210520
Time                  13.56
INSTRUM               spect
PROBHD     5 mm PABBO BB/
PULPROG               zg30
TD                    65536
SOLVENT                DMSO
NS                       16
DS                        2
SWH                8012.820 Hz
FIDRES             0.122266 Hz
AQ                4.0894465 sec
RG                   205.37
DW                   62.400 usec
DE                     6.50 usec
TE                    300.0 K
D1               1.00000000 sec
TD0                       1

======== CHANNEL f1 ========
SFO1            400.1524711 MHz
NUC1                     1H
P1                    12.00 usec
PLW1            18.00000000 W

F2 - Processing parameters
SI                    65536
SF              400.1500000 MHz
WDW                      EM
SSB       0
LB                     0.30 Hz
GB        0
PC                     1.00
```

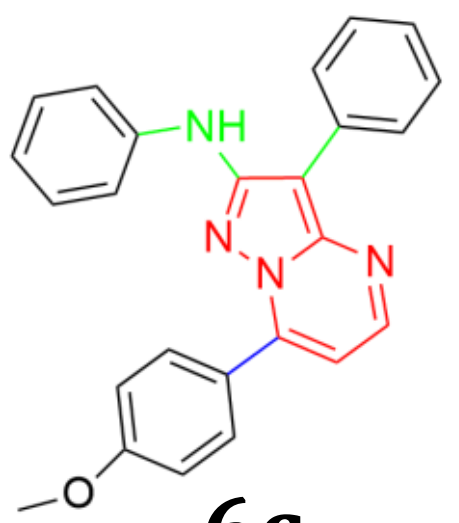

6c

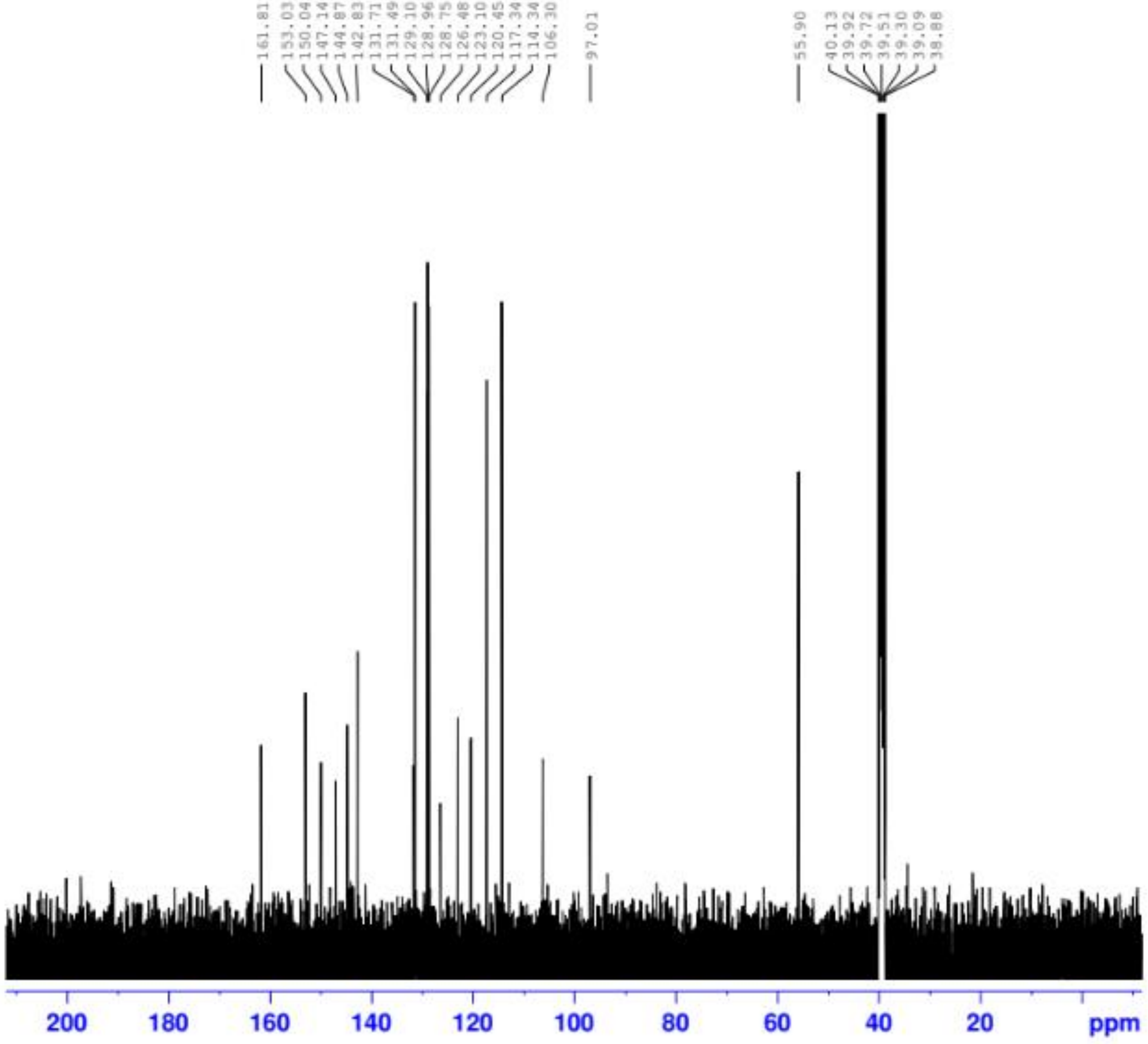

BRUKER

Current Data Parameters
NAME mohammed-attia-13c
EXPNO 2
PROCNO 1

F2 - Acquisition Parameters
Date_ 20210704
Time 9.25
INSTRUM spect
PROBHD 5 mm PABBO BB/
PULPROG zgpg30
TD 65536
SOLVENT DMSO
NS 1024
DS 4
SWH 24038.461 Hz
FIDRES 0.366798 Hz
AQ 1.3631488 sec
RG 205.37
DW 20.800 usec
DE 6.50 usec
TE 300.0 K
D1 2.00000000 sec
D11 0.03000000 sec
TD0 1

======== CHANNEL f1 ========
SFO1 100.6278588 MHz
NUC1 13C
P1 10.00 usec
PLW1 47.00000000 W

======== CHANNEL f2 ========
SFO2 400.1516006 MHz
NUC2 1H
CPDPRG[2 waltz16
PCPD2 90.00 usec
PLW2 18.00000000 W
PLW12 0.34722000 W
PLW13 0.28125000 W

F2 - Processing parameters
SI 32768
SF 100.6177975 MHz
WDW EM
SSB 0
LB 1.00 Hz
GB 0
PC 1.40

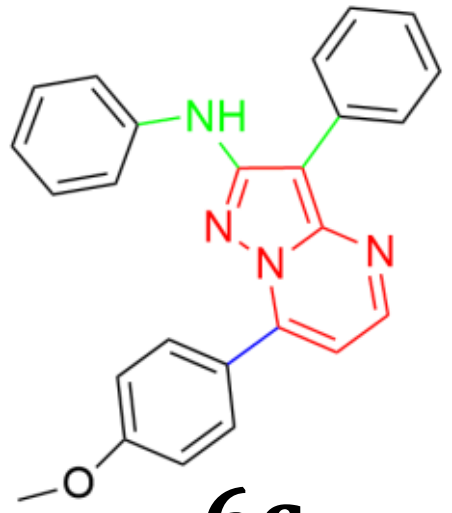

6c

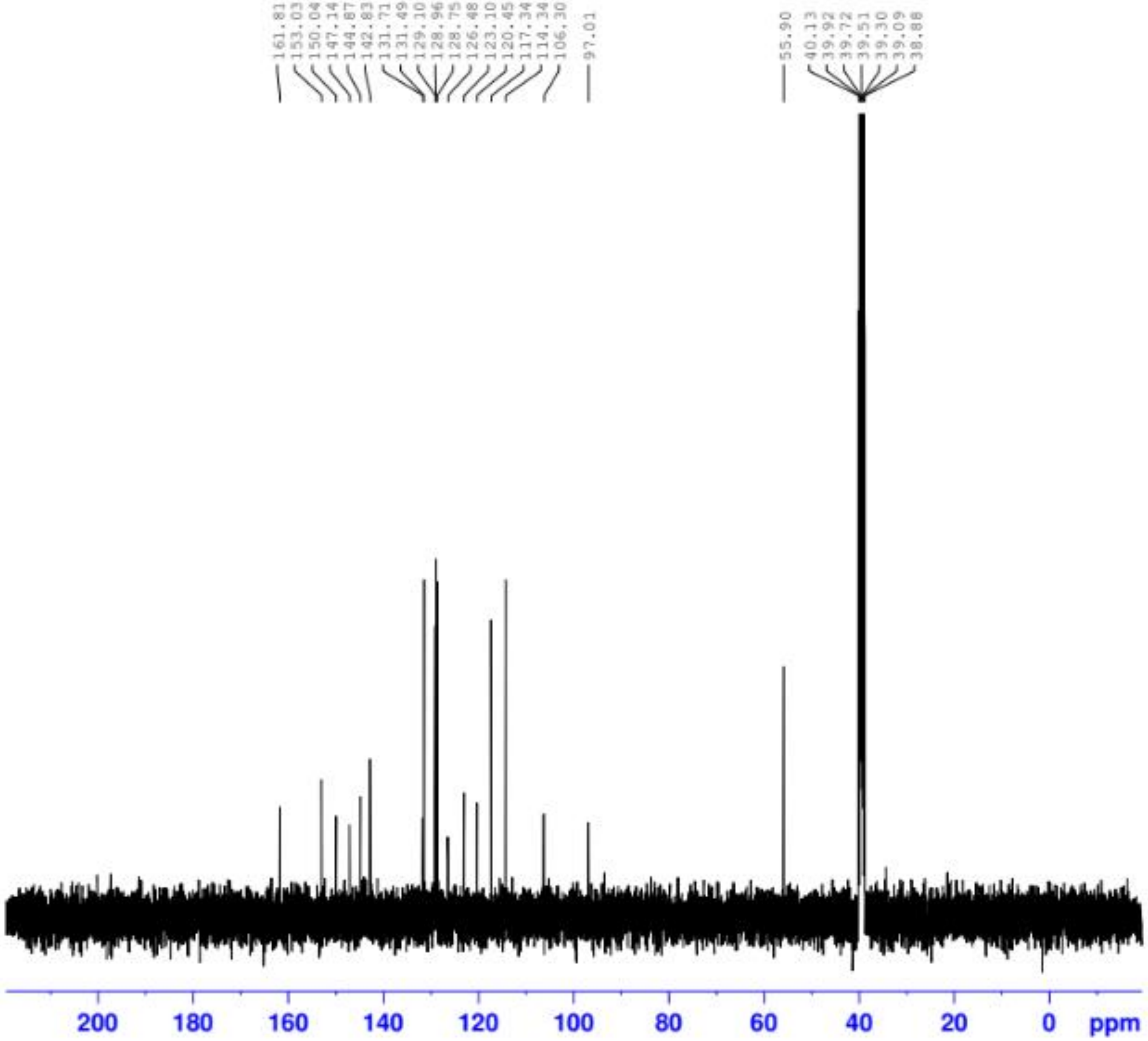

Current Data Parameters
NAME mohammed-attia-13c
EXPNO 2
PROCNO 1

F2 - Acquisition Parameters
Date_ 20210704
Time 9.25
INSTRUM spect
PROBHD 5 mm PABBO BB/
PULPROG zgpg30
TD 65536
SOLVENT DMSO
NS 1024
DS 4
SWH 24038.461 Hz
FIDRES 0.366798 Hz
AQ 1.3631488 sec
RG 205.37
DW 20.800 usec
DE 6.50 usec
TE 300.0 K
D1 2.00000000 sec
D11 0.03000000 sec
TD0 1

======== CHANNEL f1 ========
SFO1 100.6278588 MHz
NUC1 13C
P1 10.00 usec
PLW1 47.00000000 W

======== CHANNEL f2 ========
SFO2 400.1516006 MHz
NUC2 1H
CPDPRG[2 waltz16
PCPD2 90.00 usec
PLW2 18.00000000 W
PLW12 0.34722000 W
PLW13 0.28125000 W

F2 - Processing parameters
SI 32768
SF 100.6177975 MHz
WDW EM
SSB 0
LB 1.00 Hz
GB 0
PC 1.40

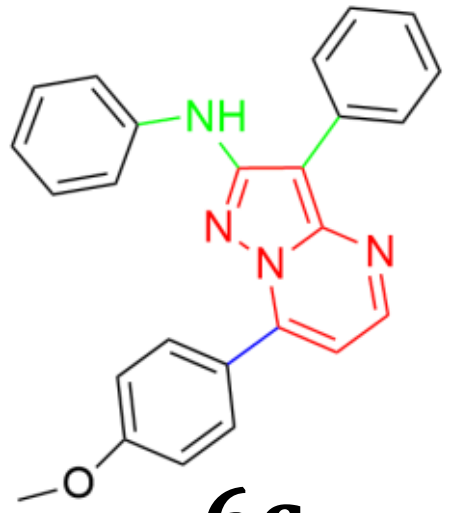

6c

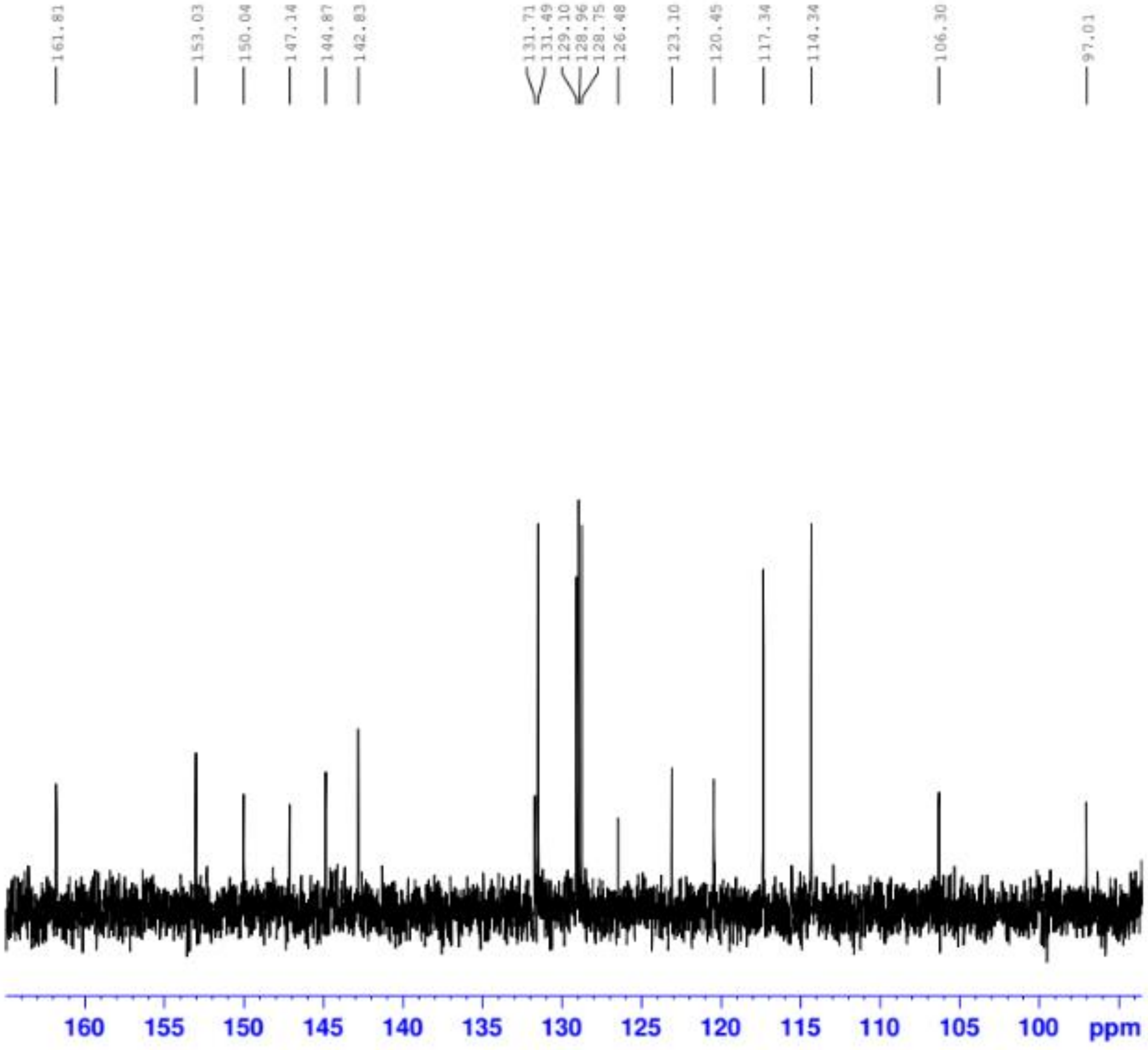

BRUKER

```
Current Data Parameters
NAME        mohammed-attia-13c
EXPNO                        2
PROCNO                       1

F2 - Acquisition Parameters
Date_                 20210704
Time                      9.25
INSTRUM                  spect
PROBHD     5 mm PABBO BB/
PULPROG                 zgpg30
TD                       65536
SOLVENT                   DMSO
NS                        1024
DS                           4
SWH                  24038.461 Hz
FIDRES                0.366798 Hz
AQ                   1.3631488 sec
RG                      205.37
DW                      20.800 usec
DE                        6.50 usec
TE                       300.0 K
D1                  2.00000000 sec
D11                 0.03000000 sec
TD0                          1

======== CHANNEL f1 ========
SFO1               100.6278588 MHz
NUC1                       13C
P1                        10.00 usec
PLW1                47.00000000 W

======== CHANNEL f2 ========
SFO2               400.1516006 MHz
NUC2                        1H
CPDPRG[2               waltz16
PCPD2                     90.00 usec
PLW2                18.00000000 W
PLW12                0.34722000 W
PLW13                0.28125000 W

F2 - Processing parameters
SI                       32768
SF                 100.6177975 MHz
WDW                         EM
SSB         0
LB                        1.00 Hz
GB          0
PC                        1.40
```

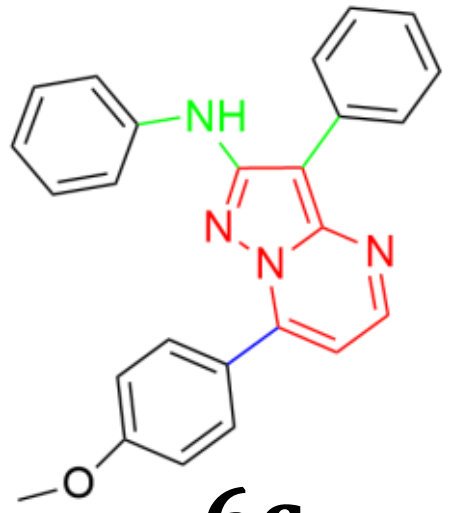

6c

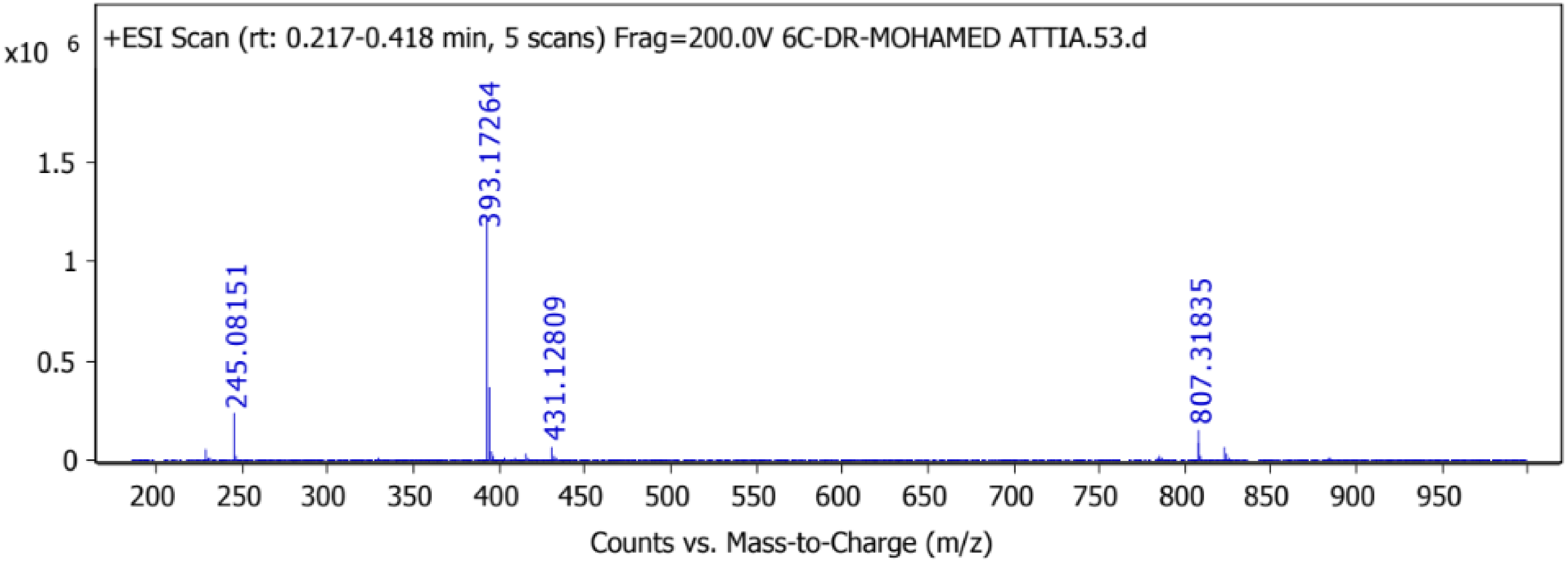

x10 6
+ESI Scan (rt: 0.217-0.418 min, 5 scans) Frag=200.0V 6C-DR-MOHAMED ATTIA.53.d
393.17264
245.08151
431.12809
807.31835
1.5
1
0.5
0
200
250
300
350
400
450
500
550
600
650
700
750
800
850
900
950
Counts vs. Mass-to-Charge (m/z)

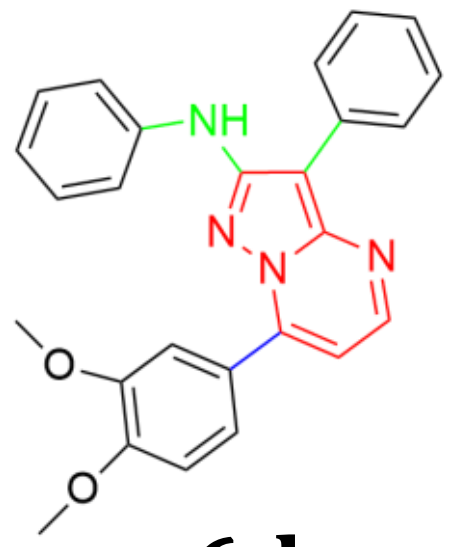

6d

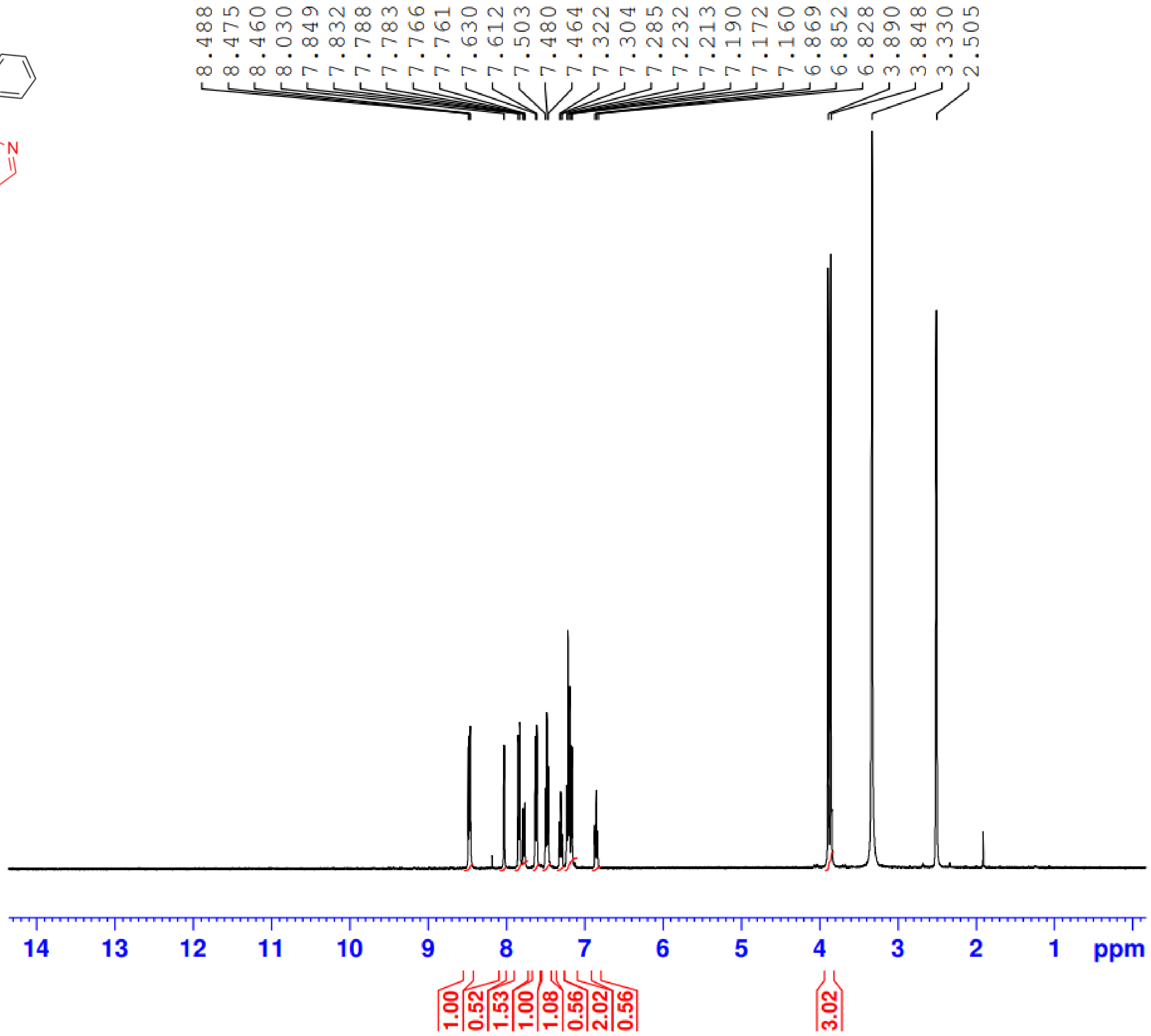

BRUKER

```
Current Data Parameters
NAME      mohammed-attia-13d
EXPNO                    1
PROCNO                   1

F2 - Acquisition Parameters
Date_             20210520
Time                 14.07
INSTRUM              spect
PROBHD   5 mm PABBO BB/
PULPROG               zg30
TD                   65536
SOLVENT               DMSO
NS                      32
DS                       2
SWH               8012.820 Hz
FIDRES            0.122266 Hz
AQ               4.0894465 sec
RG                  205.37
DW                  62.400 usec
DE                    6.50 usec
TE                   300.0 K
D1              1.00000000 sec
TD0                      1

======== CHANNEL f1 ========
SFO1           400.1524711 MHz
NUC1                    1H
P1                   12.00 usec
PLW1           18.00000000 W

F2 - Processing parameters
SI                   65536
SF             400.1500000 MHz
WDW                     EM
SSB      0
LB                    0.30 Hz
GB       0
PC                    1.00
```

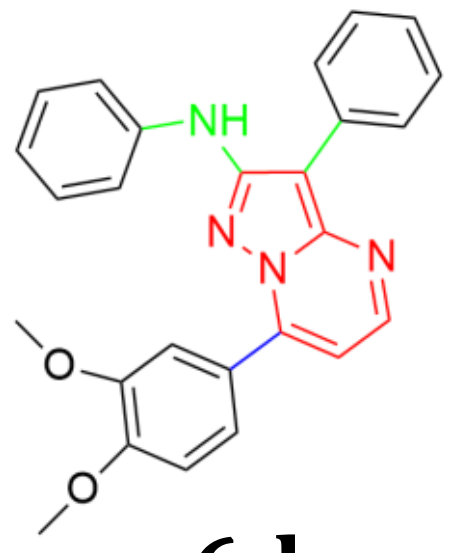

6d

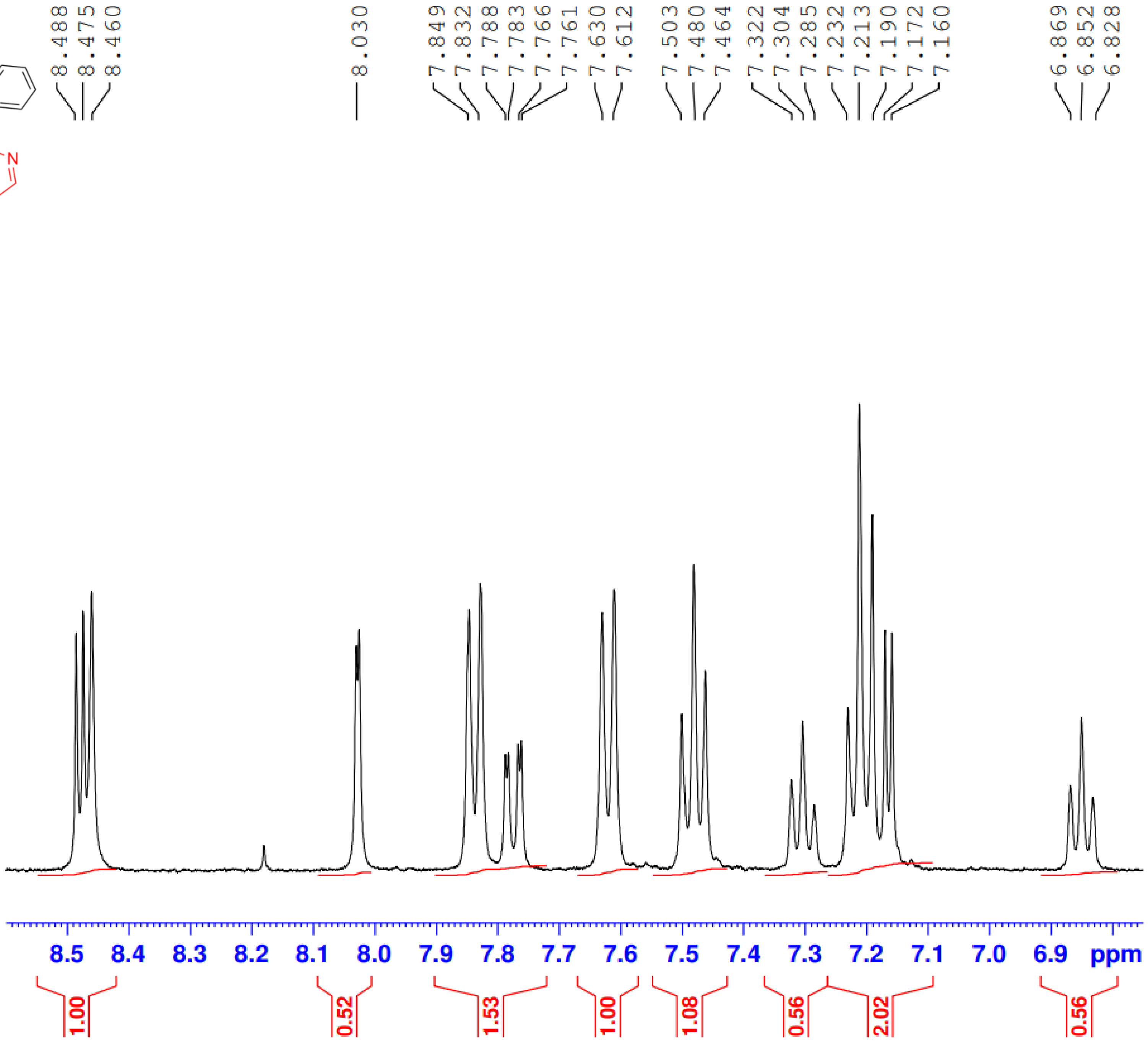

Current Data Parameters

NAME	mohammed-attia-13d
EXPNO	1
PROCNO	1

F2 - Acquisition Parameters

Date_	20210520
Time	14.07
INSTRUM	spect
PROBHD	5 mm PABBO BB/
PULPROG	zg30
TD	65536
SOLVENT	DMSO
NS	32
DS	2
SWH	8012.820 Hz
FIDRES	0.122266 Hz
AQ	4.0894465 sec
RG	205.37
DW	62.400 usec
DE	6.50 usec
TE	300.0 K
D1	1.00000000 sec
TD0	1

======== CHANNEL f1 ========

SFO1	400.1524711 MHz
NUC1	1H
P1	12.00 usec
PLW1	18.00000000 W

F2 - Processing parameters

SI	65536
SF	400.1500000 MHz
WDW	EM
SSB	0
LB	0.30 Hz
GB	0
PC	1.00

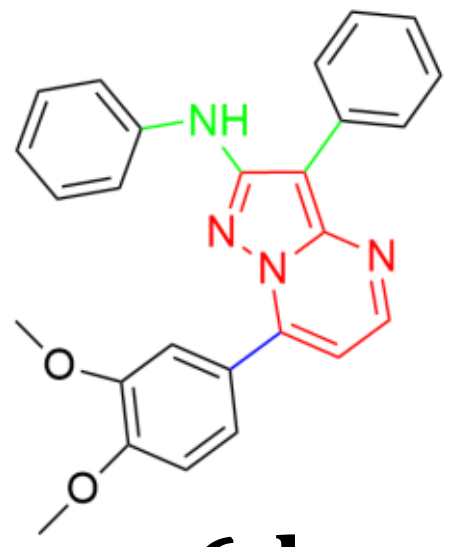
NH
N
N
N
O
O

6d

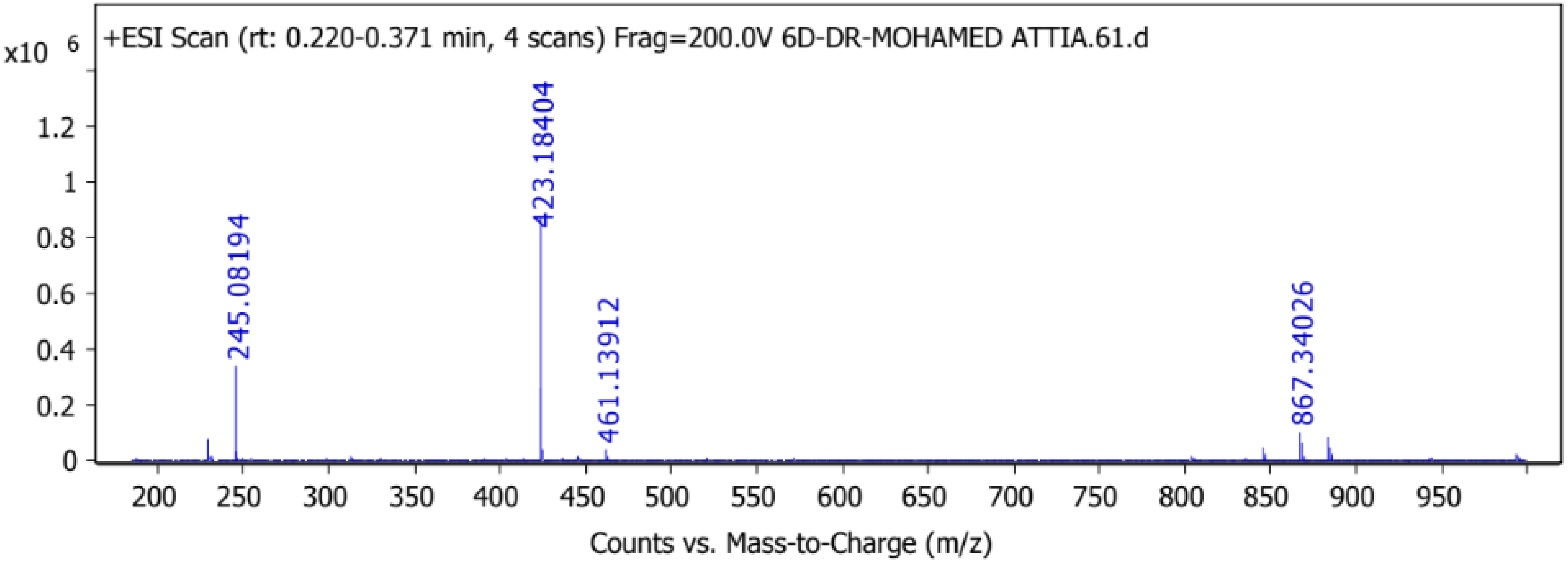
+ESI Scan (rt: 0.220-0.371 min, 4 scans) Frag=200.0V 6D-DR-MOHAMED ATTIA.61.d
x10 6
1.2
1
0.8
0.6
0.4
0.2
0
245.08194
423.18404
461.13912
867.34026
200
250
300
350
400
450
500
550
600
650
700
750
800
850
900
950
Counts vs. Mass-to-Charge (m/z)

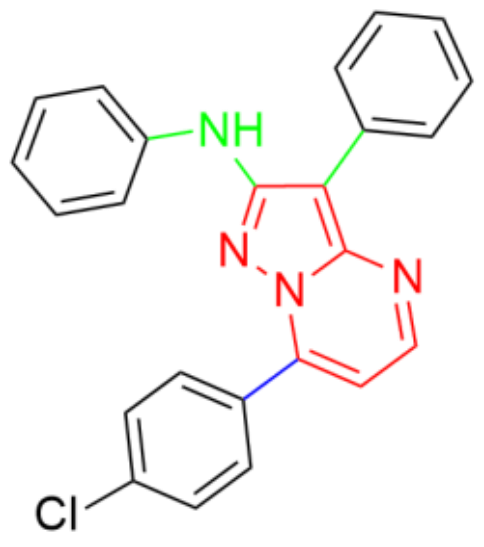

6e

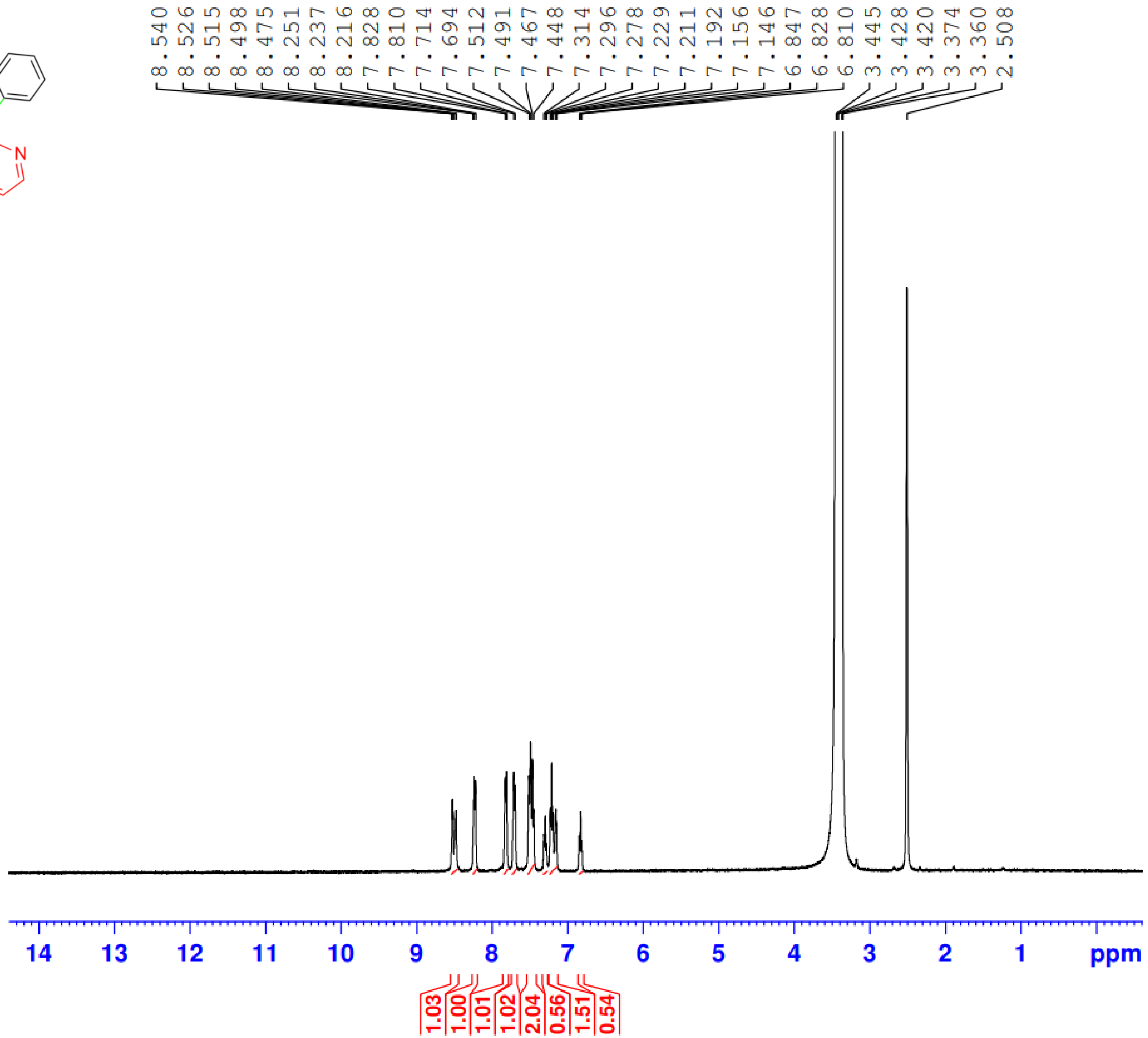

Current Data Parameters
NAME mohammed-attia-13f
EXPNO 1
PROCNO 1

F2 - Acquisition Parameters
Date_ 20210520
Time 14.57
INSTRUM spect
PROBHD 5 mm PABBO BB/
PULPROG zg30
TD 65536
SOLVENT DMSO
NS 33
DS 2
SWH 8012.820 Hz
FIDRES 0.122266 Hz
AQ 4.0894465 sec
RG 205.37
DW 62.400 usec
DE 6.50 usec
TE 300.0 K
D1 1.00000000 sec
TD0 1

======== CHANNEL f1 ========
SFO1 400.1524711 MHz
NUC1 1H
P1 12.00 usec
PLW1 18.00000000 W

F2 - Processing parameters
SI 65536
SF 400.1500000 MHz
WDW EM
SSB 0
LB 0.30 Hz
GB 0
PC 1.00

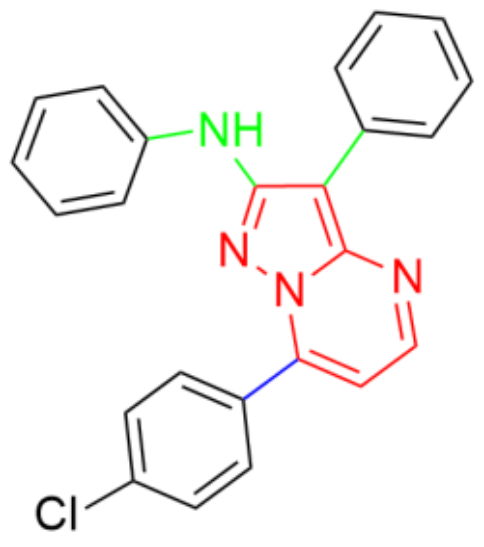

6e

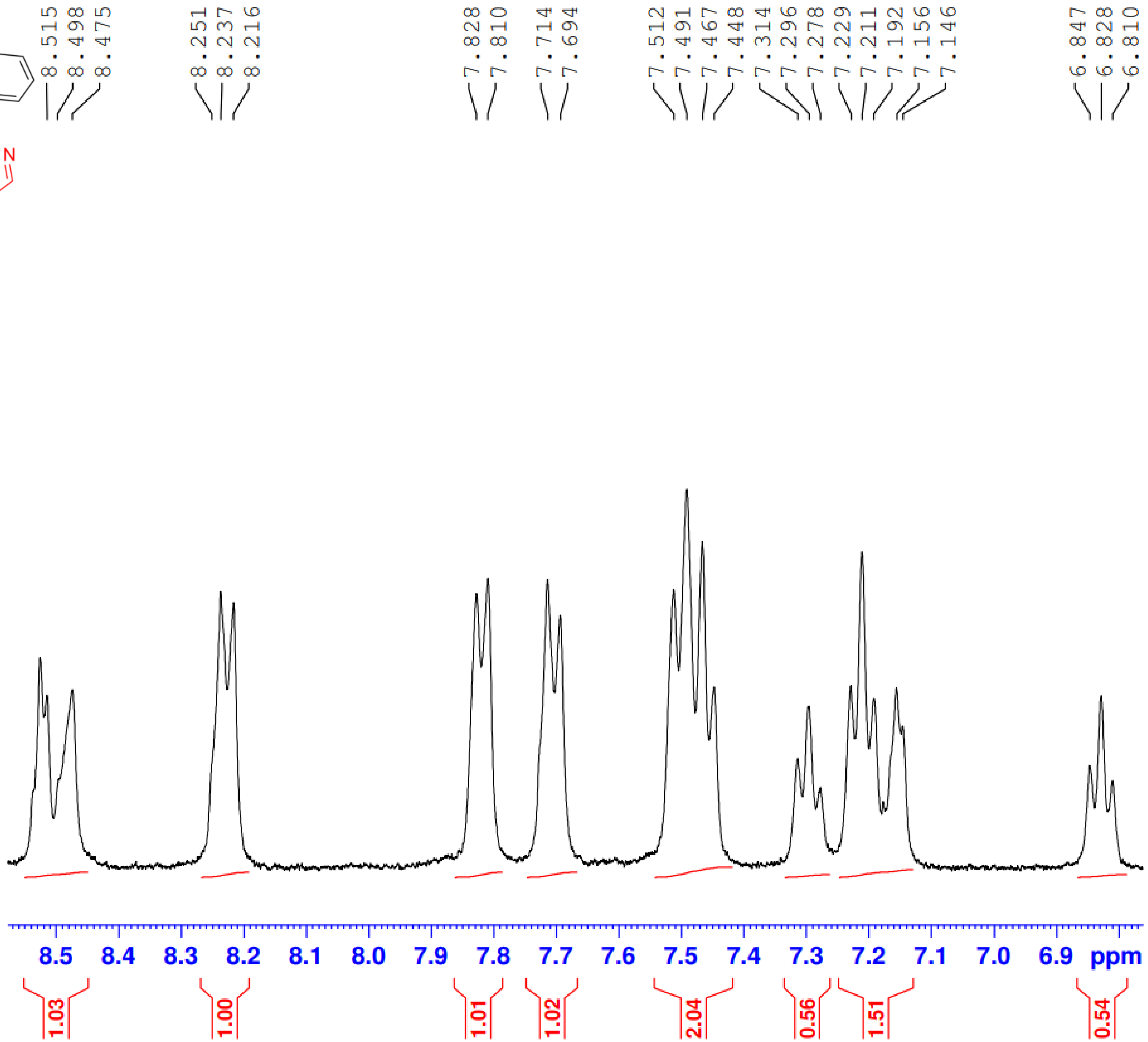


```
Current Data Parameters
NAME      mohammed-attia-13f
EXPNO                  1
PROCNO                 1

F2 - Acquisition Parameters
Date_           20210520
Time               14.57
INSTRUM            spect
PROBHD   5 mm PABBO BB/
PULPROG             zg30
TD                 65536
SOLVENT             DMSO
NS                    33
DS                     2
SWH             8012.820 Hz
FIDRES          0.122266 Hz
AQ             4.0894465 sec
RG                205.37
DW                62.400 usec
DE                  6.50 usec
TE                 300.0 K
D1            1.00000000 sec
TD0                    1

======== CHANNEL f1 ========
SFO1         400.1524711 MHz
NUC1                  1H
P1                 12.00 usec
PLW1         18.00000000 W

F2 - Processing parameters
SI                 65536
SF           400.1500000 MHz
WDW                   EM
SSB      0
LB                  0.30 Hz
GB       0
PC                  1.00
```

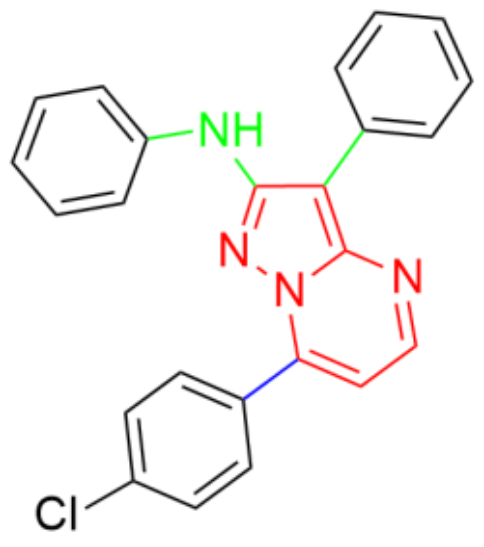

6e

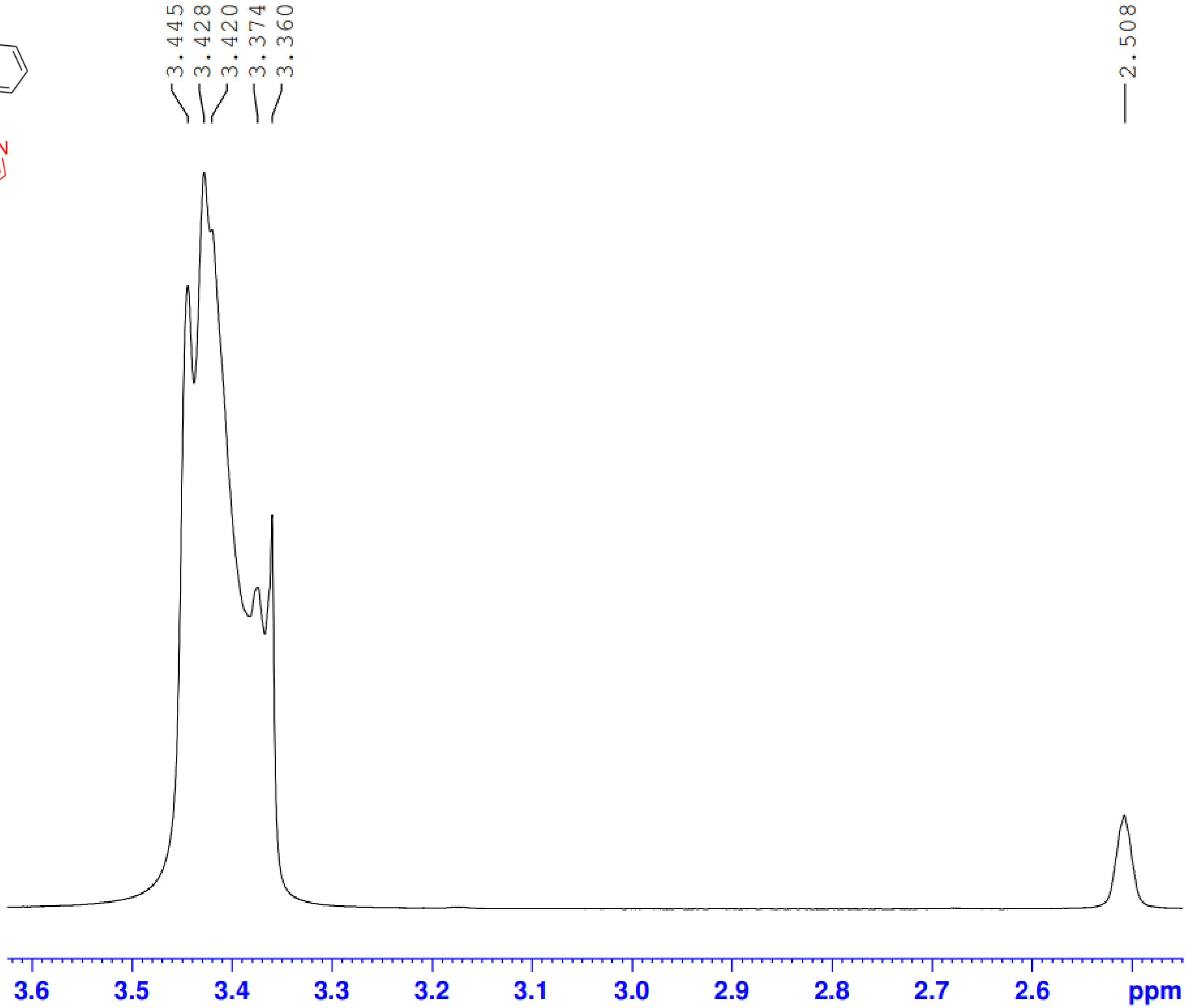

Current Data Parameters
NAME mohammed-attia-13f
EXPNO 1
PROCNO 1

F2 - Acquisition Parameters
Date_ 20210520
Time 14.57
INSTRUM spect
PROBHD 5 mm PABBO BB/
PULPROG zg30
TD 65536
SOLVENT DMSO
NS 33
DS 2
SWH 8012.820 Hz
FIDRES 0.122266 Hz
AQ 4.0894465 sec
RG 205.37
DW 62.400 usec
DE 6.50 usec
TE 300.0 K
D1 1.00000000 sec
TD0 1

======== CHANNEL f1 ========
SFO1 400.1524711 MHz
NUC1 1H
P1 12.00 usec
PLW1 18.00000000 W

F2 - Processing parameters
SI 65536
SF 400.1500000 MHz
WDW EM
SSB 0
LB 0.30 Hz
GB 0
PC 1.00

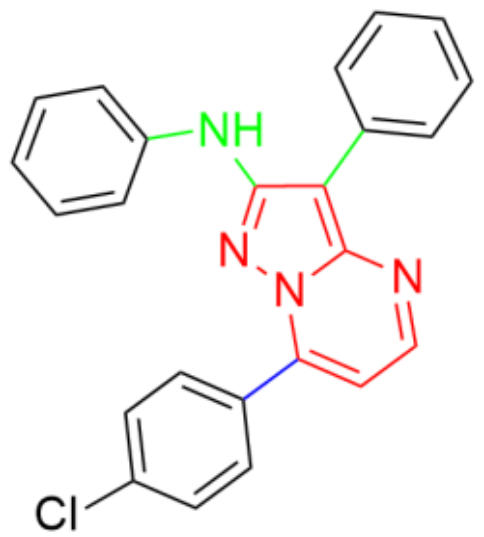

6e

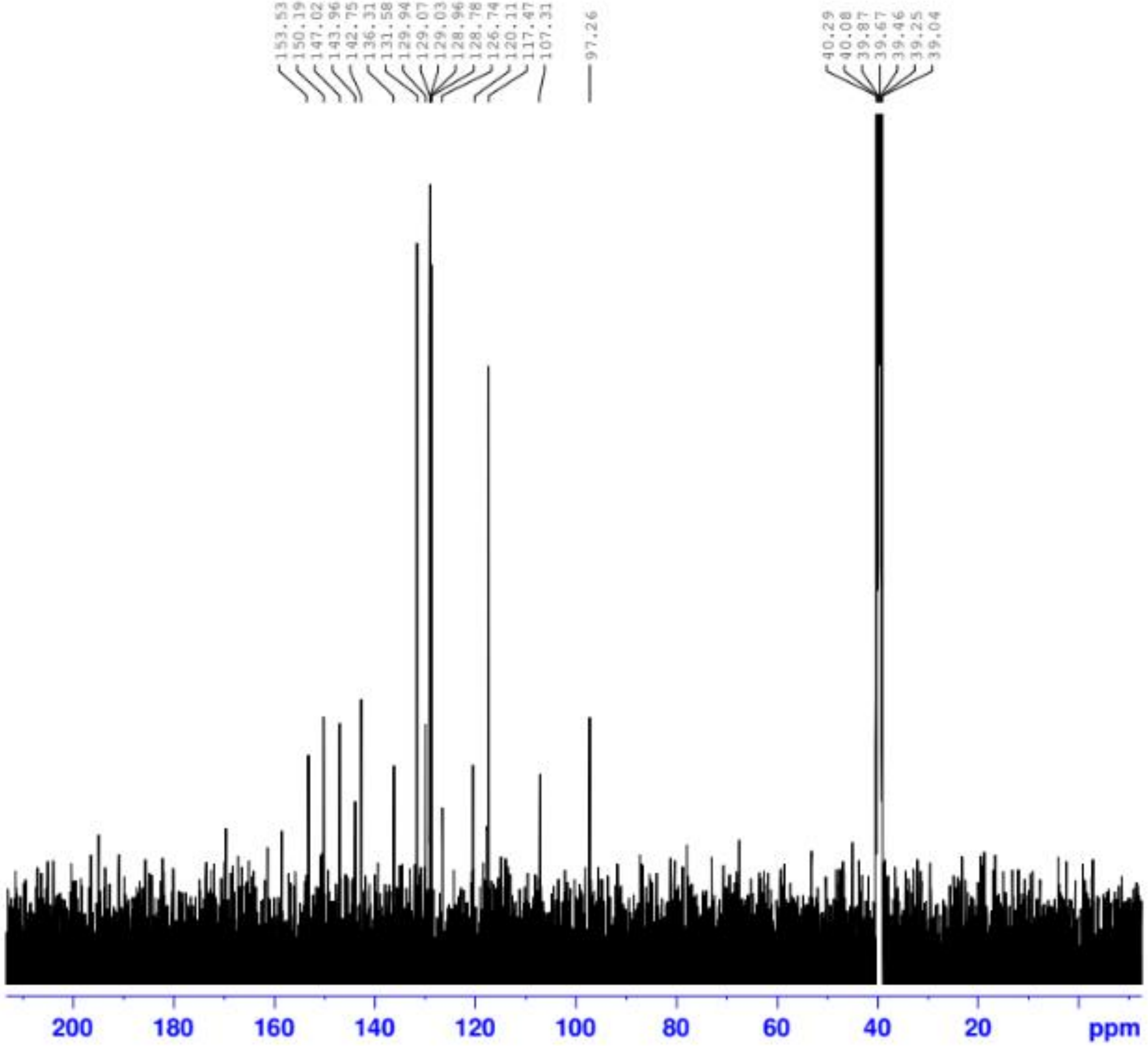

BRUKER

Current Data Parameters
NAME mohammed-attia-13f
EXPNO 2
PROCNO 1

F2 - Acquisition Parameters
Date_ 20210801
Time 16.27
INSTRUM spect
PROBHD 5 mm PABBO BB/
PULPROG zgpg30
TD 65536
SOLVENT DMSO
NS 2000
DS 4
SWH 24038.461 Hz
FIDRES 0.366798 Hz
AQ 1.3631488 sec
RG 205.37
DW 20.800 usec
DE 6.50 usec
TE 300.0 K
D1 2.00000000 sec
D11 0.03000000 sec
TD0 1

======== CHANNEL f1 ========
SFO1 100.6278588 MHz
NUC1 13C
P1 10.00 usec
PLW1 47.00000000 W

======== CHANNEL f2 ========
SFO2 400.1516006 MHz
NUC2 1H
CPDPRG[2 waltz16
PCPD2 90.00 usec
PLW2 18.00000000 W
PLW12 0.34722000 W
PLW13 0.28125000 W

F2 - Processing parameters
SI 32768
SF 100.6177975 MHz
WDW EM
SSB 0
LB 1.00 Hz
GB 0
PC 1.40

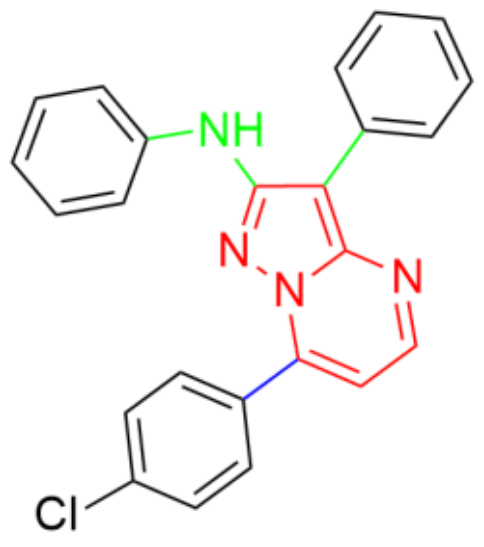

6e

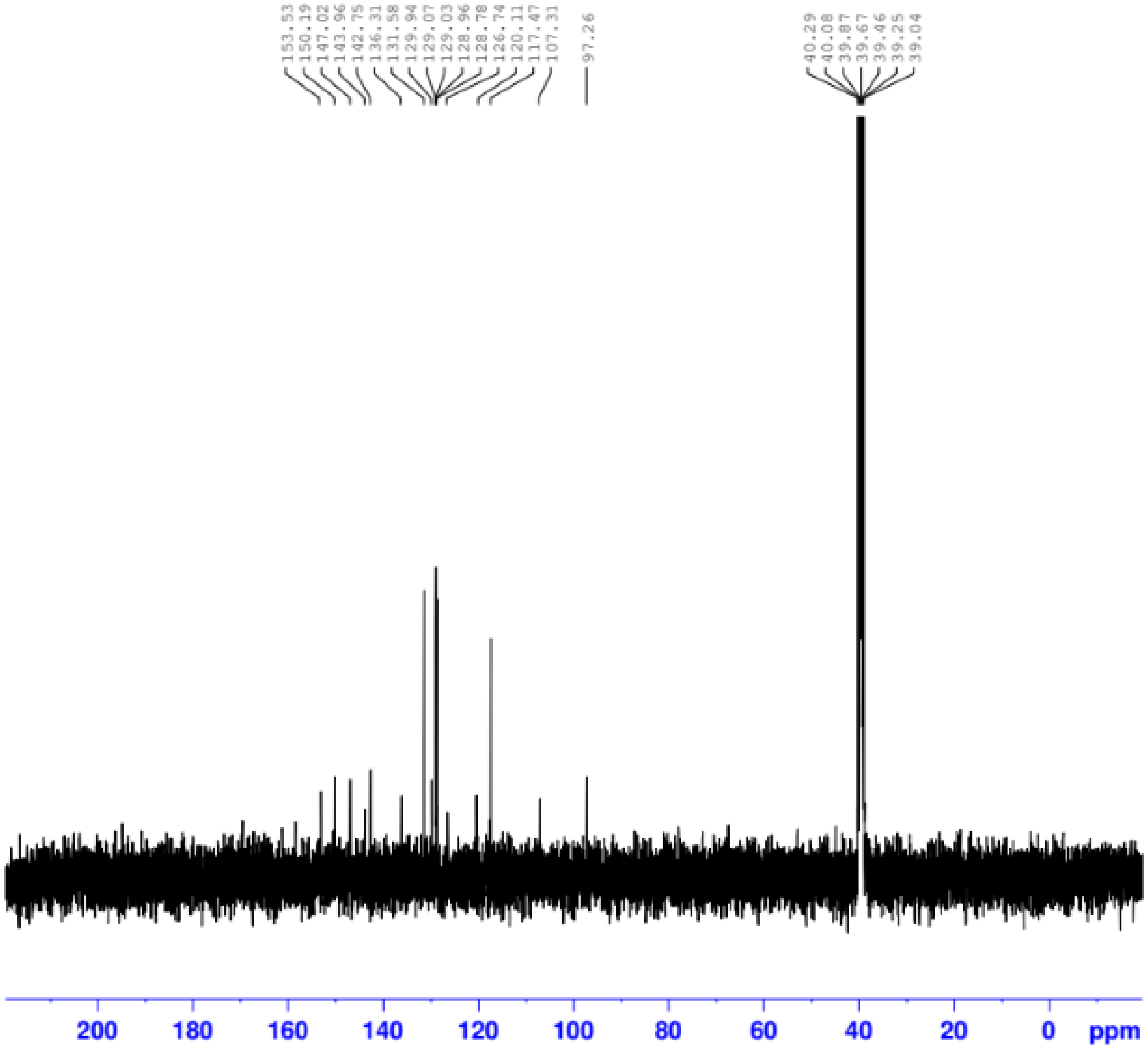

Current Data Parameters

NAME	mohammed-attia-13f	
EXPNO	2	
PROCNO	1	

F2 - Acquisition Parameters

Date_	20210801	
Time	16.27	
INSTRUM	spect	
PROBHD	5 mm PABBO BB/	
PULPROG	zgpg30	
TD	65536	
SOLVENT	DMSO	
NS	2000	
DS	4	
SWH	24038.461	Hz
FIDRES	0.366798	Hz
AQ	1.3631488	sec
RG	205.37	
DW	20.800	usec
DE	6.50	usec
TE	300.0	K
D1	2.00000000	sec
D11	0.03000000	sec
TD0	1	

======== CHANNEL f1 ========

SFO1	100.6278588	MHz
NUC1	13C	
P1	10.00	usec
PLW1	47.00000000	W

======== CHANNEL f2 ========

SFO2	400.1516006	MHz
NUC2	1H	
CPDPRG[2	waltz16	
PCPD2	90.00	usec
PLW2	18.00000000	W
PLW12	0.34722000	W
PLW13	0.28125000	W

F2 - Processing parameters

SI	32768	
SF	100.6177975	MHz
WDW	EM	
SSB	0	
LB	1.00	Hz
GB	0	
PC	1.40	

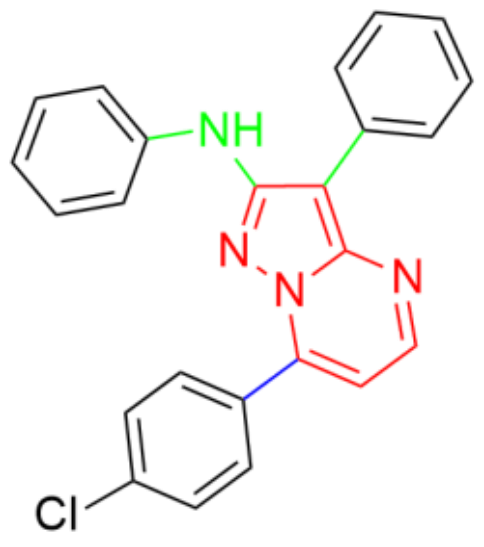

6e

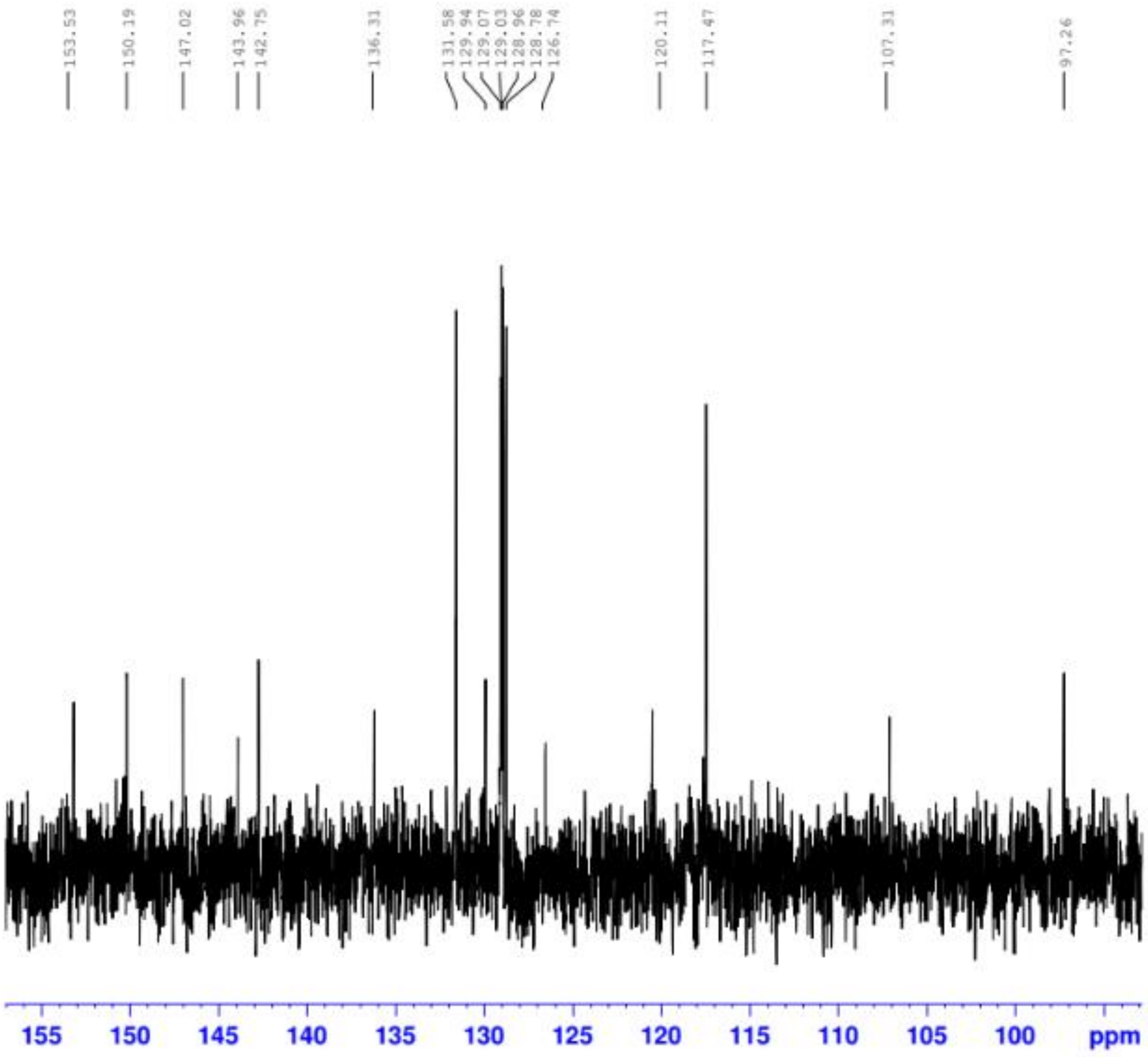


```
Current Data Parameters
NAME      mohammed-attia-13f
EXPNO                      2
PROCNO                     1

F2 - Acquisition Parameters
Date_               20210801
Time                   16.27
INSTRUM                spect
PROBHD     5 mm PABBO BB/
PULPROG              zgpg30
TD                     65536
SOLVENT                 DMSO
NS                      2000
DS                         4
SWH                24038.461 Hz
FIDRES              0.366798 Hz
AQ                 1.3631488 sec
RG                    205.37
DW                    20.800 usec
DE                      6.50 usec
TE                     300.0 K
D1                2.00000000 sec
D11               0.03000000 sec
TD0                        1

======== CHANNEL f1 ========
SFO1             100.6278588 MHz
NUC1                     13C
P1                     10.00 usec
PLW1             47.00000000 W

======== CHANNEL f2 ========
SFO2             400.1516006 MHz
NUC2                      1H
CPDPRG[2             waltz16
PCPD2                  90.00 usec
PLW2             18.00000000 W
PLW12             0.34722000 W
PLW13             0.28125000 W

F2 - Processing parameters
SI                     32768
SF               100.6177975 MHz
WDW                       EM
SSB         0
LB                      1.00 Hz
GB          0
PC                      1.40
```

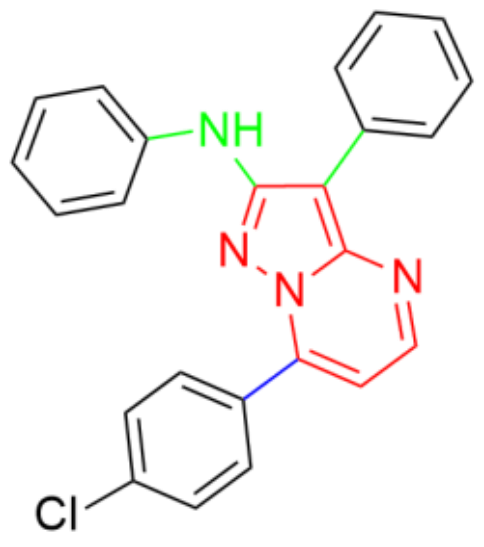
NH
N
N
N
Cl

6e

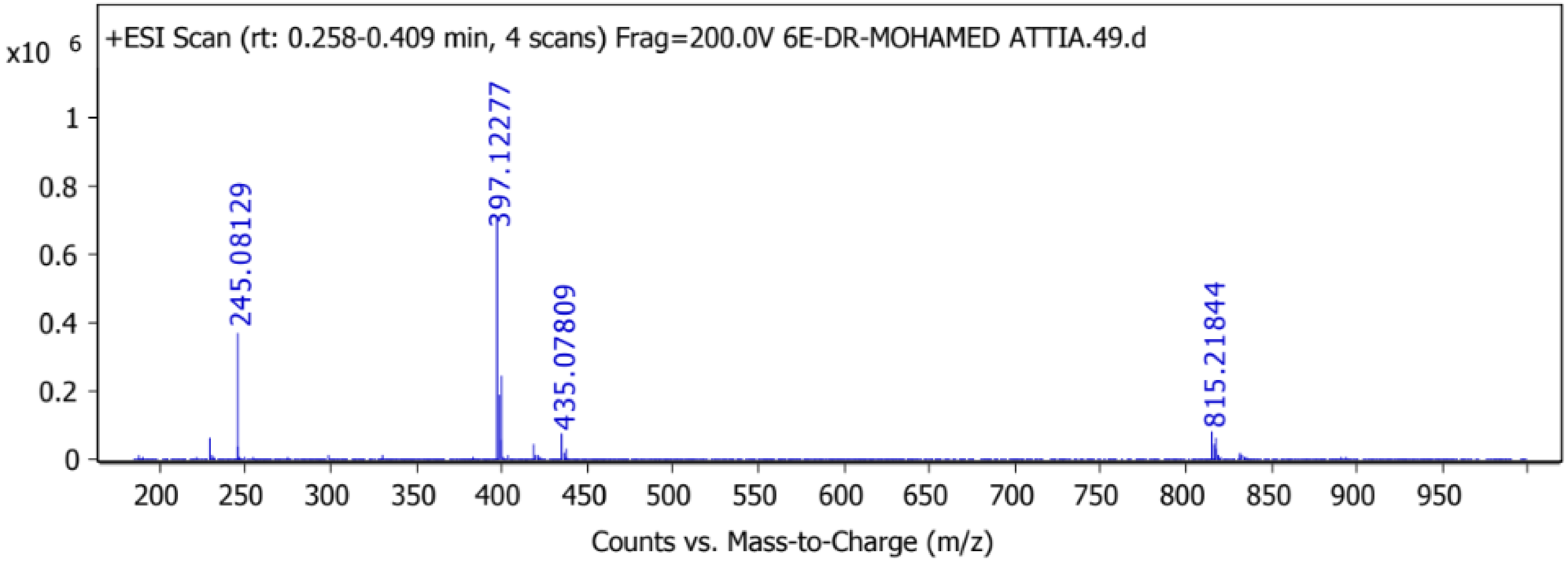
+ESI Scan (rt: 0.258-0.409 min, 4 scans) Frag=200.0V 6E-DR-MOHAMED ATTIA.49.d
x10 6
1
0.8
0.6
0.4
0.2
0
245.08129
397.12277
435.07809
815.21844
200
250
300
350
400
450
500
550
600
650
700
750
800
850
900
950
Counts vs. Mass-to-Charge (m/z)

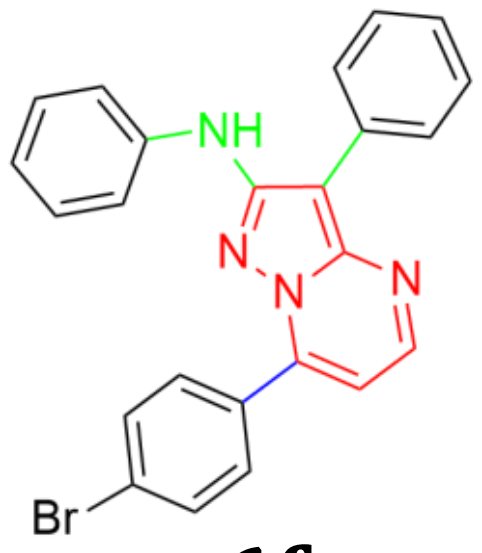

6f

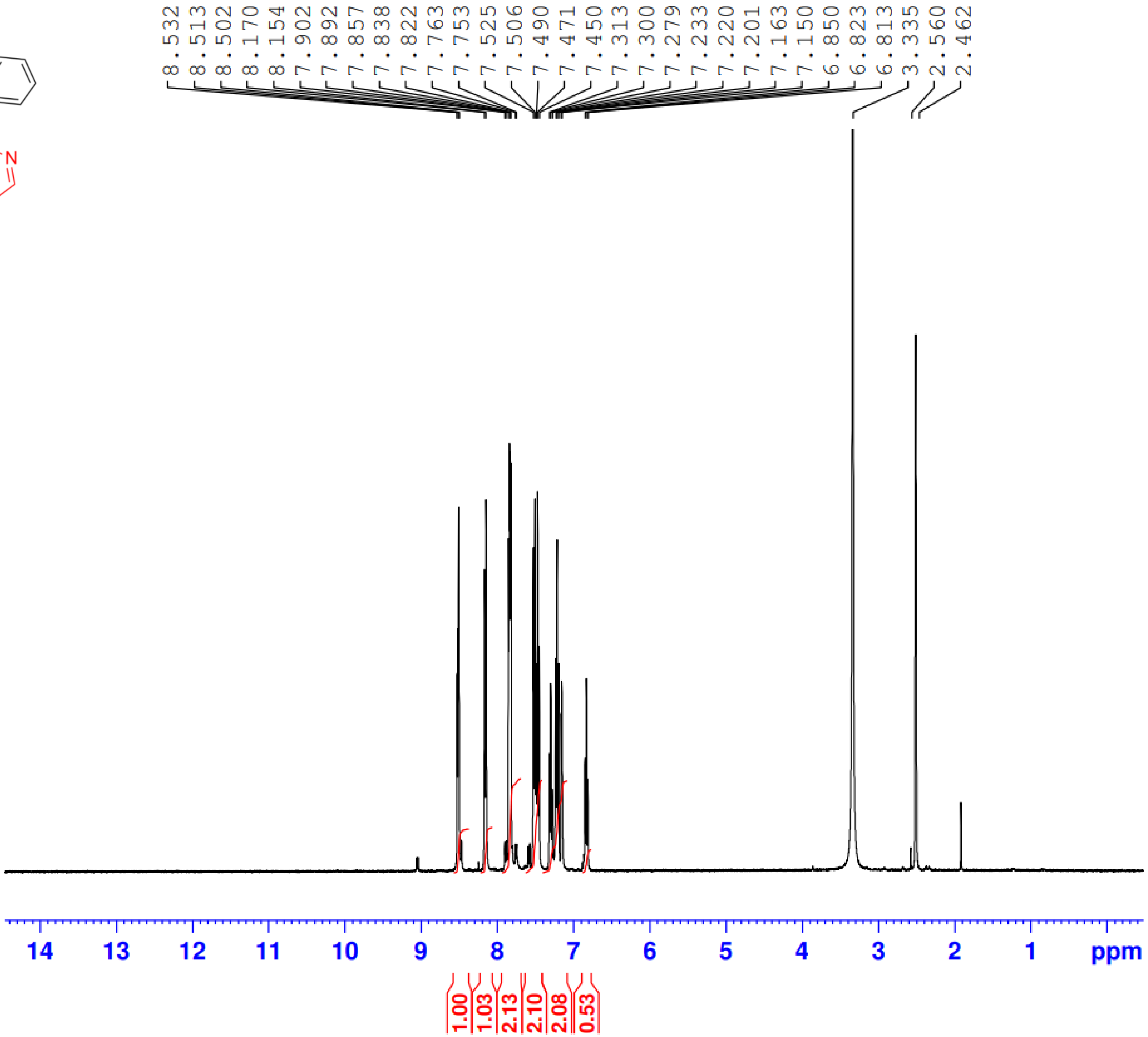


```
Current Data Parameters
NAME       mohammed-attia-13g
EXPNO                       1
PROCNO                      1

F2 - Acquisition Parameters
Date_              20210520
Time                  14.11
INSTRUM               spect
PROBHD    5 mm PABBO BB/
PULPROG                zg30
TD                    65536
SOLVENT                DMSO
NS                       21
DS                        2
SWH               8012.820 Hz
FIDRES            0.122266 Hz
AQ               4.0894465 sec
RG                   205.37
DW                   62.400 usec
DE                     6.50 usec
TE                    300.0 K
D1               1.00000000 sec
TD0                       1

======== CHANNEL f1 ========
SFO1           400.1524711 MHz
NUC1                     1H
P1                    12.00 usec
PLW1           18.00000000 W

F2 - Processing parameters
SI                    65536
SF             400.1500000 MHz
WDW                      EM
SSB       0
LB                     0.30 Hz
GB        0
PC                     1.00
```

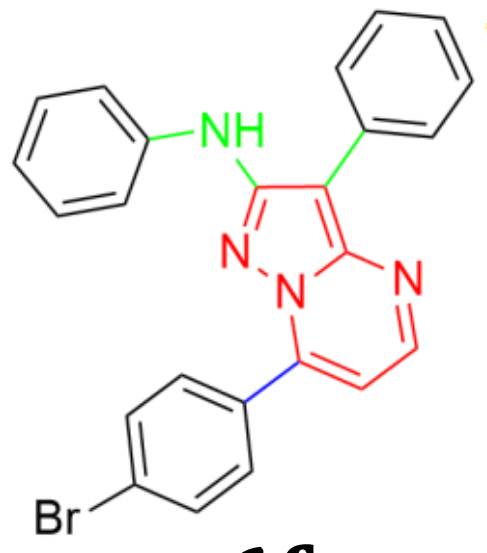

6f

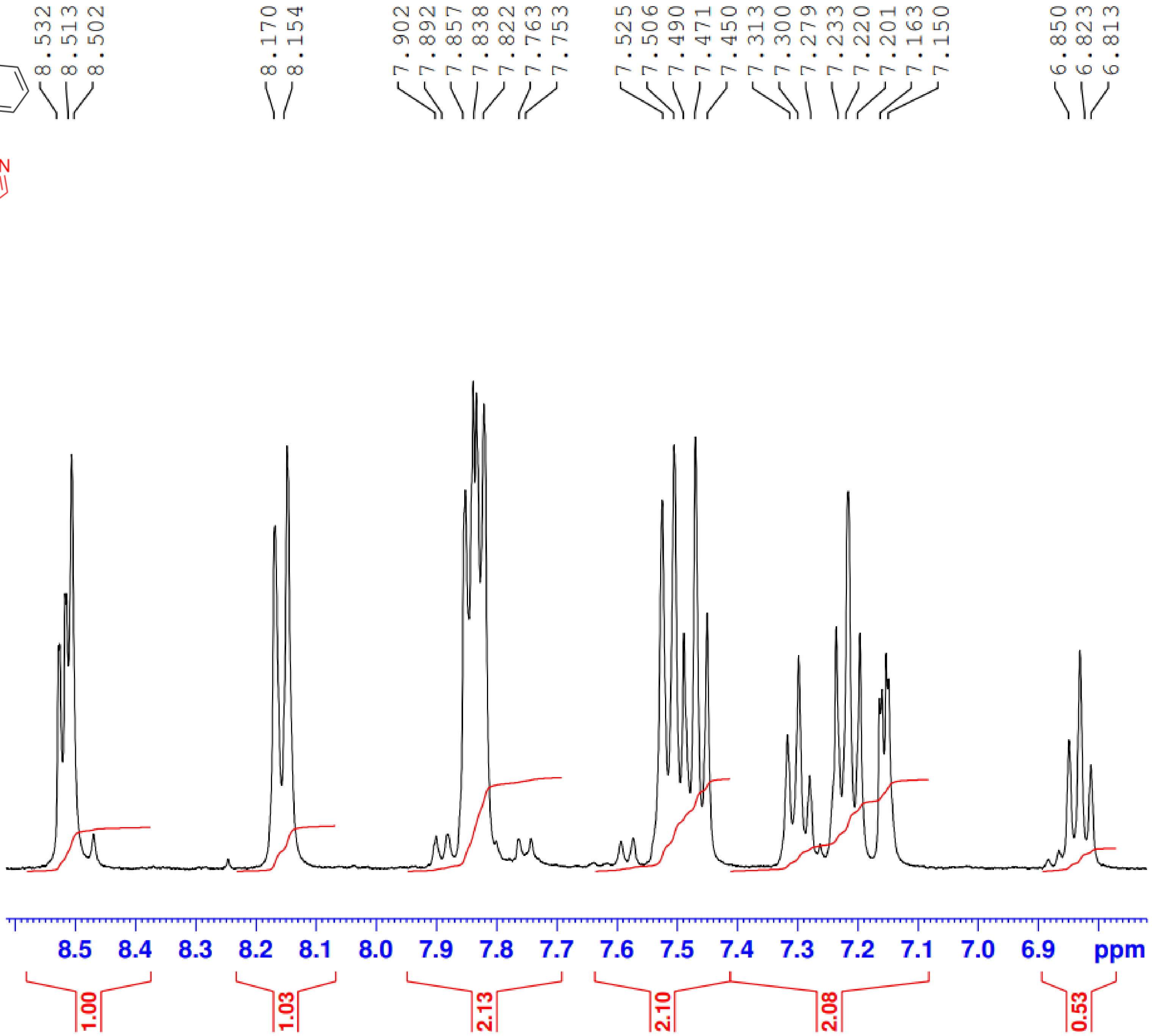


```
Current Data Parameters
NAME      mohammed-attia-13g
EXPNO                  1
PROCNO                 1

F2 - Acquisition Parameters
Date_           20210520
Time               14.11
INSTRUM            spect
PROBHD    5 mm PABBO BB/
PULPROG             zg30
TD                 65536
SOLVENT             DMSO
NS                    21
DS                     2
SWH             8012.820 Hz
FIDRES          0.122266 Hz
AQ             4.0894465 sec
RG                205.37
DW                62.400 usec
DE                  6.50 usec
TE                 300.0 K
D1            1.00000000 sec
TD0                    1

======== CHANNEL f1 ========
SFO1         400.1524711 MHz
NUC1                  1H
P1                 12.00 usec
PLW1         18.00000000 W

F2 - Processing parameters
SI                 65536
SF           400.1500000 MHz
WDW                   EM
SSB      0
LB                  0.30 Hz
GB       0
PC                  1.00
```

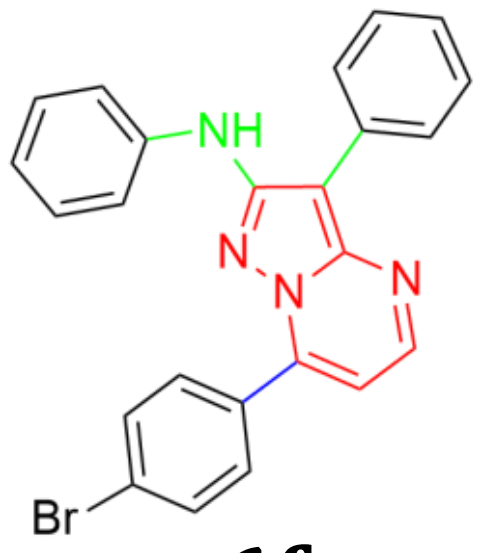

6f

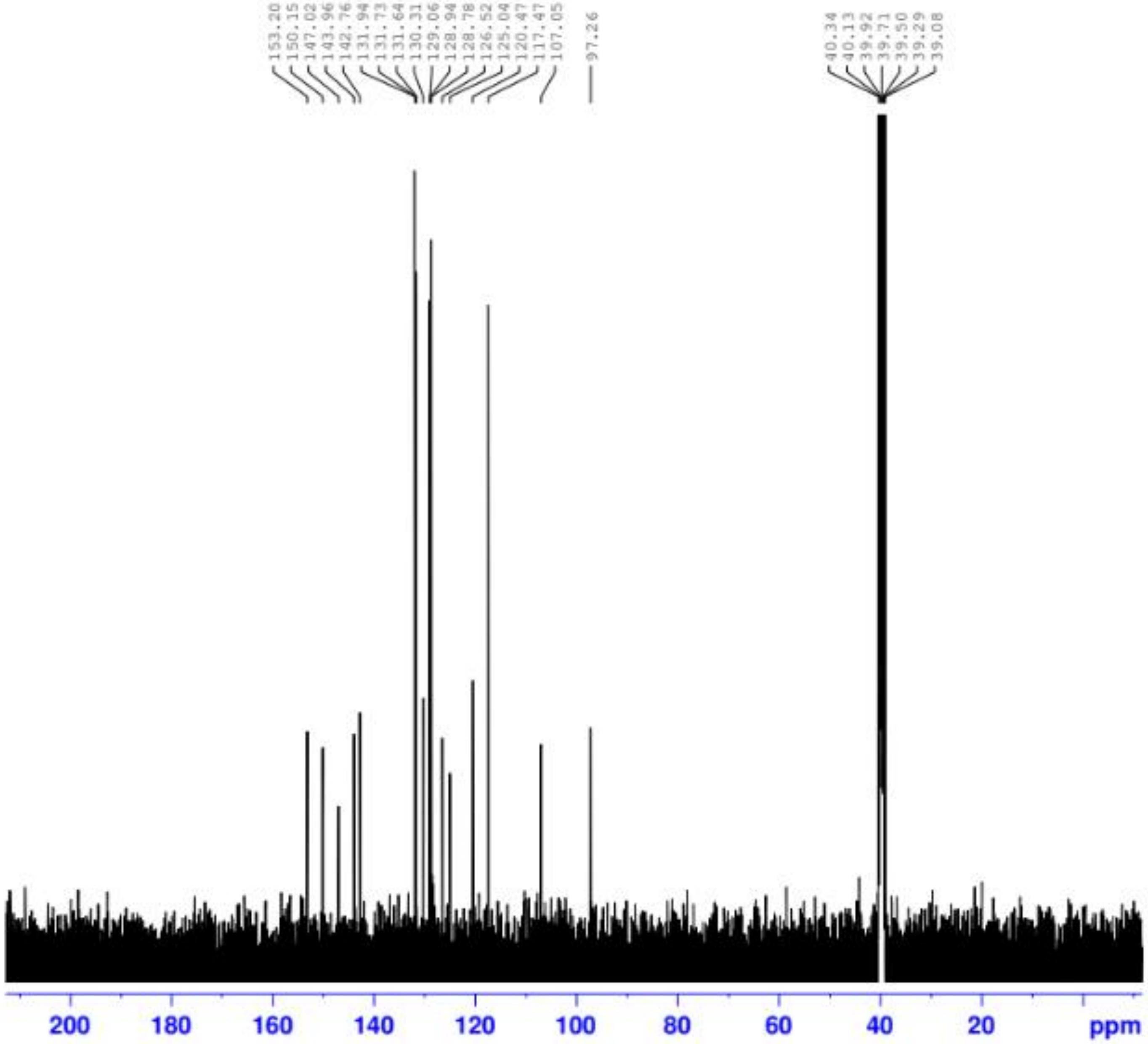

BRUKER

Current Data Parameters
NAME mohammed-attia-13g
EXPNO 2
PROCNO 1

F2 - Acquisition Parameters
Date_ 20210624
Time 17.19
INSTRUM spect
PROBHD 5 mm PABBO BB/
PULPROG zgpg30
TD 65536
SOLVENT DMSO
NS 1001
DS 4
SWH 24038.461 Hz
FIDRES 0.366798 Hz
AQ 1.3631488 sec
RG 205.37
DW 20.800 usec
DE 6.50 usec
TE 300.0 K
D1 2.00000000 sec
D11 0.03000000 sec
TD0 1

======== CHANNEL f1 ========
SFO1 100.6278588 MHz
NUC1 13C
P1 10.00 usec
PLW1 47.00000000 W

======== CHANNEL f2 ========
SFO2 400.1516006 MHz
NUC2 1H
CPDPRG[2 waltz16
PCPD2 90.00 usec
PLW2 18.00000000 W
PLW12 0.34722000 W
PLW13 0.28125000 W

F2 - Processing parameters
SI 32768
SF 100.6177975 MHz
WDW EM
SSB 0
LB 1.00 Hz
GB 0
PC 1.40

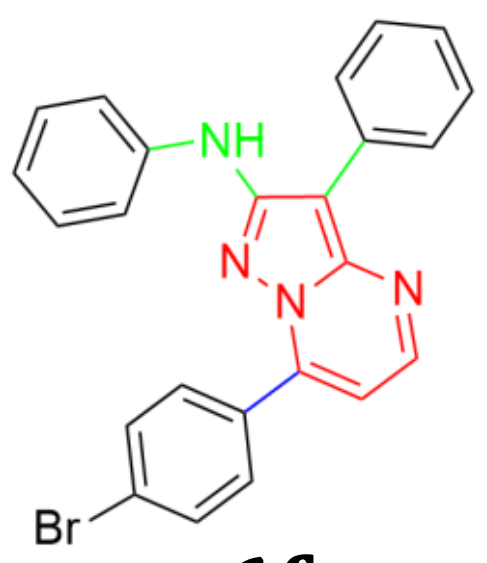

6f

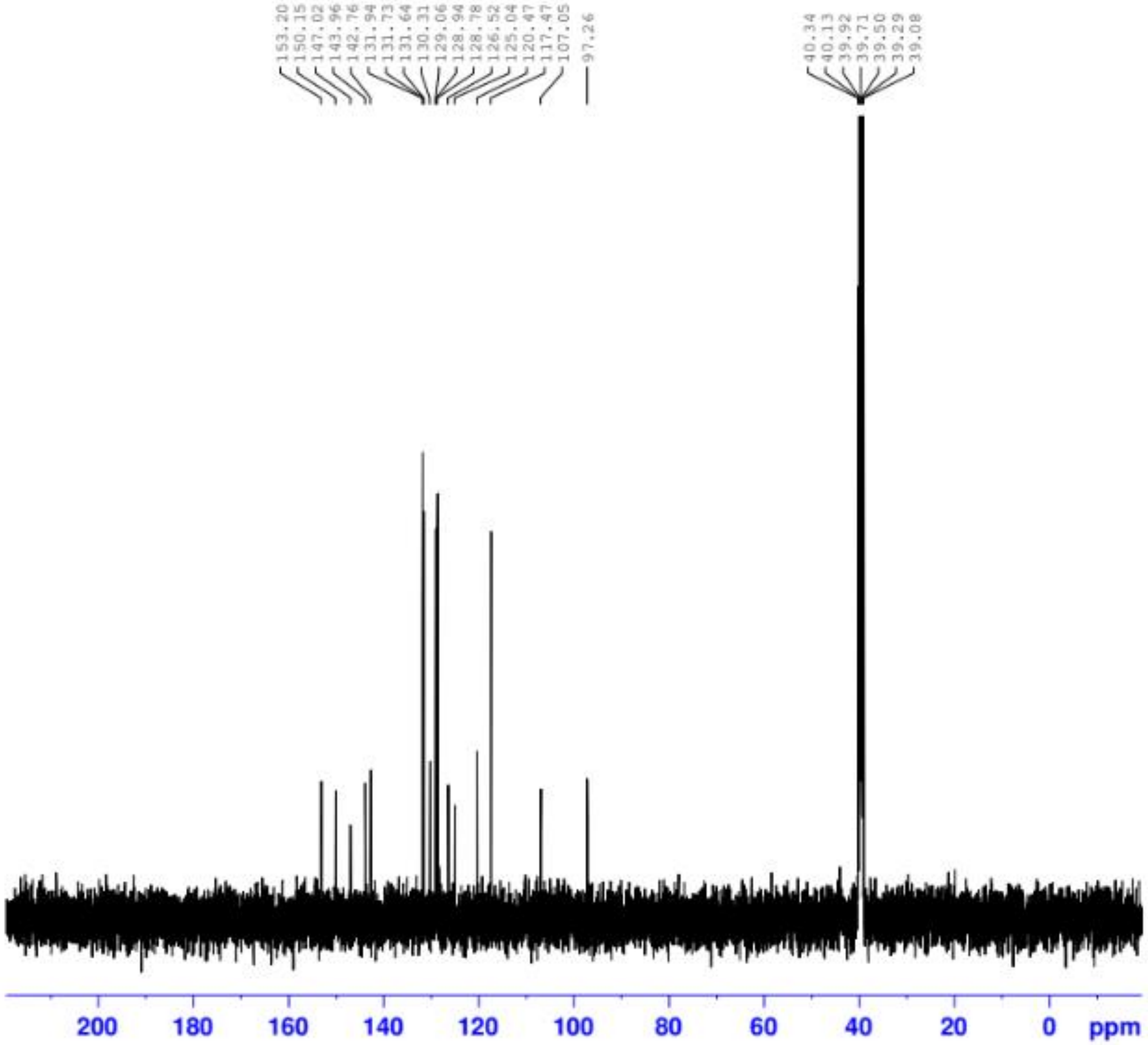

BRUKER

```
Current Data Parameters
NAME       mohammed-attia-13g
EXPNO                     2
PROCNO                    1

F2 - Acquisition Parameters
Date_              20210624
Time                  17.19
INSTRUM               spect
PROBHD   5 mm PABBO BB/
PULPROG             zgpg30
TD                    65536
SOLVENT                DMSO
NS                     1001
DS                        4
SWH               24038.461 Hz
FIDRES             0.366798 Hz
AQ                1.3631488 sec
RG                   205.37
DW                   20.800 usec
DE                     6.50 usec
TE                    300.0 K
D1               2.00000000 sec
D11              0.03000000 sec
TD0                       1

======== CHANNEL f1 ========
SFO1            100.6278588 MHz
NUC1                    13C
P1                    10.00 usec
PLW1            47.00000000 W

======== CHANNEL f2 ========
SFO2            400.1516006 MHz
NUC2                     1H
CPDPRG[2            waltz16
PCPD2                 90.00 usec
PLW2            18.00000000 W
PLW12            0.34722000 W
PLW13            0.28125000 W

F2 - Processing parameters
SI                    32768
SF              100.6177975 MHz
WDW                      EM
SSB      0
LB                     1.00 Hz
GB       0
PC                     1.40
```

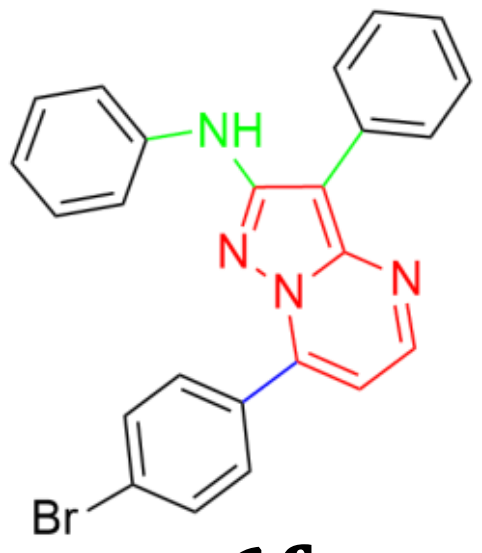

6f

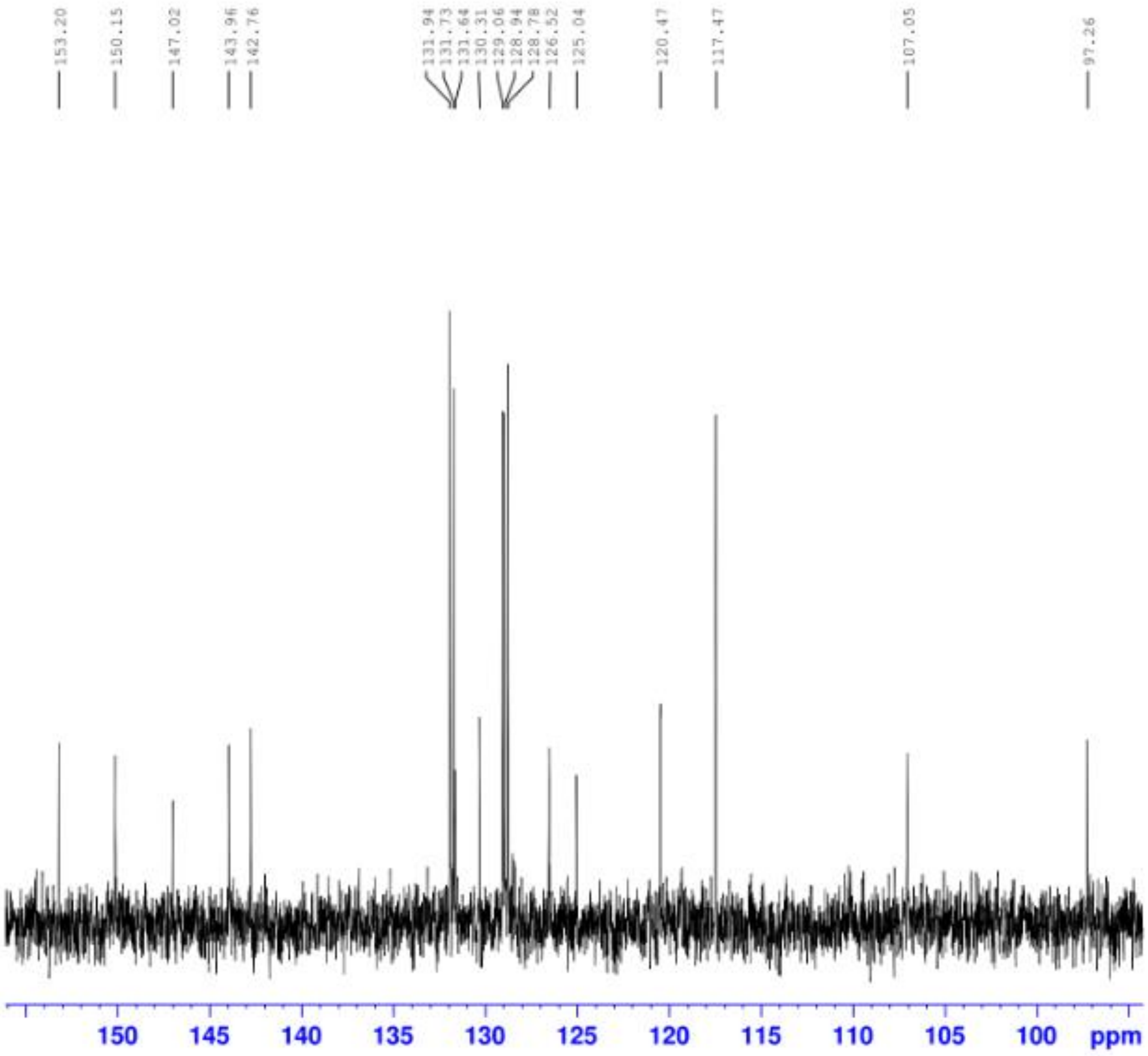

BRUKER

```
Current Data Parameters
NAME       mohammed-attia-13g
EXPNO                       2
PROCNO                      1

F2 - Acquisition Parameters
Date_              20210624
Time                  17.19
INSTRUM               spect
PROBHD    5 mm PABBO BB/
PULPROG             zgpg30
TD                   65536
SOLVENT               DMSO
NS                    1001
DS                       4
SWH             24038.461 Hz
FIDRES           0.366798 Hz
AQ              1.3631488 sec
RG                  205.37
DW                  20.800 usec
DE                    6.50 usec
TE                   300.0 K
D1              2.00000000 sec
D11             0.03000000 sec
TD0                      1

======== CHANNEL f1 ========
SFO1          100.6278588 MHz
NUC1                  13C
P1                  10.00 usec
PLW1          47.00000000 W

======== CHANNEL f2 ========
SFO2          400.1516006 MHz
NUC2                   1H
CPDPRG[2            waltz16
PCPD2               90.00 usec
PLW2          18.00000000 W
PLW12          0.34722000 W
PLW13          0.28125000 W

F2 - Processing parameters
SI                  32768
SF            100.6177975 MHz
WDW                    EM
SSB      0
LB                   1.00 Hz
GB       0
PC                   1.40
```

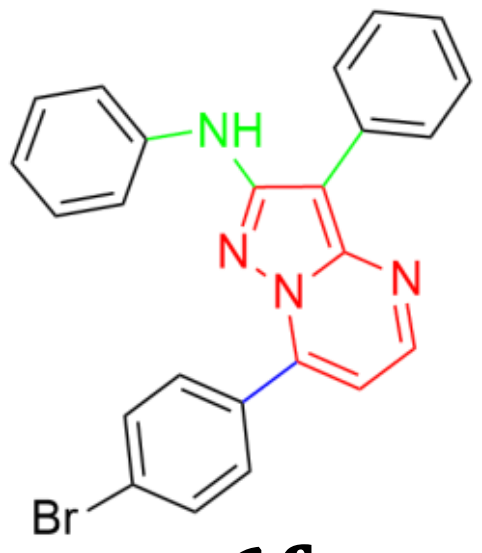
NH
N
N
N
Br

6f

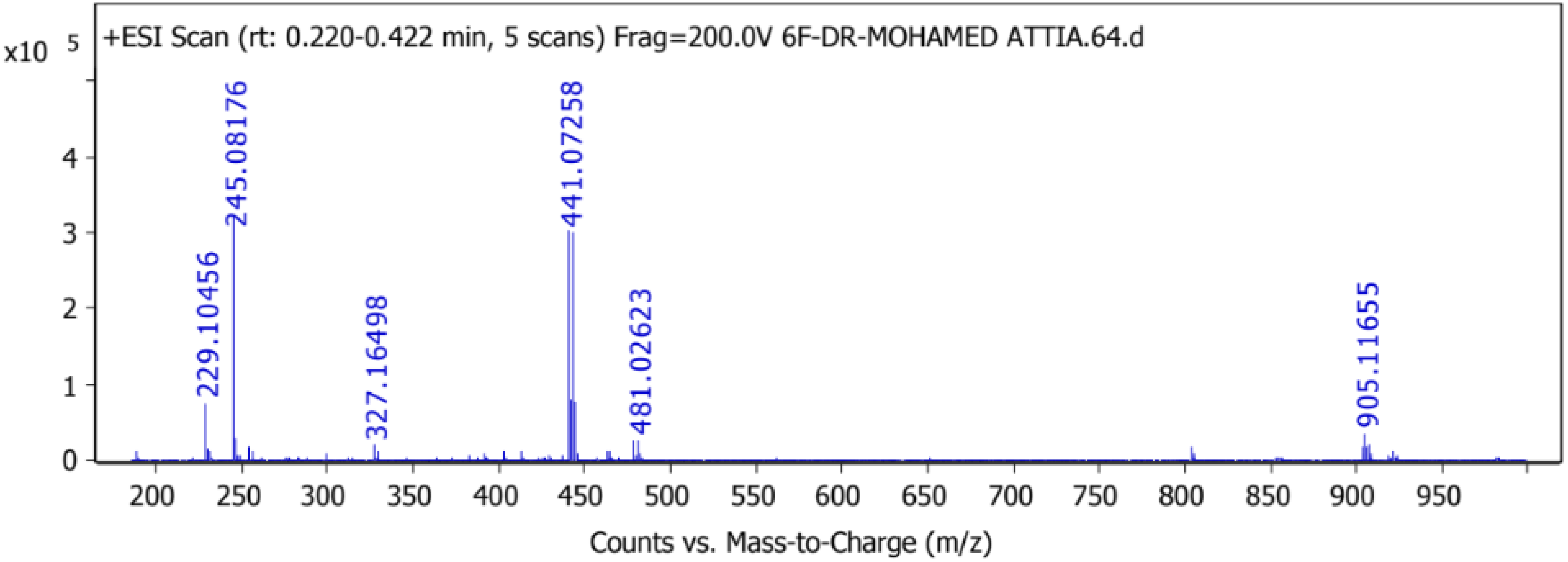
+ESI Scan (rt: 0.220-0.422 min, 5 scans) Frag=200.0V 6F-DR-MOHAMED ATTIA.64.d
x10 5
0
1
2
3
4
229.10456
245.08176
327.16498
441.07258
481.02623
905.11655
200
250
300
350
400
450
500
550
600
650
700
750
800
850
900
950
Counts vs. Mass-to-Charge (m/z)

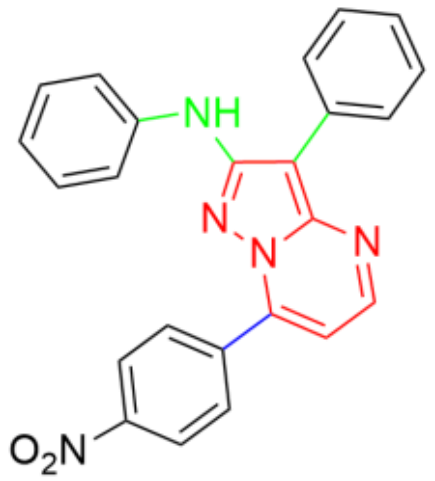

6g

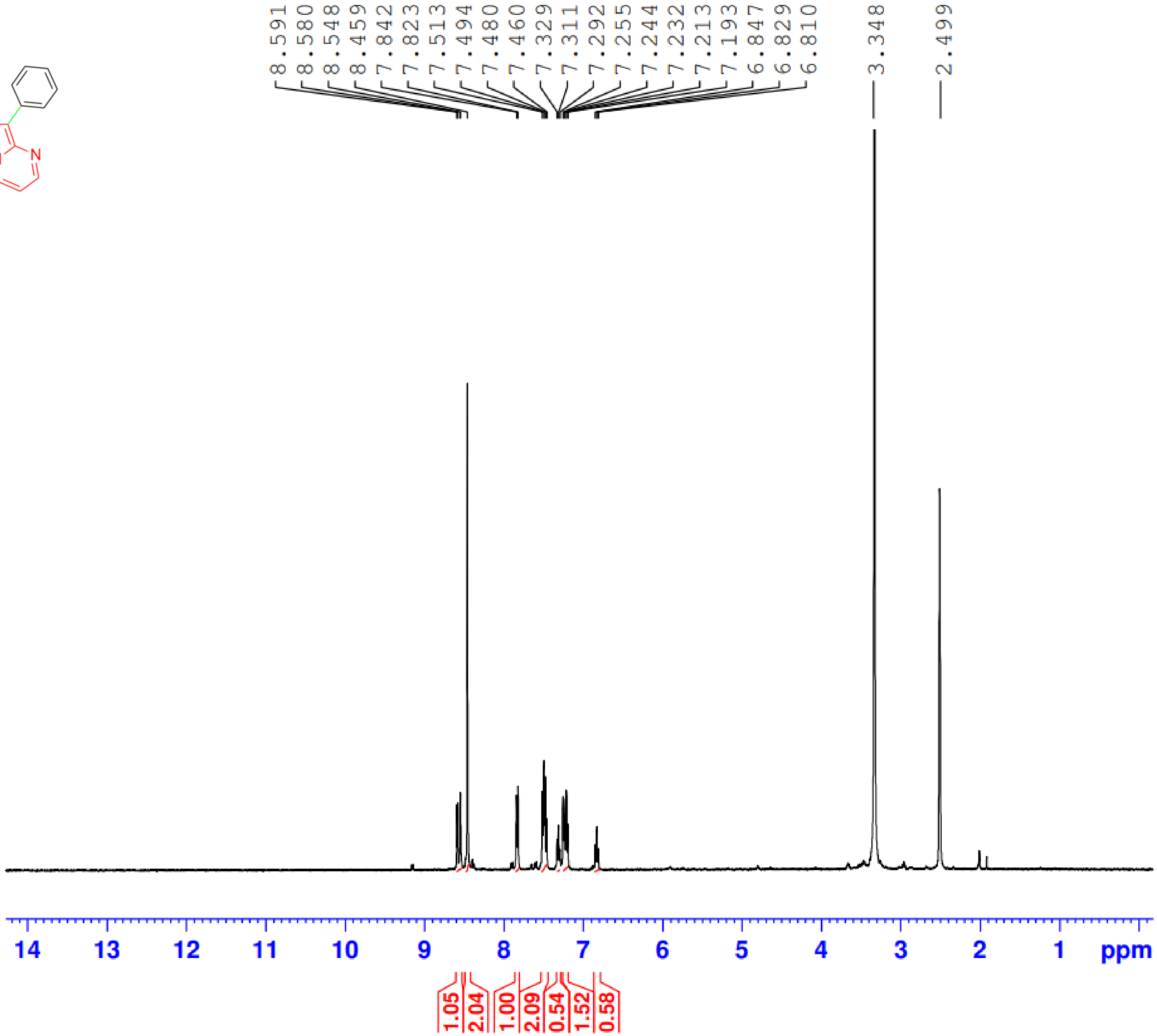

BRUKER

Current Data Parameters
NAME mohammed-attia-13h
EXPNO 1
PROCNO 1

F2 - Acquisition Parameters
Date_ 20210520
Time 14.37
INSTRUM spect
PROBHD 5 mm PABBO BB/
PULPROG zg30
TD 65536
SOLVENT DMSO
NS 24
DS 2
SWH 8012.820 Hz
FIDRES 0.122266 Hz
AQ 4.0894465 sec
RG 205.37
DW 62.400 usec
DE 6.50 usec
TE 300.0 K
D1 1.00000000 sec
TD0 1

======== CHANNEL f1 ========
SFO1 400.1524711 MHz
NUC1 1H
P1 12.00 usec
PLW1 18.00000000 W

F2 - Processing parameters
SI 65536
SF 400.1500000 MHz
WDW EM
SSB 0
LB 0.30 Hz
GB 0
PC 1.00

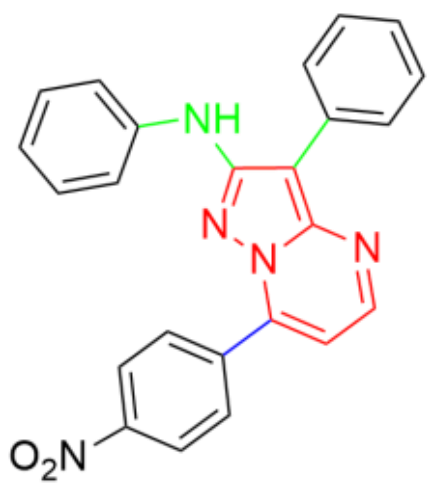

6g

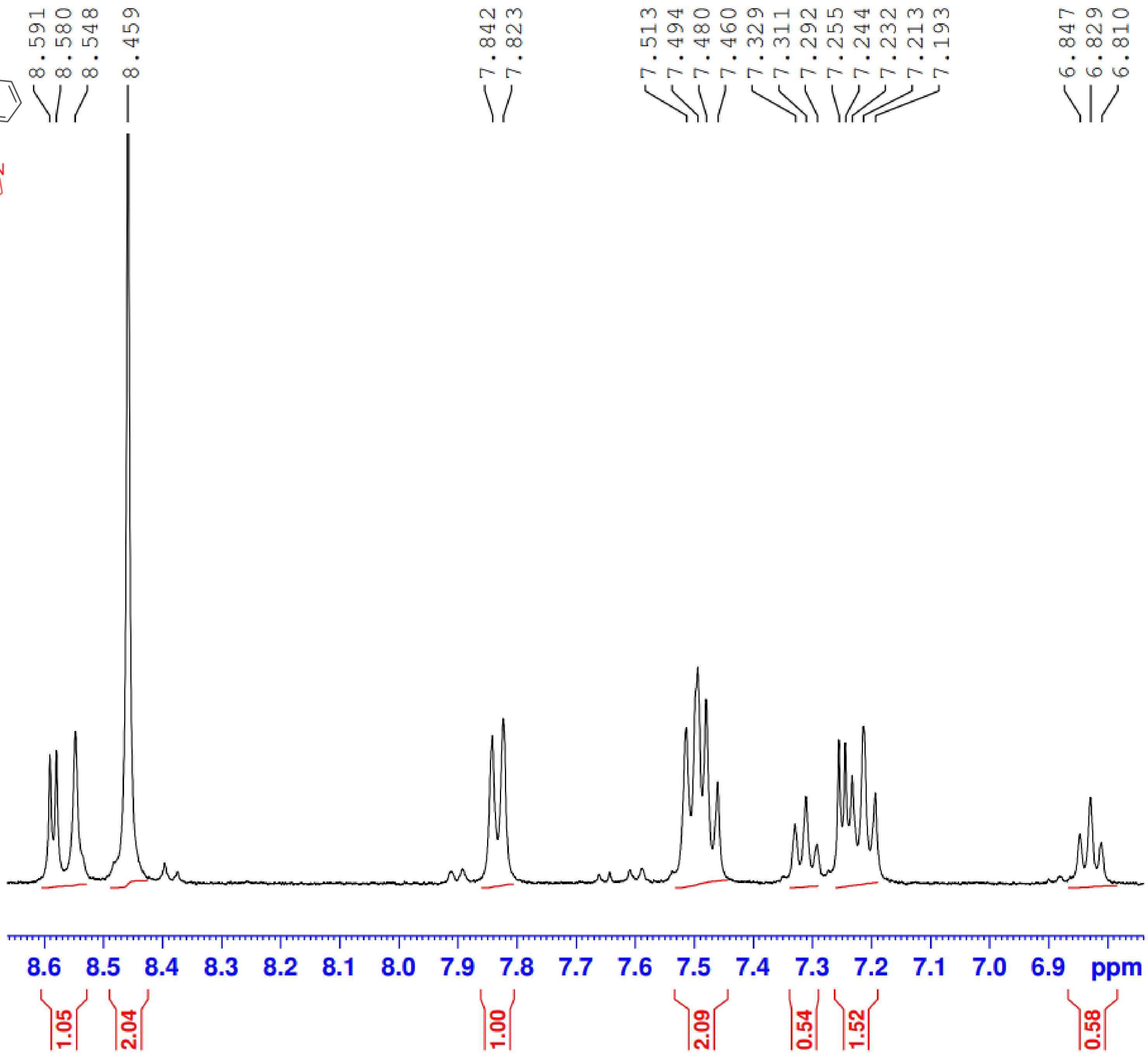


```
Current Data Parameters
NAME       mohammed-attia-13h
EXPNO                       1
PROCNO                      1

F2 - Acquisition Parameters
Date_                20210520
Time                    14.37
INSTRUM                 spect
PROBHD    5 mm PABBO BB/
PULPROG                  zg30
TD                      65536
SOLVENT                  DMSO
NS                         24
DS                          2
SWH                  8012.820 Hz
FIDRES               0.122266 Hz
AQ                  4.0894465 sec
RG                     205.37
DW                     62.400 usec
DE                       6.50 usec
TE                      300.0 K
D1                 1.00000000 sec
TD0                         1

======== CHANNEL f1 ========
SFO1              400.1524711 MHz
NUC1                       1H
P1                      12.00 usec
PLW1              18.00000000 W

F2 - Processing parameters
SI                      65536
SF                400.1500000 MHz
WDW                        EM
SSB        0
LB                       0.30 Hz
GB         0
PC                       1.00
```

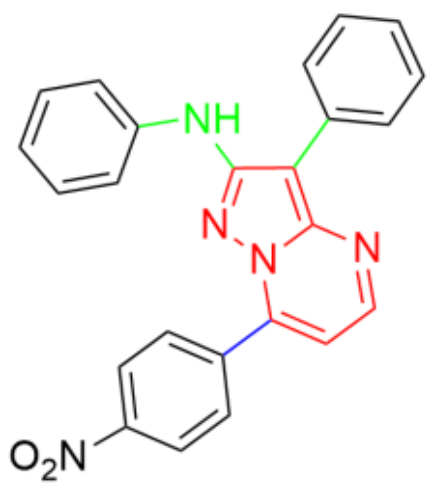

6g

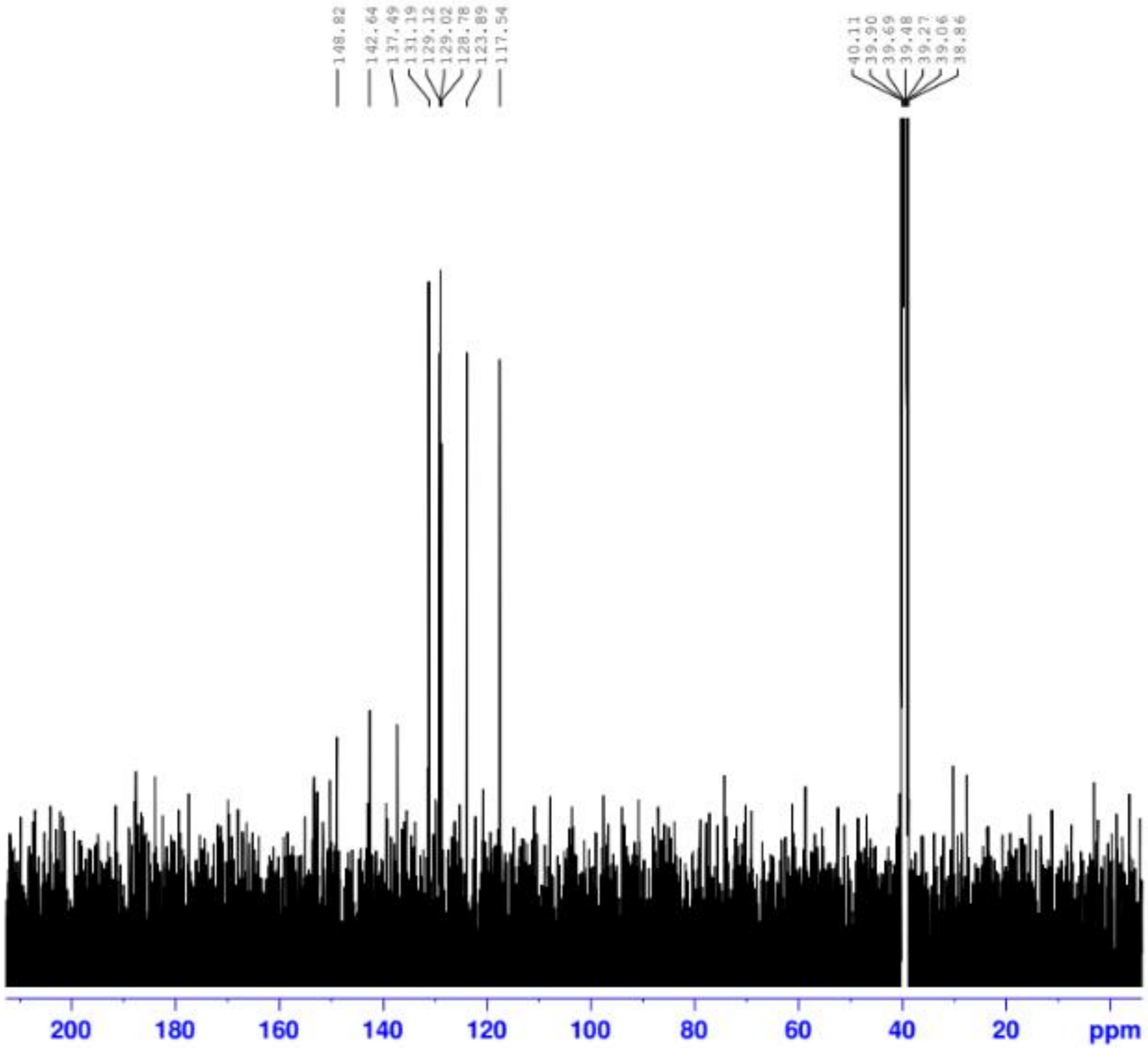

BRUKER

Current Data Parameters
NAME mohammed-attia-13h
EXPNO 3
PROCNO 1

F2 - Acquisition Parameters
Date_ 20210711
Time 17.51
INSTRUM spect
PROBHD 5 mm PABBO BB/
PULPROG zgpg30
TD 65536
SOLVENT DMSO
NS 1700
DS 4
SWH 24038.461 Hz
FIDRES 0.366798 Hz
AQ 1.3631488 sec
RG 205.37
DW 20.800 usec
DE 6.50 usec
TE 300.0 K
D1 2.00000000 sec
D11 0.03000000 sec
TD0 1

======== CHANNEL f1 ========
SFO1 100.6278588 MHz
NUC1 13C
P1 10.00 usec
PLW1 47.00000000 W

======== CHANNEL f2 ========
SFO2 400.1516006 MHz
NUC2 1H
CPDPRG[2 waltz16
PCPD2 90.00 usec
PLW2 18.00000000 W
PLW12 0.34722000 W
PLW13 0.28125000 W

F2 - Processing parameters
SI 32768
SF 100.6177975 MHz
WDW EM
SSB 0
LB 1.00 Hz
GB 0
PC 1.40

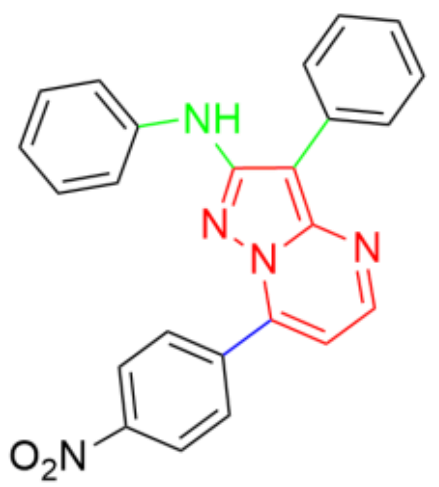

6g

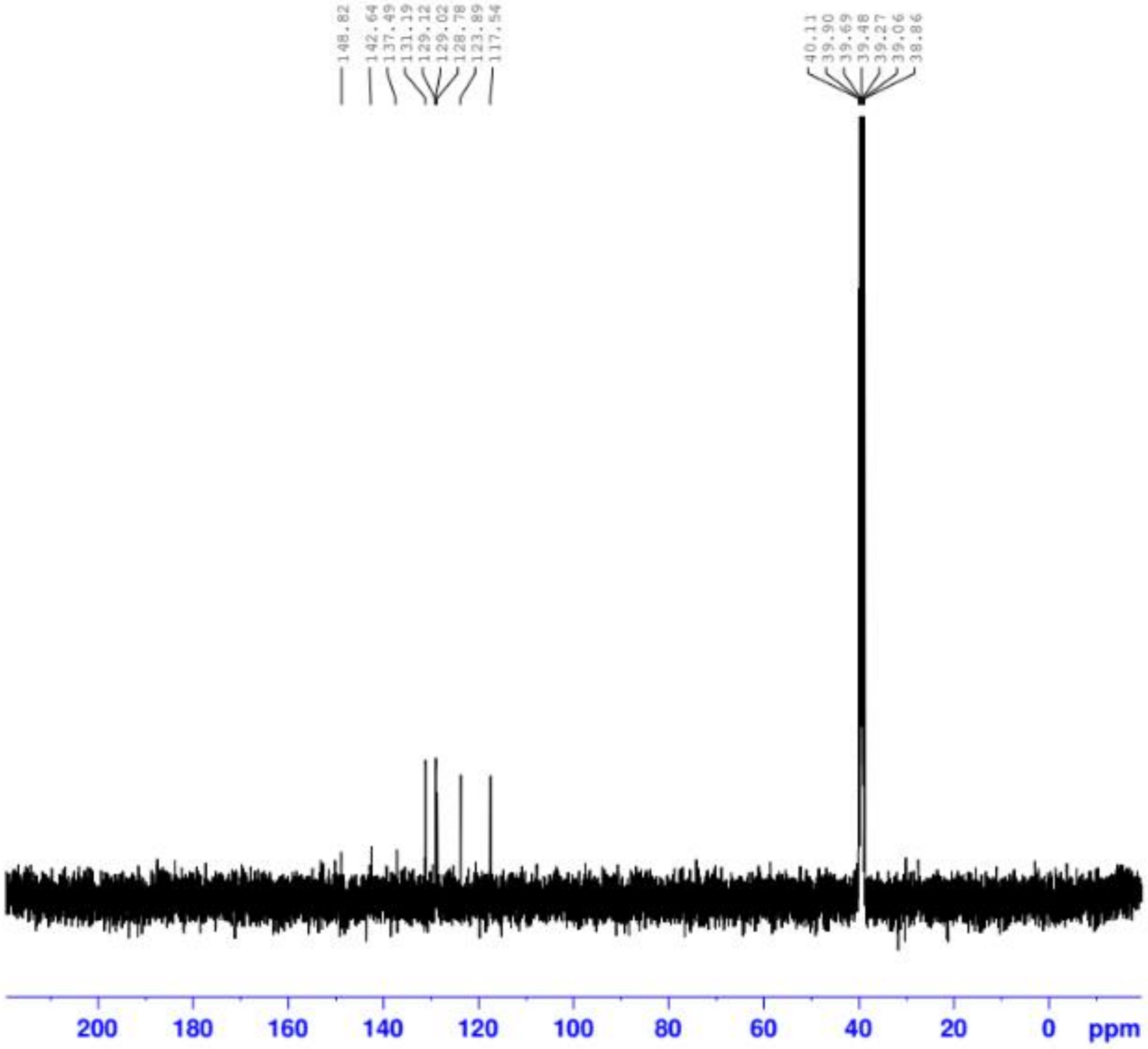

BRUKER

Current Data Parameters
NAME mohammed-attia-13h
EXPNO 3
PROCNO 1

F2 - Acquisition Parameters
Date_ 20210711
Time 17.51
INSTRUM spect
PROBHD 5 mm PABBO BB/
PULPROG zgpg30
TD 65536
SOLVENT DMSO
NS 1700
DS 4
SWH 24038.461 Hz
FIDRES 0.366798 Hz
AQ 1.3631488 sec
RG 205.37
DW 20.800 usec
DE 6.50 usec
TE 300.0 K
D1 2.00000000 sec
D11 0.03000000 sec
TD0 1

======== CHANNEL f1 ========
SFO1 100.6278588 MHz
NUC1 13C
P1 10.00 usec
PLW1 47.00000000 W

======== CHANNEL f2 ========
SFO2 400.1516006 MHz
NUC2 1H
CPDPRG[2 waltz16
PCPD2 90.00 usec
PLW2 18.00000000 W
PLW12 0.34722000 W
PLW13 0.28125000 W

F2 - Processing parameters
SI 32768
SF 100.6177975 MHz
WDW EM
SSB 0
LB 1.00 Hz
GB 0
PC 1.40

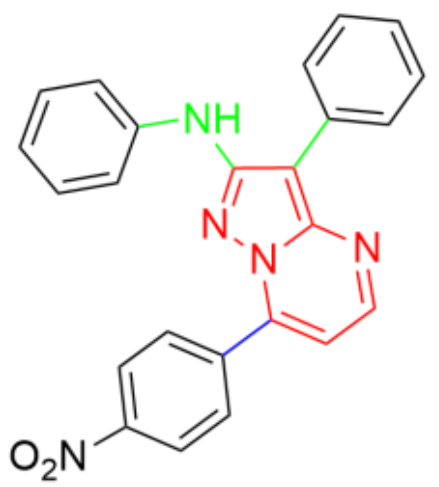

6g

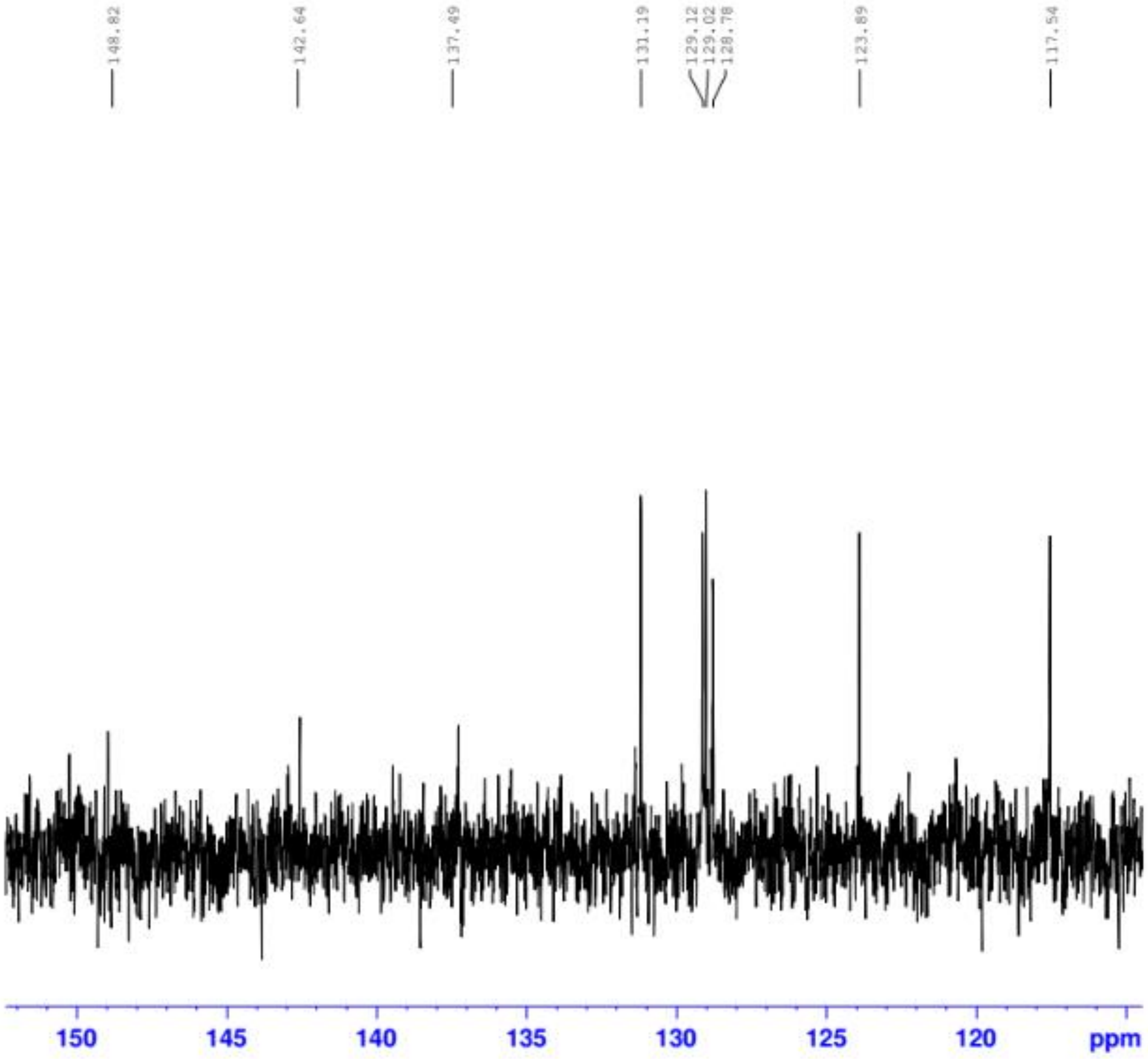

Current Data Parameters
NAME mohammed-attia-13h
EXPNO 3
PROCNO 1

F2 - Acquisition Parameters
Date_ 20210711
Time 17.51
INSTRUM spect
PROBHD 5 mm PABBO BB/
PULPROG zgpg30
TD 65536
SOLVENT DMSO
NS 1700
DS 4
SWH 24038.461 Hz
FIDRES 0.366798 Hz
AQ 1.3631488 sec
RG 205.37
DW 20.800 usec
DE 6.50 usec
TE 300.0 K
D1 2.00000000 sec
D11 0.03000000 sec
TD0 1

======== CHANNEL f1 ========
SFO1 100.6278588 MHz
NUC1 13C
P1 10.00 usec
PLW1 47.00000000 W

======== CHANNEL f2 ========
SFO2 400.1516006 MHz
NUC2 1H
CPDPRG[2 waltz16
PCPD2 90.00 usec
PLW2 18.00000000 W
PLW12 0.34722000 W
PLW13 0.28125000 W

F2 - Processing parameters
SI 32768
SF 100.6177975 MHz
WDW EM
SSB 0
LB 1.00 Hz
GB 0
PC 1.40

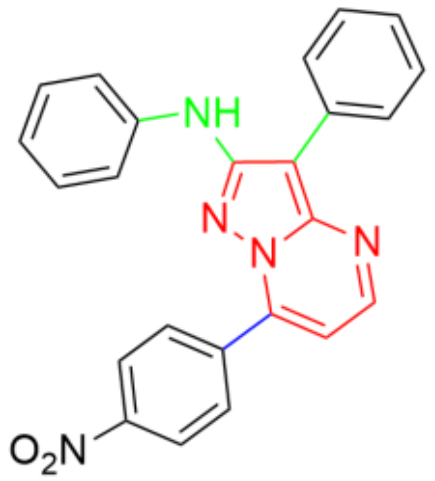
NH
N
N
N
O_2N

6g

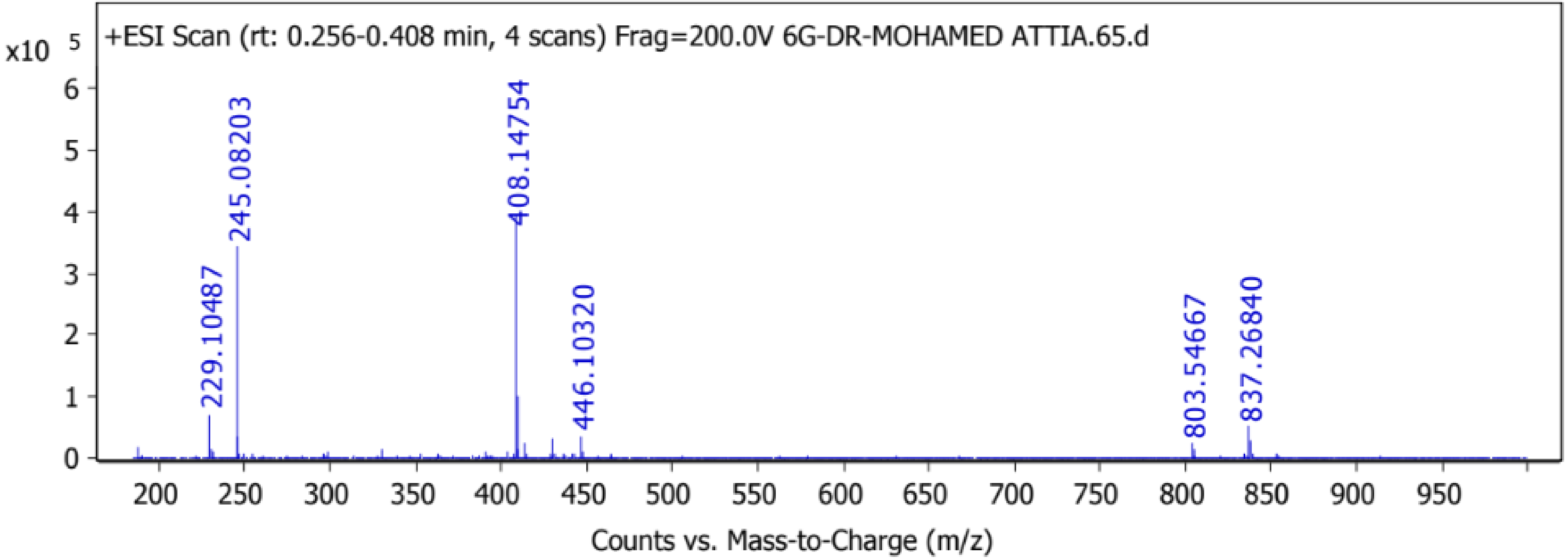
+ESI Scan (rt: 0.256-0.408 min, 4 scans) Frag=200.0V 6G-DR-MOHAMED ATTIA.65.d
x10 5
229.10487
245.08203
408.14754
446.10320
803.54667
837.26840
Counts vs. Mass-to-Charge (m/z)

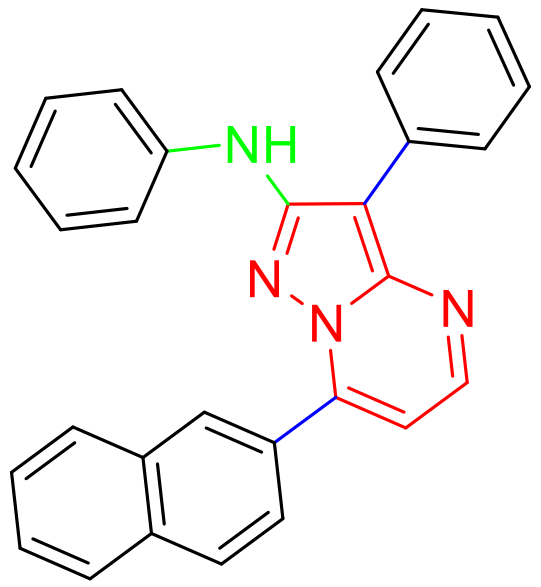

6h

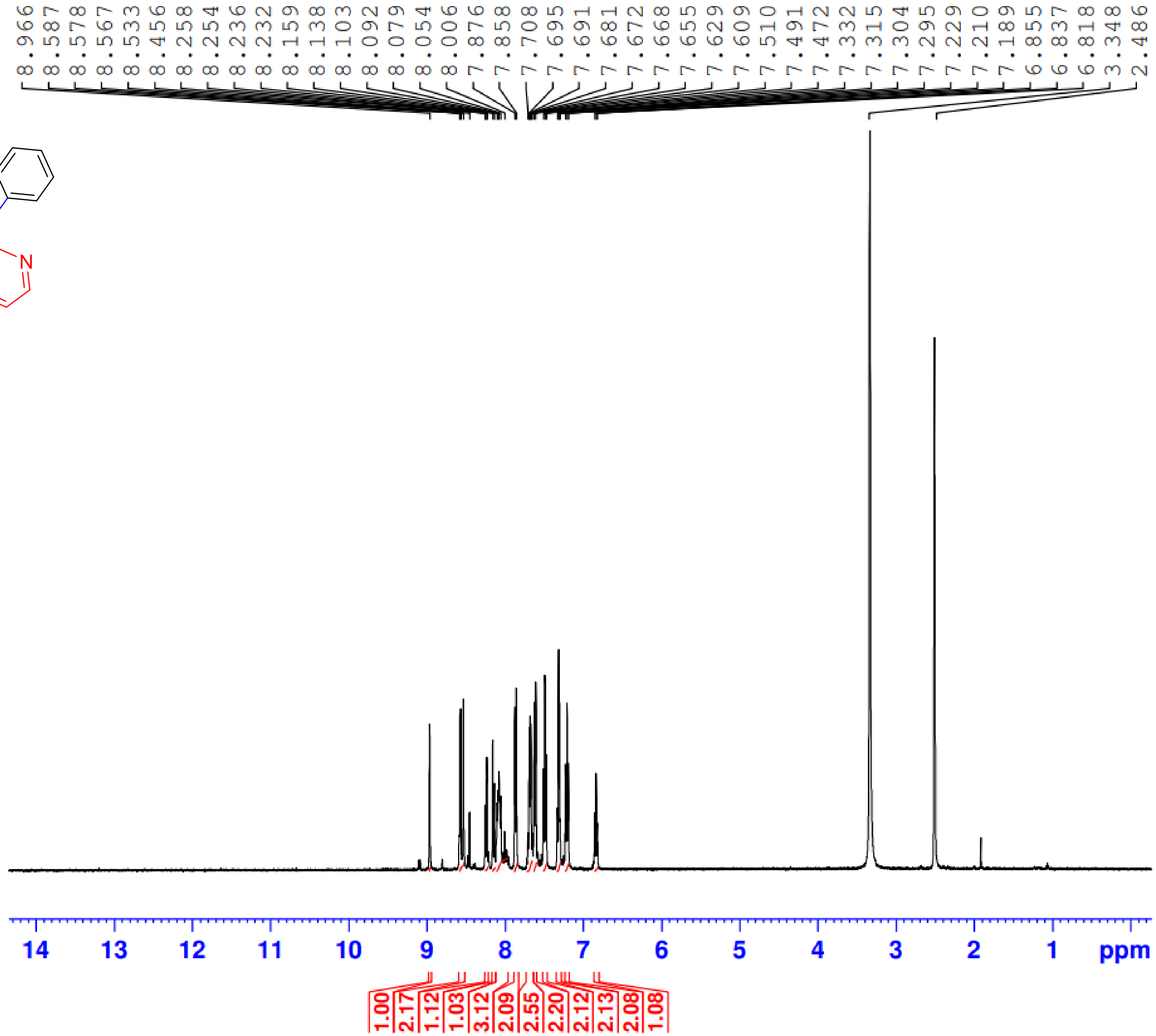

Current Data Parameters
NAME mohammed-attia-13i
EXPNO 1
PROCNO 1

F2 - Acquisition Parameters
Date_ 20210520
Time 14.15
INSTRUM spect
PROBHD 5 mm PABBO BB/
PULPROG zg30
TD 65536
SOLVENT DMSO
NS 20
DS 2
SWH 8012.820 Hz
FIDRES 0.122266 Hz
AQ 4.0894465 sec
RG 205.37
DW 62.400 usec
DE 6.50 usec
TE 300.0 K
D1 1.00000000 sec
TD0 1

======== CHANNEL f1 ========
SFO1 400.1524711 MHz
NUC1 1H
P1 12.00 usec
PLW1 18.00000000 W

F2 - Processing parameters
SI 65536
SF 400.1500000 MHz
WDW EM
SSB 0
LB 0.30 Hz
GB 0
PC 1.00

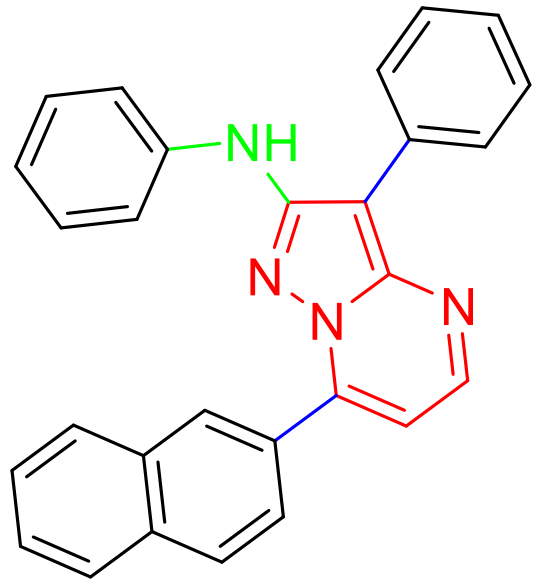

6h

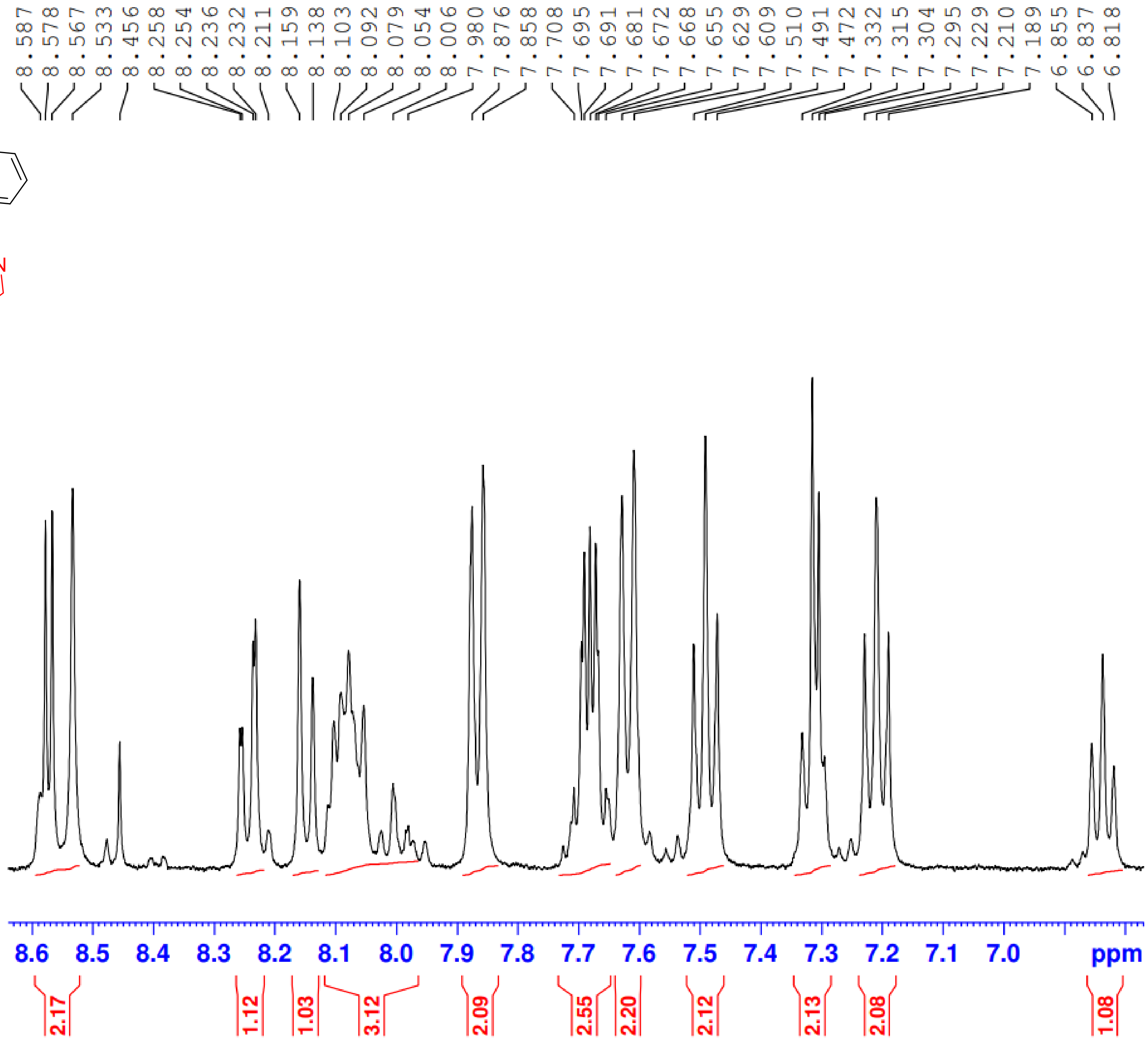


```
Current Data Parameters
NAME        mohammed-attia-13i
EXPNO                    1
PROCNO                   1

F2 - Acquisition Parameters
Date_             20210520
Time                 14.15
INSTRUM              spect
PROBHD    5 mm PABBO BB/
PULPROG               zg30
TD                   65536
SOLVENT               DMSO
NS                      20
DS                       2
SWH           8012.820 Hz
FIDRES        0.122266 Hz
AQ           4.0894465 sec
RG                  205.37
DW              62.400 usec
DE               6.50 usec
TE               300.0 K
D1          1.00000000 sec
TD0                      1

======== CHANNEL f1 ========
SFO1       400.1524711 MHz
NUC1                    1H
P1               12.00 usec
PLW1       18.00000000 W

F2 - Processing parameters
SI                   65536
SF         400.1500000 MHz
WDW                     EM
SSB       0
LB                0.30 Hz
GB        0
PC                    1.00
```

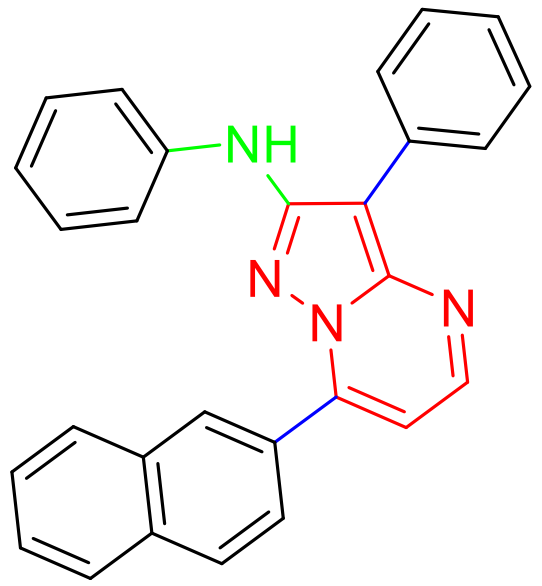

6h

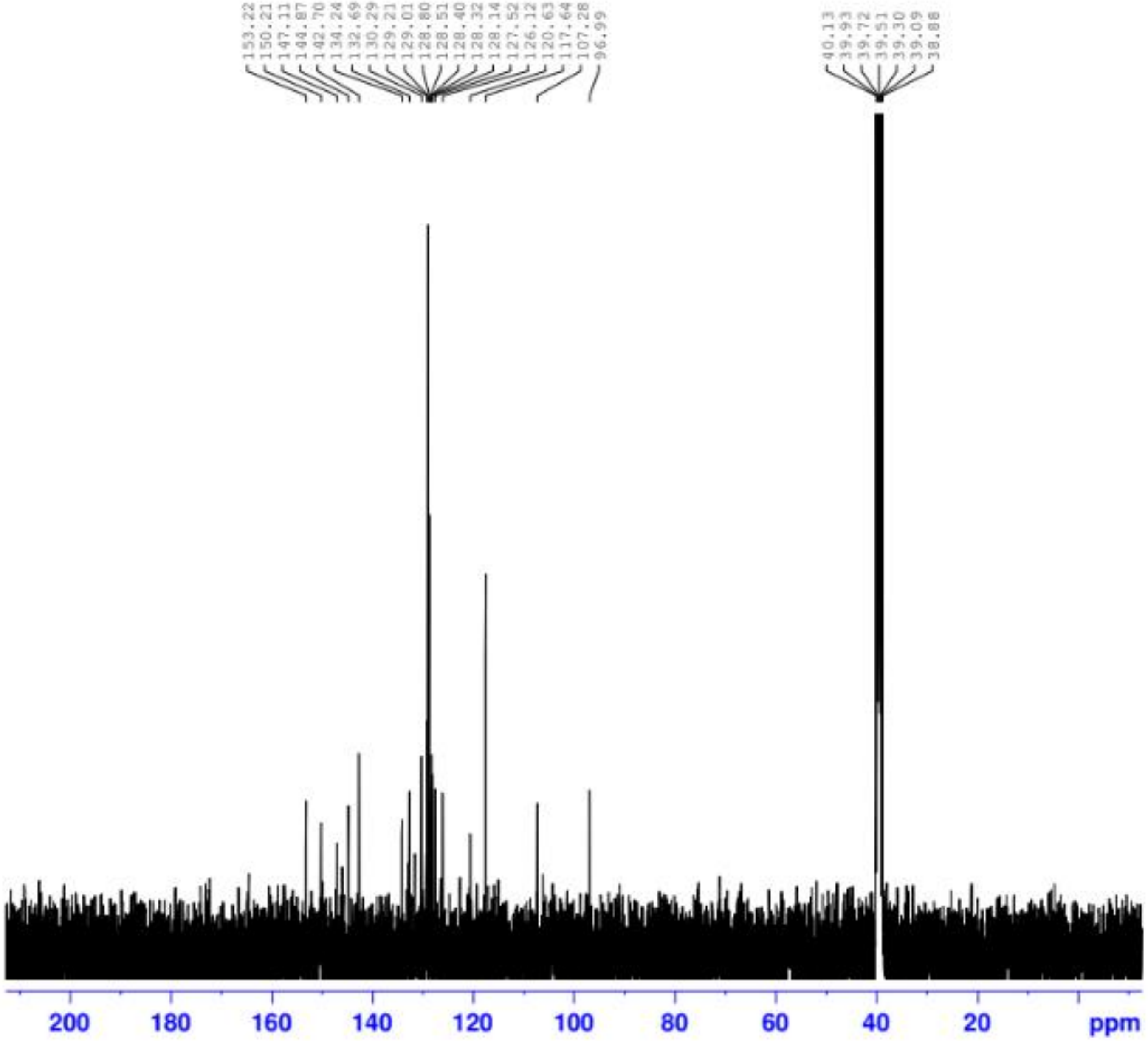

BRUKER

Current Data Parameters

NAME	mohammed-attia-13i
EXPNO	2
PROCNO	1

F2 - Acquisition Parameters

Date_	20210714
Time	10.58
INSTRUM	spect
PROBHD	5 mm PABBO BB/
PULPROG	zgpg30
TD	65536
SOLVENT	DMSO
NS	2060
DS	4
SWH	24038.461 Hz
FIDRES	0.366798 Hz
AQ	1.3631488 sec
RG	205.37
DW	20.800 usec
DE	6.50 usec
TE	300.0 K
D1	2.00000000 sec
D11	0.03000000 sec
TD0	1

======== CHANNEL f1 ========

SFO1	100.6278588 MHz
NUC1	13C
P1	10.00 usec
PLW1	47.00000000 W

======== CHANNEL f2 ========

SFO2	400.1516006 MHz
NUC2	1H
CPDPRG[2	waltz16
PCPD2	90.00 usec
PLW2	18.00000000 W
PLW12	0.34722000 W
PLW13	0.28125000 W

F2 - Processing parameters

SI	32768
SF	100.6177975 MHz
WDW	EM
SSB	0
LB	1.00 Hz
GB	0
PC	1.40

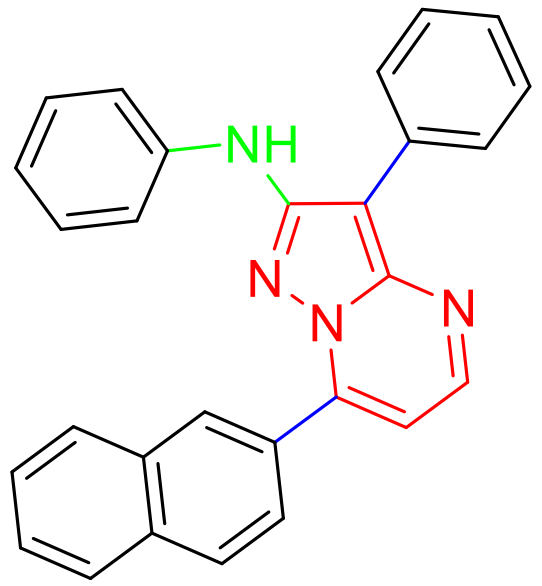

6h

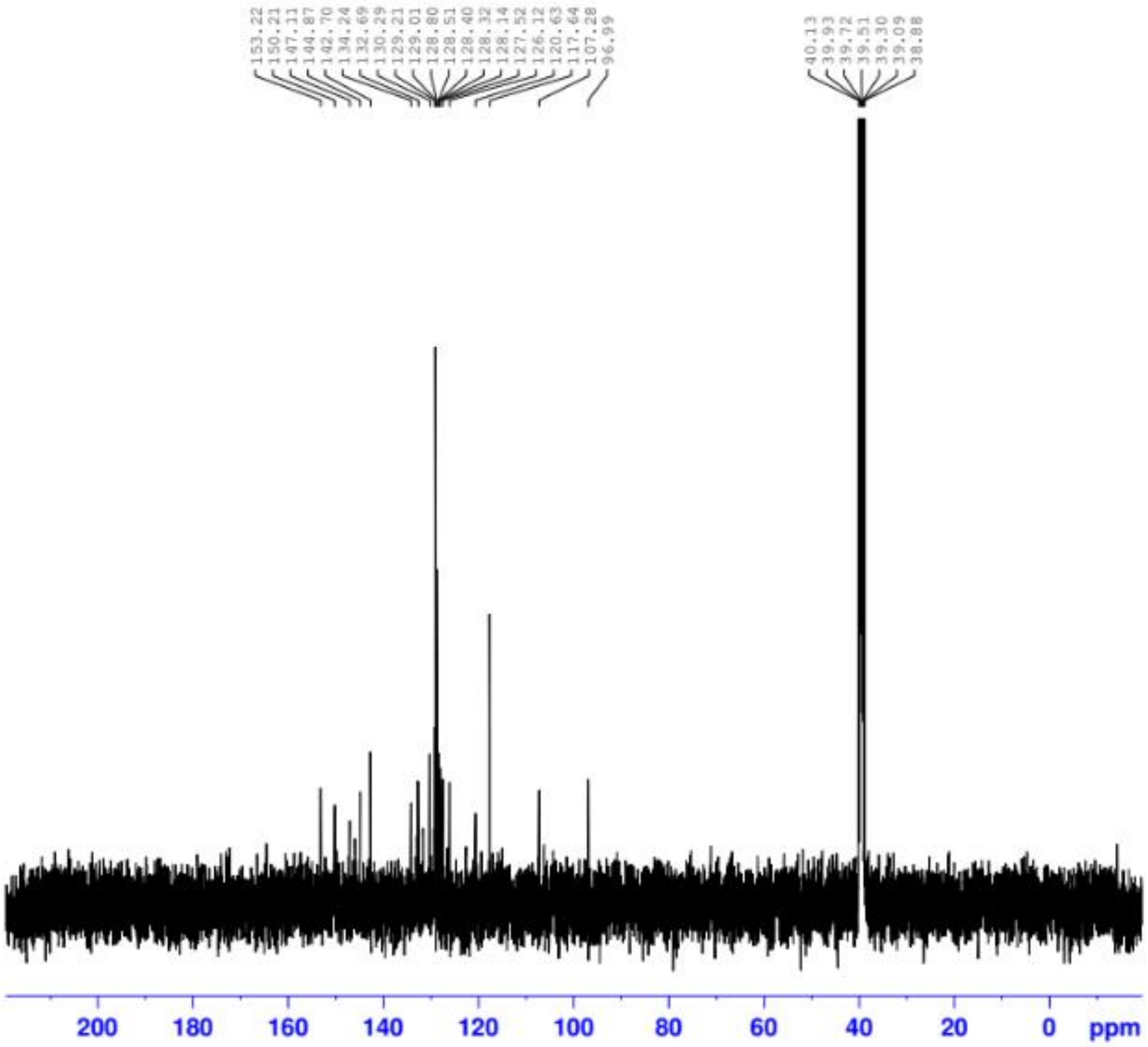

BRUKER

Current Data Parameters
NAME mohammed-attia-13i
EXPNO 2
PROCNO 1

F2 - Acquisition Parameters
Date_ 20210714
Time 10.58
INSTRUM spect
PROBHD 5 mm PABBO BB/
PULPROG zgpg30
TD 65536
SOLVENT DMSO
NS 2060
DS 4
SWH 24038.461 Hz
FIDRES 0.366798 Hz
AQ 1.3631488 sec
RG 205.37
DW 20.800 usec
DE 6.50 usec
TE 300.0 K
D1 2.00000000 sec
D11 0.03000000 sec
TD0 1

======== CHANNEL f1 ========
SFO1 100.6278588 MHz
NUC1 13C
P1 10.00 usec
PLW1 47.00000000 W

======== CHANNEL f2 ========
SFO2 400.1516006 MHz
NUC2 1H
CPDPRG[2 waltz16
PCPD2 90.00 usec
PLW2 18.00000000 W
PLW12 0.34722000 W
PLW13 0.28125000 W

F2 - Processing parameters
SI 32768
SF 100.6177975 MHz
WDW EM
SSB 0
LB 1.00 Hz
GB 0
PC 1.40

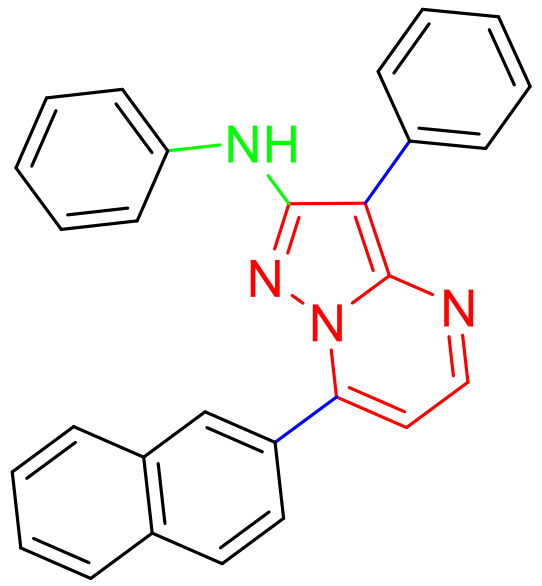

6h

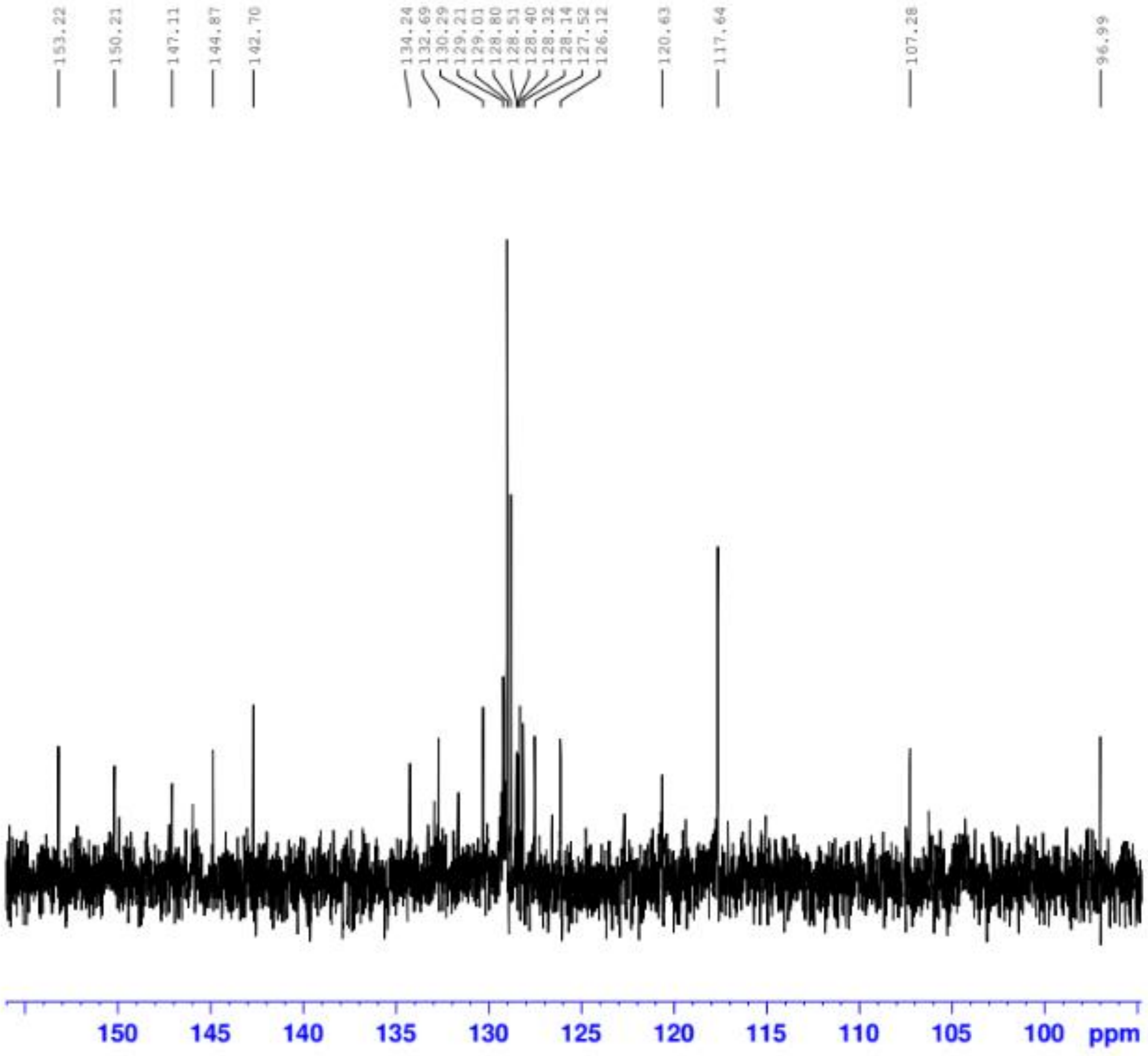

BRUKER

Current Data Parameters
NAME mohammed-attia-13i
EXPNO 2
PROCNO 1

F2 - Acquisition Parameters
Date_ 20210714
Time 10.58
INSTRUM spect
PROBHD 5 mm PABBO BB/
PULPROG zgpg30
TD 65536
SOLVENT DMSO
NS 2060
DS 4
SWH 24038.461 Hz
FIDRES 0.366798 Hz
AQ 1.3631488 sec
RG 205.37
DW 20.800 usec
DE 6.50 usec
TE 300.0 K
D1 2.00000000 sec
D11 0.03000000 sec
TD0 1

======== CHANNEL f1 ========
SFO1 100.6278588 MHz
NUC1 13C
P1 10.00 usec
PLW1 47.00000000 W

======== CHANNEL f2 ========
SFO2 400.1516006 MHz
NUC2 1H
CPDPRG[2 waltz16
PCPD2 90.00 usec
PLW2 18.00000000 W
PLW12 0.34722000 W
PLW13 0.28125000 W

F2 - Processing parameters
SI 32768
SF 100.6177975 MHz
WDW EM
SSB 0
LB 1.00 Hz
GB 0
PC 1.40

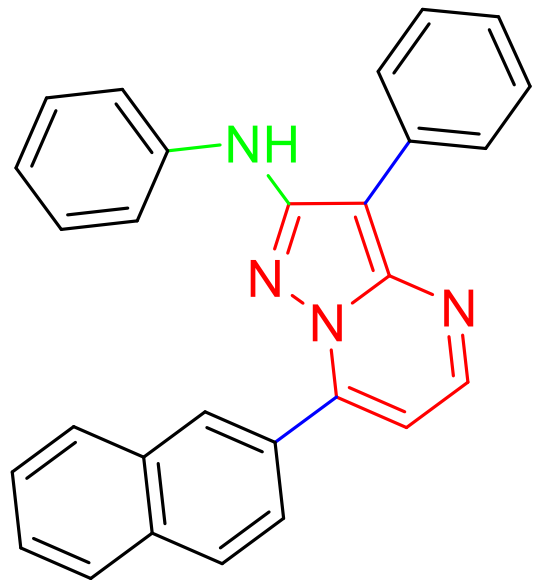
NH
N
N
N

6h

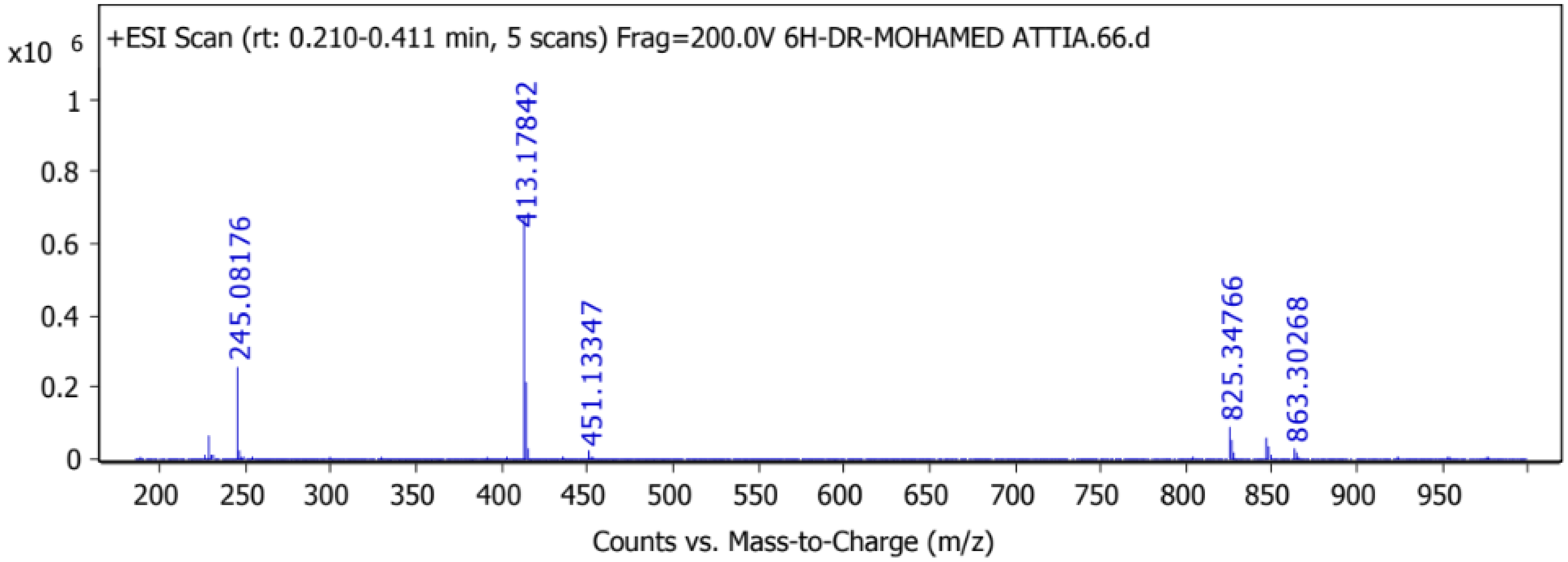
+ESI Scan (rt: 0.210-0.411 min, 5 scans) Frag=200.0V 6H-DR-MOHAMED ATTIA.66.d
x10 6
1
0.8
0.6
0.4
0.2
0
245.08176
413.17842
451.13347
825.34766
863.30268
200
250
300
350
400
450
500
550
600
650
700
750
800
850
900
950
Counts vs. Mass-to-Charge (m/z)

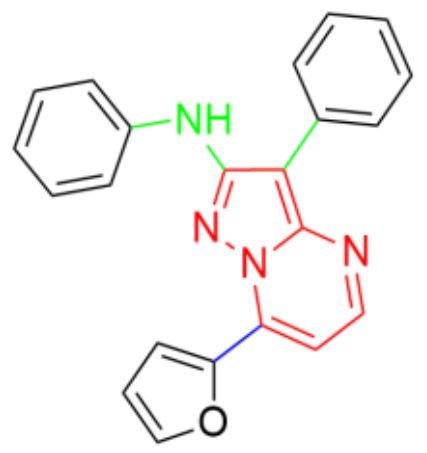

6i

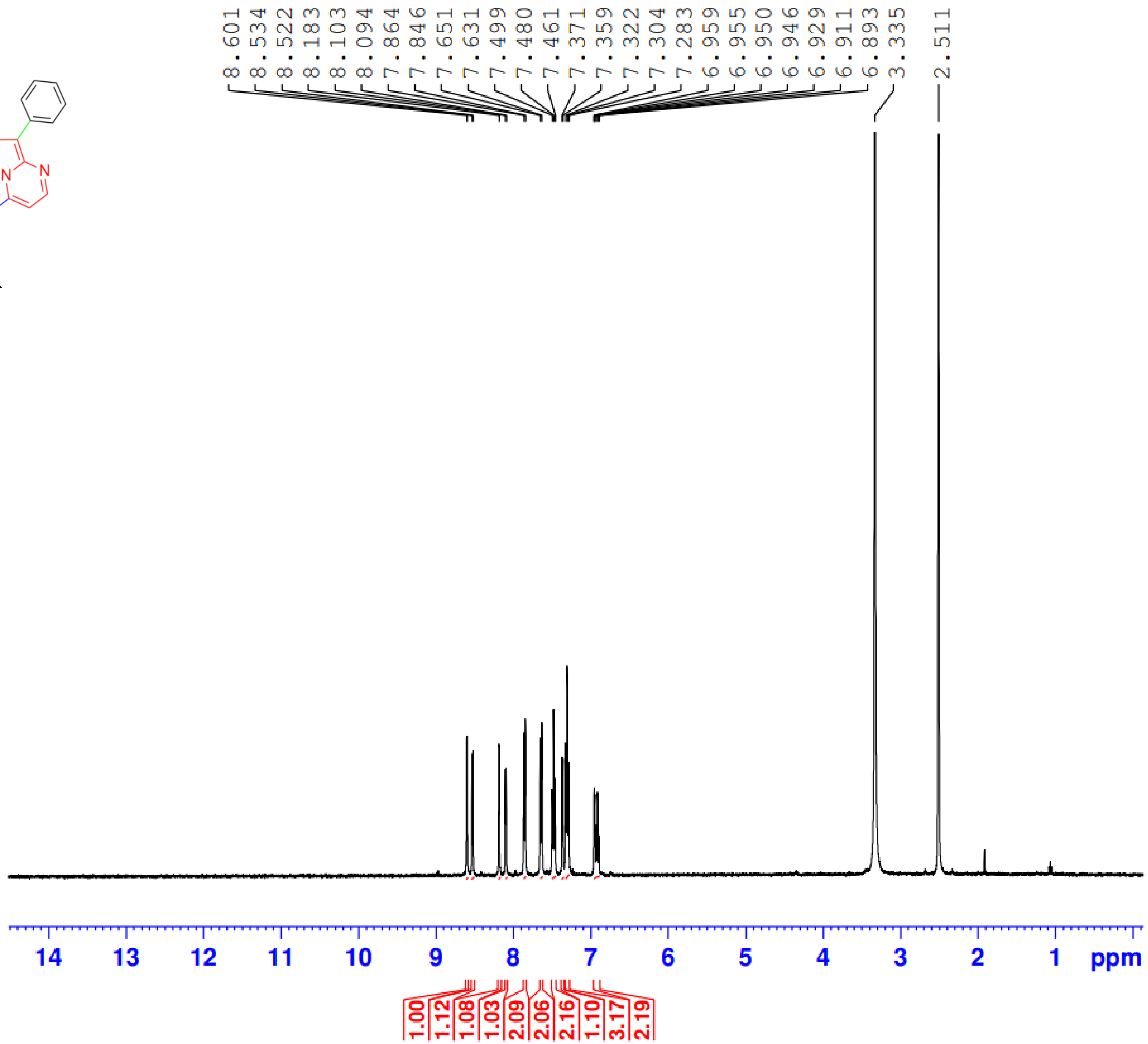

Current Data Parameters
NAME mohammed-attia-13j
EXPNO 1
PROCNO 1

F2 - Acquisition Parameters
Date_ 20210520
Time 14.25
INSTRUM spect
PROBHD 5 mm PABBO BB/
PULPROG zg30
TD 65536
SOLVENT DMSO
NS 19
DS 2
SWH 8012.820 Hz
FIDRES 0.122266 Hz
AQ 4.0894465 sec
RG 205.37
DW 62.400 usec
DE 6.50 usec
TE 300.0 K
D1 1.00000000 sec
TD0 1

======== CHANNEL f1 ========
SFO1 400.1524711 MHz
NUC1 1H
P1 12.00 usec
PLW1 18.00000000 W

F2 - Processing parameters
SI 65536
SF 400.1500000 MHz
WDW EM
SSB 0
LB 0.30 Hz
GB 0
PC 1.00

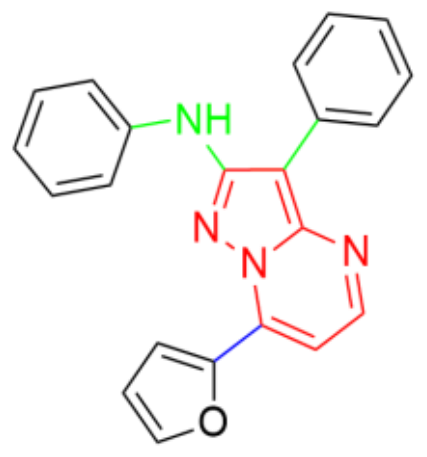

6i

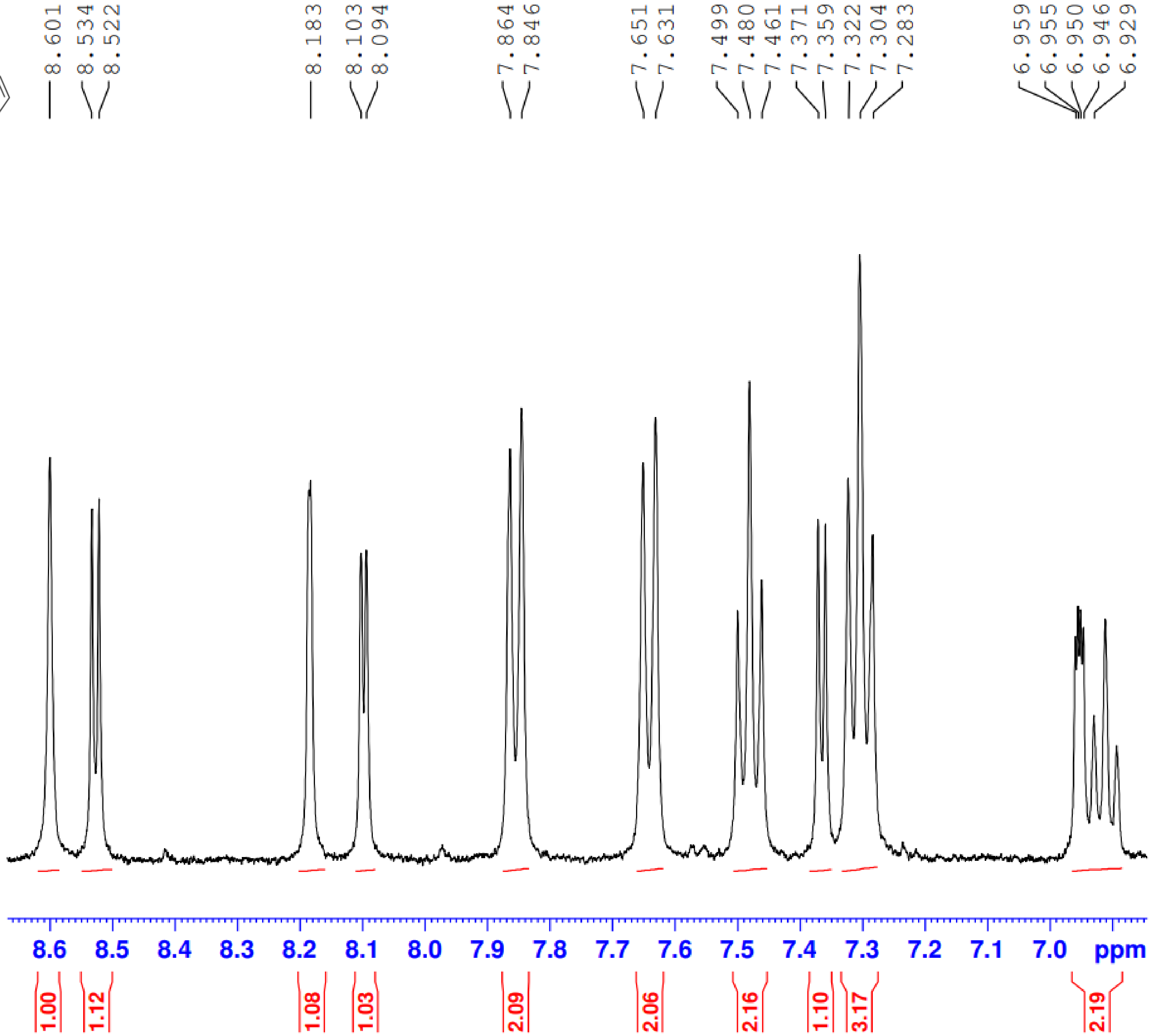

Current Data Parameters
NAME mohammed-attia-13j
EXPNO 1
PROCNO 1

F2 - Acquisition Parameters
Date_ 20210520
Time 14.25
INSTRUM spect
PROBHD 5 mm PABBO BB/
PULPROG zg30
TD 65536
SOLVENT DMSO
NS 19
DS 2
SWH 8012.820 Hz
FIDRES 0.122266 Hz
AQ 4.0894465 sec
RG 205.37
DW 62.400 usec
DE 6.50 usec
TE 300.0 K
D1 1.00000000 sec
TD0 1

======== CHANNEL f1 ========
SFO1 400.1524711 MHz
NUC1 1H
P1 12.00 usec
PLW1 18.00000000 W

F2 - Processing parameters
SI 65536
SF 400.1500000 MHz
WDW EM
SSB 0
LB 0.30 Hz
GB 0
PC 1.00

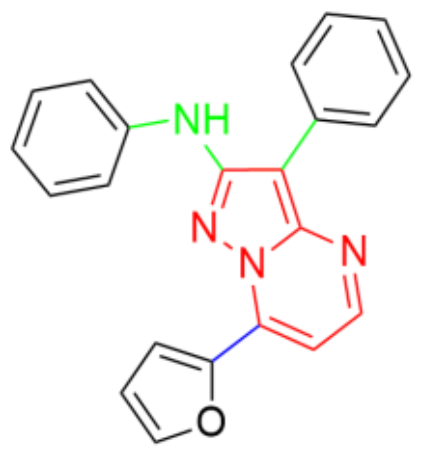

6i

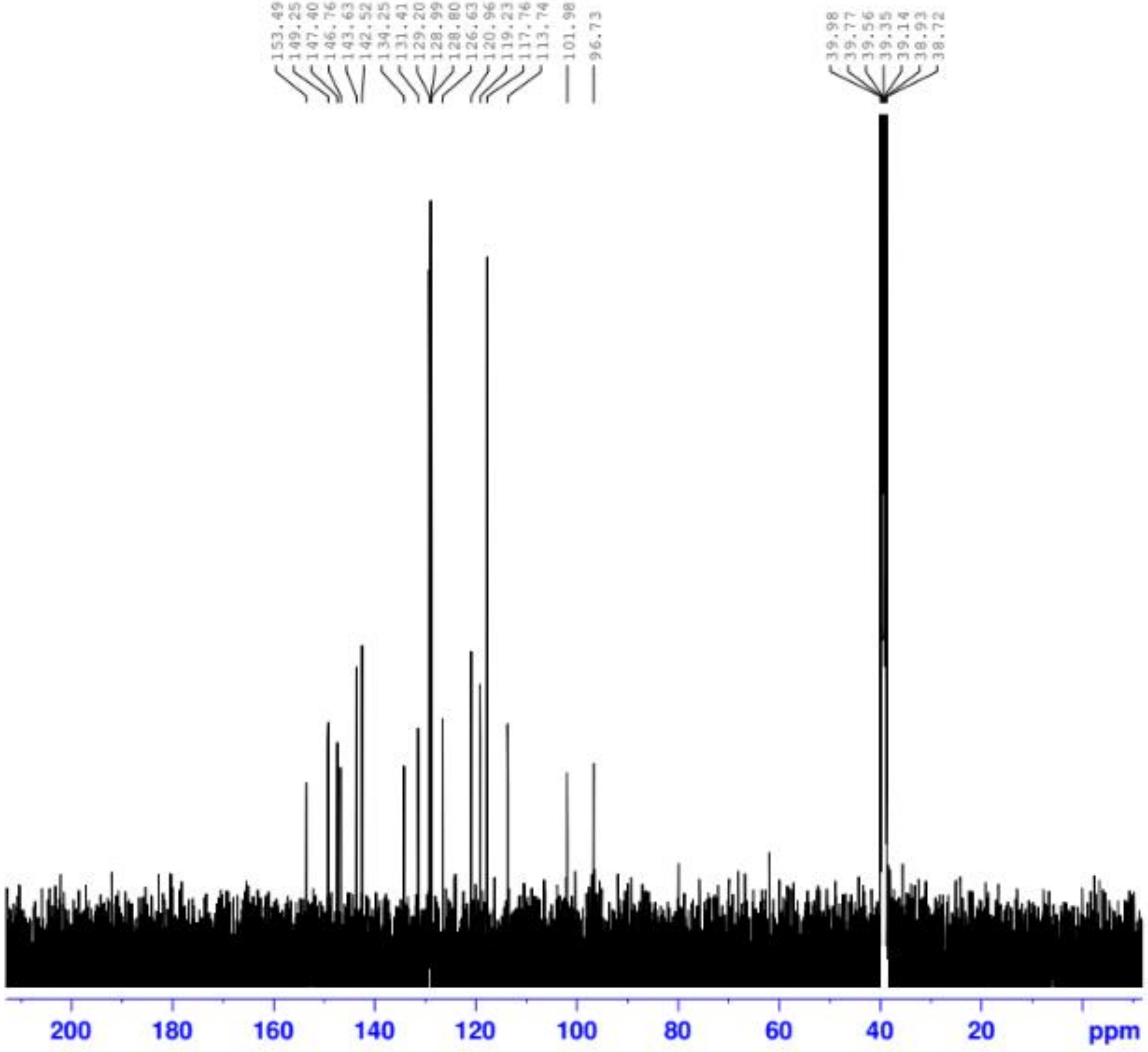

BRUKER

Current Data Parameters
NAME mohammed-attia-13j
EXPNO 2
PROCNO 1

F2 - Acquisition Parameters
Date_ 20210825
Time 9.37
INSTRUM spect
PROBHD 5 mm PABBO BB/
PULPROG zgpg30
TD 65536
SOLVENT DMSO
NS 1024
DS 4
SWH 24038.461 Hz
FIDRES 0.366798 Hz
AQ 1.3631488 sec
RG 205.37
DW 20.800 usec
DE 6.50 usec
TE 300.0 K
D1 2.00000000 sec
D11 0.03000000 sec
TD0 1

======== CHANNEL f1 ========
SFO1 100.6278588 MHz
NUC1 13C
P1 10.00 usec
PLW1 47.00000000 W

======== CHANNEL f2 ========
SFO2 400.1516006 MHz
NUC2 1H
CPDPRG[2 waltz16
PCPD2 90.00 usec
PLW2 18.00000000 W
PLW12 0.34722000 W
PLW13 0.28125000 W

F2 - Processing parameters
SI 32768
SF 100.6177975 MHz
WDW EM
SSB 0
LB 1.00 Hz
GB 0
PC 1.40

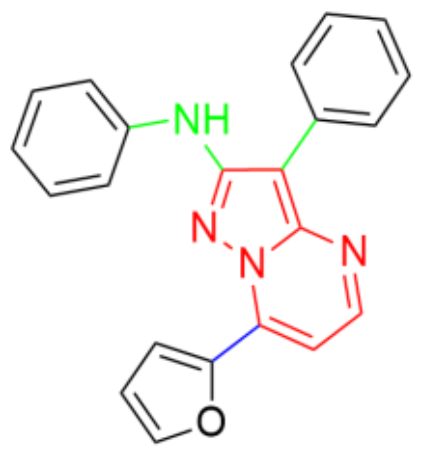

6i

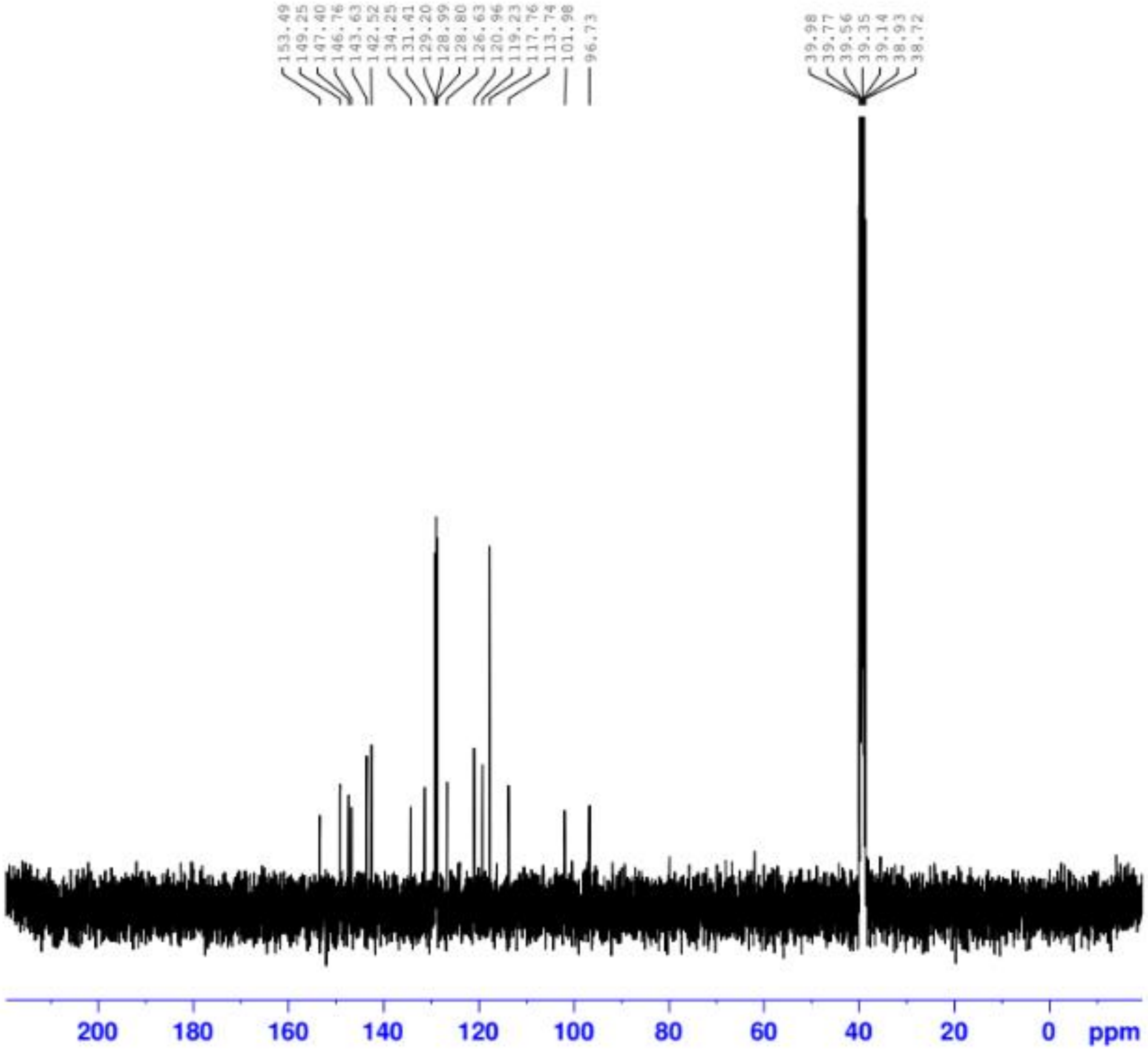

Current Data Parameters
NAME mohammed-attia-13j
EXPNO 2
PROCNO 1

F2 - Acquisition Parameters
Date_ 20210825
Time 9.37
INSTRUM spect
PROBHD 5 mm PABBO BB/
PULPROG zgpg30
TD 65536
SOLVENT DMSO
NS 1024
DS 4
SWH 24038.461 Hz
FIDRES 0.366798 Hz
AQ 1.3631488 sec
RG 205.37
DW 20.800 usec
DE 6.50 usec
TE 300.0 K
D1 2.00000000 sec
D11 0.03000000 sec
TD0 1

======== CHANNEL f1 ========
SFO1 100.6278588 MHz
NUC1 13C
P1 10.00 usec
PLW1 47.00000000 W

======== CHANNEL f2 ========
SFO2 400.1516006 MHz
NUC2 1H
CPDPRG[2 waltz16
PCPD2 90.00 usec
PLW2 18.00000000 W
PLW12 0.34722000 W
PLW13 0.28125000 W

F2 - Processing parameters
SI 32768
SF 100.6177975 MHz
WDW EM
SSB 0
LB 1.00 Hz
GB 0
PC 1.40

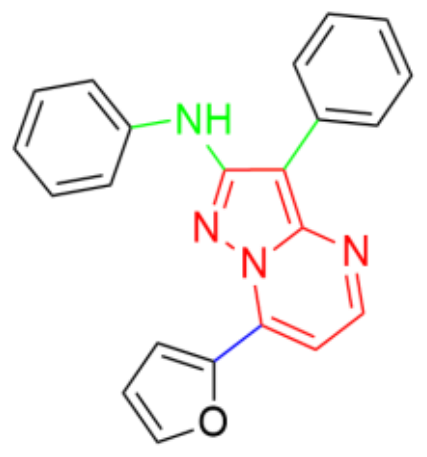

6i

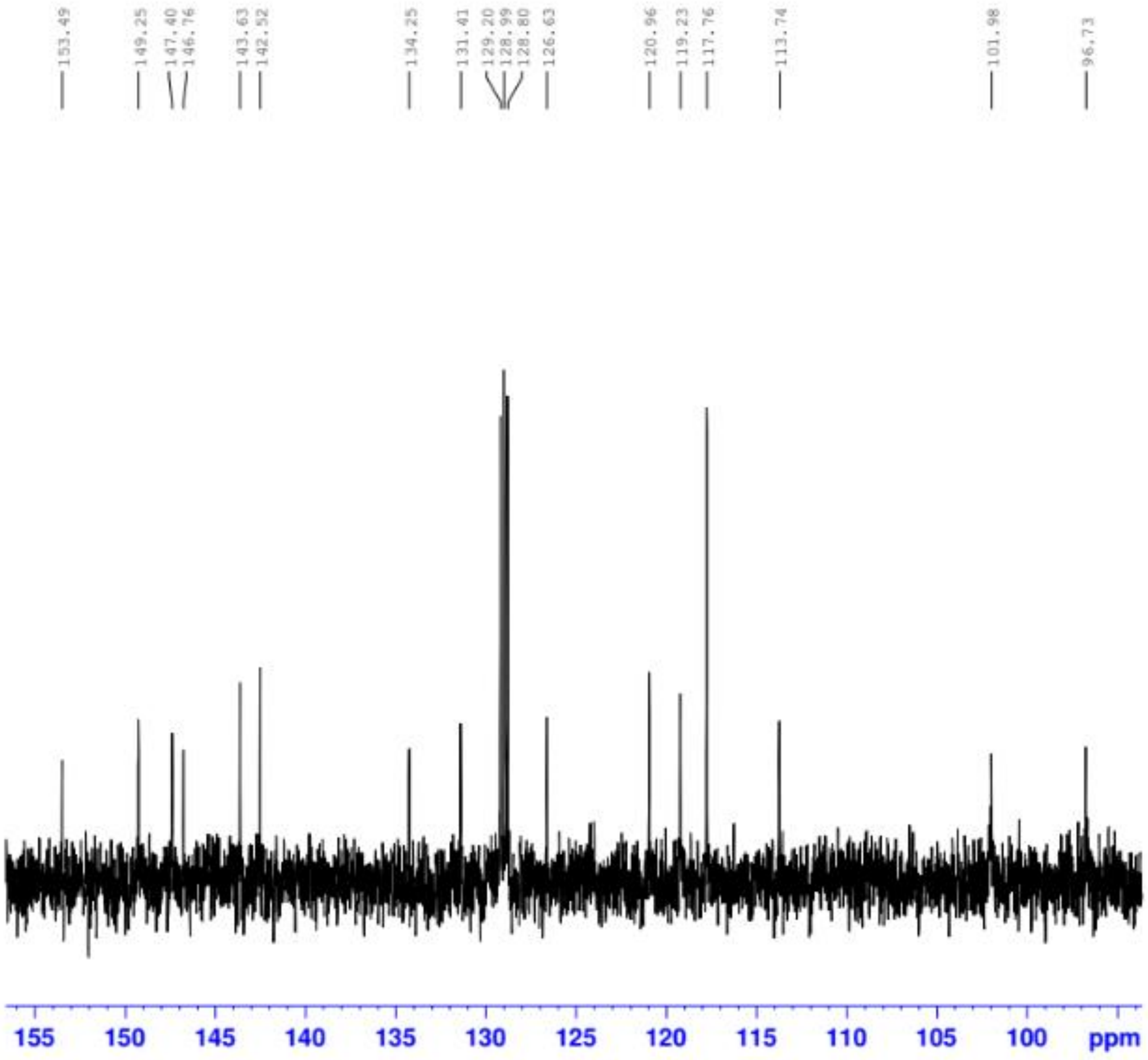

BRUKER

```
Current Data Parameters
NAME        mohammed-attia-13j
EXPNO                        2
PROCNO                       1

F2 - Acquisition Parameters
Date_               20210825
Time                    9.37
INSTRUM                spect
PROBHD    5 mm PABBO BB/
PULPROG              zgpg30
TD                    65536
SOLVENT                DMSO
NS                     1024
DS                        4
SWH               24038.461 Hz
FIDRES             0.366798 Hz
AQ                1.3631488 sec
RG                   205.37
DW                   20.800 usec
DE                     6.50 usec
TE                    300.0 K
D1               2.00000000 sec
D11              0.03000000 sec
TD0                       1

======== CHANNEL f1 ========
SFO1          100.6278588 MHz
NUC1                  13C
P1                  10.00 usec
PLW1          47.00000000 W

======== CHANNEL f2 ========
SFO2          400.1516006 MHz
NUC2                   1H
CPDPRG[2           waltz16
PCPD2               90.00 usec
PLW2          18.00000000 W
PLW12          0.34722000 W
PLW13          0.28125000 W

F2 - Processing parameters
SI                  32768
SF            100.6177975 MHz
WDW                    EM
SSB         0
LB                   1.00 Hz
GB          0
PC                   1.40
```

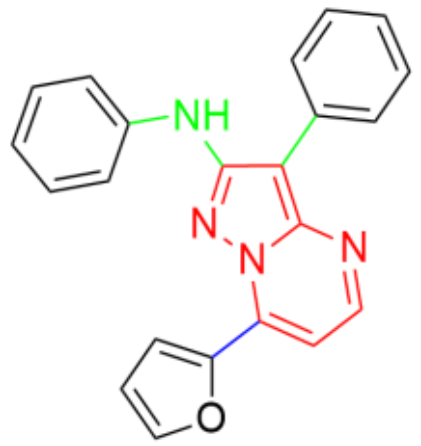
NH
N
N
N
O

6i

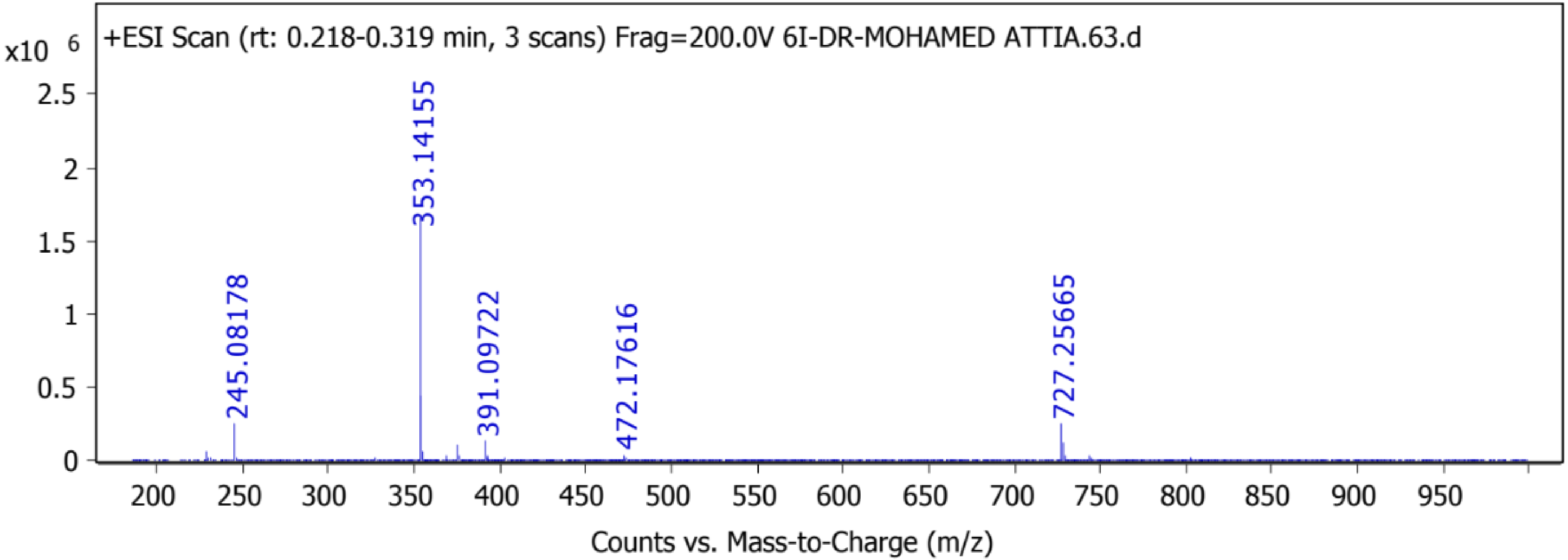
+ESI Scan (rt: 0.218-0.319 min, 3 scans) Frag=200.0V 6I-DR-MOHAMED ATTIA.63.d
x10 6
2.5
2
1.5
1
0.5
0
245.08178
353.14155
391.09722
472.17616
727.25665
200
250
300
350
400
450
500
550
600
650
700
750
800
850
900
950
Counts vs. Mass-to-Charge (m/z)

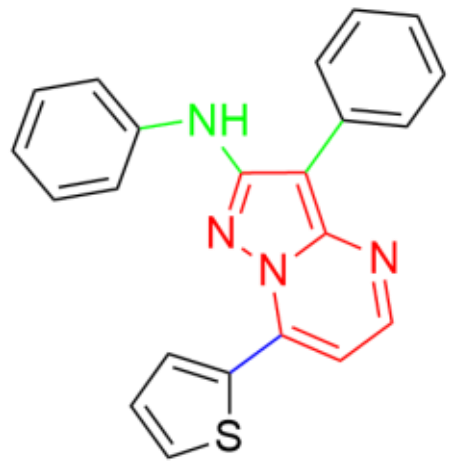

6j

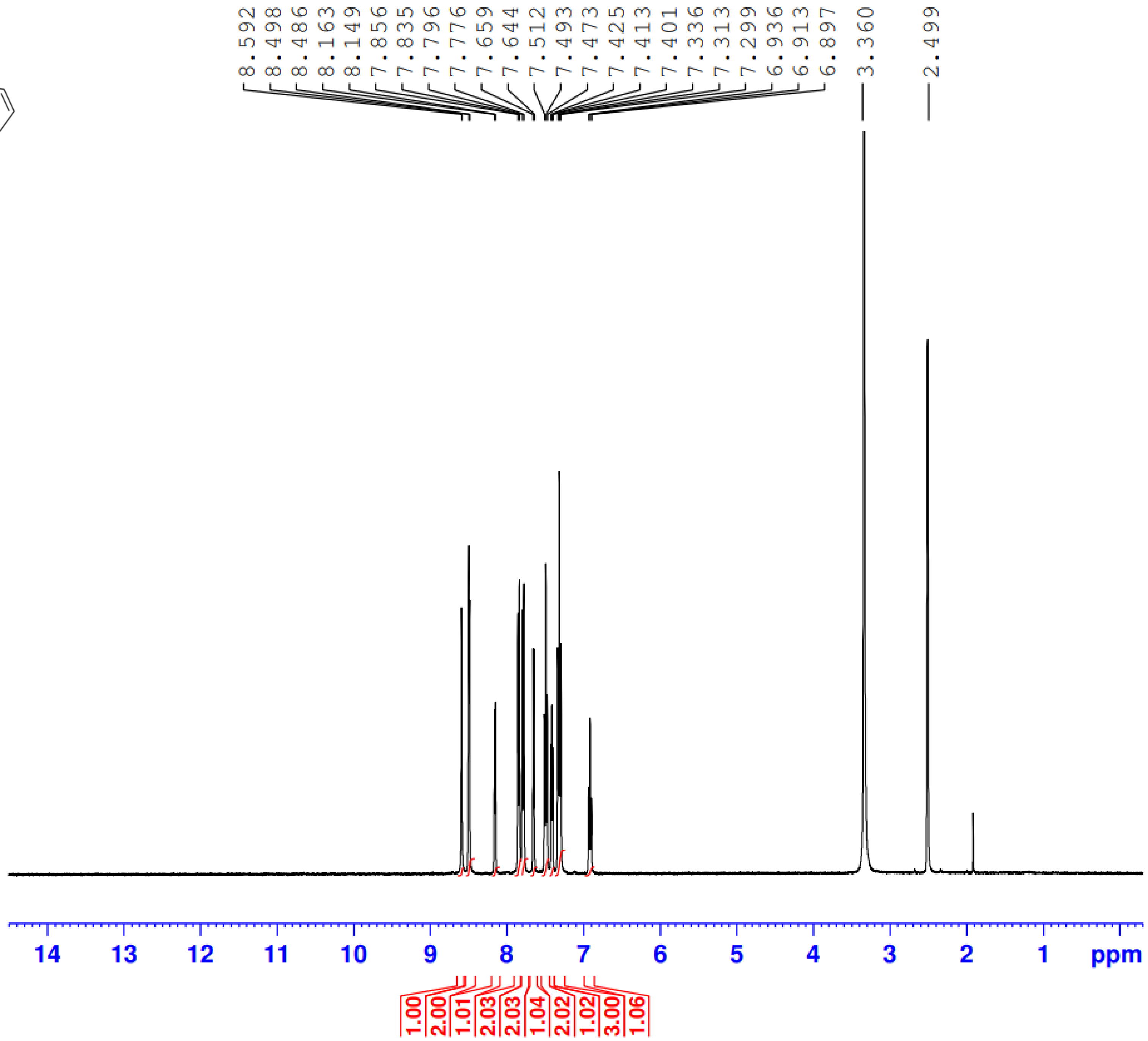

BRUKER

```
Current Data Parameters
NAME        mohammed-attia-13k
EXPNO                        1
PROCNO                       1

F2 - Acquisition Parameters
Date_                 20210520
Time                     14.01
INSTRUM                  spect
PROBHD     5 mm PABBO BB/
PULPROG                   zg30
TD                       65536
SOLVENT                   DMSO
NS                          22
DS                           2
SWH                   8012.820 Hz
FIDRES                0.122266 Hz
AQ                   4.0894465 sec
RG                      205.37
DW                      62.400 usec
DE                        6.50 usec
TE                       300.0 K
D1                  1.00000000 sec
TD0                          1

======== CHANNEL f1 ========
SFO1               400.1524711 MHz
NUC1                        1H
P1                       12.00 usec
PLW1               18.00000000 W

F2 - Processing parameters
SI                       65536
SF                 400.1500000 MHz
WDW                         EM
SSB      0
LB                        0.30 Hz
GB       0
PC                        1.00
```

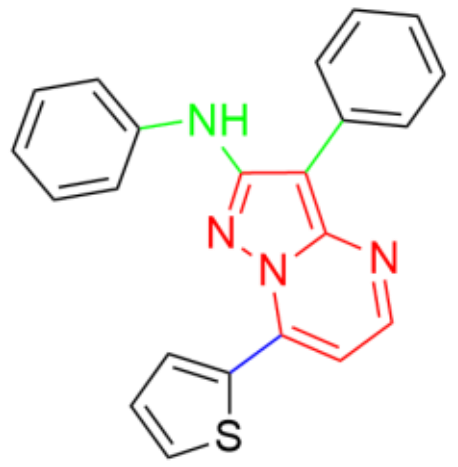

6j

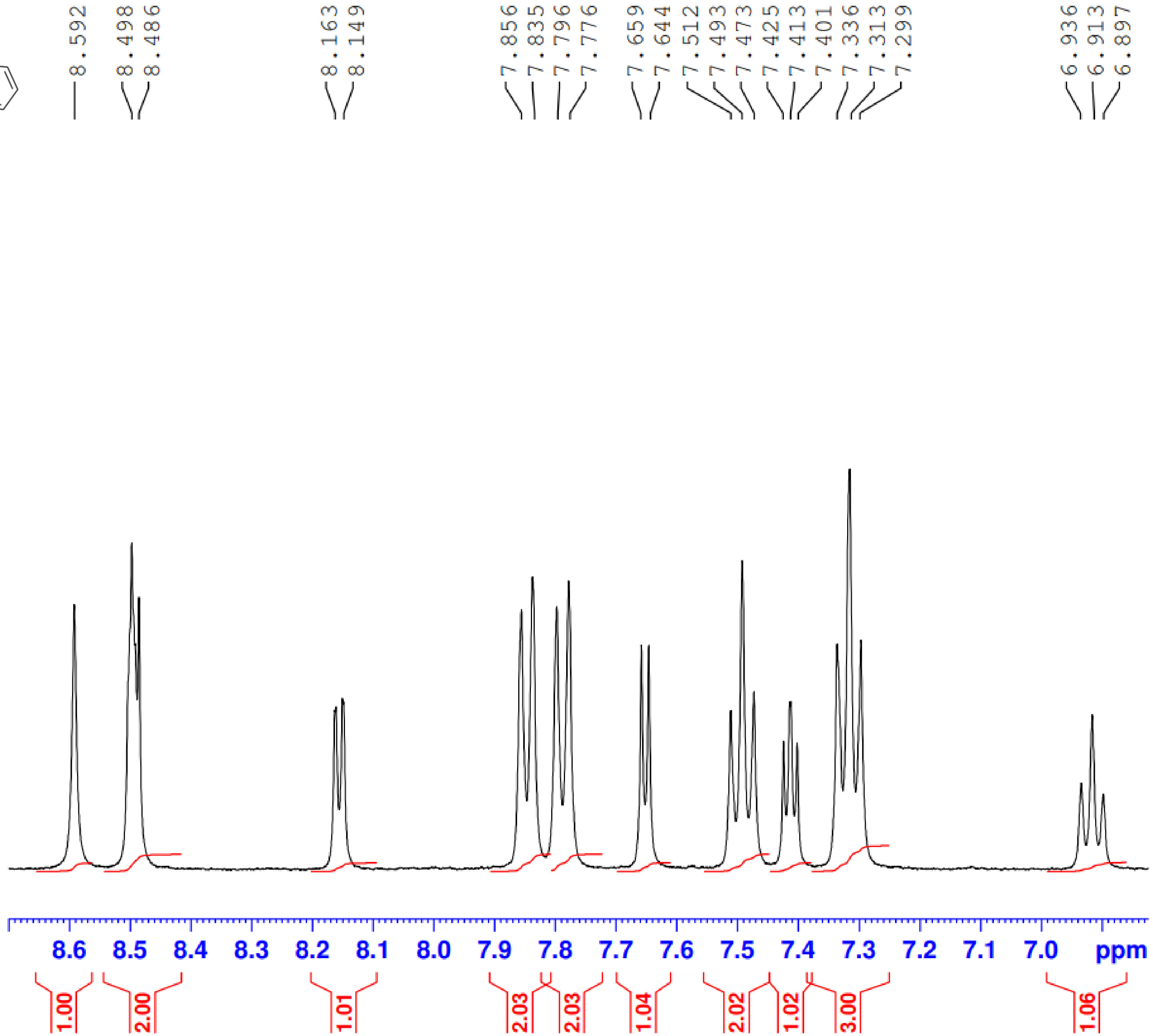


```
Current Data Parameters
NAME       mohammed-attia-13k
EXPNO                       1
PROCNO                      1

F2 - Acquisition Parameters
Date_                20210520
Time                    14.01
INSTRUM                 spect
PROBHD     5 mm PABBO BB/
PULPROG                  zg30
TD                      65536
SOLVENT                  DMSO
NS                         22
DS                          2
SWH                  8012.820 Hz
FIDRES               0.122266 Hz
AQ                  4.0894465 sec
RG                     205.37
DW                     62.400 usec
DE                       6.50 usec
TE                      300.0 K
D1                 1.00000000 sec
TD0                         1

======== CHANNEL f1 ========
SFO1              400.1524711 MHz
NUC1                       1H
P1                      12.00 usec
PLW1              18.00000000 W

F2 - Processing parameters
SI                      65536
SF                400.1500000 MHz
WDW                        EM
SSB      0
LB                       0.30 Hz
GB       0
PC                       1.00
```

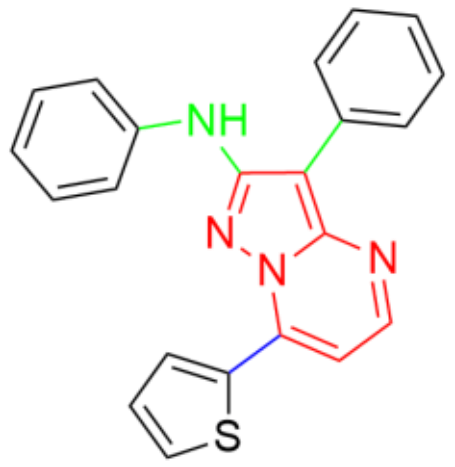

6j

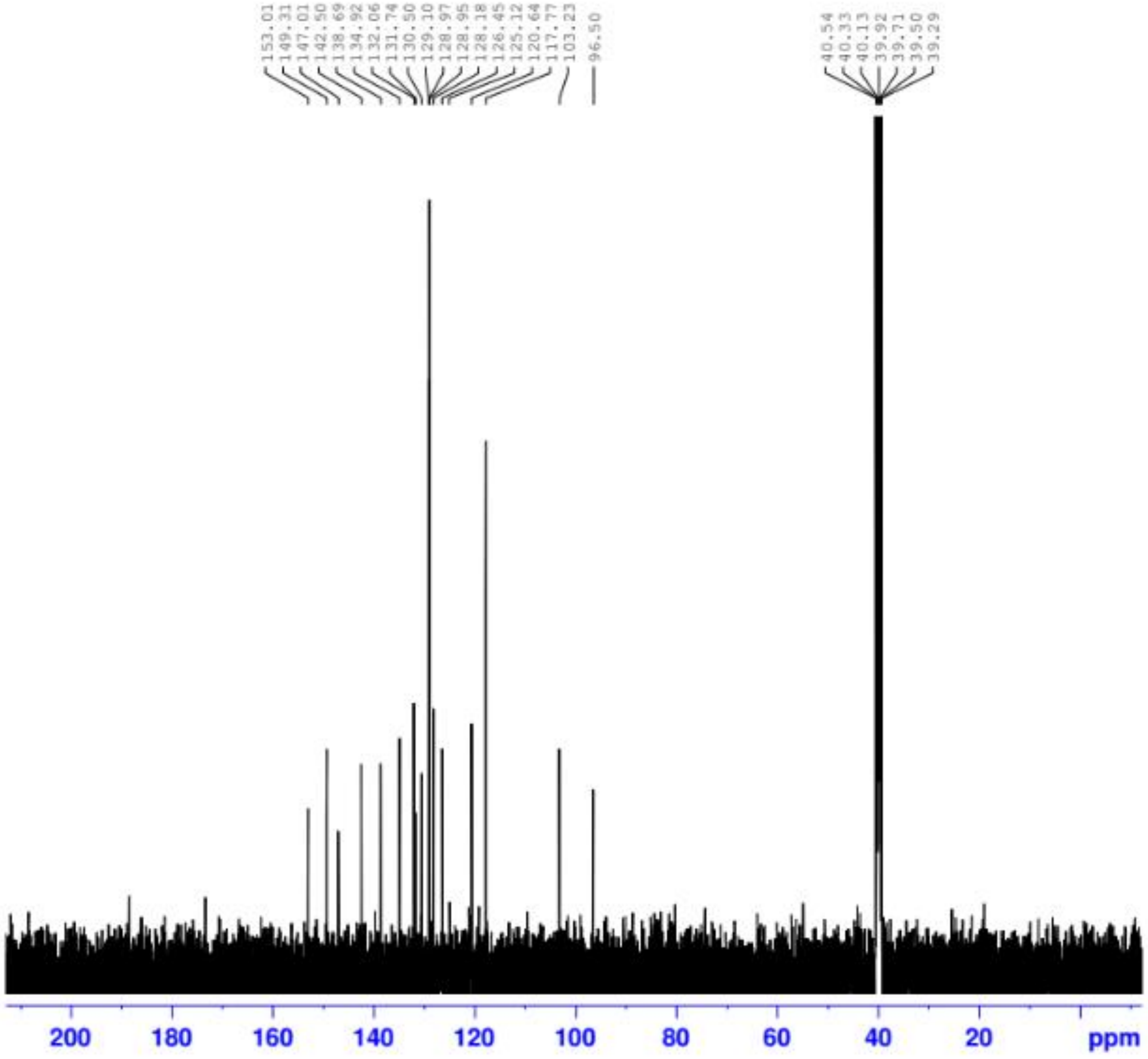

BRUKER

Current Data Parameters
NAME mohammed-attia-13k
EXPNO 2
PROCNO 1

F2 - Acquisition Parameters
Date_ 20210627
Time 13.13
INSTRUM spect
PROBHD 5 mm PABBO BB/
PULPROG zgpg30
TD 65536
SOLVENT DMSO
NS 1215
DS 4
SWH 24038.461 Hz
FIDRES 0.366798 Hz
AQ 1.3631488 sec
RG 205.37
DW 20.800 usec
DE 6.50 usec
TE 300.0 K
D1 2.00000000 sec
D11 0.03000000 sec
TD0 1

======== CHANNEL f1 ========
SFO1 100.6278588 MHz
NUC1 13C
P1 10.00 usec
PLW1 47.00000000 W

======== CHANNEL f2 ========
SFO2 400.1516006 MHz
NUC2 1H
CPDPRG[2 waltz16
PCPD2 90.00 usec
PLW2 18.00000000 W
PLW12 0.34722000 W
PLW13 0.28125000 W

F2 - Processing parameters
SI 32768
SF 100.6177975 MHz
WDW EM
SSB 0
LB 1.00 Hz
GB 0
PC 1.40

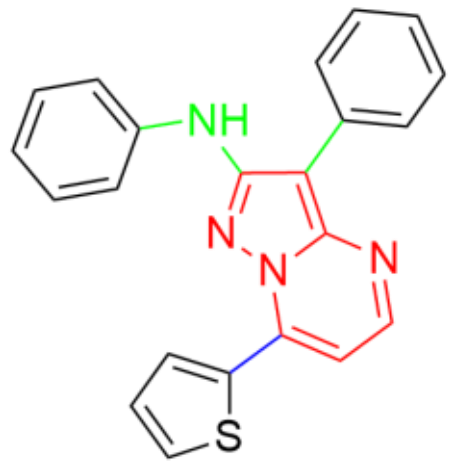

6j

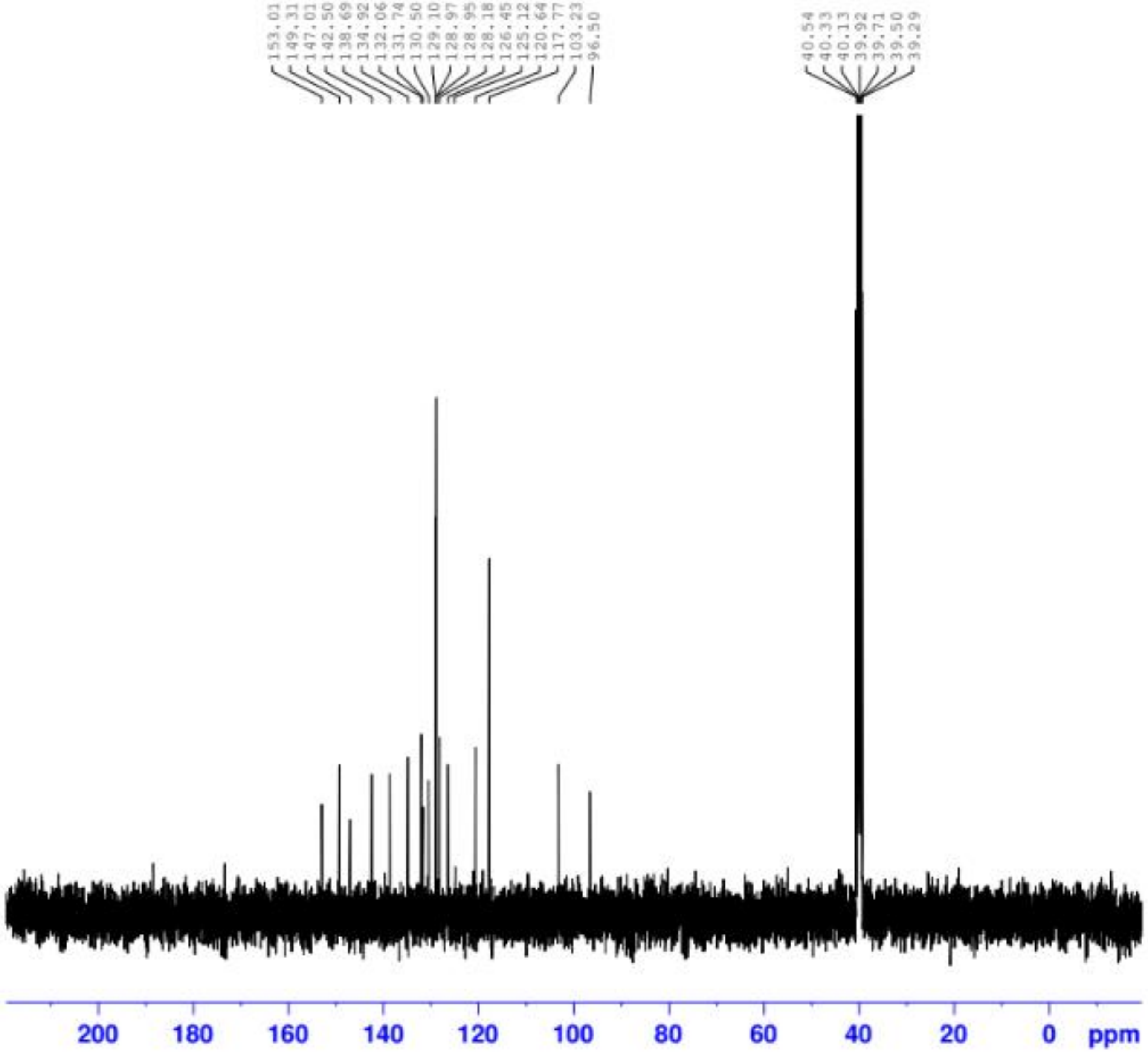

BRUKER

```
Current Data Parameters
NAME       mohammed-attia-13k
EXPNO                       2
PROCNO                      1

F2 - Acquisition Parameters
Date_              20210627
Time                  13.13
INSTRUM               spect
PROBHD   5 mm PABBO BB/
PULPROG             zgpg30
TD                   65536
SOLVENT               DMSO
NS                    1215
DS                       4
SWH              24038.461 Hz
FIDRES            0.366798 Hz
AQ               1.3631488 sec
RG                  205.37
DW                  20.800 usec
DE                    6.50 usec
TE                   300.0 K
D1              2.00000000 sec
D11             0.03000000 sec
TD0                      1

======== CHANNEL f1 ========
SFO1           100.6278588 MHz
NUC1                   13C
P1                   10.00 usec
PLW1           47.00000000 W

======== CHANNEL f2 ========
SFO2           400.1516006 MHz
NUC2                    1H
CPDPRG[2           waltz16
PCPD2                90.00 usec
PLW2           18.00000000 W
PLW12           0.34722000 W
PLW13           0.28125000 W

F2 - Processing parameters
SI                   32768
SF             100.6177975 MHz
WDW                     EM
SSB      0
LB                    1.00 Hz
GB       0
PC                    1.40
```

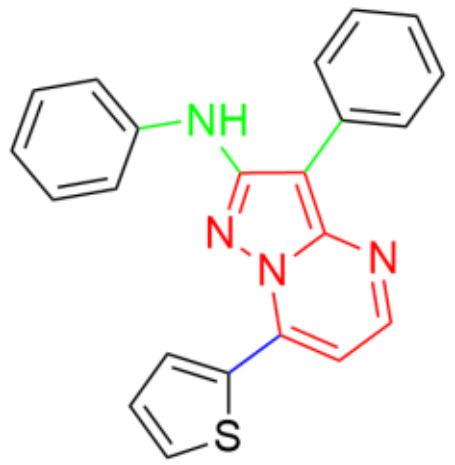

6j

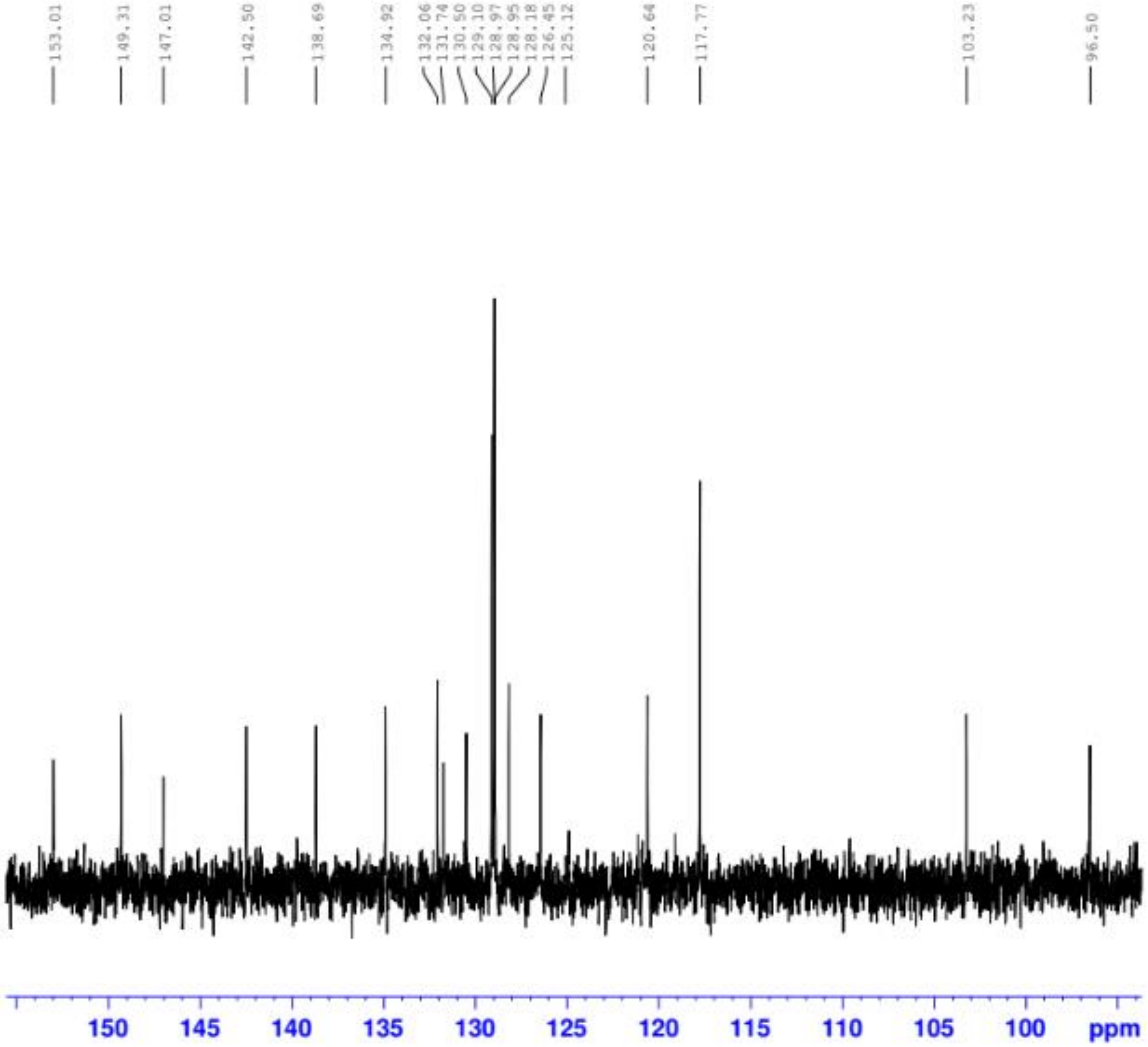

Current Data Parameters
NAME mohammed-attia-13k
EXPNO 2
PROCNO 1

F2 - Acquisition Parameters
Date_ 20210627
Time 13.13
INSTRUM spect
PROBHD 5 mm PABBO BB/
PULPROG zgpg30
TD 65536
SOLVENT DMSO
NS 1215
DS 4
SWH 24038.461 Hz
FIDRES 0.366798 Hz
AQ 1.3631488 sec
RG 205.37
DW 20.800 usec
DE 6.50 usec
TE 300.0 K
D1 2.00000000 sec
D11 0.03000000 sec
TD0 1

======== CHANNEL f1 ========
SFO1 100.6278588 MHz
NUC1 13C
P1 10.00 usec
PLW1 47.00000000 W

======== CHANNEL f2 ========
SFO2 400.1516006 MHz
NUC2 1H
CPDPRG[2 waltz16
PCPD2 90.00 usec
PLW2 18.00000000 W
PLW12 0.34722000 W
PLW13 0.28125000 W

F2 - Processing parameters
SI 32768
SF 100.6177975 MHz
WDW EM
SSB 0
LB 1.00 Hz
GB 0
PC 1.40

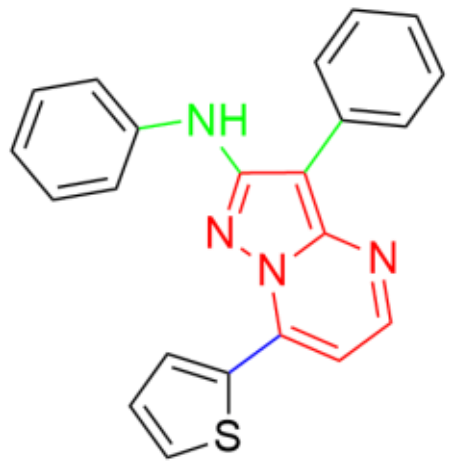
NH
N
N
N
S

6j

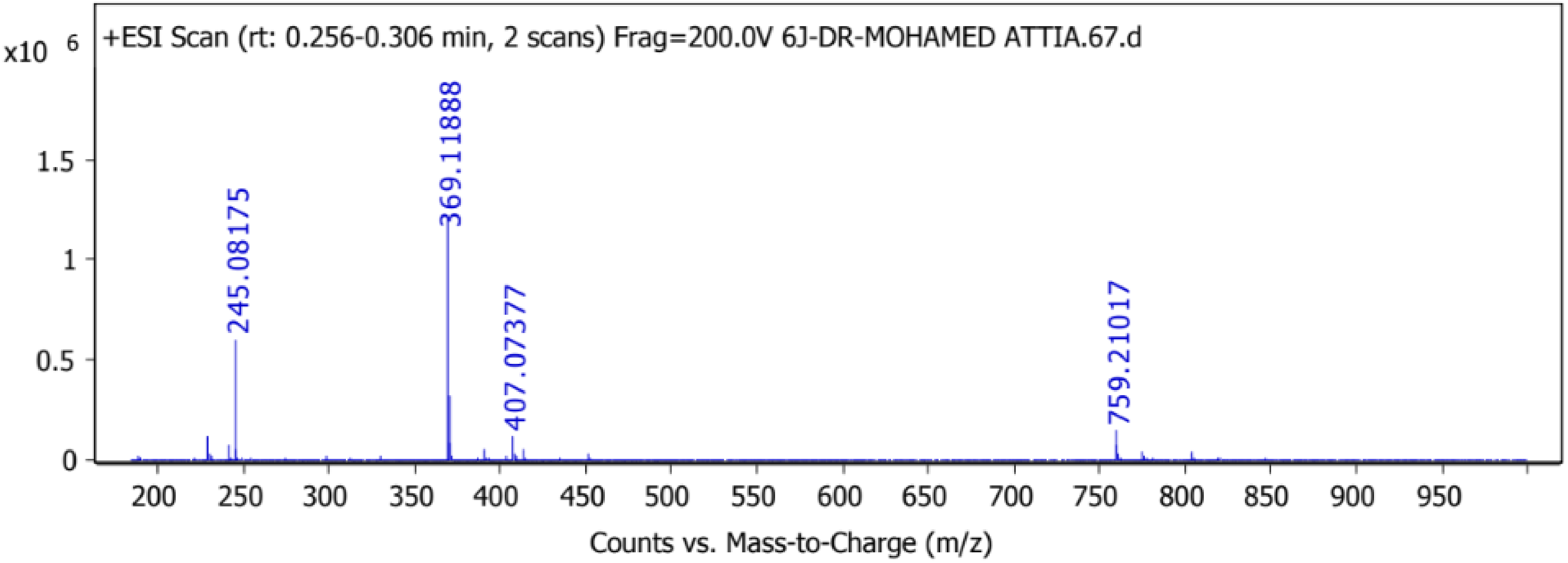
x10 6
+ESI Scan (rt: 0.256-0.306 min, 2 scans) Frag=200.0V 6J-DR-MOHAMED ATTIA.67.d
1.5
1
0.5
0
245.08175
369.11888
407.07377
759.21017
200
250
300
350
400
450
500
550
600
650
700
750
800
850
900
950
Counts vs. Mass-to-Charge (m/z)

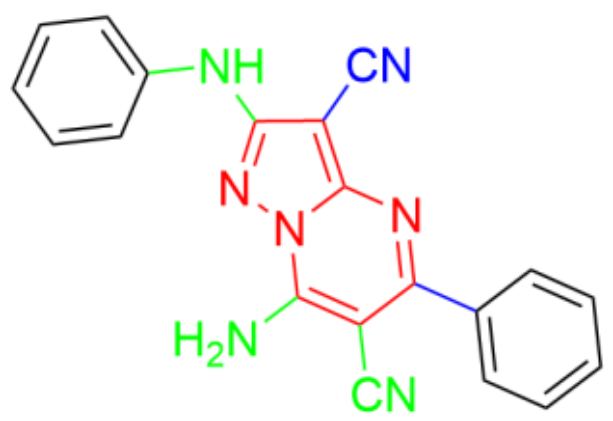

9a

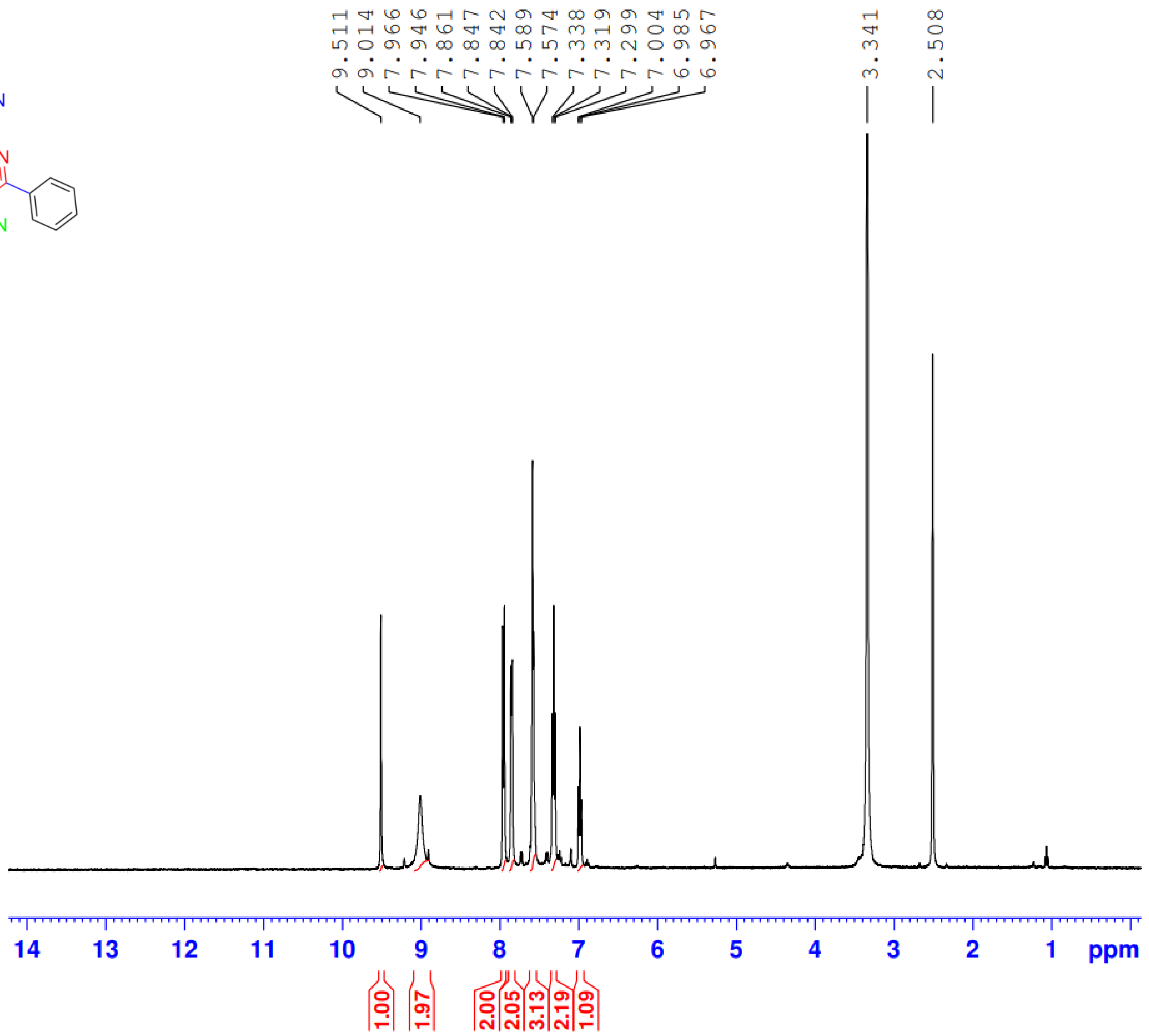


```
Current Data Parameters
NAME       mohammed-attia-19a
EXPNO                       1
PROCNO                      1

F2 - Acquisition Parameters
Date_              20210524
Time                  14.57
INSTRUM               spect
PROBHD    5 mm PABBO BB/
PULPROG                zg30
TD                    65536
SOLVENT                DMSO
NS                       32
DS                        2
SWH               8012.820 Hz
FIDRES            0.122266 Hz
AQ               4.0894465 sec
RG                   205.37
DW                   62.400 usec
DE                     6.50 usec
TE                    300.0 K
D1               1.00000000 sec
TD0                       1

======== CHANNEL f1 ========
SFO1           400.1524711 MHz
NUC1                     1H
P1                    12.00 usec
PLW1           18.00000000 W

F2 - Processing parameters
SI                    65536
SF             400.1500000 MHz
WDW                      EM
SSB      0
LB                     0.30 Hz
GB       0
PC                     1.00
```

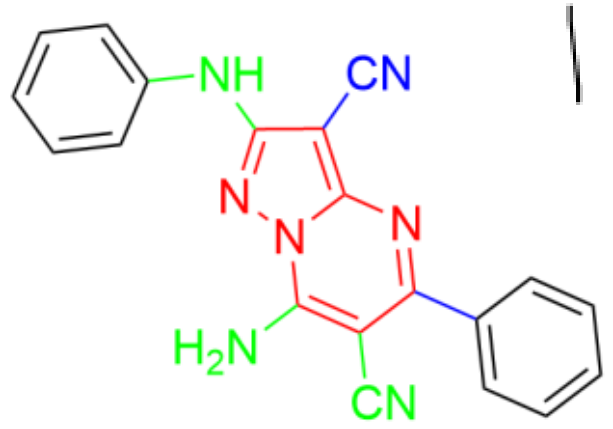

9a

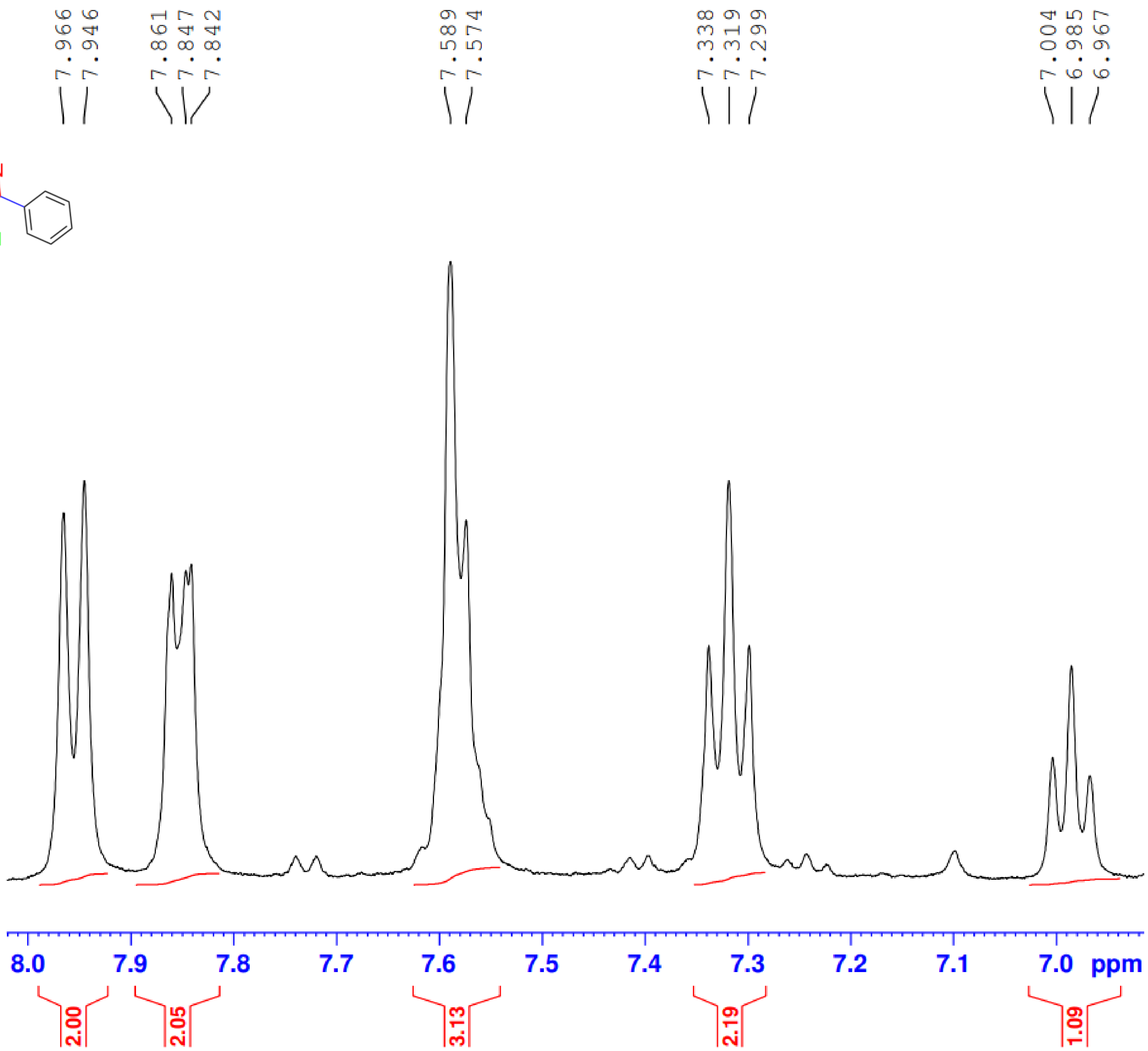

BRUKER

```
Current Data Parameters
NAME        mohammed-attia-19a
EXPNO                      1
PROCNO                     1

F2 - Acquisition Parameters
Date_               20210524
Time                   14.57
INSTRUM                spect
PROBHD     5 mm PABBO BB/
PULPROG                 zg30
TD                     65536
SOLVENT                 DMSO
NS                        32
DS                         2
SWH              8012.820 Hz
FIDRES           0.122266 Hz
AQ             4.0894465 sec
RG                    205.37
DW                62.400 usec
DE                  6.50 usec
TE                   300.0 K
D1            1.00000000 sec
TD0                        1

======== CHANNEL f1 ========
SFO1        400.1524711 MHz
NUC1                      1H
P1                 12.00 usec
PLW1          18.00000000 W

F2 - Processing parameters
SI                     65536
SF          400.1500000 MHz
WDW                       EM
SSB       0
LB                   0.30 Hz
GB        0
PC                      1.00
```

D_2O

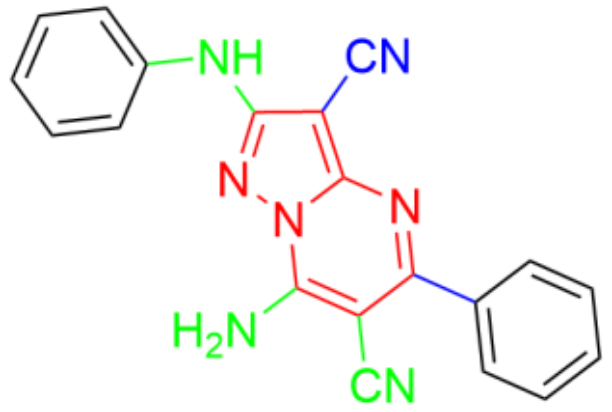

9a

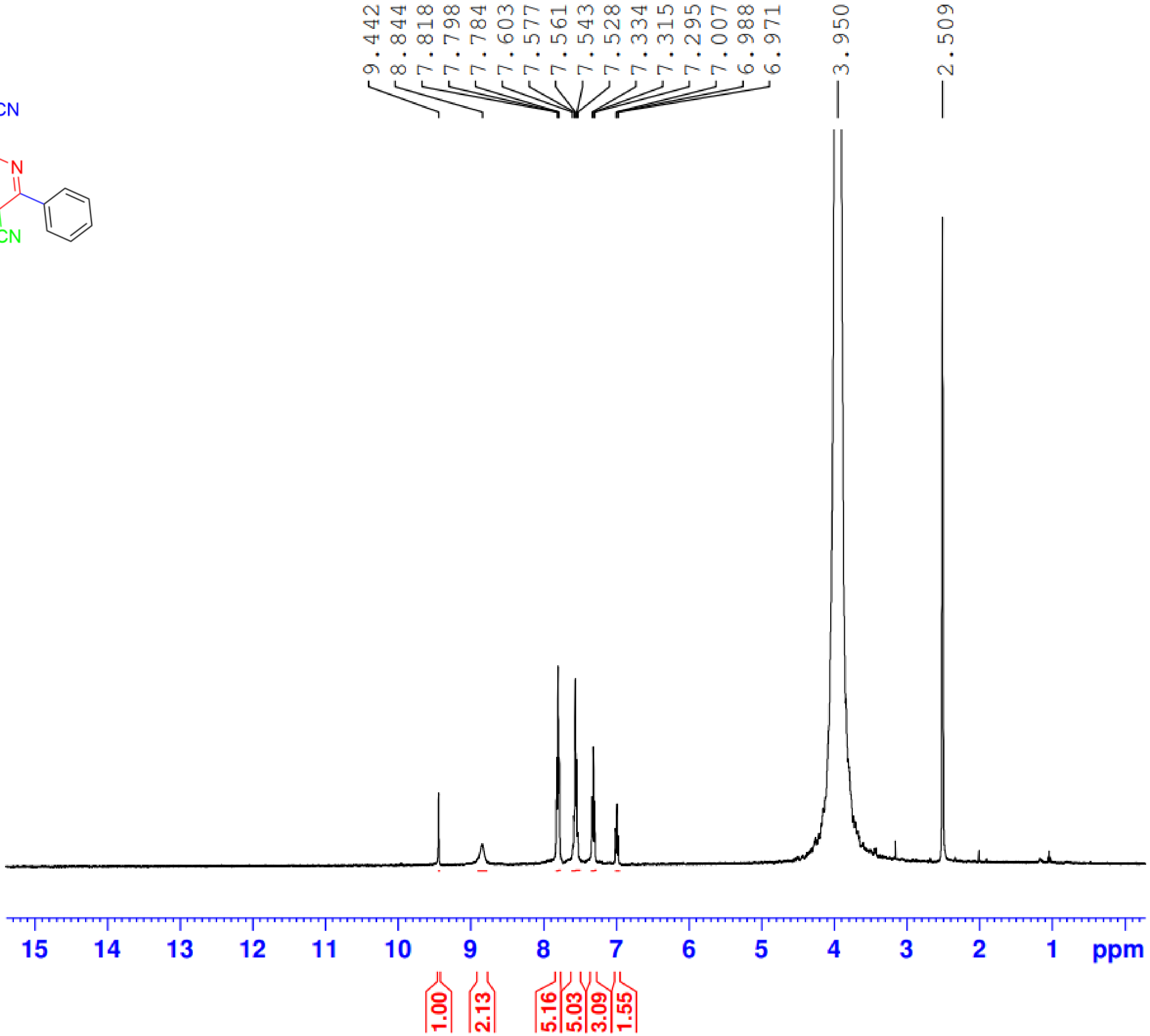


```
Current Data Parameters
NAME        mohamed-attia-19a-d2o
EXPNO                    1
PROCNO                   1

F2 - Acquisition Parameters
Date_             20210830
Time                 16.15
INSTRUM              spect
PROBHD    5 mm PABBO BB/
PULPROG              zg30
TD                   65536
SOLVENT               DMSO
NS                      79
DS                       2
SWH             8012.820 Hz
FIDRES          0.122266 Hz
AQ             4.0894465 sec
RG                   81.16
DW                  62.400 usec
DE                    6.50 usec
TE                   300.0 K
D1            1.00000000 sec
TD0                      1

======== CHANNEL f1 ========
SFO1         400.1524711 MHz
NUC1                    1H
P1                   12.00 usec
PLW1         18.00000000 W

F2 - Processing parameters
SI                   65536
SF           400.1500000 MHz
WDW                     EM
SSB       0
LB                    0.30 Hz
GB        0
PC                    1.00
```

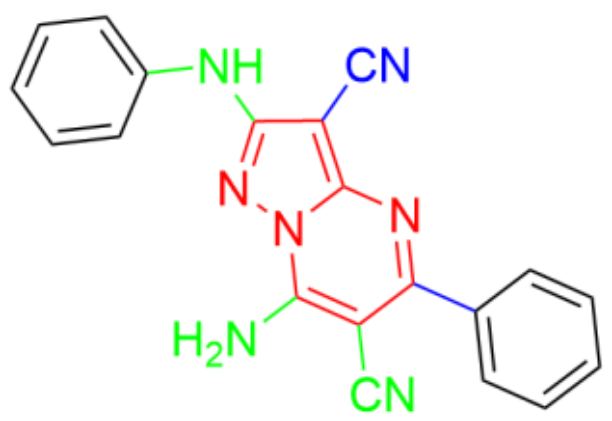

9a

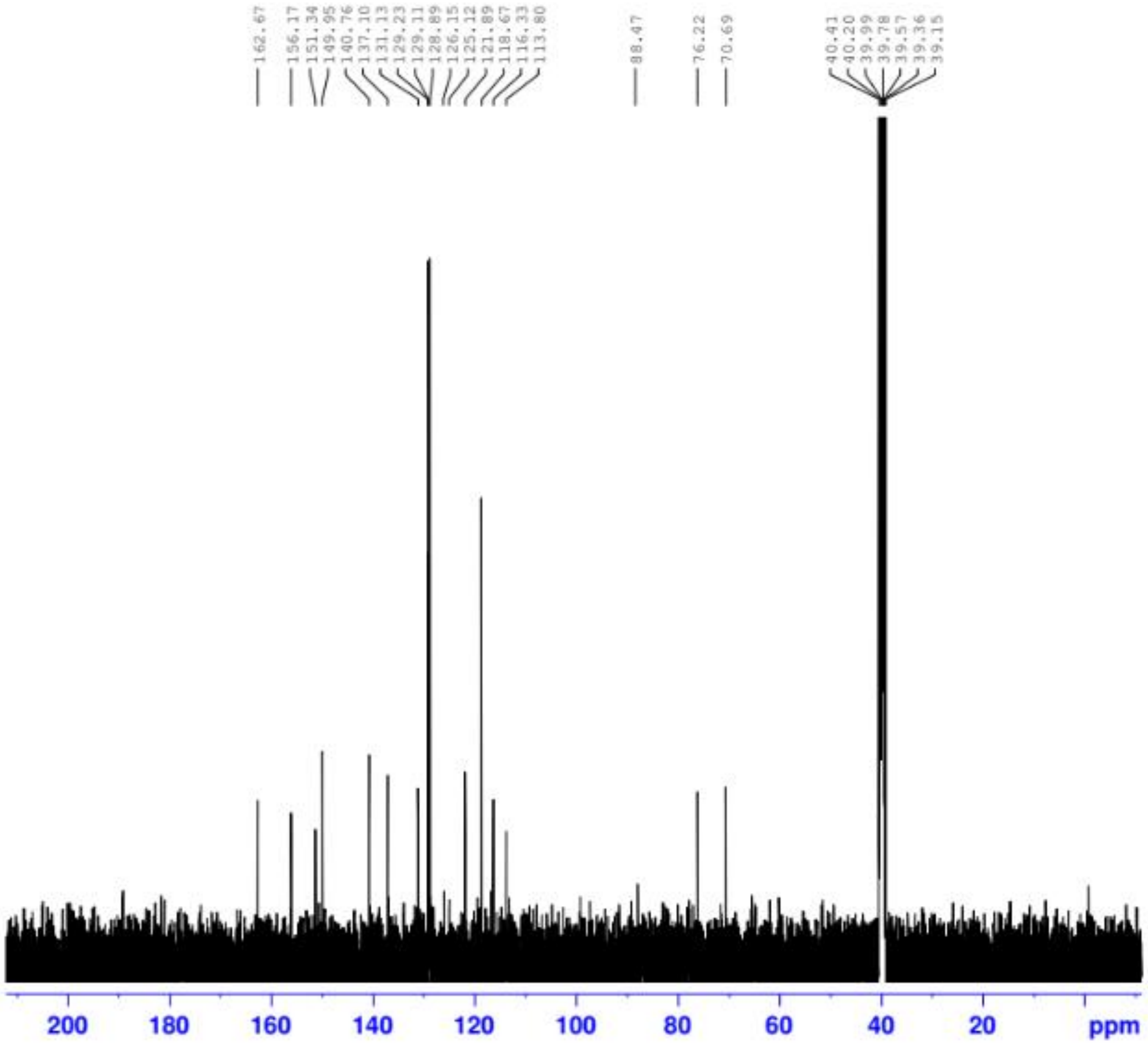

Current Data Parameters

NAME	mohammed-attia-19a
EXPNO	2
PROCNO	1

F2 - Acquisition Parameters

Date_	20210628
Time	13.43
INSTRUM	spect
PROBHD	5 mm PABBO BB/
PULPROG	zgpg30
TD	65536
SOLVENT	DMSO
NS	1638
DS	4
SWH	24038.461 Hz
FIDRES	0.366798 Hz
AQ	1.3631488 sec
RG	205.37
DW	20.800 usec
DE	6.50 usec
TE	300.0 K
D1	2.00000000 sec
D11	0.03000000 sec
TD0	1

======== CHANNEL f1 ========

SFO1	100.6278588 MHz
NUC1	13C
P1	10.00 usec
PLW1	47.00000000 W

======== CHANNEL f2 ========

SFO2	400.1516006 MHz
NUC2	1H
CPDPRG[2	waltz16
PCPD2	90.00 usec
PLW2	18.00000000 W
PLW12	0.34722000 W
PLW13	0.28125000 W

F2 - Processing parameters

SI	32768
SF	100.6177975 MHz
WDW	EM
SSB	0
LB	1.00 Hz
GB	0
PC	1.40

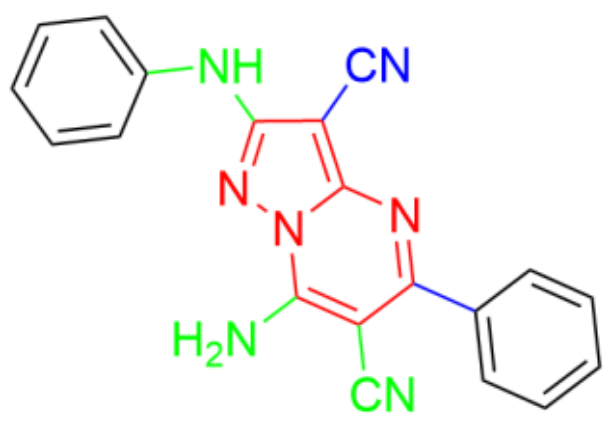

9a

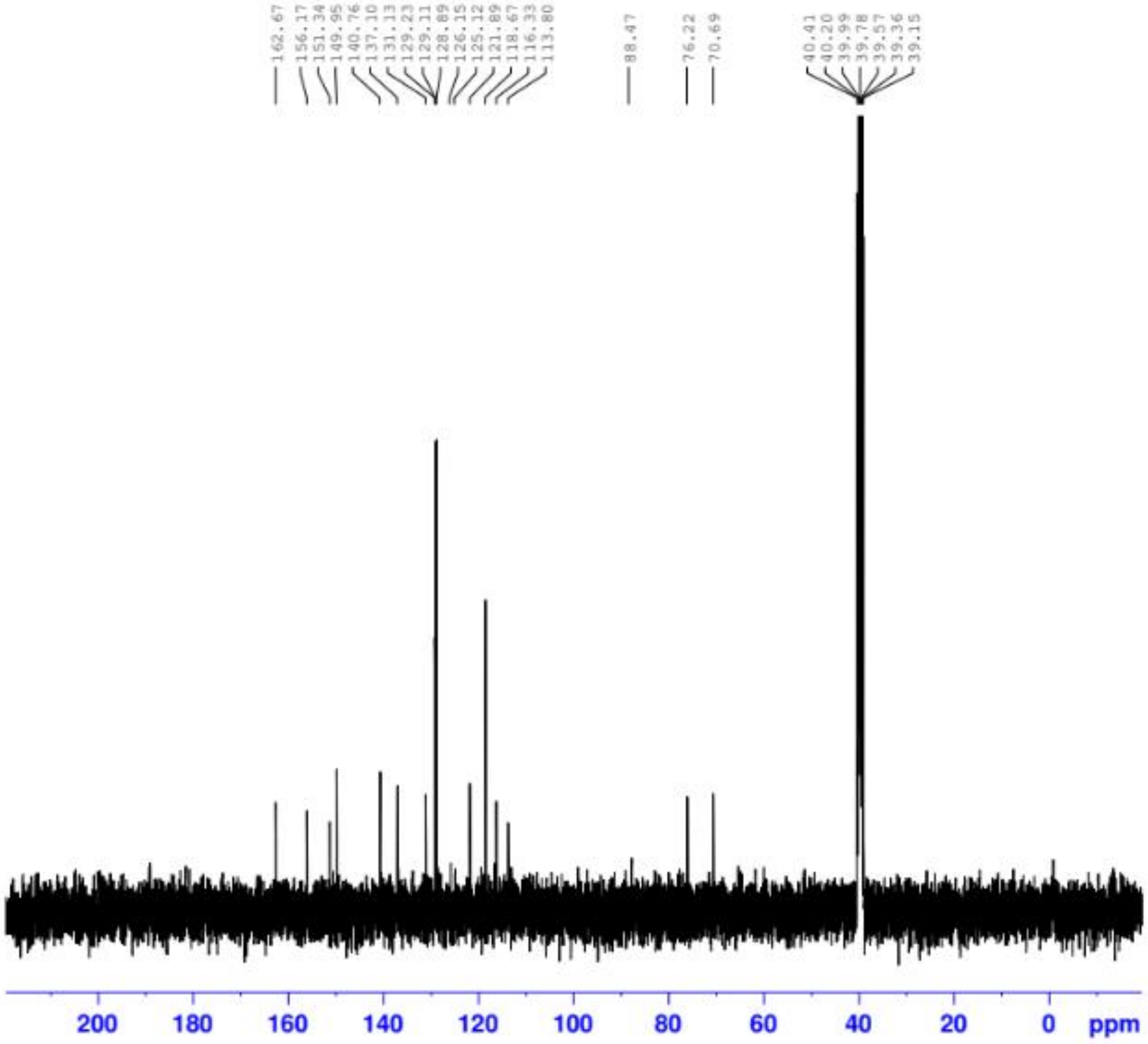

Current Data Parameters
NAME mohammed-attia-19a
EXPNO 2
PROCNO 1

F2 - Acquisition Parameters
Date_ 20210628
Time 13.43
INSTRUM spect
PROBHD 5 mm PABBO BB/
PULPROG zgpg30
TD 65536
SOLVENT DMSO
NS 1638
DS 4
SWH 24038.461 Hz
FIDRES 0.366798 Hz
AQ 1.3631488 sec
RG 205.37
DW 20.800 usec
DE 6.50 usec
TE 300.0 K
D1 2.00000000 sec
D11 0.03000000 sec
TD0 1

======== CHANNEL f1 ========
SFO1 100.6278588 MHz
NUC1 13C
P1 10.00 usec
PLW1 47.00000000 W

======== CHANNEL f2 ========
SFO2 400.1516006 MHz
NUC2 1H
CPDPRG[2 waltz16
PCPD2 90.00 usec
PLW2 18.00000000 W
PLW12 0.34722000 W
PLW13 0.28125000 W

F2 - Processing parameters
SI 32768
SF 100.6177975 MHz
WDW EM
SSB 0
LB 1.00 Hz
GB 0
PC 1.40

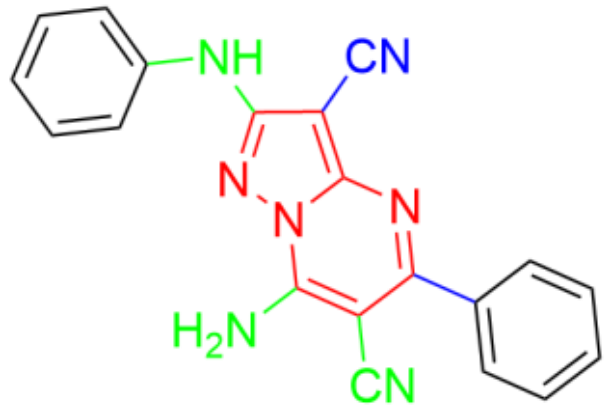

9a

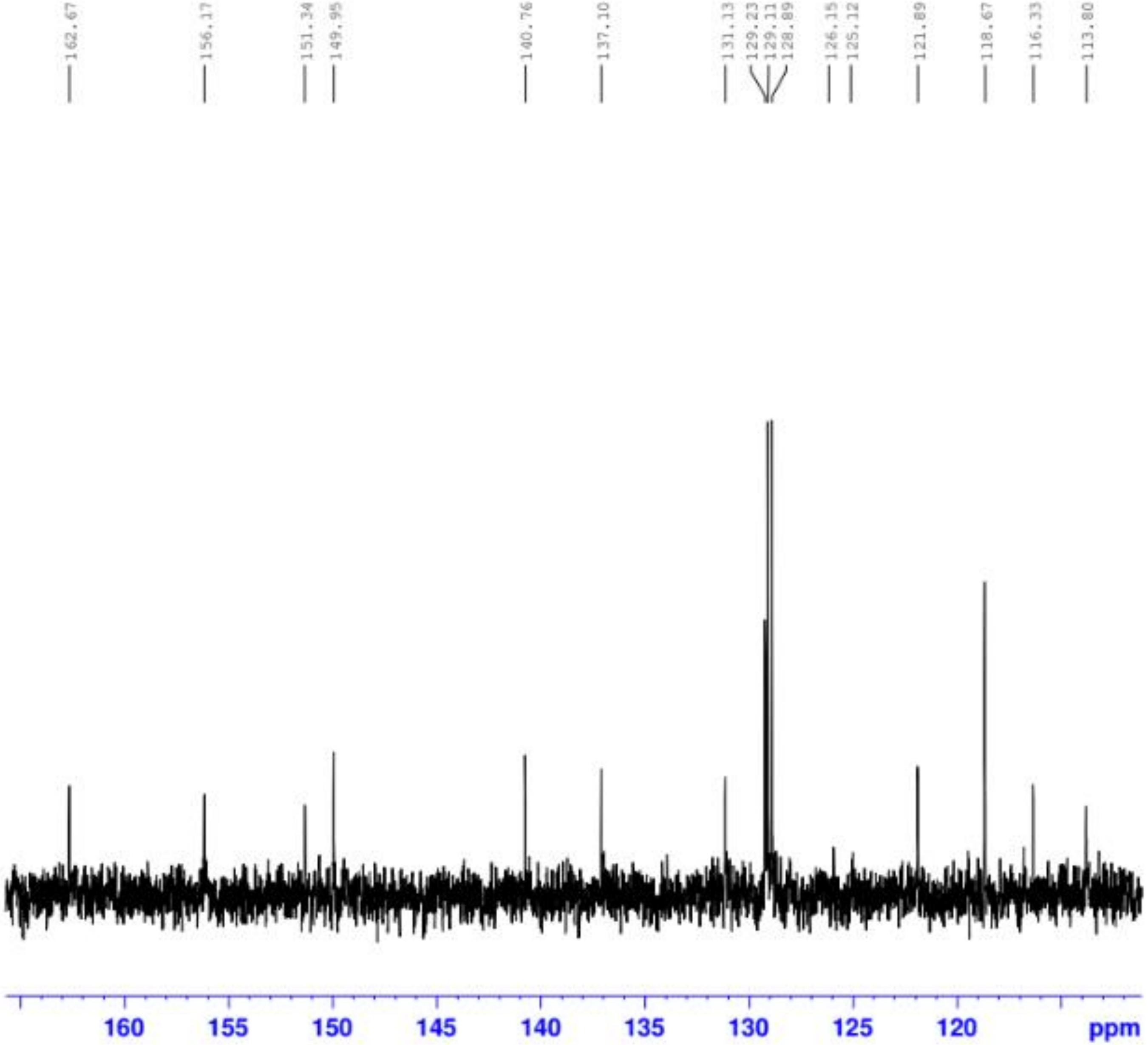


```
Current Data Parameters
NAME        mohammed-attia-19a
EXPNO                        2
PROCNO                       1

F2 - Acquisition Parameters
Date_                 20210628
Time                     13.43
INSTRUM                  spect
PROBHD     5 mm PABBO BB/
PULPROG                zgpg30
TD                       65536
SOLVENT                   DMSO
NS                        1638
DS                           4
SWH                  24038.461 Hz
FIDRES                0.366798 Hz
AQ                   1.3631488 sec
RG                      205.37
DW                      20.800 usec
DE                        6.50 usec
TE                       300.0 K
D1                  2.00000000 sec
D11                 0.03000000 sec
TD0                          1

======== CHANNEL f1 ========
SFO1               100.6278588 MHz
NUC1                       13C
P1                       10.00 usec
PLW1               47.00000000 W

======== CHANNEL f2 ========
SFO2               400.1516006 MHz
NUC2                        1H
CPDPRG[2               waltz16
PCPD2                    90.00 usec
PLW2               18.00000000 W
PLW12               0.34722000 W
PLW13               0.28125000 W

F2 - Processing parameters
SI                       32768
SF                 100.6177975 MHz
WDW                         EM
SSB      0
LB                        1.00 Hz
GB       0
PC                        1.40
```

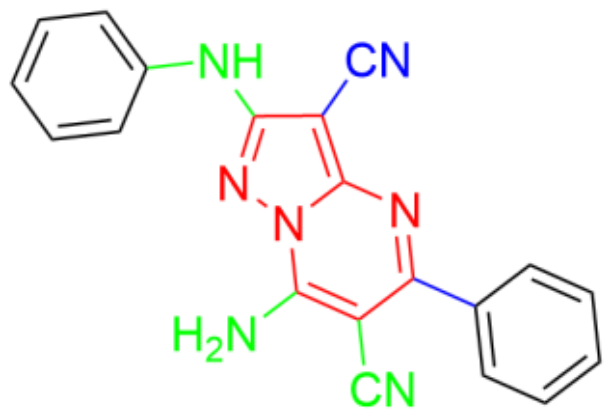
NH
CN
N
N
N
H2N
CN

9a

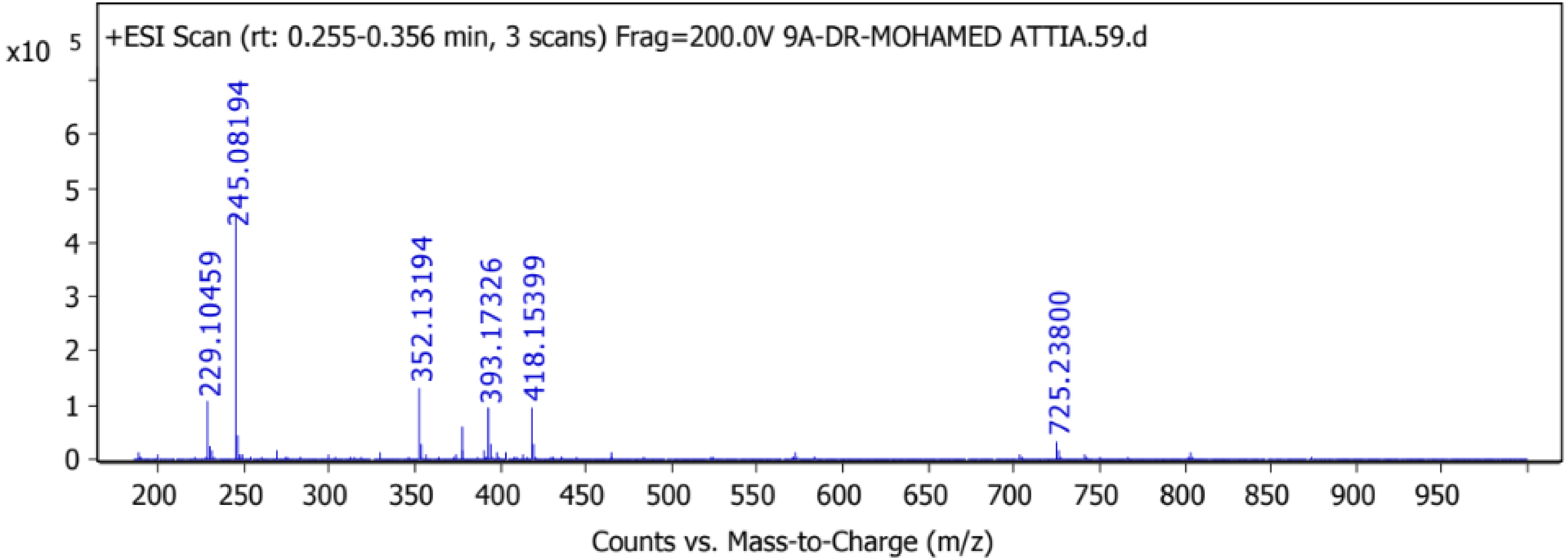
+ESI Scan (rt: 0.255-0.356 min, 3 scans) Frag=200.0V 9A-DR-MOHAMED ATTIA.59.d
x10 5
0
1
2
3
4
5
6
229.10459
245.08194
352.13194
393.17326
418.15399
725.23800
200
250
300
350
400
450
500
550
600
650
700
750
800
850
900
950
Counts vs. Mass-to-Charge (m/z)

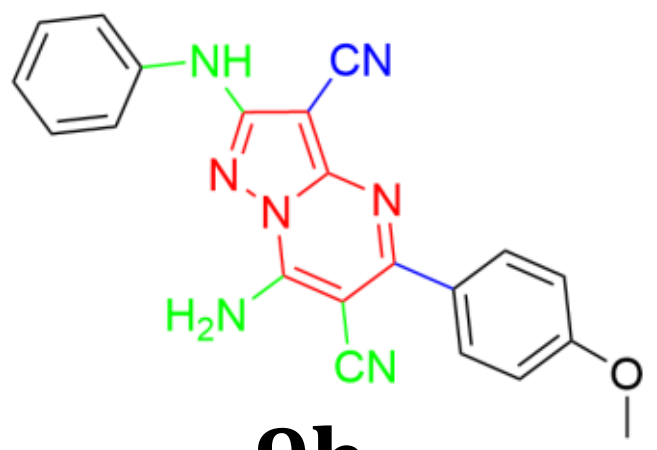

9b

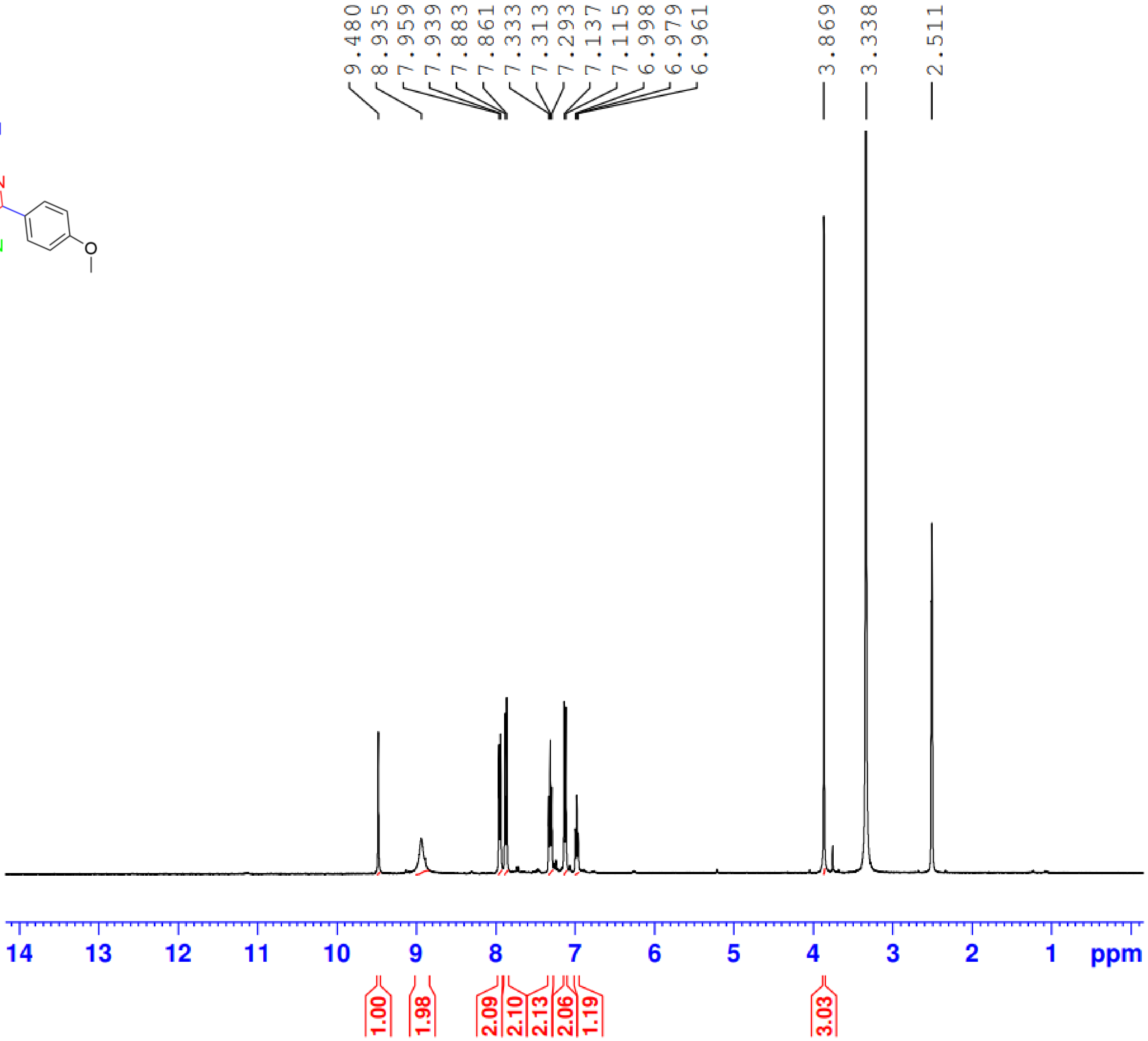

Current Data Parameters
NAME mohammed-attia-19c
EXPNO 1
PROCNO 1

F2 - Acquisition Parameters
Date_ 20210524
Time 14.46
INSTRUM spect
PROBHD 5 mm PABBO BB/
PULPROG zg30
TD 65536
SOLVENT DMSO
NS 33
DS 2
SWH 8012.820 Hz
FIDRES 0.122266 Hz
AQ 4.0894465 sec
RG 205.37
DW 62.400 usec
DE 6.50 usec
TE 300.0 K
D1 1.00000000 sec
TD0 1

======== CHANNEL f1 ========
SFO1 400.1524711 MHz
NUC1 1H
P1 12.00 usec
PLW1 18.00000000 W

F2 - Processing parameters
SI 65536
SF 400.1500000 MHz
WDW EM
SSB 0
LB 0.30 Hz
GB 0
PC 1.00

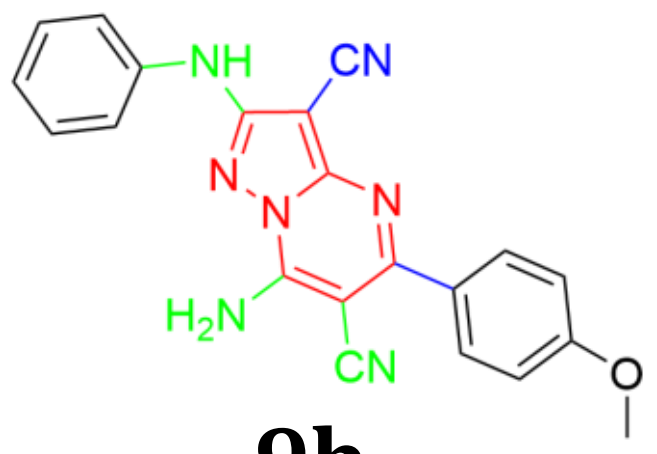

9b

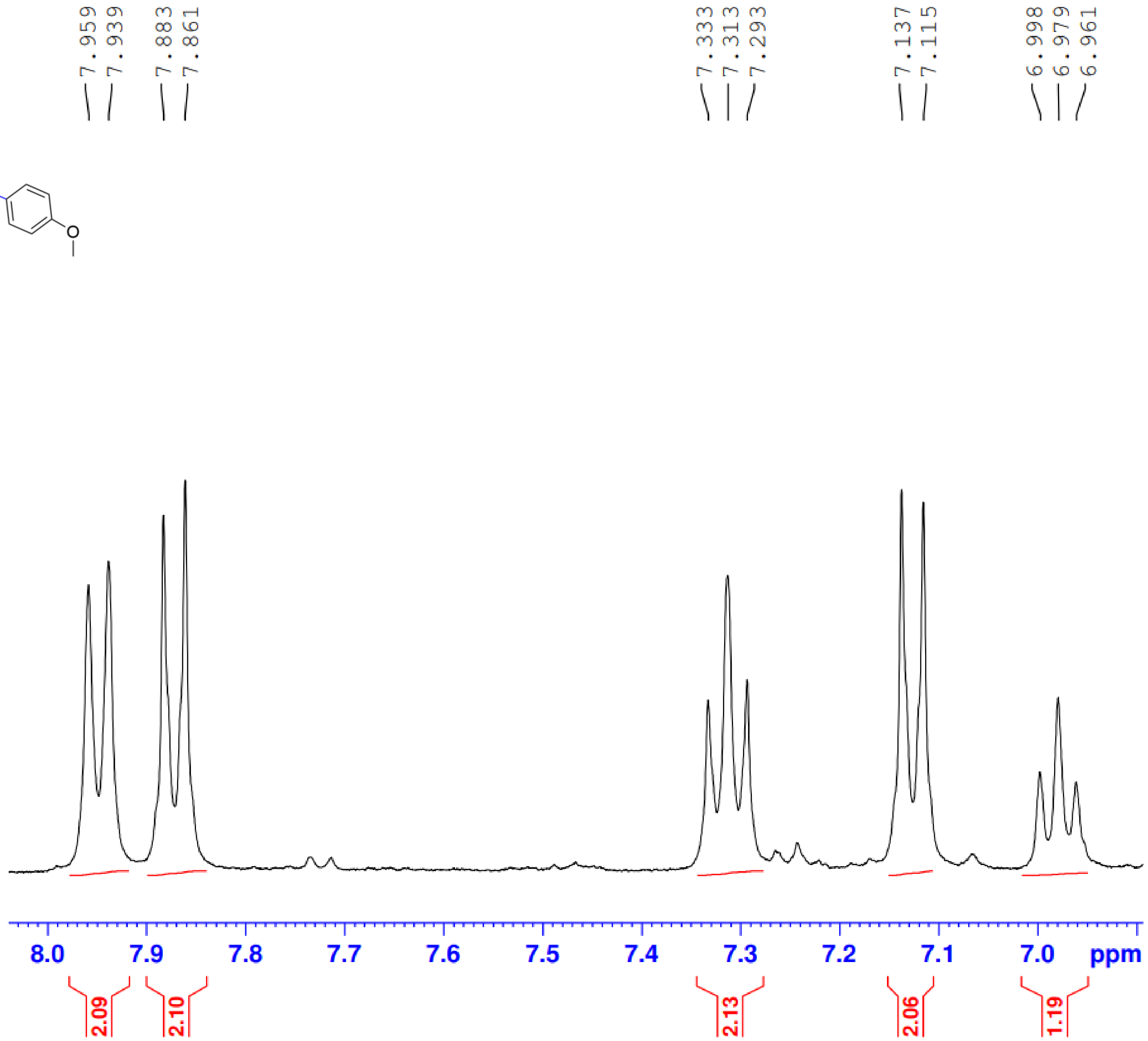

BRUKER

```
Current Data Parameters
NAME      mohammed-attia-19c
EXPNO                      1
PROCNO                     1

F2 - Acquisition Parameters
Date_               20210524
Time                   14.46
INSTRUM                spect
PROBHD    5 mm PABBO BB/
PULPROG                 zg30
TD                     65536
SOLVENT                 DMSO
NS                        33
DS                         2
SWH                 8012.820 Hz
FIDRES              0.122266 Hz
AQ                 4.0894465 sec
RG                    205.37
DW                    62.400 usec
DE                      6.50 usec
TE                     300.0 K
D1                1.00000000 sec
TD0                        1

======== CHANNEL f1 ========
SFO1             400.1524711 MHz
NUC1                      1H
P1                     12.00 usec
PLW1             18.00000000 W

F2 - Processing parameters
SI                     65536
SF               400.1500000 MHz
WDW                       EM
SSB       0
LB                      0.30 Hz
GB        0
PC                      1.00
```

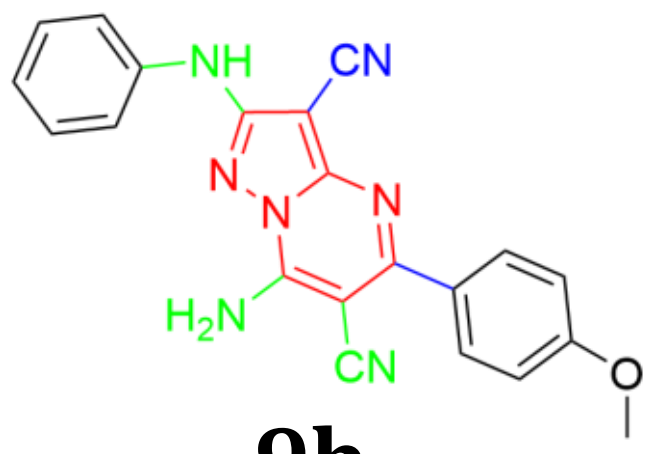

9b

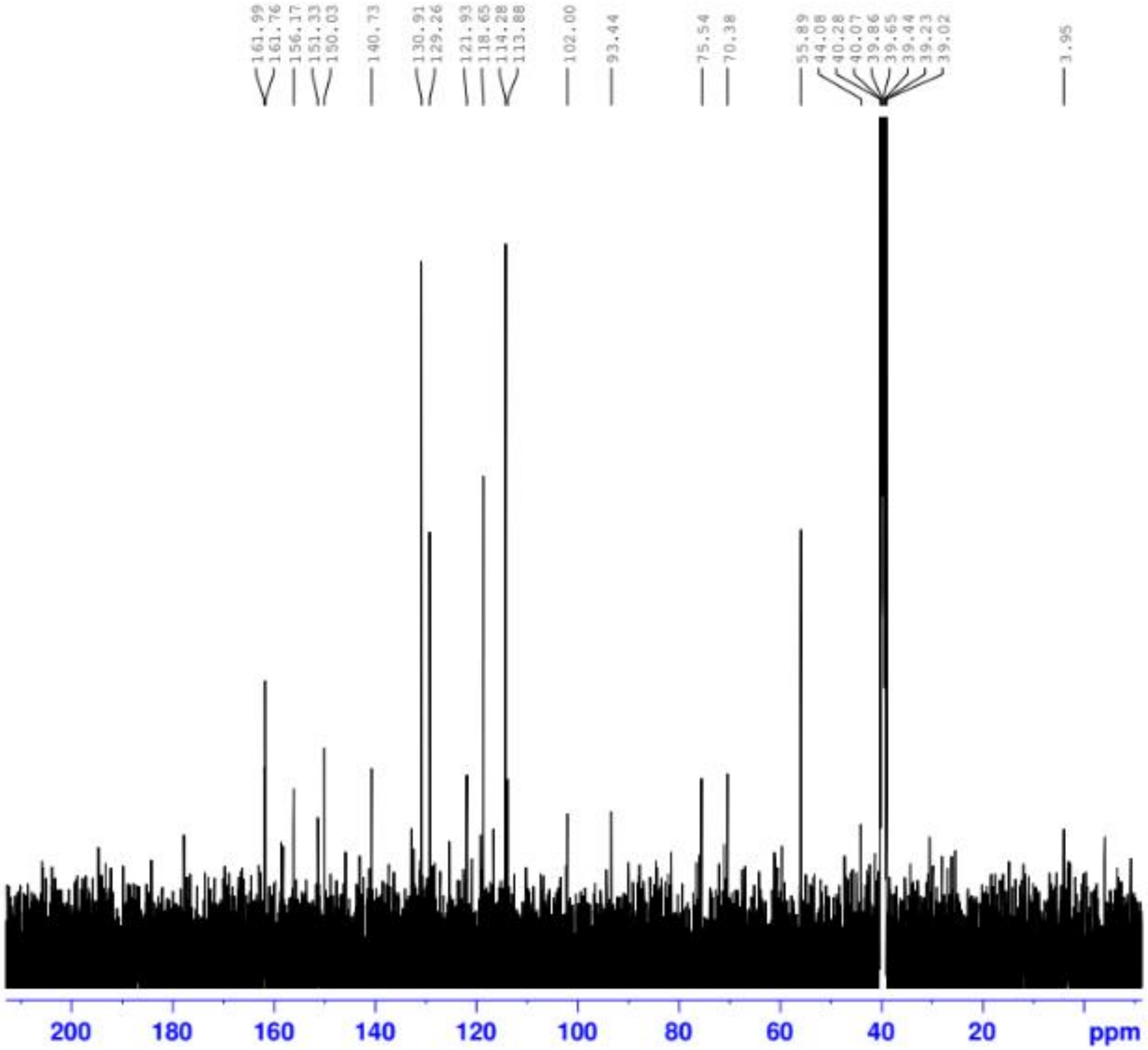

BRUKER

```
Current Data Parameters
NAME          mohammed-attia-19c
EXPNO                          2
PROCNO                         1

F2 - Acquisition Parameters
Date_                   20210624
Time                       13.50
INSTRUM                    spect
PROBHD      5 mm PABBO BB/
PULPROG                   zgpg30
TD                         65536
SOLVENT                     DMSO
NS                          1505
DS                             4
SWH                    24038.461 Hz
FIDRES                  0.366798 Hz
AQ                     1.3631488 sec
RG                        205.37
DW                        20.800 usec
DE                          6.50 usec
TE                         300.0 K
D1                    2.00000000 sec
D11                   0.03000000 sec
TD0                            1

======== CHANNEL f1 ========
SFO1                 100.6278588 MHz
NUC1                         13C
P1                         10.00 usec
PLW1                 47.00000000 W

======== CHANNEL f2 ========
SFO2                 400.1516006 MHz
NUC2                          1H
CPDPRG[2                 waltz16
PCPD2                      90.00 usec
PLW2                 18.00000000 W
PLW12                 0.34722000 W
PLW13                 0.28125000 W

F2 - Processing parameters
SI                         32768
SF                   100.6177975 MHz
WDW                           EM
SSB      0
LB                          1.00 Hz
GB       0
PC                          1.40
```

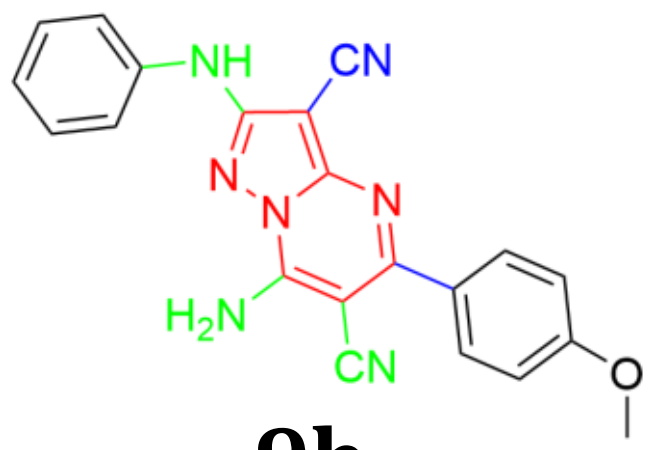

9b

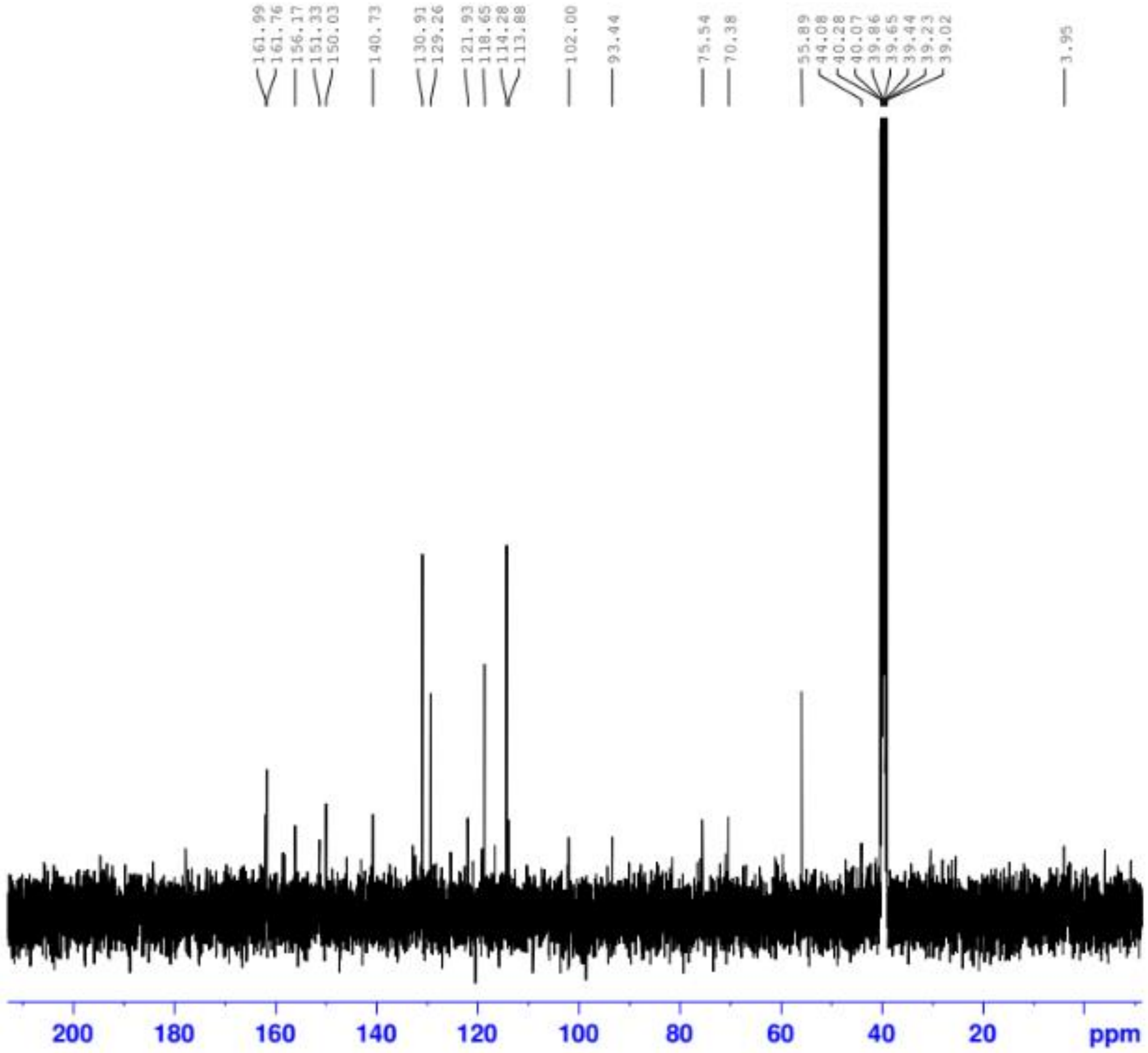

BRUKER

Current Data Parameters
NAME mohammed-attia-19c
EXPNO 2
PROCNO 1

F2 - Acquisition Parameters
Date_ 20210624
Time 13.50
INSTRUM spect
PROBHD 5 mm PABBO BB/
PULPROG zgpg30
TD 65536
SOLVENT DMSO
NS 1505
DS 4
SWH 24038.461 Hz
FIDRES 0.366798 Hz
AQ 1.3631488 sec
RG 205.37
DW 20.800 usec
DE 6.50 usec
TE 300.0 K
D1 2.00000000 sec
D11 0.03000000 sec
TD0 1

======== CHANNEL f1 ========
SFO1 100.6278588 MHz
NUC1 13C
P1 10.00 usec
PLW1 47.00000000 W

======== CHANNEL f2 ========
SFO2 400.1516006 MHz
NUC2 1H
CPDPRG[2 waltz16
PCPD2 90.00 usec
PLW2 18.00000000 W
PLW12 0.34722000 W
PLW13 0.28125000 W

F2 - Processing parameters
SI 32768
SF 100.6177975 MHz
WDW EM
SSB 0
LB 1.00 Hz
GB 0
PC 1.40

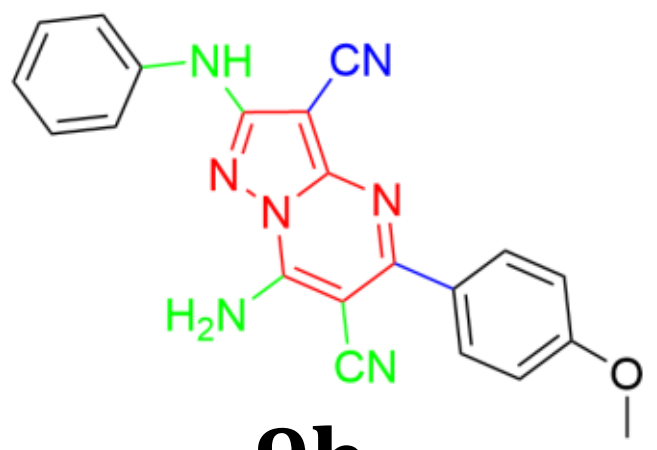

9b

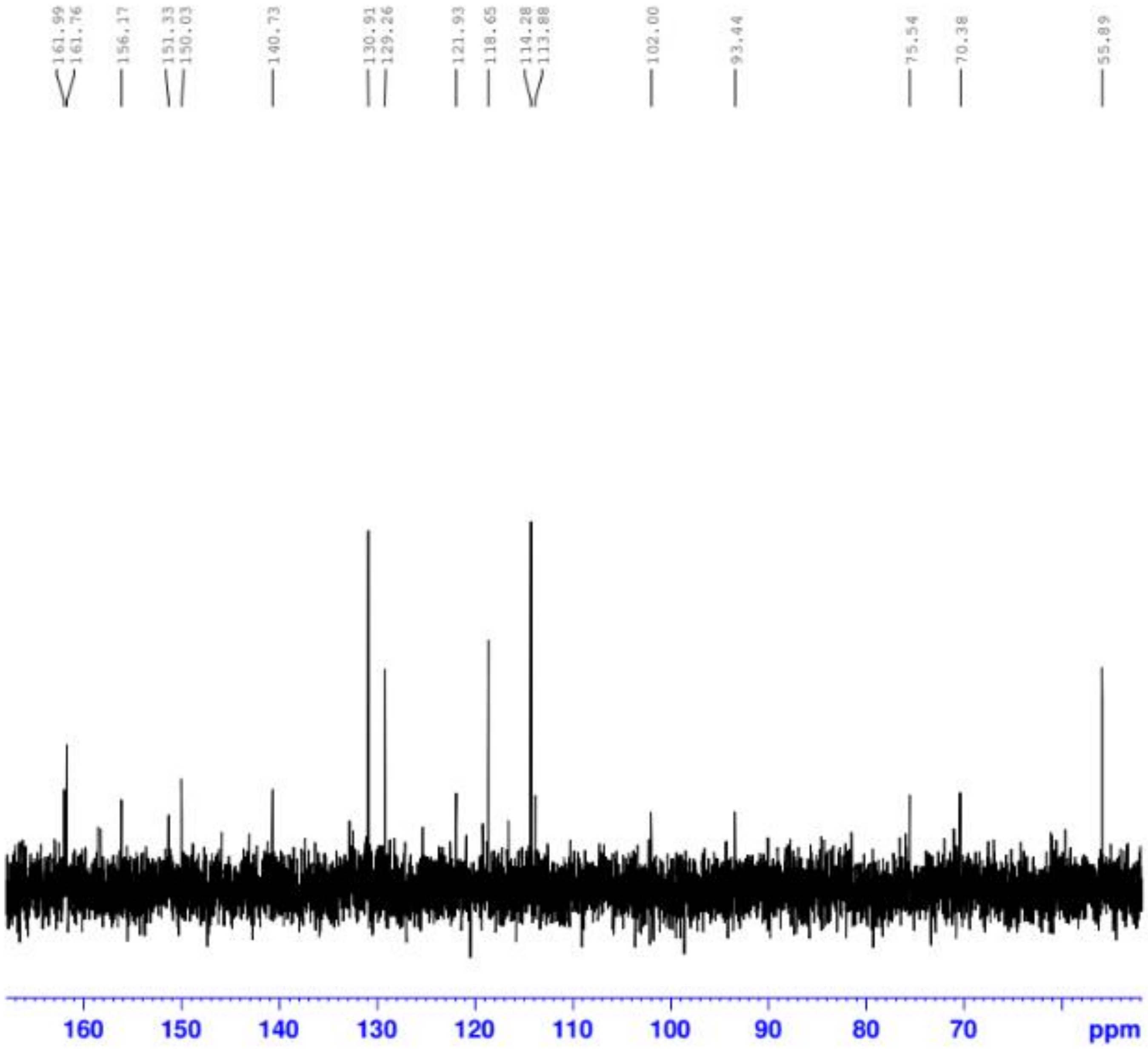

BRUKER

Current Data Parameters
NAME mohammed-attia-19c
EXPNO 2
PROCNO 1

F2 - Acquisition Parameters
Date_ 20210624
Time 13.50
INSTRUM spect
PROBHD 5 mm PABBO BB/
PULPROG zgpg30
TD 65536
SOLVENT DMSO
NS 1505
DS 4
SWH 24038.461 Hz
FIDRES 0.366798 Hz
AQ 1.3631488 sec
RG 205.37
DW 20.800 usec
DE 6.50 usec
TE 300.0 K
D1 2.00000000 sec
D11 0.03000000 sec
TD0 1

======== CHANNEL f1 ========
SFO1 100.6278588 MHz
NUC1 13C
P1 10.00 usec
PLW1 47.00000000 W

======== CHANNEL f2 ========
SFO2 400.1516006 MHz
NUC2 1H
CPDPRG[2 waltz16
PCPD2 90.00 usec
PLW2 18.00000000 W
PLW12 0.34722000 W
PLW13 0.28125000 W

F2 - Processing parameters
SI 32768
SF 100.6177975 MHz
WDW EM
SSB 0
LB 1.00 Hz
GB 0
PC 1.40

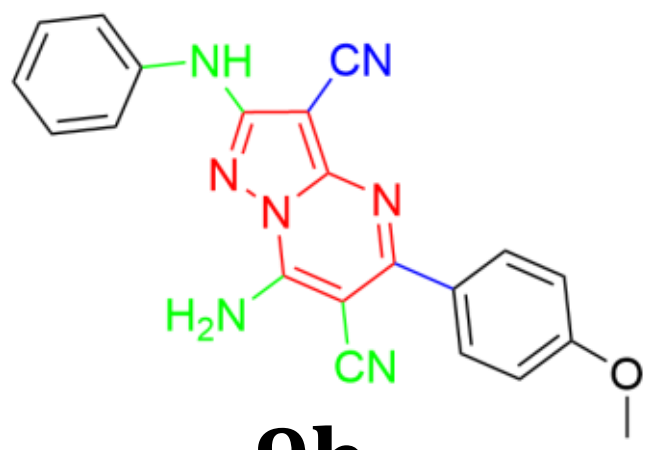
NH
CN
N
N
N
H2N
CN
O

9b

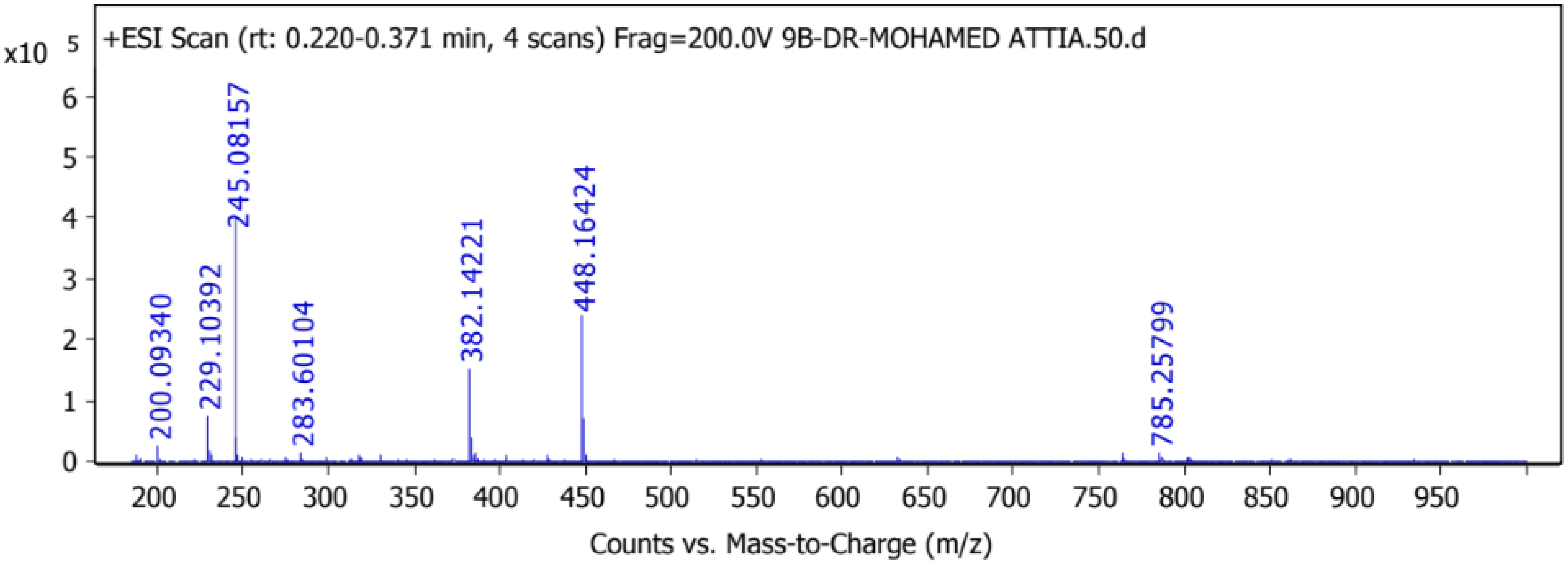
+ESI Scan (rt: 0.220-0.371 min, 4 scans) Frag=200.0V 9B-DR-MOHAMED ATTIA.50.d
x10 5
6
5
4
3
2
1
0
200.09340
229.10392
245.08157
283.60104
382.14221
448.16424
785.25799
200
250
300
350
400
450
500
550
600
650
700
750
800
850
900
950
Counts vs. Mass-to-Charge (m/z)

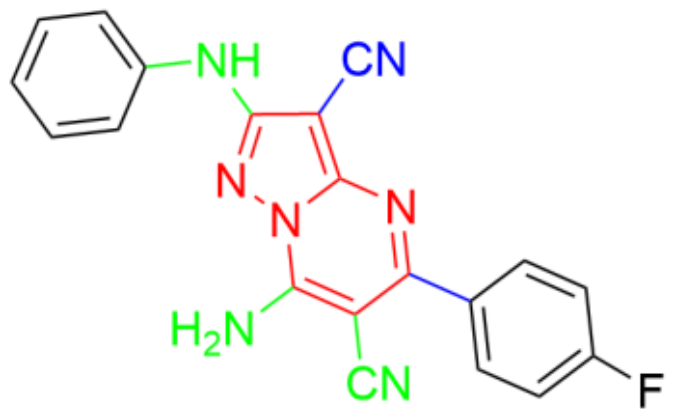

9c

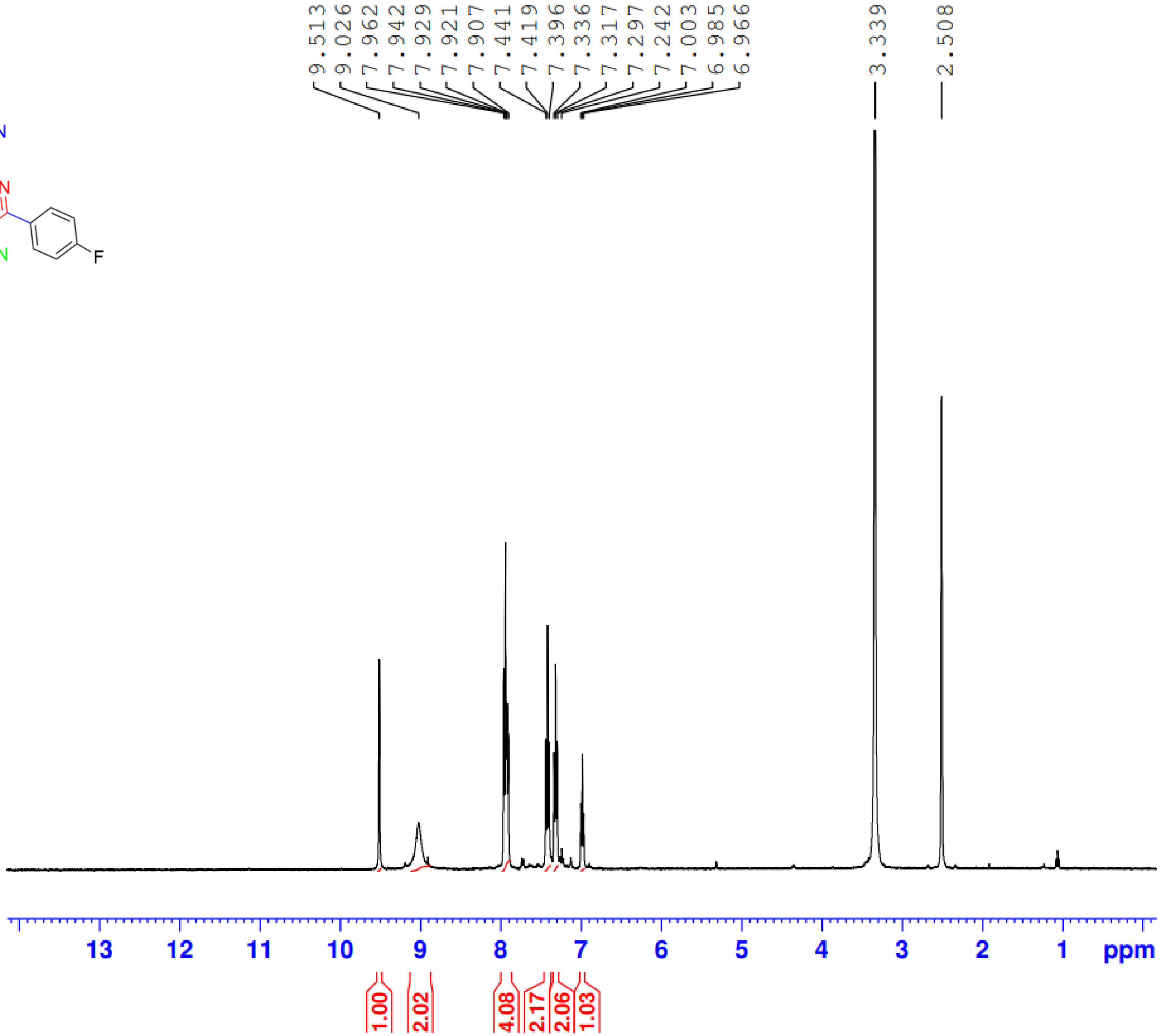


```
Current Data Parameters
NAME      mohammed-attia-19e
EXPNO                      1
PROCNO                     1

F2 - Acquisition Parameters
Date_             20210524
Time                 14.52
INSTRUM              spect
PROBHD    5 mm PABBO BB/
PULPROG               zg30
TD                   65536
SOLVENT               DMSO
NS                      35
DS                       2
SWH            8012.820 Hz
FIDRES         0.122266 Hz
AQ            4.0894465 sec
RG                  205.37
DW              62.400 usec
DE                6.50 usec
TE                 300.0 K
D1           1.00000000 sec
TD0                      1

======== CHANNEL f1 ========
SFO1        400.1524711 MHz
NUC1                    1H
P1               12.00 usec
PLW1        18.00000000 W

F2 - Processing parameters
SI                   65536
SF          400.1500000 MHz
WDW                     EM
SSB       0
LB                 0.30 Hz
GB        0
PC                    1.00
```

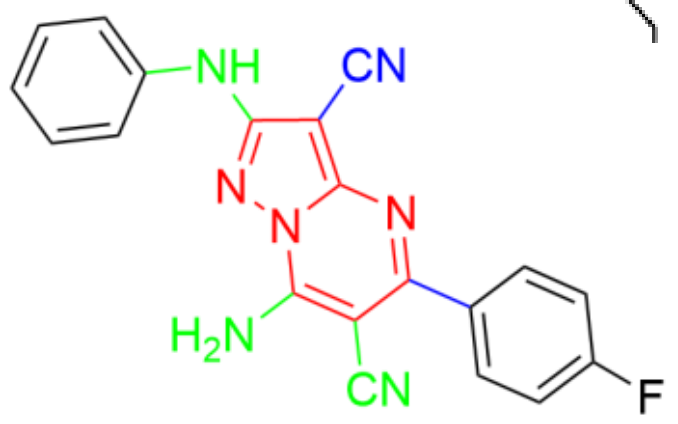

9c

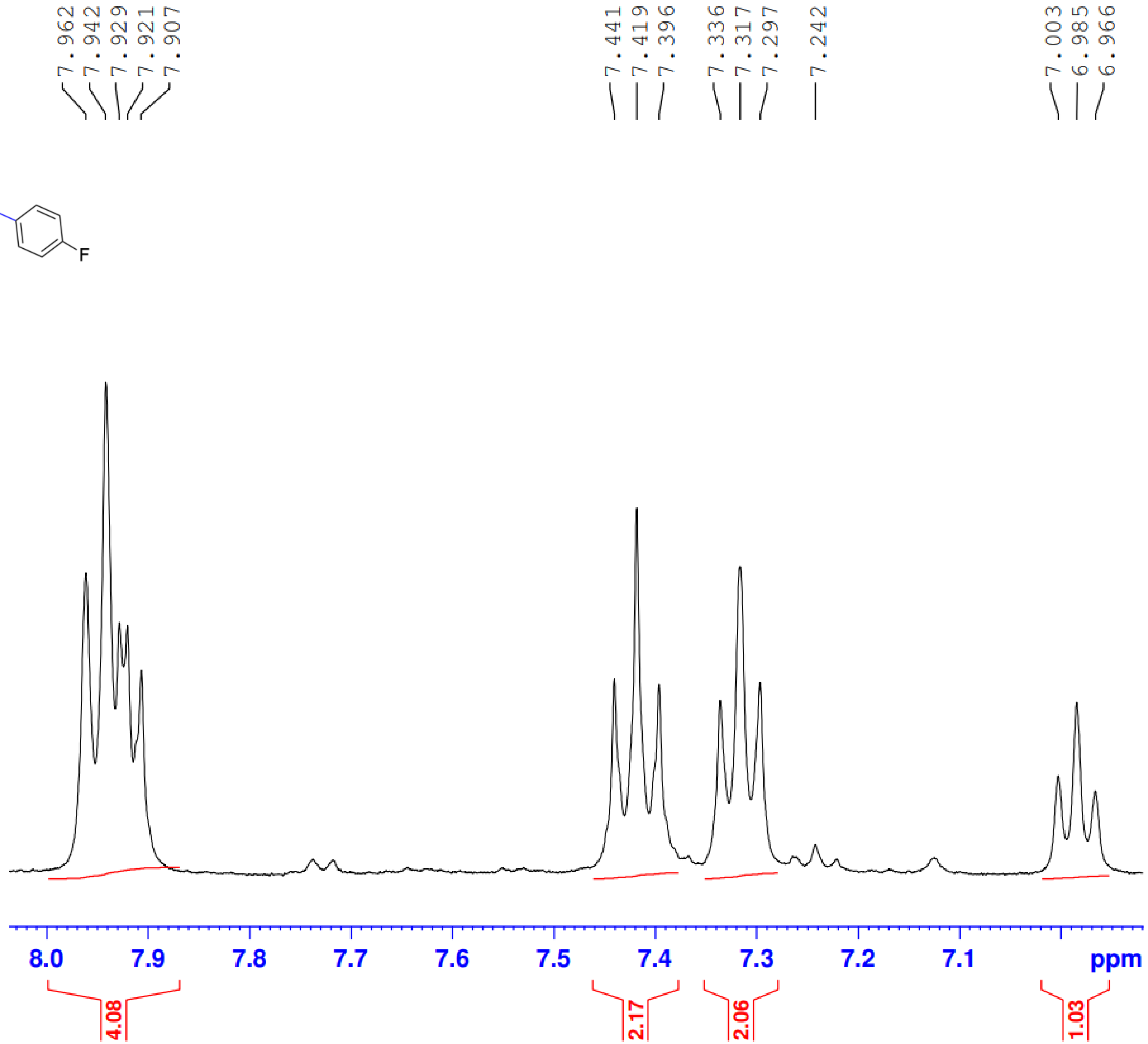

BRUKER

Current Data Parameters
NAME mohammed-attia-19e
EXPNO 1
PROCNO 1

F2 - Acquisition Parameters
Date_ 20210524
Time 14.52
INSTRUM spect
PROBHD 5 mm PABBO BB/
PULPROG zg30
TD 65536
SOLVENT DMSO
NS 35
DS 2
SWH 8012.820 Hz
FIDRES 0.122266 Hz
AQ 4.0894465 sec
RG 205.37
DW 62.400 usec
DE 6.50 usec
TE 300.0 K
D1 1.00000000 sec
TD0 1

======== CHANNEL f1 ========
SFO1 400.1524711 MHz
NUC1 1H
P1 12.00 usec
PLW1 18.00000000 W

F2 - Processing parameters
SI 65536
SF 400.1500000 MHz
WDW EM
SSB 0
LB 0.30 Hz
GB 0
PC 1.00

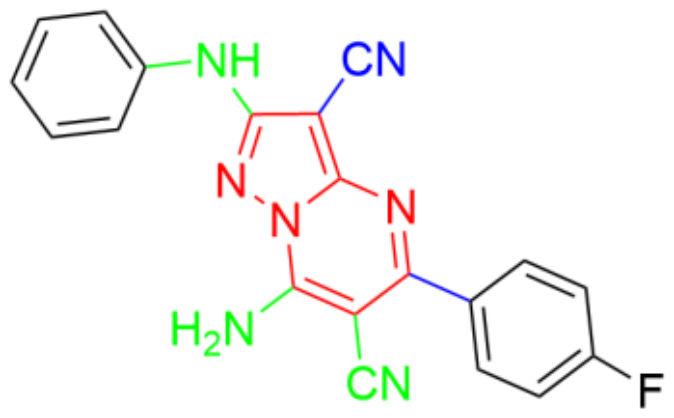

9c

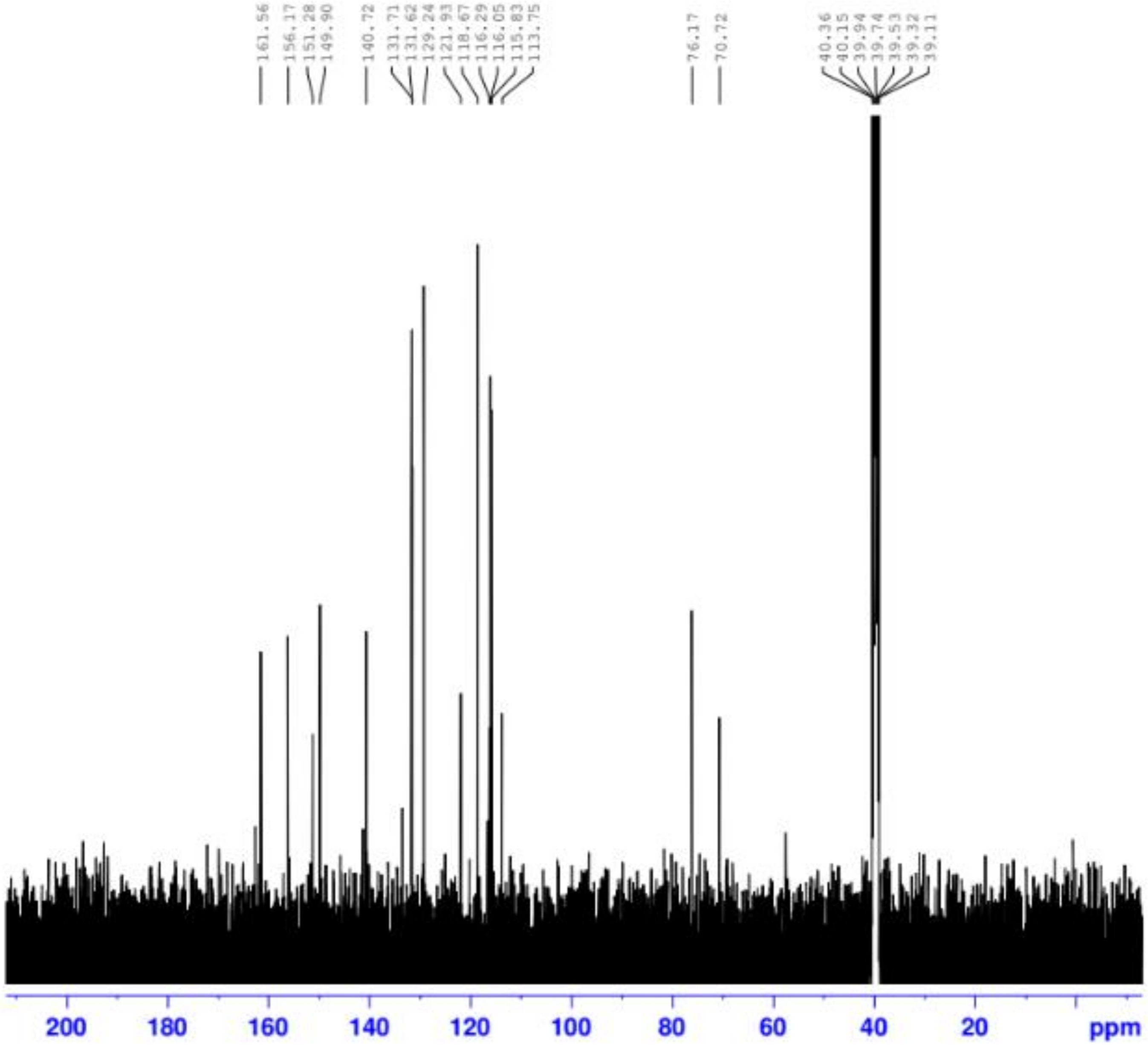

Current Data Parameters
NAME mohammed-attia-19e
EXPNO 2
PROCNO 1

F2 - Acquisition Parameters
Date_ 20210711
Time 11.59
INSTRUM spect
PROBHD 5 mm PABBO BB/
PULPROG zgpg30
TD 65536
SOLVENT DMSO
NS 1993
DS 4
SWH 24038.461 Hz
FIDRES 0.366798 Hz
AQ 1.3631488 sec
RG 205.37
DW 20.800 usec
DE 6.50 usec
TE 300.0 K
D1 2.00000000 sec
D11 0.03000000 sec
TD0 1

======== CHANNEL f1 ========
SFO1 100.6278588 MHz
NUC1 13C
P1 10.00 usec
PLW1 47.00000000 W

======== CHANNEL f2 ========
SFO2 400.1516006 MHz
NUC2 1H
CPDPRG[2 waltz16
PCPD2 90.00 usec
PLW2 18.00000000 W
PLW12 0.34722000 W
PLW13 0.28125000 W

F2 - Processing parameters
SI 32768
SF 100.6177975 MHz
WDW EM
SSB 0
LB 1.00 Hz
GB 0
PC 1.40

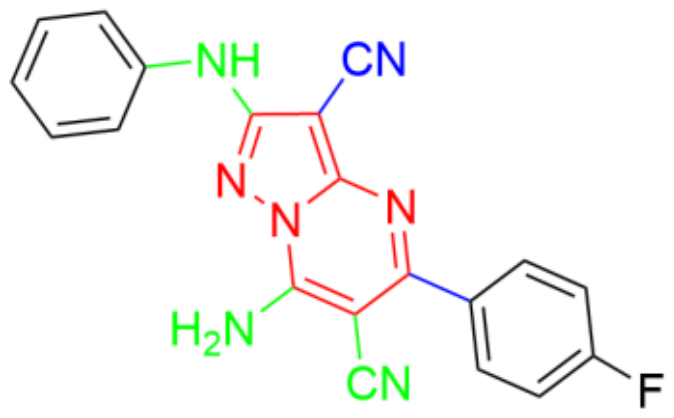

9c

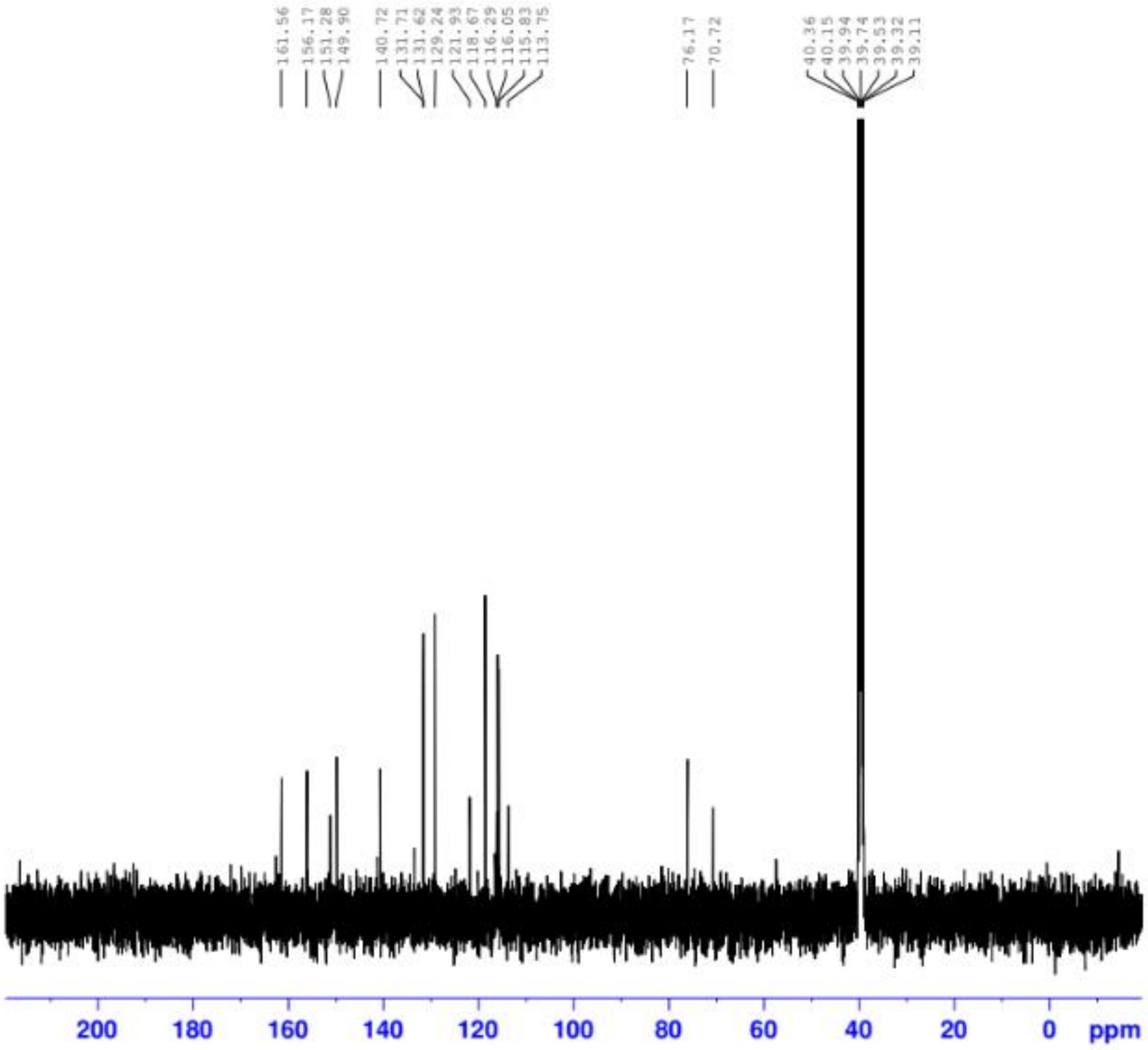


```
Current Data Parameters
NAME        mohammed-attia-19e
EXPNO                        2
PROCNO                       1

F2 - Acquisition Parameters
Date_               20210711
Time                   11.59
INSTRUM                spect
PROBHD     5 mm PABBO BB/
PULPROG              zgpg30
TD                    65536
SOLVENT                DMSO
NS                     1993
DS                        4
SWH              24038.461 Hz
FIDRES            0.366798 Hz
AQ               1.3631488 sec
RG                   205.37
DW                   20.800 usec
DE                     6.50 usec
TE                    300.0 K
D1               2.00000000 sec
D11              0.03000000 sec
TD0                       1

======== CHANNEL f1 ========
SFO1          100.6278588 MHz
NUC1                  13C
P1                  10.00 usec
PLW1          47.00000000 W

======== CHANNEL f2 ========
SFO2          400.1516006 MHz
NUC2                   1H
CPDPRG[2           waltz16
PCPD2               90.00 usec
PLW2          18.00000000 W
PLW12          0.34722000 W
PLW13          0.28125000 W

F2 - Processing parameters
SI                  32768
SF            100.6177975 MHz
WDW                    EM
SSB      0
LB                   1.00 Hz
GB       0
PC                   1.40
```

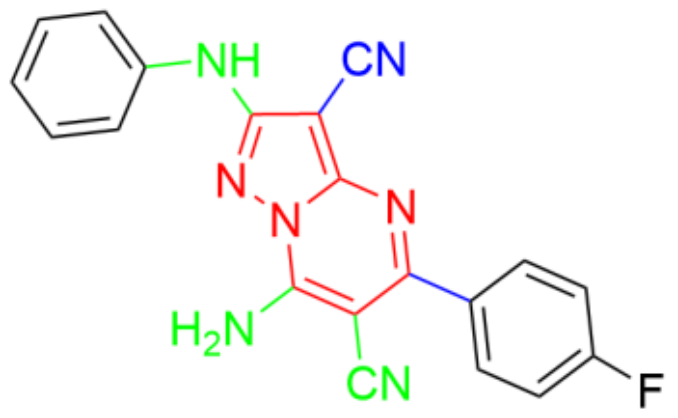

9c

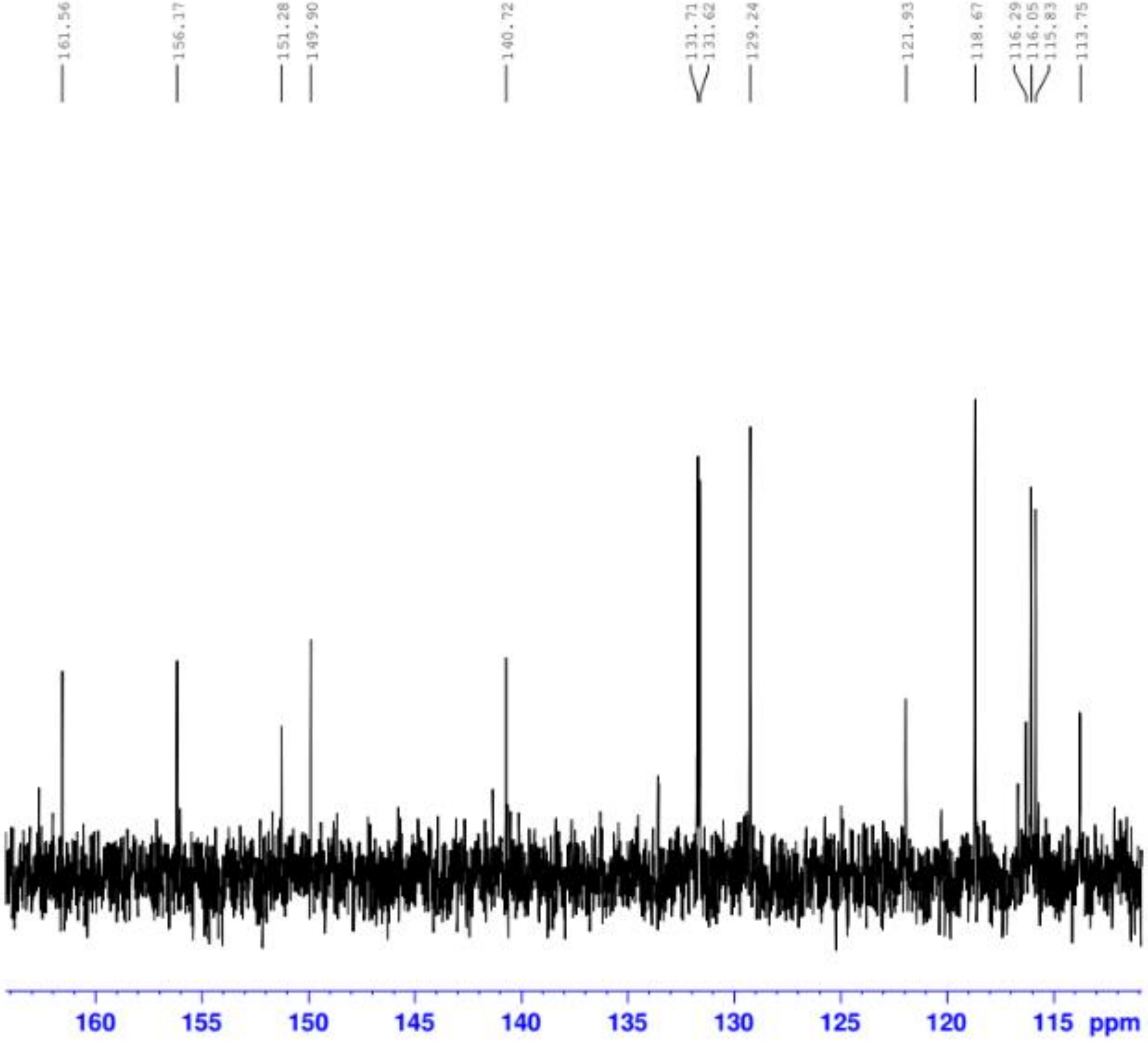

BRUKER

```
Current Data Parameters
NAME        mohammed-attia-19e
EXPNO                        2
PROCNO                       1

F2 - Acquisition Parameters
Date_                 20210711
Time                     11.59
INSTRUM                  spect
PROBHD      5 mm PABBO BB/
PULPROG                zgpg30
TD                       65536
SOLVENT                   DMSO
NS                        1993
DS                           4
SWH                  24038.461 Hz
FIDRES                0.366798 Hz
AQ                   1.3631488 sec
RG                      205.37
DW                      20.800 usec
DE                        6.50 usec
TE                       300.0 K
D1                  2.00000000 sec
D11                 0.03000000 sec
TD0                          1

======== CHANNEL f1 ========
SFO1               100.6278588 MHz
NUC1                       13C
P1                       10.00 usec
PLW1               47.00000000 W

======== CHANNEL f2 ========
SFO2               400.1516006 MHz
NUC2                        1H
CPDPRG[2               waltz16
PCPD2                    90.00 usec
PLW2               18.00000000 W
PLW12               0.34722000 W
PLW13               0.28125000 W

F2 - Processing parameters
SI                       32768
SF                 100.6177975 MHz
WDW                         EM
SSB      0
LB                        1.00 Hz
GB       0
PC                        1.40
```

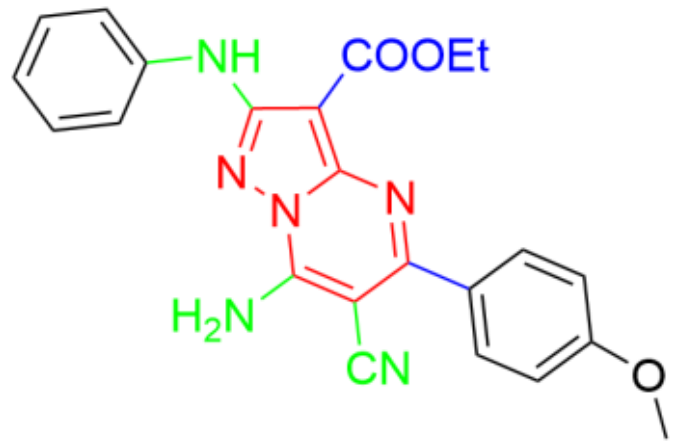

9d

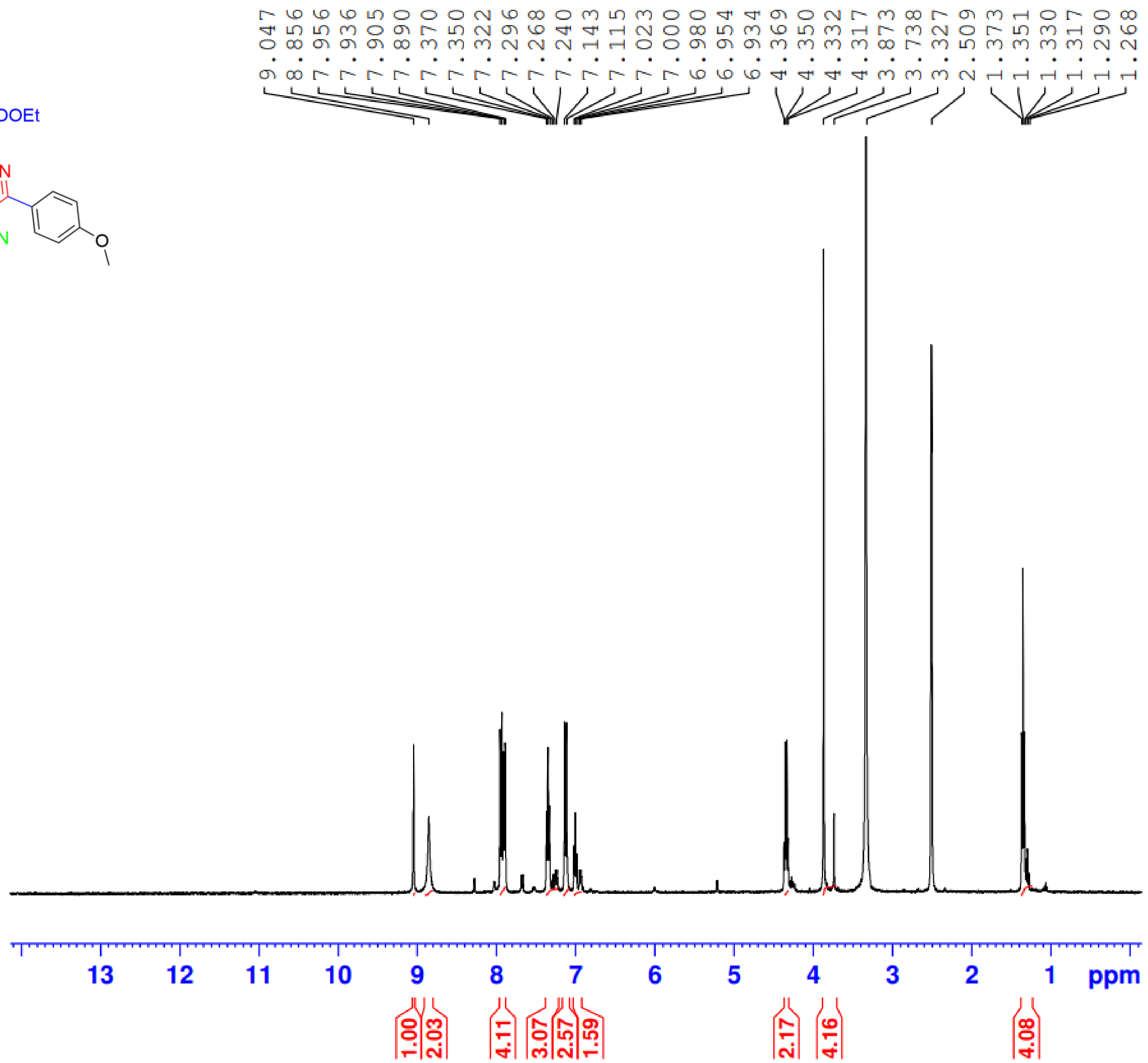


```
Current Data Parameters
NAME      mohammed-attia-23c
EXPNO                      1
PROCNO                     1

F2 - Acquisition Parameters
Date_             20210524
Time                 14.28
INSTRUM              spect
PROBHD   5 mm PABBO BB/
PULPROG               zg30
TD                   65536
SOLVENT               DMSO
NS                      26
DS                       2
SWH               8012.820 Hz
FIDRES            0.122266 Hz
AQ               4.0894465 sec
RG                  205.37
DW                  62.400 usec
DE                    6.50 usec
TE                   300.0 K
D1              1.00000000 sec
TD0                      1

======== CHANNEL f1 ========
SFO1           400.1524711 MHz
NUC1                    1H
P1                   12.00 usec
PLW1           18.00000000 W

F2 - Processing parameters
SI                   65536
SF             400.1500000 MHz
WDW                     EM
SSB       0
LB                    0.30 Hz
GB        0
PC                    1.00
```

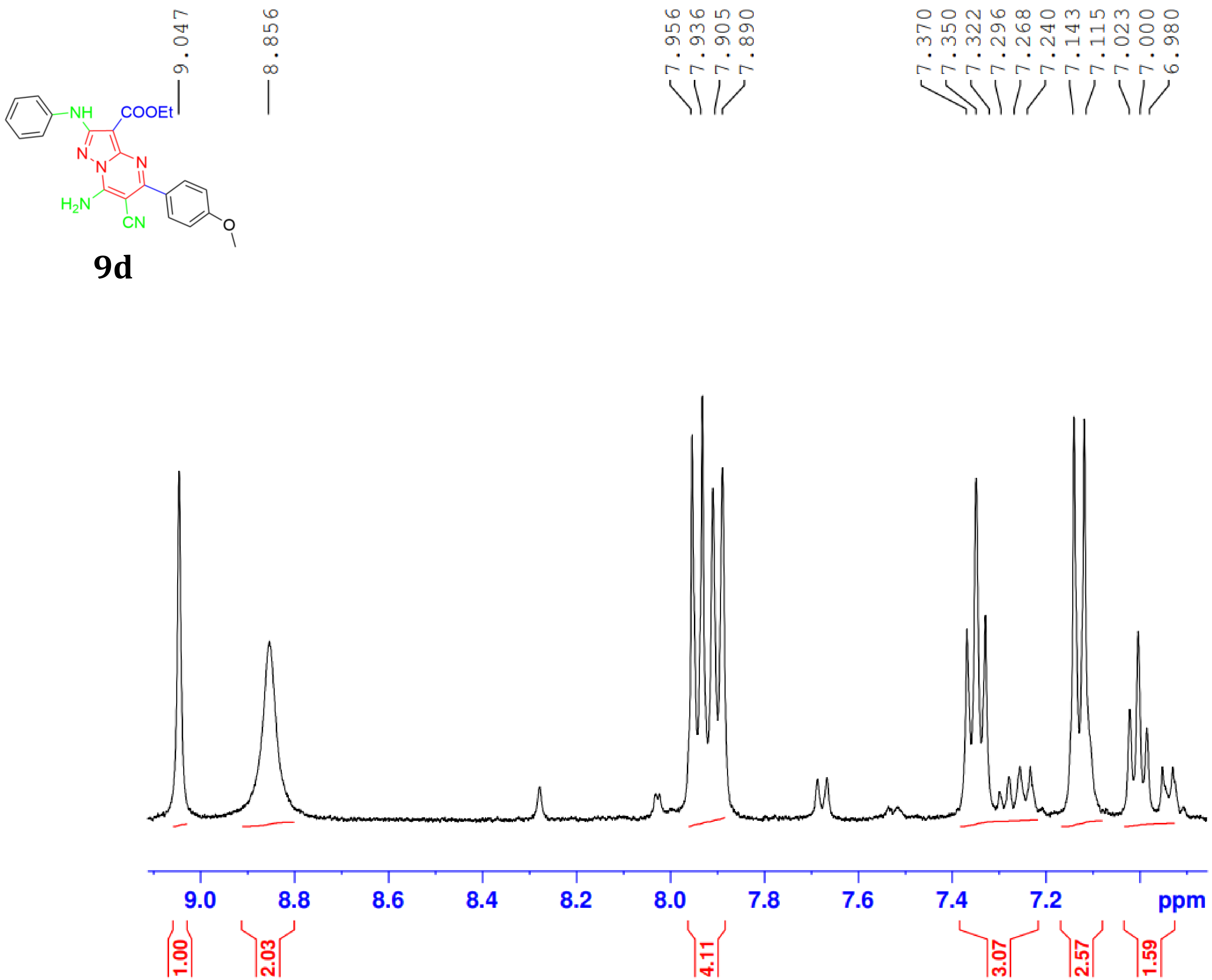


```
Current Data Parameters
NAME      mohammed-attia-23c
EXPNO                    1
PROCNO                   1

F2 - Acquisition Parameters
Date_             20210524
Time                 14.28
INSTRUM              spect
PROBHD    5 mm PABBO BB/
PULPROG               zg30
TD                   65536
SOLVENT               DMSO
NS                      26
DS                       2
SWH             8012.820 Hz
FIDRES          0.122266 Hz
AQ             4.0894465 sec
RG                  205.37
DW                  62.400 usec
DE                    6.50 usec
TE                   300.0 K
D1            1.00000000 sec
TD0                      1

======== CHANNEL f1 ========
SFO1         400.1524711 MHz
NUC1                    1H
P1                   12.00 usec
PLW1         18.00000000 W

F2 - Processing parameters
SI                   65536
SF           400.1500000 MHz
WDW                     EM
SSB       0
LB                    0.30 Hz
GB        0
PC                    1.00
```

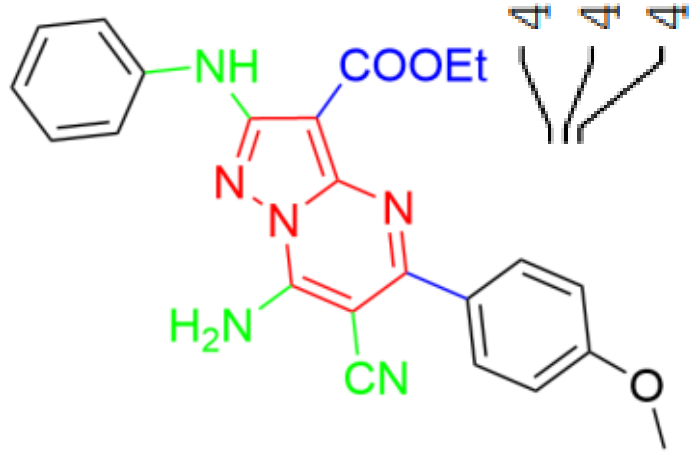

9d

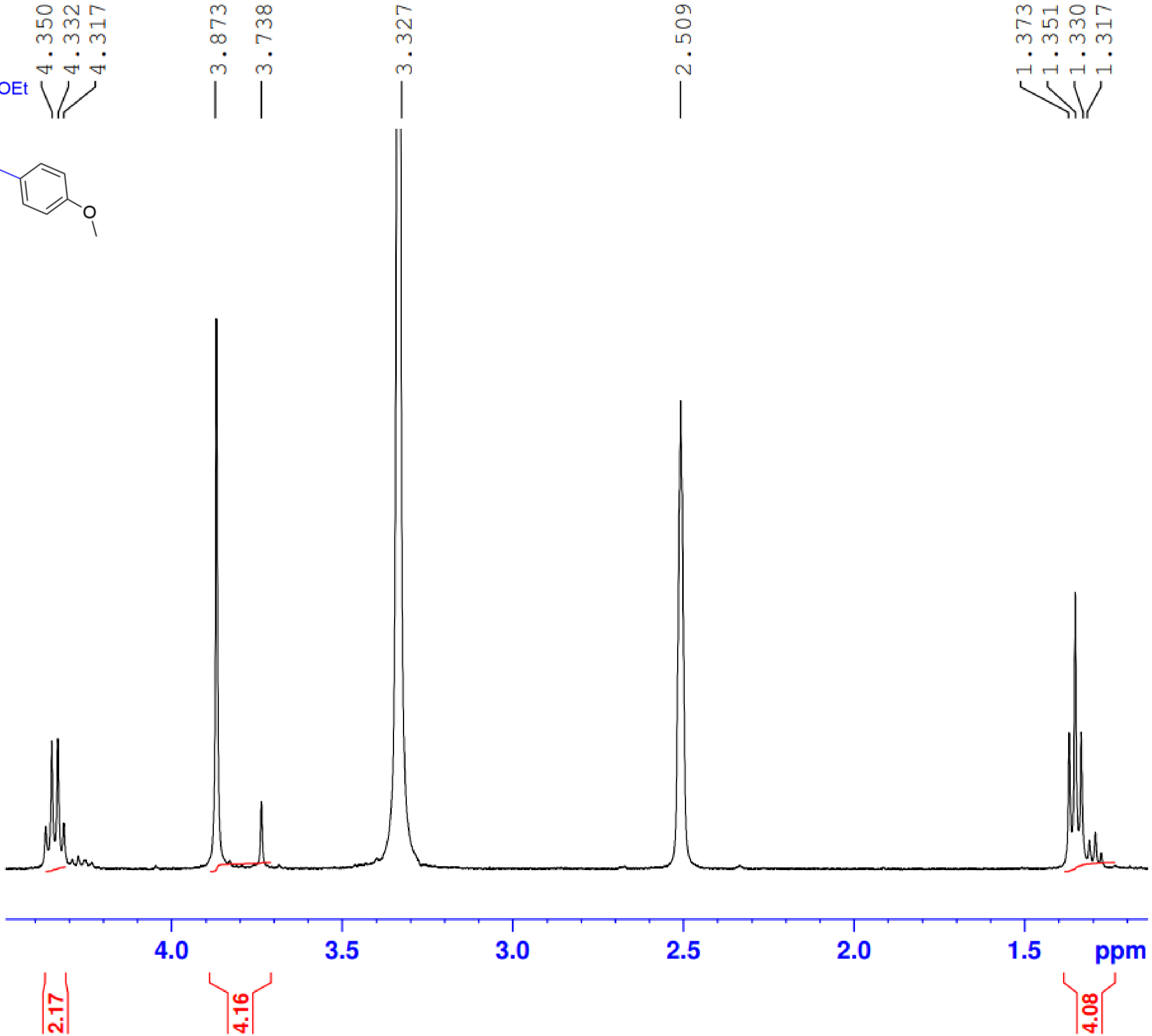


```
Current Data Parameters
NAME      mohammed-attia-23c
EXPNO                    1
PROCNO                   1

F2 - Acquisition Parameters
Date_             20210524
Time                 14.28
INSTRUM              spect
PROBHD    5 mm PABBO BB/
PULPROG               zg30
TD                   65536
SOLVENT               DMSO
NS                      26
DS                       2
SWH               8012.820 Hz
FIDRES            0.122266 Hz
AQ               4.0894465 sec
RG                  205.37
DW                  62.400 usec
DE                    6.50 usec
TE                   300.0 K
D1              1.00000000 sec
TD0                      1

======== CHANNEL f1 ========
SFO1        400.1524711 MHz
NUC1                 1H
P1                12.00 usec
PLW1        18.00000000 W

F2 - Processing parameters
SI                   65536
SF           400.1500000 MHz
WDW                     EM
SSB      0
LB                    0.30 Hz
GB       0
PC                    1.00
```

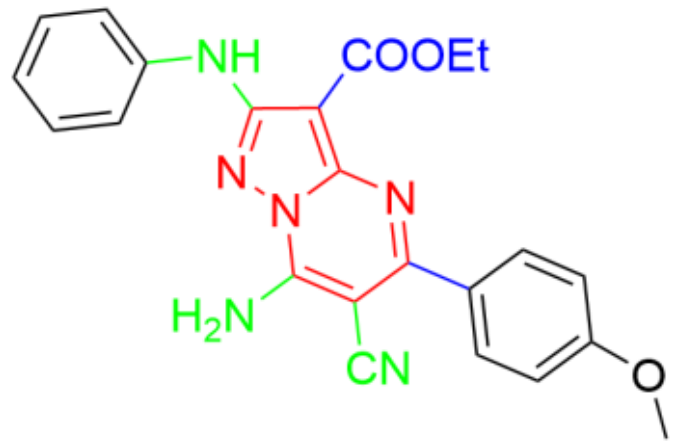

9d

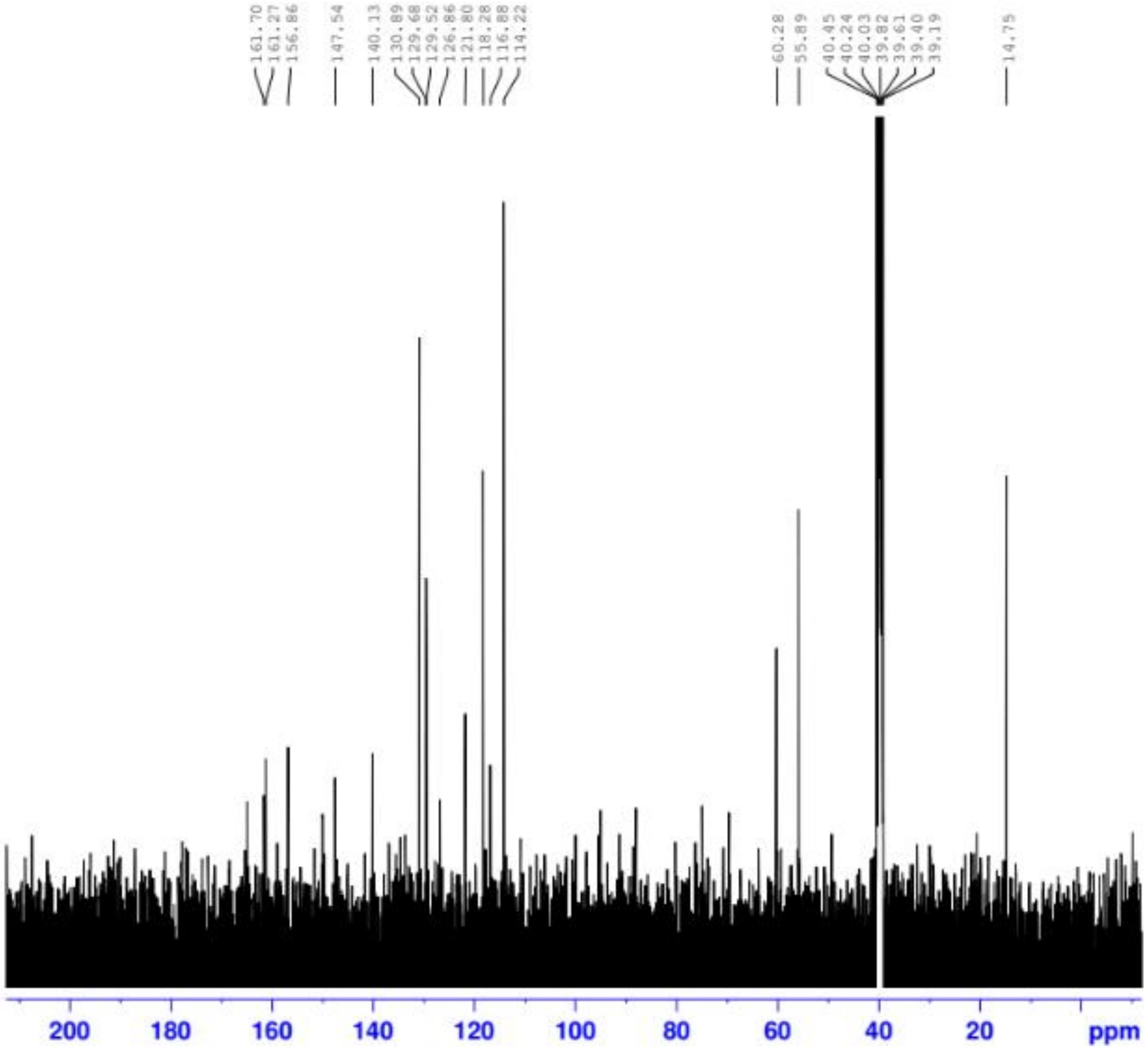

Current Data Parameters
NAME mohammed-attia-23c
EXPNO 2
PROCNO 1

F2 - Acquisition Parameters
Date_ 20210817
Time 10.48
INSTRUM spect
PROBHD 5 mm PABBO BB/
PULPROG zgpg30
TD 65536
SOLVENT DMSO
NS 1024
DS 4
SWH 24038.461 Hz
FIDRES 0.366798 Hz
AQ 1.3631488 sec
RG 205.37
DW 20.800 usec
DE 6.50 usec
TE 300.0 K
D1 2.00000000 sec
D11 0.03000000 sec
TD0 1

======== CHANNEL f1 ========
SFO1 100.6278588 MHz
NUC1 13C
P1 10.00 usec
PLW1 47.00000000 W

======== CHANNEL f2 ========
SFO2 400.1516006 MHz
NUC2 1H
CPDPRG[2 waltz16
PCPD2 90.00 usec
PLW2 18.00000000 W
PLW12 0.34722000 W
PLW13 0.28125000 W

F2 - Processing parameters
SI 32768
SF 100.6177975 MHz
WDW EM
SSB 0
LB 1.00 Hz
GB 0
PC 1.40

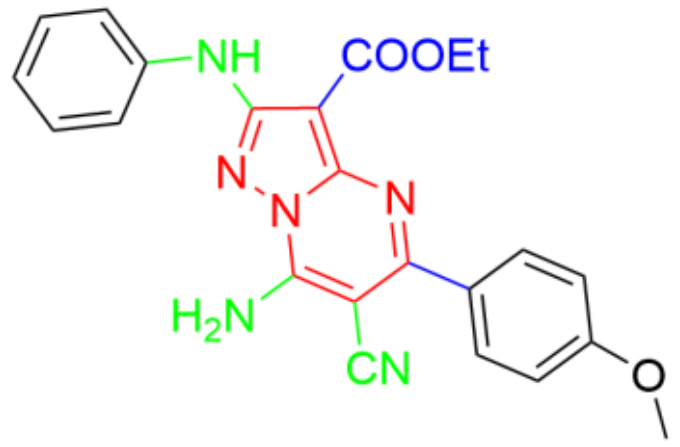

9d

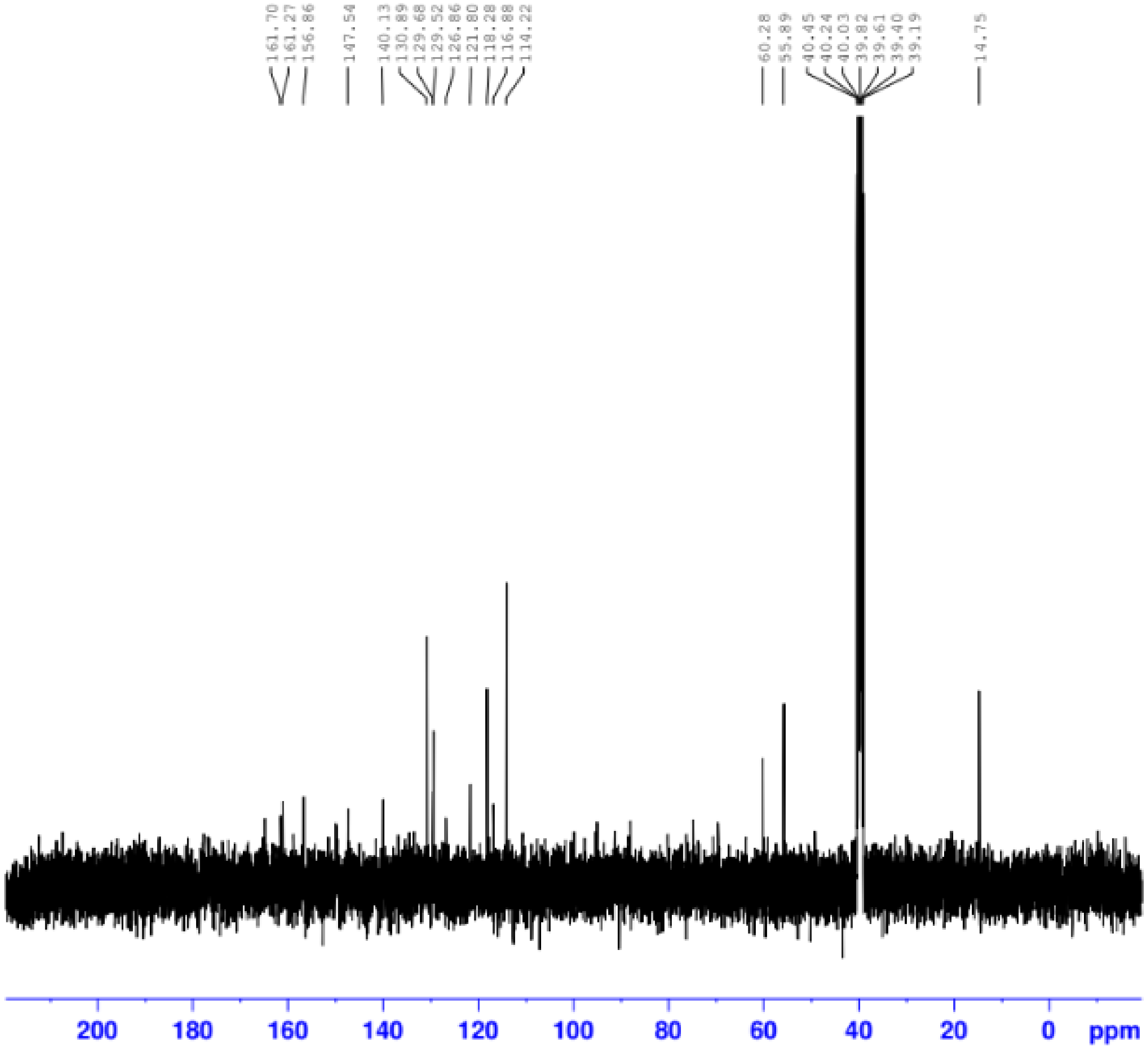

Current Data Parameters
NAME mohammed-attia-23c
EXPNO 2
PROCNO 1

F2 - Acquisition Parameters
Date_ 20210817
Time 10.48
INSTRUM spect
PROBHD 5 mm PABBO BB/
PULPROG zgpg30
TD 65536
SOLVENT DMSO
NS 1024
DS 4
SWH 24038.461 Hz
FIDRES 0.366798 Hz
AQ 1.3631488 sec
RG 205.37
DW 20.800 usec
DE 6.50 usec
TE 300.0 K
D1 2.00000000 sec
D11 0.03000000 sec
TD0 1

======== CHANNEL f1 ========
SFO1 100.6278588 MHz
NUC1 13C
P1 10.00 usec
PLW1 47.00000000 W

======== CHANNEL f2 ========
SFO2 400.1516006 MHz
NUC2 1H
CPDPRG[2 waltz16
PCPD2 90.00 usec
PLW2 18.00000000 W
PLW12 0.34722000 W
PLW13 0.28125000 W

F2 - Processing parameters
SI 32768
SF 100.6177975 MHz
WDW EM
SSB 0
LB 1.00 Hz
GB 0
PC 1.40

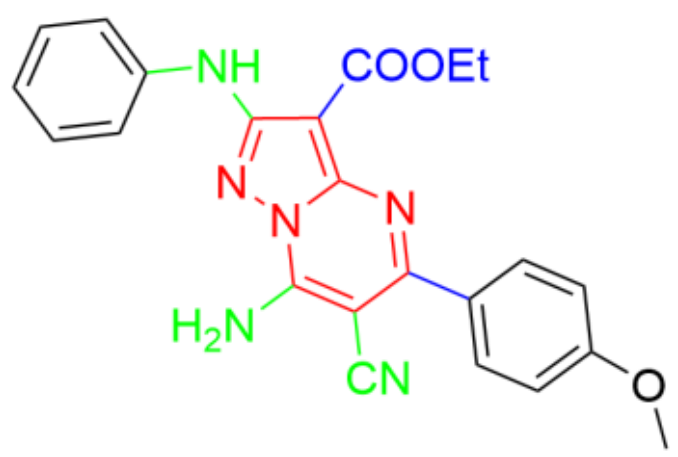

9d

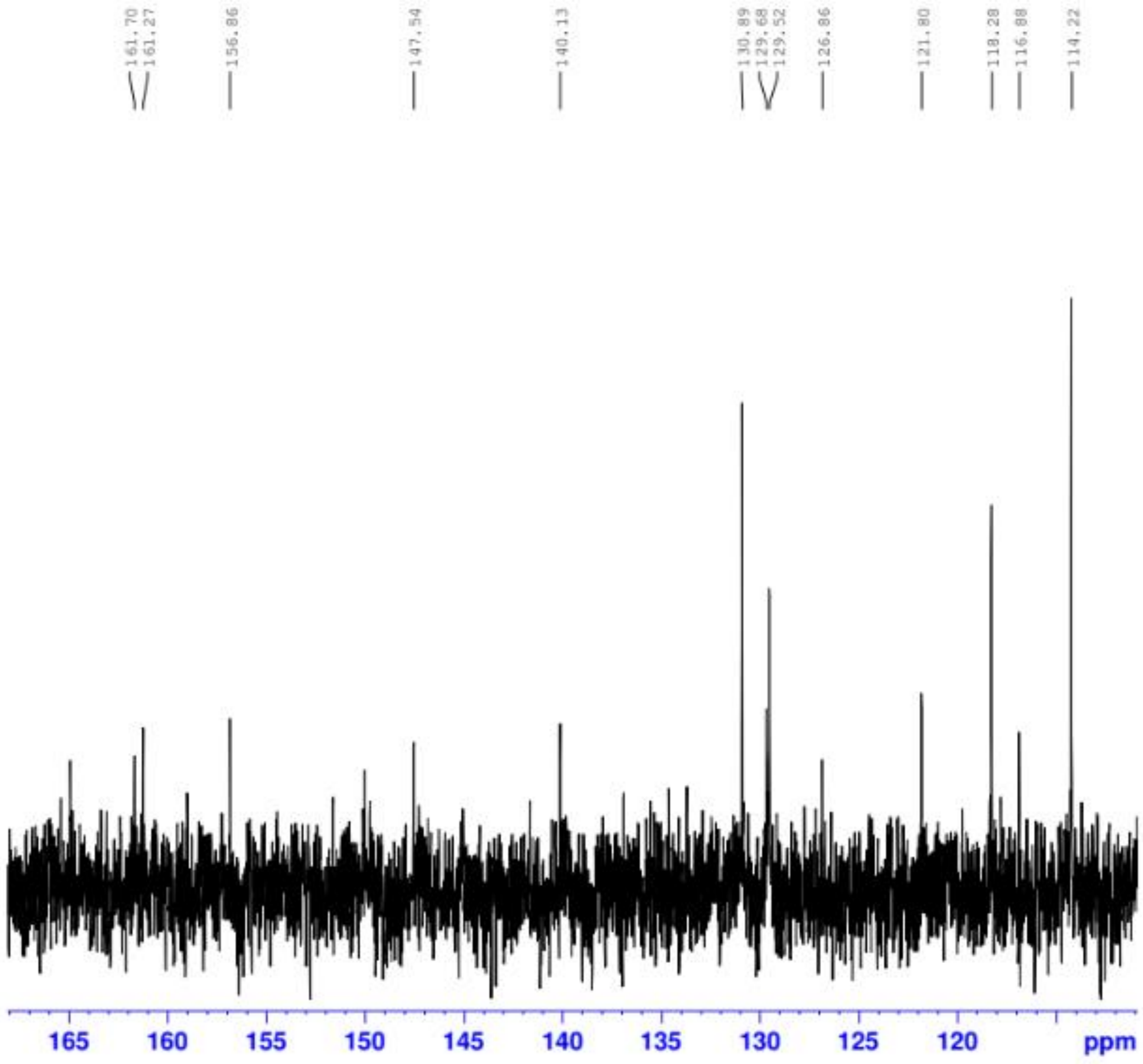


```
Current Data Parameters
NAME      mohammed-attia-23c
EXPNO                     2
PROCNO                    1

F2 - Acquisition Parameters
Date_              20210817
Time                  10.48
INSTRUM               spect
PROBHD    5 mm PABBO BB/
PULPROG             zgpg30
TD                   65536
SOLVENT               DMSO
NS                    1024
DS                       4
SWH              24038.461 Hz
FIDRES            0.366798 Hz
AQ               1.3631488 sec
RG                  205.37
DW                  20.800 usec
DE                    6.50 usec
TE                   300.0 K
D1              2.00000000 sec
D11             0.03000000 sec
TD0                      1

======== CHANNEL f1 ========
SFO1           100.6278588 MHz
NUC1                   13C
P1                   10.00 usec
PLW1           47.00000000 W

======== CHANNEL f2 ========
SFO2           400.1516006 MHz
NUC2                    1H
CPDPRG[2           waltz16
PCPD2                90.00 usec
PLW2           18.00000000 W
PLW12           0.34722000 W
PLW13           0.28125000 W

F2 - Processing parameters
SI                   32768
SF             100.6177975 MHz
WDW                     EM
SSB      0
LB                    1.00 Hz
GB       0
PC                    1.40
```

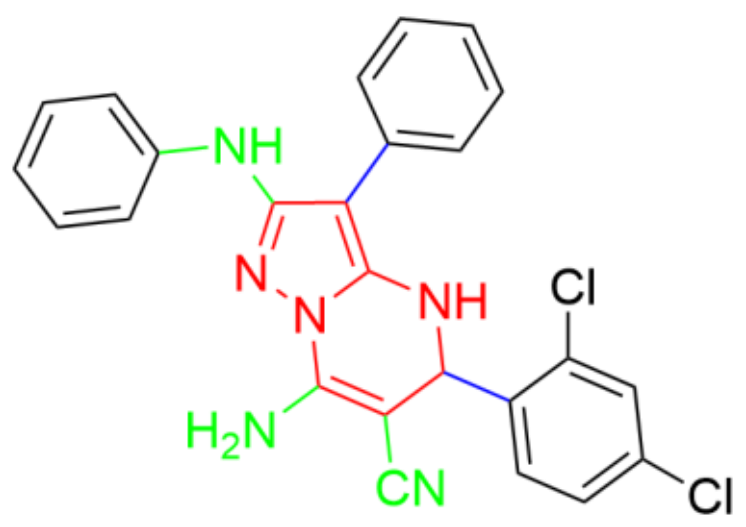

9e

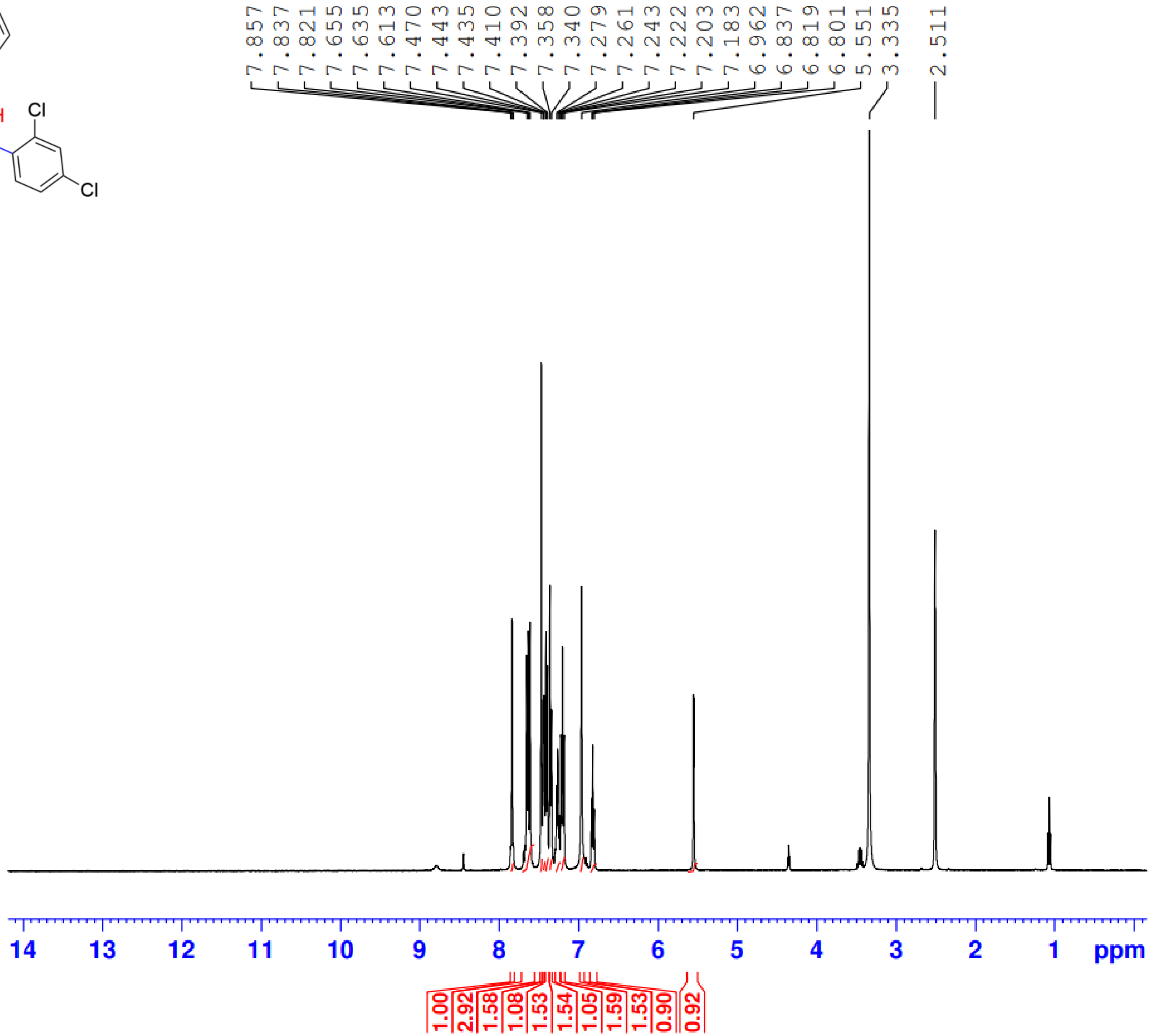

Current Data Parameters
NAME mohammed-attia-27g
EXPNO 1
PROCNO 1

F2 - Acquisition Parameters
Date_ 20210520
Time 15.53
INSTRUM spect
PROBHD 5 mm PABBO BB/
PULPROG zg30
TD 65536
SOLVENT DMSO
NS 64
DS 2
SWH 8012.820 Hz
FIDRES 0.122266 Hz
AQ 4.0894465 sec
RG 205.37
DW 62.400 usec
DE 6.50 usec
TE 300.0 K
D1 1.00000000 sec
TD0 1

======== CHANNEL f1 ========
SFO1 400.1524711 MHz
NUC1 1H
P1 12.00 usec
PLW1 18.00000000 W

F2 - Processing parameters
SI 65536
SF 400.1500000 MHz
WDW EM
SSB 0
LB 0.30 Hz
GB 0
PC 1.00

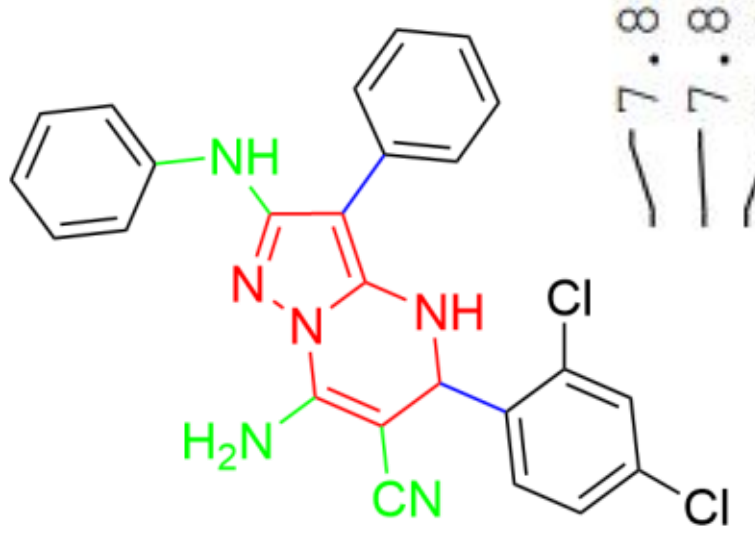
NH
N
N
NH
Cl
H2N
CN
Cl

9e

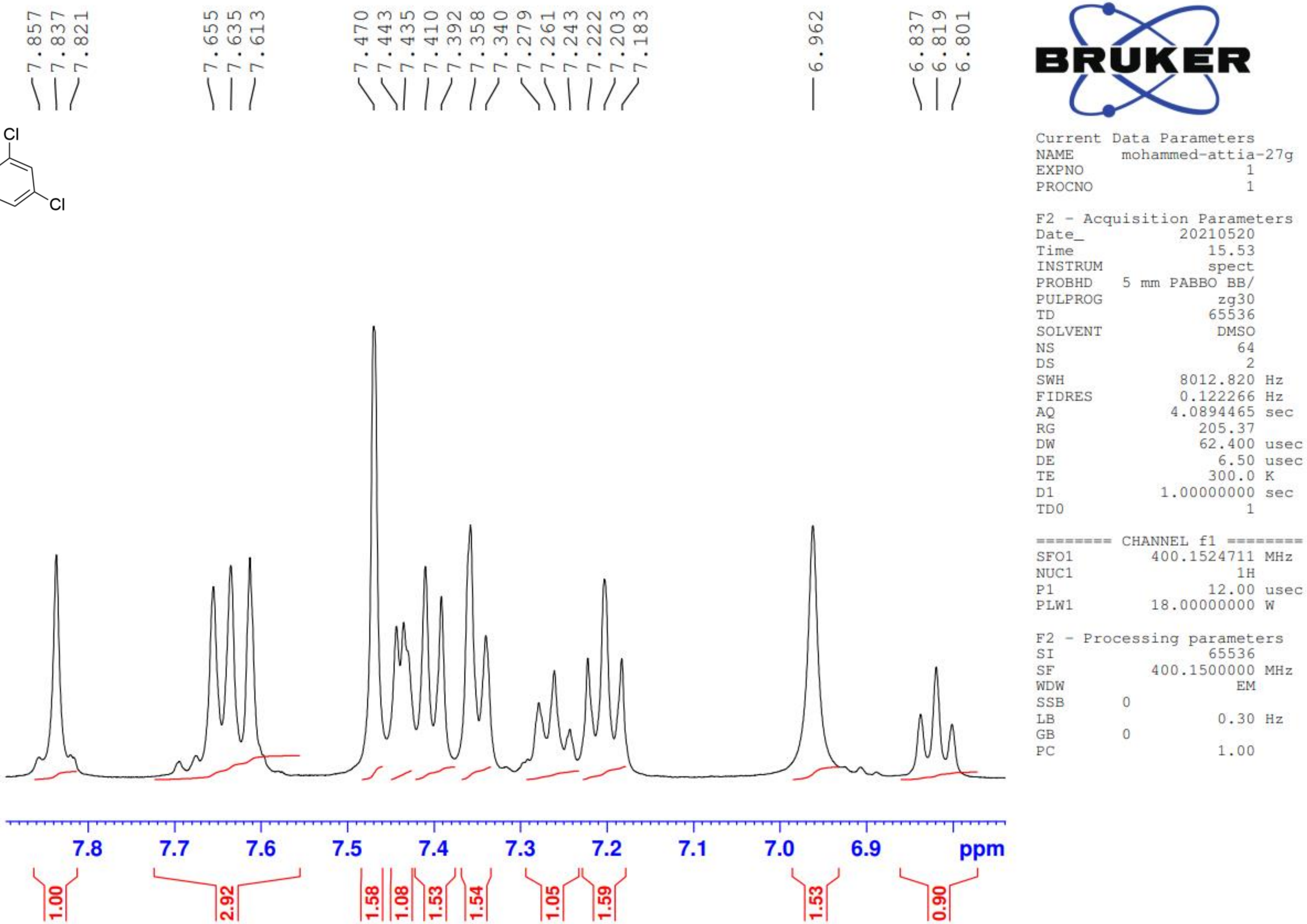
7.857
7.837
7.821
7.655
7.635
7.613
7.470
7.443
7.435
7.410
7.392
7.358
7.340
7.279
7.261
7.243
7.222
7.203
7.183
6.962
6.837
6.819
6.801
7.8
7.7
7.6
7.5
7.4
7.3
7.2
7.1
7.0
6.9
ppm
1.00
2.92
1.58
1.08
1.53
1.54
1.05
1.59
1.53
0.90
BRUKER
Current Data Parameters
NAME mohammed-attia-27g
EXPNO 1
PROCNO 1
F2 - Acquisition Parameters
Date_ 20210520
Time 15.53
INSTRUM spect
PROBHD 5 mm PABBO BB/
PULPROG zg30
TD 65536
SOLVENT DMSO
NS 64
DS 2
SWH 8012.820 Hz
FIDRES 0.122266 Hz
AQ 4.0894465 sec
RG 205.37
DW 62.400 usec
DE 6.50 usec
TE 300.0 K
D1 1.00000000 sec
TD0 1
======== CHANNEL f1 ========
SFO1 400.1524711 MHz
NUC1 1H
P1 12.00 usec
PLW1 18.00000000 W
F2 - Processing parameters
SI 65536
SF 400.1500000 MHz
WDW EM
SSB 0
LB 0.30 Hz
GB 0
PC 1.00

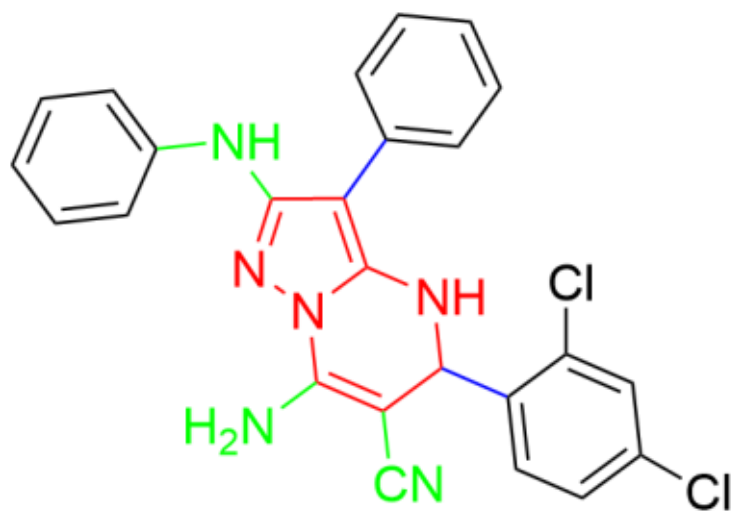

9e

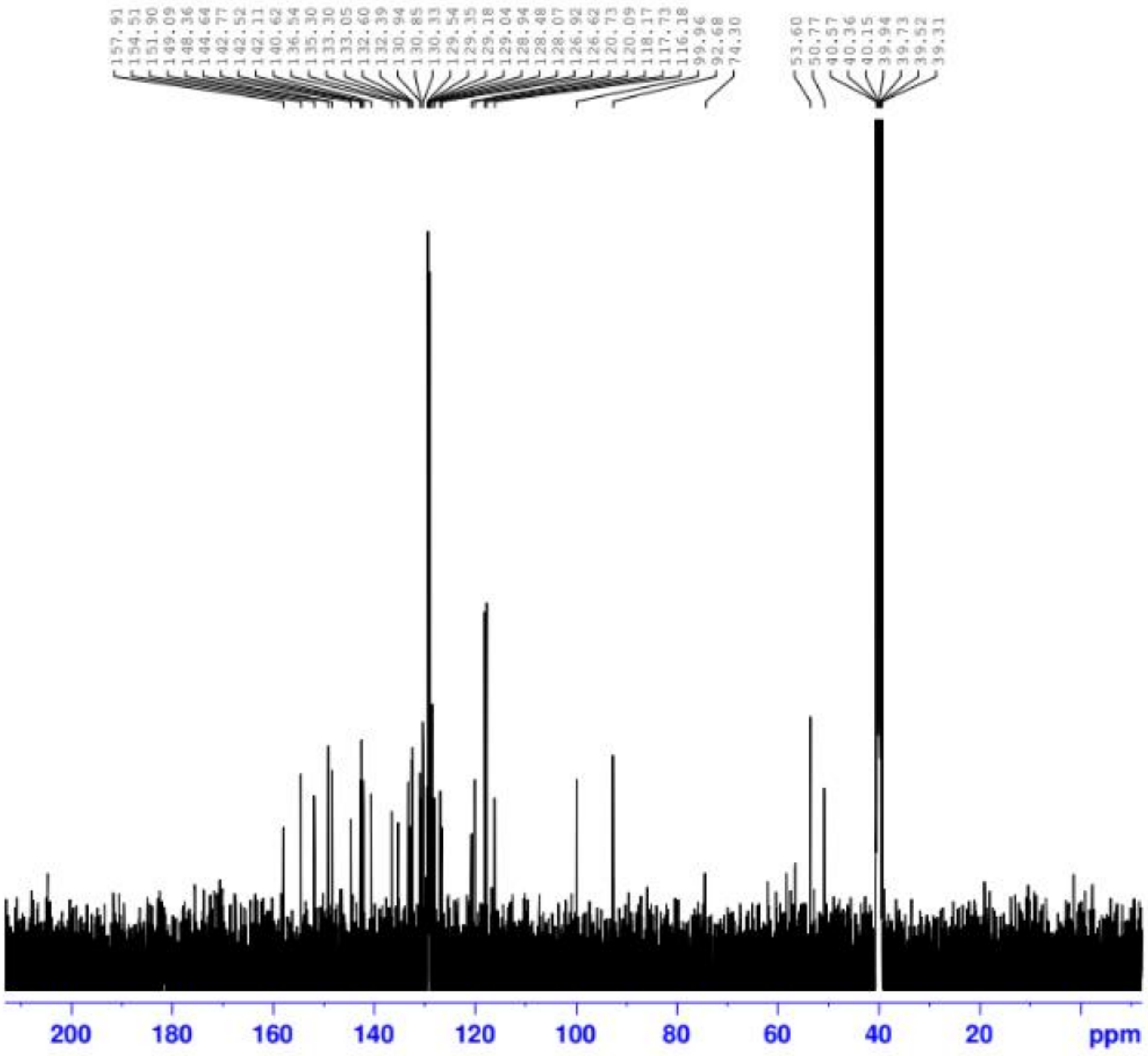

BRUKER

Current Data Parameters
NAME mohammed-attia-27g
EXPNO 2
PROCNO 1

F2 - Acquisition Parameters
Date_ 20210622
Time 10.54
INSTRUM spect
PROBHD 5 mm PABBO BB/
PULPROG zgpg30
TD 65536
SOLVENT DMSO
NS 1657
DS 4
SWH 24038.461 Hz
FIDRES 0.366798 Hz
AQ 1.3631488 sec
RG 205.37
DW 20.800 usec
DE 6.50 usec
TE 300.0 K
D1 2.00000000 sec
D11 0.03000000 sec
TD0 1

======== CHANNEL f1 ========
SFO1 100.6278588 MHz
NUC1 13C
P1 10.00 usec
PLW1 47.00000000 W

======== CHANNEL f2 ========
SFO2 400.1516006 MHz
NUC2 1H
CPDPRG[2 waltz16
PCPD2 90.00 usec
PLW2 18.00000000 W
PLW12 0.34722000 W
PLW13 0.28125000 W

F2 - Processing parameters
SI 32768
SF 100.6177975 MHz
WDW EM
SSB 0
LB 1.00 Hz
GB 0
PC 1.40

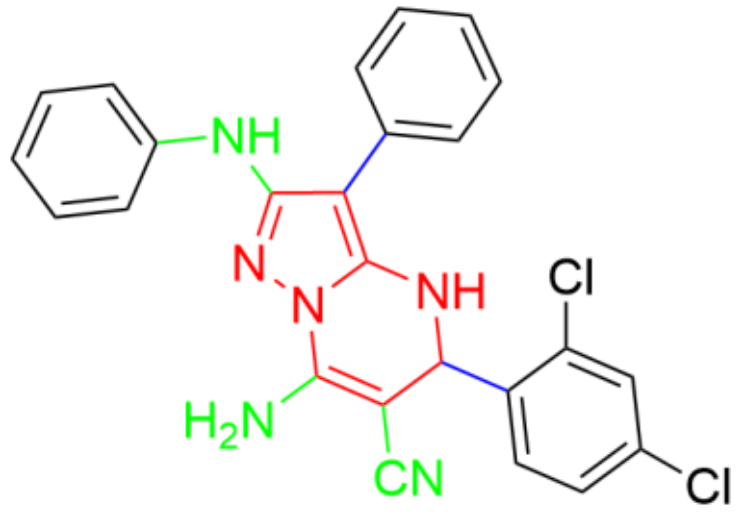

9e

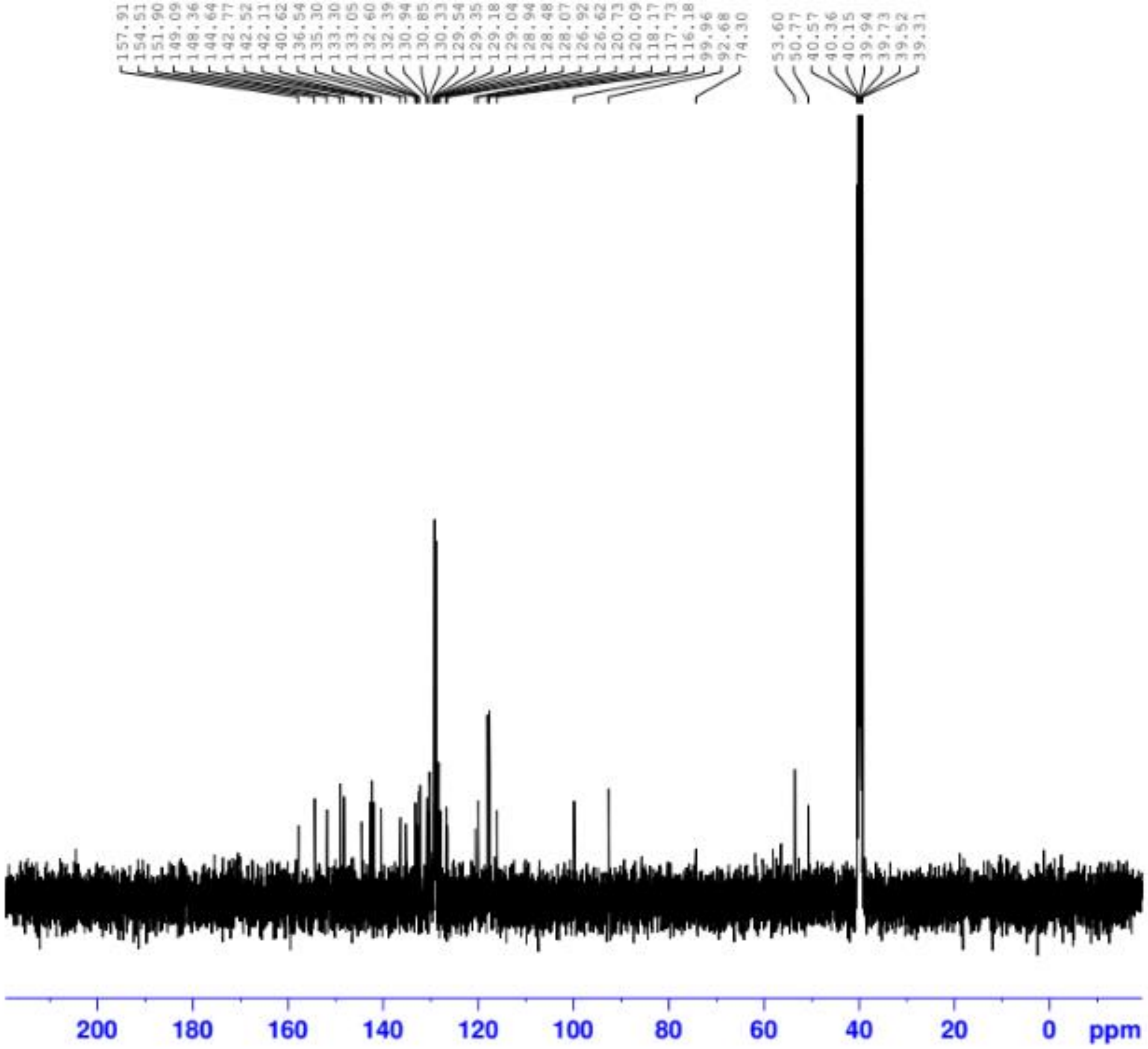

Current Data Parameters
NAME mohammed-attia-27g
EXPNO 2
PROCNO 1

F2 - Acquisition Parameters
Date_ 20210622
Time 10.54
INSTRUM spect
PROBHD 5 mm PABBO BB/
PULPROG zgpg30
TD 65536
SOLVENT DMSO
NS 1657
DS 4
SWH 24038.461 Hz
FIDRES 0.366798 Hz
AQ 1.3631488 sec
RG 205.37
DW 20.800 usec
DE 6.50 usec
TE 300.0 K
D1 2.00000000 sec
D11 0.03000000 sec
TD0 1

======== CHANNEL f1 ========
SFO1 100.6278588 MHz
NUC1 13C
P1 10.00 usec
PLW1 47.00000000 W

======== CHANNEL f2 ========
SFO2 400.1516006 MHz
NUC2 1H
CPDPRG[2 waltz16
PCPD2 90.00 usec
PLW2 18.00000000 W
PLW12 0.34722000 W
PLW13 0.28125000 W

F2 - Processing parameters
SI 32768
SF 100.6177975 MHz
WDW EM
SSB 0
LB 1.00 Hz
GB 0
PC 1.40

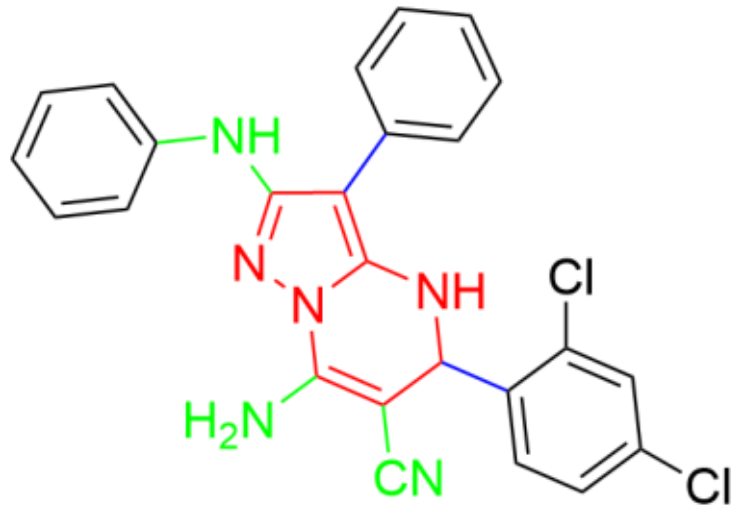

9e

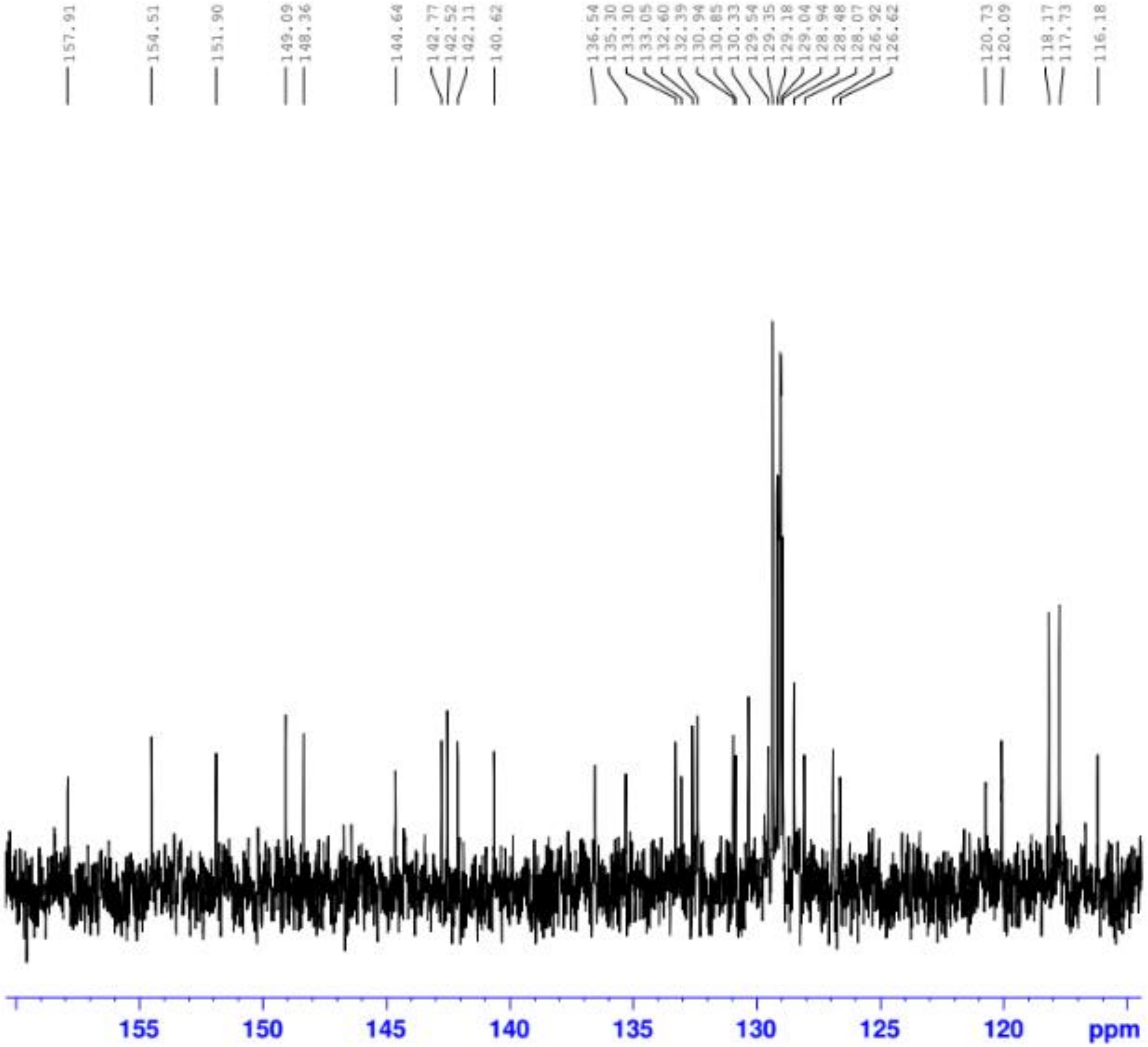

BRUKER

Current Data Parameters
NAME mohammed-attia-27g
EXPNO 2
PROCNO 1

F2 - Acquisition Parameters
Date_ 20210622
Time 10.54
INSTRUM spect
PROBHD 5 mm PABBO BB/
PULPROG zgpg30
TD 65536
SOLVENT DMSO
NS 1657
DS 4
SWH 24038.461 Hz
FIDRES 0.366798 Hz
AQ 1.3631488 sec
RG 205.37
DW 20.800 usec
DE 6.50 usec
TE 300.0 K
D1 2.00000000 sec
D11 0.03000000 sec
TD0 1

======== CHANNEL f1 ========
SFO1 100.6278588 MHz
NUC1 13C
P1 10.00 usec
PLW1 47.00000000 W

======== CHANNEL f2 ========
SFO2 400.1516006 MHz
NUC2 1H
CPDPRG[2 waltz16
PCPD2 90.00 usec
PLW2 18.00000000 W
PLW12 0.34722000 W
PLW13 0.28125000 W

F2 - Processing parameters
SI 32768
SF 100.6177975 MHz
WDW EM
SSB 0
LB 1.00 Hz
GB 0
PC 1.40

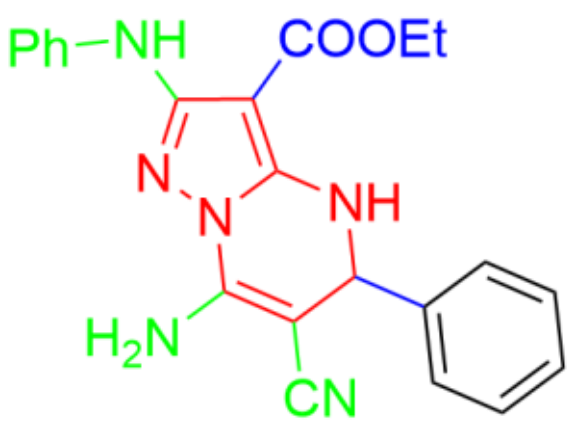

9f

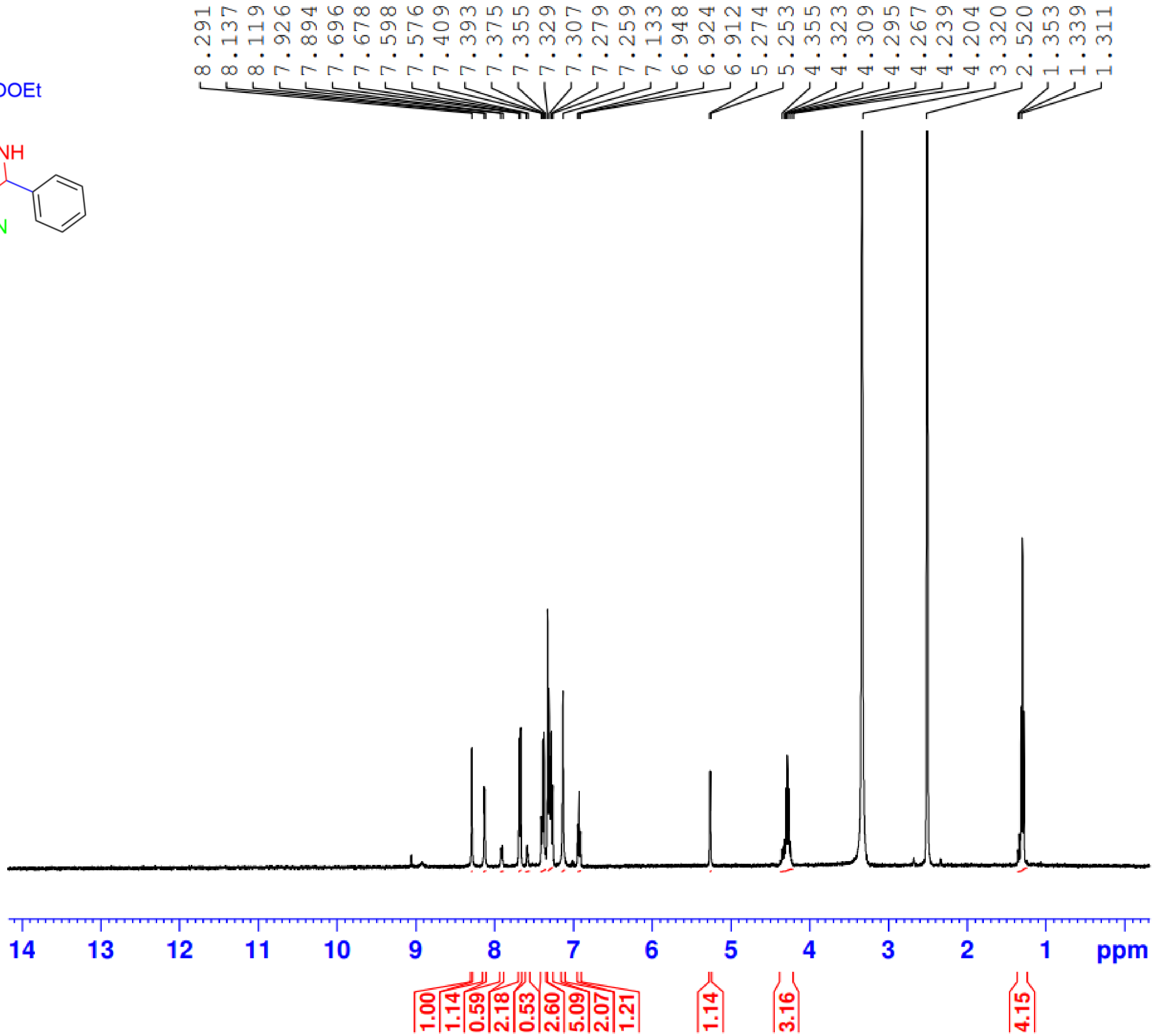

BRUKER

Current Data Parameters
NAME mohammed-attia-23a
EXPNO 1
PROCNO 1

F2 - Acquisition Parameters
Date_ 20210524
Time 14.23
INSTRUM spect
PROBHD 5 mm PABBO BB/
PULPROG zg30
TD 65536
SOLVENT DMSO
NS 39
DS 2
SWH 8012.820 Hz
FIDRES 0.122266 Hz
AQ 4.0894465 sec
RG 205.37
DW 62.400 usec
DE 6.50 usec
TE 300.0 K
D1 1.00000000 sec
TD0 1

======== CHANNEL f1 ========
SFO1 400.1524711 MHz
NUC1 1H
P1 12.00 usec
PLW1 18.00000000 W

F2 - Processing parameters
SI 65536
SF 400.1500000 MHz
WDW EM
SSB 0
LB 0.30 Hz
GB 0
PC 1.00

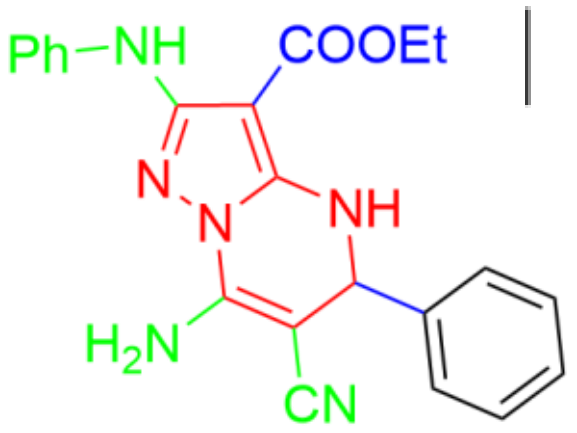

9f

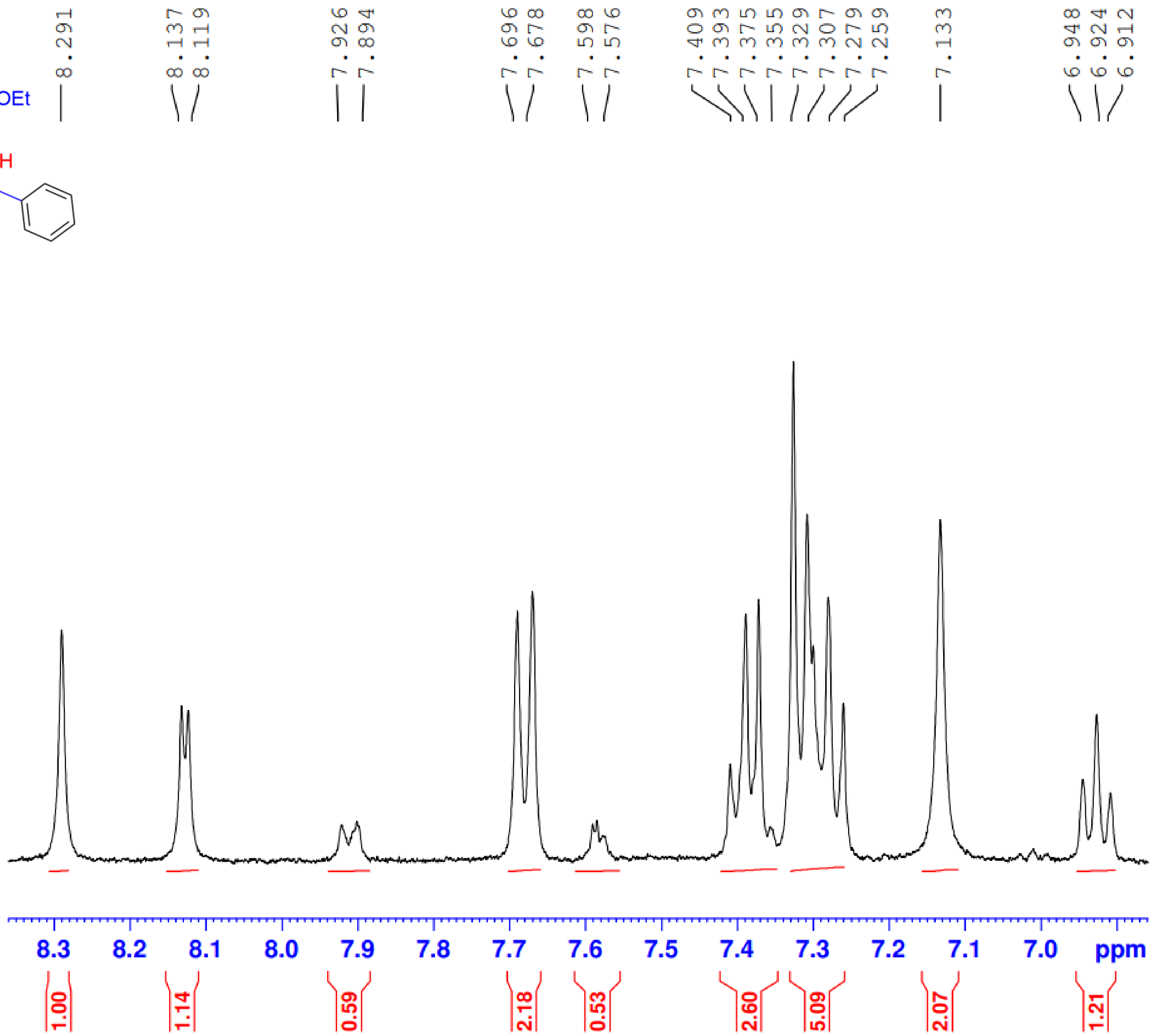


```
Current Data Parameters
NAME       mohammed-attia-23a
EXPNO                       1
PROCNO                      1

F2 - Acquisition Parameters
Date_              20210524
Time                  14.23
INSTRUM               spect
PROBHD    5 mm PABBO BB/
PULPROG                zg30
TD                    65536
SOLVENT                DMSO
NS                       39
DS                        2
SWH                8012.820 Hz
FIDRES             0.122266 Hz
AQ                4.0894465 sec
RG                   205.37
DW                   62.400 usec
DE                     6.50 usec
TE                    300.0 K
D1               1.00000000 sec
TD0                       1

======== CHANNEL f1 ========
SFO1            400.1524711 MHz
NUC1                     1H
P1                    12.00 usec
PLW1            18.00000000 W

F2 - Processing parameters
SI                    65536
SF              400.1500000 MHz
WDW                      EM
SSB      0
LB                     0.30 Hz
GB       0
PC                     1.00
```

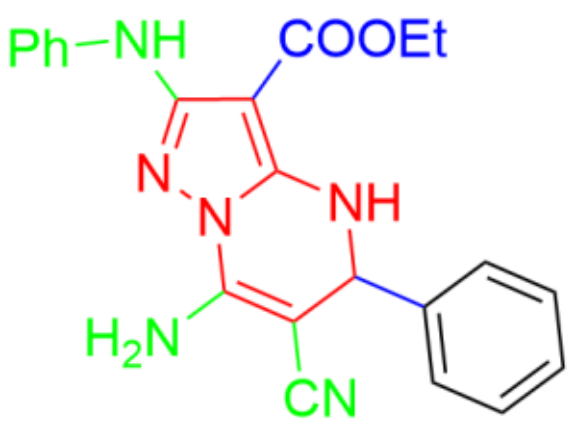

9f

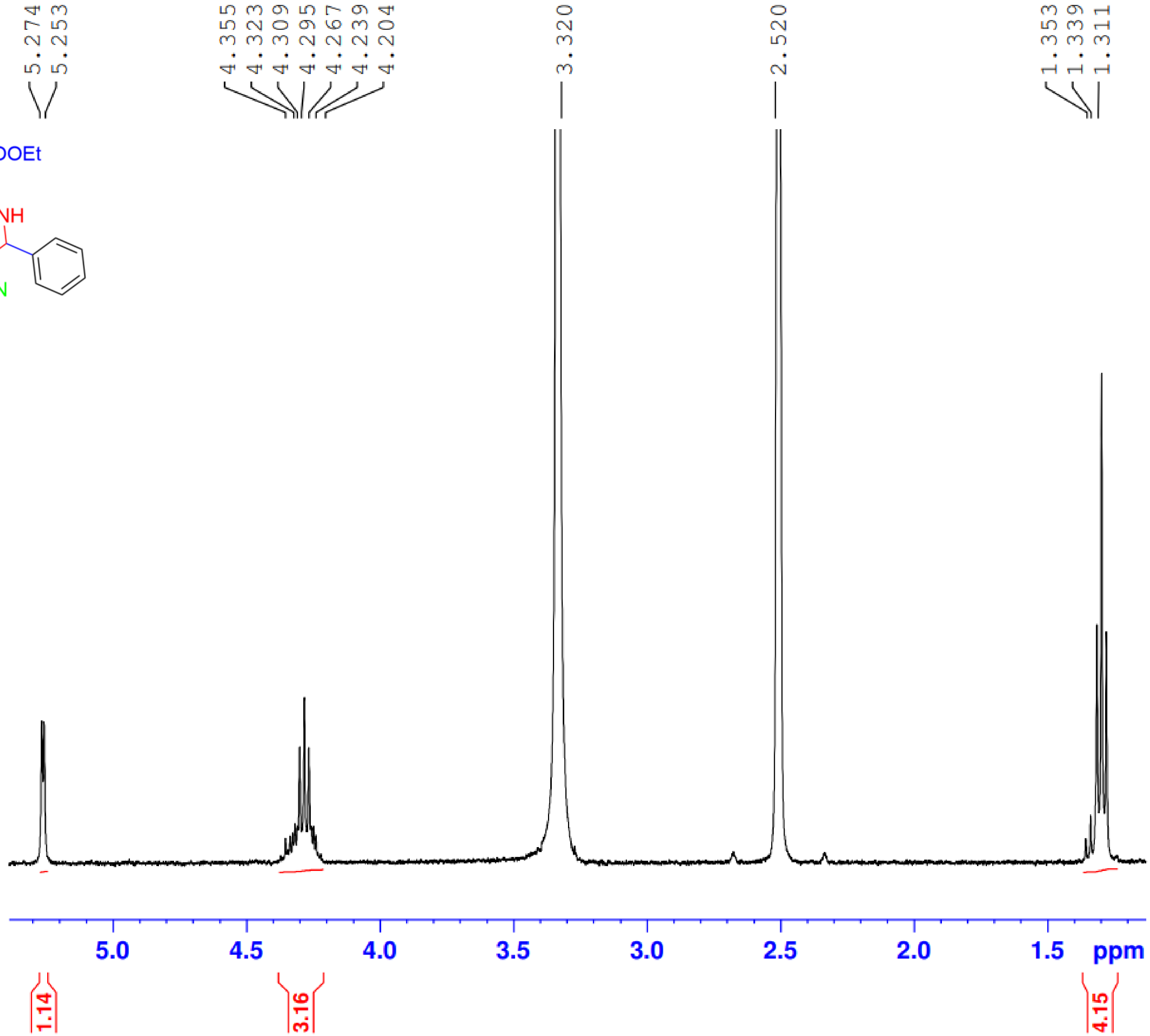

BRUKER

Current Data Parameters
NAME mohammed-attia-23a
EXPNO 1
PROCNO 1

F2 - Acquisition Parameters
Date_ 20210524
Time 14.23
INSTRUM spect
PROBHD 5 mm PABBO BB/
PULPROG zg30
TD 65536
SOLVENT DMSO
NS 39
DS 2
SWH 8012.820 Hz
FIDRES 0.122266 Hz
AQ 4.0894465 sec
RG 205.37
DW 62.400 usec
DE 6.50 usec
TE 300.0 K
D1 1.00000000 sec
TD0 1

======== CHANNEL f1 ========
SFO1 400.1524711 MHz
NUC1 1H
P1 12.00 usec
PLW1 18.00000000 W

F2 - Processing parameters
SI 65536
SF 400.1500000 MHz
WDW EM
SSB 0
LB 0.30 Hz
GB 0
PC 1.00

D_2O

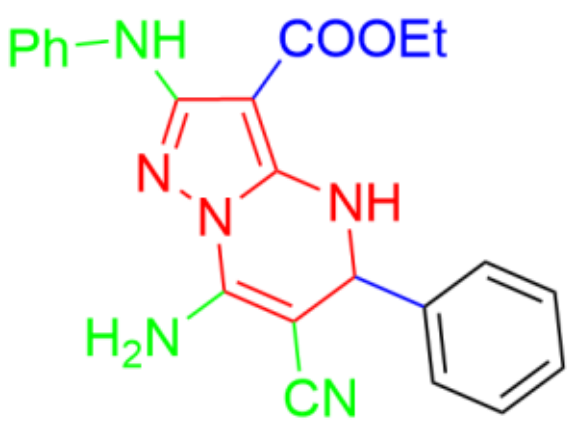

9f

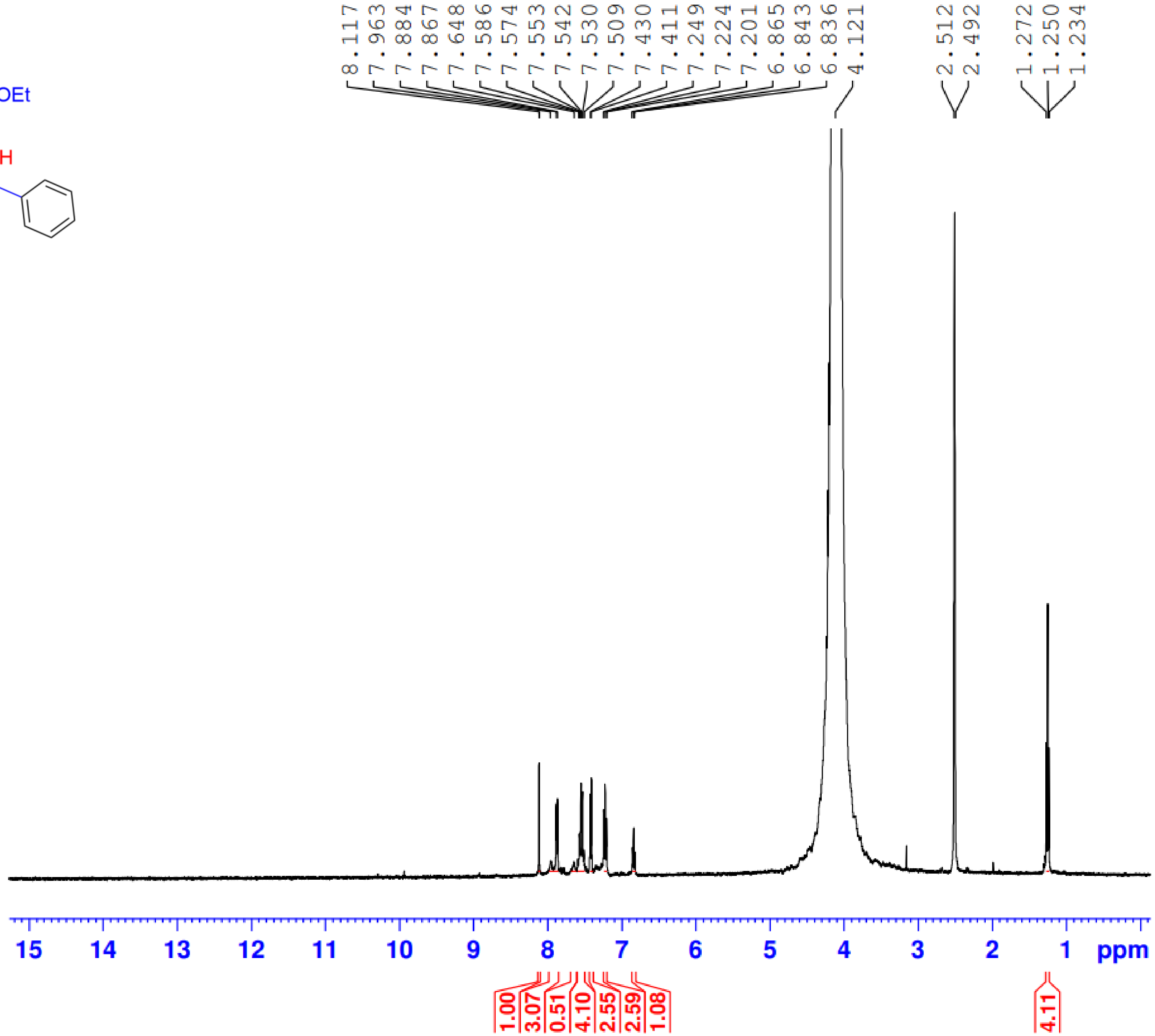


```
Current Data Parameters
NAME      mohammed-attia-23a-d2o
EXPNO                    1
PROCNO                   1

F2 - Acquisition Parameters
Date_             20210830
Time                 16.05
INSTRUM              spect
PROBHD    5 mm PABBO BB/
PULPROG               zg30
TD                   65536
SOLVENT               DMSO
NS                      32
DS                       2
SWH              8012.820 Hz
FIDRES           0.122266 Hz
AQ              4.0894465 sec
RG                   63.38
DW                  62.400 usec
DE                    6.50 usec
TE                   300.0 K
D1             1.00000000 sec
TD0                      1

======== CHANNEL f1 ========
SFO1          400.1524711 MHz
NUC1                    1H
P1                   12.00 usec
PLW1           18.00000000 W

F2 - Processing parameters
SI                   65536
SF            400.1500000 MHz
WDW                     EM
SSB      0
LB                    0.30 Hz
GB       0
PC                    1.00
```

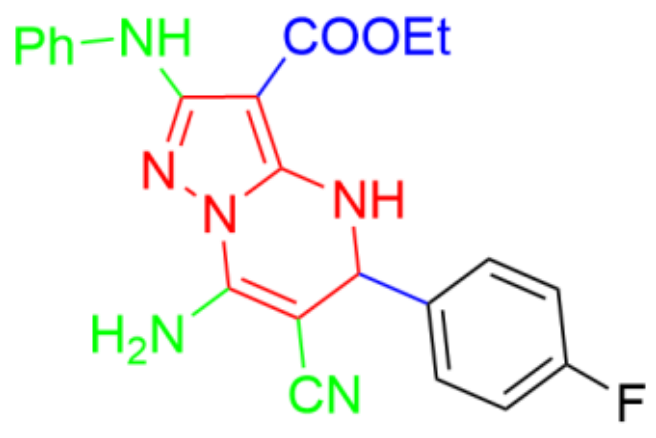

9g

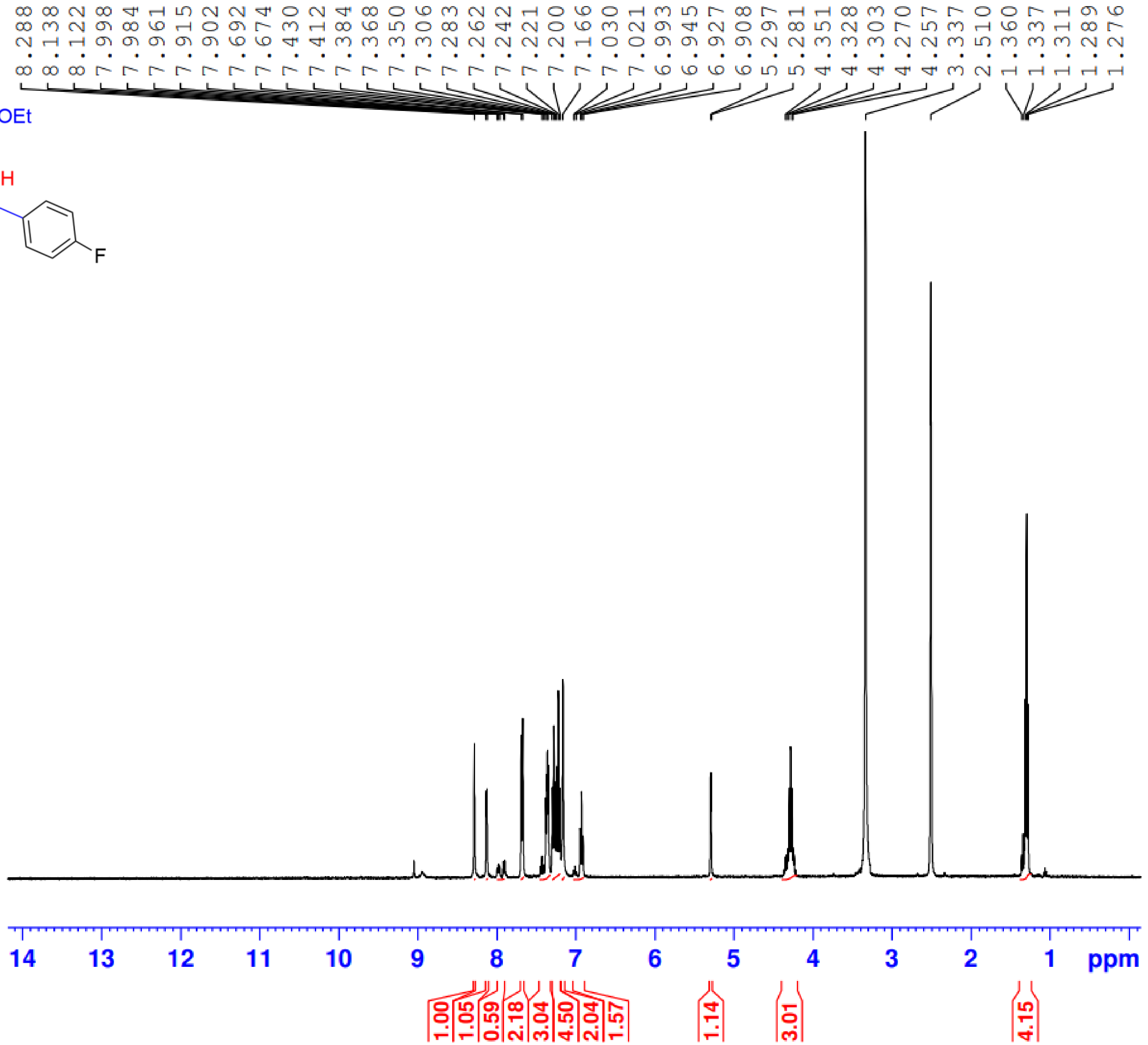

Current Data Parameters
NAME mohammed-attia-23e
EXPNO 1
PROCNO 1

F2 - Acquisition Parameters
Date_ 20210524
Time 14.17
INSTRUM spect
PROBHD 5 mm PABBO BB/
PULPROG zg30
TD 65536
SOLVENT DMSO
NS 36
DS 2
SWH 8012.820 Hz
FIDRES 0.122266 Hz
AQ 4.0894465 sec
RG 205.37
DW 62.400 usec
DE 6.50 usec
TE 300.0 K
D1 1.00000000 sec
TD0 1

======== CHANNEL f1 ========
SFO1 400.1524711 MHz
NUC1 1H
P1 12.00 usec
PLW1 18.00000000 W

F2 - Processing parameters
SI 65536
SF 400.1500000 MHz
WDW EM
SSB 0
LB 0.30 Hz
GB 0
PC 1.00

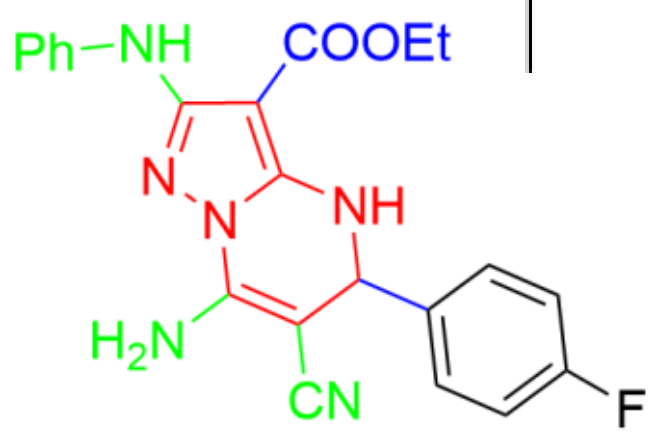

9g

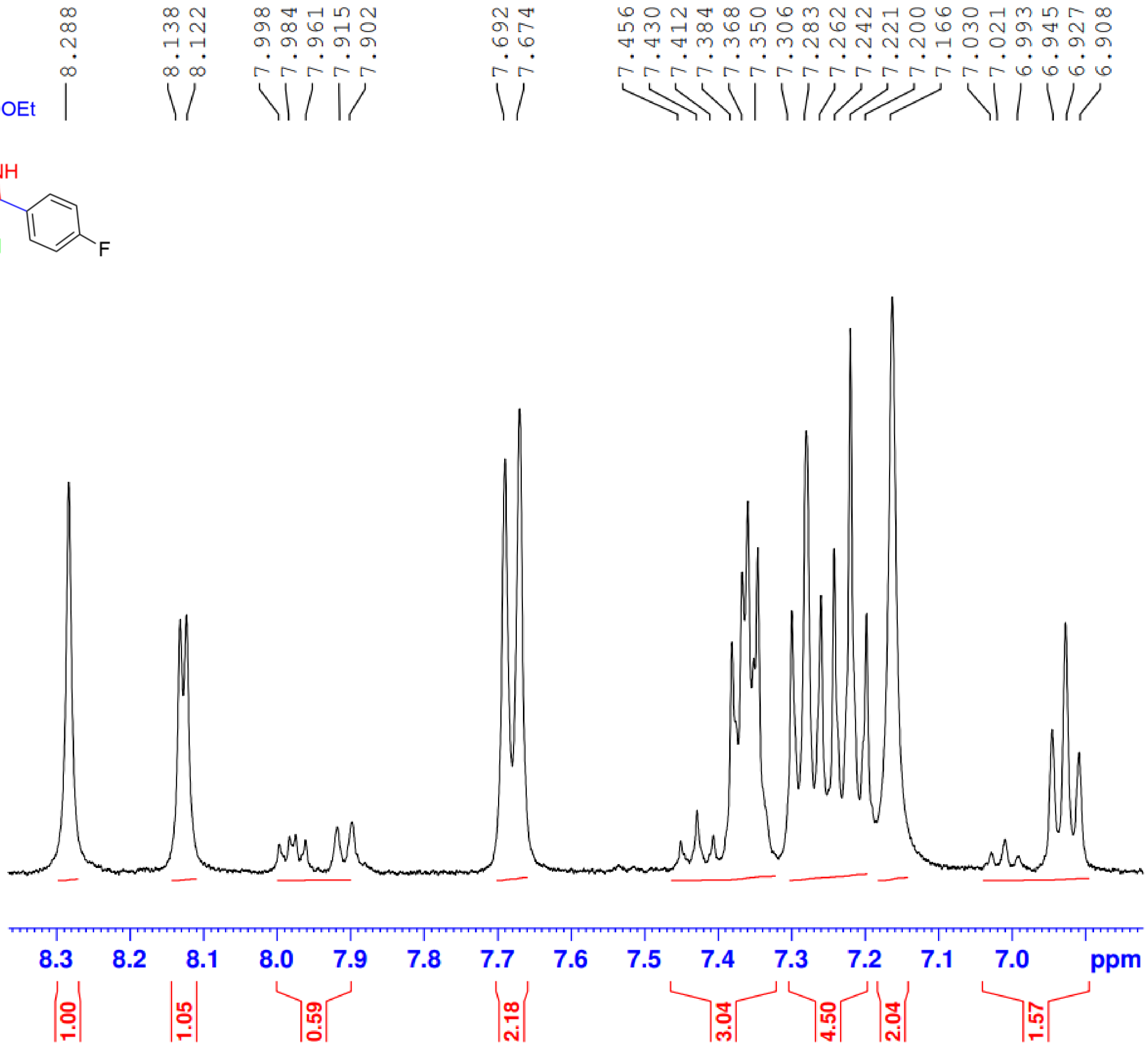

BRUKER

Current Data Parameters
NAME mohammed-attia-23e
EXPNO 1
PROCNO 1

F2 - Acquisition Parameters
Date_ 20210524
Time 14.17
INSTRUM spect
PROBHD 5 mm PABBO BB/
PULPROG zg30
TD 65536
SOLVENT DMSO
NS 36
DS 2
SWH 8012.820 Hz
FIDRES 0.122266 Hz
AQ 4.0894465 sec
RG 205.37
DW 62.400 usec
DE 6.50 usec
TE 300.0 K
D1 1.00000000 sec
TD0 1

======== CHANNEL f1 ========
SFO1 400.1524711 MHz
NUC1 1H
P1 12.00 usec
PLW1 18.00000000 W

F2 - Processing parameters
SI 65536
SF 400.1500000 MHz
WDW EM
SSB 0
LB 0.30 Hz
GB 0
PC 1.00

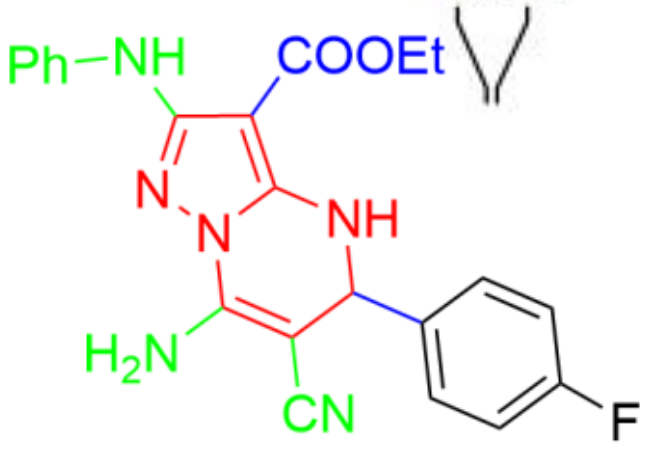

9g

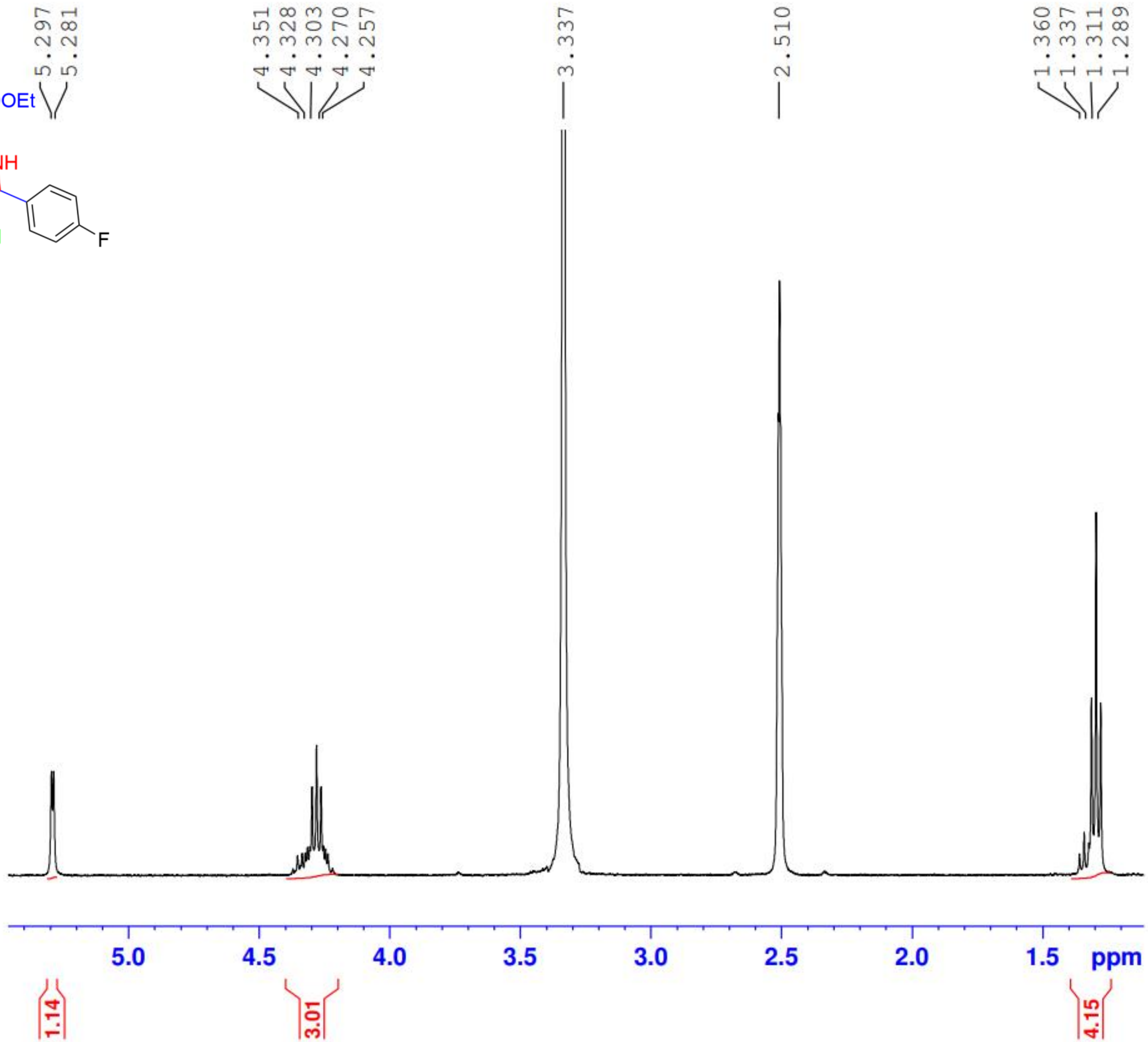


```
Current Data Parameters
NAME        mohammed-attia-23e
EXPNO                        1
PROCNO                       1

F2 - Acquisition Parameters
Date_               20210524
Time                   14.17
INSTRUM                spect
PROBHD     5 mm PABBO BB/
PULPROG                 zg30
TD                     65536
SOLVENT                 DMSO
NS                        36
DS                         2
SWH                 8012.820 Hz
FIDRES              0.122266 Hz
AQ                 4.0894465 sec
RG                    205.37
DW                    62.400 usec
DE                      6.50 usec
TE                     300.0 K
D1                1.00000000 sec
TD0                        1

======== CHANNEL f1 ========
SFO1             400.1524711 MHz
NUC1                      1H
P1                     12.00 usec
PLW1             18.00000000 W

F2 - Processing parameters
SI                     65536
SF               400.1500000 MHz
WDW                       EM
SSB       0
LB                      0.30 Hz
GB        0
PC                      1.00
```

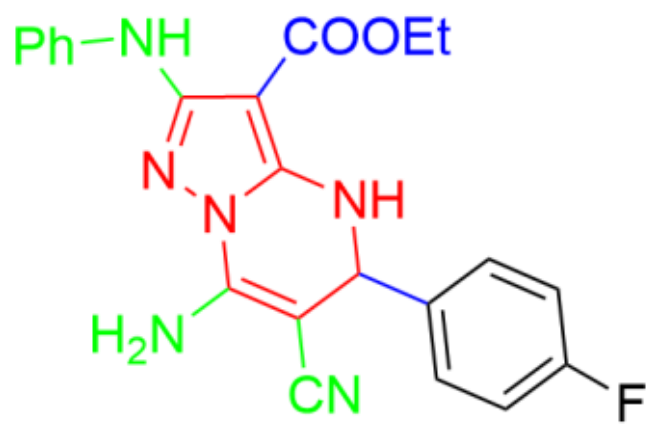

9g

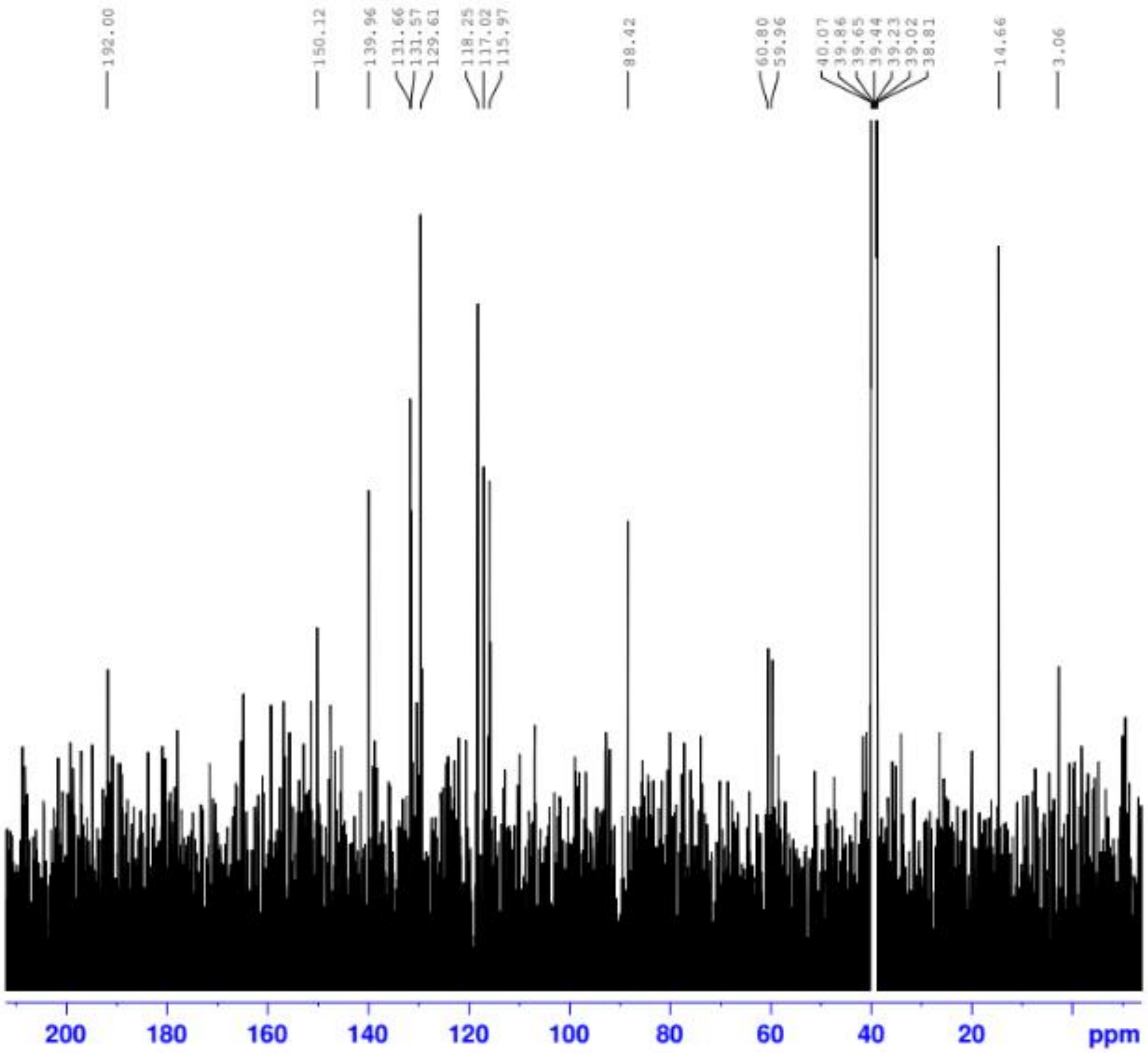

Current Data Parameters
NAME mohammed-attia-23e
EXPNO 2
PROCNO 1

F2 - Acquisition Parameters
Date_ 20210804
Time 17.32
INSTRUM spect
PROBHD 5 mm PABBO BB/
PULPROG zgpg30
TD 65536
SOLVENT DMSO
NS 5106
DS 4
SWH 24038.461 Hz
FIDRES 0.366798 Hz
AQ 1.3631488 sec
RG 205.37
DW 20.800 usec
DE 6.50 usec
TE 300.0 K
D1 2.00000000 sec
D11 0.03000000 sec
TD0 1

======== CHANNEL f1 ========
SFO1 100.6278588 MHz
NUC1 13C
P1 10.00 usec
PLW1 47.00000000 W

======== CHANNEL f2 ========
SFO2 400.1516006 MHz
NUC2 1H
CPDPRG[2 waltz16
PCPD2 90.00 usec
PLW2 18.00000000 W
PLW12 0.34722000 W
PLW13 0.28125000 W

F2 - Processing parameters
SI 32768
SF 100.6177975 MHz
WDW EM
SSB 0
LB 1.00 Hz
GB 0
PC 1.40

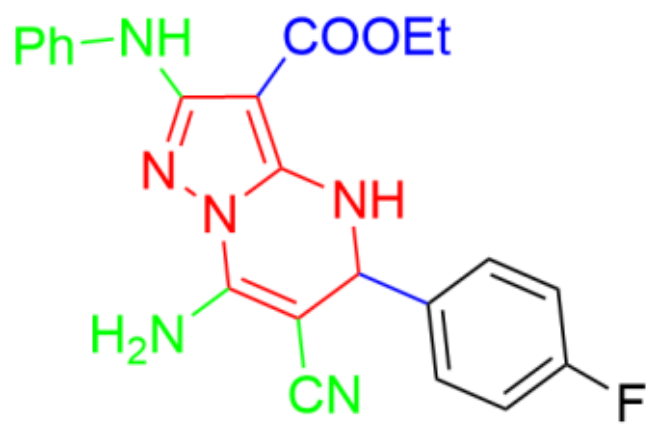

9g

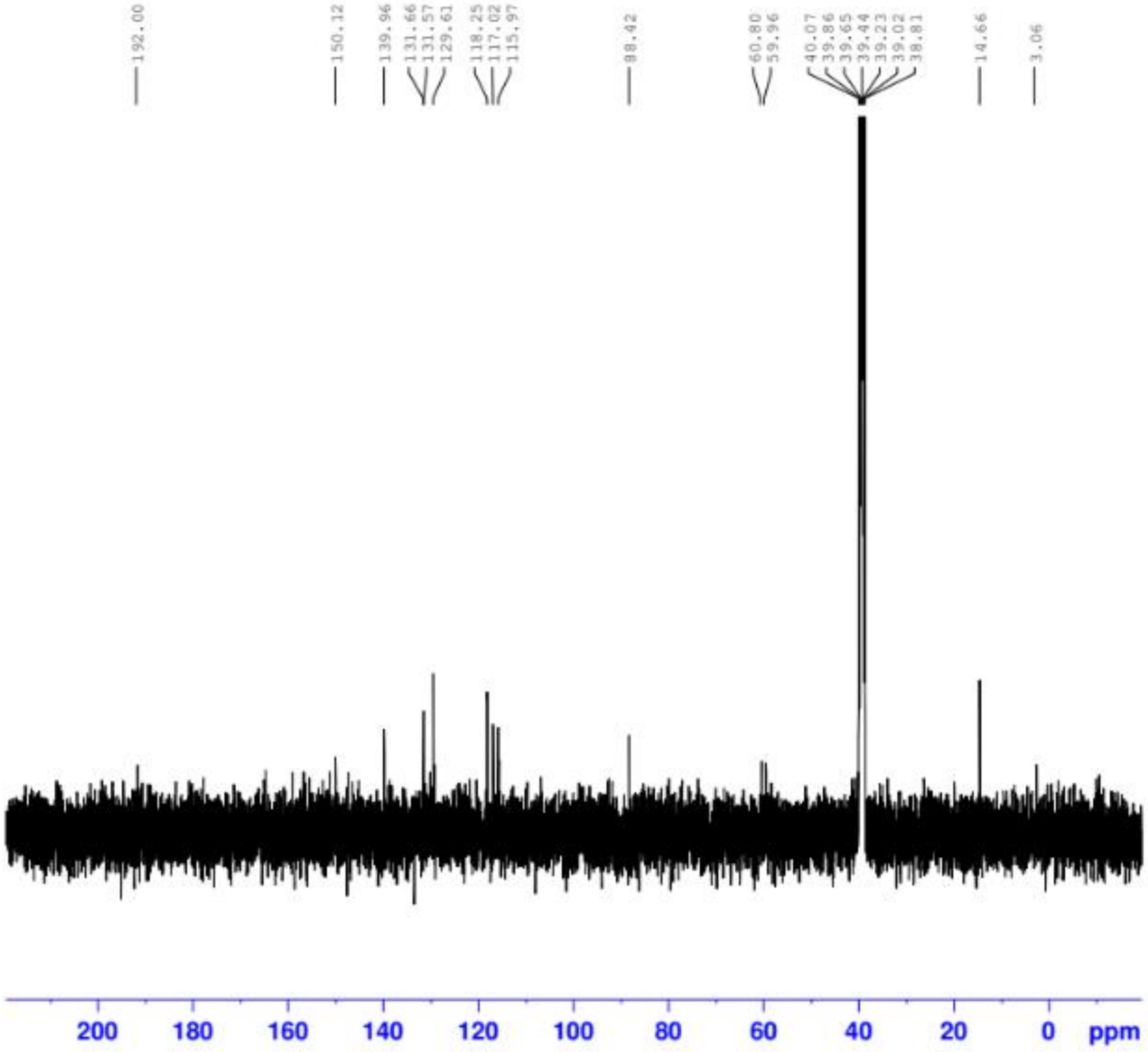

Current Data Parameters
NAME mohammed-attia-23e
EXPNO 2
PROCNO 1

F2 - Acquisition Parameters
Date_ 20210804
Time 17.32
INSTRUM spect
PROBHD 5 mm PABBO BB/
PULPROG zgpg30
TD 65536
SOLVENT DMSO
NS 5106
DS 4
SWH 24038.461 Hz
FIDRES 0.366798 Hz
AQ 1.3631488 sec
RG 205.37
DW 20.800 usec
DE 6.50 usec
TE 300.0 K
D1 2.00000000 sec
D11 0.03000000 sec
TD0 1

======== CHANNEL f1 ========
SFO1 100.6278588 MHz
NUC1 13C
P1 10.00 usec
PLW1 47.00000000 W

======== CHANNEL f2 ========
SFO2 400.1516006 MHz
NUC2 1H
CPDPRG[2 waltz16
PCPD2 90.00 usec
PLW2 18.00000000 W
PLW12 0.34722000 W
PLW13 0.28125000 W

F2 - Processing parameters
SI 32768
SF 100.6177975 MHz
WDW EM
SSB 0
LB 1.00 Hz
GB 0
PC 1.40

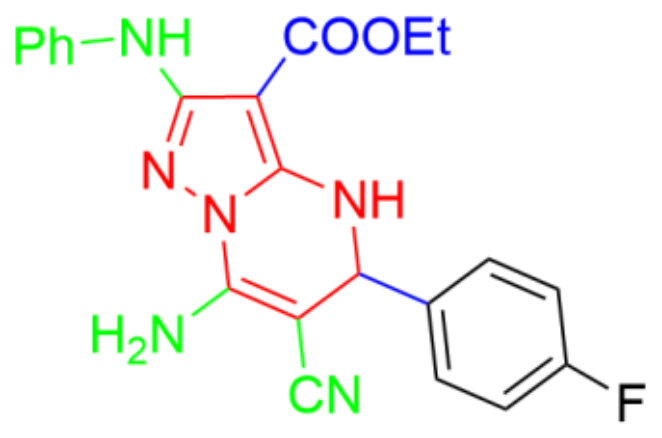

9g

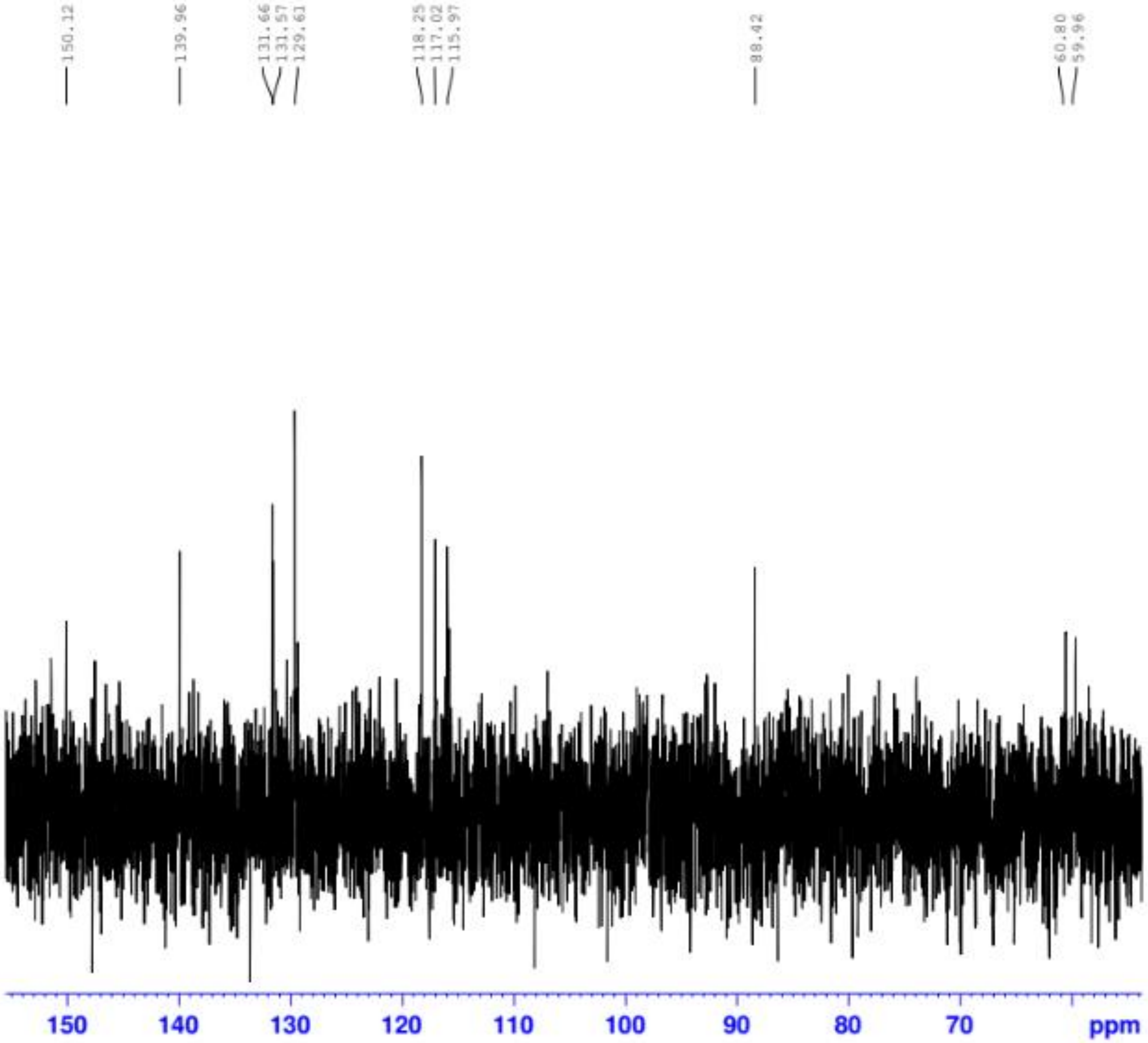


```
Current Data Parameters
NAME        mohammed-attia-23e
EXPNO                        2
PROCNO                       1

F2 - Acquisition Parameters
Date_                 20210804
Time                     17.32
INSTRUM                  spect
PROBHD     5 mm PABBO BB/
PULPROG                zgpg30
TD                       65536
SOLVENT                   DMSO
NS                        5106
DS                           4
SWH                  24038.461 Hz
FIDRES                0.366798 Hz
AQ                   1.3631488 sec
RG                      205.37
DW                      20.800 usec
DE                        6.50 usec
TE                       300.0 K
D1                  2.00000000 sec
D11                 0.03000000 sec
TD0                          1

======== CHANNEL f1 ========
SFO1               100.6278588 MHz
NUC1                       13C
P1                       10.00 usec
PLW1               47.00000000 W

======== CHANNEL f2 ========
SFO2               400.1516006 MHz
NUC2                        1H
CPDPRG[2               waltz16
PCPD2                    90.00 usec
PLW2               18.00000000 W
PLW12               0.34722000 W
PLW13               0.28125000 W

F2 - Processing parameters
SI                       32768
SF                 100.6177975 MHz
WDW                         EM
SSB      0
LB                        1.00 Hz
GB       0
PC                        1.40
```

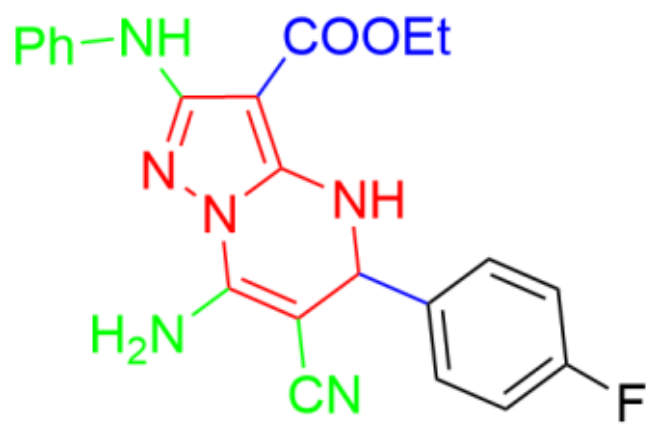
Ph–NH
COOEt
N
N
NH
H2N
CN
F

9g

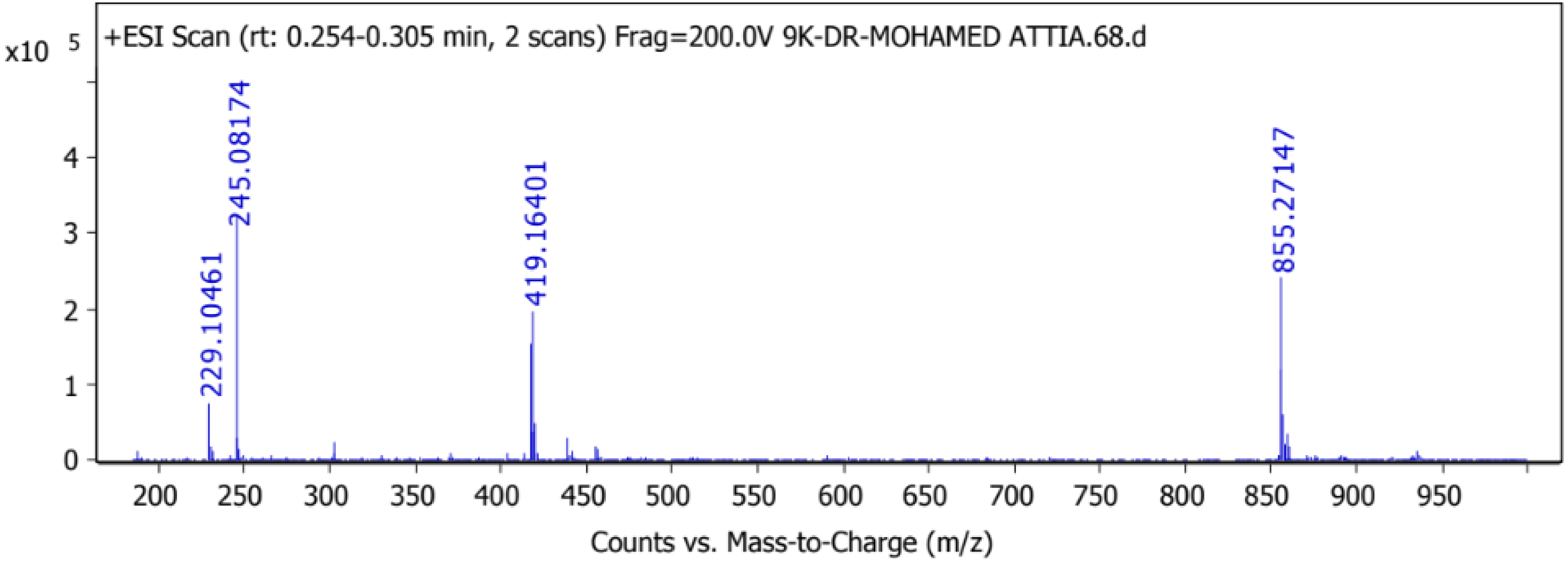
+ESI Scan (rt: 0.254-0.305 min, 2 scans) Frag=200.0V 9K-DR-MOHAMED ATTIA.68.d
x10 5
229.10461
245.08174
419.16401
855.27147
0
1
2
3
4
200
250
300
350
400
450
500
550
600
650
700
750
800
850
900
950
Counts vs. Mass-to-Charge (m/z)

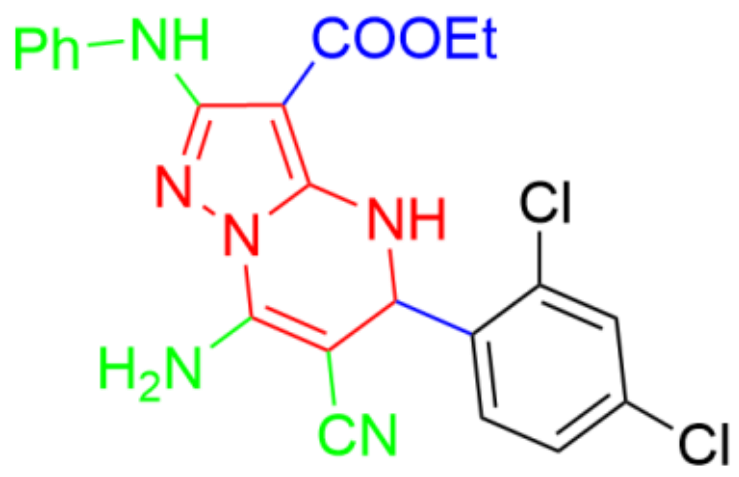

9h

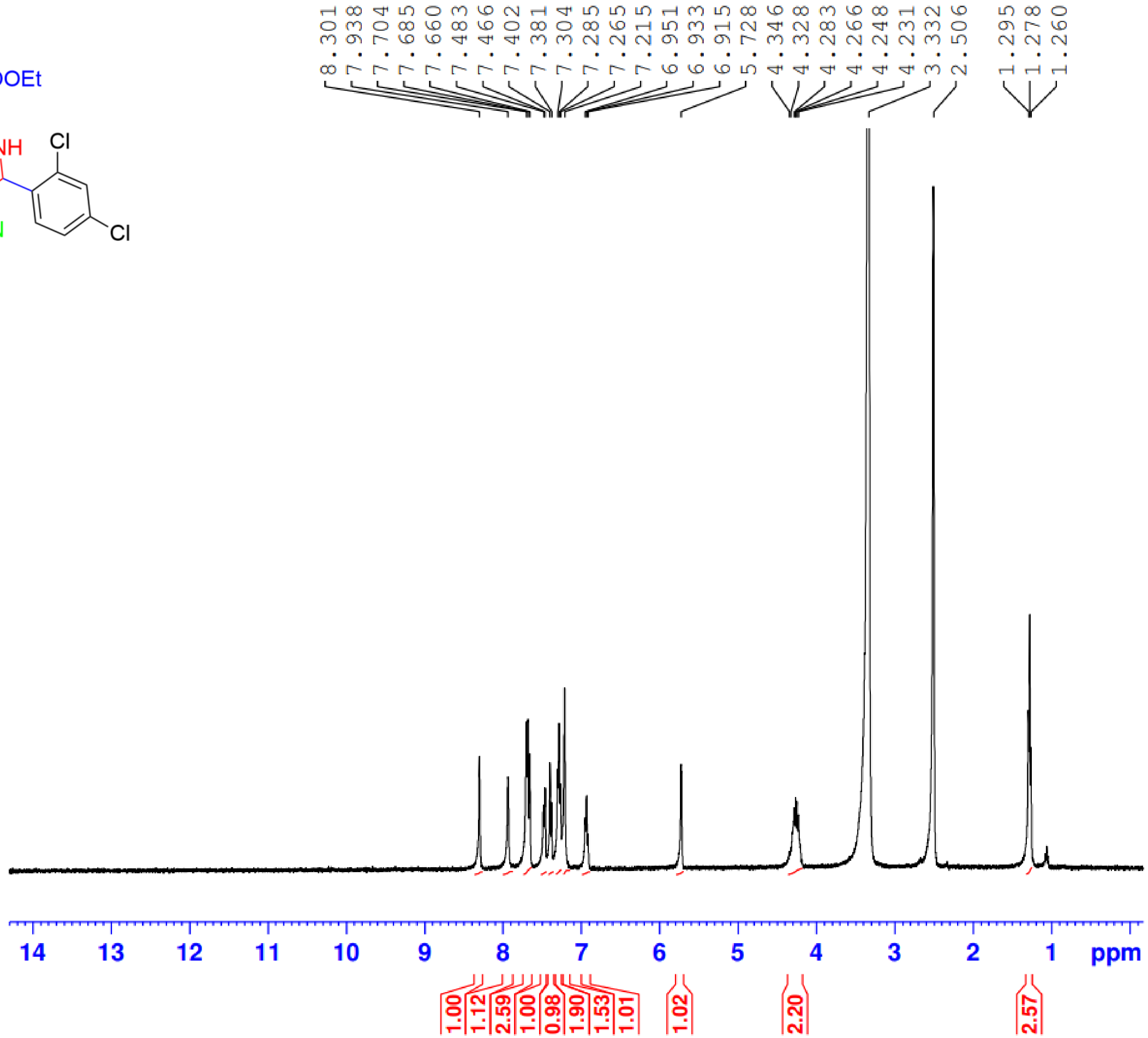

Current Data Parameters
NAME mohammed-attia-23g
EXPNO 1
PROCNO 1

F2 - Acquisition Parameters
Date_ 20210524
Time 14.40
INSTRUM spect
PROBHD 5 mm PABBO BB/
PULPROG zg30
TD 65536
SOLVENT DMSO
NS 32
DS 2
SWH 8012.820 Hz
FIDRES 0.122266 Hz
AQ 4.0894465 sec
RG 205.37
DW 62.400 usec
DE 6.50 usec
TE 300.0 K
D1 1.00000000 sec
TD0 1

======== CHANNEL f1 ========
SFO1 400.1524711 MHz
NUC1 1H
P1 12.00 usec
PLW1 18.00000000 W

F2 - Processing parameters
SI 65536
SF 400.1500000 MHz
WDW EM
SSB 0
LB 0.30 Hz
GB 0
PC 1.00

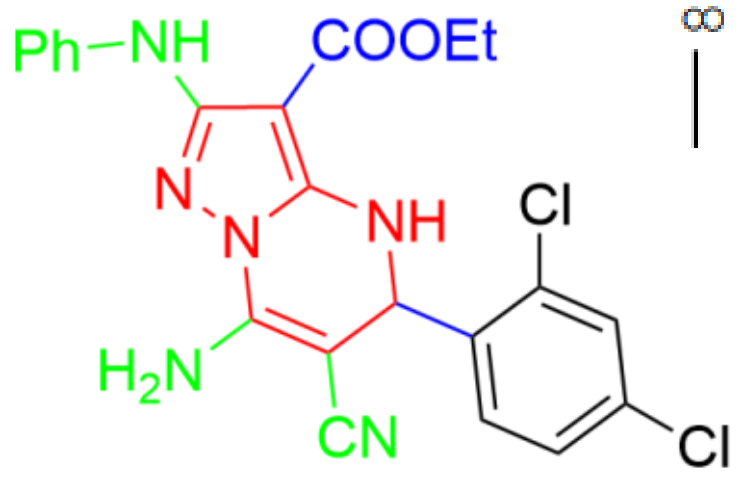

9h

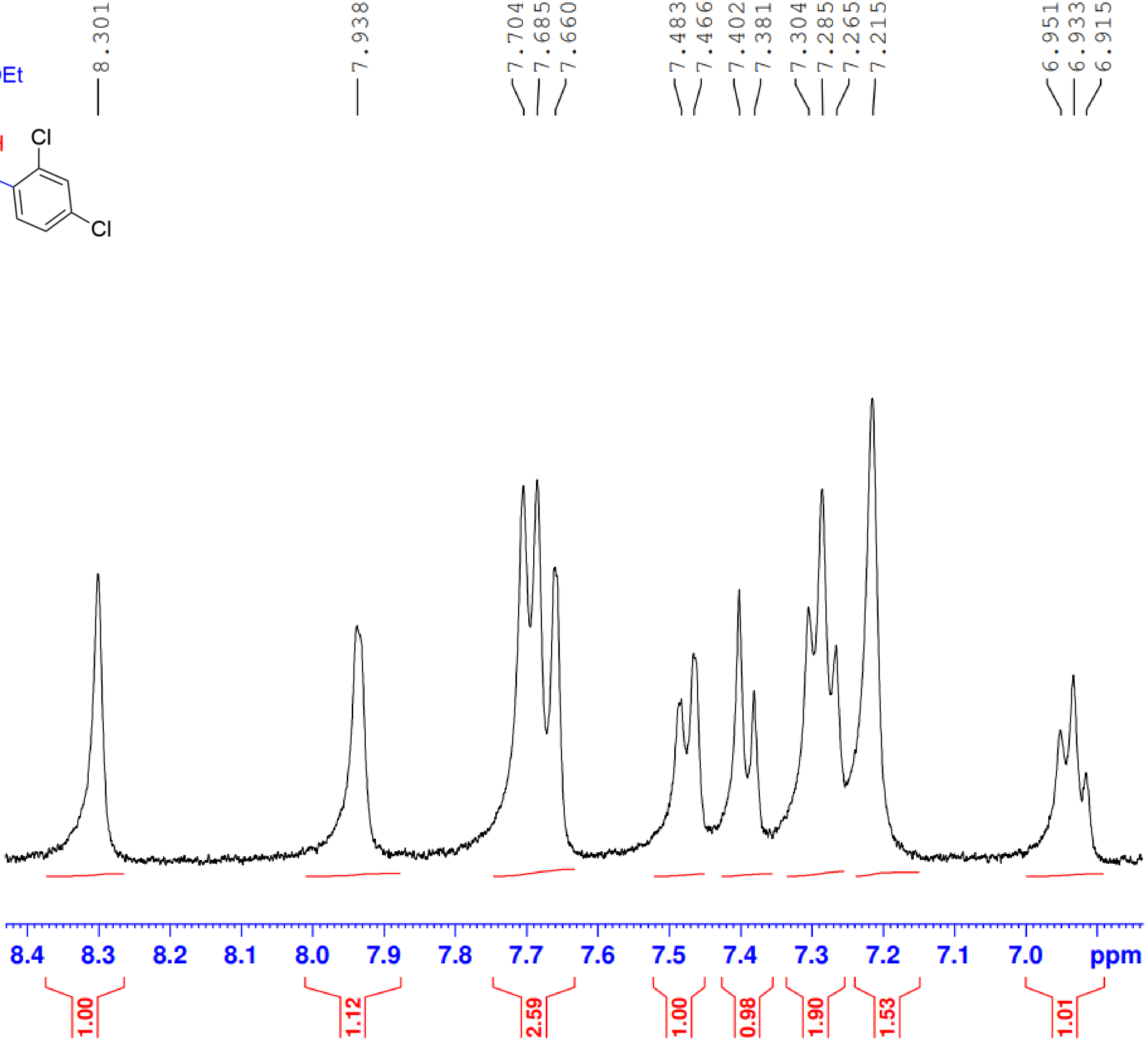

Current Data Parameters
NAME mohammed-attia-23g
EXPNO 1
PROCNO 1

F2 - Acquisition Parameters
Date_ 20210524
Time 14.40
INSTRUM spect
PROBHD 5 mm PABBO BB/
PULPROG zg30
TD 65536
SOLVENT DMSO
NS 32
DS 2
SWH 8012.820 Hz
FIDRES 0.122266 Hz
AQ 4.0894465 sec
RG 205.37
DW 62.400 usec
DE 6.50 usec
TE 300.0 K
D1 1.00000000 sec
TD0 1

======== CHANNEL f1 ========
SFO1 400.1524711 MHz
NUC1 1H
P1 12.00 usec
PLW1 18.00000000 W

F2 - Processing parameters
SI 65536
SF 400.1500000 MHz
WDW EM
SSB 0
LB 0.30 Hz
GB 0
PC 1.00

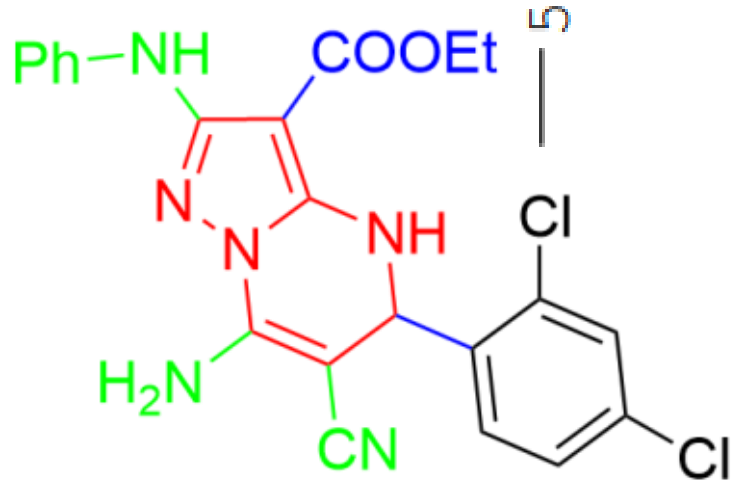

9h

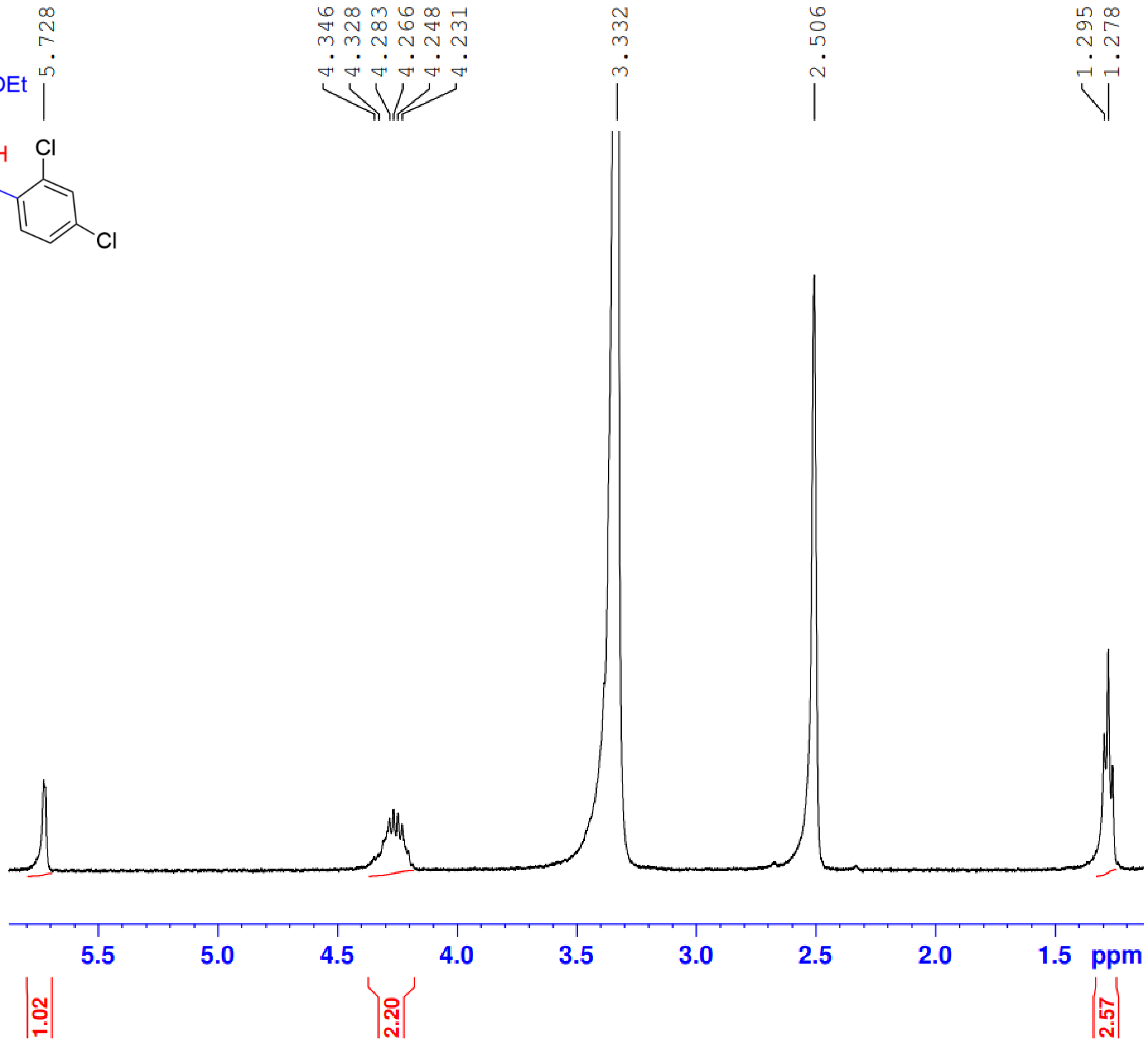

Current Data Parameters
NAME mohammed-attia-23g
EXPNO 1
PROCNO 1

F2 - Acquisition Parameters
Date_ 20210524
Time 14.40
INSTRUM spect
PROBHD 5 mm PABBO BB/
PULPROG zg30
TD 65536
SOLVENT DMSO
NS 32
DS 2
SWH 8012.820 Hz
FIDRES 0.122266 Hz
AQ 4.0894465 sec
RG 205.37
DW 62.400 usec
DE 6.50 usec
TE 300.0 K
D1 1.00000000 sec
TD0 1

======== CHANNEL f1 ========
SFO1 400.1524711 MHz
NUC1 1H
P1 12.00 usec
PLW1 18.00000000 W

F2 - Processing parameters
SI 65536
SF 400.1500000 MHz
WDW EM
SSB 0
LB 0.30 Hz
GB 0
PC 1.00

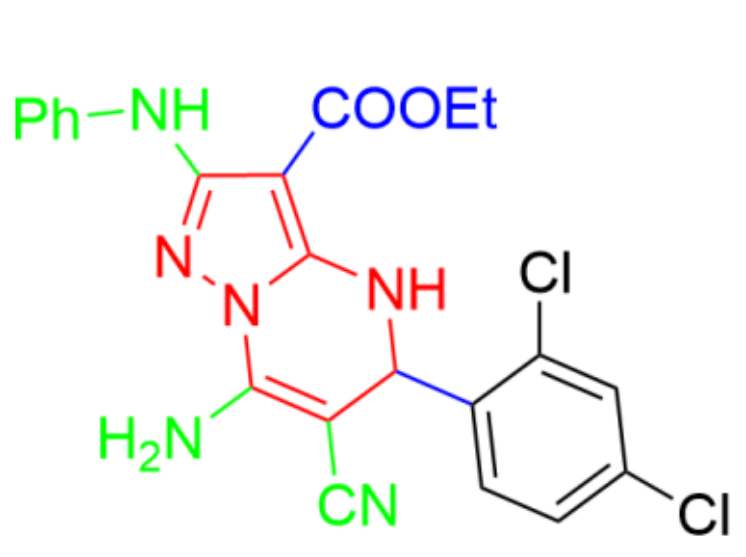

9h

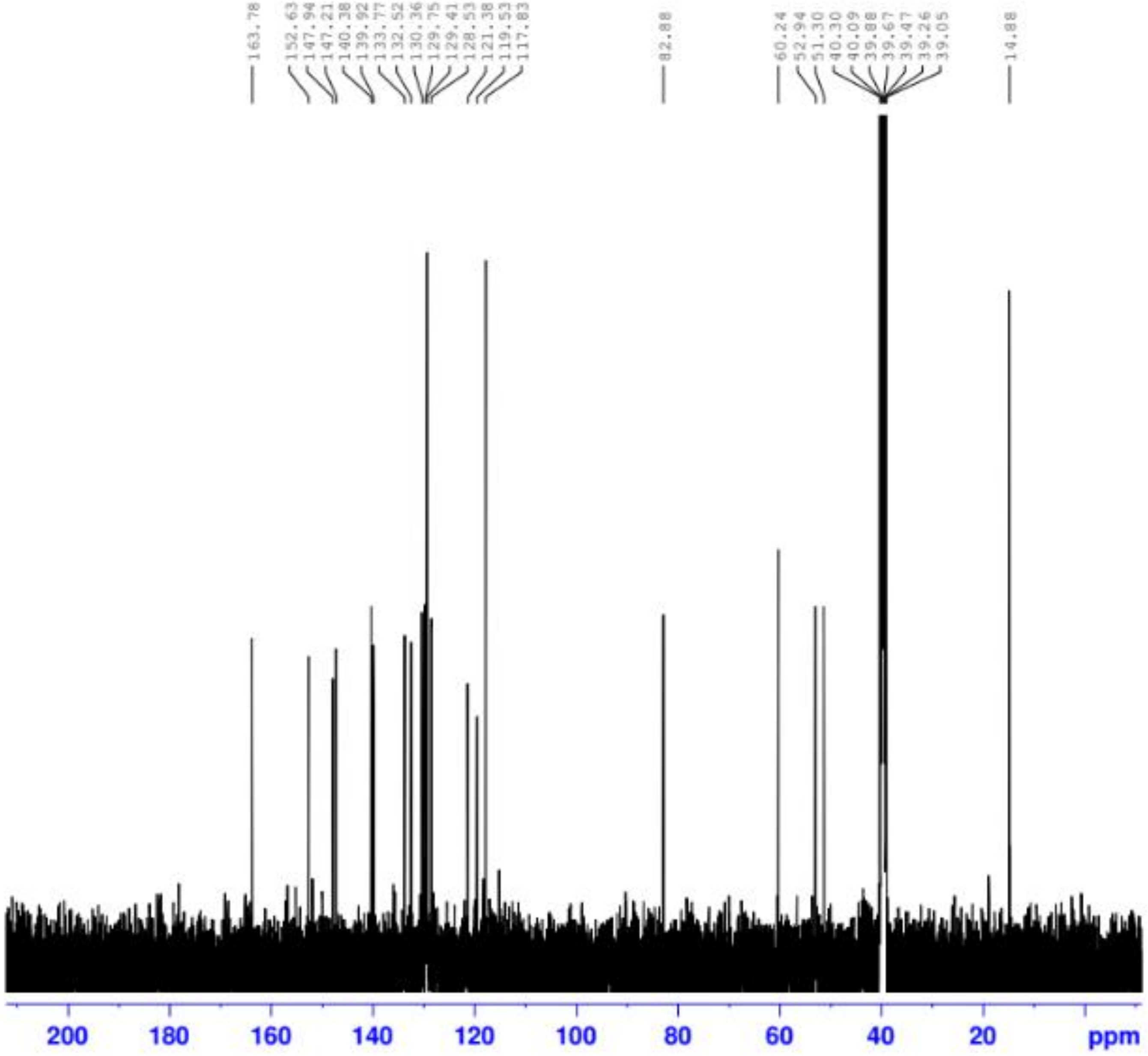

Current Data Parameters
NAME mohammed-attia-23g
EXPNO 2
PROCNO 1

F2 - Acquisition Parameters
Date_ 20210817
Time 9.17
INSTRUM spect
PROBHD 5 mm PABBO BB/
PULPROG zgpg30
TD 65536
SOLVENT DMSO
NS 937
DS 4
SWH 24038.461 Hz
FIDRES 0.366798 Hz
AQ 1.3631488 sec
RG 205.37
DW 20.800 usec
DE 6.50 usec
TE 300.0 K
D1 2.00000000 sec
D11 0.03000000 sec
TD0 1

======== CHANNEL f1 ========
SFO1 100.6278588 MHz
NUC1 13C
P1 10.00 usec
PLW1 47.00000000 W

======== CHANNEL f2 ========
SFO2 400.1516006 MHz
NUC2 1H
CPDPRG[2 waltz16
PCPD2 90.00 usec
PLW2 18.00000000 W
PLW12 0.34722000 W
PLW13 0.28125000 W

F2 - Processing parameters
SI 32768
SF 100.6177975 MHz
WDW EM
SSB 0
LB 1.00 Hz
GB 0
PC 1.40

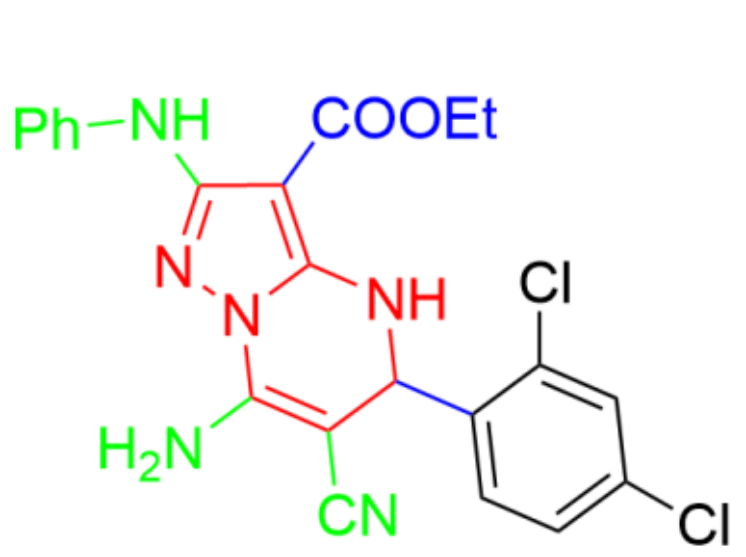

9h

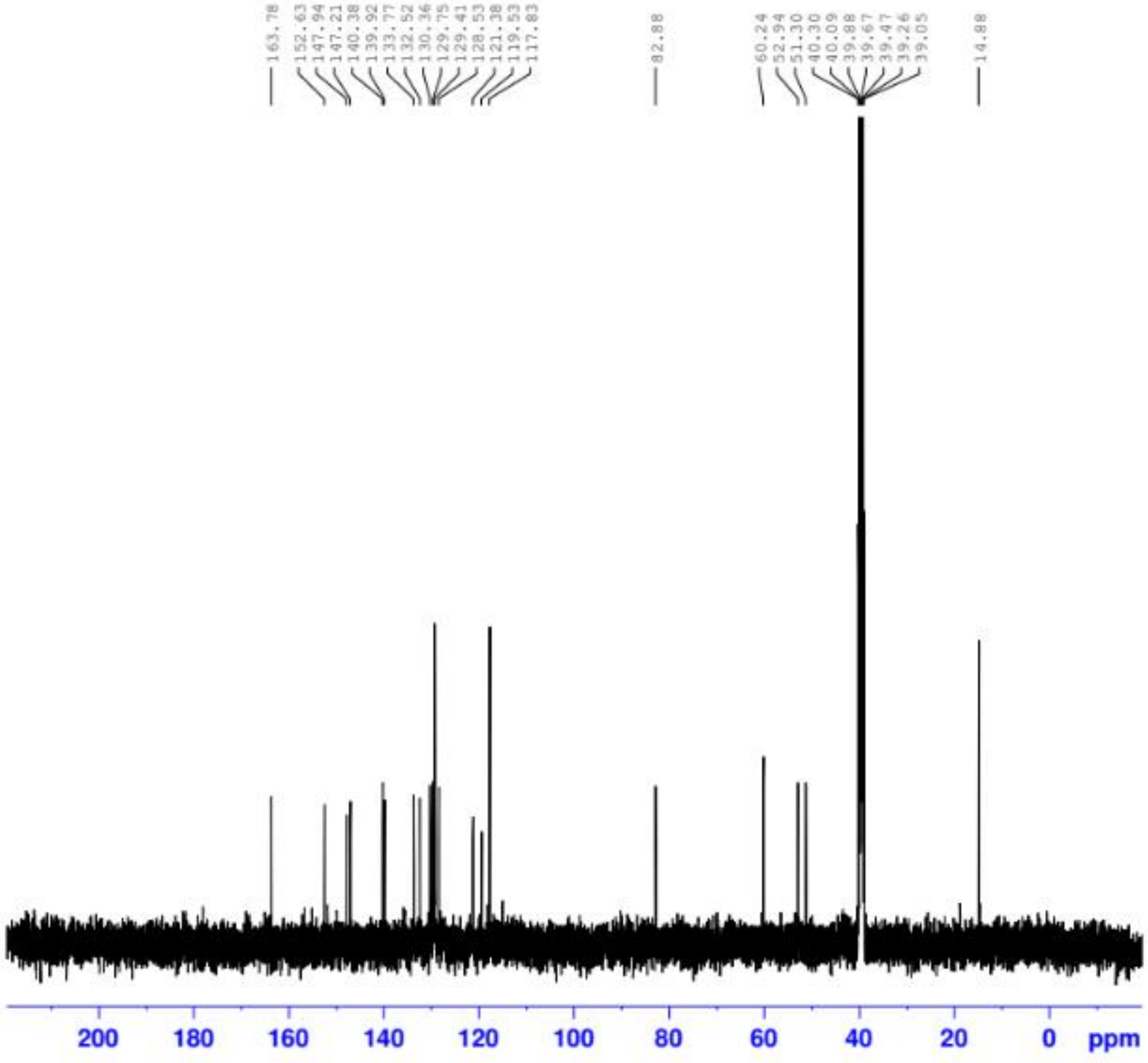


```
Current Data Parameters
NAME        mohammed-attia-23g
EXPNO                2
PROCNO               1

F2 - Acquisition Parameters
Date_            20210817
Time                 9.17
INSTRUM             spect
PROBHD     5 mm PABBO BB/
PULPROG           zgpg30
TD                 65536
SOLVENT             DMSO
NS                   937
DS                     4
SWH            24038.461 Hz
FIDRES          0.366798 Hz
AQ             1.3631488 sec
RG                205.37
DW                20.800 usec
DE                  6.50 usec
TE                 300.0 K
D1            2.00000000 sec
D11           0.03000000 sec
TD0                    1

======== CHANNEL f1 ========
SFO1         100.6278588 MHz
NUC1                 13C
P1                 10.00 usec
PLW1         47.00000000 W

======== CHANNEL f2 ========
SFO2         400.1516006 MHz
NUC2                  1H
CPDPRG[2          waltz16
PCPD2              90.00 usec
PLW2         18.00000000 W
PLW12         0.34722000 W
PLW13         0.28125000 W

F2 - Processing parameters
SI                 32768
SF           100.6177975 MHz
WDW                   EM
SSB       0
LB                  1.00 Hz
GB        0
PC                  1.40
```

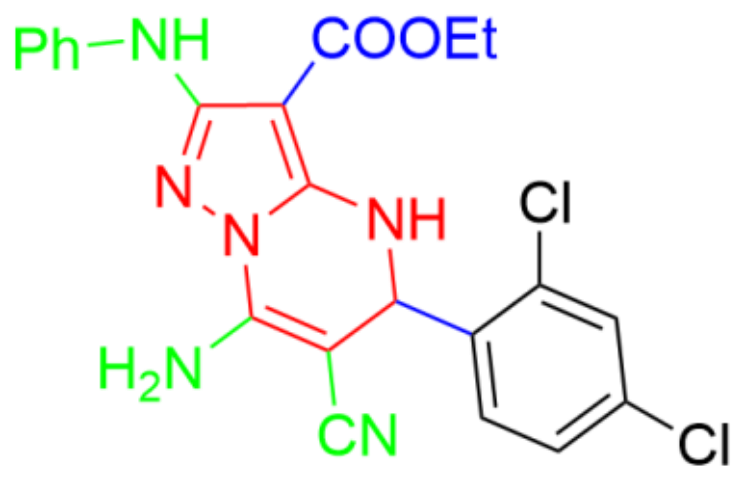

9h

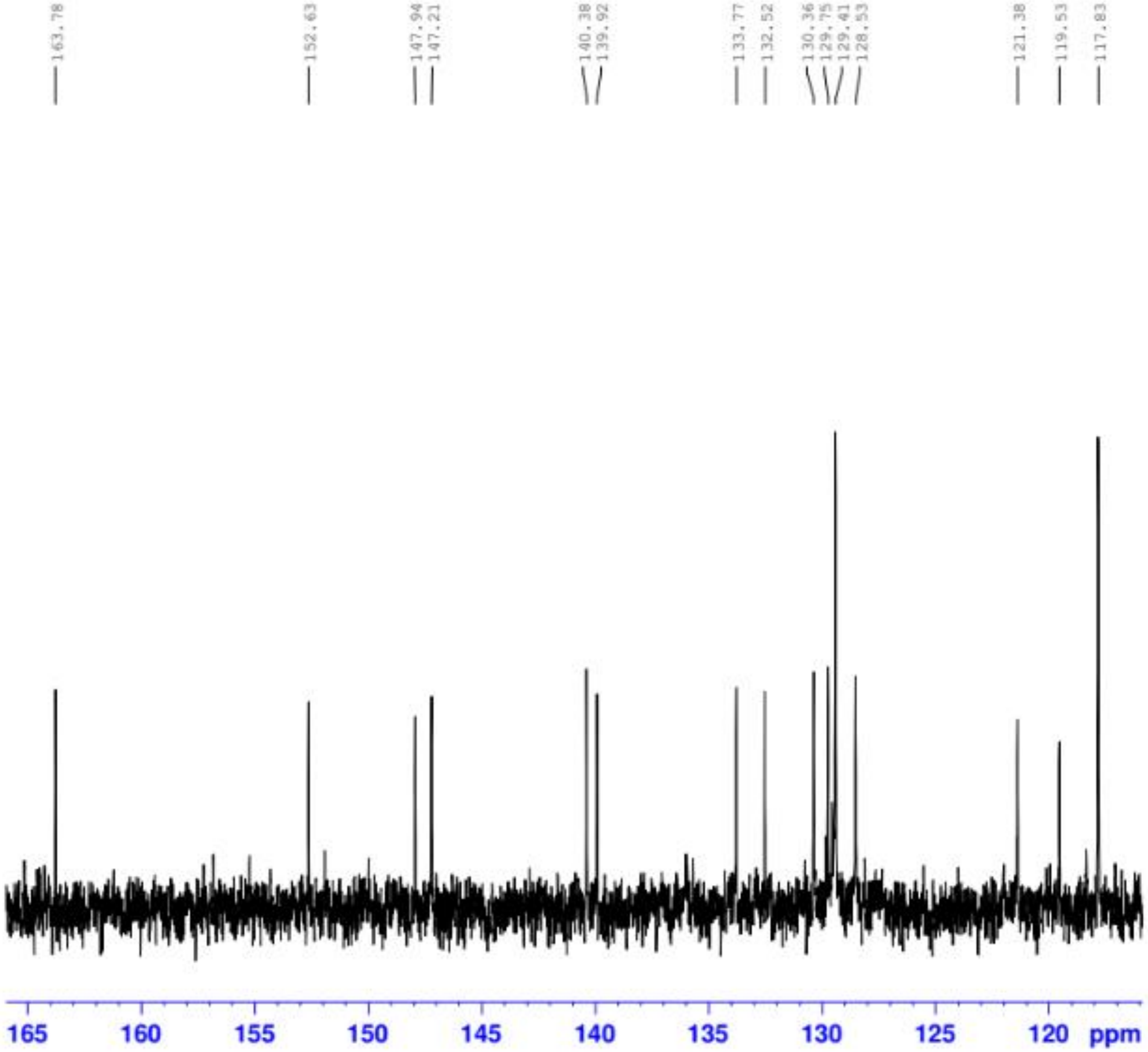

Current Data Parameters
NAME mohammed-attia-23g
EXPNO 2
PROCNO 1

F2 - Acquisition Parameters
Date_ 20210817
Time 9.17
INSTRUM spect
PROBHD 5 mm PABBO BB/
PULPROG zgpg30
TD 65536
SOLVENT DMSO
NS 937
DS 4
SWH 24038.461 Hz
FIDRES 0.366798 Hz
AQ 1.3631488 sec
RG 205.37
DW 20.800 usec
DE 6.50 usec
TE 300.0 K
D1 2.00000000 sec
D11 0.03000000 sec
TD0 1

======== CHANNEL f1 ========
SFO1 100.6278588 MHz
NUC1 13C
P1 10.00 usec
PLW1 47.00000000 W

======== CHANNEL f2 ========
SFO2 400.1516006 MHz
NUC2 1H
CPDPRG[2 waltz16
PCPD2 90.00 usec
PLW2 18.00000000 W
PLW12 0.34722000 W
PLW13 0.28125000 W

F2 - Processing parameters
SI 32768
SF 100.6177975 MHz
WDW EM
SSB 0
LB 1.00 Hz
GB 0
PC 1.40

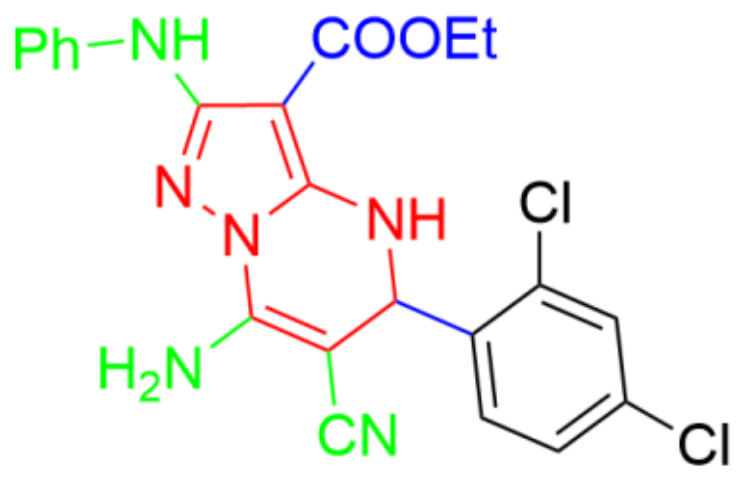
Ph—NH
COOEt
N
N
NH
Cl
H_2N
CN
Cl

9h

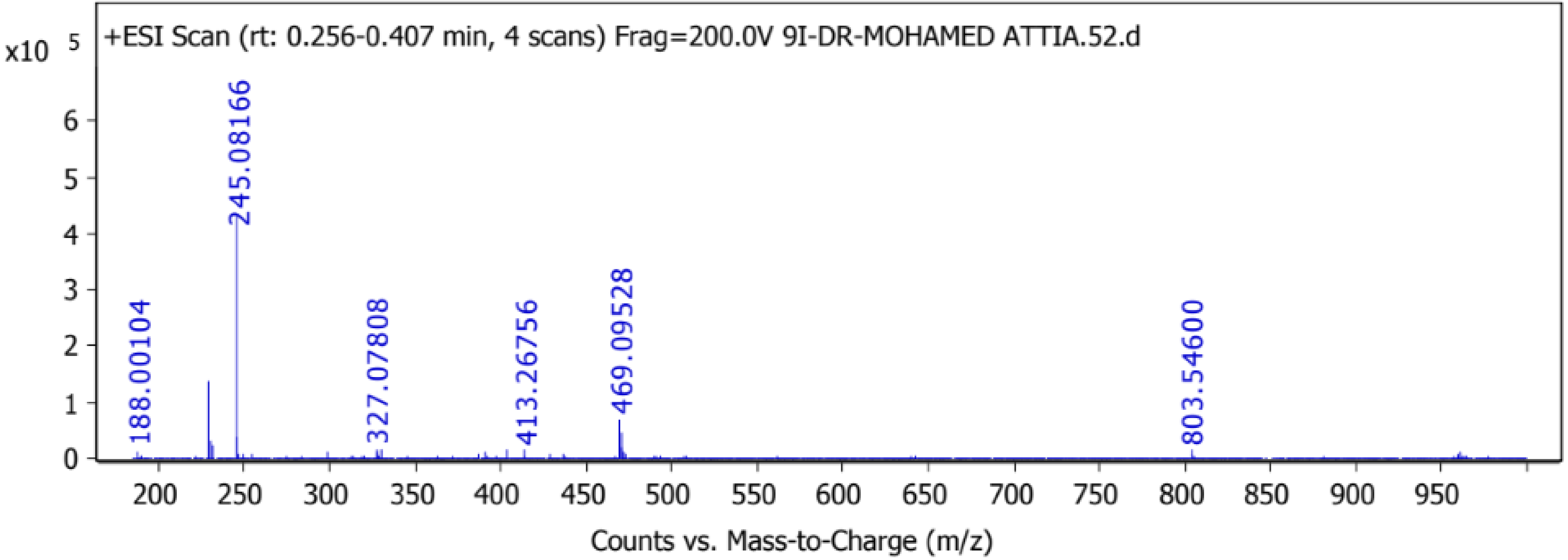
+ESI Scan (rt: 0.256-0.407 min, 4 scans) Frag=200.0V 9I-DR-MOHAMED ATTIA.52.d
x10 5
0
1
2
3
4
5
6
188.00104
245.08166
327.07808
413.26756
469.09528
803.54600
200
250
300
350
400
450
500
550
600
650
700
750
800
850
900
950
Counts vs. Mass-to-Charge (m/z)

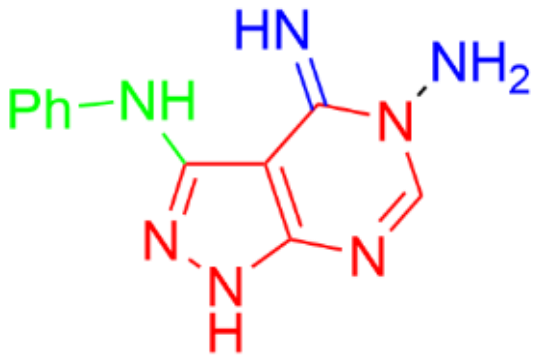

11

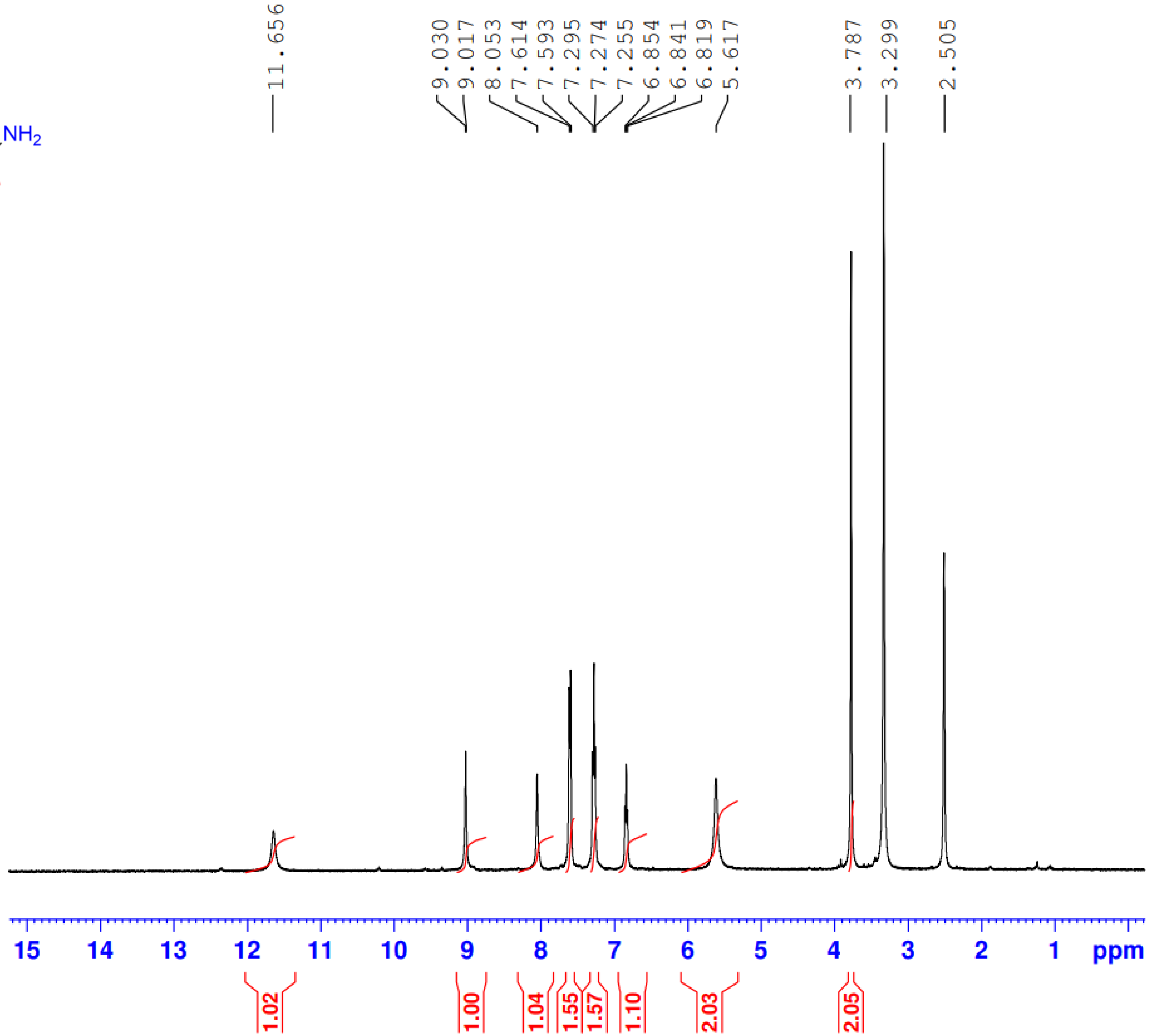

BRUKER

Current Data Parameters
NAME Mohammed-attia-33
EXPNO 1
PROCNO 1

F2 - Acquisition Parameters
Date_ 20210517
Time 14.02
INSTRUM spect
PROBHD 5 mm PABBO BB/
PULPROG zg30
TD 65536
SOLVENT DMSO
NS 33
DS 2
SWH 8012.820 Hz
FIDRES 0.122266 Hz
AQ 4.0894465 sec
RG 205.37
DW 62.400 usec
DE 6.50 usec
TE 300.0 K
D1 1.00000000 sec
TD0 1

======== CHANNEL f1 ========
SFO1 400.1524711 MHz
NUC1 1H
P1 12.00 usec
PLW1 18.00000000 W

F2 - Processing parameters
SI 65536
SF 400.1500000 MHz
WDW EM
SSB 0
LB 0.30 Hz
GB 0
PC 1.00

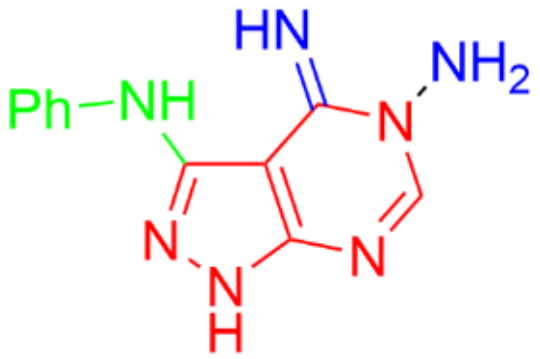

11

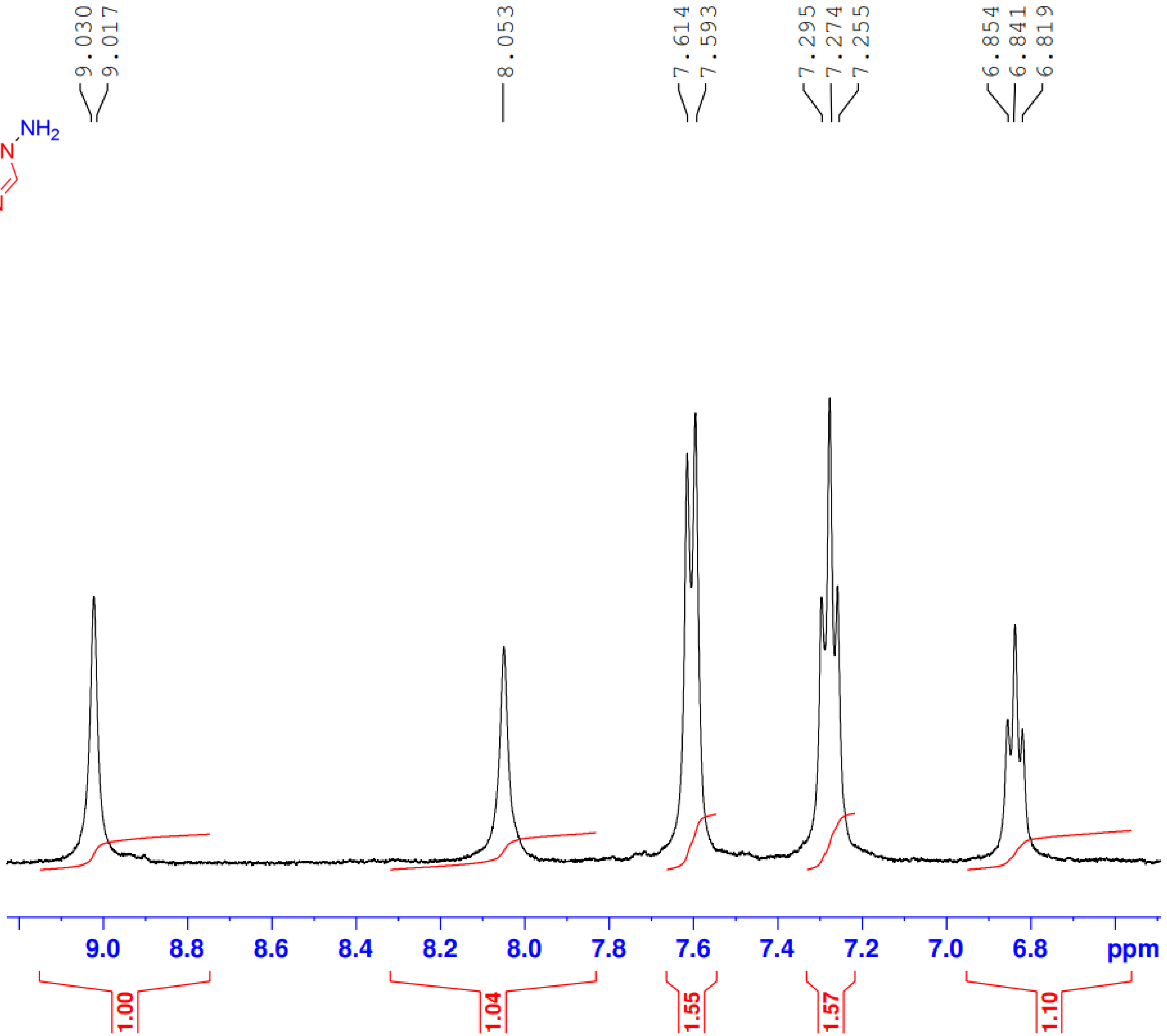


```
Current Data Parameters
NAME      Mohammed-attia-33
EXPNO                     1
PROCNO                    1

F2 - Acquisition Parameters
Date_              20210517
Time                  14.02
INSTRUM               spect
PROBHD    5 mm PABBO BB/
PULPROG                zg30
TD                    65536
SOLVENT                DMSO
NS                       33
DS                        2
SWH               8012.820 Hz
FIDRES            0.122266 Hz
AQ               4.0894465 sec
RG                   205.37
DW                   62.400 usec
DE                     6.50 usec
TE                    300.0 K
D1               1.00000000 sec
TD0                       1

======== CHANNEL f1 ========
SFO1           400.1524711 MHz
NUC1                     1H
P1                    12.00 usec
PLW1            18.00000000 W

F2 - Processing parameters
SI                    65536
SF             400.1500000 MHz
WDW                      EM
SSB       0
LB                     0.30 Hz
GB        0
PC                     1.00
```

D_2O

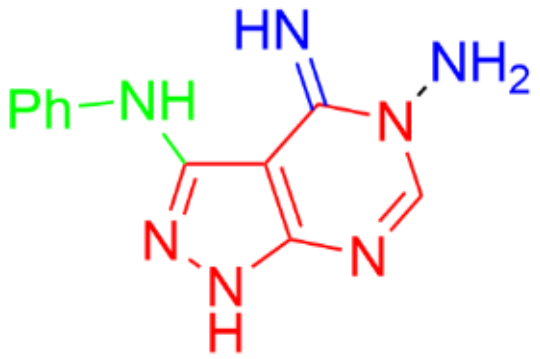

11

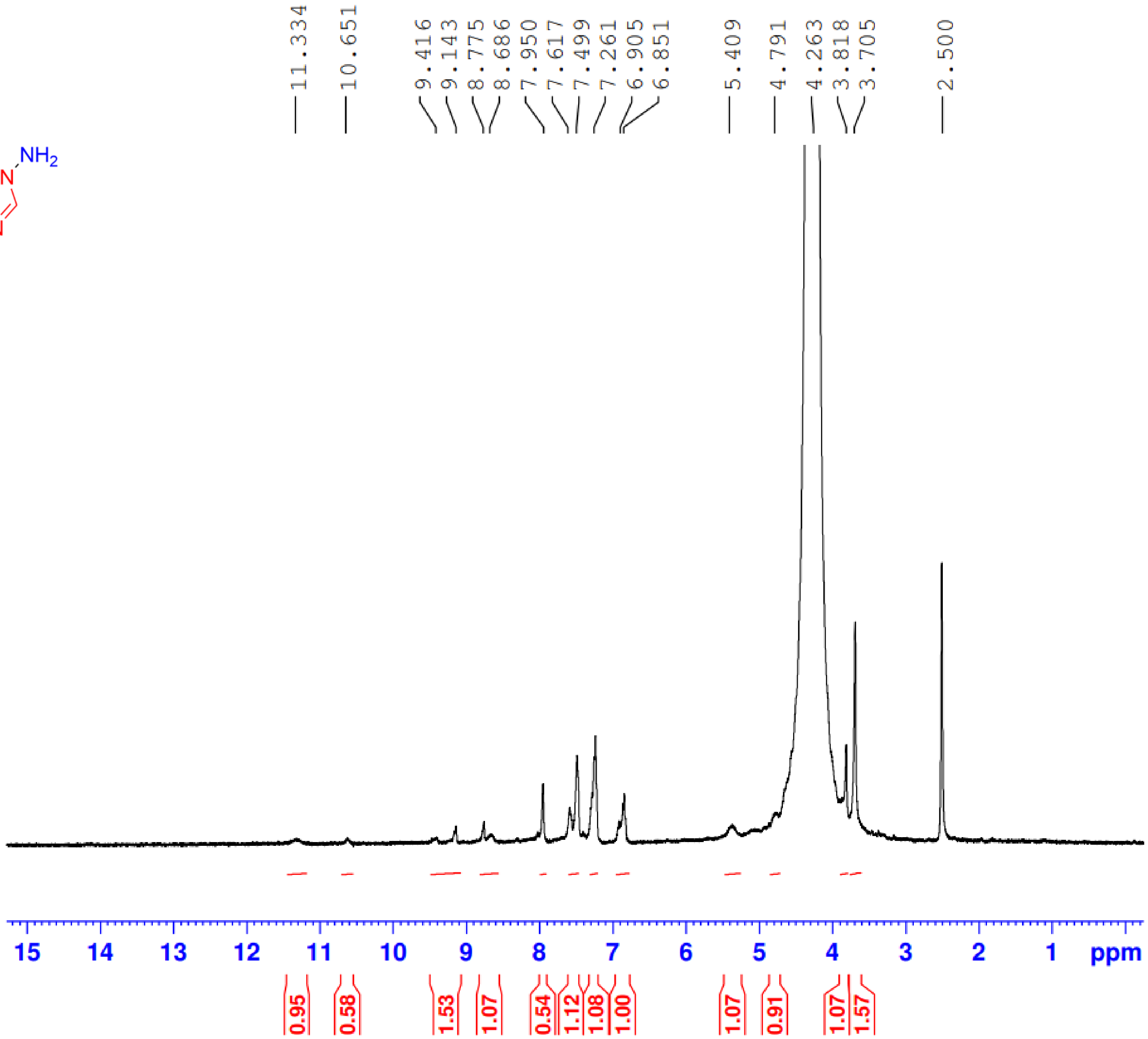


```
Current Data Parameters
NAME       Mohammed-attia-33-d2o
EXPNO                      2
PROCNO                     1

F2 - Acquisition Parameters
Date_               20210830
Time                   14.58
INSTRUM                spect
PROBHD    5 mm PABBO BB/
PULPROG                 zg30
TD                     65536
SOLVENT                 DMSO
NS                        18
DS                         2
SWH                 8012.820 Hz
FIDRES              0.122266 Hz
AQ                 4.0894465 sec
RG                     63.38
DW                    62.400 usec
DE                      6.50 usec
TE                     300.0 K
D1                1.00000000 sec
TD0                        1

======== CHANNEL f1 ========
SFO1             400.1524711 MHz
NUC1                      1H
P1                     12.00 usec
PLW1             18.00000000 W

F2 - Processing parameters
SI                     65536
SF               400.1500000 MHz
WDW                       EM
SSB       0
LB                      0.30 Hz
GB        0
PC                      1.00
```

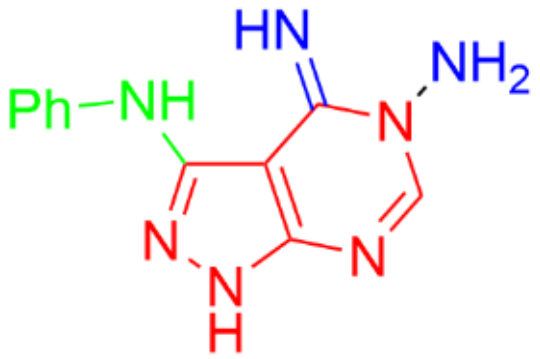

11

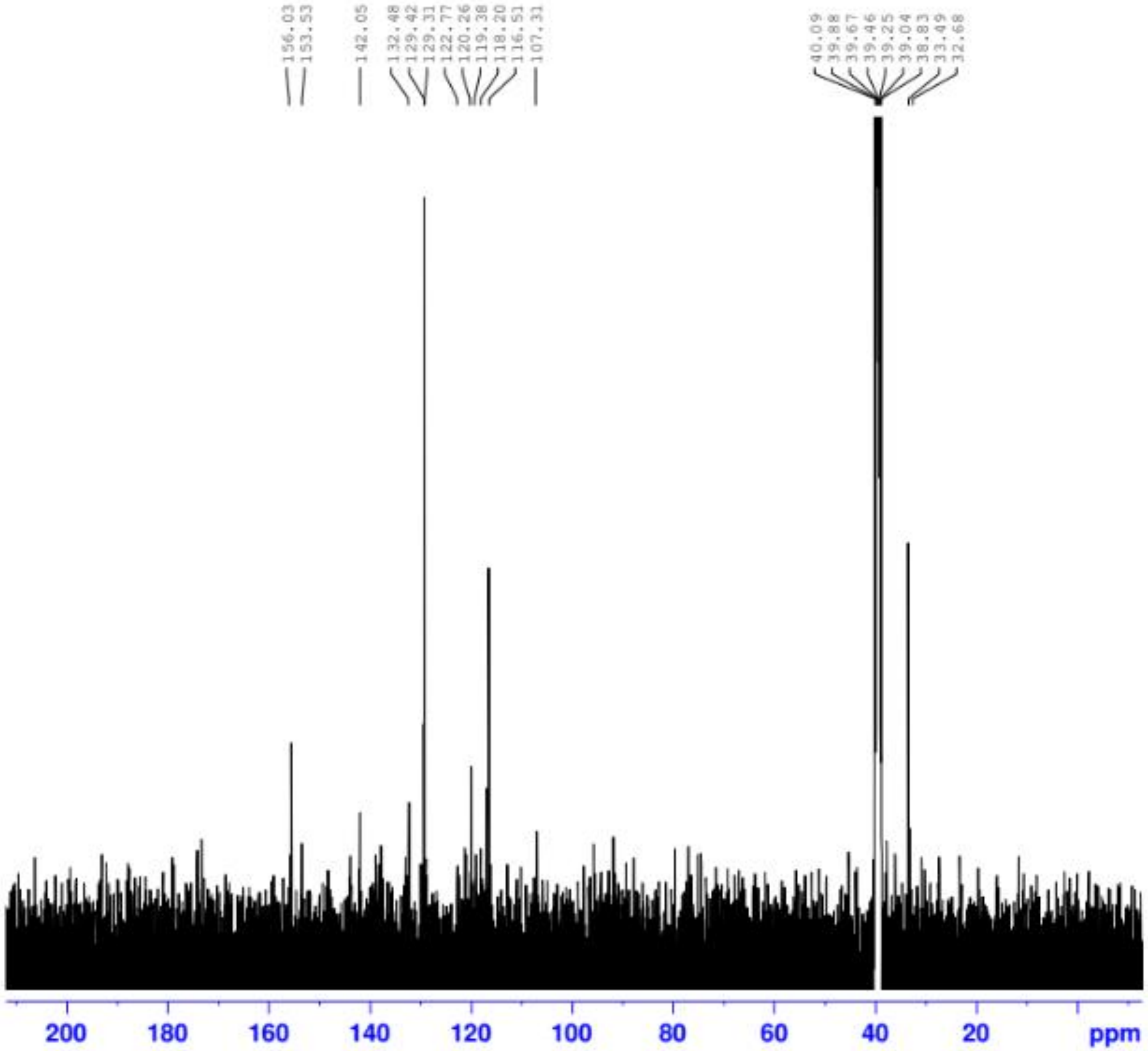

BRUKER

Current Data Parameters
NAME Mohammed-attia-33
EXPNO 2
PROCNO 1

F2 - Acquisition Parameters
Date_ 20210712
Time 14.49
INSTRUM spect
PROBHD 5 mm PABBO BB/
PULPROG zgpg30
TD 65536
SOLVENT DMSO
NS 2004
DS 4
SWH 24038.461 Hz
FIDRES 0.366798 Hz
AQ 1.3631488 sec
RG 205.37
DW 20.800 usec
DE 6.50 usec
TE 300.0 K
D1 2.00000000 sec
D11 0.03000000 sec
TD0 1

======== CHANNEL f1 ========
SFO1 100.6278588 MHz
NUC1 13C
P1 10.00 usec
PLW1 47.00000000 W

======== CHANNEL f2 ========
SFO2 400.1516006 MHz
NUC2 1H
CPDPRG[2 waltz16
PCPD2 90.00 usec
PLW2 18.00000000 W
PLW12 0.34722000 W
PLW13 0.28125000 W

F2 - Processing parameters
SI 32768
SF 100.6177975 MHz
WDW EM
SSB 0
LB 1.00 Hz
GB 0
PC 1.40

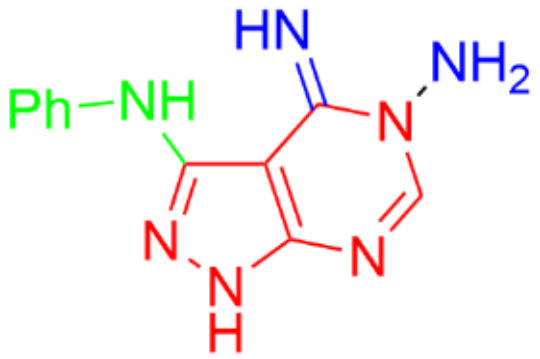

11

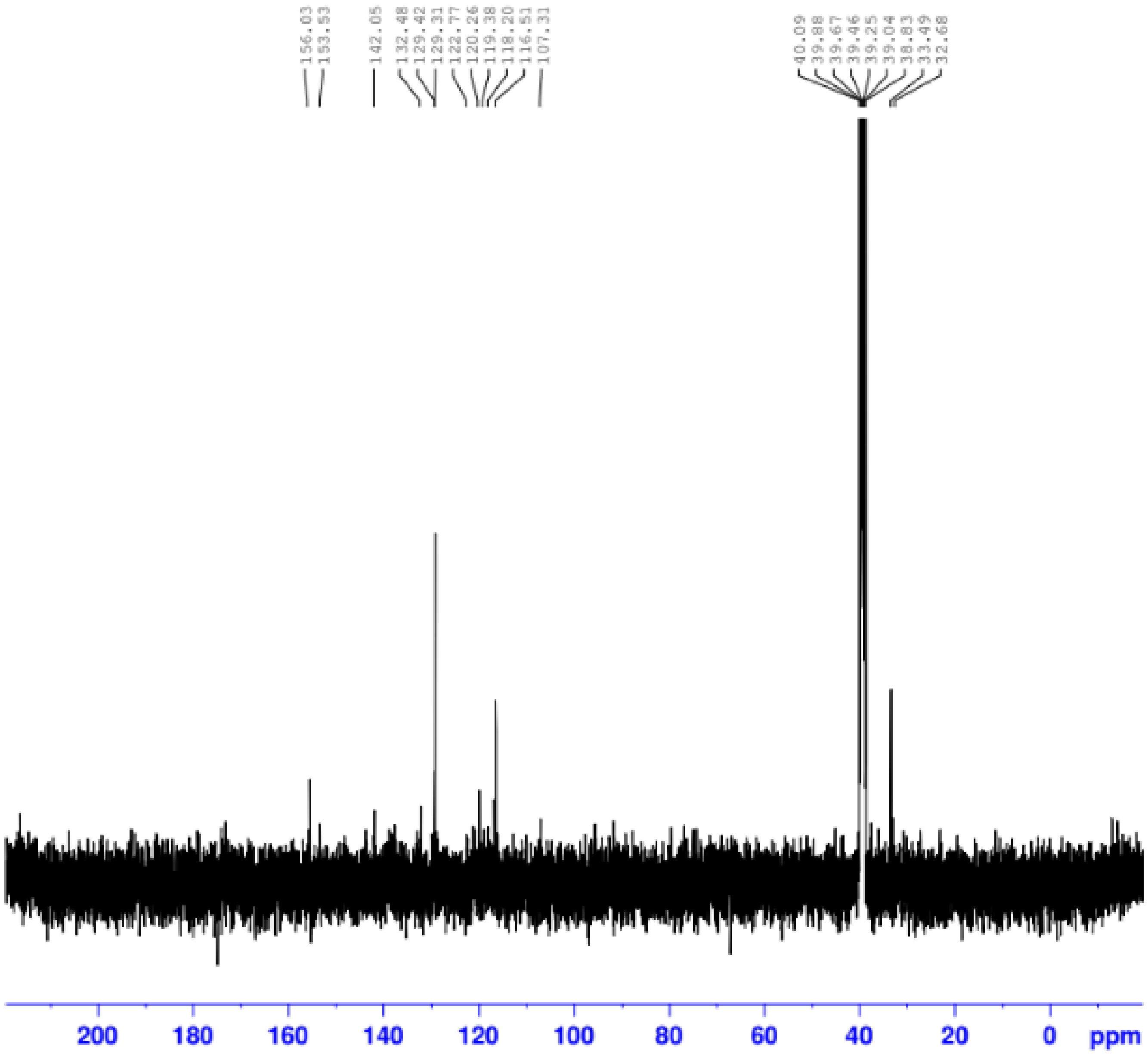

BRUKER

```
Current Data Parameters
NAME       Mohammed-attia-33
EXPNO                      2
PROCNO                     1

F2 - Acquisition Parameters
Date_               20210712
Time                   14.49
INSTRUM                spect
PROBHD    5 mm PABBO BB/
PULPROG              zgpg30
TD                    65536
SOLVENT                DMSO
NS                     2004
DS                        4
SWH               24038.461 Hz
FIDRES             0.366798 Hz
AQ                1.3631488 sec
RG                   205.37
DW                   20.800 usec
DE                     6.50 usec
TE                    300.0 K
D1               2.00000000 sec
D11              0.03000000 sec
TD0                       1

======== CHANNEL f1 ========
SFO1            100.6278588 MHz
NUC1                    13C
P1                    10.00 usec
PLW1            47.00000000 W

======== CHANNEL f2 ========
SFO2            400.1516006 MHz
NUC2                     1H
CPDPRG[2            waltz16
PCPD2                 90.00 usec
PLW2            18.00000000 W
PLW12            0.34722000 W
PLW13            0.28125000 W

F2 - Processing parameters
SI                    32768
SF              100.6177975 MHz
WDW                      EM
SSB       0
LB                     1.00 Hz
GB        0
PC                     1.40
```

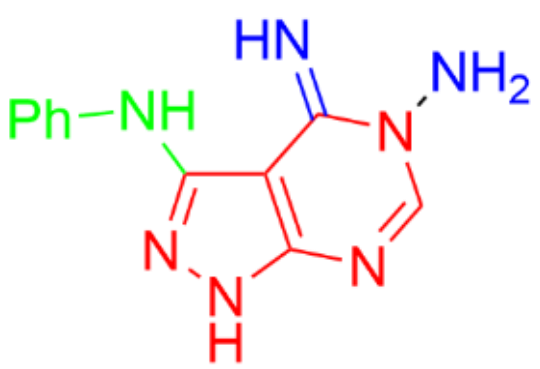

11

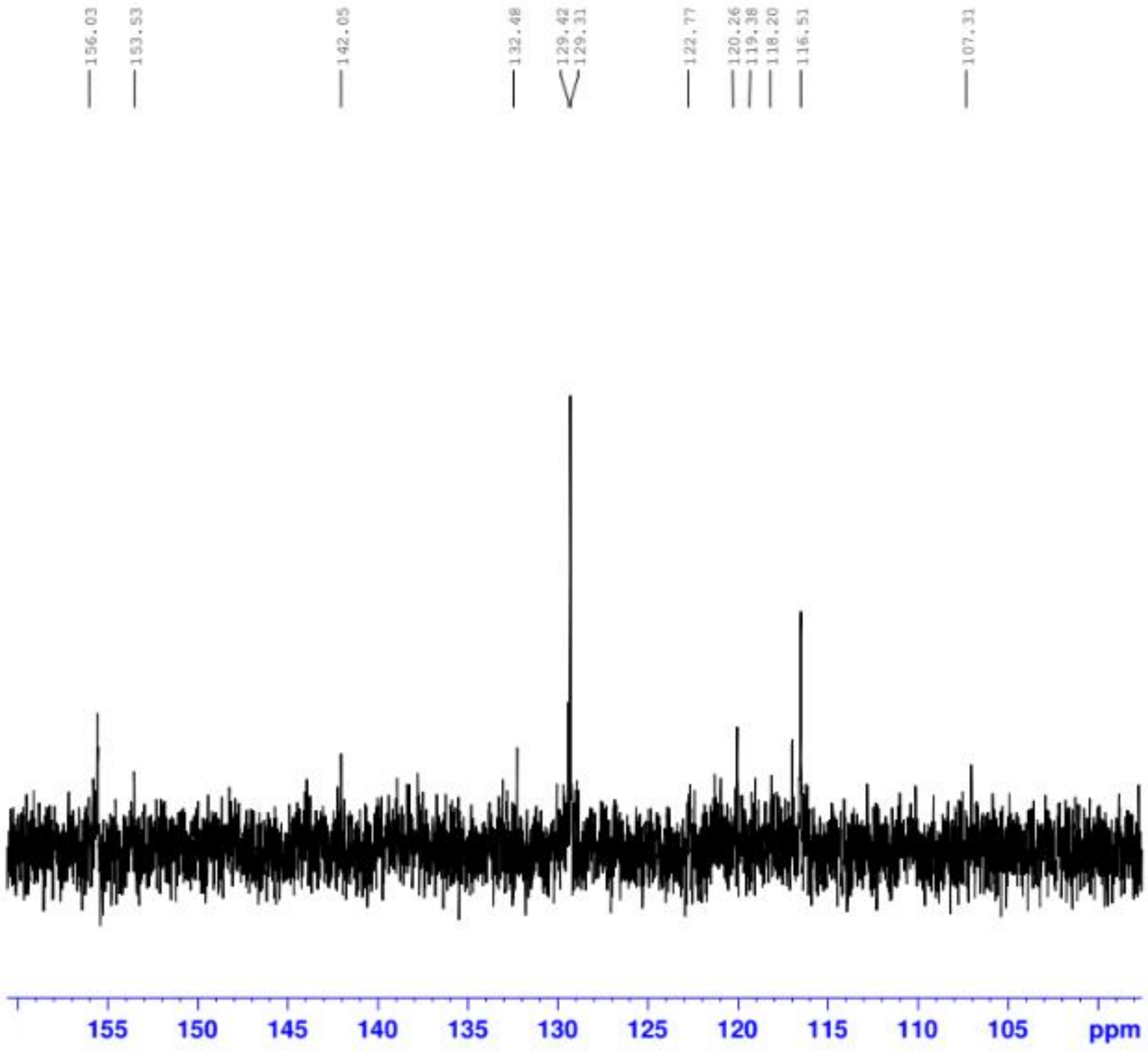

BRUKER

Current Data Parameters
NAME Mohammed-attia-33
EXPNO 2
PROCNO 1

F2 - Acquisition Parameters
Date_ 20210712
Time 14.49
INSTRUM spect
PROBHD 5 mm PABBO BB/
PULPROG zgpg30
TD 65536
SOLVENT DMSO
NS 2004
DS 4
SWH 24038.461 Hz
FIDRES 0.366798 Hz
AQ 1.3631488 sec
RG 205.37
DW 20.800 usec
DE 6.50 usec
TE 300.0 K
D1 2.00000000 sec
D11 0.03000000 sec
TD0 1

======== CHANNEL f1 ========
SFO1 100.6278588 MHz
NUC1 13C
P1 10.00 usec
PLW1 47.00000000 W

======== CHANNEL f2 ========
SFO2 400.1516006 MHz
NUC2 1H
CPDPRG[2 waltz16
PCPD2 90.00 usec
PLW2 18.00000000 W
PLW12 0.34722000 W
PLW13 0.28125000 W

F2 - Processing parameters
SI 32768
SF 100.6177975 MHz
WDW EM
SSB 0
LB 1.00 Hz
GB 0
PC 1.40

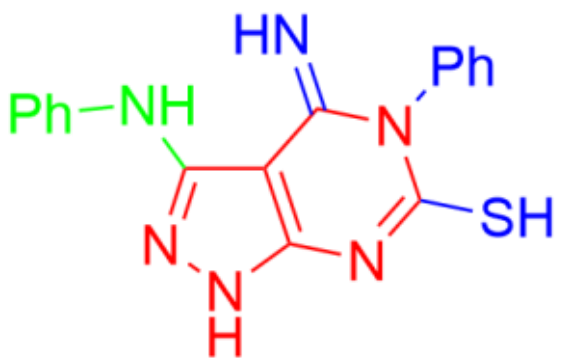

12

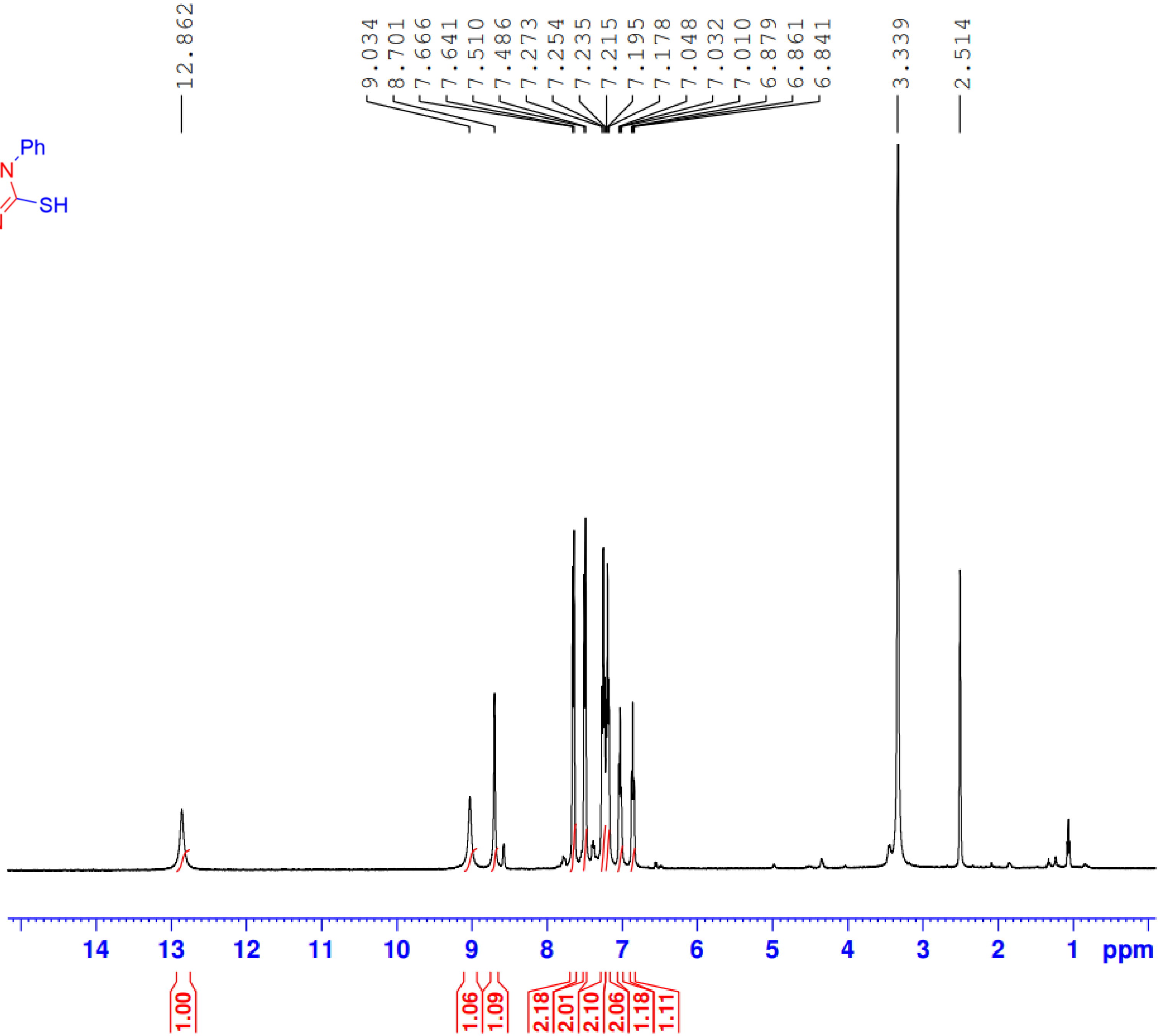

Current Data Parameters
NAME mohamed-hasaneen-3S
EXPNO 1
PROCNO 1

F2 - Acquisition Parameters
Date_ 20210221
Time 9.43
INSTRUM spect
PROBHD 5 mm PABBO BB/
PULPROG zg30
TD 65536
SOLVENT DMSO
NS 109
DS 2
SWH 8012.820 Hz
FIDRES 0.122266 Hz
AQ 4.0894465 sec
RG 205.37
DW 62.400 usec
DE 6.50 usec
TE 299.9 K
D1 1.00000000 sec
TD0 1

======== CHANNEL f1 ========
SFO1 400.1524711 MHz
NUC1 1H
P1 12.00 usec
PLW1 18.00000000 W

F2 - Processing parameters
SI 65536
SF 400.1500000 MHz
WDW EM
SSB 0
LB 0.30 Hz
GB 0
PC 1.00

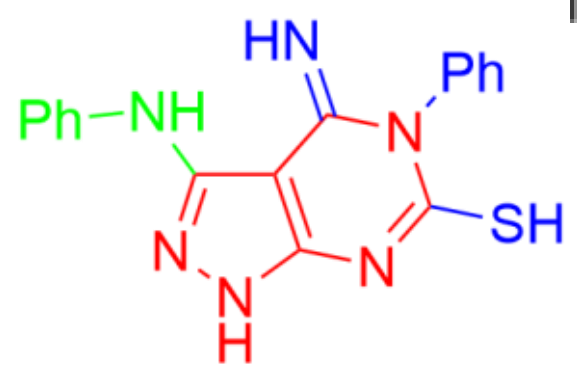

12

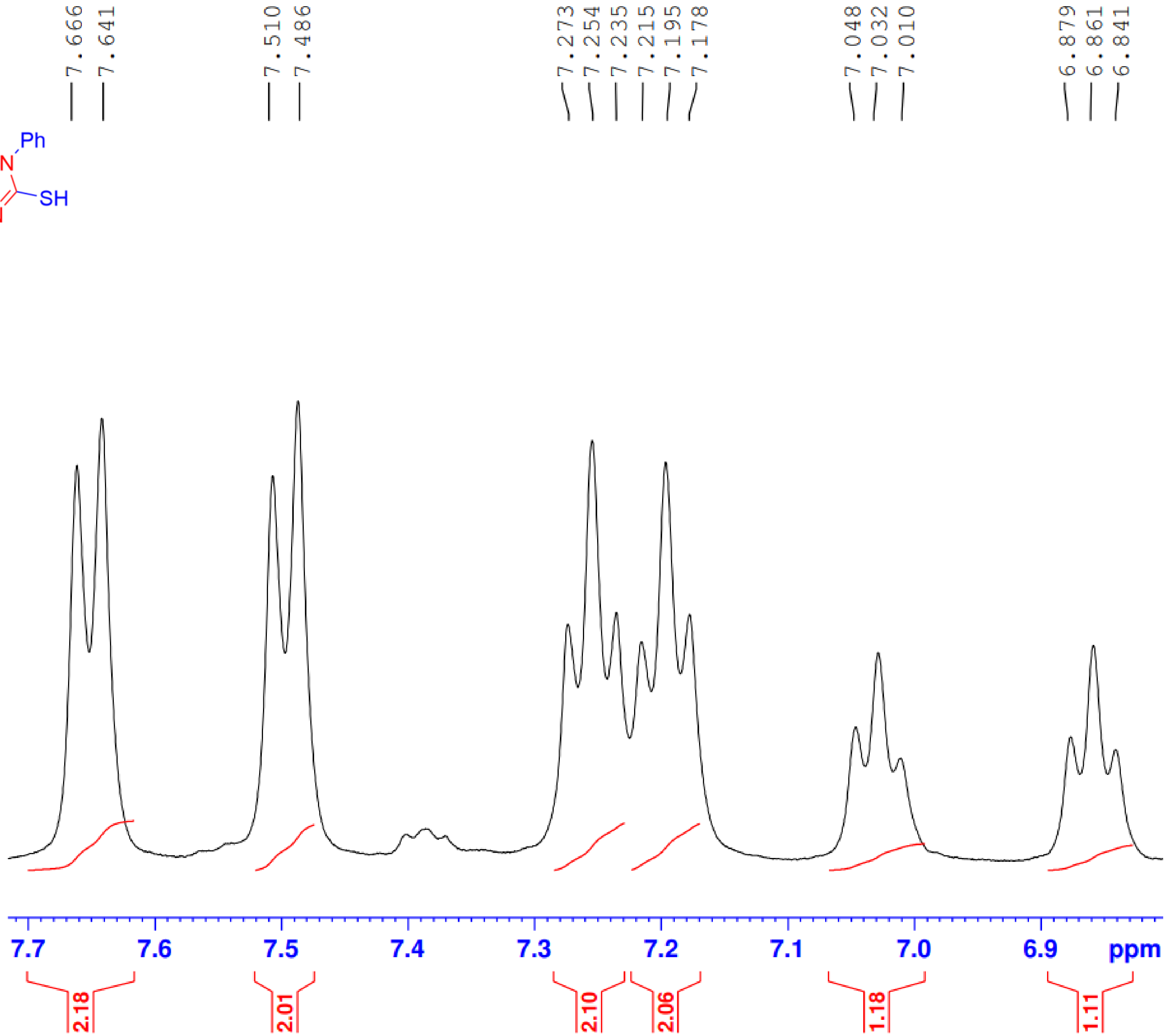

BRUKER

Current Data Parameters
NAME mohamed-hasaneen-3S
EXPNO 1
PROCNO 1

F2 - Acquisition Parameters
Date_ 20210221
Time 9.43
INSTRUM spect
PROBHD 5 mm PABBO BB/
PULPROG zg30
TD 65536
SOLVENT DMSO
NS 109
DS 2
SWH 8012.820 Hz
FIDRES 0.122266 Hz
AQ 4.0894465 sec
RG 205.37
DW 62.400 usec
DE 6.50 usec
TE 299.9 K
D1 1.00000000 sec
TD0 1

======== CHANNEL f1 ========
SFO1 400.1524711 MHz
NUC1 1H
P1 12.00 usec
PLW1 18.00000000 W

F2 - Processing parameters
SI 65536
SF 400.1500000 MHz
WDW EM
SSB 0
LB 0.30 Hz
GB 0
PC 1.00

D_2O

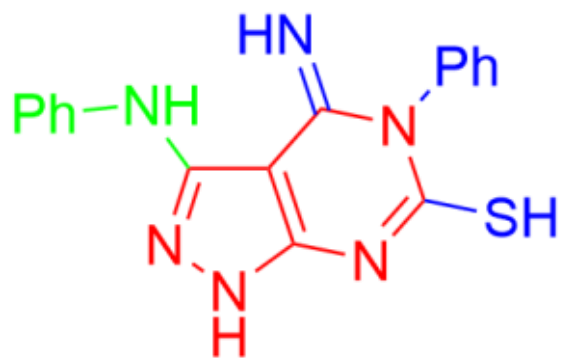

12

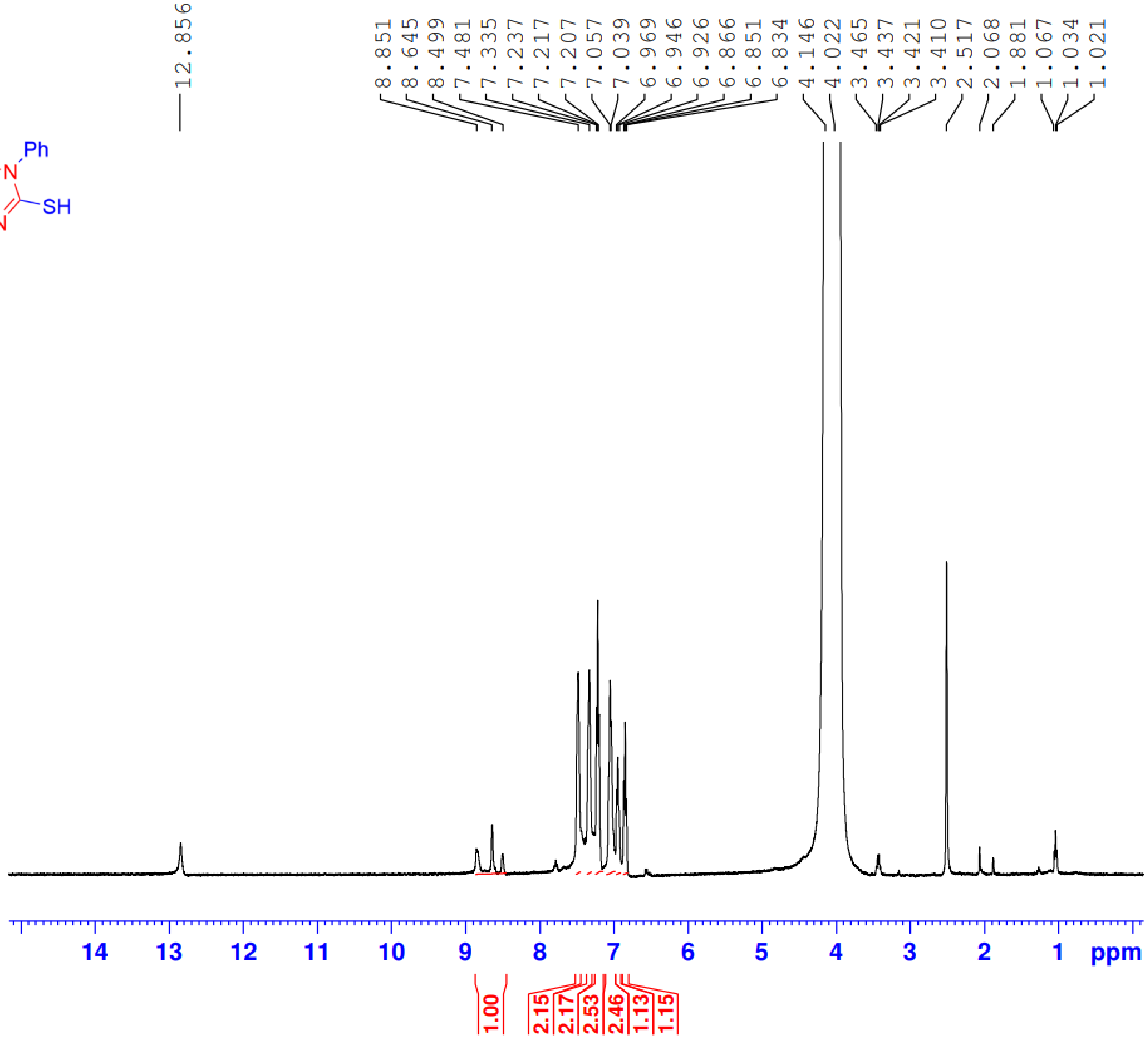


```
Current Data Parameters
NAME      mohamed-hasaneen-3S-d2o
EXPNO                    2
PROCNO                   1

F2 - Acquisition Parameters
Date_             20210830
Time                 14.34
INSTRUM              spect
PROBHD     5 mm PABBO BB/
PULPROG               zg30
TD                   65536
SOLVENT               DMSO
NS                      64
DS                       2
SWH            8012.820 Hz
FIDRES         0.122266 Hz
AQ            4.0894465 sec
RG                  117.48
DW               62.400 usec
DE                 6.50 usec
TE                 300.0 K
D1           1.00000000 sec
TD0                      1

======== CHANNEL f1 ========
SFO1        400.1524711 MHz
NUC1                    1H
P1                12.00 usec
PLW1        18.00000000 W

F2 - Processing parameters
SI                   65536
SF          400.1500000 MHz
WDW                     EM
SSB       0
LB                 0.30 Hz
GB        0
PC                    1.00
```

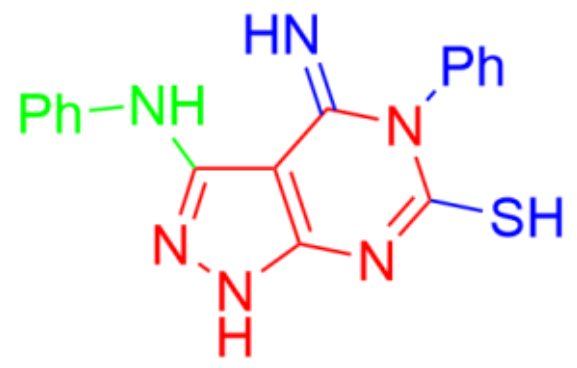

12

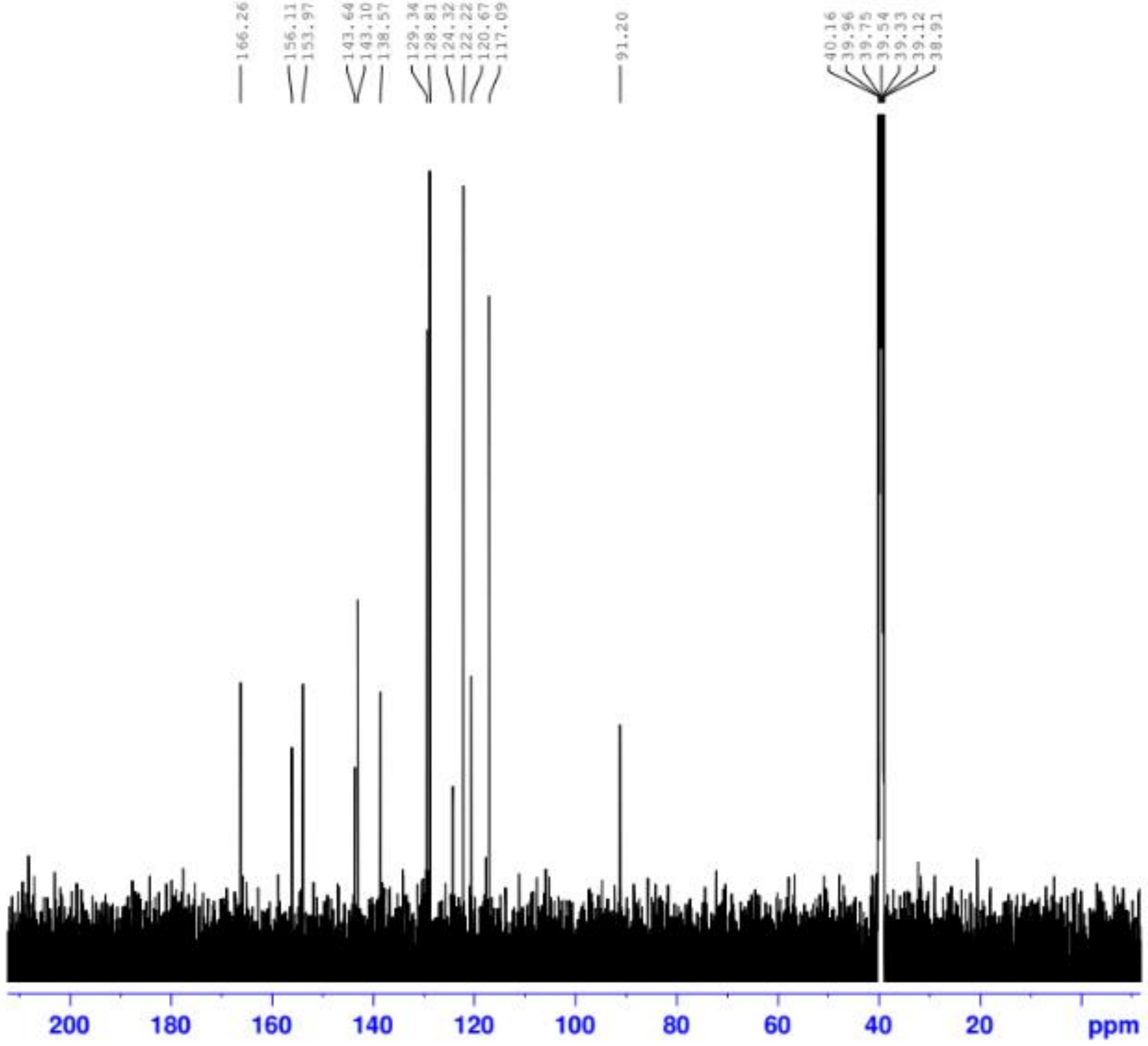


```
Current Data Parameters
NAME        Mohammed-attia-31
EXPNO                       2
PROCNO                      1

F2 - Acquisition Parameters
Date_                20210712
Time                    12.27
INSTRUM                 spect
PROBHD    5 mm PABBO BB/
PULPROG               zgpg30
TD                      65536
SOLVENT                  DMSO
NS                       2259
DS                          4
SWH                 24038.461 Hz
FIDRES               0.366798 Hz
AQ                  1.3631488 sec
RG                     205.37
DW                     20.800 usec
DE                       6.50 usec
TE                      300.0 K
D1                 2.00000000 sec
D11                0.03000000 sec
TD0                         1

======== CHANNEL f1 ========
SFO1              100.6278588 MHz
NUC1                      13C
P1                      10.00 usec
PLW1              47.00000000 W

======== CHANNEL f2 ========
SFO2              400.1516006 MHz
NUC2                       1H
CPDPRG[2              waltz16
PCPD2                   90.00 usec
PLW2              18.00000000 W
PLW12              0.34722000 W
PLW13              0.28125000 W

F2 - Processing parameters
SI                      32768
SF                100.6177975 MHz
WDW                        EM
SSB          0
LB                       1.00 Hz
GB           0
PC                       1.40
```

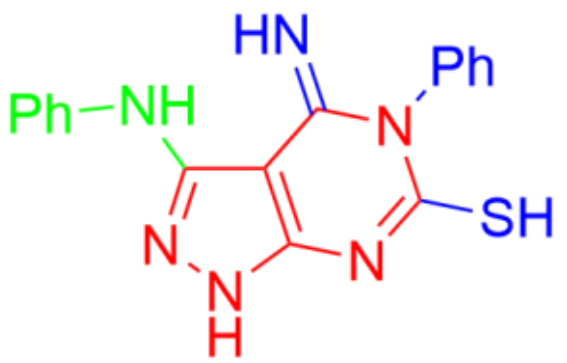
HN
Ph
Ph–NH
N
SH
N
N
N
H

12

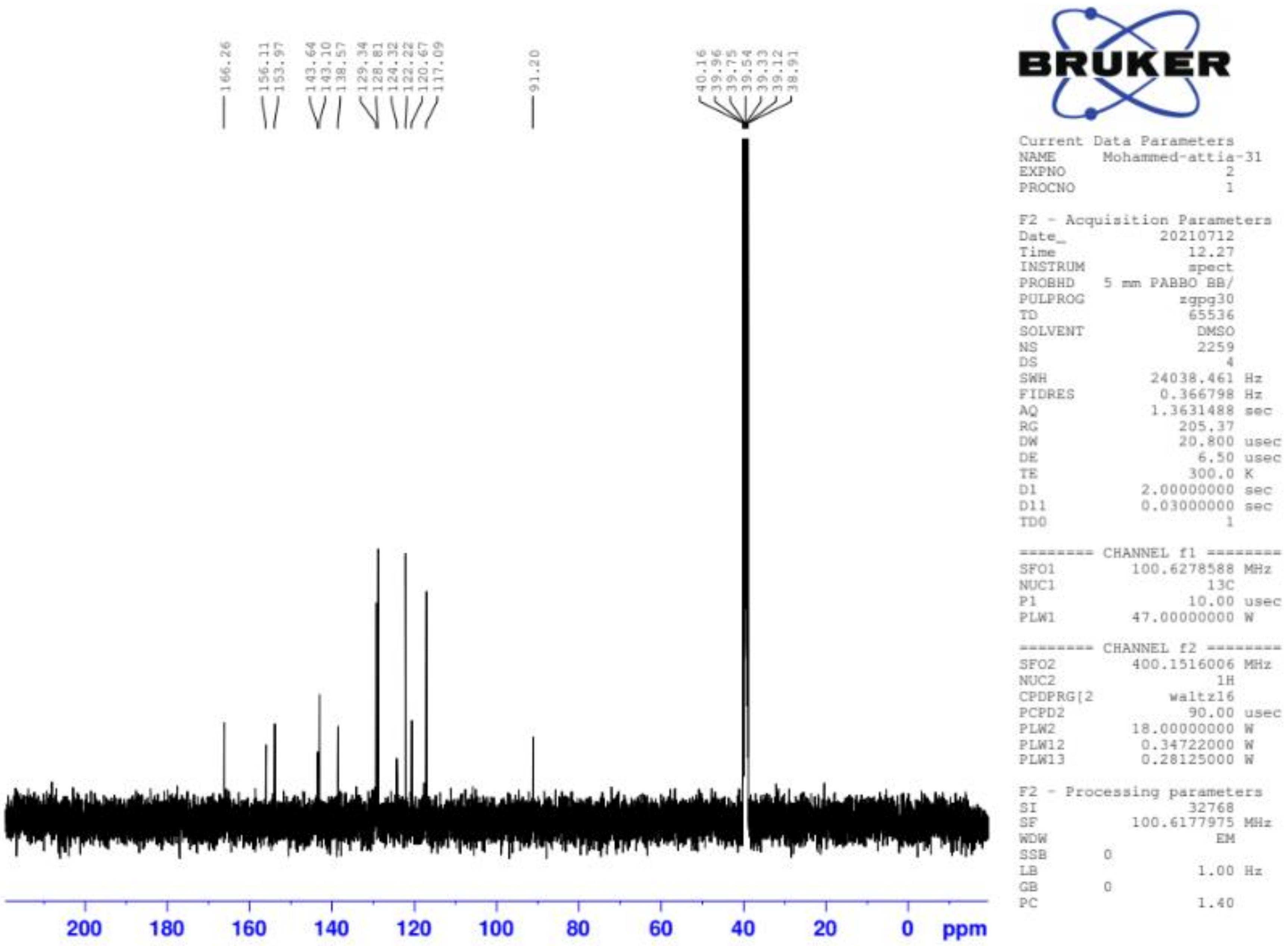
166.26
156.11
153.97
143.64
143.10
138.57
129.34
128.81
124.32
122.22
120.67
117.09
91.20
40.16
39.96
39.75
39.54
39.33
39.12
38.91
200
180
160
140
120
100
80
60
40
20
0
ppm
BRUKER
Current Data Parameters
NAME Mohammed-attia-31
EXPNO 2
PROCNO 1
F2 - Acquisition Parameters
Date_ 20210712
Time 12.27
INSTRUM spect
PROBHD 5 mm PABBO BB/
PULPROG zgpg30
TD 65536
SOLVENT DMSO
NS 2259
DS 4
SWH 24038.461 Hz
FIDRES 0.366798 Hz
AQ 1.3631488 sec
RG 205.37
DW 20.800 usec
DE 6.50 usec
TE 300.0 K
D1 2.00000000 sec
D11 0.03000000 sec
TD0 1
======== CHANNEL f1 ========
SFO1 100.6278588 MHz
NUC1 13C
P1 10.00 usec
PLW1 47.00000000 W
======== CHANNEL f2 ========
SFO2 400.1516006 MHz
NUC2 1H
CPDPRG[2 waltz16
PCPD2 90.00 usec
PLW2 18.00000000 W
PLW12 0.34722000 W
PLW13 0.28125000 W
F2 - Processing parameters
SI 32768
SF 100.6177975 MHz
WDW EM
SSB 0
LB 1.00 Hz
GB 0
PC 1.40

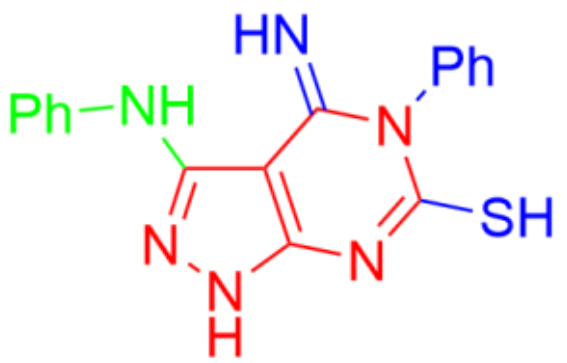

12

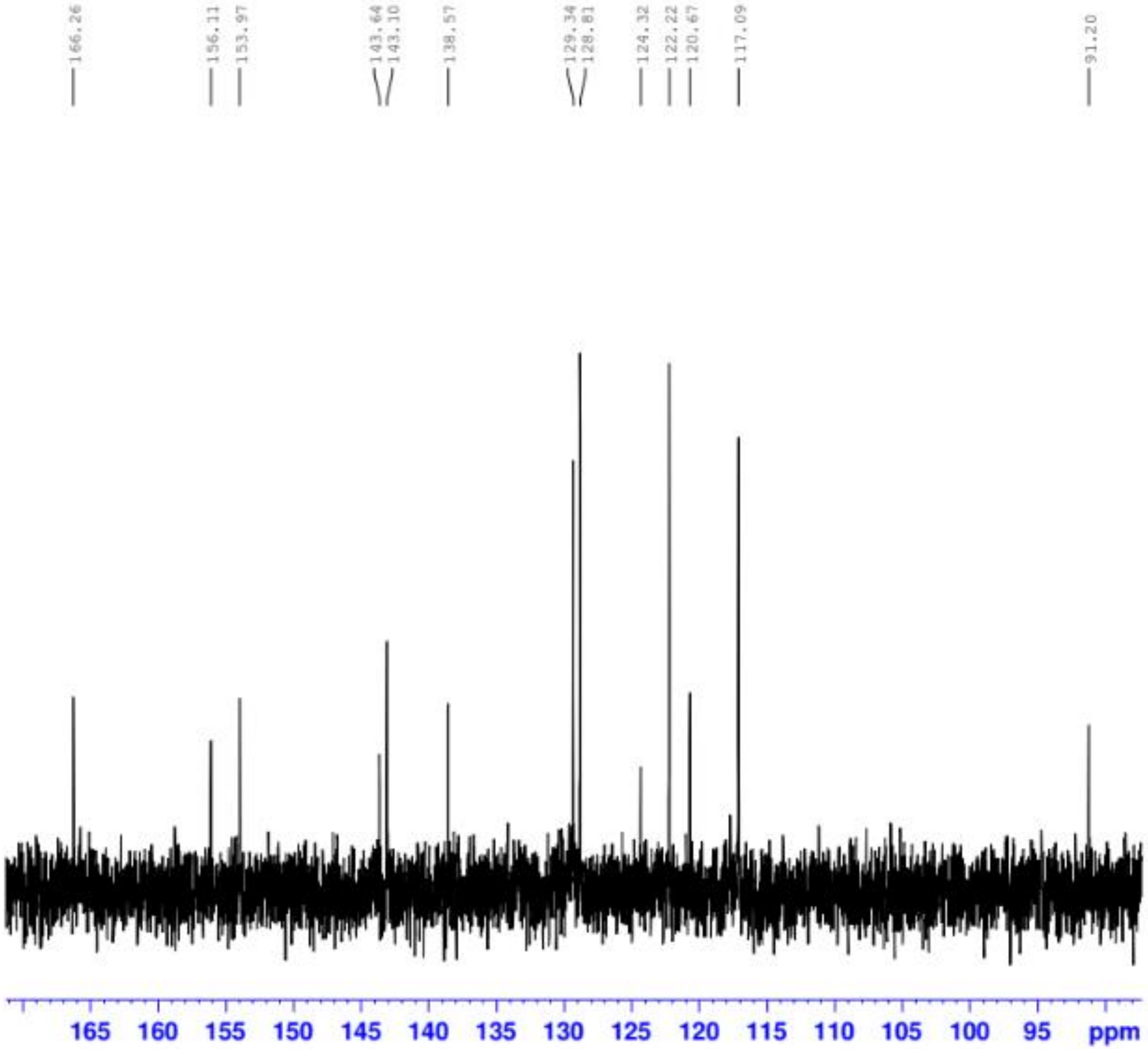

BRUKER

Current Data Parameters
NAME Mohammed-attia-31
EXPNO 2
PROCNO 1

F2 - Acquisition Parameters
Date_ 20210712
Time 12.27
INSTRUM spect
PROBHD 5 mm PABBO BB/
PULPROG zgpg30
TD 65536
SOLVENT DMSO
NS 2259
DS 4
SWH 24038.461 Hz
FIDRES 0.366798 Hz
AQ 1.3631488 sec
RG 205.37
DW 20.800 usec
DE 6.50 usec
TE 300.0 K
D1 2.00000000 sec
D11 0.03000000 sec
TD0 1

======== CHANNEL f1 ========
SFO1 100.6278588 MHz
NUC1 13C
P1 10.00 usec
PLW1 47.00000000 W

======== CHANNEL f2 ========
SFO2 400.1516006 MHz
NUC2 1H
CPDPRG[2 waltz16
PCPD2 90.00 usec
PLW2 18.00000000 W
PLW12 0.34722000 W
PLW13 0.28125000 W

F2 - Processing parameters
SI 32768
SF 100.6177975 MHz
WDW EM
SSB 0
LB 1.00 Hz
GB 0
PC 1.40

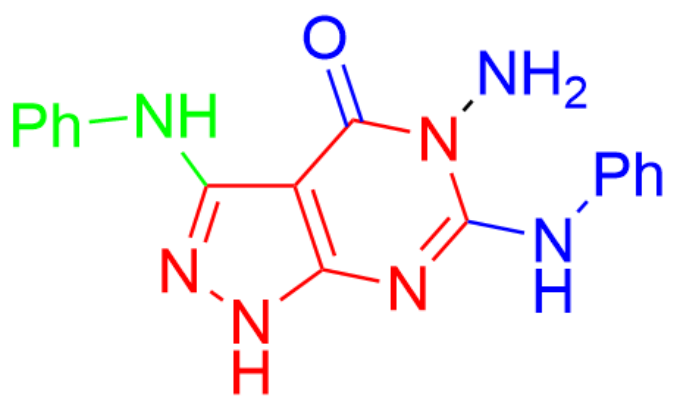

13

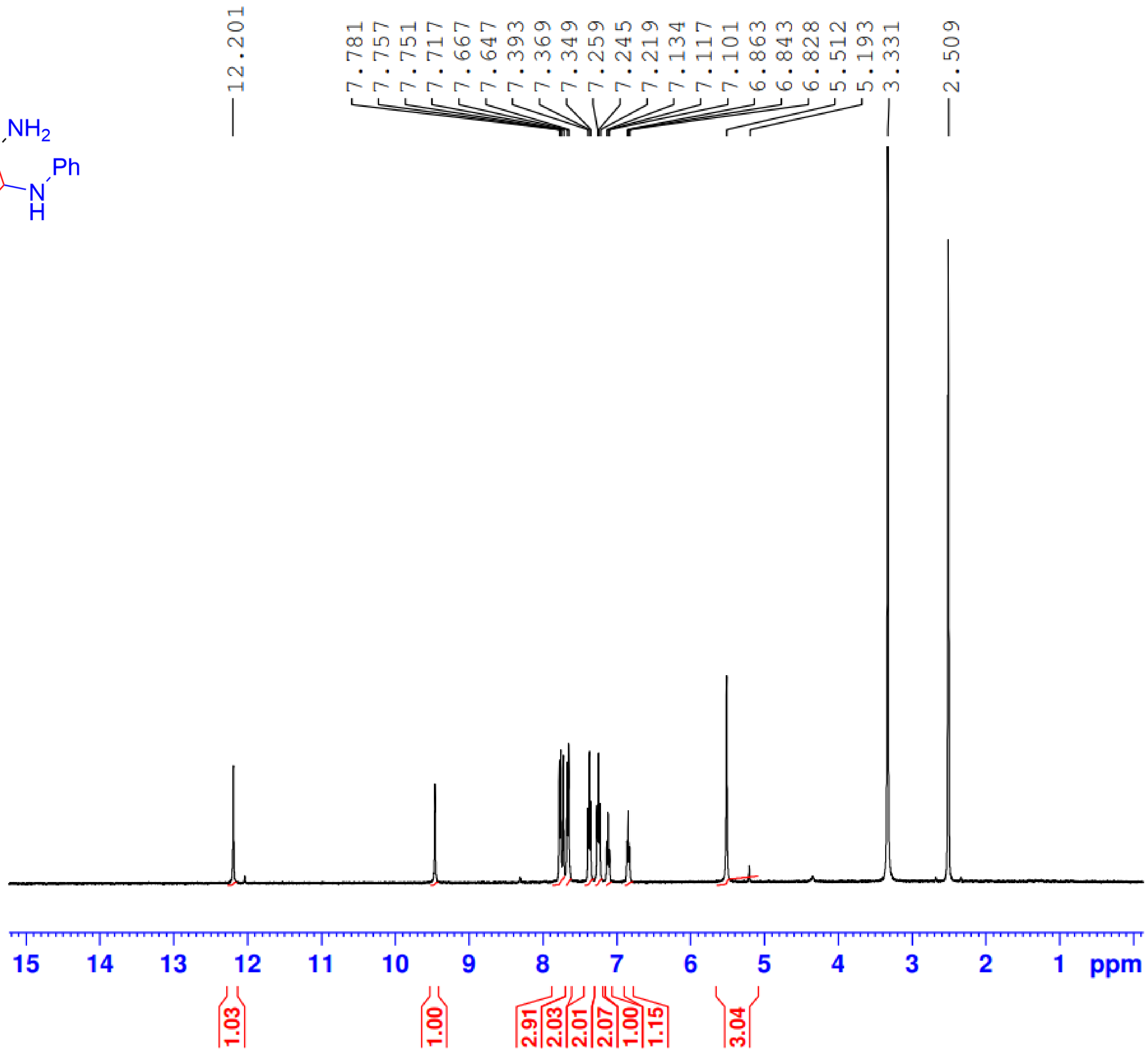

Current Data Parameters
NAME Mohammed-attia-38
EXPNO 1
PROCNO 1

F2 - Acquisition Parameters
Date_ 20210517
Time 13.30
INSTRUM spect
PROBHD 5 mm PABBO BB/
PULPROG zg30
TD 65536
SOLVENT DMSO
NS 34
DS 2
SWH 8012.820 Hz
FIDRES 0.122266 Hz
AQ 4.0894465 sec
RG 205.37
DW 62.400 usec
DE 6.50 usec
TE 300.0 K
D1 1.00000000 sec
TD0 1

======== CHANNEL f1 ========
SFO1 400.1524711 MHz
NUC1 1H
P1 12.00 usec
PLW1 18.00000000 W

F2 - Processing parameters
SI 65536
SF 400.1500000 MHz
WDW EM
SSB 0
LB 0.30 Hz
GB 0
PC 1.00

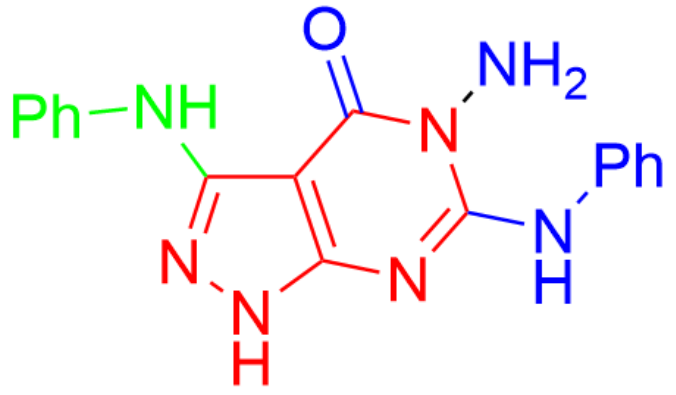

13

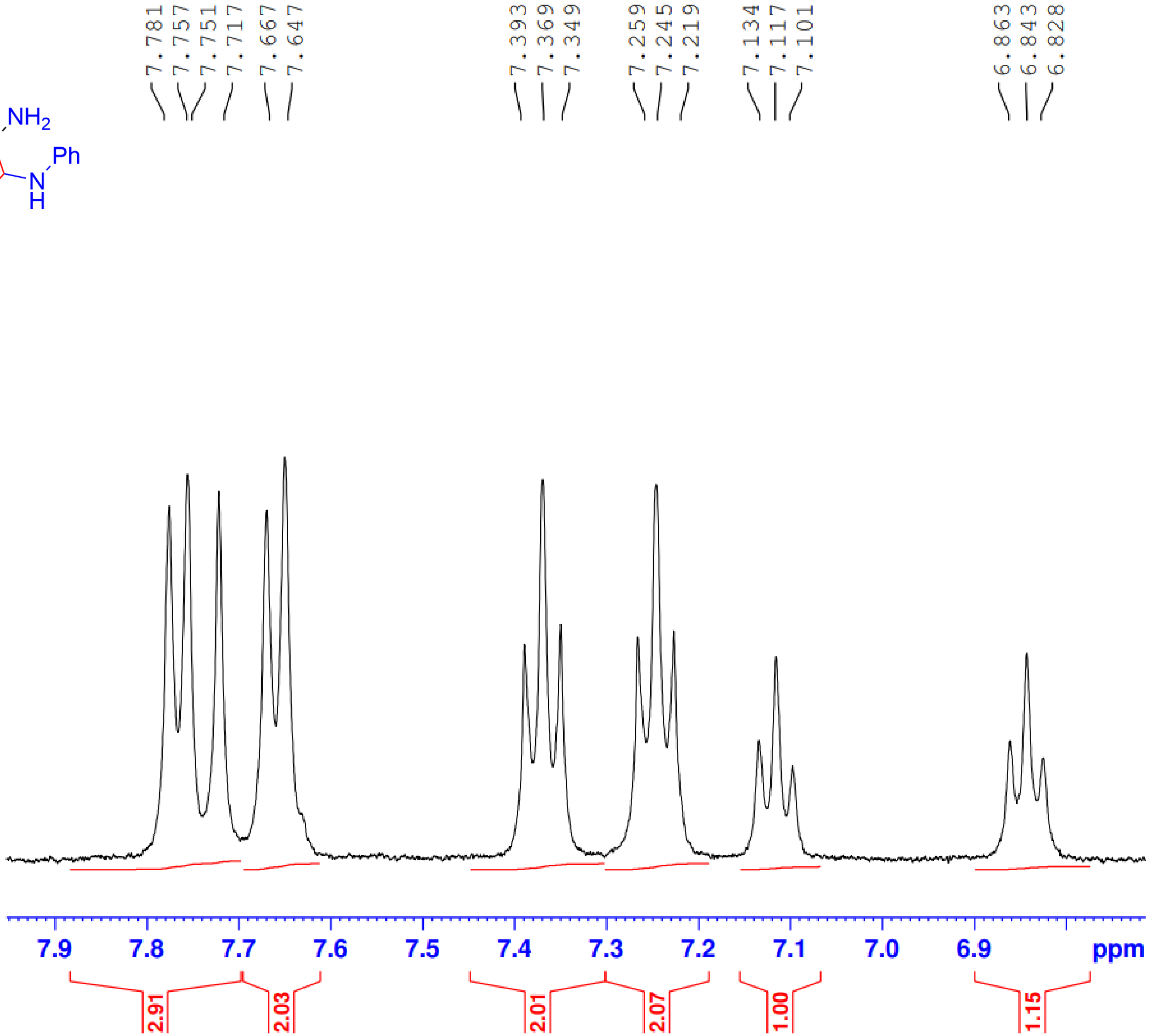

BRUKER

Current Data Parameters
NAME Mohammed-attia-38
EXPNO 1
PROCNO 1

F2 - Acquisition Parameters
Date_ 20210517
Time 13.30
INSTRUM spect
PROBHD 5 mm PABBO BB/
PULPROG zg30
TD 65536
SOLVENT DMSO
NS 34
DS 2
SWH 8012.820 Hz
FIDRES 0.122266 Hz
AQ 4.0894465 sec
RG 205.37
DW 62.400 usec
DE 6.50 usec
TE 300.0 K
D1 1.00000000 sec
TD0 1

======== CHANNEL f1 ========
SFO1 400.1524711 MHz
NUC1 1H
P1 12.00 usec
PLW1 18.00000000 W

F2 - Processing parameters
SI 65536
SF 400.1500000 MHz
WDW EM
SSB 0
LB 0.30 Hz
GB 0
PC 1.00

D_2O

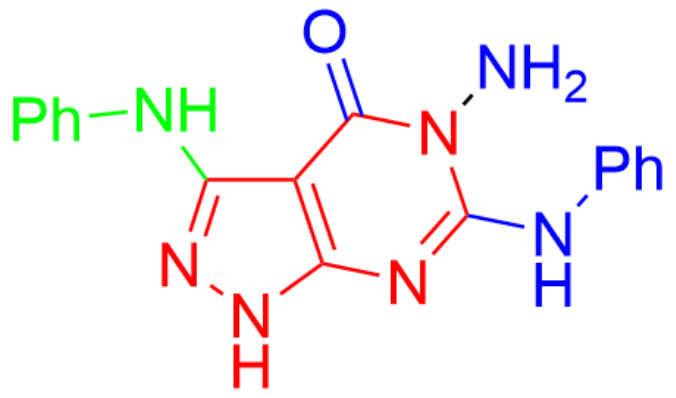

13

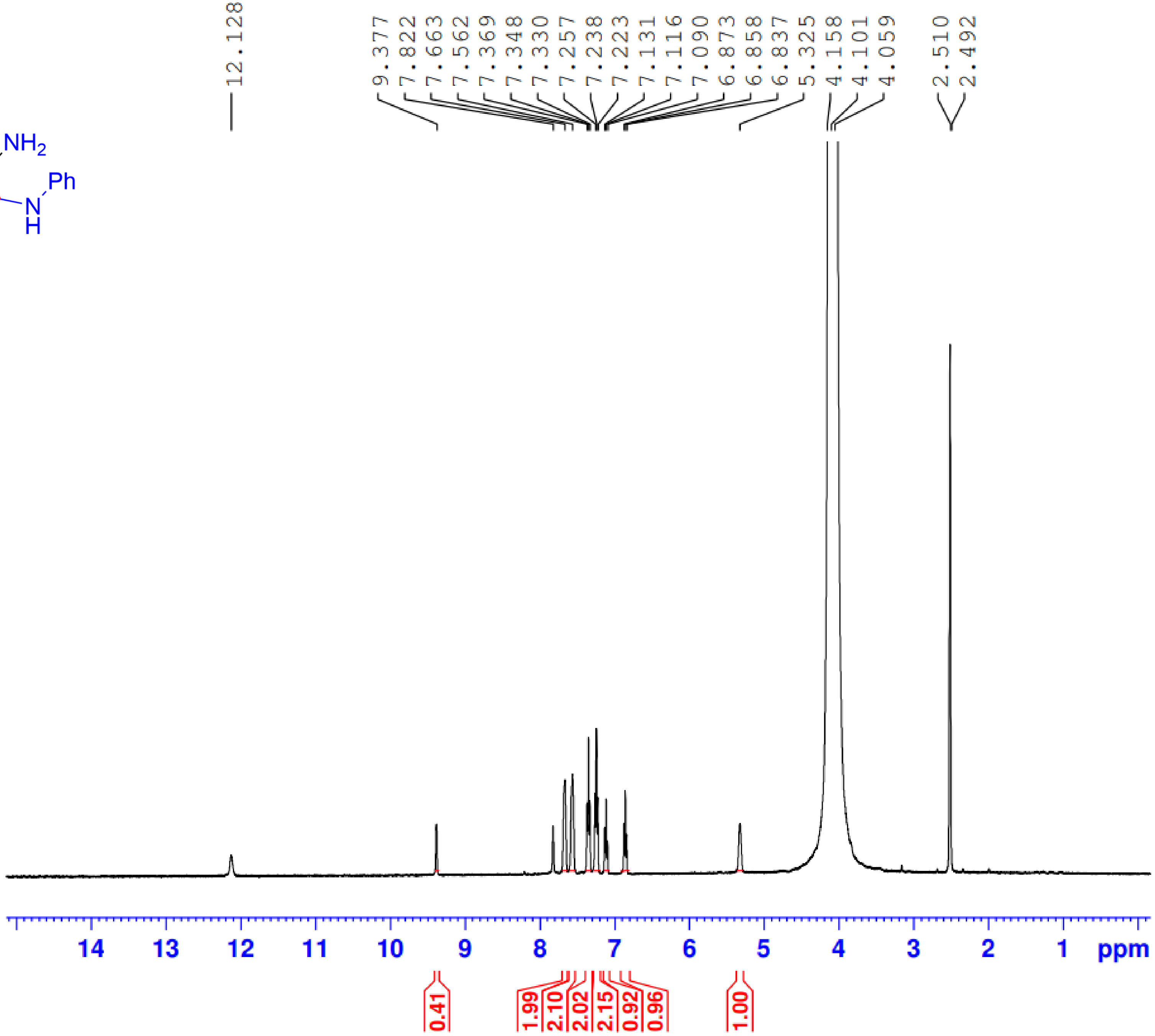

BRUKER

Current Data Parameters
NAME Mohammed-attia-38-d2o
EXPNO 2
PROCNO 1

F2 - Acquisition Parameters
Date_ 20210830
Time 13.59
INSTRUM spect
PROBHD 5 mm PABBO BB/
PULPROG zg30
TD 65536
SOLVENT DMSO
NS 64
DS 2
SWH 8012.820 Hz
FIDRES 0.122266 Hz
AQ 4.0894465 sec
RG 81.16
DW 62.400 usec
DE 6.50 usec
TE 300.0 K
D1 1.00000000 sec
TD0 1

======== CHANNEL f1 ========
SFO1 400.1524711 MHz
NUC1 1H
P1 12.00 usec
PLW1 18.00000000 W

F2 - Processing parameters
SI 65536
SF 400.1500000 MHz
WDW EM
SSB 0
LB 0.30 Hz
GB 0
PC 1.00

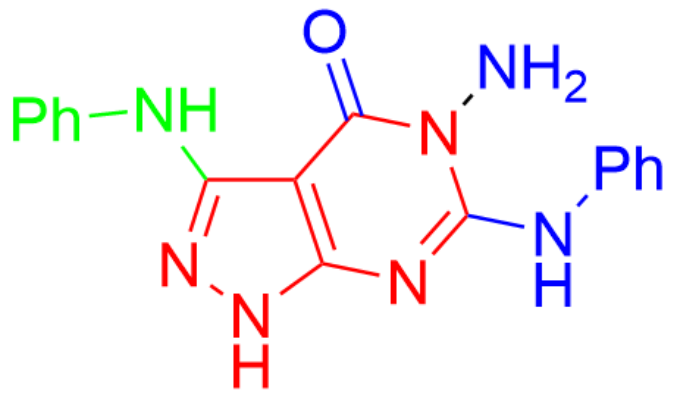

O
NH2
Ph–NH
N
Ph
N
N
N
H
N
H

13

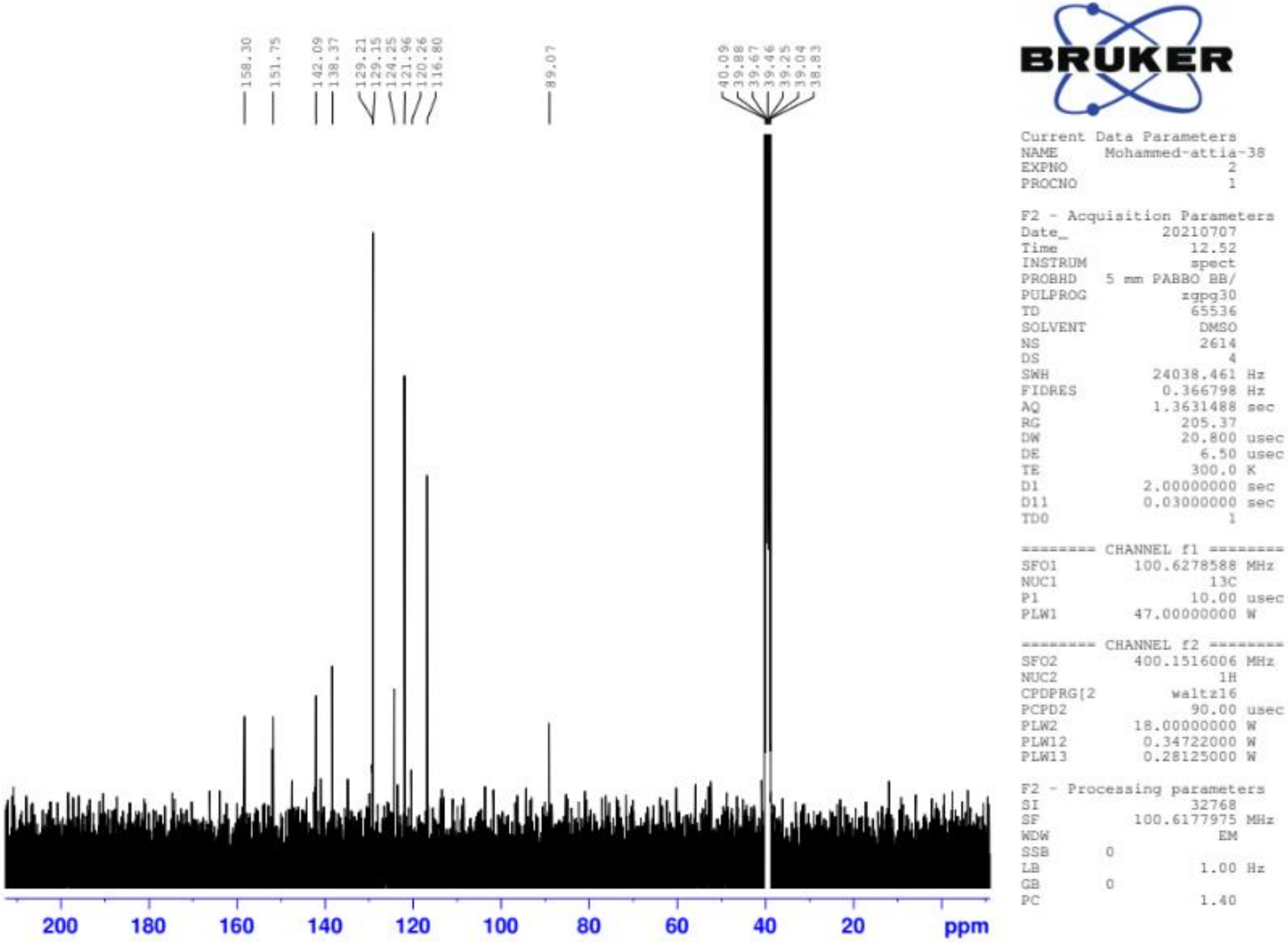

158.30
151.75
142.09
138.37
129.21
129.15
124.25
121.96
120.26
116.80
89.07
40.09
39.88
39.67
39.46
39.25
39.04
38.83
200
180
160
140
120
100
80
60
40
20
ppm
BRUKER
Current Data Parameters
NAME Mohammed-attia-38
EXPNO 2
PROCNO 1
F2 - Acquisition Parameters
Date_ 20210707
Time 12.52
INSTRUM spect
PROBHD 5 mm PABBO BB/
PULPROG zgpg30
TD 65536
SOLVENT DMSO
NS 2614
DS 4
SWH 24038.461 Hz
FIDRES 0.366798 Hz
AQ 1.3631488 sec
RG 205.37
DW 20.800 usec
DE 6.50 usec
TE 300.0 K
D1 2.00000000 sec
D11 0.03000000 sec
TD0 1
======== CHANNEL f1 ========
SFO1 100.6278588 MHz
NUC1 13C
P1 10.00 usec
PLW1 47.00000000 W
======== CHANNEL f2 ========
SFO2 400.1516006 MHz
NUC2 1H
CPDPRG[2 waltz16
PCPD2 90.00 usec
PLW2 18.00000000 W
PLW12 0.34722000 W
PLW13 0.28125000 W
F2 - Processing parameters
SI 32768
SF 100.6177975 MHz
WDW EM
SSB 0
LB 1.00 Hz
GB 0
PC 1.40

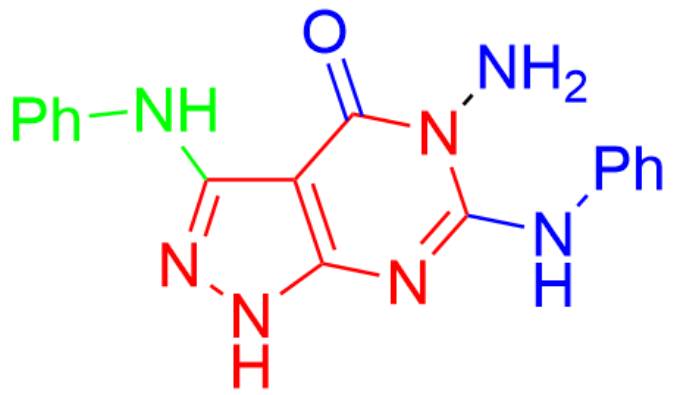

13

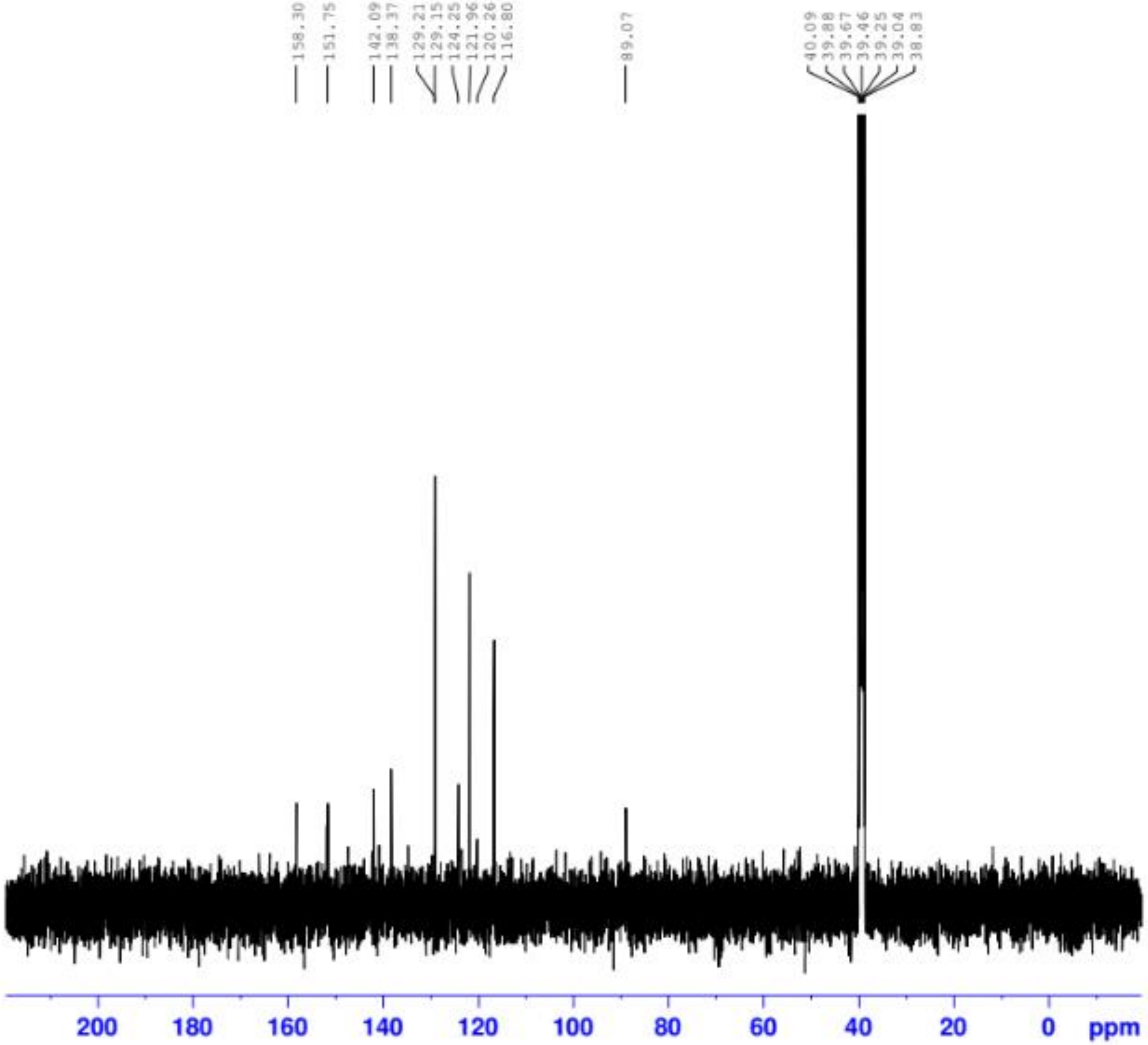

BRUKER

Current Data Parameters
NAME Mohammed-attia-38
EXPNO 2
PROCNO 1

F2 - Acquisition Parameters
Date_ 20210707
Time 12.52
INSTRUM spect
PROBHD 5 mm PABBO BB/
PULPROG zgpg30
TD 65536
SOLVENT DMSO
NS 2614
DS 4
SWH 24038.461 Hz
FIDRES 0.366798 Hz
AQ 1.3631488 sec
RG 205.37
DW 20.800 usec
DE 6.50 usec
TE 300.0 K
D1 2.00000000 sec
D11 0.03000000 sec
TD0 1

======== CHANNEL f1 ========
SFO1 100.6278588 MHz
NUC1 13C
P1 10.00 usec
PLW1 47.00000000 W

======== CHANNEL f2 ========
SFO2 400.1516006 MHz
NUC2 1H
CPDPRG[2 waltz16
PCPD2 90.00 usec
PLW2 18.00000000 W
PLW12 0.34722000 W
PLW13 0.28125000 W

F2 - Processing parameters
SI 32768
SF 100.6177975 MHz
WDW EM
SSB 0
LB 1.00 Hz
GB 0
PC 1.40

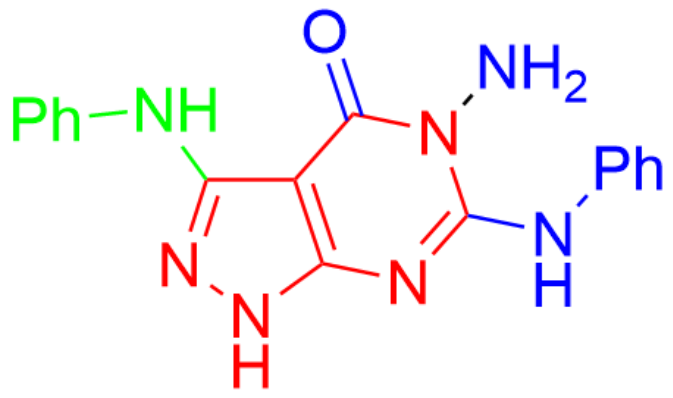

13

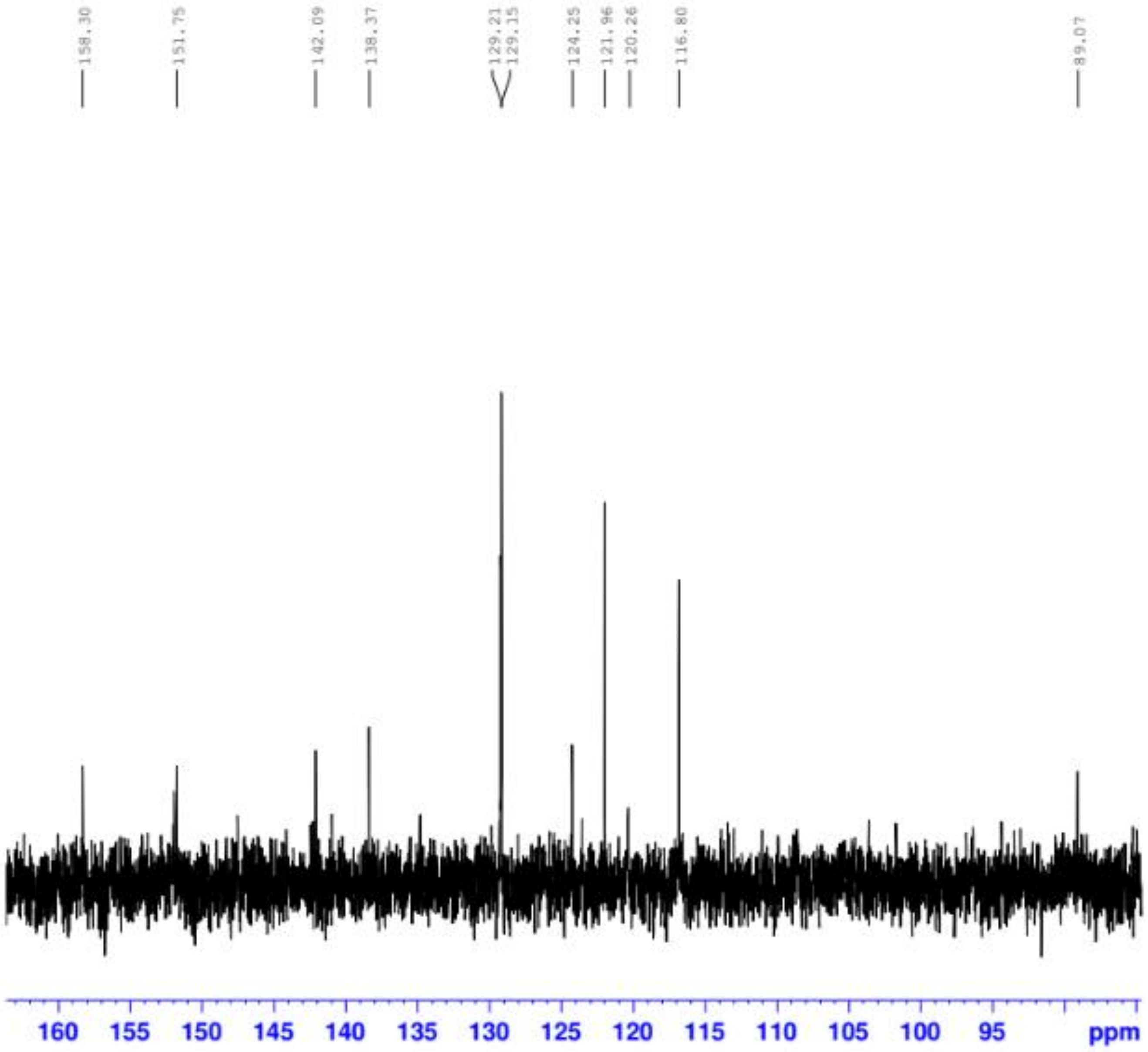

Current Data Parameters
NAME Mohammed-attia-38
EXPNO 2
PROCNO 1

F2 - Acquisition Parameters
Date_ 20210707
Time 12.52
INSTRUM spect
PROBHD 5 mm PABBO BB/
PULPROG zgpg30
TD 65536
SOLVENT DMSO
NS 2614
DS 4
SWH 24038.461 Hz
FIDRES 0.366798 Hz
AQ 1.3631488 sec
RG 205.37
DW 20.800 usec
DE 6.50 usec
TE 300.0 K
D1 2.00000000 sec
D11 0.03000000 sec
TD0 1

======== CHANNEL f1 ========
SFO1 100.6278588 MHz
NUC1 13C
P1 10.00 usec
PLW1 47.00000000 W

======== CHANNEL f2 ========
SFO2 400.1516006 MHz
NUC2 1H
CPDPRG[2 waltz16
PCPD2 90.00 usec
PLW2 18.00000000 W
PLW12 0.34722000 W
PLW13 0.28125000 W

F2 - Processing parameters
SI 32768
SF 100.6177975 MHz
WDW EM
SSB 0
LB 1.00 Hz
GB 0
PC 1.40

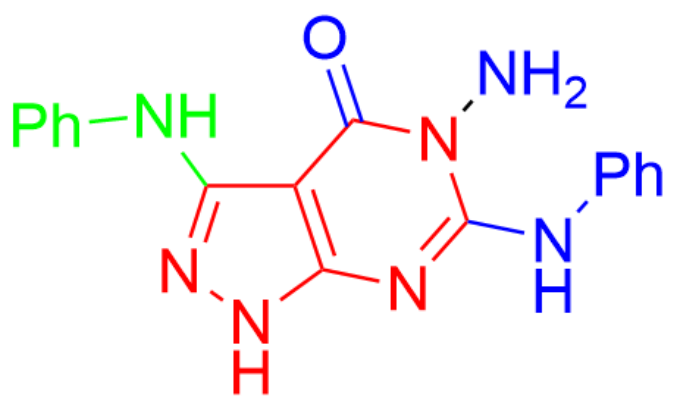

Ph
NH
O
NH2
N
Ph
N
N
N
H
N
H

13

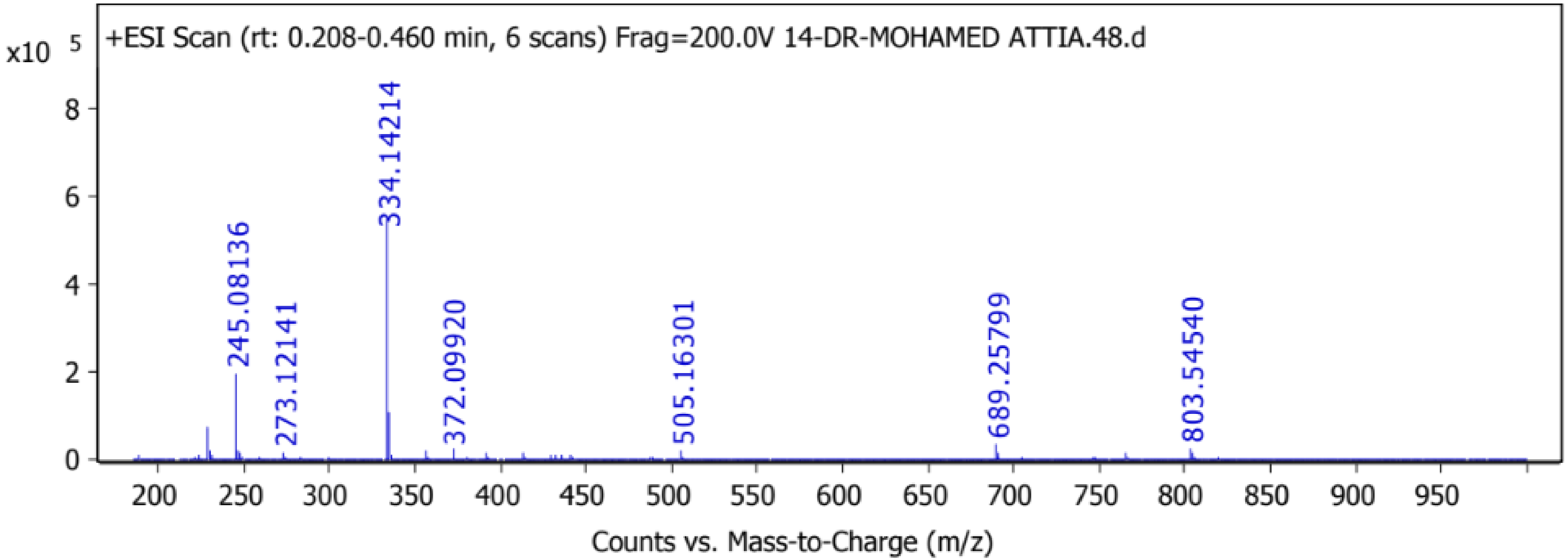

+ESI Scan (rt: 0.208-0.460 min, 6 scans) Frag=200.0V 14-DR-MOHAMED ATTIA.48.d
x10 5
8
6
4
2
0
245.08136
273.12141
334.14214
372.09920
505.16301
689.25799
803.54540
200
250
300
350
400
450
500
550
600
650
700
750
800
850
900
950
Counts vs. Mass-to-Charge (m/z)
