# Supplementary material for: Dual inhibitors of CDK2 and TRKA kinases: design, synthesis, antiproliferative activity and molecular modeling of novel pyrazolopyrimidine derivatives
Source: RSC Adv. 2026 Sep 16;16(44):55449–69. doi: 10.1039/d6ra02521h (PMC13579623; doi:10.1039/d6ra02521h)
Supplement: Supplementary file 2 [file RA-016-D6RA02521H-s002.pdf]

Supplementary Information (SI) for RSC Advances.
This journal is © The Royal Society of Chemistry 2026

Biology

Developmental Therapeutics Program | **NSC:** D-832029 / 1 | **Conc:** 1.00E-5 Molar | **Test Date:** Aug 09, 2021

One Dose Mean Graph | **Experiment ID:** 2108OS72 | **Report Date:** Sep 15, 2021

Panel/Cell Line	Growth Percent
Leukemia	
CCRF-CEM	83.46
HL-60(TB)	91.07
K-562	56.95
MOLT-4	80.21
RPMI-8226	71.53
SR	64.28
Non-Small Cell Lung Cancer	
A549/ATCC	100.88
EKVX	94.94
HOP-62	104.11
HOP-92	104.81
NCI-H226	105.18
NCI-H23	95.22
NCI-H322M	97.41
NCI-H460	102.10
NCI-H522	89.89
Colon Cancer	
COLO 205	93.51
HCC-2998	105.59
HCT-116	92.27
HCT-15	77.73
HT29	53.93
KM12	70.90
SW-620	88.00
CNS Cancer	
SF-268	108.91
SF-295	90.80
SF-539	85.92
SNB-19	114.15
SNB-75	87.03
U251	90.76
Melanoma	
LOX IMVI	89.54
MALME-3M	114.19
M14	82.51
MDA-MB-435	90.29
SK-MEL-2	93.40
SK-MEL-28	94.47
SK-MEL-5	98.54
UACC-257	109.15
UACC-62	87.87
Ovarian Cancer	
IGROV1	94.81
OVCAR-3	123.04
OVCAR-4	90.33
OVCAR-5	95.71
OVCAR-8	96.58
NCI/ADR-RES	89.18
SK-OV-3	110.92
Renal Cancer	
786-0	96.58
A498	77.73
ACHN	98.32
CAKI-1	94.16
RXF 393	85.21
SN12C	99.65
UO-31	93.50
Prostate Cancer	
PC-3	95.91
DU-145	99.03
Breast Cancer	
MCF7	86.96
MDA-MB-231/ATCC	99.70
HS 578T	94.46
BT-549	130.33
T-47D	82.31
MDA-MB-468	102.18
Mean	93.19
Delta	39.26
Range	76.40

Mean Growth Percent - Growth Percent

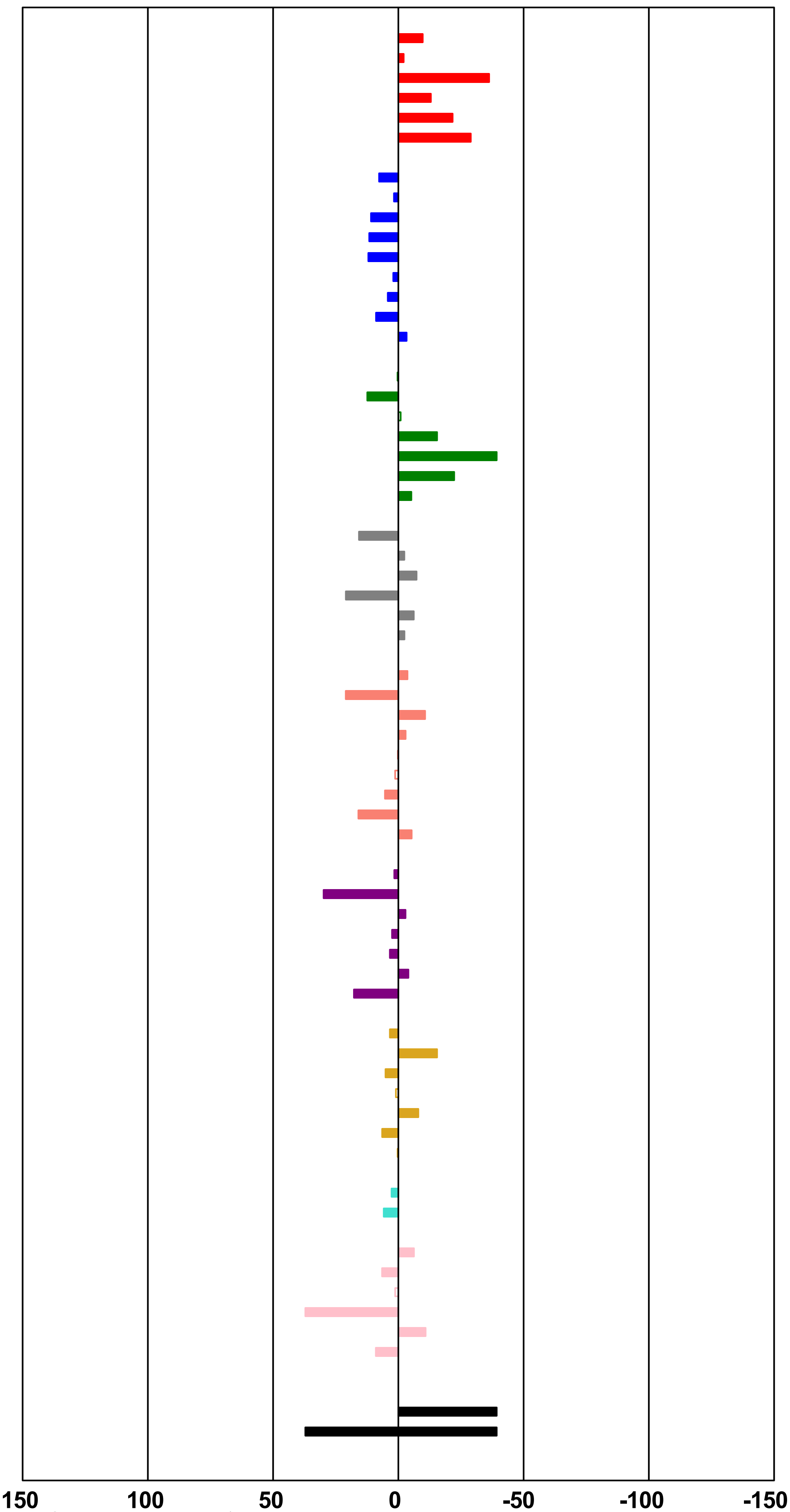

6a

Figure 1. Mean graph of compound **6a**

Developmental Therapeutics Program	NSC: D-832030 / 1	Conc: 1.00E-5 Molar	Test Date: Aug 09, 2021
One Dose Mean Graph	Experiment ID: 2108OS72		Report Date: Sep 15, 2021

Panel/Cell Line	Growth Percent
Leukemia	
CCRF-CEM	83.09
HL-60(TB)	92.90
K-562	49.39
MOLT-4	82.02
RPMI-8226	62.86
SR	68.85
Non-Small Cell Lung Cancer	
A549/ATCC	103.40
EKVX	94.85
HOP-62	101.37
HOP-92	116.82
NCI-H226	111.20
NCI-H23	92.65
NCI-H322M	96.30
NCI-H460	101.49
NCI-H522	85.93
Colon Cancer	
COLO 205	90.62
HCC-2998	111.20
HCT-116	83.71
HCT-15	74.43
HT29	52.69
KM12	80.15
SW-620	91.90
CNS Cancer	
SF-268	111.94
SF-295	86.46
SF-539	92.04
SNB-19	112.68
SNB-75	101.07
U251	101.50
Melanoma	
LOX IMVI	92.93
MALME-3M	111.13
M14	86.27
MDA-MB-435	90.46
SK-MEL-2	105.07
SK-MEL-28	94.10
SK-MEL-5	99.09
UACC-257	114.76
UACC-62	86.48
Ovarian Cancer	
IGROV1	86.78
OVCAR-3	125.18
OVCAR-4	87.35
OVCAR-5	93.02
OVCAR-8	98.16
NCI/ADR-RES	92.83
SK-OV-3	109.13
Renal Cancer	
786-0	97.72
A498	84.90
ACHN	99.99
CAKI-1	95.81
RXF 393	94.76
SN12C	99.55
UO-31	95.28
Prostate Cancer	
PC-3	97.66
DU-145	102.09
Breast Cancer	
MCF7	88.85
MDA-MB-231/ATCC	95.28
HS 578T	97.84
BT-549	119.67
T-47D	86.73
MDA-MB-468	104.82
Mean	94.36
Delta	44.97
Range	75.79

Mean Growth Percent - Growth Percent

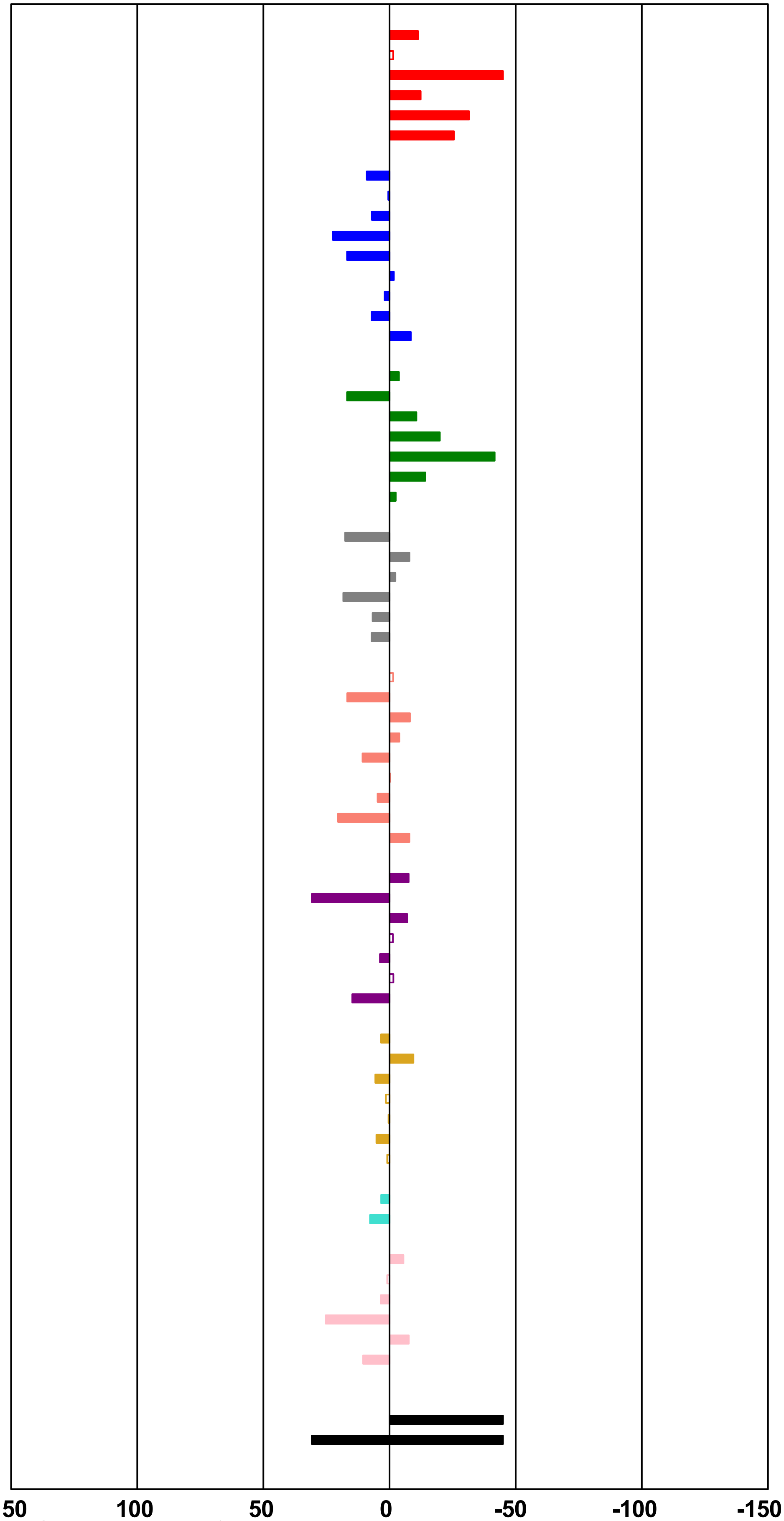

6b

Figure 2. Mean graph of compound **6b**

Developmental Therapeutics Program

One Dose Mean Graph

**NSC:** D-832031 / 1	**Conc:** 1.00E-5 Molar	**Test Date:** Aug 09, 2021
**Experiment ID:** 2108OS72		**Report Date:** Sep 15, 2021

Panel/Cell Line	Growth Percent
Leukemia	
CCRF-CEM	68.84
HL-60(TB)	87.71
K-562	37.75
MOLT-4	73.46
RPMI-8226	42.81
SR	50.73
Non-Small Cell Lung Cancer	
A549/ATCC	92.32
EKVX	96.52
HOP-62	92.30
HOP-92	100.22
NCI-H226	105.09
NCI-H23	95.74
NCI-H322M	96.83
NCI-H460	95.43
NCI-H522	77.37
Colon Cancer	
COLO 205	96.32
HCC-2998	107.80
HCT-116	75.84
HCT-15	63.55
HT29	55.55
KM12	63.37
SW-620	93.83
CNS Cancer	
SF-268	105.60
SF-295	72.97
SF-539	78.23
SNB-19	112.20
SNB-75	90.70
U251	87.05
Melanoma	
LOX IMVI	94.77
MALME-3M	112.68
M14	77.48
MDA-MB-435	86.29
SK-MEL-2	96.10
SK-MEL-28	89.74
SK-MEL-5	96.94
UACC-257	107.90
UACC-62	90.68
Ovarian Cancer	
IGROV1	84.64
OVCAR-3	131.05
OVCAR-4	79.19
OVCAR-5	92.83
OVCAR-8	86.41
NCI/ADR-RES	82.85
SK-OV-3	109.34
Renal Cancer	
786-0	69.70
A498	76.69
ACHN	98.21
CAKI-1	91.99
RXF 393	79.97
SN12C	96.81
UO-31	94.18
Prostate Cancer	
PC-3	82.34
DU-145	99.96
Breast Cancer	
MCF7	84.96
MDA-MB-231/ATCC	94.02
HS 578T	92.50
BT-549	128.71
T-47D	77.60
MDA-MB-468	102.60
Mean	88.22
Delta	50.47
Range	93.30

Mean Growth Percent - Growth Percent

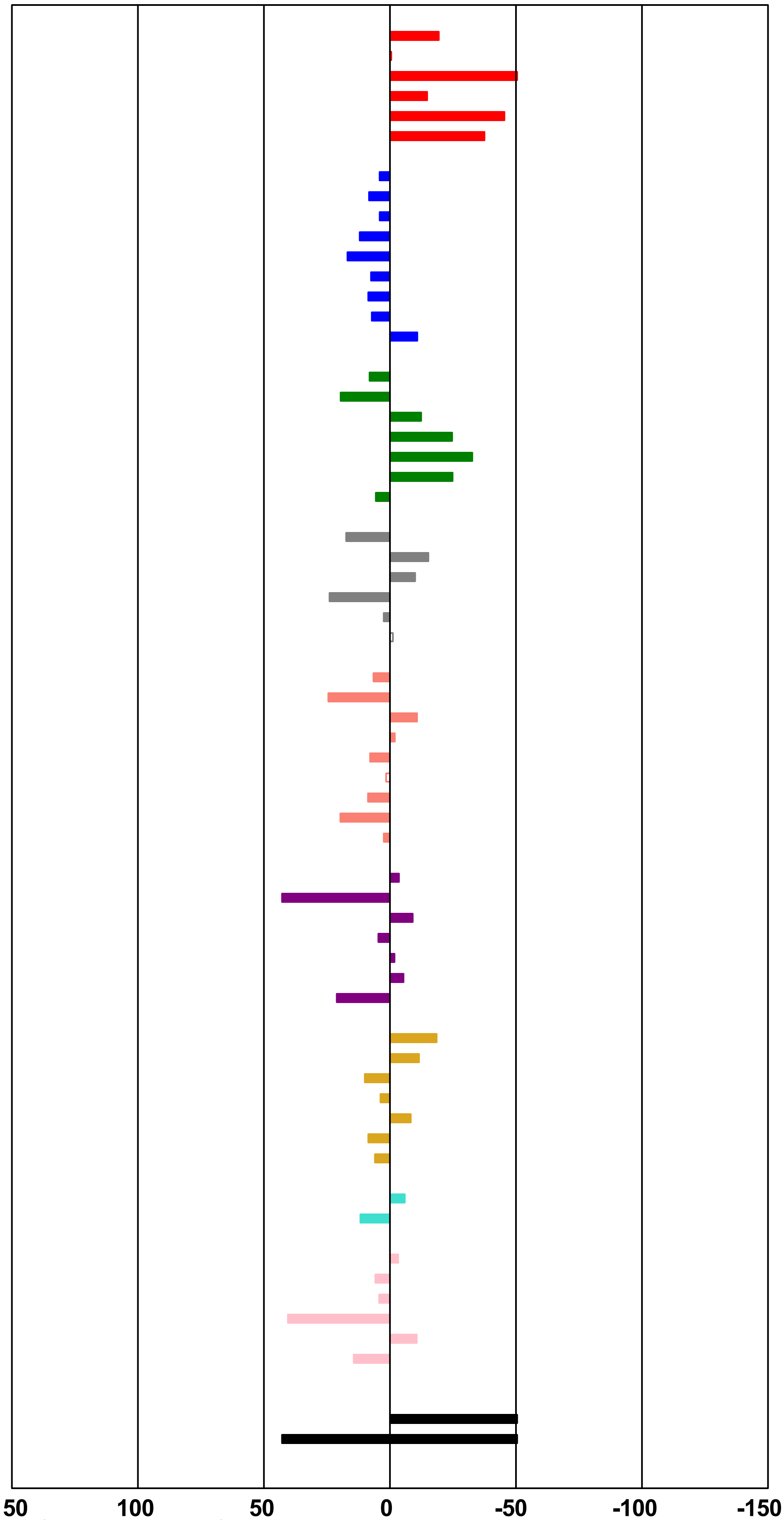

6c

Figure 3. Mean graph of compound **6c**

Developmental Therapeutics Program

One Dose Mean Graph

NSC: D-832032 / 1	Conc: 1.00E-5 Molar	Test Date: Aug 09, 2021
Experiment ID: 2108OS72		Report Date: Sep 15, 2021

Panel/Cell Line	Growth Percent
**Leukemia**	
CCRF-CEM	74.48
HL-60(TB)	92.27
K-562	67.04
MOLT-4	75.34
RPMI-8226	67.58
SR	60.29
**Non-Small Cell Lung Cancer**	
A549/ATCC	98.78
EKVX	99.72
HOP-62	105.00
HOP-92	85.20
NCI-H226	96.96
NCI-H23	94.59
NCI-H322M	92.64
NCI-H460	103.52
NCI-H522	87.21
**Colon Cancer**	
COLO 205	106.16
HCC-2998	104.95
HCT-116	86.17
HCT-15	70.42
HT29	70.66
KM12	72.09
SW-620	94.99
**CNS Cancer**	
SF-268	103.40
SF-295	95.29
SF-539	81.00
SNB-19	110.40
SNB-75	88.94
U251	101.47
**Melanoma**	
LOX IMVI	83.45
MALME-3M	111.32
M14	95.80
MDA-MB-435	95.12
SK-MEL-2	104.50
SK-MEL-28	102.27
SK-MEL-5	91.83
UACC-257	112.88
UACC-62	100.43
**Ovarian Cancer**	
IGROV1	84.72
OVCAR-3	123.54
OVCAR-4	92.42
OVCAR-5	89.40
OVCAR-8	88.09
NCI/ADR-RES	88.82
SK-OV-3	109.44
**Renal Cancer**	
786-0	102.57
A498	91.72
ACHN	94.11
CAKI-1	92.85
RXF 393	89.75
SN12C	97.22
UO-31	88.53
**Prostate Cancer**	
PC-3	87.66
DU-145	98.06
**Breast Cancer**	
MCF7	92.70
MDA-MB-231/ATCC	79.33
HS 578T	90.60
BT-549	106.28
T-47D	84.96
MDA-MB-468	96.18
Mean	92.43
Delta	32.14
Range	63.25

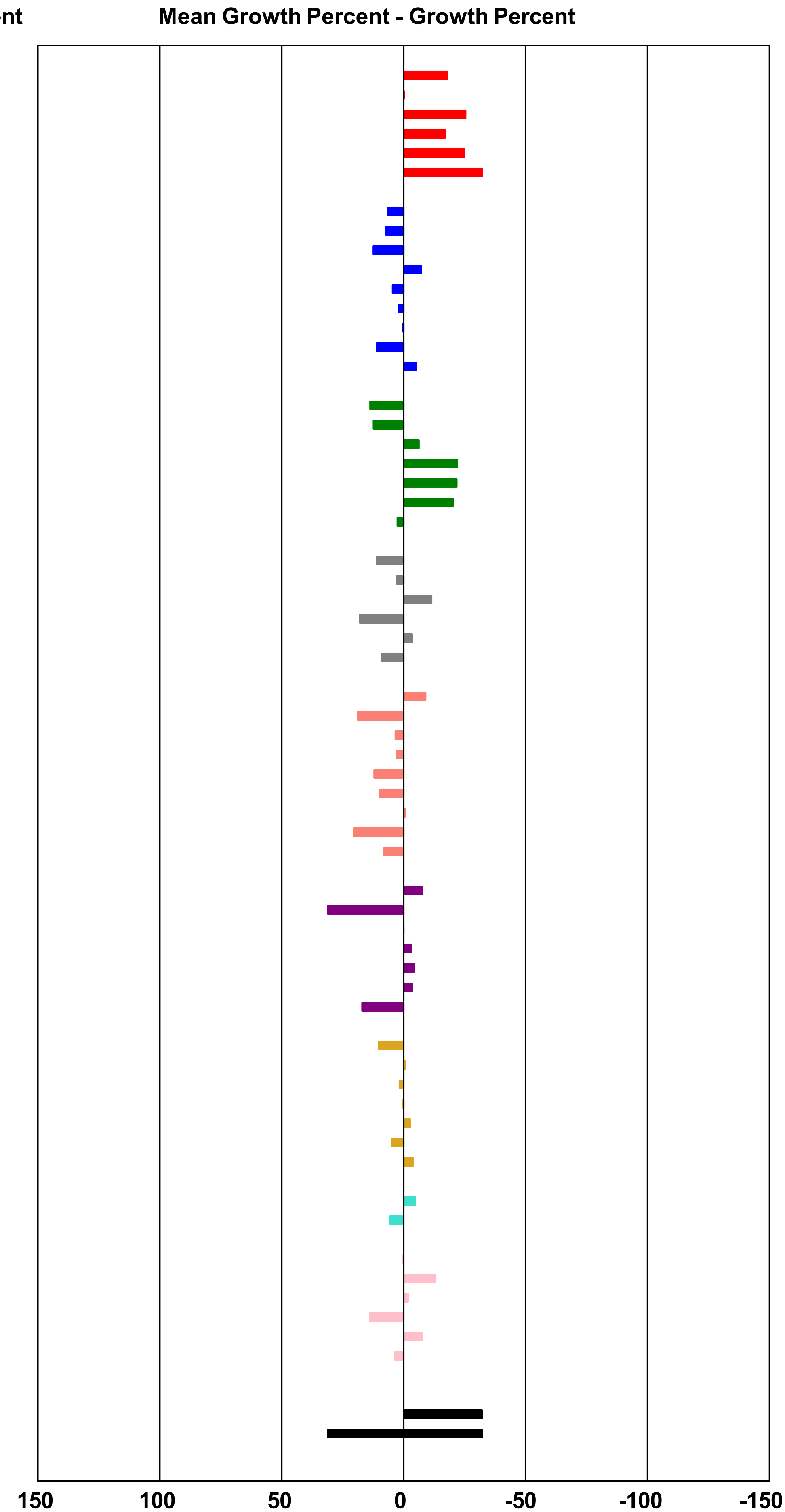

6d

Figure 4. Mean graph of compound **6d**

**Developmental Therapeutics Program**<br>**One Dose Mean Graph**	**NSC:** D-832033 / 1	**Conc:** 1.00E-5 Molar	**Test Date:** Aug 09, 2021
	**Experiment ID:** 2108OS72		**Report Date:** Sep 15, 2021

Panel/Cell Line	Growth Percent
Leukemia	
CCRF-CEM	99.79
HL-60(TB)	105.64
K-562	63.63
MOLT-4	91.20
RPMI-8226	90.34
SR	74.21
Non-Small Cell Lung Cancer	
A549/ATCC	106.53
EKVX	103.71
HOP-62	106.87
HOP-92	114.97
NCI-H226	118.42
NCI-H23	97.69
NCI-H322M	107.33
NCI-H460	106.70
NCI-H522	95.98
Colon Cancer	
COLO 205	109.55
HCC-2998	110.88
HCT-116	80.35
HCT-15	80.56
HT29	72.93
KM12	82.60
SW-620	98.17
CNS Cancer	
SF-268	111.86
SF-295	100.19
SF-539	96.99
SNB-19	113.86
SNB-75	101.51
U251	104.46
Melanoma	
LOX IMVI	102.90
MALME-3M	123.22
M14	93.41
MDA-MB-435	96.34
SK-MEL-2	118.24
SK-MEL-28	96.88
SK-MEL-5	101.35
UACC-257	119.35
UACC-62	94.29
Ovarian Cancer	
IGROV1	87.14
OVCAR-3	131.83
OVCAR-4	97.16
OVCAR-5	98.18
OVCAR-8	105.72
NCI/ADR-RES	101.57
SK-OV-3	121.49
Renal Cancer	
786-0	100.05
A498	89.49
ACHN	104.49
CAKI-1	99.97
RXF 393	101.69
SN12C	101.58
UO-31	93.99
Prostate Cancer	
PC-3	106.85
DU-145	109.90
Breast Cancer	
MCF7	91.49
MDA-MB-231/ATCC	106.40
HS 578T	100.93
BT-549	102.93
T-47D	95.27
MDA-MB-468	113.93
Mean	100.93
Delta	37.30
Range	68.20

Mean Growth Percent - Growth Percent

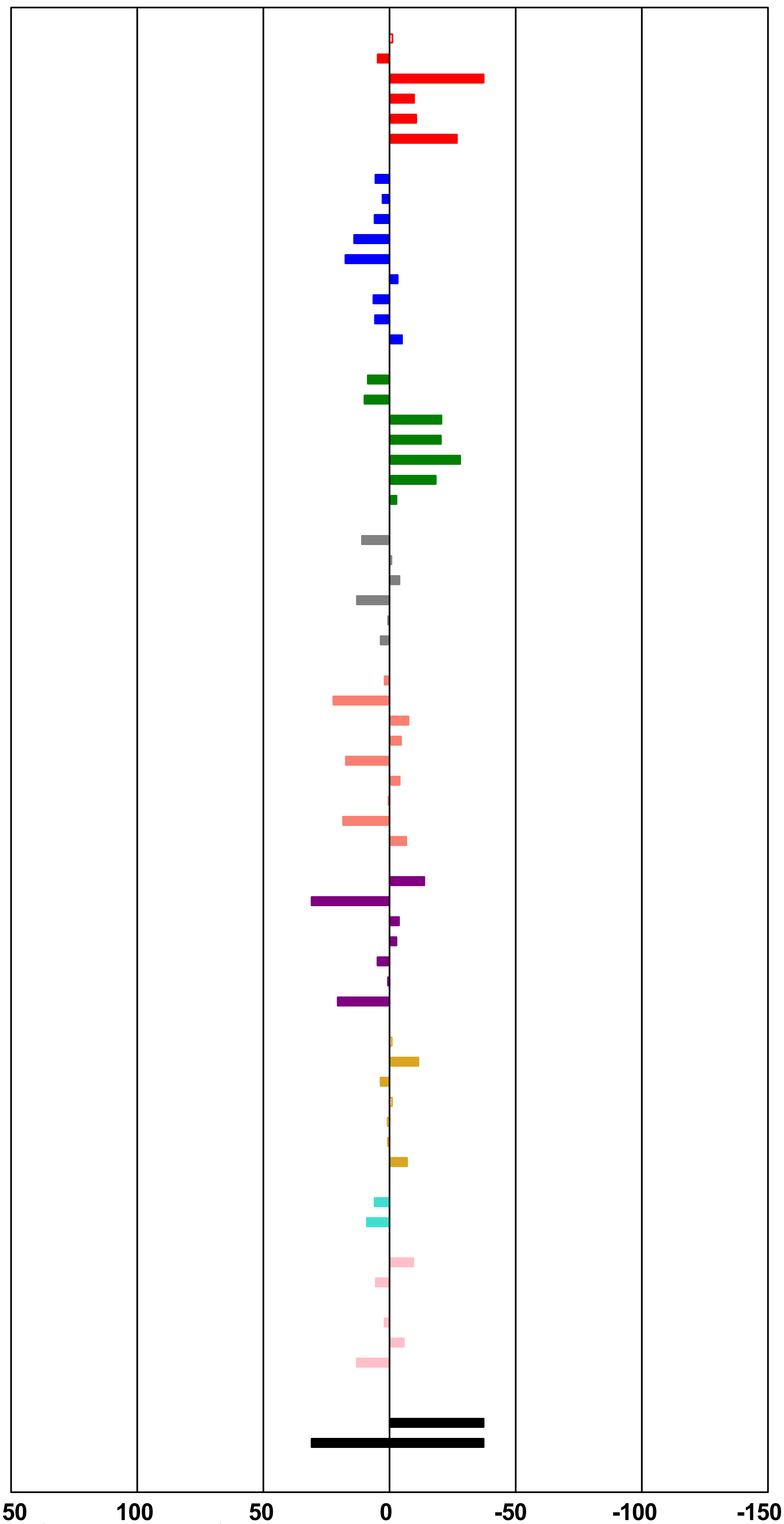

6e

Figure 5. Mean graph of compound **6e**

Developmental Therapeutics Program One Dose Mean Graph	NSC: D-832034 / 1	Conc: 1.00E-5 Molar	Test Date: Aug 09, 2021
	Experiment ID: 2108OS72		Report Date: Sep 15, 2021

Panel/Cell Line	Growth Percent
Leukemia	
CCRF-CEM	91.57
HL-60(TB)	106.62
K-562	58.58
MOLT-4	95.14
RPMI-8226	84.98
SR	77.19
Non-Small Cell Lung Cancer	
A549/ATCC	107.80
EKVX	100.34
HOP-62	114.91
HOP-92	116.27
NCI-H226	112.50
NCI-H23	101.07
NCI-H322M	109.15
NCI-H460	104.97
NCI-H522	93.88
Colon Cancer	
COLO 205	110.46
HCC-2998	110.78
HCT-116	80.07
HCT-15	78.32
HT29	66.43
KM12	81.08
SW-620	95.67
CNS Cancer	
SF-268	111.98
SF-295	97.81
SF-539	91.53
SNB-19	113.63
SNB-75	109.14
U251	97.96
Melanoma	
LOX IMVI	99.25
MALME-3M	115.82
M14	97.05
MDA-MB-435	94.30
SK-MEL-2	103.23
SK-MEL-28	99.02
SK-MEL-5	100.32
UACC-257	113.40
UACC-62	94.74
Ovarian Cancer	
IGROV1	85.13
OVCAR-3	134.60
OVCAR-4	97.13
OVCAR-5	98.40
OVCAR-8	104.42
NCI/ADR-RES	101.94
SK-OV-3	126.03
Renal Cancer	
786-0	98.60
A498	91.33
ACHN	105.10
CAKI-1	101.56
RXF 393	92.82
SN12C	100.60
UO-31	96.30
Prostate Cancer	
PC-3	118.03
DU-145	107.84
Breast Cancer	
MCF7	90.76
MDA-MB-231/ATCC	103.20
HS 578T	101.96
BT-549	114.89
T-47D	93.47
MDA-MB-468	112.65
Mean	100.23
Delta	41.65
Range	76.02

Mean Growth Percent - Growth Percent

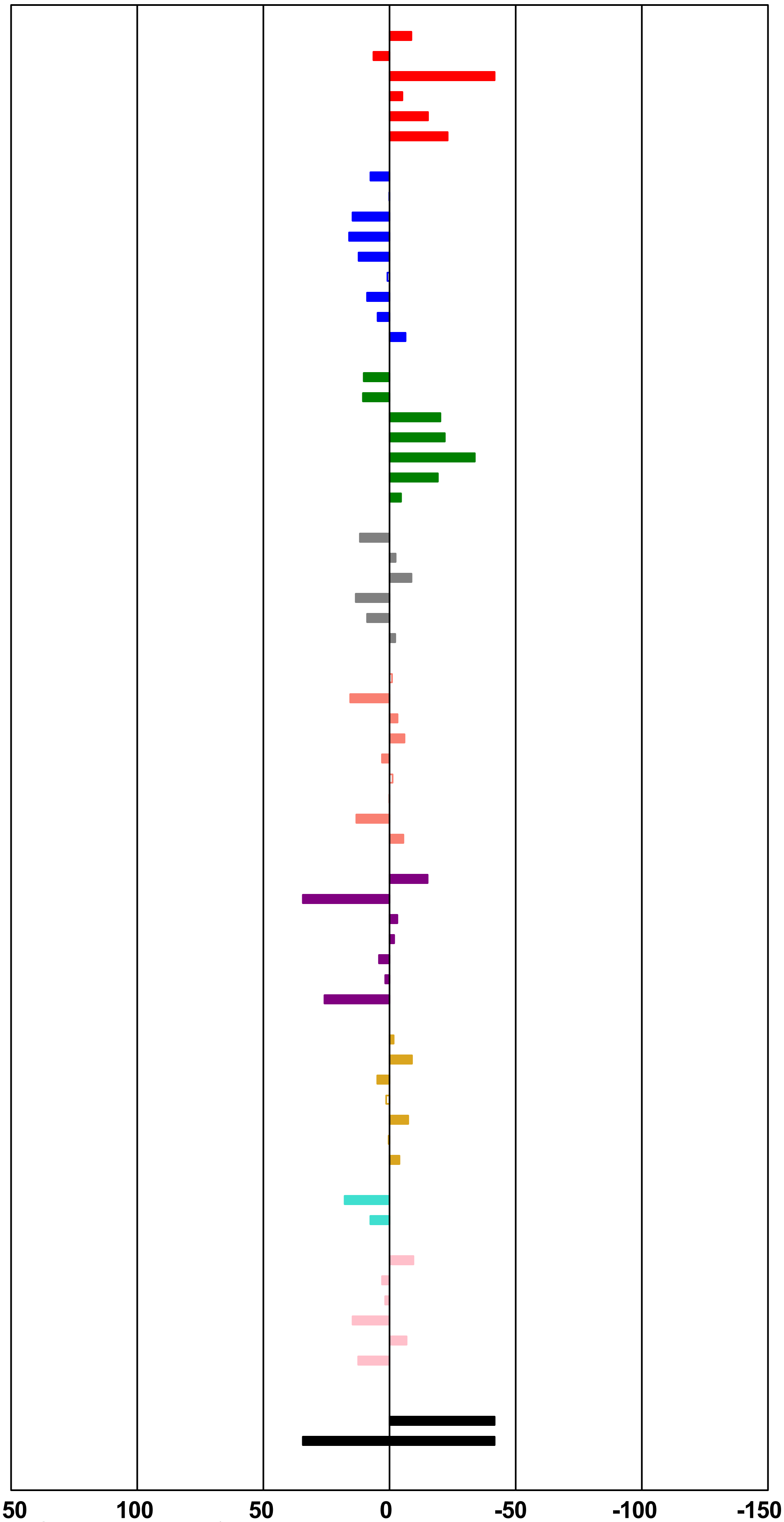

6f

Figure 6. Mean graph of compound **6f**

Developmental Therapeutics Program One Dose Mean Graph	NSC: D-832035 / 1	Conc: 1.00E-5 Molar	Test Date: Aug 09, 2021
	Experiment ID: 2108OS72		Report Date: Sep 15, 2021

Panel/Cell Line	Growth Percent
Leukemia	
CCRF-CEM	90.07
HL-60(TB)	117.40
MOLT-4	86.44
RPMI-8226	102.09
Non-Small Cell Lung Cancer	
A549/ATCC	94.42
EKVX	92.10
HOP-62	105.05
HOP-92	106.66
NCI-H226	106.82
NCI-H23	98.59
NCI-H322M	108.94
NCI-H460	107.94
NCI-H522	94.12
Colon Cancer	
COLO 205	106.66
HCC-2998	118.24
HCT-116	77.56
HCT-15	79.37
HT29	78.26
KM12	92.87
SW-620	101.64
CNS Cancer	
SF-268	103.04
SF-295	104.25
SF-539	106.00
SNB-19	102.41
SNB-75	99.54
U251	105.49
Melanoma	
LOX IMVI	108.66
MALME-3M	120.85
M14	99.29
MDA-MB-435	101.15
SK-MEL-2	100.72
SK-MEL-28	106.04
SK-MEL-5	99.12
UACC-257	114.84
UACC-62	91.11
Ovarian Cancer	
IGROV1	92.37
OVCAR-3	131.66
OVCAR-4	100.82
OVCAR-5	114.65
OVCAR-8	100.51
NCI/ADR-RES	99.15
SK-OV-3	123.44
Renal Cancer	
786-0	90.86
A498	81.80
ACHN	99.73
CAKI-1	92.59
RXF 393	86.74
SN12C	105.71
UO-31	79.16
Prostate Cancer	
PC-3	99.71
DU-145	105.57
Breast Cancer	
MCF7	90.53
MDA-MB-231/ATCC	101.45
HS 578T	102.70
BT-549	116.77
T-47D	93.81
MDA-MB-468	101.31
Mean	100.68
Delta	23.12
Range	54.10

Mean Growth Percent - Growth Percent

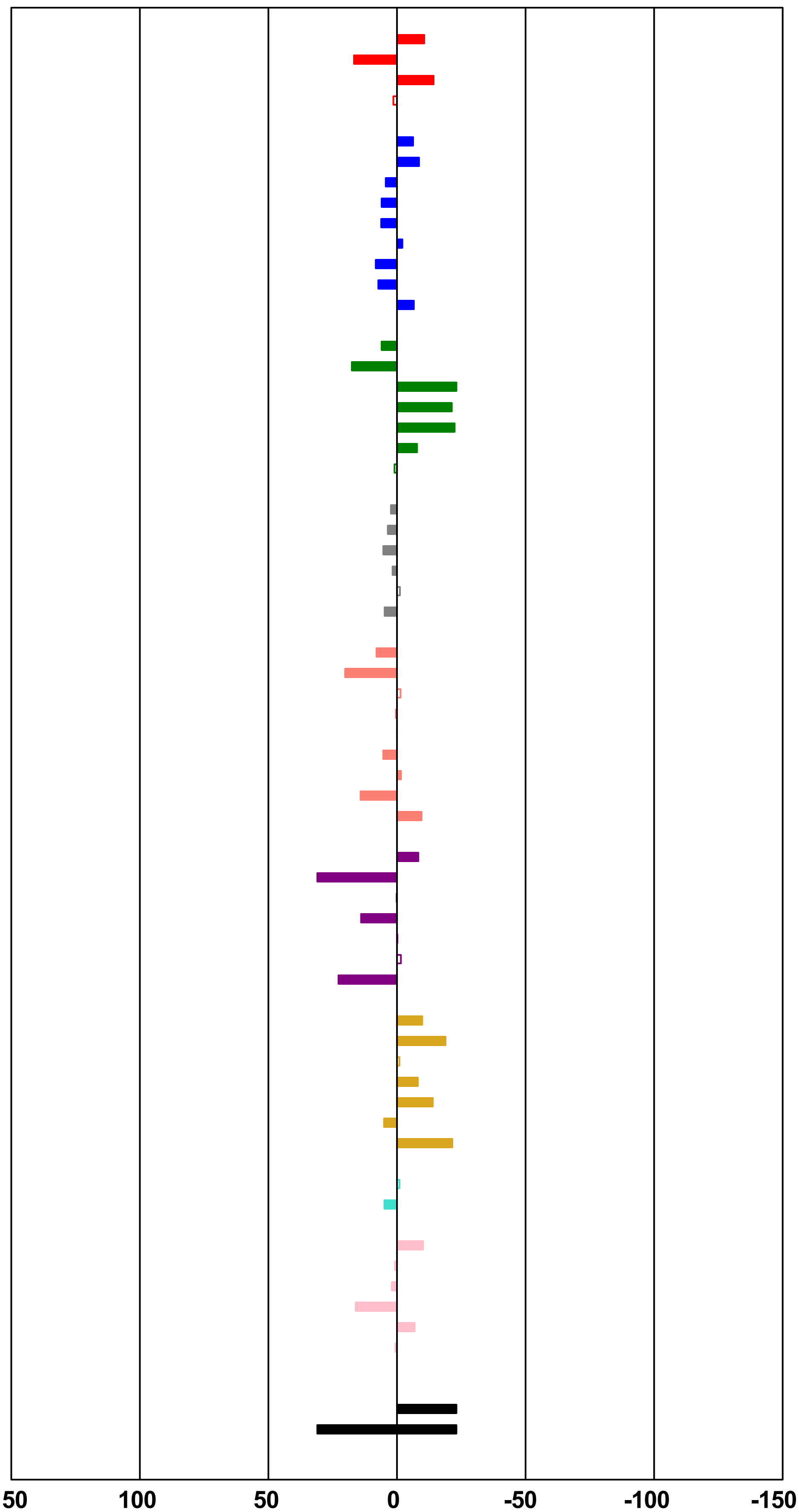

6g

Figure 7. Mean graph of compound **6g**

Developmental Therapeutics Program One Dose Mean Graph	NSC: D-832036 / 1	Conc: 1.00E-5 Molar	Test Date: Aug 09, 2021
	Experiment ID: 2108OS72		Report Date: Sep 15, 2021

Panel/Cell Line	Growth Percent
**Leukemia**	
CCRF-CEM	95.50
HL-60(TB)	101.07
K-562	80.07
MOLT-4	98.33
RPMI-8226	103.05
SR	86.60
**Non-Small Cell Lung Cancer**	
A549/ATCC	115.92
EKVX	104.97
HOP-62	113.64
HOP-92	117.60
NCI-H226	110.10
NCI-H23	96.34
NCI-H322M	111.73
NCI-H460	110.20
NCI-H522	110.80
**Colon Cancer**	
COLO 205	113.23
HCC-2998	112.95
HCT-116	99.26
HCT-15	91.13
HT29	84.50
KM12	89.65
SW-620	97.82
**CNS Cancer**	
SF-268	114.54
SF-295	102.35
SF-539	99.31
SNB-19	114.16
SNB-75	109.11
U251	107.57
**Melanoma**	
LOX IMVI	111.35
MALME-3M	112.20
M14	95.33
MDA-MB-435	101.43
SK-MEL-2	128.04
SK-MEL-28	98.96
SK-MEL-5	100.97
UACC-257	116.65
UACC-62	98.49
**Ovarian Cancer**	
IGROV1	104.47
OVCAR-3	128.84
OVCAR-4	100.49
OVCAR-5	101.79
OVCAR-8	102.76
NCI/ADR-RES	101.52
SK-OV-3	121.32
**Renal Cancer**	
786-0	104.43
A498	101.04
ACHN	107.79
CAKI-1	100.99
RXF 393	110.12
SN12C	105.50
UO-31	100.21
**Prostate Cancer**	
PC-3	101.15
DU-145	110.53
**Breast Cancer**	
MCF7	94.12
MDA-MB-231/ATCC	102.79
HS 578T	110.24
BT-549	127.25
T-47D	96.26
MDA-MB-468	111.34
Mean	105.08
Delta	25.01
Range	48.77

Mean Growth Percent - Growth Percent

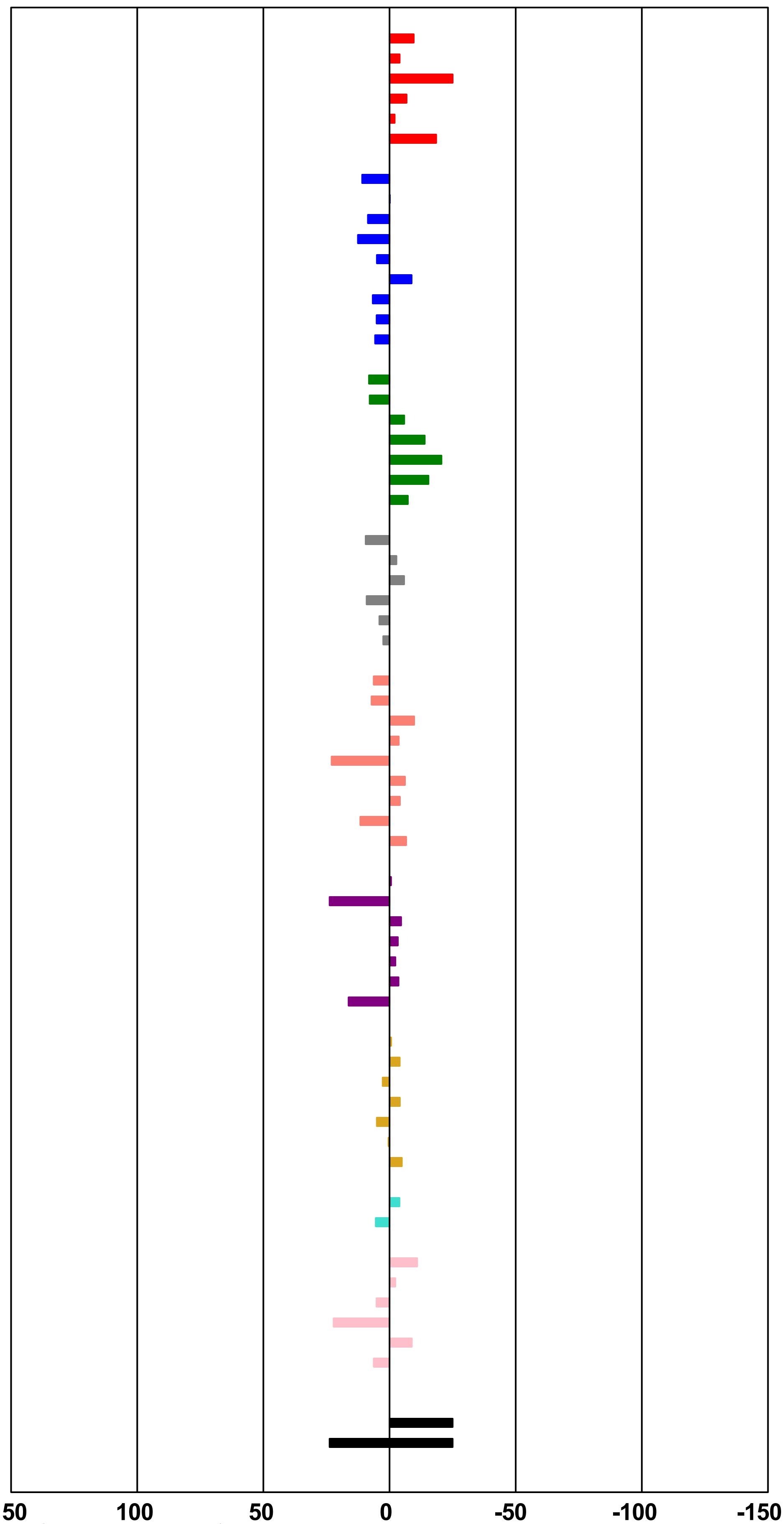

6h

Figure 8. Mean graph of compound **6h**

Developmental Therapeutics Program
One Dose Mean Graph

**NSC:** D-832037 / 1	**Conc:** 1.00E-5 Molar
**Test Date:** Aug 09, 2021	**Experiment ID:** 2108OS72
**Report Date:** Sep 15, 2021	

Panel/Cell Line	Growth Percent
Leukemia	
CCRF-CEM	91.41
HL-60(TB)	101.46
K-562	95.50
MOLT-4	92.09
RPMI-8226	94.27
SR	87.85
Non-Small Cell Lung Cancer	
A549/ATCC	106.26
EKVX	90.42
HOP-62	96.94
HOP-92	101.72
NCI-H226	97.07
NCI-H23	90.33
NCI-H322M	104.02
NCI-H460	104.48
NCI-H522	94.14
Colon Cancer	
COLO 205	114.64
HCC-2998	112.98
HCT-116	83.61
HCT-15	80.66
HT29	79.38
KM12	85.90
SW-620	93.43
CNS Cancer	
SF-268	105.73
SF-295	98.54
SF-539	94.84
SNB-19	107.30
SNB-75	92.66
U251	96.95
Melanoma	
LOX IMVI	107.28
MALME-3M	117.44
M14	97.87
MDA-MB-435	102.71
SK-MEL-2	107.29
SK-MEL-28	101.71
SK-MEL-5	98.76
UACC-257	112.76
UACC-62	90.33
Ovarian Cancer	
IGROV1	76.14
OVCAR-3	128.62
OVCAR-4	91.18
OVCAR-5	103.53
OVCAR-8	98.04
NCI/ADR-RES	97.22
SK-OV-3	108.16
Renal Cancer	
786-0	105.44
A498	82.94
ACHN	100.06
CAKI-1	105.01
RXF 393	96.43
SN12C	101.49
UO-31	94.19
Prostate Cancer	
PC-3	100.67
DU-145	109.35
Breast Cancer	
MCF7	31.26
MDA-MB-231/ATCC	97.17
HS 578T	94.95
BT-549	123.06
T-47D	52.23
MDA-MB-468	62.50
Mean	96.45
Delta	65.19
Range	97.36

Mean Growth Percent - Growth Percent

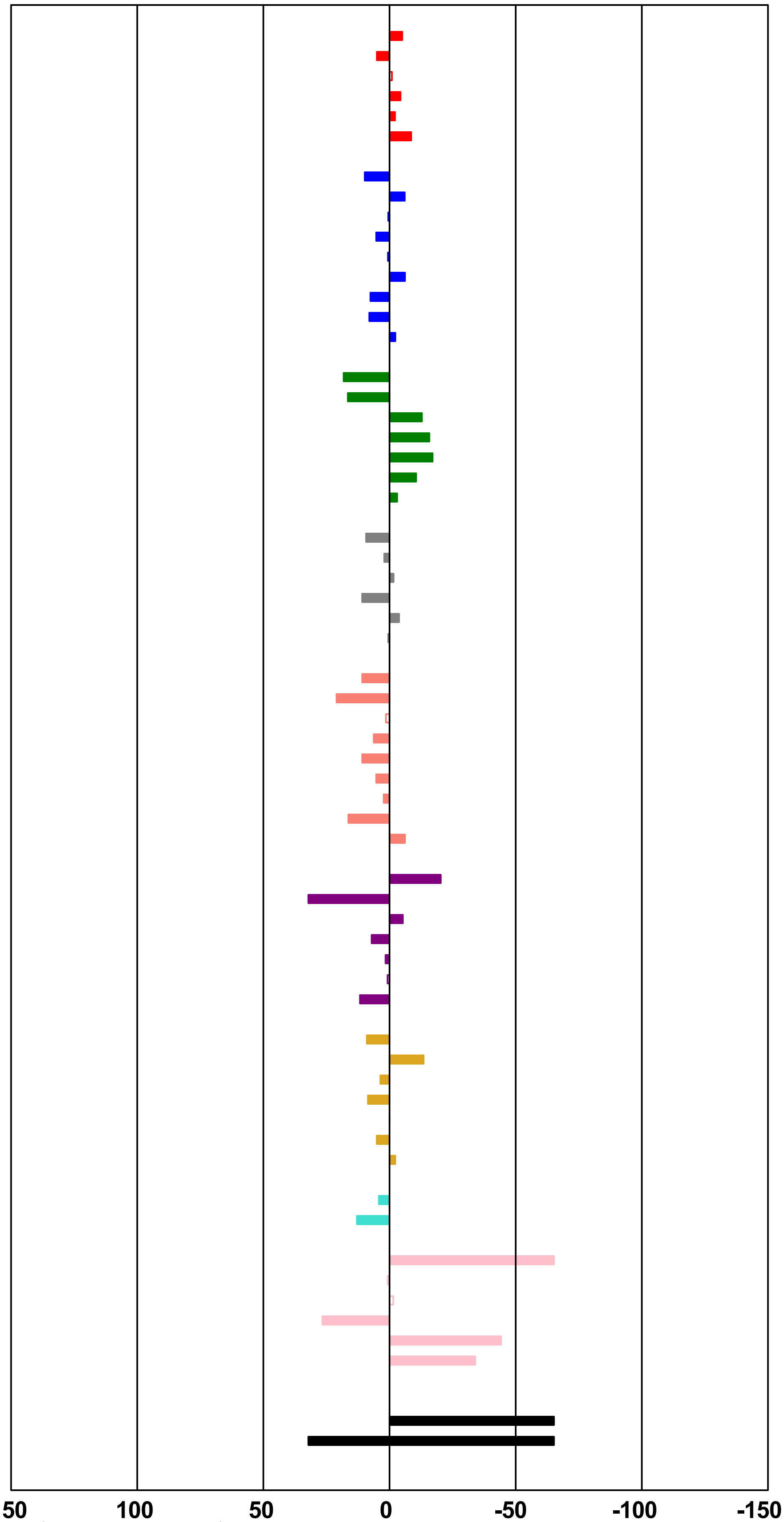

6i

Figure 9. Mean graph of compound **6i**

Developmental Therapeutics Program

One Dose Mean Graph

**NSC:** D-832038 / 1	**Conc:** 1.00E-5 Molar
**Test Date:** Aug 09, 2021	**Experiment ID:** 2108OS72
**Report Date:** Sep 15, 2021	

Panel/Cell Line	Growth Percent
Leukemia	
CCRF-CEM	89.28
HL-60(TB)	100.15
K-562	75.77
MOLT-4	93.23
RPMI-8226	87.95
SR	78.11
Non-Small Cell Lung Cancer	
A549/ATCC	104.55
EKVX	82.23
HOP-62	94.76
HOP-92	105.74
NCI-H226	88.53
NCI-H23	86.16
NCI-H322M	100.39
NCI-H460	101.80
NCI-H522	94.67
Colon Cancer	
COLO 205	105.56
HCC-2998	112.61
HCT-116	87.22
HCT-15	79.02
HT29	68.33
KM12	80.89
SW-620	89.26
CNS Cancer	
SF-268	114.63
SF-295	88.79
SF-539	84.39
SNB-19	113.68
SNB-75	86.89
U251	98.05
Melanoma	
LOX IMVI	90.84
MALME-3M	112.71
M14	83.64
MDA-MB-435	98.74
SK-MEL-2	113.09
SK-MEL-28	97.31
SK-MEL-5	96.74
UACC-257	122.30
UACC-62	86.30
Ovarian Cancer	
IGROV1	67.93
OVCAR-3	127.51
OVCAR-4	90.75
OVCAR-5	102.16
OVCAR-8	98.46
NCI/ADR-RES	94.91
SK-OV-3	98.55
Renal Cancer	
786-0	102.31
A498	103.84
ACHN	90.06
CAKI-1	86.42
RXF 393	102.06
SN12C	107.37
UO-31	92.27
Prostate Cancer	
PC-3	91.16
DU-145	105.56
Breast Cancer	
MCF7	55.88
MDA-MB-231/ATCC	82.66
HS 578T	96.81
BT-549	107.44
T-47D	40.20
MDA-MB-468	77.51
Mean	93.49
Delta	53.29
Range	87.31

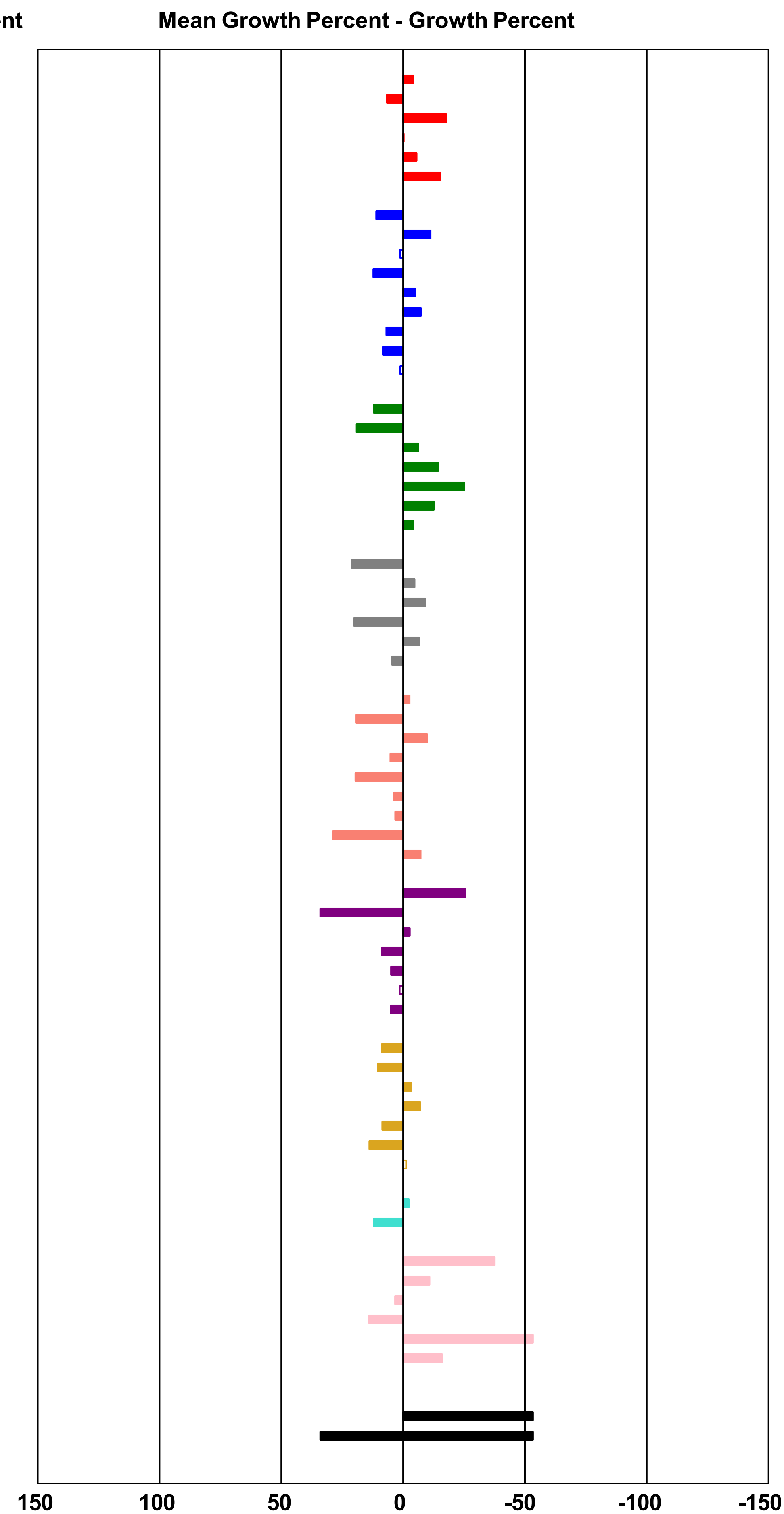

6j

Figure 10. Mean graph of compound **6j**

Developmental Therapeutics Program<br>One Dose Mean Graph	**NSC:** D-832039 / 1	**Conc:** 1.00E-5 Molar	**Test Date:** Aug 09, 2021
	**Experiment ID:** 2108OS72		**Report Date:** Sep 15, 2021

Panel/Cell Line	Growth Percent
Leukemia	
CCRF-CEM	98.35
HL-60(TB)	103.33
K-562	101.77
MOLT-4	100.75
RPMI-8226	110.62
SR	91.57
Non-Small Cell Lung Cancer	
A549/ATCC	102.01
EKVX	94.12
HOP-62	90.20
HOP-92	85.14
NCI-H226	111.94
NCI-H23	109.22
NCI-H322M	111.90
NCI-H460	106.91
NCI-H522	95.57
Colon Cancer	
COLO 205	108.04
HCC-2998	117.51
HCT-116	107.87
HCT-15	98.24
HT29	103.75
KM12	88.17
SW-620	104.75
CNS Cancer	
SF-268	102.01
SF-295	101.16
SF-539	101.28
SNB-19	100.12
SNB-75	101.74
U251	103.97
Melanoma	
LOX IMVI	112.81
MALME-3M	109.90
M14	111.52
MDA-MB-435	100.35
SK-MEL-2	106.08
SK-MEL-28	101.49
SK-MEL-5	100.40
UACC-257	113.64
UACC-62	101.27
Ovarian Cancer	
IGROV1	109.52
OVCAR-3	114.32
OVCAR-4	98.17
OVCAR-5	100.88
OVCAR-8	104.07
NCI/ADR-RES	104.66
SK-OV-3	112.22
Renal Cancer	
786-0	82.79
A498	63.61
ACHN	103.29
CAKI-1	89.73
RXF 393	108.60
SN12C	103.40
UO-31	96.02
Prostate Cancer	
PC-3	100.80
DU-145	104.36
Breast Cancer	
MCF7	93.74
MDA-MB-231/ATCC	111.07
HS 578T	101.76
BT-549	93.94
T-47D	96.16
MDA-MB-468	95.07
Mean	101.66
Delta	38.05
Range	53.90

Mean Growth Percent - Growth Percent

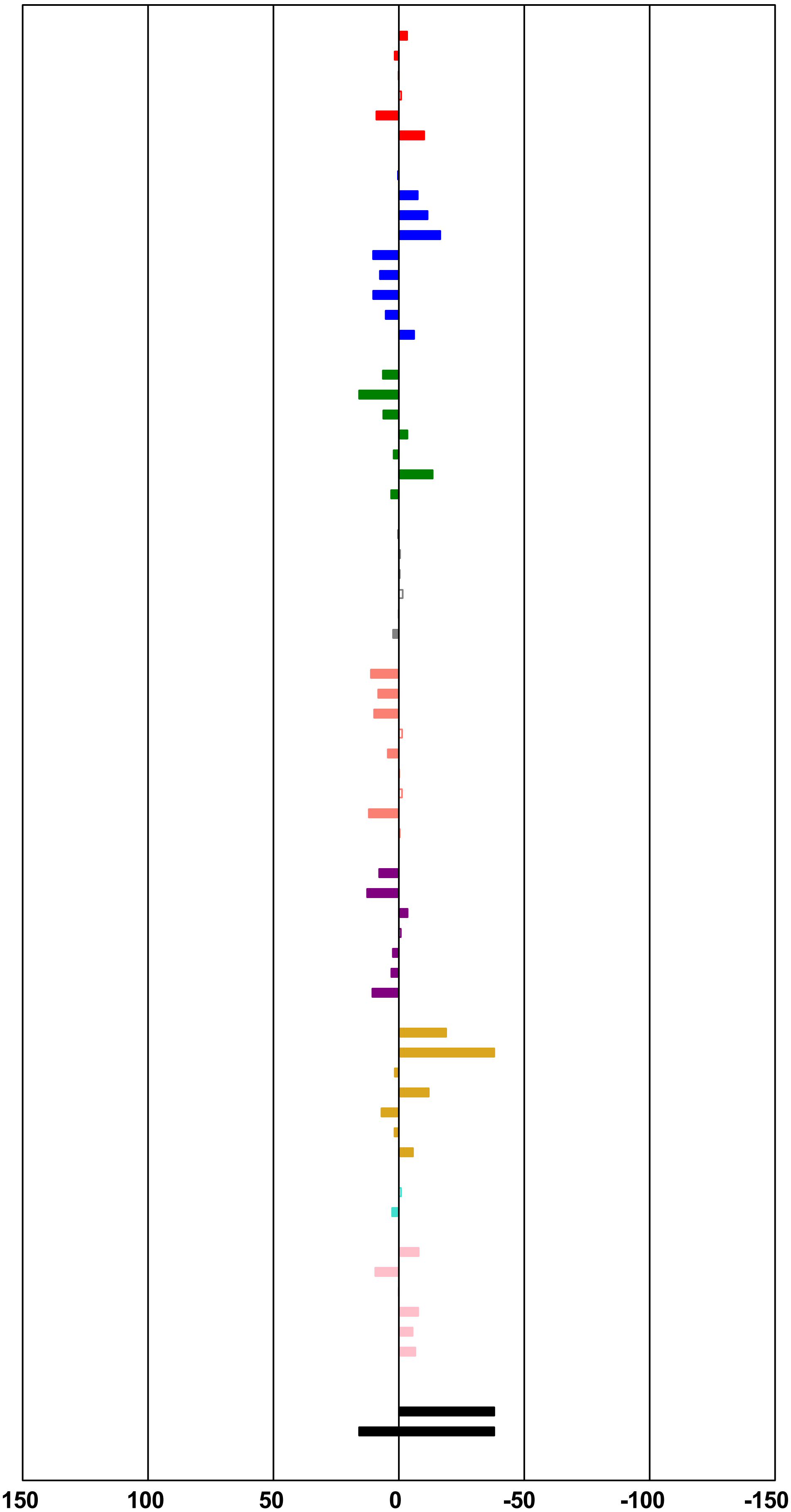

9a

Figure 11. Mean graph of compound **9a**

Developmental Therapeutics Program One Dose Mean Graph	NSC: D-832040 / 1	Conc: 1.00E-5 Molar	Test Date: Aug 09, 2021
	Experiment ID: 2108OS72		Report Date: Sep 15, 2021

Panel/Cell Line	Growth Percent
**Leukemia**	
CCRF-CEM	95.87
HL-60(TB)	98.53
K-562	93.67
MOLT-4	101.45
RPMI-8226	97.63
SR	84.30
**Non-Small Cell Lung Cancer**	
A549/ATCC	91.37
EKVX	101.76
HOP-62	103.03
HOP-92	95.86
NCI-H226	107.06
NCI-H23	93.80
NCI-H322M	107.80
NCI-H460	82.11
NCI-H522	101.77
**Colon Cancer**	
COLO 205	105.70
HCC-2998	108.02
HCT-116	100.52
HCT-15	91.60
HT29	95.42
KM12	92.02
SW-620	94.48
**CNS Cancer**	
SF-268	95.57
SF-295	101.75
SF-539	85.86
SNB-19	97.86
SNB-75	86.08
U251	103.10
**Melanoma**	
LOX IMVI	96.43
MALME-3M	107.57
M14	87.29
MDA-MB-435	86.20
SK-MEL-2	116.89
SK-MEL-28	101.57
SK-MEL-5	98.81
UACC-257	106.02
UACC-62	100.49
**Ovarian Cancer**	
IGROV1	107.31
OVCAR-3	118.41
OVCAR-4	75.15
OVCAR-5	104.00
OVCAR-8	99.67
NCI/ADR-RES	100.51
SK-OV-3	123.82
**Renal Cancer**	
786-0	94.23
A498	79.28
ACHN	106.34
CAKI-1	94.04
RXF 393	100.44
SN12C	103.06
UO-31	103.95
**Prostate Cancer**	
PC-3	100.41
DU-145	70.87
**Breast Cancer**	
MCF7	89.03
MDA-MB-231/ATCC	104.36
HS 578T	76.98
BT-549	81.62
T-47D	94.27
MDA-MB-468	100.95
Mean	97.36
Delta	26.49
Range	52.95

Mean Growth Percent - Growth Percent

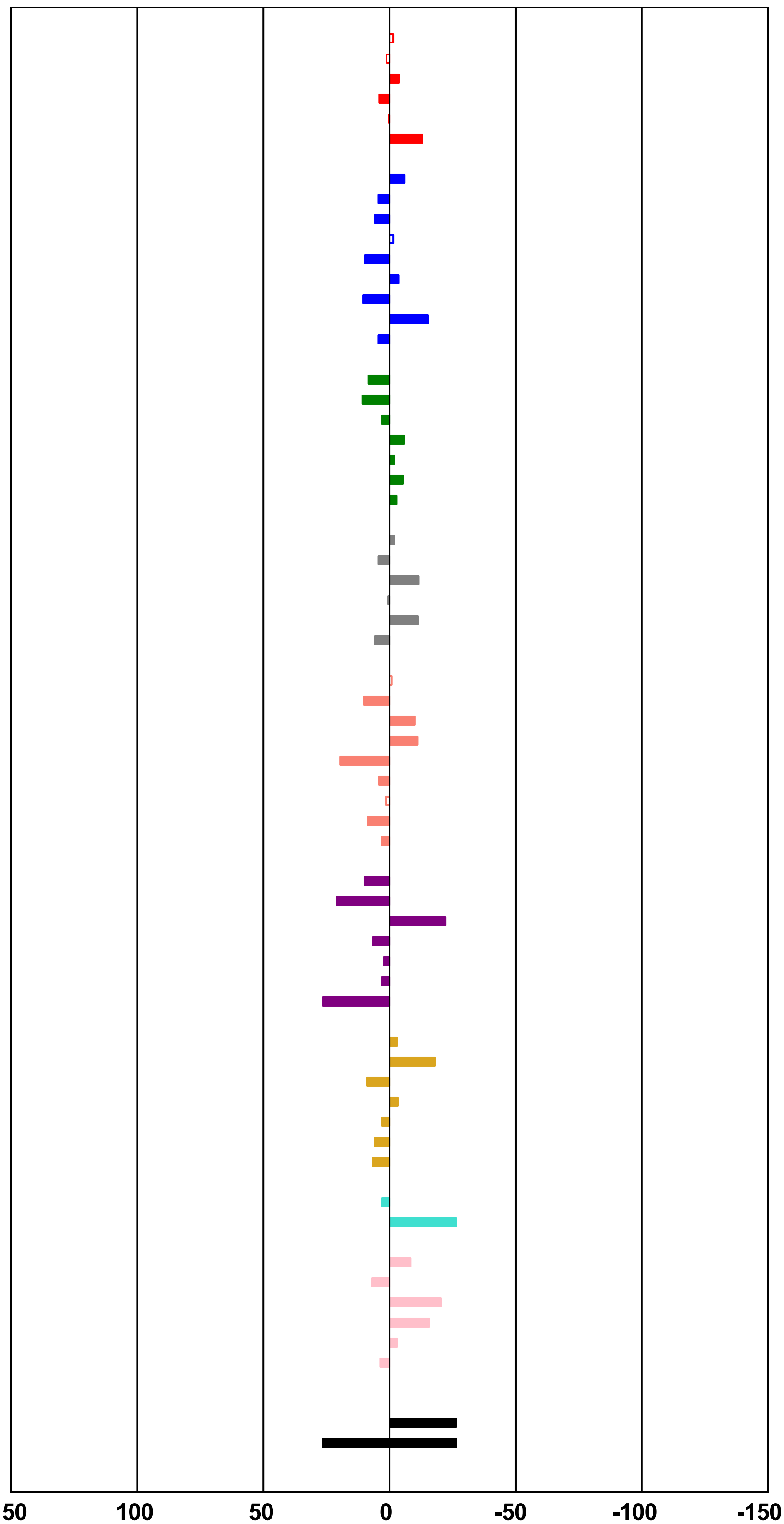

9b

Figure 12. Mean graph of compound **9b**

Developmental Therapeutics Program One Dose Mean Graph	NSC: D-832041 / 1	Conc: 1.00E-5 Molar	Test Date: Aug 09, 2021
	Experiment ID: 2108OS72		Report Date: Sep 15, 2021

Panel/Cell Line	Growth Percent
**Leukemia**	
CCRF-CEM	96.11
HL-60(TB)	103.76
K-562	99.50
MOLT-4	102.31
RPMI-8226	104.06
SR	93.26
**Non-Small Cell Lung Cancer**	
A549/ATCC	101.21
EKVX	92.76
HOP-62	97.27
HOP-92	92.51
NCI-H226	112.11
NCI-H23	101.45
NCI-H322M	104.68
NCI-H460	106.15
NCI-H522	97.91
**Colon Cancer**	
COLO 205	109.74
HCC-2998	109.32
HCT-116	104.70
HCT-15	98.70
HT29	102.06
KM12	97.89
SW-620	106.43
**CNS Cancer**	
SF-268	95.35
SF-295	99.29
SF-539	97.27
SNB-19	96.30
SNB-75	101.21
U251	103.68
**Melanoma**	
LOX IMVI	99.78
MALME-3M	112.79
M14	108.63
MDA-MB-435	95.53
SK-MEL-2	104.71
SK-MEL-28	101.83
SK-MEL-5	99.21
UACC-257	105.58
UACC-62	99.79
**Ovarian Cancer**	
IGROV1	106.94
OVCAR-3	106.17
OVCAR-4	102.86
OVCAR-5	98.85
OVCAR-8	101.08
NCI/ADR-RES	102.93
SK-OV-3	116.86
**Renal Cancer**	
786-0	86.94
A498	66.32
ACHN	105.26
CAKI-1	90.67
RXF 393	109.81
SN12C	97.72
UO-31	90.74
**Prostate Cancer**	
PC-3	96.73
DU-145	102.66
**Breast Cancer**	
MCF7	91.70
MDA-MB-231/ATCC	109.17
HS 578T	99.64
BT-549	109.21
T-47D	88.32
MDA-MB-468	102.79
Mean	100.65
Delta	34.33
Range	50.54

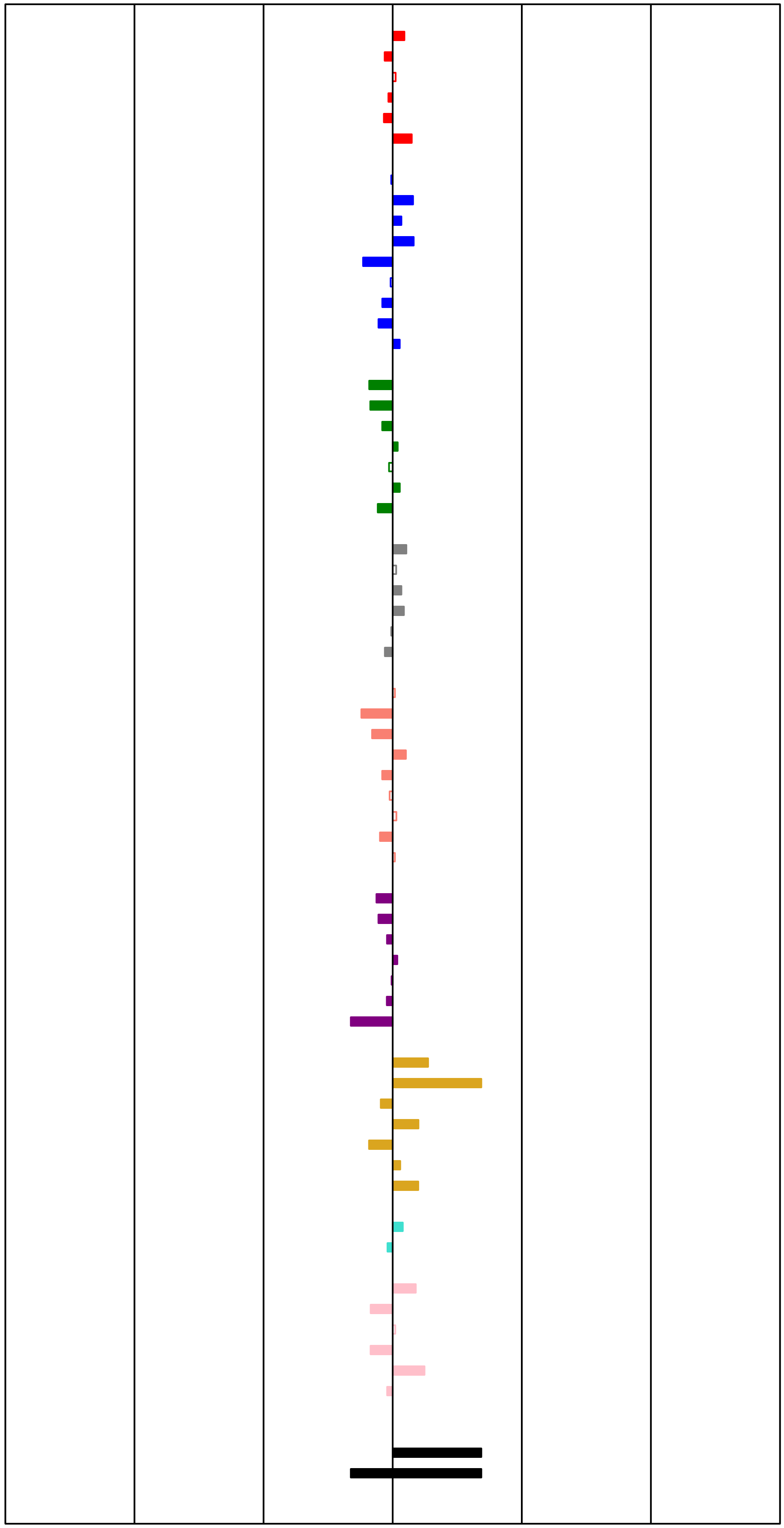

9c

Figure 13. Mean graph of compound **9c**

Developmental Therapeutics Program	NSC: D-832043 / 1	Conc: 1.00E-5 Molar	Test Date: Aug 09, 2021
One Dose Mean Graph	Experiment ID: 2108OS72		Report Date: Sep 15, 2021

Panel/Cell Line	Growth Percent
Leukemia	
CCRF-CEM	89.34
HL-60(TB)	112.34
MOLT-4	94.68
RPMI-8226	88.71
Non-Small Cell Lung Cancer	
A549/ATCC	69.00
EKVX	69.21
HOP-62	61.29
HOP-92	46.96
NCI-H226	42.34
NCI-H23	74.88
NCI-H322M	95.50
NCI-H460	61.52
NCI-H522	83.28
Colon Cancer	
COLO 205	96.34
HCC-2998	92.52
HCT-116	77.77
HCT-15	84.63
HT29	76.66
KM12	83.01
SW-620	94.72
CNS Cancer	
SF-268	61.87
SF-295	42.80
SF-539	52.08
SNB-19	76.50
SNB-75	-15.32
U251	71.35
Melanoma	
LOX IMVI	70.96
MALME-3M	61.07
M14	78.64
MDA-MB-435	79.04
SK-MEL-2	89.17
SK-MEL-28	78.44
SK-MEL-5	87.45
UACC-257	95.42
UACC-62	80.36
Ovarian Cancer	
IGROV1	85.53
OVCAR-3	100.90
OVCAR-4	35.87
OVCAR-5	100.89
OVCAR-8	66.13
NCI/ADR-RES	73.33
SK-OV-3	55.90
Renal Cancer	
786-0	32.81
A498	75.53
ACHN	38.53
CAKI-1	52.77
RXF 393	46.00
SN12C	86.61
UO-31	76.69
Prostate Cancer	
PC-3	66.07
DU-145	66.35
Breast Cancer	
MCF7	70.31
MDA-MB-231/ATCC	78.08
HS 578T	19.39
BT-549	52.65
T-47D	61.35
MDA-MB-468	73.14
Mean	70.52
Delta	85.84
Range	127.66

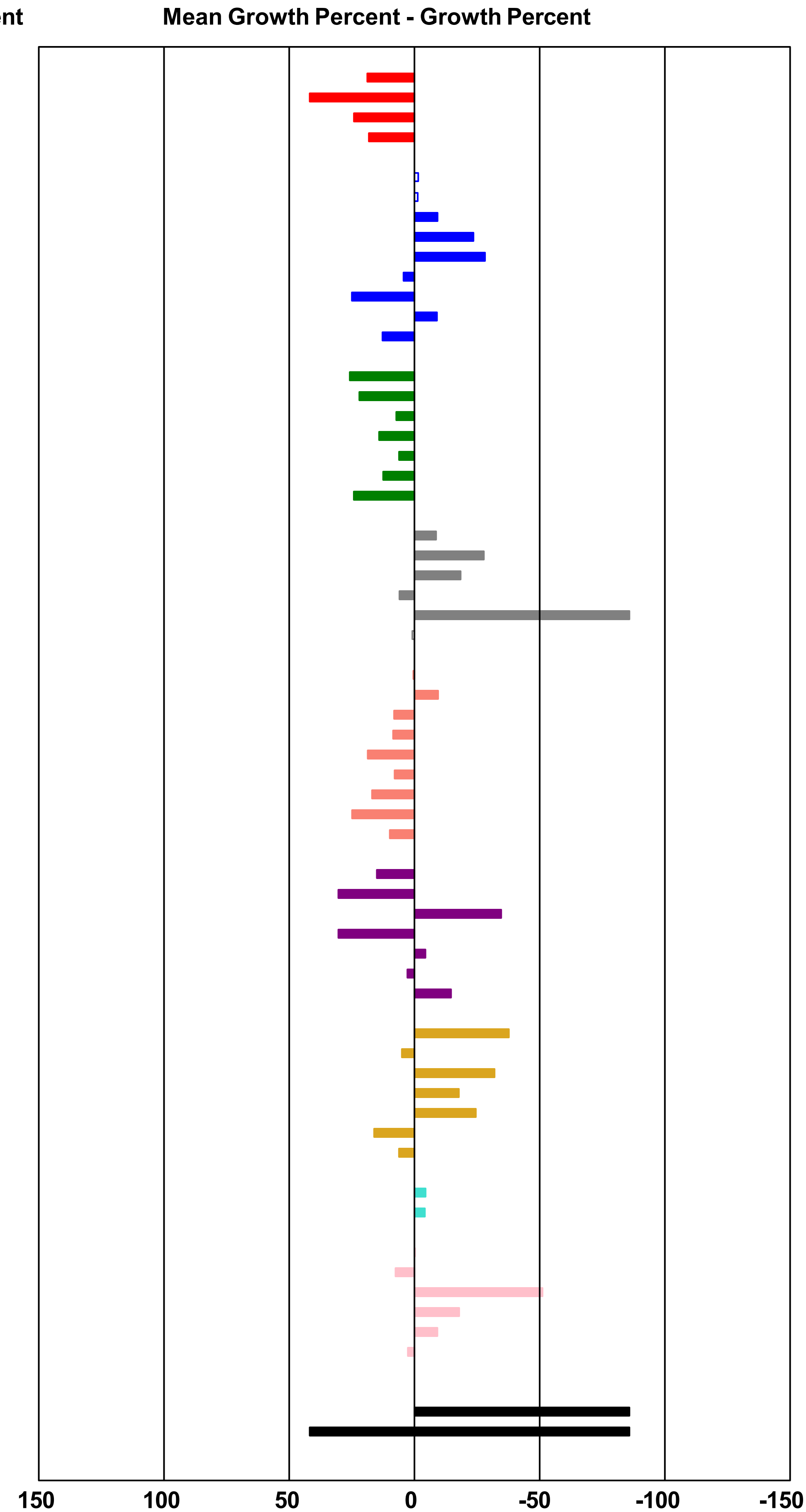

9d

Figure 14. Mean graph of compound **9d**

Developmental Therapeutics Program<br>One Dose Mean Graph	NSC: D-832513 / 1	Conc: 1.00E-5 Molar	Test Date: Aug 30, 2021
	Experiment ID: 2108OS84		Report Date: Oct 29, 2021

Panel/Cell Line	Growth Percent
**Leukemia**	
CCRF-CEM	52.79
HL-60(TB)	41.49
K-562	30.47
MOLT-4	26.32
RPMI-8226	30.41
SR	34.66
**Non-Small Cell Lung Cancer**	
A549/ATCC	61.91
EKVX	50.64
HOP-62	92.52
HOP-92	56.61
NCI-H226	65.08
NCI-H23	57.53
NCI-H322M	67.60
NCI-H460	49.78
NCI-H522	69.59
**Colon Cancer**	
COLO 205	66.26
HCC-2998	90.49
HCT-116	47.21
HCT-15	40.00
HT29	71.09
KM12	58.40
SW-620	67.67
**CNS Cancer**	
SF-268	61.36
SF-295	61.53
SF-539	80.59
SNB-19	70.96
SNB-75	57.83
U251	56.91
**Melanoma**	
MALME-3M	86.83
M14	66.81
MDA-MB-435	42.33
SK-MEL-2	74.08
SK-MEL-28	84.05
SK-MEL-5	86.22
UACC-257	85.34
UACC-62	61.60
**Ovarian Cancer**	
IGROV1	54.77
OVCAR-3	62.84
OVCAR-4	62.95
OVCAR-5	90.04
OVCAR-8	74.06
NCI/ADR-RES	60.10
SK-OV-3	105.74
**Renal Cancer**	
786-0	59.39
ACHN	53.14
CAKI-1	48.38
RXF 393	58.90
SN12C	57.96
TK-10	109.72
UO-31	36.18
**Prostate Cancer**	
PC-3	43.70
DU-145	75.24
**Breast Cancer**	
MCF7	32.32
MDA-MB-231/ATCC	69.46
HS 578T	89.40
BT-549	102.90
T-47D	48.06
MDA-MB-468	59.26
Mean	63.09
Delta	36.77
Range	83.40

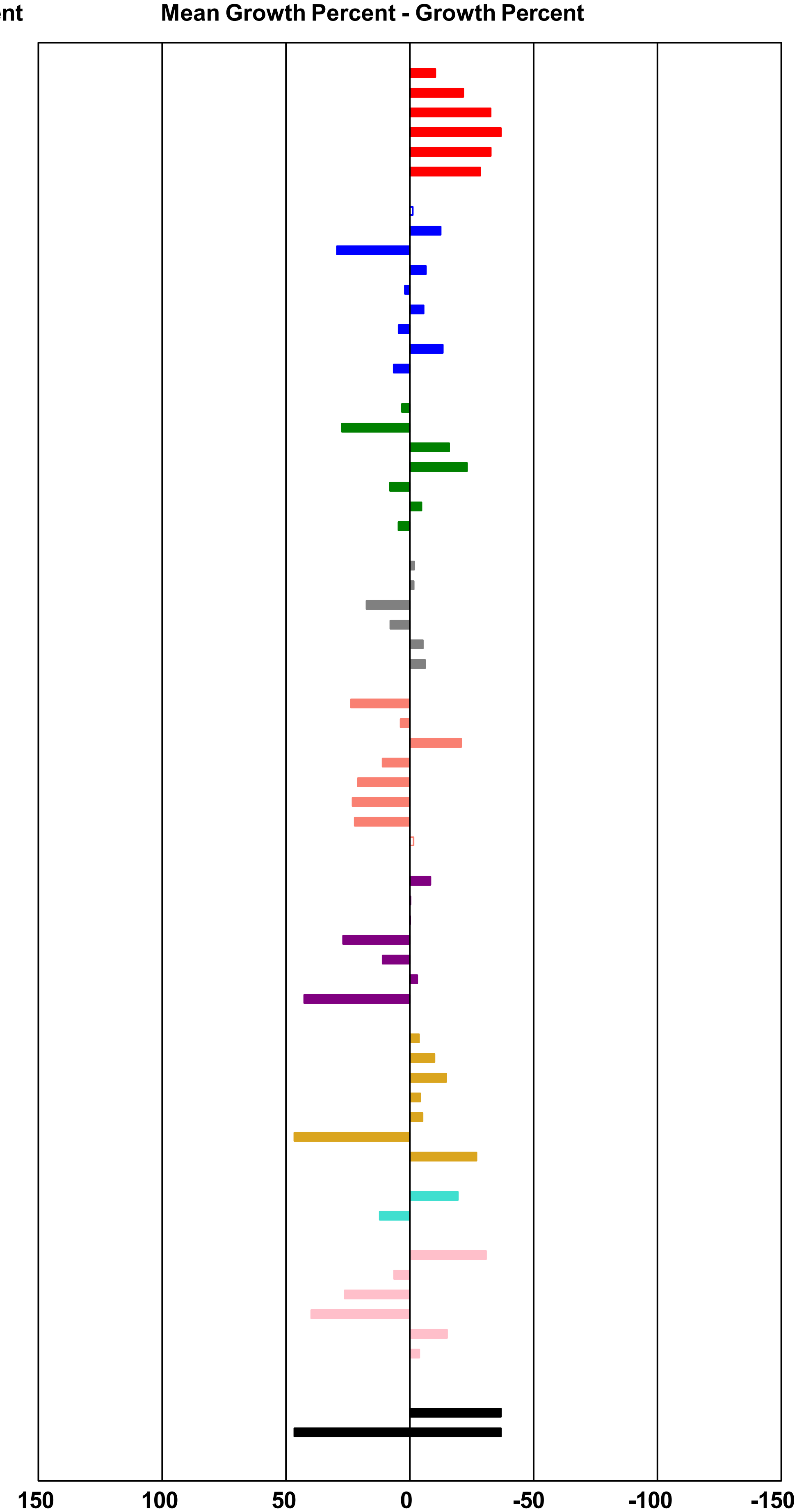

9e

Figure 15. Mean graph of compound **9e**

Developmental Therapeutics Program	NSC: D-832042 / 1	Conc: 1.00E-5 Molar	Test Date: Aug 09, 2021
One Dose Mean Graph	Experiment ID: 2108OS72		Report Date: Sep 15, 2021

Panel/Cell Line	Growth Percent
**Leukemia**	
CCRF-CEM	87.94
HL-60(TB)	106.33
K-562	103.08
MOLT-4	107.72
RPMI-8226	103.10
SR	98.99
**Non-Small Cell Lung Cancer**	
A549/ATCC	104.88
EKVX	86.91
HOP-62	105.33
HOP-92	90.86
NCI-H226	99.27
NCI-H23	103.45
NCI-H322M	104.63
NCI-H460	95.82
NCI-H522	96.32
**Colon Cancer**	
COLO 205	112.05
HCC-2998	109.65
HCT-116	101.30
HCT-15	95.92
HT29	100.35
KM12	95.39
SW-620	102.19
**CNS Cancer**	
SF-268	91.55
SF-295	95.24
SF-539	101.08
SNB-19	100.76
SNB-75	98.05
U251	104.30
**Melanoma**	
LOX IMVI	107.46
MALME-3M	103.16
M14	99.34
MDA-MB-435	94.58
SK-MEL-2	102.10
SK-MEL-28	98.54
SK-MEL-5	98.59
UACC-257	105.08
UACC-62	95.50
**Ovarian Cancer**	
IGROV1	107.93
OVCAR-3	109.07
OVCAR-4	102.07
OVCAR-5	98.07
OVCAR-8	98.99
NCI/ADR-RES	100.15
SK-OV-3	121.52
**Renal Cancer**	
786-0	94.00
A498	64.60
ACHN	100.44
CAKI-1	90.65
RXF 393	98.08
SN12C	100.27
UO-31	91.19
**Prostate Cancer**	
PC-3	95.30
DU-145	89.27
**Breast Cancer**	
MCF7	85.80
MDA-MB-231/ATCC	104.86
HS 578T	90.24
BT-549	96.17
T-47D	87.70
MDA-MB-468	103.62
Mean	98.93
Delta	34.33
Range	56.92

Mean Growth Percent - Growth Percent

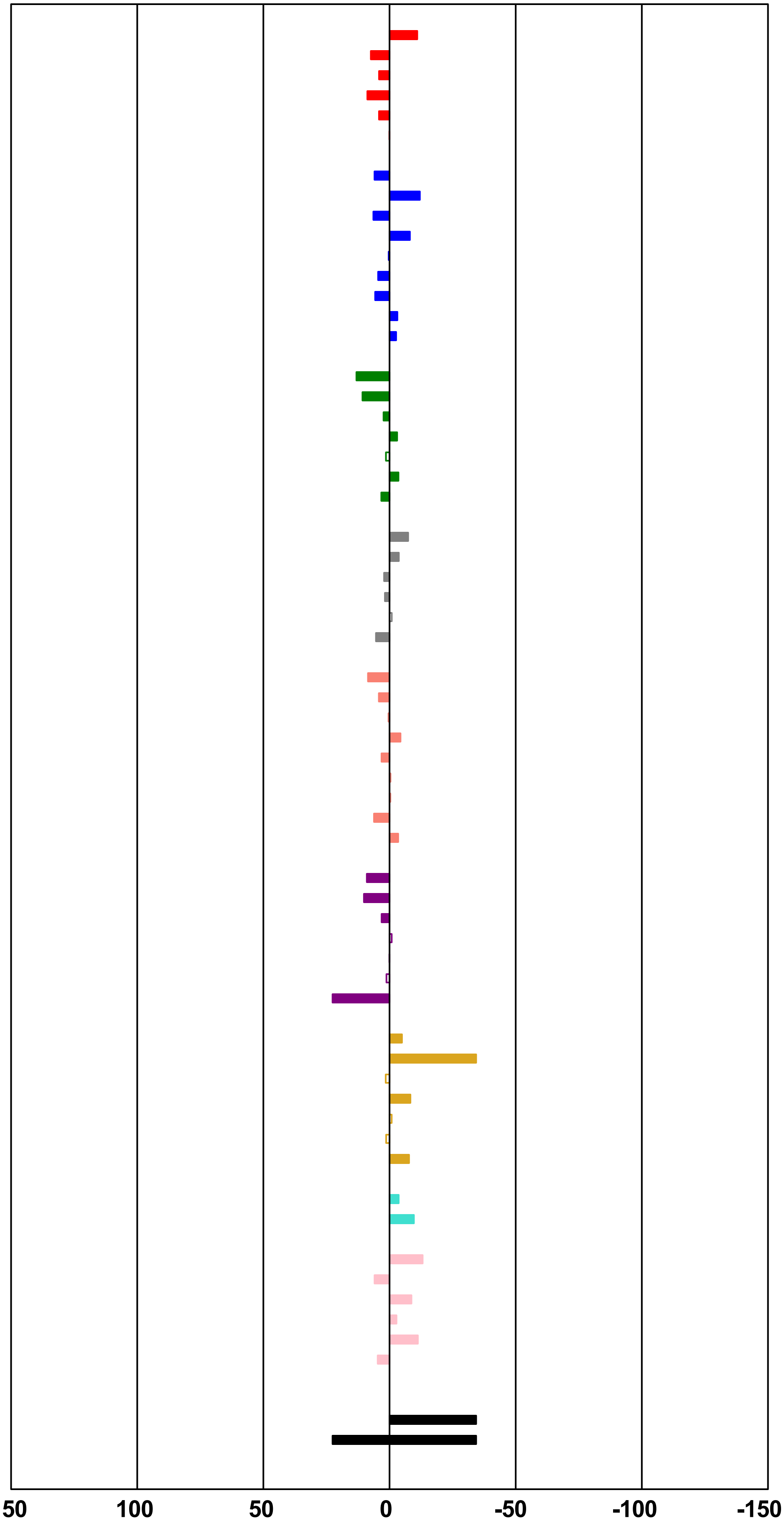

9f

Figure 16. Mean graph of compound **9f**

Developmental Therapeutics Program One Dose Mean Graph	**NSC:** D-832520 / 1	**Conc:** 1.00E-5 Molar	**Test Date:** Aug 30, 2021
	**Experiment ID:** 2108OS84		**Report Date:** Oct 29, 2021

Panel/Cell Line	Growth Percent
Leukemia	
CCRF-CEM	68.05
HL-60(TB)	72.57
K-562	52.30
MOLT-4	46.00
RPMI-8226	70.42
SR	64.98
Non-Small Cell Lung Cancer	
A549/ATCC	96.10
EKVX	83.13
HOP-62	91.65
HOP-92	99.46
NCI-H226	90.59
NCI-H23	83.85
NCI-H322M	100.97
NCI-H460	86.80
NCI-H522	90.45
Colon Cancer	
COLO 205	93.43
HCC-2998	109.60
HCT-116	87.42
HCT-15	69.77
HT29	97.02
KM12	87.31
SW-620	86.49
CNS Cancer	
SF-268	85.60
SF-295	86.47
SF-539	88.13
SNB-19	93.98
SNB-75	82.44
U251	88.70
Melanoma	
MALME-3M	91.85
M14	91.23
MDA-MB-435	81.18
SK-MEL-2	100.01
SK-MEL-28	100.42
SK-MEL-5	88.69
UACC-257	100.73
UACC-62	84.57
Ovarian Cancer	
IGROV1	93.08
OVCAR-3	109.68
OVCAR-4	86.99
OVCAR-5	107.72
OVCAR-8	97.40
NCI/ADR-RES	97.39
SK-OV-3	93.20
Renal Cancer	
786-0	80.78
ACHN	81.59
CAKI-1	91.23
RXF 393	84.89
SN12C	95.92
TK-10	108.73
UO-31	78.86
Prostate Cancer	
PC-3	80.26
DU-145	83.23
Breast Cancer	
MCF7	67.62
MDA-MB-231/ATCC	78.59
HS 578T	79.60
BT-549	98.66
T-47D	64.64
MDA-MB-468	82.51
Mean	86.81
Delta	40.81
Range	63.68

Mean Growth Percent - Growth Percent

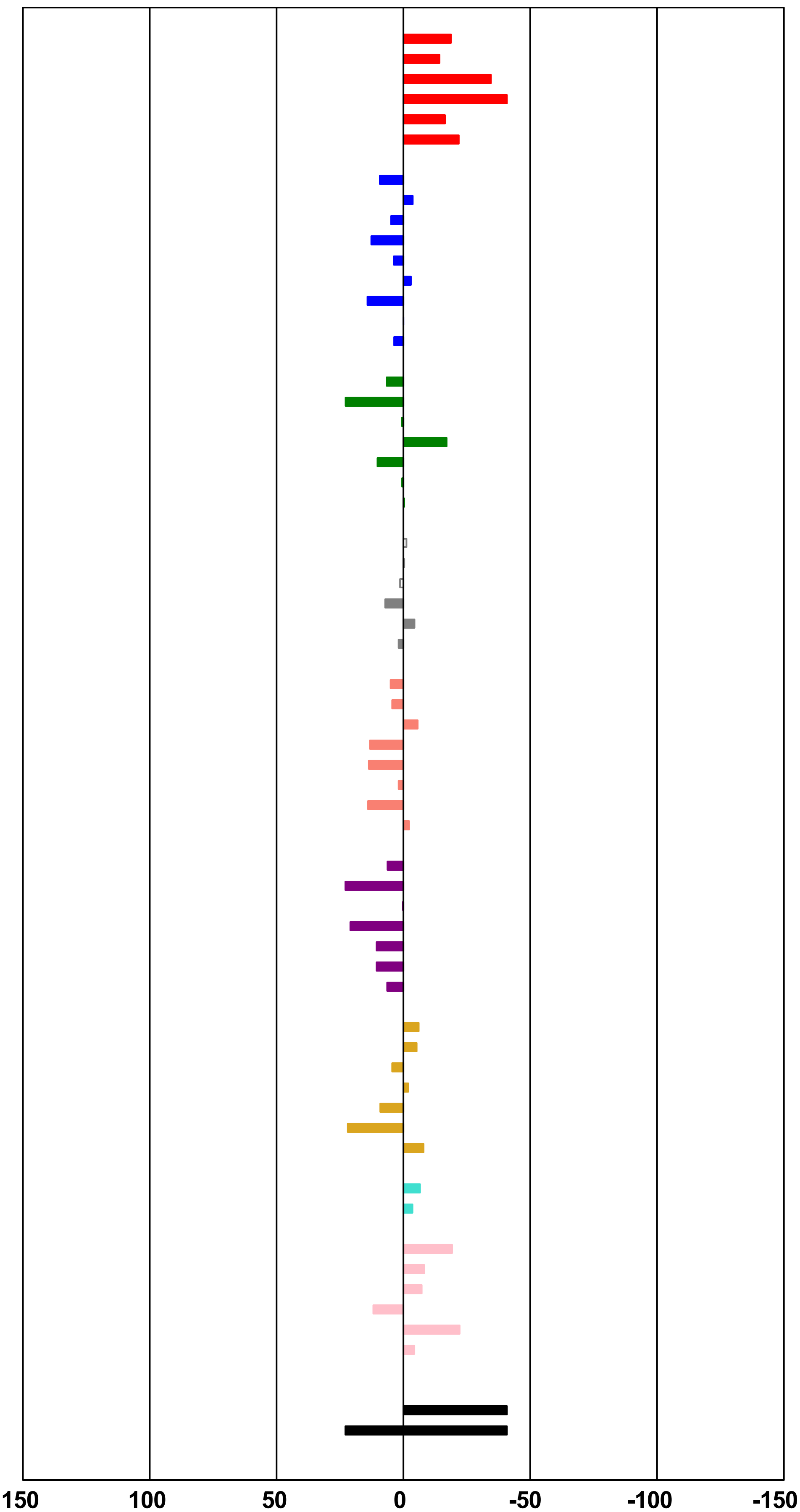

9g

Figure 17. Mean graph of compound **9g**

Developmental Therapeutics Program

One Dose Mean Graph

**NSC:** D-832521 / 1	**Conc:** 1.00E-5 Molar	**Test Date:** Aug 30, 2021
**Experiment ID:** 2108OS84		**Report Date:** Oct 29, 2021

Panel/Cell Line	Growth Percent
Leukemia	
CCRF-CEM	76.58
HL-60(TB)	86.36
K-562	71.49
MOLT-4	48.84
RPMI-8226	70.00
SR	58.13
Non-Small Cell Lung Cancer	
A549/ATCC	99.93
EKVX	86.46
HOP-62	93.80
HOP-92	75.94
NCI-H226	84.50
NCI-H23	77.75
NCI-H322M	85.52
NCI-H460	87.82
NCI-H522	82.32
Colon Cancer	
COLO 205	109.25
HCC-2998	106.94
HCT-116	74.88
HCT-15	66.78
HT29	91.80
KM12	90.58
SW-620	88.74
CNS Cancer	
SF-268	83.72
SF-295	93.20
SF-539	82.47
SNB-19	82.46
SNB-75	64.20
U251	91.73
Melanoma	
MALME-3M	104.23
M14	88.54
MDA-MB-435	73.20
SK-MEL-2	95.10
SK-MEL-28	103.52
SK-MEL-5	95.84
UACC-257	109.22
UACC-62	66.40
Ovarian Cancer	
IGROV1	89.18
OVCAR-3	95.54
OVCAR-4	78.78
OVCAR-5	94.19
OVCAR-8	92.14
NCI/ADR-RES	87.95
SK-OV-3	119.58
Renal Cancer	
786-0	87.55
ACHN	84.29
CAKI-1	82.07
RXF 393	80.45
SN12C	90.01
TK-10	147.86
UO-31	79.99
Prostate Cancer	
PC-3	78.98
DU-145	99.03
Breast Cancer	
MCF7	37.82
MDA-MB-231/ATCC	78.96
HS 578T	87.36
BT-549	110.98
T-47D	76.80
MDA-MB-468	70.77
Mean	86.18
Delta	48.36
Range	110.04

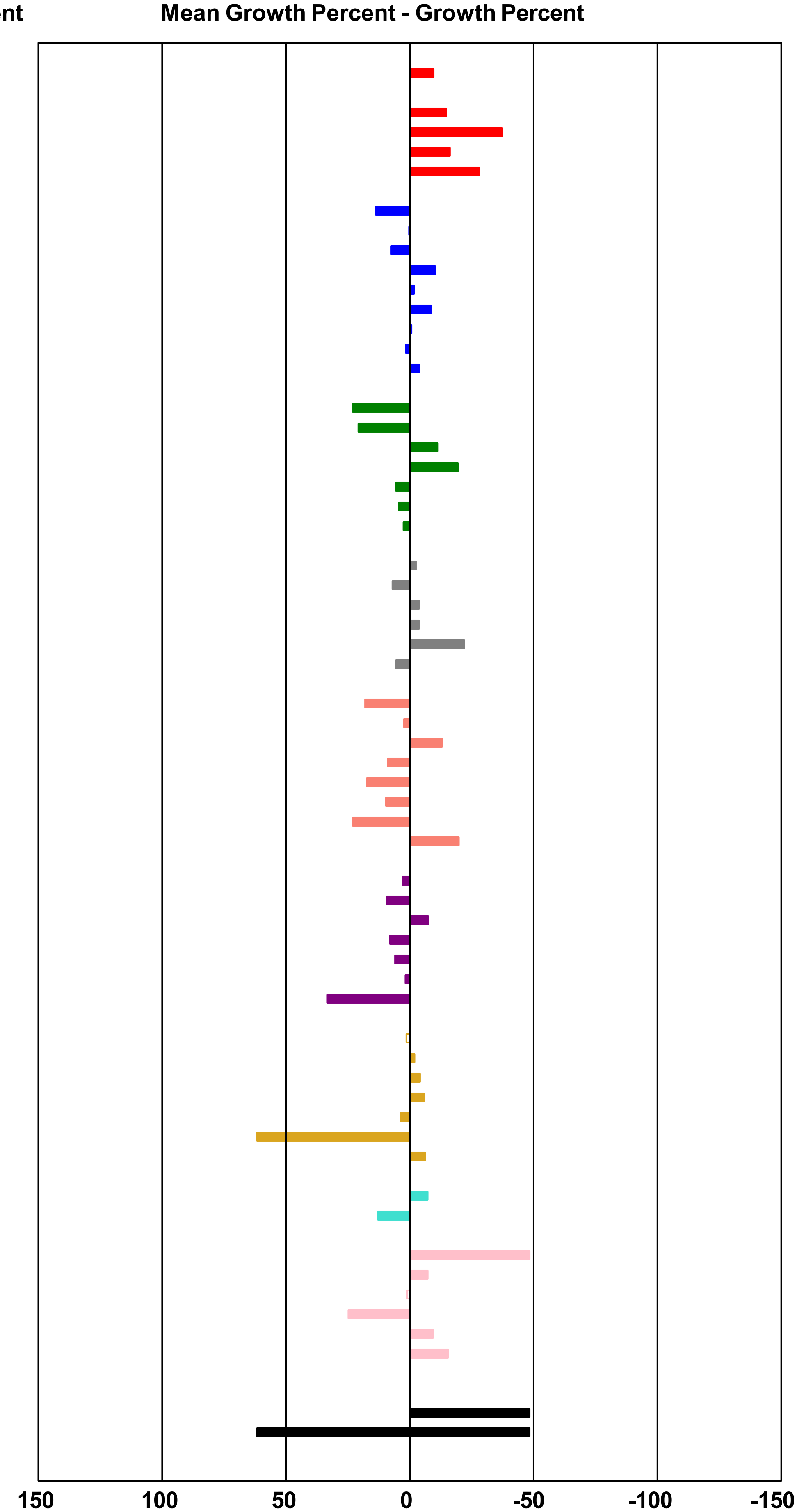

9h

Figure 18. Mean graph of compound **9h**

Developmental Therapeutics Program One Dose Mean Graph	**NSC:** D-832514 / 1	**Conc:** 1.00E-5 Molar	**Test Date:** Aug 30, 2021
	**Experiment ID:** 2108OS84		**Report Date:** Oct 29, 2021

Panel/Cell Line	Growth Percent
Leukemia	
CCRF-CEM	56.79
HL-60(TB)	77.79
K-562	90.66
MOLT-4	81.06
RPMI-8226	102.51
SR	73.49
Non-Small Cell Lung Cancer	
A549/ATCC	105.70
EKVX	84.11
HOP-62	91.28
HOP-92	88.67
NCI-H226	86.23
NCI-H23	83.03
NCI-H322M	95.29
NCI-H460	101.09
NCI-H522	93.20
Colon Cancer	
COLO 205	102.48
HCC-2998	101.97
HCT-116	96.55
HCT-15	92.91
HT29	99.37
KM12	107.59
SW-620	102.13
CNS Cancer	
SF-268	87.13
SF-295	103.14
SF-539	103.26
SNB-19	89.07
SNB-75	84.09
U251	96.38
Melanoma	
MALME-3M	88.47
M14	92.66
MDA-MB-435	96.99
SK-MEL-2	101.89
SK-MEL-28	92.55
SK-MEL-5	100.84
UACC-257	98.29
UACC-62	75.95
Ovarian Cancer	
IGROV1	73.82
OVCAR-3	86.14
OVCAR-4	90.23
OVCAR-5	100.82
OVCAR-8	96.57
NCI/ADR-RES	104.63
SK-OV-3	96.16
Renal Cancer	
786-0	103.35
ACHN	92.76
CAKI-1	89.26
RXF 393	104.65
SN12C	94.99
TK-10	199.04
UO-31	82.74
Prostate Cancer	
PC-3	89.34
DU-145	103.46
Breast Cancer	
MCF7	79.31
MDA-MB-231/ATCC	82.63
HS 578T	92.67
BT-549	112.67
T-47D	82.87
MDA-MB-468	87.45
Mean	94.31
Delta	37.52
Range	142.25

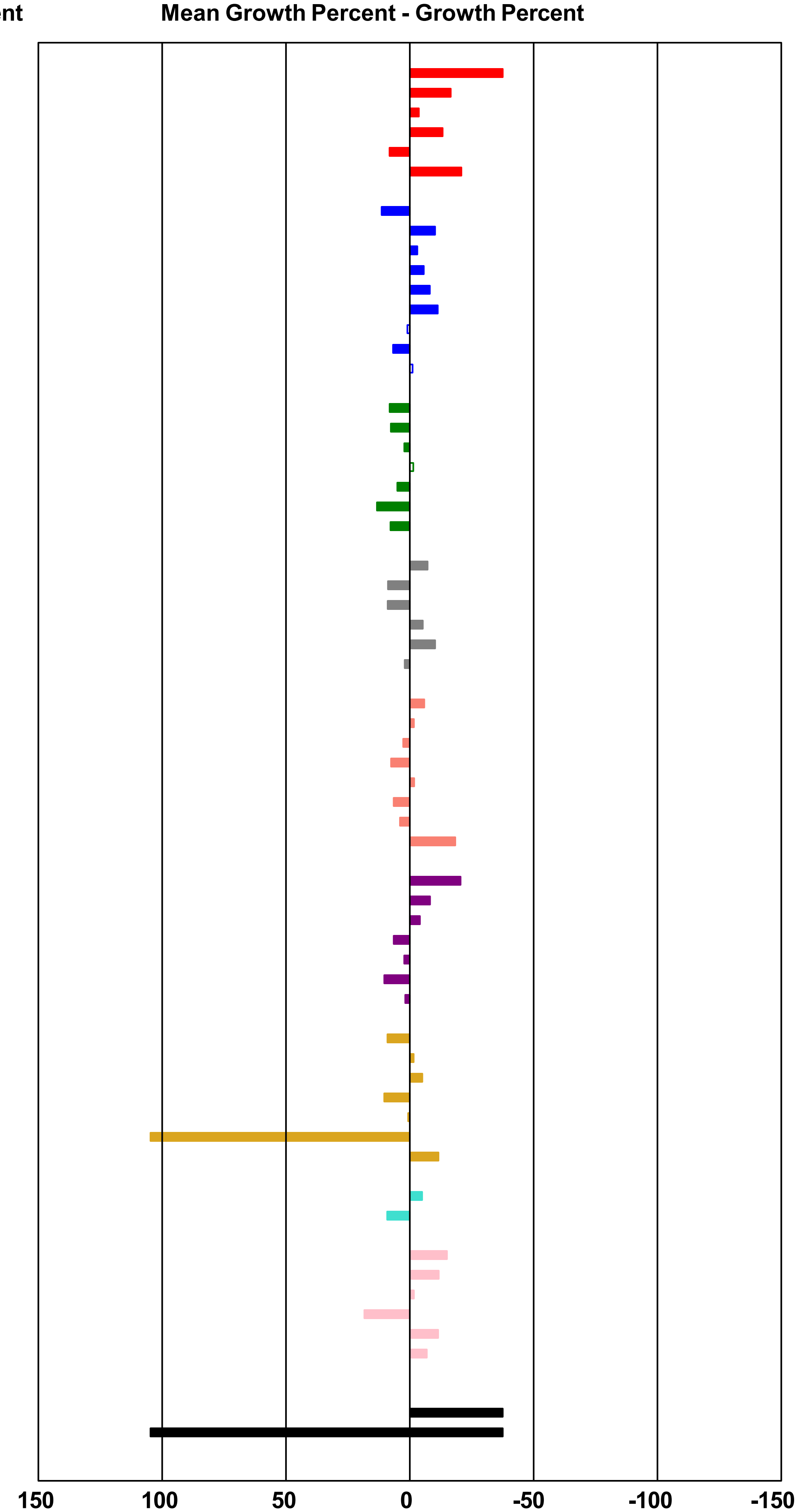

11

Figure 19. Mean graph of compound **11**

Developmental Therapeutics Program
One Dose Mean Graph

NSC: D-832515 / 1	Conc: 1.00E-5 Molar	Test Date: Aug 30, 2021
Experiment ID: 2108OS84		Report Date: Oct 29, 2021

Panel/Cell Line	Growth Percent
**Leukemia**	
CCRF-CEM	102.56
HL-60(TB)	118.17
K-562	109.62
MOLT-4	80.06
RPMI-8226	109.50
SR	89.50
**Non-Small Cell Lung Cancer**	
A549/ATCC	95.26
EKVX	99.81
HOP-62	111.22
HOP-92	126.37
NCI-H226	111.48
NCI-H23	90.49
NCI-H322M	97.49
NCI-H460	117.58
NCI-H522	100.99
**Colon Cancer**	
COLO 205	120.16
HCC-2998	115.81
HCT-116	111.75
HCT-15	105.59
HT29	116.78
KM12	92.07
SW-620	111.91
**CNS Cancer**	
SF-268	115.42
SF-295	116.96
SF-539	111.43
SNB-19	112.64
SNB-75	85.59
U251	108.11
**Melanoma**	
MALME-3M	131.98
M14	115.11
MDA-MB-435	98.68
SK-MEL-2	122.44
SK-MEL-28	119.74
SK-MEL-5	99.23
UACC-257	110.49
UACC-62	115.47
**Ovarian Cancer**	
IGROV1	120.68
OVCAR-3	110.20
OVCAR-4	108.07
OVCAR-5	134.00
OVCAR-8	106.40
NCI/ADR-RES	112.72
SK-OV-3	105.81
**Renal Cancer**	
786-0	97.55
ACHN	106.08
CAKI-1	98.94
RXF 393	129.86
SN12C	103.03
TK-10	131.77
UO-31	101.98
**Prostate Cancer**	
PC-3	114.98
DU-145	115.94
**Breast Cancer**	
MCF7	91.46
MDA-MB-231/ATCC	107.03
HS 578T	93.13
BT-549	138.37
T-47D	96.13
MDA-MB-468	92.91
Mean	108.87
Delta	28.81
Range	58.31

Mean Growth Percent - Growth Percent

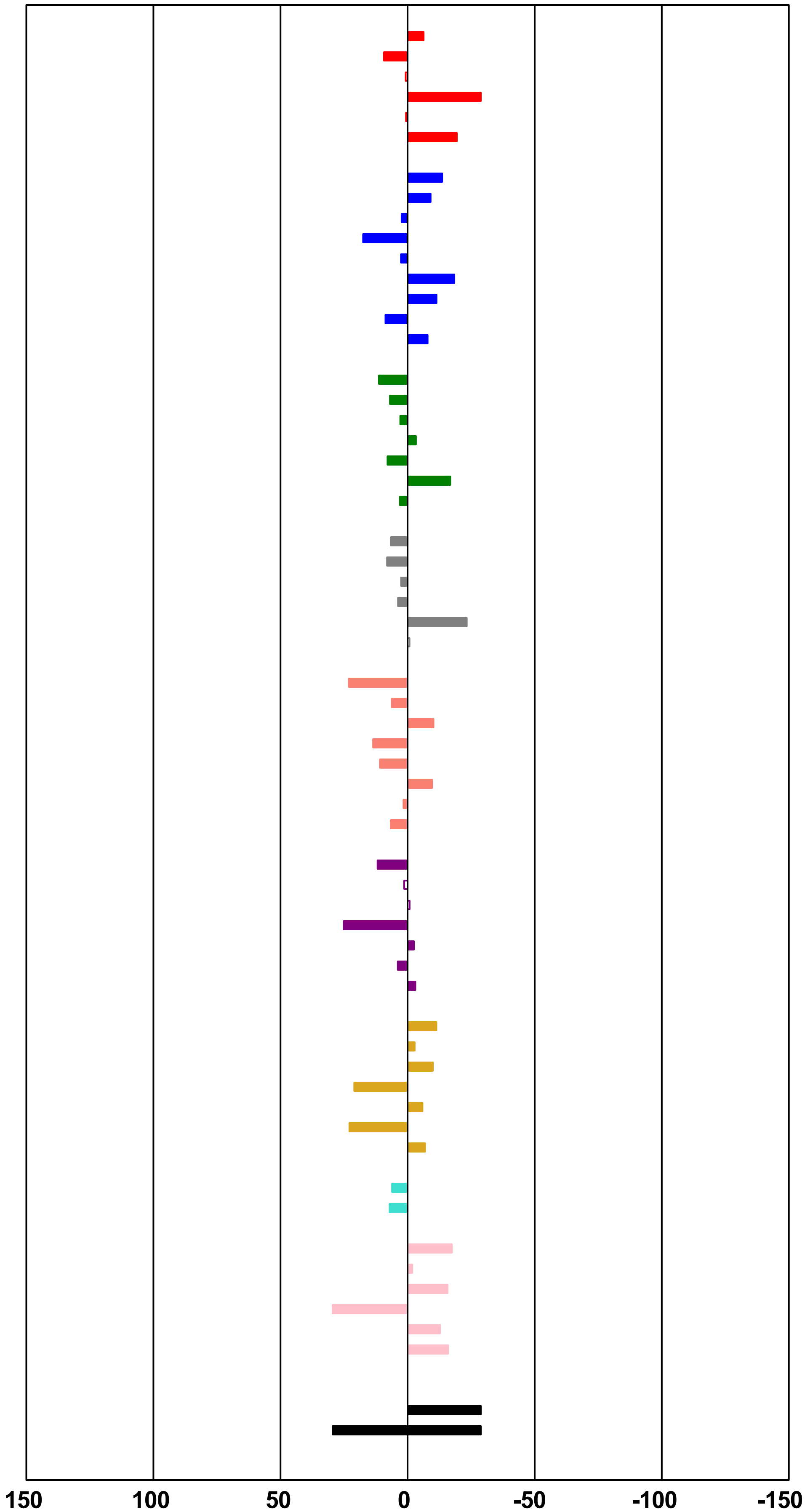

12

Figure 20. Mean graph of compound **12**

Developmental Therapeutics Program One Dose Mean Graph	NSC: D-832518 / 1	Conc: 1.00E-5 Molar	Test Date: Aug 30, 2021
	Experiment ID: 2108OS84		Report Date: Oct 29, 2021

Panel/Cell Line	Growth Percent
**Leukemia**	
CCRF-CEM	108.43
HL-60(TB)	79.72
K-562	108.72
MOLT-4	102.00
RPMI-8226	115.31
SR	89.16
**Non-Small Cell Lung Cancer**	
A549/ATCC	107.21
EKVX	100.04
HOP-62	93.55
HOP-92	97.47
NCI-H226	104.59
NCI-H23	98.37
NCI-H322M	112.49
NCI-H460	106.58
NCI-H522	102.47
**Colon Cancer**	
COLO 205	105.47
HCC-2998	116.26
HCT-116	116.25
HCT-15	95.65
HT29	105.74
KM12	102.54
SW-620	103.13
**CNS Cancer**	
SF-268	107.07
SF-295	100.08
SF-539	108.13
SNB-19	106.48
SNB-75	101.22
U251	105.05
**Melanoma**	
MALME-3M	113.24
M14	103.15
MDA-MB-435	110.40
SK-MEL-2	122.65
SK-MEL-28	106.54
SK-MEL-5	101.04
UACC-257	109.09
UACC-62	99.02
**Ovarian Cancer**	
IGROV1	103.56
OVCAR-3	121.71
OVCAR-4	104.19
OVCAR-5	105.23
OVCAR-8	108.32
NCI/ADR-RES	110.85
SK-OV-3	101.65
**Renal Cancer**	
786-0	103.48
ACHN	99.19
CAKI-1	90.30
RXF 393	103.06
SN12C	100.74
TK-10	158.60
UO-31	109.59
**Prostate Cancer**	
PC-3	106.70
DU-145	103.50
**Breast Cancer**	
MCF7	92.88
MDA-MB-231/ATCC	94.63
HS 578T	98.09
BT-549	122.22
T-47D	102.76
MDA-MB-468	96.67
Mean	105.21
Delta	25.49
Range	78.88

Mean Growth Percent - Growth Percent

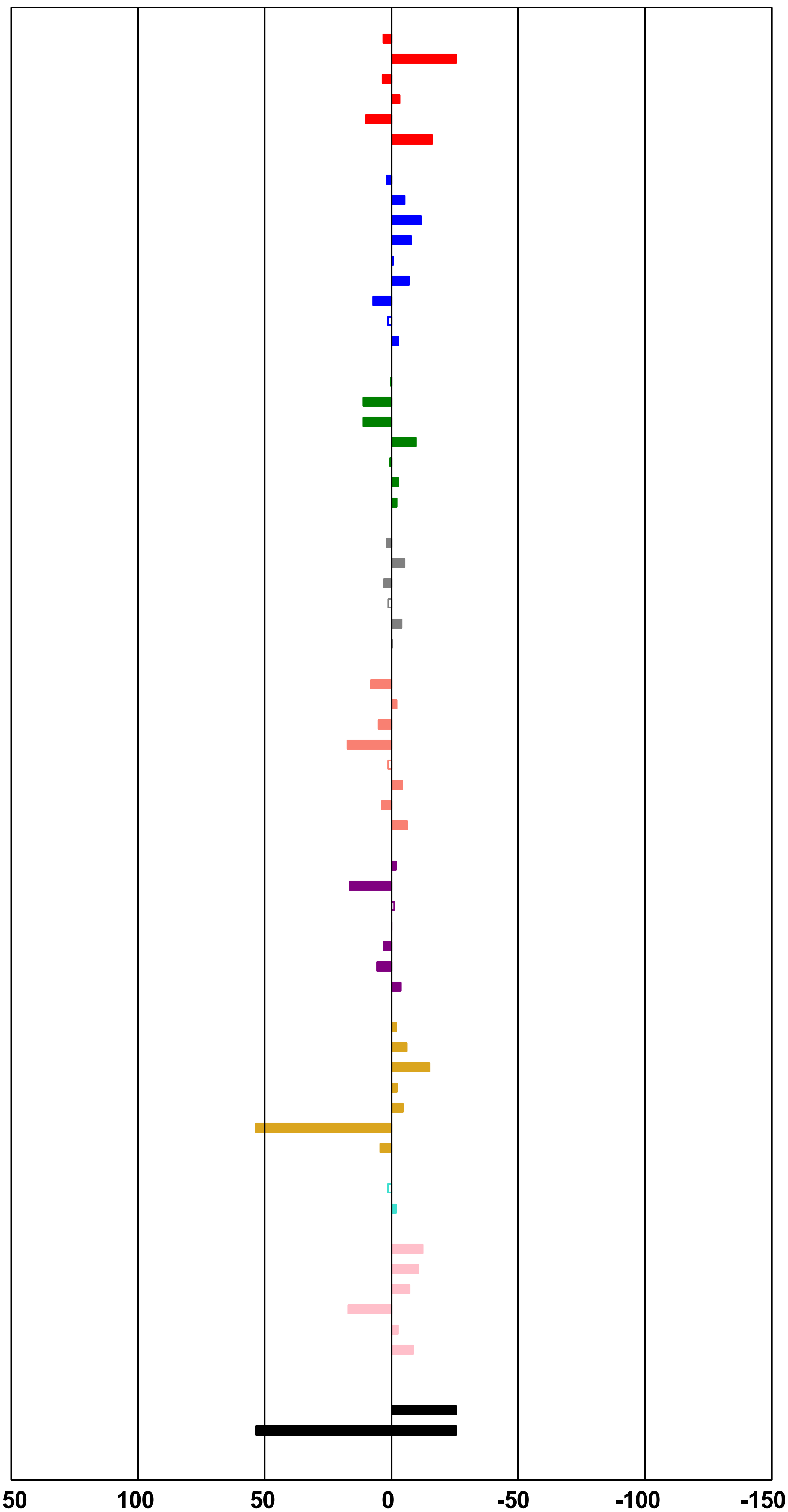

13

Figure 21. Mean graph of compound **13**

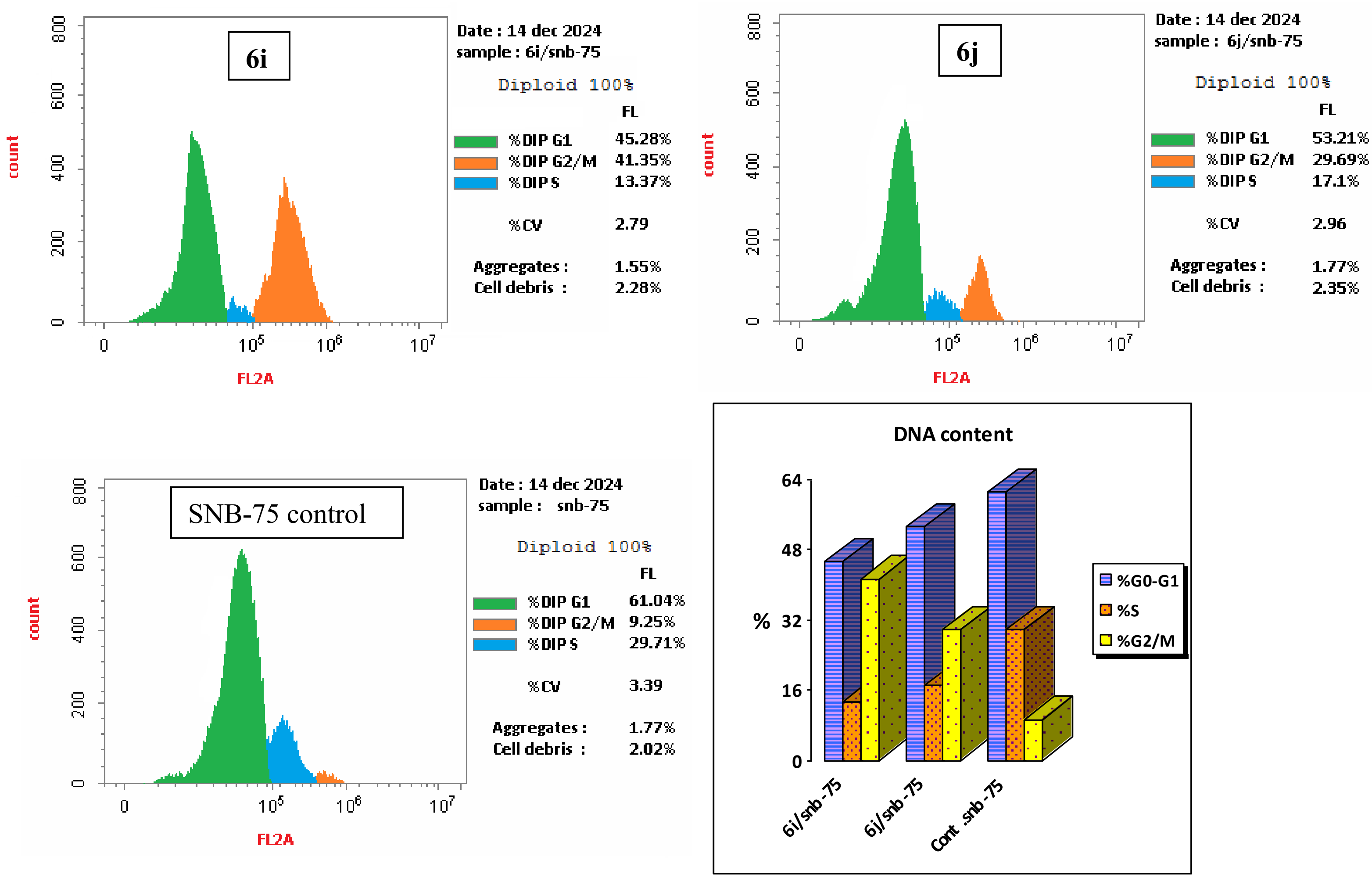

Figure 22. Assessment of cell cycle progression for compounds **6i** and **6j** on SNB-75 cell line (attempt1)

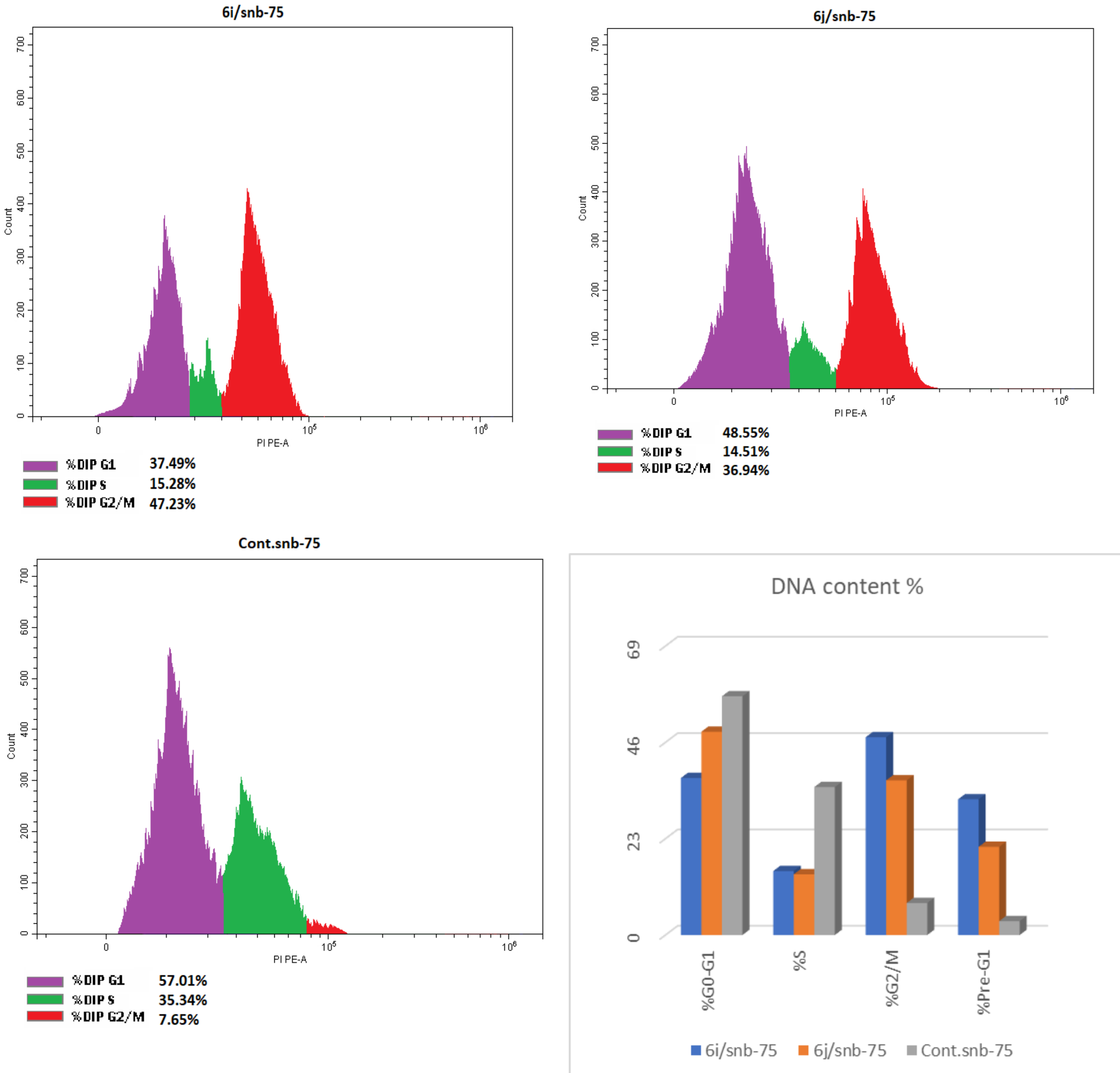

Figure 23. Assessment of cell cycle progression for compounds **6i** and **6j** on SNB-75 cell line (attempt2)

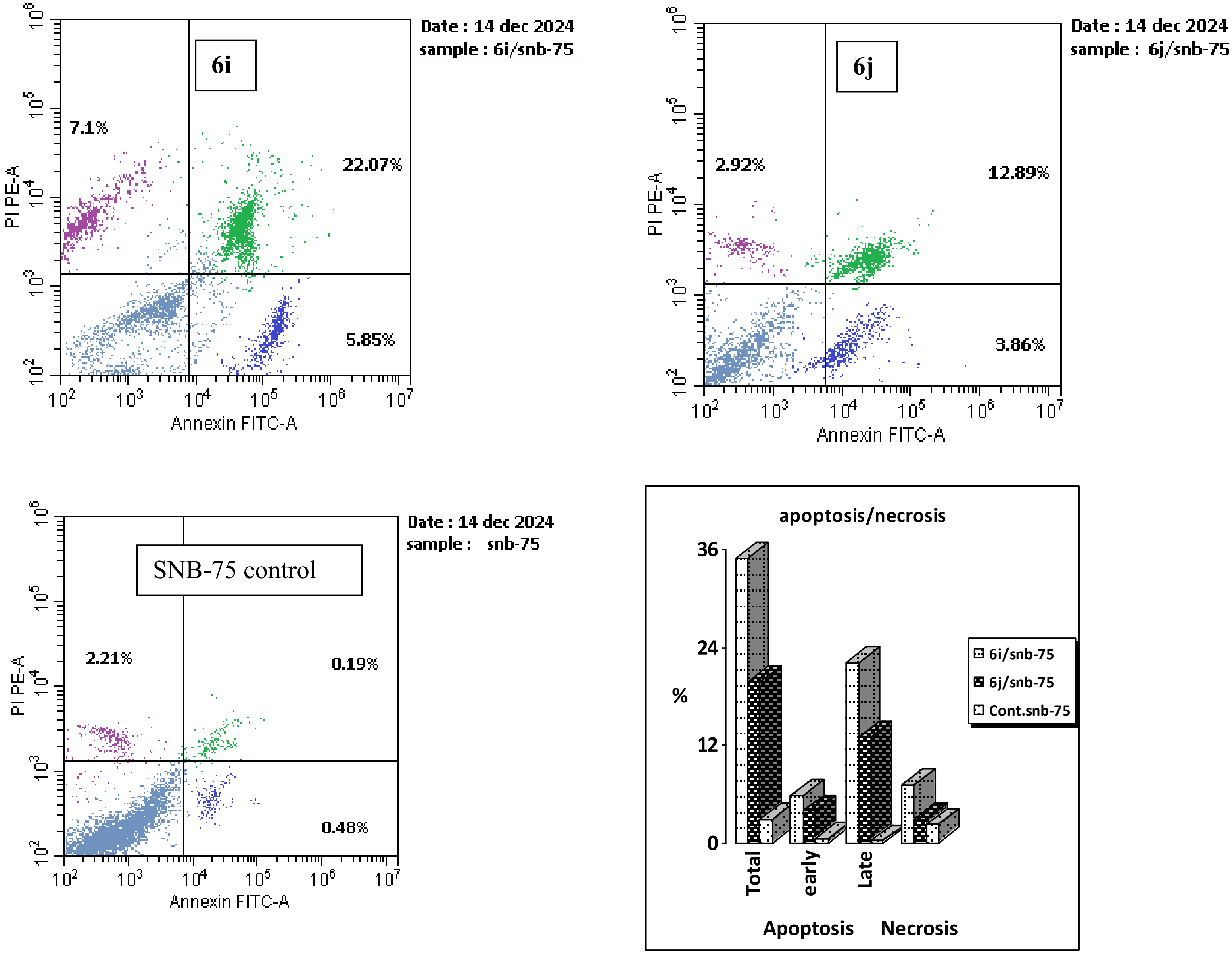

Figure 24. Assessment of Apoptotic and Necrotic Effects of compounds **6i** and **6j** on SNB-75 cell line (attempt1).

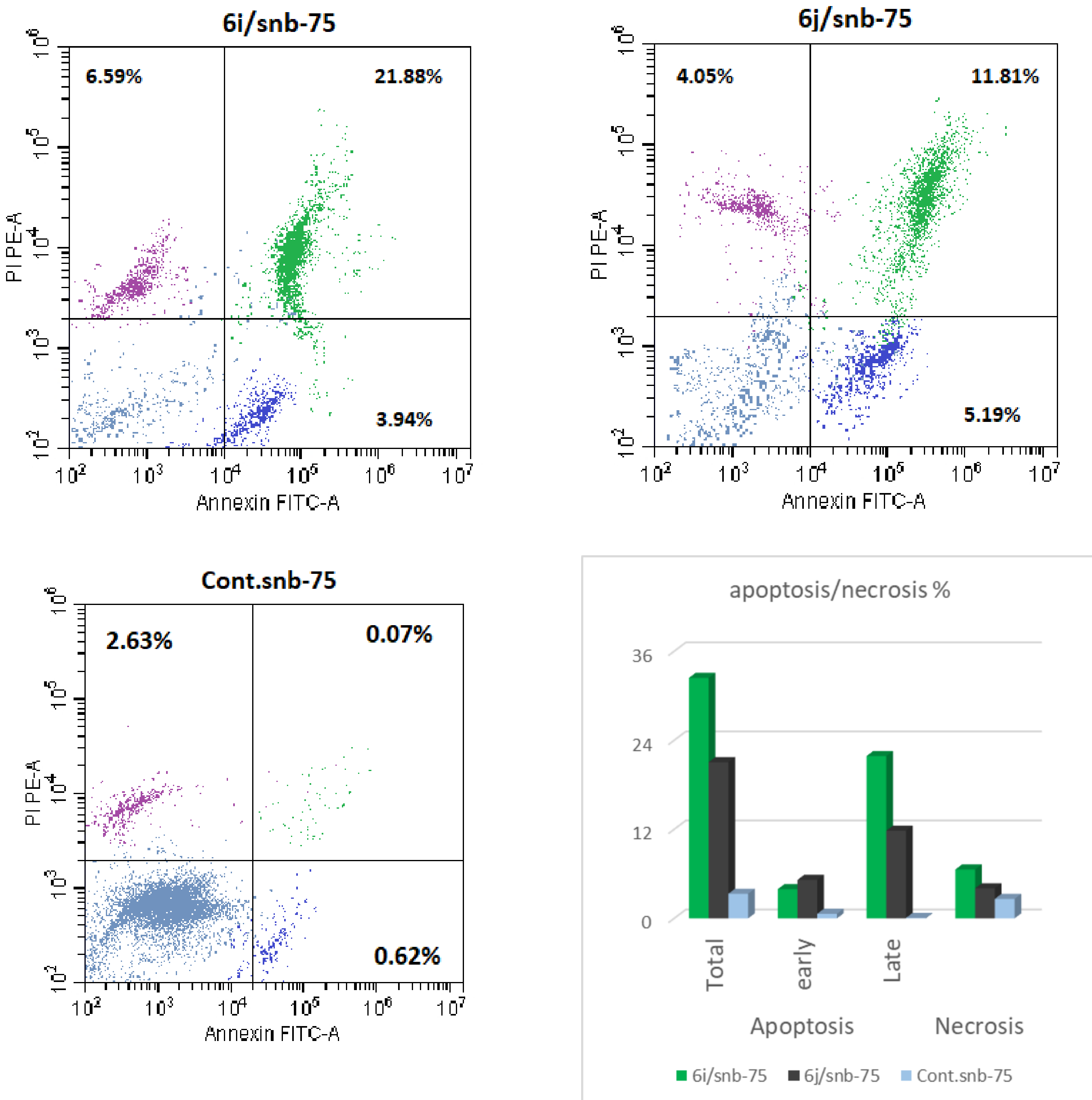

Figure 25. Assessment of Apoptotic and Necrotic Effects of compounds **6i** and **6j** on SNB-75 cell line (attempt2)

Insilco study

Table 1: Computer aided ADME screening of the synthesized compounds

	Parameters									
CPD ID	GI Absorption	BBB permeation	Pg-p substrate	CYP1A2 inhibitor	CYP2C19 inhibitor	CYP2C9 inhibitor	CYP2D6 inhibitor	CYP3 A4 inhibitor	Logkp (Skin permeation)	Bioavailability score
**6a**	High	Yes	Yes	Yes	Yes	No	Yes	Yes	-4.61	0.55
**6b**	High	No	Yes	Yes	Yes	No	No	Yes	-4.44	0.55
**6c**	High	No	Yes	Yes	Yes	Yes	Yes	Yes	-4.82	0.55
**6d**	High	No	Yes	No	Yes	Yes	Yes	Yes	-5.02	0.55
**6e**	High	No	Yes	Yes	Yes	No	No	Yes	-4.38	0.55
**6f**	High	No	Yes	No	Yes	No	No	No	-4.6	0.55
**6g**	High	No	No	Yes	Yes	No	No	No	-5.01	0.55
**6h**	Low	No	No	No	Yes	No	No	No	-4.03	0.55
**6i**	High	Yes	Yes	Yes	Yes	No	Yes	Yes	-5.19	0.55
**6j**	High	No	Yes	Yes	Yes	Yes	Yes	Yes	-4.85	0.55
**9a**	High	No	No	Yes	No	Yes	No	Yes	-5.56	0.55
**9b**	High	No	No	Yes	No	Yes	No	Yes	-5.77	0.55
**9c**	High	No	No	Yes	No	Yes	No	Yes	-5.6	0.55
**9d**	High	No	No	Yes	Yes	Yes	No	Yes	-5.7	0.55
**9e**	High	No	No	No	Yes	Yes	Yes	No	-4.49	0.55
**9f**	High	No	No	Yes	Yes	Yes	No	Yes	-5.54	0.55
**9g**	High	No	No	Yes	No	Yes	No	Yes	-5.58	0.55
**9h**	High	No	No	Yes	Yes	Yes	No	Yes	-5.07	0.55
**11**	High	No	No	No	No	No	No	No	-7.25	0.55
**12**	High	No	No	Yes	No	No	No	No	-5.78	0.55
**13**	High	No	No	Yes	No	No	No	No	-6.51	0.55
**Dinaciclib**	High	No	Yes	n/d	n/d	n/d	n/d	n/d	-7.36	0.55
**Larotrecitinib**	High	No	Yes	n/d	n/d	n/d	n/d	n/d	-7.71	0.55
**Milciclib**	High	No	Yes	n/d	n/d	n/d	n/d	n/d	-7.12	0.55

Table 2: Toxicity Prediction of the newly synthesized compounds

	Predicted target					
**Compound**	Hepatotoxicity	Carcinogenicity	Immunotoxicity	Mutagenicity	Cytotoxicity	Acute toxicity
**6a**	Active	Active	Inactive	Active	Inactive	Class 4
**6b**	Active	Active	Inactive	Active	Inactive	Class 4
**6c**	Active	Active	Inactive	Active	Inactive	Class 4
**6d**	Active	Active	Inactive	Active	Active	Class 4
**6e**	Active	Inactive	Inactive	Inactive	Inactive	Class 4
**6f**	Active	Inactive	Inactive	Active	Inactive	Class 4
**6g**	Active	Active	Inactive	Active	Inactive	Class 4
**6h**	Active	Active	Inactive	Active	Inactive	Class 4
**6i**	Active	Active	Inactive	Active	Inactive	Class 4
**6j**	Active	Active	Inactive	Active	Inactive	Class 4
**9a**	Active	Active	Inactive	Active	Inactive	Class 3
**9b**	Active	Active	Inactive	Active	Inactive	Class 3
**9c**	Active	Active	Inactive	Active	Inactive	Class 3
**9d**	Inactive	Active	Inactive	Active	Inactive	Class 3
**9e**	Active	Active	Inactive	Inactive	Inactive	Class 4
**9f**	Active	Active	Inactive	Inactive	Inactive	Class 4
**9g**	Active	Active	Inactive	Inactive	Inactive	Class 4
**9h**	Active	Inactive	Inactive	Inactive	Inactive	Class 4
**11**	Active	Active	Inactive	Active	Inactive	Class 4
**12**	Active	Active	Inactive	Active	Inactive	Class 4
**13**	Active	Active	Inactive	Inactive	Inactive	Class 4
**Dinaciclib**	Active	Active	Inactive	Active	Inactive	Class 4
**Larotrectinib**	Inactive	Inactive	Active	Inactive	Inactive	Class 4
**Milciclib**	Inactive	Active	Inactive	Inactive	Inactive	Class 3
